# Alternating-Polarity Electrolysis Enables Efficient Enantioselective Semipinacol Rearrangement Using Sodium Chloride

**DOI:** 10.1021/jacs.6c09845

**Published:** 2026-07-04

**Authors:** Zhijie Zhou, Qiaolin Yan, Zherui Zhang, Minhua Shao, Chaoshen Zhang, Jianwei Sun

**Affiliations:** † Department of Chemistry and the Hong Kong Branch of Chinese National Engineering Research Centre for Tissue Restoration & Reconstruction, 58207 The Hong Kong University of Science and Technology, Clear Water Bay, Kowloon, Hong Kong SAR 999077, China; ‡ Department of Chemical and Biological Engineering, The Hong Kong University of Science and Technology, Clear Water Bay, Kowloon, Hong Kong SAR 999077, China

## Abstract

Enantioselective chlorination using sustainable chloride sources has long been a pursuit in organic synthesis. Moving away from stoichiometric, corrosive, and costly electrophilic reagents, we report the use of NaCl as a green chlorine source for the enantioselective chlorination of organic molecules without the need for chemical oxidants. By leveraging electrochemical *in situ* oxidation, we demonstrate the first electricity-driven enantioselective semipinacol rearrangement. Central to this approach is a dual-organocatalyst phase-transfer system that synergistically manages both phase transfer and enantiomeric induction. Notably, the implementation of alternating current (AC) proved essential in preventing catalyst deposition on the electrode surface, enabling high efficiency at a synthetically useful scale. This work represents a rare application of AC in asymmetric electrosynthesis and its inaugural use in organocatalysis. A variety of cyclic and functionalized ketones bearing all-carbon quaternary stereocenters were synthesized with high enantioselectivity and excellent functional group compatibility. Detailed mechanistic insights supported by control experiments and cyclic voltammetry as well as DFT calculation further elucidate the catalytic cycle. This protocol establishes a practical platform for future enantioselective chlorination processes using sustainable chloride sources.

## Graphical abstract

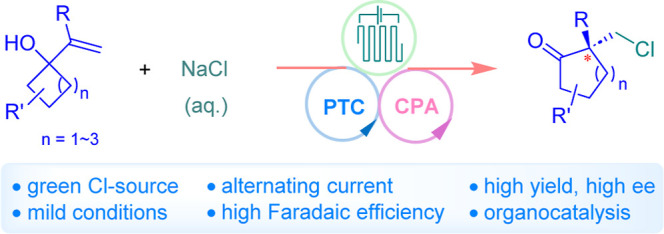

## Introduction

Organochlorides are ubiquitous structural motifs in natural products, pharmaceuticals, and polymer materials.^1–3^ Beyond their occurrence in functional molecules, they serve as indispensable intermediates in organic synthesis.^4^ In particular, chiral alkyl chlorides represent a versatile class of both functional and functionalizable units; they offer a unique balance of synthetic utility and superior stability compared to their alkyl bromide counterparts.^1–4^ Consequently, the development of enantioselective processes involving C­(sp^3^)–Cl bond formation has been a longstanding goal. However, despite the extensive success of racemic methods, asymmetric variants remain significantly underexplored.^5,6^

Over the past few decades, various ingenious chiral catalysts and systems have been developed for enantioselective chlorination, with chiral nucleophilic catalysts (e.g., amines, sulfides, and selenides) emerging as the most successful.^6^ However, these powerful electrophilic chlorination processes inevitably rely on stoichiometric electrophilic chlorine sources (e.g., NCS and its variants). These reagents are often corrosive, costly, and suffer from poor atom economy (Scheme 1a). In contrast, while nucleophilic chlorine sources like NaCl are more environmentally benign and inexpensive, they typically necessitate the use of stoichiometric chemical oxidants or substrates in high oxidation states.^7^ Consequently, these approaches often fail to fully address the fundamental concerns regarding safety and environmental impact.

**Scheme 1:**
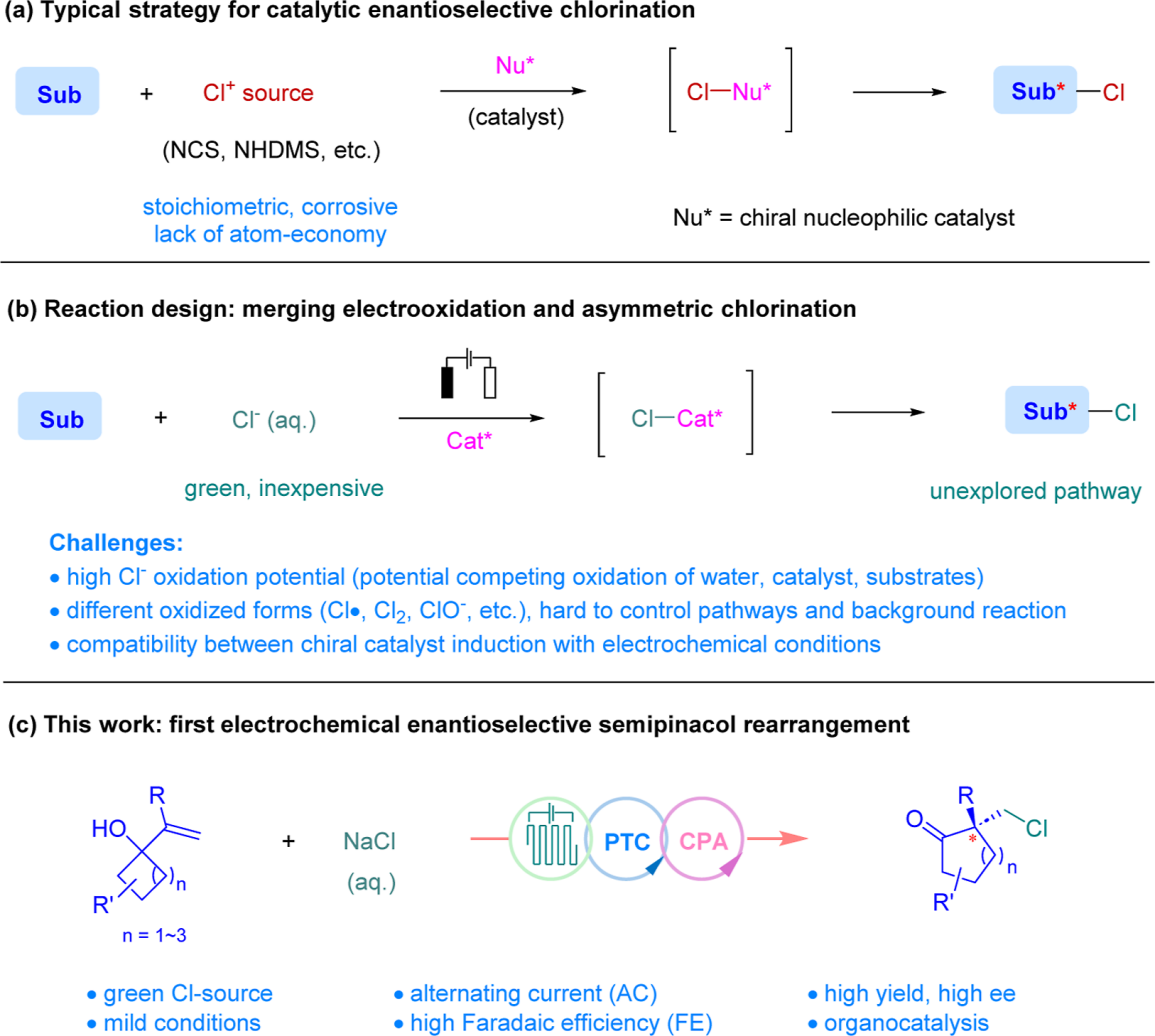
Introduction to Catalytic Enantioselective Chlorination and Reaction Design

Recently, electrochemical oxidation has emerged as a powerful alternative to chemical oxidation, offering significant opportunities for sustainable synthesis.^8^ Indeed, the anodic oxidation of chloride anions provides an attractive route to electrophilic chlorinating species, a principle utilized for decades, most notably in the industrial chlor-alkali process.^9–11^ However, its application in enantioselective chlorination has remained largely unsuccessful, hindered by several formidable challenges (Scheme 1b). First, unlike bromide oxidation,^12^ the high oxidation potential of chloride often triggers the competing oxidation of water or the chiral catalyst itself, leading to low Faradaic efficiency and catalyst decomposition. Second, anodic oxidation typically generates a nonselective mixture of reactive species (e.g., Cl^•^, Cl_2_, and ClO_n_^–^),^11^ which may participate in background pathways that bypass the chiral catalyst.^10a^ The selective generation of only one of these species for a specific pathway requires strict control of conditions in a very narrow window.^11,13^ Moreover, distinct from conventional reagents like NCS, these *in situ* generated chlorine sources (e.g., Cl_2_) lack anchoring groups (such as carbonyls) to facilitate stereocontrol through hydrogen bonding or coordination. Furthermore, there might be compatibility issues between enantioselective induction (often relying on weak interaction) with the typical polar and/or aqueous media for electrolysis. Many organocatalysts are prone to electrode deposition (passivation), which coats the electrode surface and prematurely halts the catalytic cycle. As a result, an efficient green asymmetric chlorination protocol employing electrochemical oxidation of chloride has remained an unsolved task despite its great synthetic potential.^14^

To overcome these barriers, we report the first electrochemical enantioselective semipinacol rearrangement, a type of useful organic transformation for complex molecule synthesis (Scheme 1c).^15,16^ By utilizing NaCl as a sustainable chlorine source within a dual-organocatalyst phase-transfer system, we achieved precise C­(sp^3^)–Cl bond formation. Crucially, we identified square-wave low-frequency alternating current (AC) as the enabling technology for this transformation. Unlike direct current (DC), the AC waveform effectively eliminates catalyst deposition and suppresses overoxidation, thereby restoring high Faradaic efficiency and maintaining the integrity of the chiral environment. This work represents the inaugural application of AC in organocatalytic asymmetric electrosynthesis, providing a robust platform for green enantioselective chlorination.

## Results and Discussion

We initiated our study using **1a** as the model substrate. A biphasic system was designed to partition the reaction: electrochemical oxidation occurs in a buffered aqueous phase containing NaCl as both the electrolyte and chlorine source, while the stereocontrolled semipinacol rearrangement proceeds in the organic phase. To facilitate this process and achieve enantioselective induction, we employed a chiral anion-mediated phase-transfer system, which contains the use of a chiral phosphoric acid (CPA) as a chiral anion source^17^ in combination with an achiral DABCO-derived tertiary amine as the halogen carrier. Notably, while similar systems have been established for electrophilic fluorination and bromination, they have never been demonstrated for chlorination; the corresponding Cl_2_-bound reagents remained unknown, presumably due to their inherent instability.^18^

In our preliminary evaluation of this electrochemical system (Table 1), BINOL- and [H_8_]­BINOL-derived CPAs (**A** and **B**) showed negligible catalytic activity when paired with **PTC-1**. However, the SPINOL-based CPA **C1** afforded the desired product **3a** in nearly quantitative yield with 59% ee, validating the feasibility of the dual-catalytic design (entry 3). Further screening of chiral catalysts revealed that CPA **D1**, derived from recently developed SPHENOL skeleton,^19^ provided the highest enantioselectivity (80% ee, entry 4). Varying the substituents on the backbone did not further improve the outcome (entry 5). Increasing the pH of the aqueous phase or substituting NaCl with other chloride salts (LiCl or KCl) led to a decrease in both yield and enantioselectivity (entry 6 and more details in the Supporting Information). However, maintaining the pH value in a range of 1.5–3 did not affect the reaction outcome. We also monitored the reaction progress, which indicated that the acidity of the reaction mixture and the product ee value did not change over time during the reaction (see the Supporting Information for details). The requirement of low pH for high efficiency is likely related to the narrow Cl_2_ generation window.^11^ Moreover, a less acidic aqueous phase could promote decomposition of Cl_2_ and thus lead to low Faradaic efficiency. Finally, optimization of the phase-transfer catalyst showed that **PTC-2**, bearing two ^*t*^Bu groups, significantly enhanced the enantioselectivity to 91% ee (entry 7). Alternative electrode materials were also evaluated but proved inferior to the initial setup (see the Supporting Information for details).

**Table 1:**
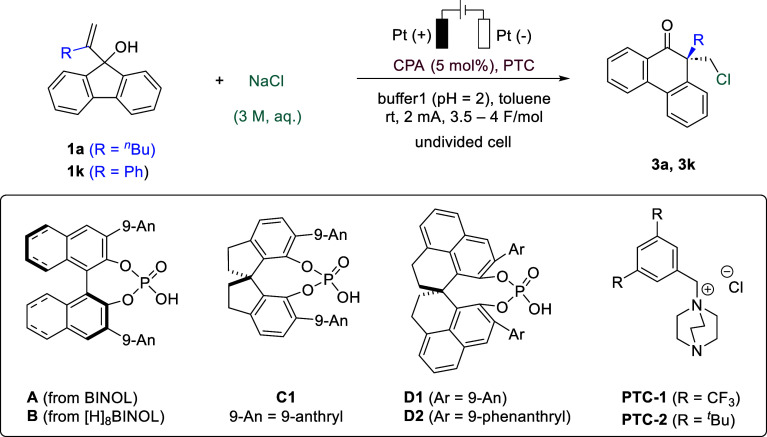
Evaluation of Reaction Conditions^a^

entry	1	CPA	PTC	yield	ee
1	**1a**	**A**	**PTC-1**	<5%	–
2	**1a**	**B**	**PTC-1**	12%	27%
3	**1a**	**C1**	**PTC-1**	>95%	59%
4	**1a**	**D1**	**PTC-1**	>95%	80%
5	**1a**	**D2**	**PTC-1**	>95%	54%
6^b^	**1a**	**D1**	**PTC-1**	25%	55%
7	**1a**	**D1**	**PTC-2**	>95%	91%
8^c^	**1a**	**D1**	**PTC-2**	27%	84%
9^c^	**1k**	**D1**	**PTC-2**	13%	95%
10^c^ ^,^ ^d^	**1k**	**D1**	**PTC-2**	93%	96%
11^c^ ^,^ ^d^	**1a**	**D1**	**PTC-2**	99%	92%
12^e^	NCS (1.5 equiv) instead of electrolysis			<5%	–

a Reaction conditions: **1a** (0.02 mmol), **CPA** (5 mol %), **PTC** (20 mol %), NaCl (3 M aq.), buffer1 (H_3_PO_4_/NaH_2_PO_4_ = 0.5 M/0.5 M, pH = 2, 2 mL), toluene (1.5 mL), Pt anode and cathode (10 mm × 25 mm × 0.1 mm), stirring speed: 1500 r/min, DC (2 mA), rt, 3.7 F/mol (1 h). Yield was determined by ^1^H NMR analysis of the crude reaction mixture with CH_2_Br_2_ as an internal standard. Ee value was determined by chiral HPLC.

b Buffer2 (NaH_2_PO_4_/Na_2_HPO_4_ = 0.5 M/0.5 M, pH = 6.4, 2 mL) instead buffer1 was used.

c Scale-up reaction with 0.3 mmol substrate: **D1** (5 mol %), **PTC-2** (10 mol %), NaCl (3 M aq.), buffer1 (H_3_PO_4_/NaH_2_PO_4_ = 0.5 M/0.5 M, pH = 2, 10 mL), toluene (7.5 mL) Pt anode and cathode (15 mm × 15 mm × 0.2 mm), stirring speed: 1500 r/min, DC (2 mA), rt, 2.7 F/mol (11 h). Isolated yield was obtained. Ee value was determined by chiral HPLC.

d Alternating current (2 mA, 0.0017 Hz) was applied.

e Reaction solvent: H_2_O/toluene = 10 mL/7.5 mL, 12 h. Other conditions were same as entry 10.

The encouraging results obtained at the initial scale (0.02 mmol) prompted us to evaluate the generality of this green electrochemical system at a more synthetically relevant scale. However, at 0.3 mmol scale, the reaction of **1a** yielded only 27% of the desired product (entry 8). A similar decline in efficiency was observed for substrate **1k** (entry 9), indicating a systemic limitation of the electrochemical setup during scale-up. In both cases, the reaction halted at low conversion; continued electrolysis resulted in a surge in cell potential (Figure 1a), signaling an invalid electrochemical process to the designed reaction occurred. A close examination of the anode surface revealed the formation of a conspicuous deposit (Figure 1c), which was subsequently identified as an analogue of the phase-transfer catalyst (see more details in the Supporting Information). While replacing the electrode temporarily resumed the conversion, the deposition persisted. We attributed the low conversion to electrode passivation and the concurrent depletion of the active phase-transfer catalyst from the solution.

**Figure 1:**
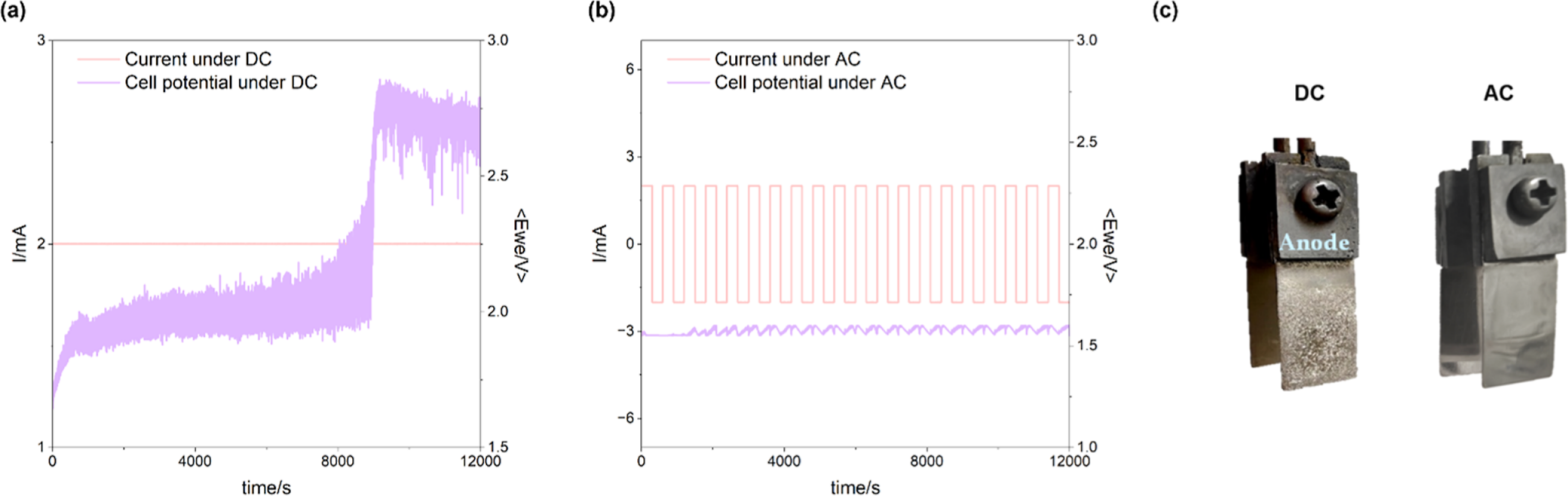
Cell potential and electrode surface after the reaction under direct current (DC) and alternating current (AC) electrolysis. (a) The curves of cell potential and electric current over time during the course of a 0.3 mmol reaction of **1k** under DC electrolysis. (b) The curves of cell potential and electric current over time during the course of a 0.3 mmol reaction of **1k** under pulsed AC electrolysis. (c) The appearance of the anodes after DC and AC electrolysis. Deposition of white solids was observed at the anode surface after DC (but not AC) electrolysis.

To circumvent this, we envisioned that periodically reversing the current direction (effectively switching the anode and cathode) might prevent the accumulation of this deposit.^20,21^ To our delight, the implementation of square-wave low-frequency AC completely resolved the issue, affording full conversion and excellent enantioselectivity at 0.3 mmol scale. Under these conditions, electrode deposition was eliminated, and the cell potential remained stable throughout the reaction (Figure 1b,c). Both the reactions of **1a** and **1k** proceeded to completion, providing the corresponding products with both high efficiency and excellent enantioselectivity (entries 10 and 11). Faradaic efficiency (FE) was calculated to be around 80%. We also evaluated other waveforms of AC for electrolysis, including sine-wave and other frequencies. They all gave high efficiency and enantioselectivity as long as alternating polarity was applied (more details in the Supporting Information). It is worth noting that while AC has recently gained traction in various catalytic systems, its utility in enantioselective synthesis remains rare. To our knowledge, this represents the inaugural application of AC in asymmetric organocatalysis.^20,21^

We also compared this electrolytic system with the conventional chlorination strategy. With the same catalysts, use of NCS did not show any chlorination reactivity (entry 12). Moreover, with Cl_2_ gas balloon as the chlorine source, high chemical yield was observed, but the enantioselectivity was mediocre (vide infra, Figure 2a). These results underscore that the advantages of this biphasic electrochemical system extend beyond the use of a sustainable chlorine source; it enables a level of stereocontrol unattainable by traditional methods, likely due to the controlled, *in situ* generation of active chlorine which suppresses racemic background pathways.

**Figure 2:**
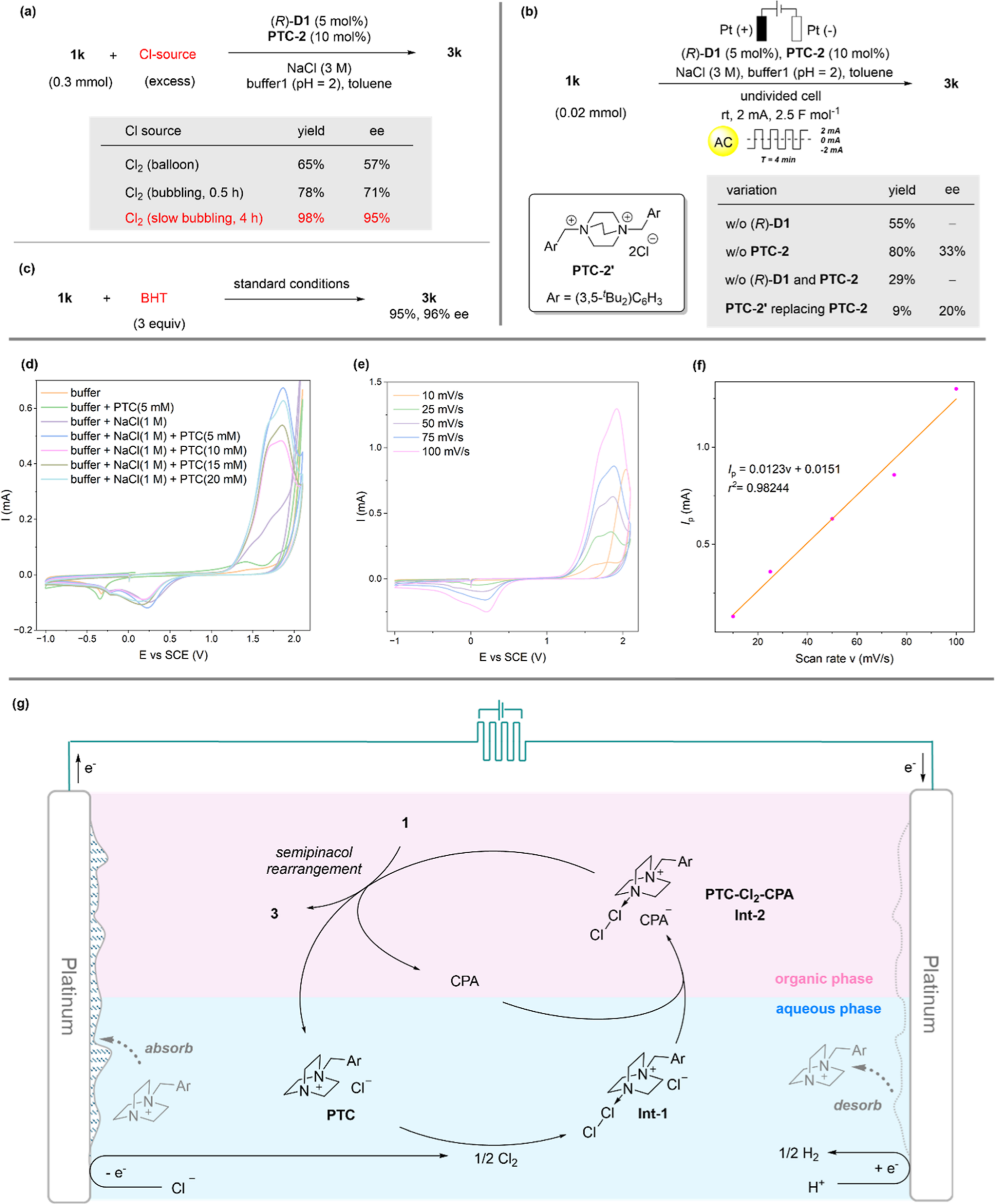
Control experiments, CV analysis and proposed mechanism (for half-period). (a) Control reactions using stoichiometric amounts Cl_2_ gas. (b) Control experiments in the absence of **PTC-2** and/or (*R*)-**D1** and using **PTC-2′** in place of **PTC-2**. (c) Effect of BHT. (d) Cyclic voltammetry studies of solutions containing different amounts of NaCl and **PTC-2**. (e) Cyclic voltammetry studies of the buffered solution of 1 M NaCl and 20 mM **PTC-2** under different scan rates. (f) A linear relationship was observed between oxidation current *I*_p_ and scan rate *v*. (g) Proposed mechanism.

The green electrolytic protocol proved highly versatile and general, facilitating the enantioselective chlorinative rearrangement of a broad range of substrates (Scheme 2). Alkenes bearing various alkyl and aryl substituents reacted smoothly to afford the corresponding ketones containing all-carbon quaternary stereocenters at the α-position, a pivotal structural motif in organic synthesis. The mild reaction conditions tolerated a diverse array of functional groups, including halides, nitriles, silyl ethers, esters, and phthalimides.

**Scheme 2:**
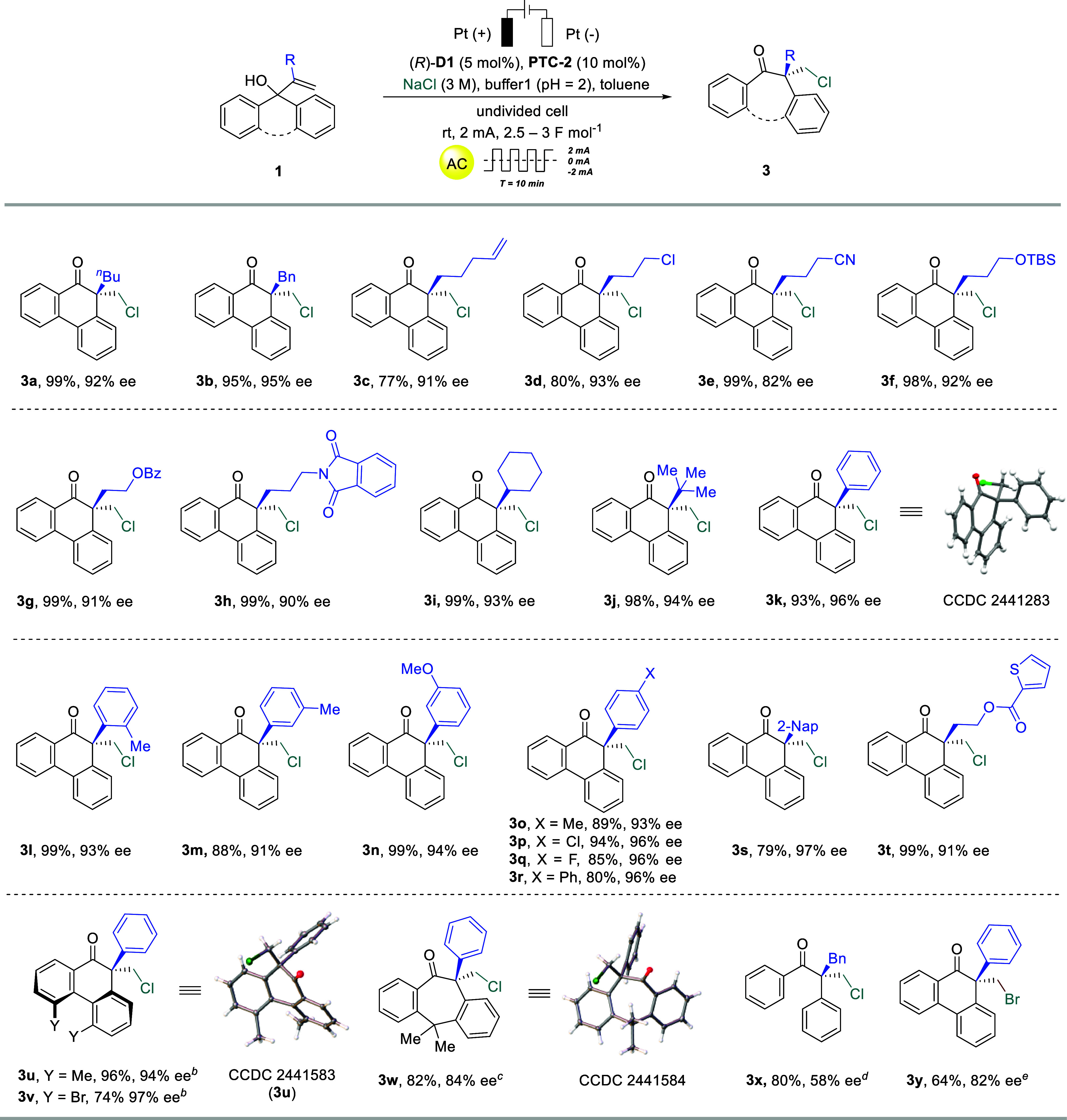
Evaluation of Reaction Scope for Electrochemical Asymmetric Semipinacol Rearrangement^a^

a Reaction conditions: **1** (0.3 mmol), (*R*)-**D1** (5 mol %), **PTC-2** (10 mol %), NaCl (3 M aq.), buffer1 (10 mL), toluene (7.5 mL), Pt anode and cathode (15 mm × 15 mm × 0.2 mm), stirring speed: 1500 rpm, square-wave low-frequency AC (2 mA, 0.0017 Hz), rt, 10 – 12 h (2.5–3.0 F·mol^–1^).
b 20:1 dr.
c (*R*)-**D2** and PhCl were used in place of (*R*)-**D1** and toluene, respectively.
d (*R*)-**C2** and **PTC-7** were used (structures in the Supporting Information).
e NaBr was used in place of NaCl.

Furthermore, this protocol exhibited excellent chemoselectivity; for instance, product **3c** was obtained as the major product, leaving the additional isolated olefin unit intact. Oxidizable heteroaromatic rings, such as thiophene, were also compatible, with **3t** being isolated in quantitative yield. Notably, the presence of bulky substituents at the reactive center, such as cyclohexyl (**3i**) and *tert*-butyl (**3j**), did not diminish the high efficiency of the transformation.

This protocol is also capable of simultaneously generating two stereogenic elements. For instance, both axial and central chirality were established in **3u** and **3v** with high enantio- and diastereoselectivity. Additionally, with minor modifications to the reaction conditions, this methodology was extended to the ring expansion of the six-membered substrate **1w**, yielding the enantioenriched benzene-fused seven-membered scaffold **3w**, a structural motif frequently encountered in bioactive molecules and pharmaceuticals.^22^ With a different combination of CPA and PTC catalysts, this protocol was also capable of generating acyclic ketones (**3x**), albeit with moderate enantioselectivity. Notably, the use of NaBr in place of NaCl permitted successful extension of this process to bromination (**3y**). However, replacing NaCl with NaI did not lead to successful iodination. Internal olefins and some other substrates, such as unsymmetrical ones, were also evaluated. They generally gave the desired products in high yield, but with diminished enantioselectivity (see the Supporting Information for details). Finally, the absolute configurations of **3k**, **3u**, and **3w** were unambiguously confirmed by X-ray crystallographic analysis.

Our electrochemical protocol could also be applied in the chlorinative ring expansion of vinyl-substituted cyclobutanols, leading to diverse chiral cyclopentanones, a family of broadly useful structural motifs (Scheme 3).^23^ With the 3-substituted prochiral cyclobutanols, two stereogenic centers were established in a single step with high enantio- and diastereoselectivity. The relative and absolute configurations of these products were unambiguously assigned by X-ray crystallography (details in the Supporting Information). Interestingly, when cyclobutyl pivalates were employed as substrates, treating the crude reaction mixture with Et_3_N resulted in *in situ* elimination to directly afford enantioenriched cyclopentenones (**4l** and **4m**).

**Scheme 3:**
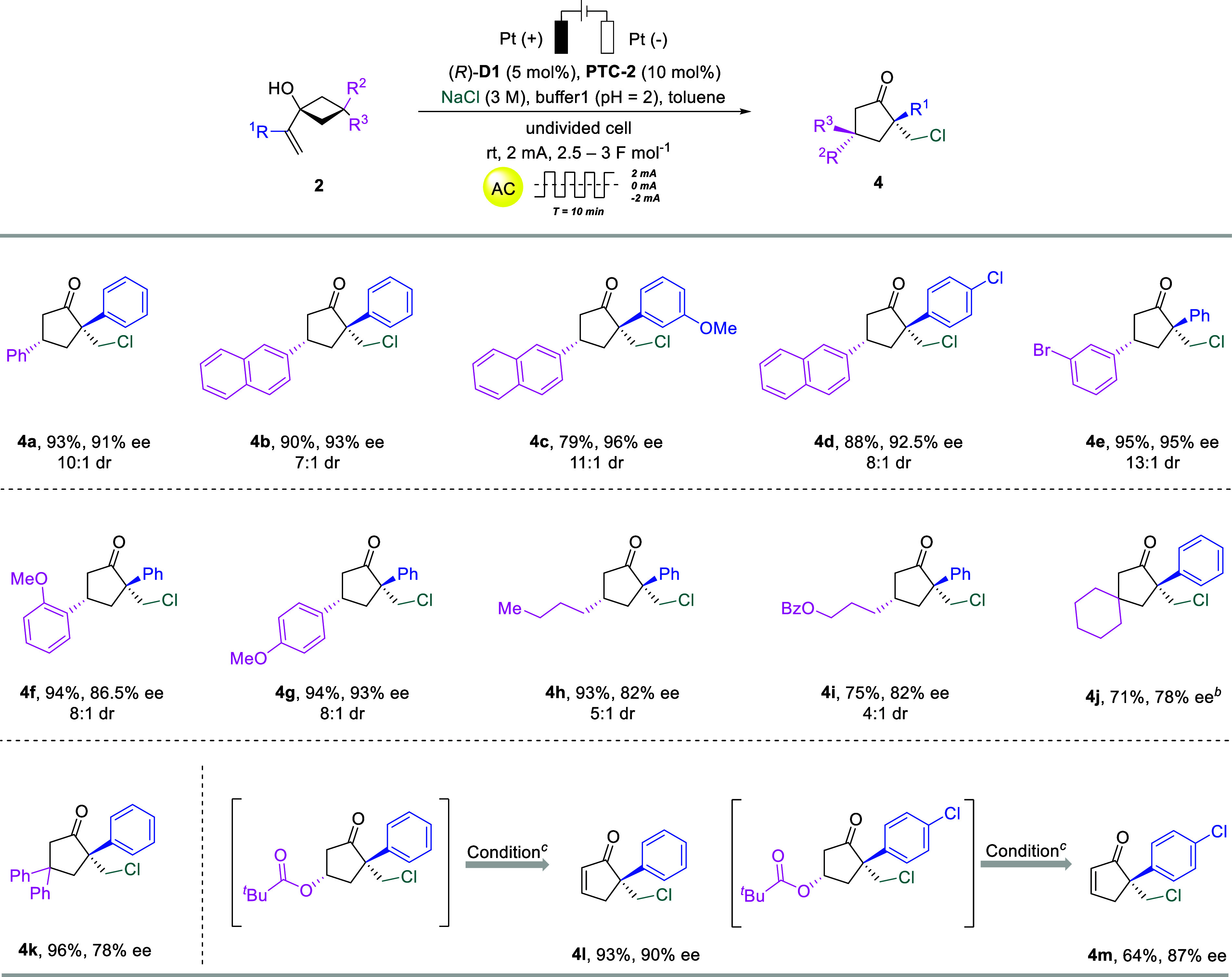
Evaluation of Reaction Scope for Synthesis of Chiral Cyclopentanones and Cyclopentenones^a^

a Reaction conditions: **2** (0.3 mmol), (*R*)-**D1** (5 mol %), **PTC-2** (10 mol %), NaCl (3 M aq.), buffer1 (10 mL), toluene (7.5 mL), Pt anode and cathode (15 mm × 15 mm × 0.2 mm), stirring speed: 1500 rpm, square-wave low-frequency AC (2 mA, 0.0017 Hz), rt, 10 – 12 h (2.5–3.0 F·mol^–1^).
b Run at 0 °C.
c The crude product was treated with Et_3_N (1.5 mL) and EtOAc (15 mL) before workup.

The practicality of this electrochemical protocol was further demonstrated by a 3 mmol reaction of **1k**. By employing pulsed AC at 10 mA and 0.008 Hz, we achieved an essentially quantitative synthesis of **3k** with no erosion of enantioselectivity (Scheme 4a). The resulting alkyl chloride and ketone moieties provided a versatile handle for the synthesis of various enantioenriched building blocks. For instance, treating **3k** with NaN_3_ triggered a Schmidt rearrangement, giving rise to lactam **5**, which shares the same core of the drug molecule Benazepril.^24^ A subsequent Wittig reaction of **3k** provided olefin **6** in 81% yield. Furthermore, the diastereoselective addition of MeLi or hydride afforded the corresponding alcohols **7** and **8**, respectively, with high diastereocontrol. Notably, these transformations proceeded with essentially no loss of enantiopurity, underscoring the robustness of the enantioenriched products. Additionally, the enantioenriched cyclopentenone **4l** underwent photocatalytic bromination to produce mono- and dibrominated compounds **9** and **10**, which are valuable precursors to other functionalized five-membered carbocycles (Scheme 4b). The slight loss of enantiopurity of **9** might be related to the involvement of diastereomeric intermediates that reacted with different reaction rate constants.

**Scheme 4:**
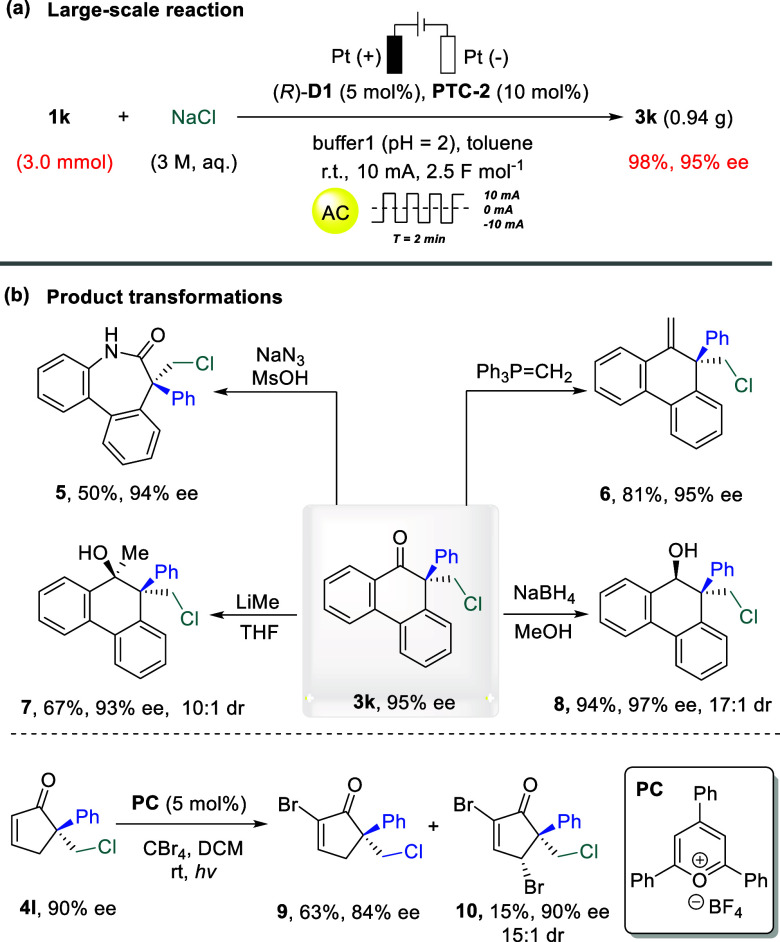
Scale–Up Reaction and Product Transformations

To elucidate the mechanism of this electrochemical process, we conducted several control experiments. First, since Cl_2_ might be generated *in situ* from electrolysis and likely served as the active chlorinating species, we evaluated its direct use for the reaction. Under otherwise identical conditions, using a Cl_2_ balloon failed to achieve comparable enantioselectivity and chemical efficiency. We hypothesized that an excess of Cl_2_ oversaturates the catalyst, triggering a significant racemic background reaction that compromises stereocontrol. To test this, we introduced Cl_2_ via slow bubbling to mimic its gradual generation during electrolysis (Figure 2a). When the bubbling was completed over a 30 min period, both the yield and enantioselectivity increased significantly, though the presence of some racemic background reaction was still evident. However, by further reducing the addition rate to a 4 h duration, we achieved both high chemical efficiency and excellent enantioselectivity. These results closely mirrored the electrochemical outcomes and corroborated the distinct advantage of controlled *in situ* reagent generation. These observations implied that the key requirement for high enantioselectivity is the controlled supply of a low steady-state concentration of Cl_2_ to avoid oversaturation of the PTC (Figure 2a).

The individual roles of the CPA and PTC were also investigated (Figure 2b). In the absence of either (*R*)-**D1** or **PTC-2**, the reactions of **1k** still proceeded, albeit at a reduced rate. A qualitative comparison of the reaction rates with and without (*R*)-**D1** was also investigated (see the Supporting Information for details). Conversely, the absence of both catalysts resulted in negligible conversion, confirming that both components are essential for accelerating the transformation. The ability of the CPA to independently promote the enantioselective pathway, though less efficiently, suggests a mechanism involving CPA-mediated activation of either the substrate or the chlorine species. Furthermore, using **PTC-2′** led to minimal product formation, consistent with the amine functionality acting as the Cl_2_ carrier. Notably, the enantioselective influence of CPA and the chemical efficiency were both reduced in this instance, suggesting that the bis­(ammonium) dication may sequester the CPA anion, likely through competitive phase transfer or precipitation. Finally, the addition of BHT to the standard reaction had no detrimental effect, indicating that the process does not proceed via a radical pathway. This distinguishes our protocol from the electrochemical chlorination of olefins reported by Lin and co-workers,^10a^ and it is also consistent with the chemoselectivity observed for substrate **3c** (Figure 2c).

Cyclic voltammetry (CV) experiments were conducted to gain deeper insights into the electrochemical mechanism. Initially, a blank buffer solution exhibited no significant oxidation peaks, with the onset of water oxidation occurring at approximately 2.0 V (vs SCE). Upon the addition of NaCl (1 M), an oxidation peak appeared at ∼1.5 V, accompanied by a corresponding reduction peak at ∼0.25 V; these features are indicative of the quasi-reversible oxidation of Cl^–^.^25^ Subsequently, the addition of **PTC-2** (5 mM) to the NaCl solution triggered a dramatic surge in the anodic current at 1.5 V. This enhancement suggests that **PTC-2** effectively binds to the Cl_2_ generated at the anode, thereby producing a catalytic current by facilitating its removal from the interface and driving the catalytic oxidation.

This observation further supports the role of the phase transfer catalyst as an efficient halogen carrier within the biphasic system. To further elucidate this behavior, we increased the loading of **PTC-2** in expectation of further current enhancement. Surprisingly, the current dropped considerably at 10 mM loading compared to the 5 mM trial. Subsequent increases to 15 mM and 20 mM resulted in some enhancements, but the peak currents remaining lower than those observed at 5 mM. This anomalous trend is consistent with our earlier observation of electrode passivation; at higher concentrations, the deposition of the **PTC-2** analog on the anode might be more pronounced, thereby impeding the catalytic current (Figure 2d).

Finally, we examined the influence of the scan rate on the system (Figure 2e). As the scan rate increased from 10 mV/s to 100 mV/s, the peak oxidation current (*I*_p_) rose accordingly. A linear relationship was observed between *I*_p_ and the scan rate (*v*), rather than the square root of the scan rate (*v* ^1/2^) (Figure 2f). This linear correlation is a classic indicator of an adsorption-controlled process, further corroborating that the deposition of species onto the electrode surface dictates the electrochemical behavior of this system.^26^

Based on the above results, a proposed catalytic pathway is illustrated in Figure 2g. In the aqueous phase, anodic oxidation of chloride generates Cl_2_, intercepted by the amine moiety of the phase-transfer catalyst to form **Int-1** as a donor–acceptor complex. This adduct is subsequently transported into the organic phase upon exchange with the chiral phosphate anion as a multicomponent complex **Int-2**. This assembly serves as the active chiral electrophilic chlorine source, triggering the enantioselective semipinacol rearrangement. Following the formation of the enantioenriched product, the PTC and CPA catalysts are regenerated and partition back into the aqueous phase for the next catalytic cycle. Although attempts to isolate and characterize **Int-1** or **Int-2** were fruitless, we performed density functional theory (DFT) calculations, which revealed that the formation of a loosely associated PTC^•^Cl_2_ species is thermodynamically more favorable than forming a covalent N–Cl bond (e.g., [R_3_N–Cl]^+^Cl^–^) in both organic and aqueous phases (see more details in the Supporting Information).

Under direct current conditions, the PTC or its derivatives gradually accumulate on the anode surface, leading to electrode passivation and a concomitant surge in cell potential. This phenomenon ultimately halts electrolysis and accounts for the low conversions observed in large-scale DC experiments. In contrast, the application of AC involves periodic polarity reversal; the anode periodically functions as a cathode, which facilitates the resolubilization of the deposited PTC back into the bulk solution. This dynamic surface cleaning maintains the integrity of the electrode interface and ensures a continuous efficient catalytic cycle.

## Conclusion

In summary, we have established the first organocatalytic electrochemical enantioselective chlorination protocol founded on the in situ oxidation of chloride. This work constitutes the inaugural demonstration of an electricity-driven enantioselective semipinacol rearrangement, offering a sustainable and atom-economical route to high-value synthetic motifs. Key to this success is a synergistic dual-organocatalyst phase-transfer system that orchestrates both interfacial transport and enantiomeric induction. While standard direct current proved sufficient for small-scale transformations, scale-up efforts identified a critical bottleneck in electrode passivation. Mechanistic studies, including the observation of a linear relationship between peak current and scan rate, confirmed that the process is dictated by catalyst adsorption onto the electrode surface. We demonstrate that square-wave low-frequency alternating current effectively circumvents this limitation, enabling robust, reproducible, and highly efficient synthesis even at gram scale. This study represents a rare application of AC in asymmetric electrosynthesis and its first implementation in the field of organocatalysis. With our SPHENOL-derived CPA as the optimal catalyst, this protocol exhibits broad substrate generality, affording a diverse array of cyclic and functionalized ketones bearing all-carbon quaternary stereocenters with high enantioselectivity and excellent functional group compatibility. The synthetic utility of the resulting products was further showcased through the divergent transformations into other sophisticated enantioenriched building blocks. This work provides a high-performance, practical platform for advancing enantioselective chlorination using sustainable chlorine sources.

## Supplementary Material



## Notes

Additional experimental and computational details, and spectroscopic data of all compounds (PDF)
