# Supplementary material for: Alternating-Polarity Electrolysis Enables Efficient Enantioselective Semipinacol Rearrangement Using Sodium Chloride
Source: J Am Chem Soc. 2026 Jul 4;148(27):29464–74. doi: 10.1021/jacs.6c09845 (PMC13383718; doi:10.1021/jacs.6c09845)
Supplement: Supplementary file 1 [file ja6c09845_si_001.pdf]

Supporting Information

Alternating-Polarity Electrolysis Enables Efficient Enantioselective Semipinacol Rearrangement Using Sodium Chloride

Zhijie Zhou,[a] Qiaolin Yan,[a] Zherui Zhang,[a] Minhua Shao,[b]
Chaoshen Zhang,[a] and Jianwei Sun[ab]*

[a]*Department of Chemistry and the Hong Kong Branch of Chinese National Engineering Research Centre for Tissue Restoration & Reconstruction, The Hong Kong University of Science and Technology, Clear Water Bay, Kowloon 999077, Hong Kong SAR, China*

[b]*Department of Chemical and Biological Engineering, The Hong Kong University of Science and Technology, Clear Water Bay, Kowloon, Hong Kong SAR 999077, China*

Table of Contents

I. General Information ... S-2
II. Electrochemical Setup ... S-3
III. Synthesis of Substrates ... S-4
IV. Synthesis of the Phase-Transfer Catalysts ... S-30
V. Reaction Condition Optimization ... S-32
VI. Electrochemical Chlorinative Semipinacol Rearrangement ... S-41
VII. Mechanistic Studies ... S-70
VIII. Large-Scale Synthesis and Product Derivatizations ... S-81
IX. Determination of Products Stereochemistry ... S-88
X. DFT Calculations ... S-95
XI. References ... S-110
NMR Spectra and HPLC Traces

I. General Information

Flash column chromatography was performed over silica gel (200-300 or 300-400 mesh) purchased from Qindao Haiyang Co., China or SiliCycle® Inc., Canada. All air or moisture sensitive reactions were conducted in oven-dried glassware under nitrogen atmosphere using anhydrous solvents. Tetrahydrofuran was distilled from sodium/benzophenone. Anhydrous *N*,*N*-dimethyl formamide, dichloromethane, methanol, toluene, diethyl ether, acetonitrile, and *n*-hexane were purified by the Innovative® solvent purification system. Other anhydrous solvents were purchased from Sigma-Aldrich®, J&K® and Energy® and used as received. The phosphate buffer (pH = 7) was purchased from ThermoFisher® and other buffer solutions were homemade by mixing the corresponding phosphate salts in aqueous solution. Chemicals were purchased from commercial suppliers, such as Sigma-Aldrich®, J&K®, Energy® and used without further purification unless otherwise stated. NMR spectra were recorded with a Bruker AVII, AVIII or NEO 400 spectrometer at 400 MHz (^{1}H NMR), 101 MHz (^{13}C NMR), 162 MHz (^{31}P NMR) and 376 MHz (^{19}F NMR). Chemical shifts (δ values) were reported in ppm using residue solvent peaks as an internal standard (^{1}H NMR: $CDCl_3$ at 7.26 ppm, DMSO-d_6 at 2.54 ppm, CD_3CN at 1.94 ppm and ^{13}C NMR: $CDCl_3$ at 77.00 ppm, DMSO-d_6 at 39.5 ppm). Data for ^{1}H NMR were recorded as follows: chemical shift (δ, ppm), multiplicity (s = singlet; d = doublet; t = triplet; q = quarter; p = pentet; sept = septet; m = multiplet; br = broad), coupling constant (Hz), integration. The mass spectra were collected from a Waters Xevo G2-XS Tof, with ESI source; or a Waters GCT premier with EI/CI source. Optical rotations were measured on a JASCO P-2000 polarimeter or an AUTOPOL I Automatic polarimeter with $[\alpha]_D$ values reported in degrees; concentration (c) is in 10 mg/mL. Enantioselectivities were recorded on an Agilent HPLC instrument, using a chiral stationary phase column (Daicel CHIRALPAK® AD-3, AS-3, IB N-3, IC-3, ID-3, IE-3, CHIRALCEL® OD-3, OX-3, OZ-3). The methods were calibrated with the corresponding racemic mixtures.

II. Electrochemical Setup

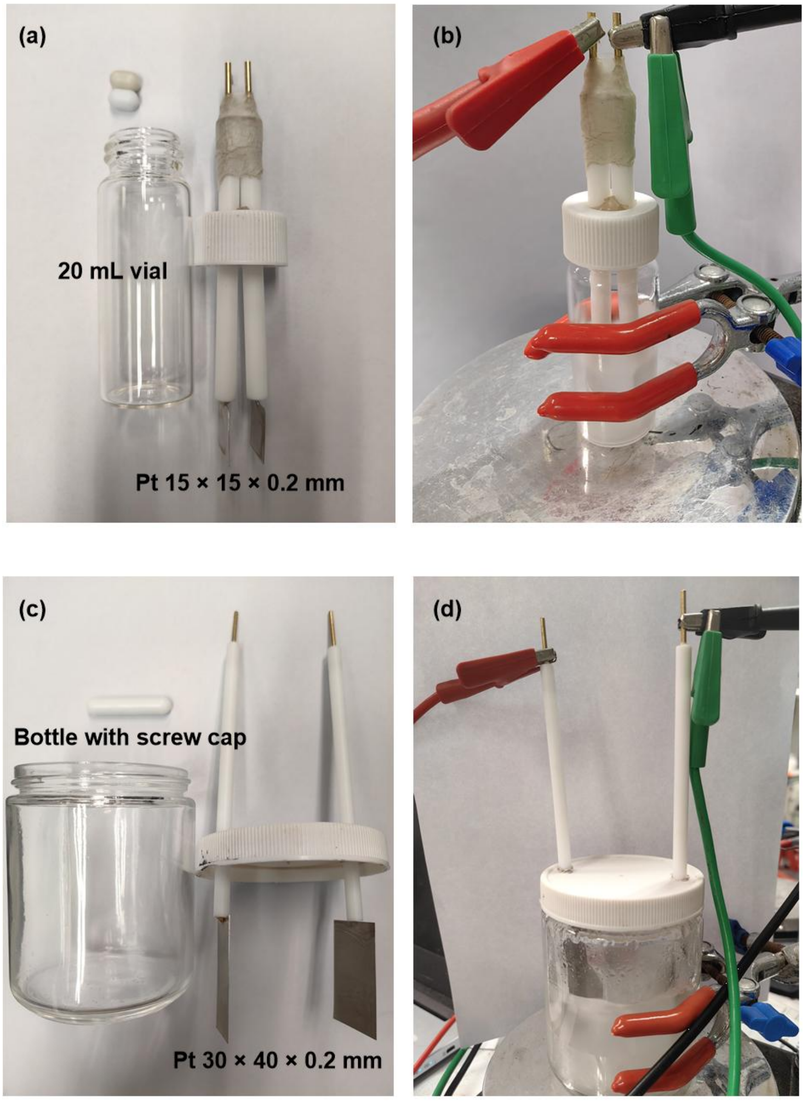

Figure S1. Electrochemical setup. (a) 20-mL vial, platinum anode, and cathode (15 mm × 15 mm × 0.2 mm). (b) Electrochemical setup for a 0.3-mmol scale reaction. (c) 200-mL cylindrical bottle with a screw cap, a platinum cathode, and an anode (30 mm × 45 mm × 0.2 mm). (d) Electrochemical setup for a 3.0-mmol scale reaction.

III. Synthesis of Substrates

Substrates **1x**[1] and **2k**[2] are known compounds. They were prepared according to the literature procedures.

General Procedure A

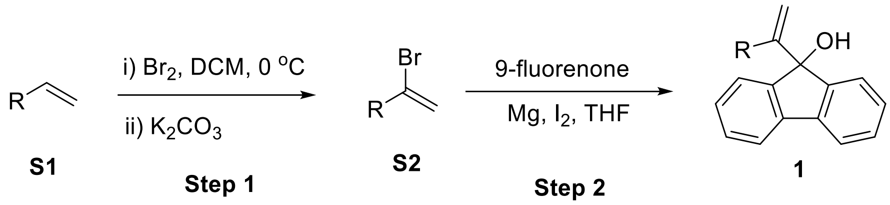

Step 1. At 0 °C, to a solution of **S1** (1.0 equiv) in DCM (0.5 M) was added bromine (1.2 equiv) dropwise. After stirring at 0 °C for 1 h, a saturated aqueous solution of Na_2SO_3 was added to quench the reaction. The mixture was stirred for another 15 min before the layers were separated. The aqueous layer was extracted with DCM. The combined organic layers were dried over anhydrous Na_2SO_4, filtered, and concentrated. The crude dibromide was used directly without further purification.

To a solution of the crude dibromide in THF/MeOH (v/v = 1:1, 0.33 M) was added K_2CO_3 (2.0 equiv). The mixture was allowed to stir at room temperature overnight before it was quenched with brine. Next, EtOAc (20 mL) was added to extract the product. The layers were separated and the aqueous layer was extracted with another EtOAc (40 mL). The combined organic layers were dried over anhydrous Na_2SO_4, filtered, and concentrated. The residue was purified by flash column chromatography on silica gel to afford the corresponding product **S2**.

Step 2. Under N_2, to an oven-dried Schlenk tube were added dry magnesium turnings (2.8 equiv) and a small grain of iodine. A small amount (about 1 mL) of a solution of **S2** (2.5 equiv, 1 M in anhydrous THF) was added to trigger the reaction (note: heat until the brown color of iodine disappeared) and the rest solution was added dropwise to keep the mixture in mild boiling. After stirring at room temperature for another 1

h, it was added to a solution of 9-fluorenone (1.0 equiv) in anhydrous THF (0.5 M) dropwise under N_2 atmosphere at 0 °C. The reaction was allowed to warm to room temperature and stirred for another 1 – 3 h before it was quenched by a saturated aqueous solution of NH_4Cl. The layers were separated and the aqueous layer was extracted by EtOAc (10 mL × 3). The combined organic layers were dried over anhydrous Na_2SO_4, filtered, and concentrated. The residue was purified by flash column chromatography on silica gel to afford the desired product **1**.

General Procedure B

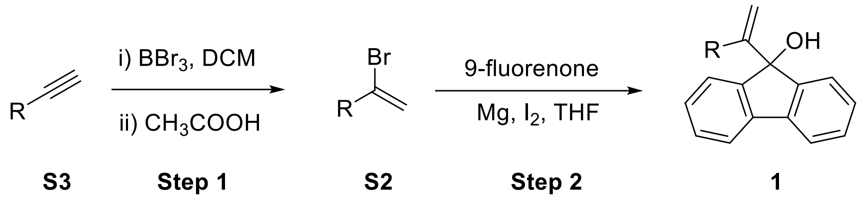

Step 1. Under N_2, to a solution of BBr_3 (0.5 equiv) in dry DCM (0.5 M) was added alkyne **S3** (1.0 equiv) dropwise at -78 °C. The mixture was then allowed to warm to room temperature and stirred for 3 h before acetic acid (6.0 equiv) was added slowly. After stirring at the same temperature for another 1 h, the solid Na_2CO_3 was slowly added to consume the excess acetic acid. Brine was then added and the organic layer was separated. The aqueous layer was extracted with DCM (20 mL × 3). The combined organic layers were dried over anhydrous Na_2SO_4, filtered, and concentrated. The residue was purified by flash column chromatography on silica gel to afford the corresponding product **S2**.

Step 2 was run by the same procedure described in the General Procedure A.

General Procedure C

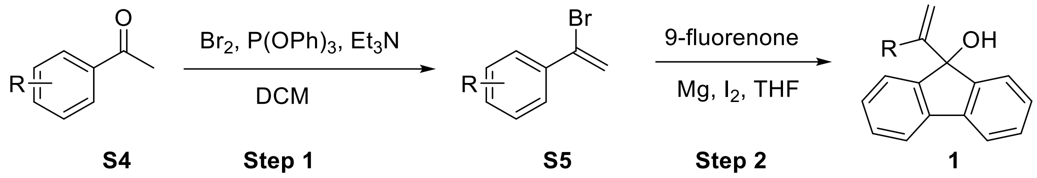

Step 1. Under N_2, to a solution of $P(OPh)_3$ (1.1 equiv) in DCM (0.4 M) was added bromine (1.2 equiv) dropwise at -78 °C. After stirring at -78 °C for 10 min, Et_3N (1.3 equiv) was added, and the mixture was stirred for another 10 min. Then, **S4** (1.0 equiv) was added, and the mixture was allowed to warm to room temperature and stirred overnight following by heating to reflux for 2 h. The reaction was quenched by a solution of saturated aqueous Na_2SO_3. Brine was added and the layers were separated. The aqueous layer was extracted with DCM (20 mL × 3). The combined organic layers were dried over anhydrous Na_2SO_4, filtered, and concentrated. The residue was purified by flash column chromatography on silica gel to afford the corresponding product **S5**.

Step 2 was run by the same procedure described in the General Procedure A.

General Procedure D

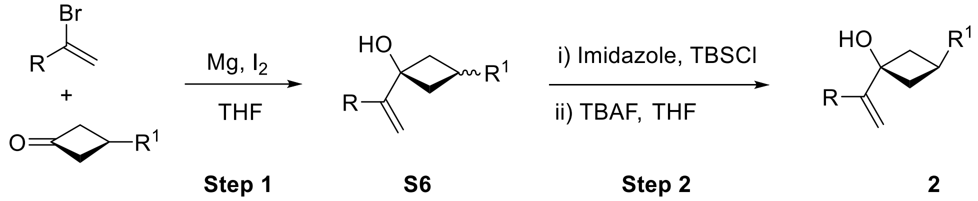

Step 1. Under N_2, to an oven-dried Schlenk tube were added dry magnesium turnings (2.8 equiv) and a small grain of iodine. A small amount (about 1 mL) of a solution of *gem*-substituted bromoethene (2.5 equiv, 1 M in anhydrous THF) was added to trigger the reaction (note: heat until the brown color of iodine disappeared) and the rest solution was added dropwise to keep the mixture in mild boiling. After stirring at room temperature for another 1 h, it was added to a solution of 3-substituted cyclobutanone (1.0 equiv) in anhydrous THF (0.5 M) dropwise under N_2 atmosphere.

The reaction was allowed to stir at room temperature for another 1 – 3 h before it was quenched by a saturated aqueous solution of NH_4Cl. The layers were separated and the aqueous layer was extracted by EtOAc (10 mL × 3). The combined organic layers were dried over anhydrous Na_2SO_4, filtered, and concentrated. The residue was purified by flash column chromatography on silica gel to afford the desired product **S6** as *cis*- and *trans*- mixtures.

Step 2. To a solution of **S6** in dry DCM (0.2 M) was added imidazole (4.0 equiv) and *tert*-butyldimethylsilyl chloride (3.0 equiv) sequentially. The reaction mixture was stirred overnight at room temperature before it was quenched by brine. The layers were separated and the aqueous layer was extracted by DCM (10 mL × 3). The combined organic layers were dried over anhydrous Na_2SO_4, filtered, and concentrated. The residue was purified by flash column chromatography on silica gel to afford the *tert*-butyldimethylsilyl protected *cis*-**S6**, which was then dissolved in THF (0.1 M) and treated with TBAF (1.1 equiv, I M in THF) at 0 °C. The reaction mixture was allowed to warm to room temperature and stirred overnight before it was quenched by a saturated aqueous solution of NH_4Cl. The layers were separated and the aqueous layer was extracted by EtOAc (10 mL × 3). The combined organic layers were dried over anhydrous Na_2SO_4, filtered, and concentrated. The residue was purified by flash column chromatography on silica gel to afford the desired product **1** as *cis*-isomer.

*Note: When R^1 was OPiv, **Step 2** was omitted as the corresponding **S6** was obtained as a single isomer.*

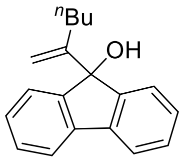

1a

9-(Hex-1-en-2-yl)-9*H*-fluoren-9-ol (1a) was prepared as a colorless oil according to the

General Procedure B (eluent: *n*-hexane/EtOAc = 10:1, 72% yield over two steps).

^{1}H NMR (400 MHz, $CDCl_3$) δ 7.64 (dd, J_1 = 7.1 Hz, J_2 = 1.4 Hz, 2H), 7.39 – 7.35 (m, 4H), 7.30 – 7.26 (m, 2H), 5.84 (d, J = 1.1 Hz, 1H), 5.18 – 5.17 (m, 1H), 2.21 (s, 1H), 1.40 – 1.37 (m, 2H), 1.20 – 1.12 (m, 2H), 1.00 (dq, J_1 = 14.2 Hz, J_2 = 7.2 Hz, 2H), 0.64 (t, J = 7.3 Hz, 3H) ppm.

^{13}C NMR (101 MHz, $CDCl_3$) δ 149.4, 148.5, 140.3, 129.0, 128.1, 123.9, 120.0, 109.0, 84.7, 30.3, 30.2, 22.1, 13.7 ppm.

HRMS (ES-) Calcd for $C_{19}H_{19}O^-$ [M-H]$^-$: 263.1441, Found: 263.1440.

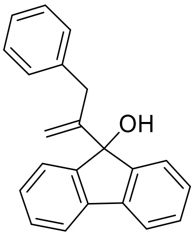

1b

9-(3-Phenylprop-1-en-2-yl)-9*H*-fluoren-9-ol (1b) was prepared as a white solid according to the General Procedure B (eluent: *n*-hexane/EtOAc = 10:1, 66% yield after two steps).

^{1}H NMR (400 MHz, $CDCl_3$) δ 7.66 – 7.64 (m, 2H), 7.40 – 7.36 (m, 4H), 7.30 – 7.26 (m, 2H), 7.14 – 7.06 (m, 3H), 6.82 – 6.80 (m, 2H), 5.91 (d, J = 1.0 Hz, 1H), 4.91 (d, J = 1.4 Hz, 1H), 2.71 (s, 2H), 2.25 (d, J = 2.1 Hz, 1H) ppm.

^{13}C NMR (101 MHz, $CDCl_3$) δ 149.7, 148.0, 140.4, 139.4, 129.2 (2C), 128.3, 127.9, 125.8, 124.1, 120.1, 112.1, 84.6, 37.7 ppm.

HRMS (CI+) Calcd for $C_{22}H_{18}O^+$ (M^+): 298.1358, Found: 298.1363.

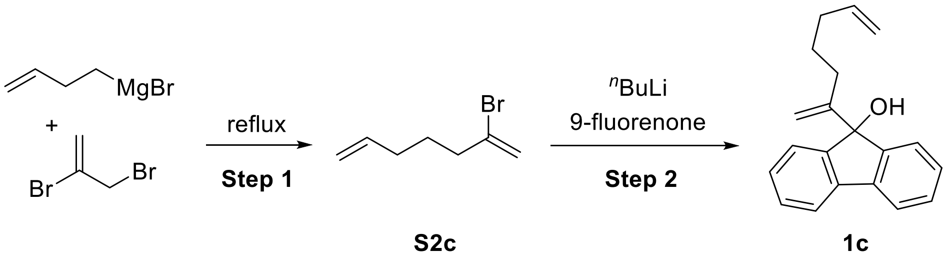

9-(Hepta-1,6-dien-2-yl)-9*H*-fluoren-9-ol (1c) was prepared by the following procedure.

Step 1. Under N_2, a solution of 3-butenylmagnesium bromide (5.0 mL, 5.0 mmol, 1.0 equiv, 1M in THF) was heated to 45 °C and 2,3-dibromopropene (0.5 mL, 5.0 mmol, 1.0 equiv) was added dropwise. The mixture was allowed to stir for 5 h at the same temperature before a saturated aqueous solution of NH_4Cl was added to quench the reaction. The layers were separated and the aqueous layer was extracted with EtOAc (20 mL × 3). The combined organic layers were dried over anhydrous Na_2SO_4, filtered, and concentrated. The crude vinyl bromide was used directly in the next step without further purification.

Step 2. Under N_2, to a solution of **S2c** (500 mg, 2.85 mmol, 1.0 equiv) in anhydrous THF (0.5 M) at -78 °C was added ^{n}BuLi (1.78 mL, 2.85 mmol, 1.0 equiv, 1.6 M in *n*-hexane) dropwise. After stirring at the same temperature for 1 h, a solution of 9-fluorenone (540 mg, 3.0 mmol, 1.05 equiv, 1 M in THF) was slowly added. The mixture was allowed to warm to room temperature and stirred overnight. Brine was added to quench the reaction and the layers were separated. The aqueous layer was extracted with EtOAc (20 mL × 3). The combined organic layers were dried over anhydrous Na_2SO_4, filtered, and concentrated. The residue was purified by flash column chromatography (eluent: *n*-hexane/EtOAc = 10:1) on silica gel to afford the desired product **1c** as a colorless oil (525.1 mg, 38% yield after two steps).

^{1}H NMR (400 MHz, $CDCl_3$) δ 7.65 – 7.63 (m, 2H), 7.39 – 7.35 (m, 4H), 7.30 – 7.26 (m, 2H), 5.86 (s, 1H), 5.60 – 5.50 (m, 1H), 5.18 (d, *J* = 1.4 Hz, 1H), 4.81 – 4.75 (m, 2H), 2.22 (s, 1H), 1.77 – 1.72 (m, 2H), 1.42 (t, *J* = 7.6 Hz, 2H), 1.30 – 1.23 (m, 2H) ppm.

^{13}C NMR (101 MHz, $CDCl_3$) δ 149.1, 148.4, 140.2, 138.5, 129.1, 128.1, 123.9, 120.0, 114.2, 109.3, 84.6, 33.0, 30.1, 27.3 ppm.

HRMS (ES-) Calcd for $C_{20}H_{19}O^-$ [M-H]$^-$: 275.1441, Found: 275.1440.

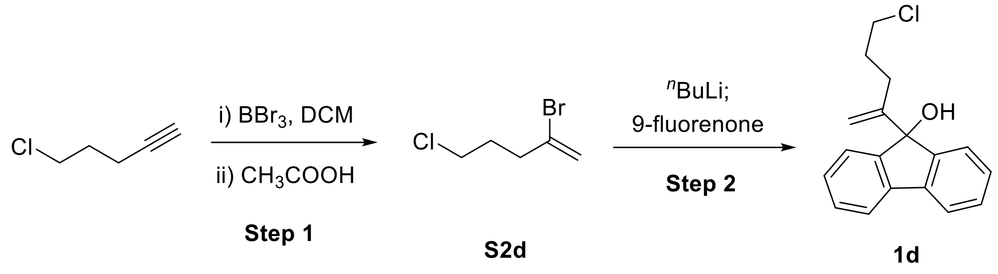

9-(5-Chloropent-1-en-2-yl)-9*H*-fluoren-9-ol (1d) was prepared as the following procedure.

Step 1. Under N_2, to a solution of BBr_3 (30.0 mL, 30.0 mmol, 0.5 equiv) in dry DCM (0.5 M) was added 5-chloro-1-pentyne (6.15 g, 60.0 mmol, 1.0 equiv) dropwise at -78 ^{o}C. The mixture was then allowed to warm to room temperature and stirred for 3 h before acetic acid (21.0 mL, 180.0 mmol 6.0 equiv) was added slowly. After stirring at the same temperature for another 1 h, the solid Na_2CO_3 (8 g) was slowly added to neutralize the reaction mixture. Brine was then added and the organic layer was separated. The aqueous phase was extracted with DCM (20 mL × 3). The combined organic layers were dried over anhydrous Na_2SO_4, filtered, and concentrated. The residue was purified by flash column chromatography on silica gel to afford the corresponding product **S2d**.

Step 2. Under N_2, to a solution of **S2d** (5.5 g, 30.1 mmol, 1.0 equiv) in anhydrous THF (0.5 M) at -78 ^{o}C was added ^{n}BuLi (19.75 mL, 31.6 mmol, 1.05 equiv, 1.6 M in *n*-hexane) dropwise. After stirring at the same temperature for 1 h, a solution of 9-fluorenone (5.4 g, 30.1 mmol, 1.0 equiv, 1 M in THF) was slowly added. The mixture was allowed to warm to room temperature and stirred overnight. Brine was then added and the organic layer was separated. The aqueous phase was extracted with EtOAc (30 mL × 3). The combined organic layers were dried over anhydrous Na_2SO_4, filtered, and concentrated. The residue was purified by flash column chromatography (eluent: *n*-hexane/EtOAc = 10:1) on silica gel to afford the desired product **1d** as a yellow solid (4.54 g, 27% yield after two steps).

^{1}H NMR (400 MHz, $CDCl_3$) δ 7.54 (d, J = 7.5 Hz, 2H), 7.31 – 7.17 (m, 6H), 5.77 (s, 1H),

5.07 (d, *J* = 1.1 Hz, 1H), 3.12 (t, *J* = 6.5 Hz, 2H), 2.44 (s, 1H), 1.54 – 1.44 (m, 4H) ppm.

^{13}C NMR (101 MHz, $CDCl_3$) *δ* 148.1, 147.6, 140.1, 129.2, 128.2, 123.9, 120.1, 110.2, 84.4, 44.2, 31.2, 28.0 ppm.

HRMS (CI+) Calcd for $C_{18}H_{17}ClO^+$ (M^+): 284.0968, Found: 284.0963.

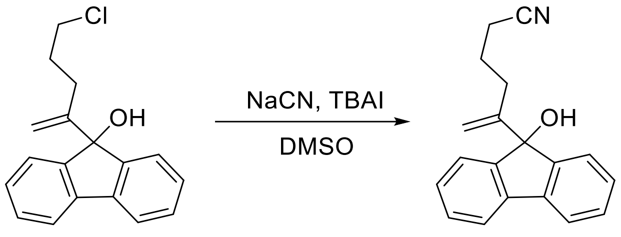

1d **1e**

5-(9-Hydroxy-9*H*-fluoren-9-yl)hex-5-enenitrile (1ae). To a solution of **1d** (570 mg, 2.0 mmol, 1.0 equiv) in DMSO (0.5 M) was added NaCN (196 mg, 4.0 mmol, 2.0 equiv) and TBAI (100 mg). The mixture was allowed to stir at 70 °C for 48 h. An aqueous solution of NaOH (1M) was added and the mixture was extracted with DCM (20 mL × 3). The combined organic layers were dried over anhydrous Na_2SO_4, filtered, and concentrated. The residue was purified by flash column chromatography (eluent: *n*-hexane/EtOAc = 5:1) on silica gel to afford the desired product **1e** as a white solid (458 mg, 76% yield).

^{1}H NMR (400 MHz, $CDCl_3$) δ 7.65 (d, *J* = 7.5 Hz, 2H), 7.41 – 7.36 (m, 4H), 7.32 – 7.28 (m, 2H), 5.94 (s, 1H), 5.18 (s, 1H), 2.24 (s, 1H), 2.02 (t, *J* = 7.2 Hz, 2H), 1.56 (t, *J* = 7.4 Hz, 2H), 1.50 – 1.43 (m, 2H) ppm.

^{13}C NMR (101 MHz, $CDCl_3$) δ 147.9, 147.2, 140.1, 129.4, 128.3, 123.8, 120.2, 119.3, 110.8, 84.3, 29.9, 24.1, 16.3 ppm.

HRMS (ES-) Calcd for $C_{19}H_{16}NO^-$ $[M-H]^-$: 274.1237, Found: 274.1233.

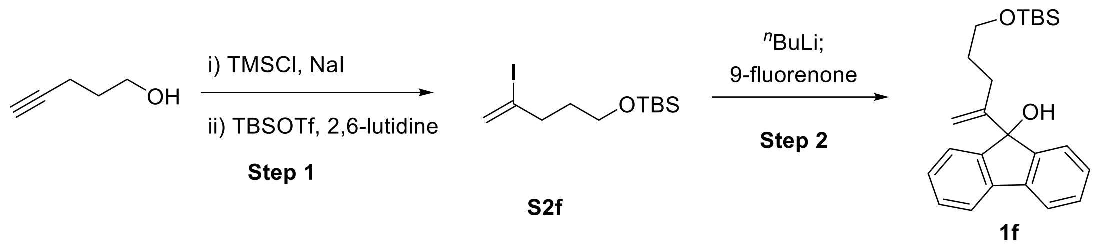

9-(5-((*tert*-Butyldimethylsilyl)oxy)pent-1-en-2-yl)-9*H*-fluoren-9-ol (1f) was prepared as the following procedure.

Step 1. Under N_2, to a mixture of NaI (3.75 g, 25.0 mmol, 2.5 equiv) in MeCN (2.5 M) was added TMSCl (1.09 g, 10.0 mmol, 1.0 equiv) at room temperature. Then H_2O (90.0 mg, 5.0 mmol, 0.5 equiv) was added and the mixture was allowed to stir at room temperature for 10 min before 4-pentyn-1-ol (841.2 mg, 10.0 mmol, 1.0 equiv) was added. The reaction was stirred at room temperature for at least 1 h until the 4-pentyn-1-ol was fully consumed according to TLC analysis. Brine was then added and the organic layer was separated. The aqueous phase was extracted with EtOAc (20 mL × 3). The combined organic layers were dried over anhydrous Na_2SO_4, filtered, and concentrated. The residue was used directly in the next step without further purification.

At 0 °C, to a solution of crude product (837.0 mg, 3.95 mmol, 1.0 equiv) and 2,6-lutidine (1.02 g, 9.48 mmol, 2.4 equiv) in anhydrous DCM (0.25 M) was added TBSOTf (1.25 g, 4.74 mmol, 1.2 equiv) dropwise. The mixture was allowed to warm to room temperature and stirred for 1.5 h. Brine was then added and the organic layer was separated. The aqueous phase was extracted with DCM (20 mL × 3). The combined organic layers were dried over anhydrous Na_2SO_4, filtered, and concentrated. The residue was purified by flash column chromatography (eluent: *n*-hexane/EtOAc = 20:1) on silica gel to afford the corresponding product **S2f**.

Step 2. Under N_2, to a solution of **S2f** (894.0 mg, 2.74 mmol, 1.1 equiv) in anhydrous THF (0.5 M) at -78 °C was added ^{n}BuLi (1.71 mL, 2.74 mmol, 1.1 equiv, 1.6 M in *n*-hexane) dropwise. After stirring at the same temperature for 1 h, a solution of 9-

fluorenone (448.7 mg, 2.94 mmol, 1.0 equiv, 1 M in THF) was slowly added. The mixture was allowed to warm to room temperature and stirred overnight. A saturated NH_4Cl solution was added to quench the reaction, and the organic layer was separated. The aqueous phase was extracted with EtOAc (20 mL × 3). The combined organic layers were dried over anhydrous Na_2SO_4, filtered, and concentrated. The residue was purified by flash column chromatography (eluent: *n*-hexane/EtOAc = 10:1) on silica gel to afford the desired product **1f** as a white solid (278 mg, 7% yield after two steps).

^{1}H NMR (400 MHz, $CDCl_3$) δ 7.63 (d, *J* = 7.5 Hz, 2H), 7.39 – 7.34 (m, 4H), 7.39 – 7.26 (m, 2H), 5.85 (s, 1H), 5.18 (d, *J* = 1.1 Hz, 1H), 3.32 – 3.29 (m, 2H), 2.23 (s, 1H), 1.46 – 1.36 (m, 4H), 0.75 (s, 9H), -0.14 (s, 6H) ppm.

^{13}C NMR (101 MHz, $CDCl_3$) δ 149.2, 148.4, 140.3, 129.1, 128.2, 123.9, 120.0, 109.0, 84.7, 62.6, 31.3, 27.0, 25.8, 18.2, -5.4 ppm.

HRMS (ES-) Calcd for $C_{24}H_{31}O_2Si^-$ [M-H]$^-$: 379.2099, Found: 379.2091.

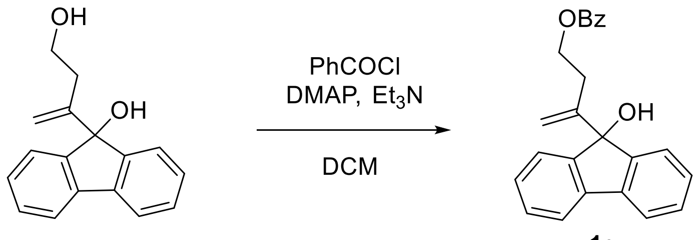

1g

3-(9-Hydroxy-9*H*-fluoren-9-yl)but-3-en-1-yl benzoate (1g). At 0 °C, To a solution of 9-(4-hydroxybut-1-en-2-yl)-9*H*-fluoren-9-ol (153.2 mg, 0.6 mmol, 1.0 equiv), DMAP (36.0 mg, 0.3 mmol, 0.5 equiv) and triethylamine (303.6 mg, 3.0 mmol, 5.0 equiv) in anhydrous DCM (0.25 M) was added benzoyl chloride (126.5 mg, 0.9 mmol, 1.1 equiv) dropwise. The mixture was allowed to warm to room temperature and stirred for 10 min. Brine was then added and the organic layer was separated. The aqueous phase was extracted with DCM (20 mL × 3). The combined organic layers were dried over anhydrous Na_2SO_4, filtered, and concentrated. The residue was purified by flash column chromatography (eluent: *n*-hexane/EtOAc = 2:1) on silica gel to afford the desired product **1g** as a white solid (226.9 mg, 99% yield).

^{1}H NMR (400 MHz, $CDCl_3$) δ 7.92 – 7.90 (m, 2H), 7.65 (d, *J* = 7.5 Hz, 2H), 7.52 (tt, J_1 = 7.4 Hz, J_2 = 1.8 Hz, 1H), 7.42 – 7.36 (m, 6H), 7.28 (td, J_1 = 7.4 Hz, J_2 = 1.1 Hz, 2H), 5.96 (s, 1H), 5.32 (d, *J* = 1.1 Hz, 1H), 4.08 (t, *J* = 7.0 Hz, 2H), 2.36 (s, 1H), 1.92 (dd, J_1 = 7.0 Hz, J_2 = 6.6 Hz, 2H) ppm.

^{13}C NMR (101 MHz, $CDCl_3$) δ 166.3, 147.8, 145.0, 140.2, 132.8, 130.2, 129.5, 129.3, 128.3, 128.2, 124.0, 120.2, 111.6, 84.3, 63.4, 30.1 ppm.

HRMS (ES+) Calcd for $C_{24}H_{20}NaO_3^+$ [M+Na]$^+$: 379.1305, Found: 379.1313.

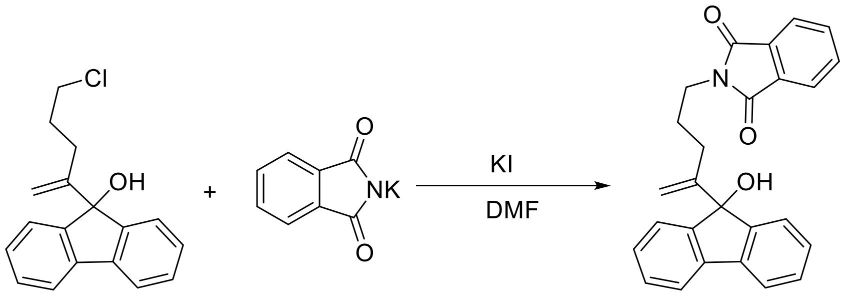

1d 1h

2-(4-(9-Hydroxy-9*H*-fluoren-9-yl)pent-4-en-1-yl)isoindoline-1,3-dione (1h). The mixture of **1d** (569.6 mg, 1.0 equiv), potassium phthalimide (444.5 mg, 1.2 equiv) and KI (99.6 mg, 0.6 mmol, 0.3 equiv) in DMF (0.33 M) was allowed to stir at 70 °C overnight. Brine was then added and the mixture was extracted with EtOAc (20 mL × 3). The combined organic layers were dried over anhydrous Na_2SO_4, filtered, and concentrated. The residue was purified by flash column chromatography (eluent: *n*-hexane/EtOAc = 5:1) on silica gel to afford the desired product **1h** as a white solid (442.9 mg, 56% yield).

^{1}H NMR (400 MHz, $CDCl_3$) δ 7.78 – 7.75 (m, 2H), 7.71 – 7.67 (m, 2H), 7.54 (d, *J* = 7.3 Hz, 2H), 7.36 (d, *J* = 7.4 Hz, 2H), 7.31 (t, *J* = 6.9 Hz, 2H), 7.25 (t, *J* = 7.3 Hz, 2H), 5.88 (s, 1H), 5.22 (s, 1H), 3.35 (t, *J* = 7.3 Hz, 2H), 2.27 (s, 1H), 1.59 – 1.44 (m, 4H) ppm.

^{13}C NMR (101 MHz, $CDCl_3$) δ 168.1, 148.1, 148.0, 140.1, 133.7, 132.1, 129.1, 128.2, 123.9, 123.1, 120.0, 109.8, 84.5, 37.4, 27.8, 27.0 ppm.

HRMS (ES+) Calcd for $C_{26}H_{21}NNaO_3^+$ [M+Na]$^+$: 418.1414, Found: 418.1423.

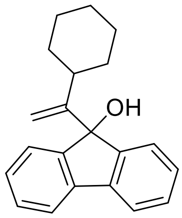

1i

9-(1-Cyclohexylvinyl)-9*H*-fluoren-9-ol (1i) was prepared as a white solid according to the General Procedure B (eluent: *n*-hexane/EtOAc = 10:1, 66% yield).

^{1}H NMR (400 MHz, $CDCl_3$) δ 7.64 (dd, J_1 = 6.6 Hz, J_2 = 1.8 Hz, 2H), 7.39 – 7.35 (m, 4H), 7.29 – 7.25 (m, 2H), 5.86 (s, 1H), 5.21 (d, J = 0.8 Hz, 1H), 2.16 (s, 1H), 1.48 – 1.41 (m, 3H), 1.27 (dd, J_1 = 13.0 Hz, J_2 = 1.5 Hz, 2H), 1.13 – 0.88 (m, 4H), 0.72 (qt, J_1 = 13.6 Hz, J_2 = 3.6 Hz, 2H) ppm.

^{13}C NMR (101 MHz, $CDCl_3$) δ 155.6, 148.1, 140.5, 129.0, 127.9, 124.2, 120.0, 108.5, 84.7, 39.4, 34.9, 26.8, 25.9 ppm.

HRMS (CI+) Calcd for $C_{21}H_{22}O^+$ (M^+): 290.1671, Found: 290.1671.

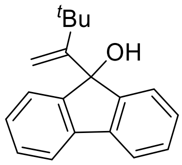

1j

9-(3,3-Dimethylbut-1-en-2-yl)-9*H*-fluoren-9-ol (1j) was prepared as a colorless oil according to the General Procedure A (eluent: hexanes/Et_2O = 20:1,17% yield).

^{1}H NMR (400 MHz, $CDCl_3$) δ 7.65 – 7.63 (m, 2H), 7.39 – 7.35 (m, 4H), 7.30 – 7.26 (m, 2H), 5.96 (s, 1H), 5.50 (d, J = 0.8 Hz, 1H), 2.08 (s, 1H), 0.81 (s, 9H) ppm.

^{13}C NMR (101 MHz, $CDCl_3$) δ 156.5, 150.1, 140.1, 128.9, 128.0, 124.3, 120.4, 112.9, 84.1, 35.5, 31.7 ppm.

HRMS (CI+) Calcd for $C_{19}H_{20}O^+$ (M^+): 264.1514, Found: 264.1512.

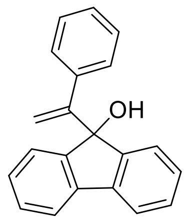

1k

9-(1-Phenylvinyl)-9*H*-fluoren-9-ol (1k) was prepared as a white solid according to the General Procedure A (eluent: *n*-hexane/EtOAc = 10:1, 61% yield after two steps).

^{1}H NMR (400 MHz, $CDCl_3$) δ 7.54 (d, *J* = 7.5 Hz, 2H), 7.46 – 7.44 (m, 2H), 7.32 (td, J_1 = 7.4 Hz, J_2 = 1.3 Hz, 2H), 7.26 (td, J_1 = 7.4 Hz, J_2 = 1.2 Hz, 2H), 7.03 – 6.99 (m, 1H), 6.95 – 6.91 (m, 2H), 6.71 – 6.69 (m, 2H), 6.08 (d, *J* = 1.7 Hz, 1H), 5.40 (d, *J* = 1.7 Hz, 1H), 2.34 (s, 1H).

^{13}C NMR (101 MHz, $CDCl_3$) δ 150.4, 148.0, 140.1, 139.9, 129.1, 128.1, 128.0, 127.2, 126.8, 124.4, 120.1, 115.3, 84.0 ppm.

HRMS (ES-) Calcd for $C_{21}H_{15}O^-$ $[M-H]^-$: 283.1128, Found: 283.1128.

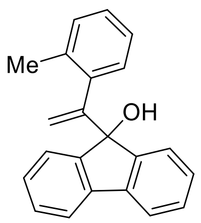

1l

9-(1-(*o*-Tolyl)vinyl)-9*H*-fluoren-9-ol (1l) was prepared as a white solid according to the General Procedure C (eluent: *n*-hexane/EtOAc = 10:1, 65% yield after two steps).

^{1}H NMR (400 MHz, $CDCl_3$) δ 7.54 (d, *J* = 7.3 Hz, 2H), 7.47 – 7.45 (m, 2H), 7.33 (td, J_1 = 7.4 Hz, J_2 = 1.2 Hz, 2H), 7.26 (td, J_1 = 7.4 Hz, J_2 = 1.2 Hz, 2H), 7.03 (dd, J_1 = 5.0 Hz, J_2 = 1.0 Hz, 2H), 6.87 – 6.81 (m, 1H), 6.63 (d, *J* = 7.6 Hz, 1H), 5.84 (d, *J* = 1.6 Hz, 1H), 5.09 (d, *J* = 1.6 Hz, 1H), 2.34 (s, 1H), 2.15 (s, 3H) ppm.

^{13}C NMR (101 MHz, $CDCl_3$) δ 148.8, 148.3, 139.9, 138.9, 136.3, 129.9, 129.0, 128.9, 127.7, 126.9, 124.6, 124.2, 120.0, 116.4, 84.8, 20.3 ppm.

HRMS (CI+) Calcd for $C_{22}H_{18}O^+$ (M^+): 298.1358, Found: 298.1356.

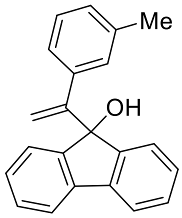

1m

9-(1-(*m*-Tolyl)vinyl)-9*H*-fluoren-9-ol (1m) was prepared as a white solid according to the General Procedure C (eluent: *n*-hexane/EtOAc = 10:1, 54% yield after two steps).

^{1}H NMR (400 MHz, $CDCl_3$) δ 7.54 (d, *J* = 7.5 Hz, 2H), 7.47 – 7.45 (m, 2H), 7.33 (td, J_1 = 7.4 Hz, J_2 = 1.3 Hz, 2H), 7.27 (td, J_1 = 7.4 Hz, J_2 = 1.3 Hz, 2H), 6.84 – 6.81 (m, 2H), 6.59 (s, 1H), 6.51 – 6.47 (m, 1H), 6.04 (d, *J* = 1.7 Hz, 1H), 5.39 (d, *J* = 1.7 Hz, 1H), 2.39 (s, 1H), 2.09 (s, 3H) ppm.

^{13}C NMR (101 MHz, $CDCl_3$) δ 150.4, 148.1, 140.1, 139.8, 136.6, 129.0, 128.9, 128.0, 127.5, 127.0, 125.0, 124., 120.0, 115.1, 84.0, 21.2 ppm.

HRMS (CI+) Calcd for $C_{22}H_{18}O^+$ (M^+): 298.1358, Found: 298.1351.

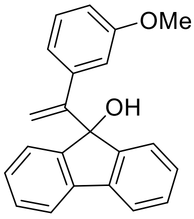

1n

9-(1-(3-Methoxyphenyl)vinyl)-9*H*-fluoren-9-ol (1n) was prepared as a white solid according to the General Procedure C (eluent: *n*-hexane/EtOAc = 10:1, 82% yield).

^{1}H NMR (400 MHz, $CDCl_3$) δ 7.55 (dd, J_1 = 6.9 Hz, J_2 = 0.8 Hz, 2H), 7.47 – 7.45 (m, 2H), 7.29 (dtd, J_1 = 22.1 Hz, J_2 = 7.4 Hz, J_3 = 1.2 Hz, 4H), 6.86 (t, *J* = 7.9 Hz, 1H), 6.55 (ddd, J_1 = 8.3 Hz, J_2 = 2.6 Hz, J_3 = 0.9 Hz, 1H), 6.38 – 6.36 (m, 1H), 6.17 (dd, J_1 = 2.4 Hz, J_2 = 1.7 Hz, 1H), 6.11 (d, *J* = 1.7 Hz, 1H), 5.44 (d, *J* = 1.7 Hz, 1H), 3.44 (s, 3H), 2.36 (s, 1H) ppm.

^{13}C NMR (101 MHz, $CDCl_3$) δ 158.3, 150.1, 148.1, 141.2, 140.2, 129.2, 128.3, 128.1, 124.4, 120.7, 120.1, 115.3, 113.2, 112.7, 83.9, 54.7 ppm.

HRMS (CI+) Calcd for $C_{22}H_{18}O_2^+$ (M^+): 314.1307, Found: 314.1306.

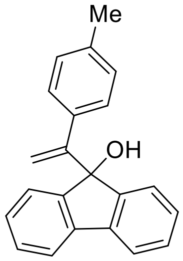

1o

9-(1-(*p*-Tolyl)vinyl)-9*H*-fluoren-9-ol (1o) was prepared as a white solid according to the General Procedure A (eluent: *n*-hexane/EtOAc = 10:1, 47% yield).

^{1}H NMR (400 MHz, $CDCl_3$) δ 7.55 (d, *J* = 7.4 Hz, 2H), 7.45 (d, *J* = 7.4 Hz, 2H), 7.32 (td, J_1 = 7.4 Hz, J_2 = 1.1 Hz, 2H), 7.26 (td, *J* = 7.3, 1.0 Hz, 2H), 6.75 (d, *J* = 8.0 Hz, 2H), 6.61 (d, *J* = 8.1 Hz, 2H), 6.04 (d, *J* = 1.7 Hz, 1H), 5.38 (d, *J* = 1.7 Hz, 1H), 2.34 (s, 1H), 2.14 (s, 3H) ppm.

^{13}C NMR (101 MHz, $CDCl_3$) δ 150.2, 148.2, 140.1, 137.0, 136.4, 129.1, 128.1, 128.0, 127.8, 124.4, 120.1, 115.0, 84.0, 20.9 ppm.

HRMS (CI+) Calcd for $C_{22}H_{18}O^+$ (M^+): 298.1358, Found: 298.1354.

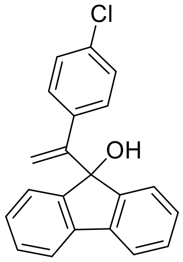

1p

9-(1-(4-Chlorophenyl)vinyl)-9*H*-fluoren-9-ol (1p) was prepared as a white solid according to the General Procedure C (eluent: *n*-hexane/EtOAc = 10:1, 64% yield).

^{1}H NMR (400 MHz, $CDCl_3$) δ 7.54 (d, *J* = 7.5 Hz, 2H), 7.45 – 7.43 (m, 2H), 7.33 (td, J_1 = 7.4 Hz, J_2 = 1.2 Hz, 2H), 7.27 (td, J_1 = 7.4 Hz, J_2 = 1.2 Hz, 2H), 6.91 – 6.88 (m, 2H), 6.62 – 6.59 (m, 2H), 6.10 (d, *J* = 1.6 Hz, 1H), 5.38 (d, *J* = 1.6 Hz, 1H), 2.35 (s, 1H) ppm.

^{13}C NMR (101 MHz, $CDCl_3$) δ 149.3, 147.7, 140.1, 138.4, 132.7, 129.3 (2C), 128.2, 127.4, 124.3, 120.2, 115.7, 83.8 ppm.

HRMS (CI+) Calcd for $C_{21}H_{15}ClO^+$ (M^+): 318.0811, Found: 318.0812.

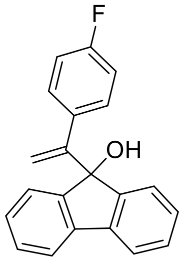

1q

9-(1-(4-Fluorophenyl)vinyl)-9*H*-fluoren-9-ol (1q) was prepared as a white solid according to the General Procedure C (eluent: *n*-hexane/EtOAc = 10:1, 62% yield).

^{1}H NMR (400 MHz, $CDCl_3$) δ 7.54 – 7.52 (m, 2H), 7.44 (dd, J_1 = 6.8 Hz, J_2 = 0.7 Hz, 2H), 7.33 (td, J_1 = 7.4 Hz, J_2 = 1.3 Hz, 2H), 7.27 (td, J_1 = 7.4 Hz, J_2 = 1.3 Hz, 2H), 6.66 – 6.59 (m, 4H), 6.08 (d, *J* = 1.7 Hz, 1H), 5.36 (d, *J* = 1.7 Hz, 1H), 2.35 (s, 1H) ppm.

^{13}C NMR (101 MHz, $CDCl_3$) δ 160.7 (d, *J* = 246.6 Hz), 149.4, 147.8, 140.1, 135.9 (d, *J* = 3.5 Hz), 129.6 (d, *J* = 8.0 Hz), 129.2, 128.1, 124.3, 120.1, 115.5, 114.1 (d, *J* = 21.2 Hz), 84.1 ppm.

^{19}F NMR (376 MHz, $CDCl_3$) 115.7 ppm.

HRMS (CI+) Calcd for $C_{21}H_{15}FO^+$ (M^+): 302.1107, Found: 302.1102.

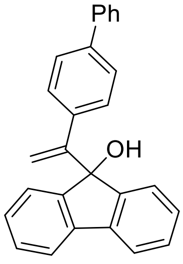

1r

9-(1-([1,1'-Biphenyl]-4-yl)vinyl)-9*H*-fluoren-9-ol (1r) was prepared as a white solid according to the General Procedure C (eluent: *n*-hexane/EtOAc = 10:1, 83% yield).

^{1}H NMR (400 MHz, $CDCl_3$) δ 7.57 (d, *J* = 7.3 Hz, 2H), 7.49 – 7.47 (m, 2H), 7.45 – 7.42 (m, 2H), 7.36 – 7.32 (m, 4H), 7.30 – 7.25 (m, 3H), 7.21 – 7.18 (m, 2H), 6.82 – 6.78 (m, 2H), 6.11 (d, *J* = 1.6 Hz, 1H), 5.47 (d, *J* = 1.7 Hz, 1H), 2.37 (s, 1H) ppm.

^{13}C NMR (101 MHz, $CDCl_3$) δ 150.0, 148.1, 140.6, 140.2, 139.4, 139.0, 129.2, 128.6, 128.4, 128.2, 127.1, 126.8, 126.0, 124.4, 120.2, 115.5, 84.0 ppm.

HRMS (CI+) Calcd for $C_{27}H_{20}O^+$ (M^+): 360.1514, Found: 360.1514.

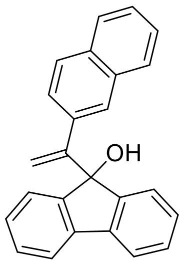

1s

9-(1-(Naphthalen-2-yl)vinyl)-9*H*-fluoren-9-ol (1s) was prepared as a white solid according to the General Procedure A (eluent: *n*-hexane/EtOAc = 10:1, 76% yield).

^{1}H NMR (400 MHz, $CDCl_3$) δ 7.61 – 7.60 (m, 1H), 7.51 – 7.46 (m, 5H), 7.40 (dd, J_1 = 8.5 Hz, J_2 = 1.8 Hz, 1H), 7.31 – 7.22 (m, 6H), 7.16 (s, 1H), 6.87 – 6.84 (m, 1H), 6.14 (t, J = 1.9 Hz, 1H), 5.48 (t, J = 2.0 Hz, 1H), 2.40 (d, J = 7.2 Hz, 1H) ppm.

^{13}C NMR (101 MHz, $CDCl_3$) δ 150.3, 148.0, 140.1, 137.5, 132.6, 132.2, 129.2, 128.1, 128.0, 127.2, 126.9, 126.6 (2C), 125.6, 125.5, 124.4, 120.1, 115.9, 84.1 ppm.

HRMS (CI+) Calcd for $C_{25}H_{18}O^+$ (M^+): 334.1358, Found: 334.1351.

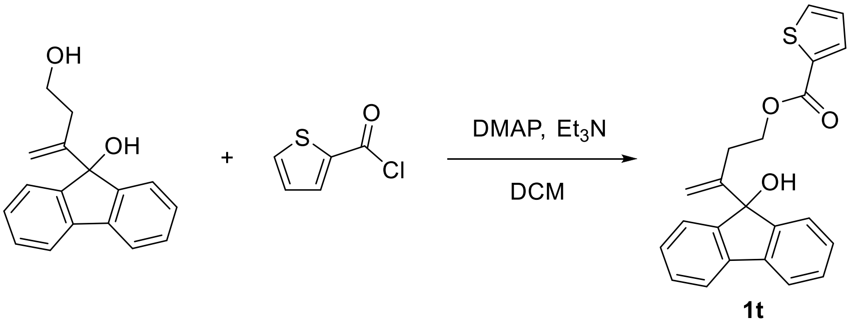

1t

3-(9-Hydroxy-9H-fluoren-9-yl)but-3-en-1-yl thiophene-2-carboxylate (1t). At 0 °C, to a solution of 9-(4-hydroxybut-1-en-2-yl)-9*H*-fluoren-9-ol (151.4 mg, 0.6 mmol, 1.0 equiv), DMAP (36.6 mg, 0.3 mmol, 0.5 equiv) and triethylamine (303.6 mg, 3.0 mmol, 5.0 equiv) in anhydrous DCM (0.25 M) was added thiophene-2-carbonyl chloride (96.4 mg, 0.66 mmol, 1.1 equiv) dropwise. The mixture was allowed to warm to room temperature and stirred for 10 min. Brine was then added and the organic layer was separated. The aqueous phase was extracted with DCM (20 mL × 3). The combined organic layers were dried over anhydrous Na_2SO_4, filtered, and concentrated. The

residue was purified by flash column chromatography (eluent: *n*-hexane/EtOAc = 2:1) on silica gel to afford the desired product **1t** as a white solid (232.0 mg, 99% yield).

^{1}H NMR (400 MHz, $CDCl_3$) δ 7.68 (dd, J_1 = 3.7 Hz, J_2 = 1.2 Hz, 1H), 7.65 (d, J = 7.5 Hz, 2H), 7.51 (dd, J_1 = 5.0 Hz, J_2 = 1.2 Hz, 1H), 7.39 (dt, J_1 = 7.5 Hz, J_2 = 4.2 Hz, 4H), 7.30 – 7.26 (m, 2H), 7.05 (dd, J_1 = 4.9 Hz, J_2 = 3.8 Hz, 1H), 5.95 (s, 1H), 5.31 (d, J = 0.9 Hz, 1H), 4.04 (t, J = 7.0 Hz, 2H), 2.33 (s, 1H), 1.90 (t, J = 6.9 Hz, 2H) ppm.

^{13}C NMR (101 MHz, $CDCl_3$) δ 161.9, 147.8, 144.9, 140.2, 133.8, 133.3, 132.2, 129.4, 128.3, 127.6, 124.0, 120.2, 111.8, 84.3, 63.6, 30.1 ppm.

HRMS (ES+) Calcd for $C_{22}H_{18}NaO_3S^+$ $[M+Na]^+$: 385.0869, Found: 385.0874.

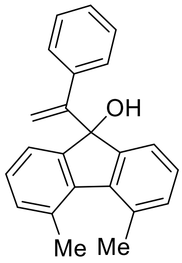

1u

4,5-Dimethyl-9-(1-phenylvinyl)-9*H*-fluoren-9-ol (1u) was prepared as a white solid according to the General Procedure A, except that 9-fluorenone was replaced by 4,5-dimethyl-9*H*-fluoren-9-one (eluent: *n*-hexane/EtOAc = 10:1, 67% yield).

^{1}H NMR (400 MHz, $CDCl_3$) δ 7.32 (d, J = 7.2 Hz, 2H), 7.18 (t, J = 7.4 Hz, 2H), 7.12 (d, J = 7.2 Hz, 2H), 7.00 (tt, J_1 = 7.3 Hz, J_2 = 2.4 Hz, 1H), 6.94 – 6.90 (m, 2H), 6.62 – 6.59 (m, 2H), 6.09 (d, J = 1.8 Hz, 1H), 5.39 (d, J = 1.8 Hz, 1H), 2.61 (s, 6H) 2.26 (s, 1H) ppm.

^{13}C NMR (101 MHz, $CDCl_3$) δ 151.1, 149.1, 140.2, 140.1, 133.0, 132.2, 127.9, 127.7, 127.1, 126.7, 121.9, 115.0, 82.9, 24.8 ppm.

HRMS (ES-) Calcd for $C_{23}H_{19}O^-$ $[M-H]^-$: 311.1441, Found: 311.1451.

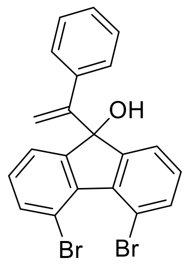

1v

4,5-Dibromo-9-(1-phenylvinyl)-9*H*-fluoren-9-ol (1v) was prepared as a white solid according to the General Procedure A, except that 9-fluorenone was replaced by 4,5-dibromo-*9H*-fluoren-9-one (eluent: *n*-hexane/EtOAc = 10:1, 63% yield).

^{1}H NMR (400 MHz, $CDCl_3$) δ 7.61 (dd, J_1 = 7.9 Hz, J_2 = 1.0 Hz, 2H), 7.45 (dd, J_1 = 7.4 Hz, J_2 = 1.0 Hz, 2H), 7.19 (t, *J* = 7.5 Hz, 2H), 7.08 – 7.04 (m, 1H), 7.00 – 6.96 (m, 2H), 6.58 – 6.55 (m, 2H), 6.10 (d, *J* = 1.6 Hz, 1H), 5.43 (d, *J* = 1.5 Hz, 1H), 2.48 (s, 1H) ppm.

^{13}C NMR (101 MHz, $CDCl_3$) δ 151.4, 149.8, 139.2, 139.1, 136.1, 129.7, 127.7, 127.4, 127.2, 123.0, 116.1, 115.4, 83.1 ppm.

HRMS (ES-) Calcd for $C_{21}H_{13}Br_2O^-$ [M-H]$^-$: 438.9339, Found: 438.9335.

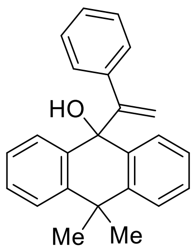

1w

10,10-Dimethyl-9-(1-phenylvinyl)-9,10-dihydroanthracen-9-ol (1w) was prepared as a white solid according to the General Procedure A, except that 9-fluorenone was replaced by 10,10-dimethylanthracen-9(10*H*)-one (eluent: *n*-hexane/EtOAc = 10:1, 84% yield).

^{1}H NMR (400 MHz, $CDCl_3$) δ 7.80 – 7.75 (m, 2H), 7.40 – 7.28 (m, 6H), 6.97 – 6.92 (m, 1H), 6.87 – 6.83 (m, 2H), 6.29 – 6.27 (m, 2H), 6.12 (d, *J* = 2.0 Hz, 1H), 5.19 (d, *J* = 2.0 Hz, 1H), 2.53 (s, 1H), 1.62 (s, 3H), 0.95 (s, 3H) ppm.

^{13}C NMR (101 MHz, $CDCl_3$) δ 155.3, 143.0, 140.6, 136.1, 129.0, 128.1, 128.0, 126.9, 126.5, 126.4, 126.3, 111.7, 74.4, 36.9, 34.2, 33.0 ppm.

HRMS (ES-) Calcd for $C_{24}H_{21}O^-$ [M-H]$^-$: 325.1598, Found: 325.1601.

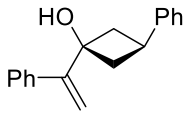

2a

***cis*-3-Phenyl-1-(1-phenylvinyl)cyclobutan-1-ol (2a)** was prepared as a white solid according to the General Procedure D (eluent: *n*-hexane/EtOAc = 10:1, 54% yield, *cis*/*trans* > 20:1). This is a known compound, and the spectra data are consistent with the literature.[3]

^{1}H NMR (400 MHz, $CDCl_3$) δ 7.60 – 7.58 (m, 2H), 7.42 – 7.29 (m, 7H), 7.26 – 7.22 (m, 1H), 5.61 (s, 1H), 5.54 (s, 1H), 3.10 – 2.97 (m, 3H), 2.51 – 2.45 (m, 2H), 2.24 (brs, 1H) ppm.

^{13}C NMR (101 MHz, $CDCl_3$) δ 151.3, 144.6, 138.9, 128.3, 128.2, 127.7, 127.6, 126.6, 126.0, 113.6, 73.2, 43.6, 30.4 ppm.

HRMS (CI+) Calcd for $C_{18}H_{18}O^+$ (M^+): 250.1352, Found: 250.1351.

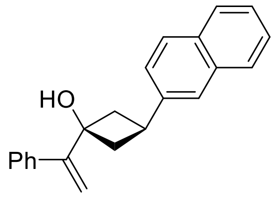

2b

***cis*-3-(Naphthalen-2-yl)-1-(1-phenylvinyl)cyclobutan-1-ol (2b)** was prepared as a white solid according to the General Procedure D (eluent: *n*-hexane/EtOAc = 10:1, 30% yield, *cis*/*trans* > 20:1).

^{1}H NMR (400 MHz, $CDCl_3$) δ 7.83 – 7.80 (m, 3H), 7.69 (s, 1H), 7.61 – 7.58 (m, 2H), 7.50 – 7.33 (m, 6H), 5.63 (s, 1H), 5.55 (s, 1H), 3.27 – 3.18 (m, 1H), 3.09 – 3.03 (m, 2H), 2.60 – 2.53 (m, 2H), 2.21 (s, 1H) ppm.

^{13}C NMR (101 MHz, $CDCl_3$) δ 151.4, 142.1, 139.0, 133.4, 132.1, 128.3, 128.0, 127.7 (2C), 127.6 127.5, 126.0, 125.4, 125.3, 124.6, 113.7, 73.3, 43.5, 30.6 ppm.

HRMS (ES+) Calcd for $C_{22}H_{20}NaO^+$ [M+Na]$^+$: 323.1406, Found: 323.1411.

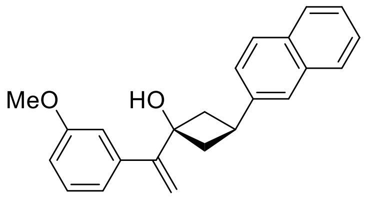

2c

***cis*-1-(1-(3-Methoxyphenyl)vinyl)-3-(naphthalen-2-yl)cyclobutan-1-ol (2c)** was prepared as a white solid according to the General Procedure D (eluent: *n*-hexane/EtOAc = 10:1, 29% yield, *cis*/*trans* = 11.5:1).

^{1}H NMR (400 MHz, $CDCl_3$) δ 7.80 (m, 3H), 7.67 (s, 1H), 7.48 – 7.40 (m, 3H), 7.30 (t, J = 7.9 Hz, 1H), 7.16 – 7.12 (m, 2H), 6.89 (dd, J_1 = 8.2 Hz, J_2 = 2.6 Hz, 1H), 5.62 (s, 1H), 5.53 (s, 1H), 3.84 (s, 3H), 3.22 (p, J = 9.1 Hz, 1H), 3.05 (ddt, J_1 = 11.9 Hz, J_2 = 8.0 Hz, J_3 = 2.6 H, 2H), 2.55 (ddt, J_1 = 12.0 Hz, J_2 = 9.1 Hz, J_3 = 2.6 H, 2H).

^{13}C NMR (101 MHz, $CDCl_3$) δ 159.4, 151.4, 142.1, 140.5, 133.4, 132.1, 129.3, 128.0, 127.6, 127.5, 126.0, 125.4, 125.3, 124.7, 120.1, 113.9, 113.7, 113.0, 73.3, 55.2, 43.5, 30.6 ppm.

HRMS (ES+) Calcd for $C_{23}H_{22}NaO_2^+$ [M+Na]$^+$: 353.1512, Found: 353.1519.

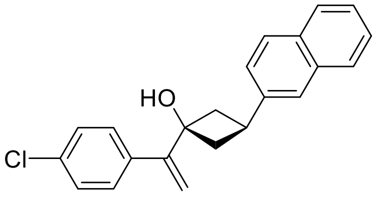

2d

***cis*-1-(1-(4-Chlorophenyl)vinyl)-3-(naphthalen-2-yl)cyclobutan-1-ol (2d)** was prepared as a white solid according to the General Procedure D (eluent: *n*-hexane/EtOAc = 10:1, 31% yield, *cis*/*trans* > 20:1).

^{1}H NMR (400 MHz, $CDCl_3$) δ 7.80 (m, 3H), 7.66 (s, 1H), 7.55 – 7.52 (m, 2H), 7.49 – 7.40 (m, 3H), 7.36 – 7.32 (m, 2H), 5.63 (s, 1H), 5.54 (s, 1H), 3.19 (p, J = 9.1 Hz, 1H), 3.01 (ddt, J_1 = 10.8 Hz, J_2 = 8.1 Hz, J_3 = 2.7 H, 2H), 2.53 (ddt, J_1 = 12.1 Hz, J_2 = 9.2 Hz, J_3 = 2.6 H, 2H), 2.00 (brs, 1H) ppm.

^{13}C NMR (101 MHz, $CDCl_3$) δ 150.3, 141.9, 137.4, 133.6, 133.4, 132.1, 129.1, 128.4, 128.0,

127.6, 127.5, 126.0, 125.3 (2C), 124.6, 114.3, 73.3, 43.4, 30.6 ppm.

HRMS (ES+) Calcd for $C_{22}H_{19}ClNaO^+$ [M+Na]$^+$: 357.1017, Found: 357.1024.

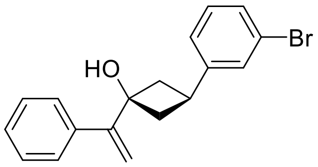

2e

***cis*-3-(3-Bromophenyl)-1-(1-phenylvinyl)cyclobutan-1-ol (2e)** was prepared as a white solid according to the General Procedure D (eluent: *n*-hexane/EtOAc = 10:1, 13% yield, *cis/trans* > 20:1).

^{1}H NMR (400 MHz, $CDCl_3$) δ 7.55 – 7.52 (m, 2H), 7.41 – 7.30 (m, 5H), 7.19 – 7.15 (m, 2H), 5.56 (s, 1H), 5.50 (s, 1H), 3.06 – 2.93 (m, 3H), 2.46 – 2.39 (m, 2H), 2.07 (brs, 1H) ppm.

^{13}C NMR (101 MHz, $CDCl_3$) δ151.2, 147.1, 138.8, 129.9 (2C), 129.1, 128.3, 127.8, 127.7, 125.3, 122.5, 113.8, 73.2, 43.4, 30.2 ppm.

HRMS (ES+) Calcd for $C_{18}H_{17}BrNaO^+$ [M+Na]$^+$: 351.0355, Found: 351.0358.

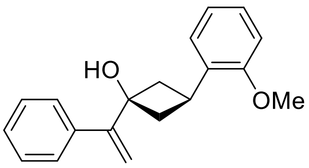

2f

***cis*-3-(2-Methoxyphenyl)-1-(1-phenylvinyl)cyclobutan-1-ol (2f)** was prepared as a white solid according to the General Procedure D (eluent: *n*-hexane/EtOAc = 10:1, 29% yield, *cis/trans* > 20:1).

^{1}H NMR (400 MHz, $CDCl_3$) δ 7.58 – 7.55 (m, 2H), 7.39 – 7.29 (m, 3H), 7.28 – 7.25 (m, 1H), 7.19 (td, J_1 = 7.7 Hz, J_2 = 1.5 Hz, 1H), 6.95 (td, J_1 = 7.5 Hz, J_2 = 0.9 Hz, 1H), 6.83 (dd, J_1 = 8.1 Hz, J_2 = 0.8 Hz, 1H), 5.60 (s, 1H), 5.51 (d, J = 0.5 Hz, 1H), 3.80 (s, 2H), 3.30 – 3.21 (m, 1H), 3.01 – 2.95 (m, 2H), 2.45 – 2.38 (m, 1H), 2.00 (brs, 1H) ppm.

^{13}C NMR (101 MHz, $CDCl_3$) δ 157.4, 151.3, 139.1, 132.7, 128.2, 127.7, 127.5, 127.0, 126.8,

120.3, 113.6, 110.0, 73.7, 55.2, 42.5, 25.5 ppm.

HRMS (ES+) Calcd for $C_{19}H_{20}NaO_2^+$ $[M+Na]^+$: 303.1356, Found: 303.1358.

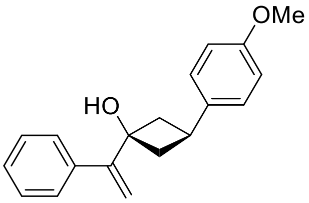

2g

***cis*-3-(4-Methoxyphenyl)-1-(1-phenylvinyl)cyclobutan-1-ol (2g)** was prepared as a white solid according to the General Procedure D (eluent: *n*-hexane/EtOAc = 10:1, 16% yield, *cis*/*trans* > 20:1).

^{1}H NMR (400 MHz, $CDCl_3$) δ 7.56 – 7.53 (m, 2H), 7.39 – 7.30 (m, 3H), 7.21 – 7.18 (m, 2H), 6.88 – 6.84 (m, 2H), 5.57 (s, 1H), 5.50 (s, 1H), 3.80 (s, 3H), 3.02 – 2.92 (m, 3H), 2.44 – 2.37 (m, 2H), 2.01 (brs, 1H) ppm.

^{13}C NMR (101 MHz, $CDCl_3$) δ 157.9, 151.4, 139.0, 136.9, 128.3, 127.7 (2C), 127.6, 113.8, 113.7, 73.2, 55.3, 43.9, 29.9 ppm.

HRMS (ES+) Calcd for $C_{19}H_{20}NaO_2^+$ $[M+Na]^+$: 303.1356, Found: 303.1365.

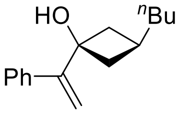

2h

***cis*-3-Butyl-1-(1-phenylvinyl)cyclobutan-1-ol (2h)** was prepared as a colorless oil according to the General Procedure D (eluent: *n*-hexane/EtOAc = 10:1, 5% yield, *cis*/*trans* = 19:1).

^{1}H NMR (400 MHz, $CDCl_3$) δ 7.51 – 7.47 (m, 2H), 7.36 – 7.27 (m, 3H), 5.44 (s, 1H), 5.39 (d, J = 0.6 Hz, 1H), 2.68 – 2.62 (m, 2H), 1.92 – 1.87 (m, 2H), 1.82 – 1.71 (m, 1H), 1.48 (dd, J_1 = 15.0 Hz, J_2 = 7.4 Hz, 2H), 1.34 – 1.16 (m, 4H), 0.88 (t, J = 7.2 Hz, 3H) ppm.

^{13}C NMR (101 MHz, $CDCl_3$) δ 152.0, 139.2, 128.1, 127.7, 127.5, 113.0, 73.9, 42.2, 36.8, 29.7, 25.9, 22.6, 14.1 ppm.

HRMS (CI+) Calcd for $C_{16}H_{22}O^+$ (M^+): 230.1615, Found: 230.1670.

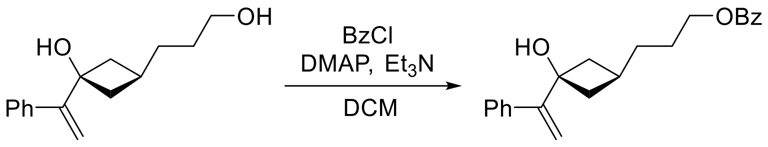

2i

***cis*-3-Hydroxy-3-(1-phenylvinyl)cyclobutyl)propyl benzoate (2i).** At 0 °C, To a solution of *cis*-3-(3-hydroxypropyl)-1-(1-phenylvinyl)cyclobutan-1-ol (161.6 mg, 0.7 mmol, 1.0 equiv), DMAP (42.7 mg, 0.35 mmol, 0.5 equiv), and triethylamine (354 mg, 3.5 mmol, 5.0 equiv) in anhydrous DCM 3 mL) was added benzoyl chloride (108.2 mg, 0.77 mmol, 1.1 equiv) dropwise. The mixture was allowed to warm to room temperature and stirred for 10 min. Brine was then added, and the organic layer was separated. The aqueous layer was extracted with DCM (20 mL × 3). The combined organic layers were dried over anhydrous Na_2SO_4, filtered, and concentrated. The residue was purified by flash column chromatography (eluent: *n*-hexane/EtOAc = 10:1) on silica gel to afford the desired product **2i** as a colorless oil (216 mg, 92% yield, *cis/trans* > 20:1).

^{1}H NMR (400 MHz, $CDCl_3$) δ 8.05 – 8.03 (m, 2H), 7.58 – 7.53 (m, 1H), 7.50 – 7.42 (m, 4H), 7.36 – 7.28 (m, 3H), 5.44 (s, 1H), 5.40 (s, 1H), 4.30 (t, *J* = 6.3 Hz, 1H), 2.71 – 2.67 (m, 2H), 2.01 – 1.81 (m, 4H), 1.75 – 1.62 (m, 4H) ppm.

^{13}C NMR (101 MHz, $CDCl_3$) δ 166.6, 151.9, 139.0, 132.8, 130.4, 129.5, 128.3, 128.2, 127.6, 127.5, 113.2, 73.8, 64.9, 42.0, 33.3, 26.7, 25.6 ppm.

HRMS (ES+) Calcd for $C_{22}H_{24}NaO_3^+$ $[M+Na]^+$: 359.1618, Found: 359.1626.

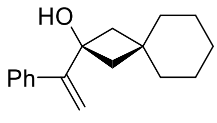

2j

2-(1-Phenylvinyl)spiro[3.5]nonan-2-ol (2j) was prepared as a yellow oil, which solidified when storage in a refrigerator, according to the General Procedure D (eluent:

n-hexane/EtOAc = 10:1, 19% yield).

¹H NMR (400 MHz, $CDCl_3$) δ 7.50 – 7.47 (m, 2H), 7.35 – 7.27 (m, 3H), 5.36 – 5.35 (m, 2H), 2.28 – 2.25 (m, 2H), 2.06 – 2.03 (m, 2H), 1.83 (s, 1H), 1.66 – 1.33 (m, 10H) ppm.

¹³C NMR (101 MHz, $CDCl_3$) δ 154.2, 139.1, 128.1, 127.5 (2C), 113.0, 73.7, 46.0, 39.2, 38.8, 31.3, 25.8, 23.0, 22.9 ppm.

HRMS (CI+) Calcd for $C_{17}H_{22}O^+$ (M^+): 242.1665, Found: 242.1671.

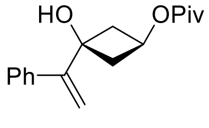

2l

***cis*-3-Hydroxy-3-(1-phenylvinyl)cyclobutyl pivalate (2l)** was prepared as a colorless oil according to the General Procedure D (eluent: *n*-hexane/EtOAc = 10:1, 28% yield, *cis*/*trans* > 20:1).

¹H NMR (400 MHz, $CDCl_3$) δ 7.47 – 7.45 (m, 2H), 7.36 – 7.30 (m, 3H), 5.42 (d, *J* = 11.6 Hz, 2H), 4.60 (p, *J* = 7.2 Hz, 1H), 3.04 – 2.99 (m, 2H), 2.41 – 2.36 (m, 2H), 2.17 (s, 1H), 1.19 (s, 9H) ppm.

¹³C NMR (101 MHz, $CDCl_3$) δ 178.2, 151.3, 138.5, 128.3, 127.8, 127.6, 113.9, 71.2, 61.5, 43.8, 38.5, 27.1 ppm.

HRMS (ES+) Calcd for $C_{17}H_{22}NaO_3^+$ $[M+Na]^+$: 297.1461, Found: 297.1461.

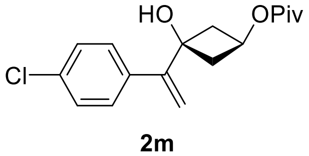

2m

***cis*-3-(1-(4-Chlorophenyl)vinyl)-3-hydroxycyclobutyl pivalate (2m)** was prepared as a yellow solid according to the General Procedure D (eluent: *n*-hexane/EtOAc = 10:1, 10% yield, *cis*/*trans* > 20:1).

¹H NMR (400 MHz, $CDCl_3$) δ 7.43 – 7.39 (m, 2H), 7.31 – 7.28 (m, 2H), 5.43 (d, *J* = 12.1 Hz, 2H), 4.58 (p, *J* = 7.1 Hz, 1H), 3.00 – 2.94 (m, 2H), 2.39 – 2.33 (m, 2H), 2.18 (s, 1H),

1.19 (s, 9H) ppm.

^{13}C NMR (101 MHz, $CDCl_3$) δ 178.2, 150.2, 136.9, 133.7, 128.9, 128.4, 114.5, 71.2, 61.4, 43.6, 38.4, 27.1 ppm.

HRMS (ES+) Calcd for $C_{17}H_{21}ClNaO_3^+$ $[M+Na]^+$: 331.1071, Found: 331.1076.

IV. Synthesis of the Phase-Transfer Catalysts

PTC-1, **PTC-2**, **PTC-4**, and **PTC-5** are known compounds.[4,5] **PTC-3** and **PTC-6** were prepared according to the literature procedure.[6]

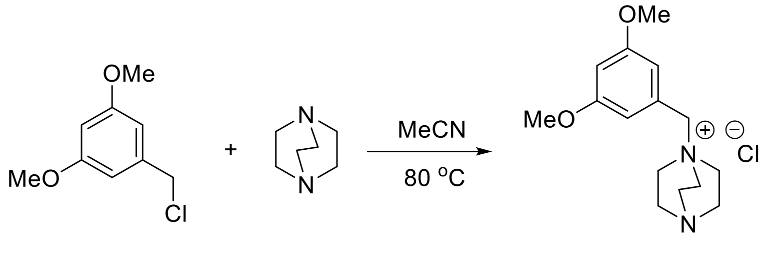

PTC-3

1-(3,5-Dimethoxybenzyl)-1,4-diazabicyclo[2.2.2]octan-1-ium chloride (PTC-3). A solution of 1-(chloromethyl)-3,5-dimethoxybenzene (559.8 mg, 3.0 mmol, 1.0 equiv) and DABCO (1.0 g, 9.0 mmol, 3.0 equiv) in MeCN (12 mL) was heated to 80 °C and stirred overnight. Then, Et_2O (20 mL) was added, and the generated white precipitate was filtered and washed with Et_2O (10 mL x 3) to give the pure product in 80% yield (717 mg).

^{1}H NMR (400 MHz, DMSO-d_6) δ 6.72 (d, J = 2.2 Hz, 2H), 6.69 (t, J = 2.2 Hz, 1H), 4.47 (s, 2H), 3.82 (s, 6H), 3.36 – 3.33 (m, 6H), 3.07 – 3.03 (m, 6H) ppm.

^{13}C NMR (101 MHz, DMSO-d_6) δ 160.6, 129.0, 111.2, 101.5, 66.5, 55.4, 51.8, 44.7 ppm.

HRMS (ES+) Calcd for $C_{15}H_{23}N_2O_2^+$ $[M-Cl]^+$: 263.1754, Found: 263.1756.

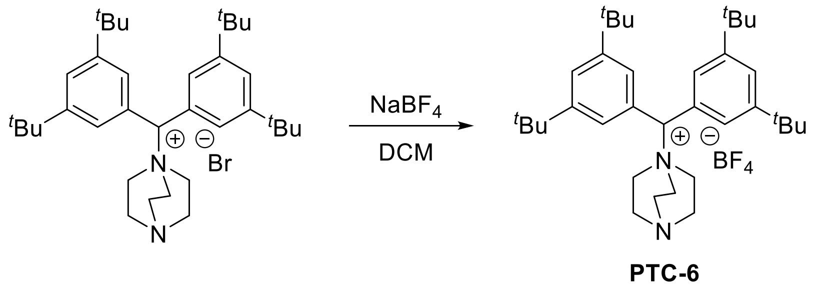

PTC-6

1-(Bis(3,5-di-*tert*-butylphenyl)methyl)-1,4-diazabicyclo[2.2.2]octan-1-ium tetrafluoroborate (PTC-6). A solution of 1-(bis(3,5-di-*tert*-butylphenyl)methyl)-1,4-diazabicyclo[2.2.2]octan-1-ium chloride (50 mg, 0.1 mmol, 1.0 equiv), $NaBF_4$ (55 mg, 0.5 mmol, 5.0 equiv) and a few drops of acetone in DCM (0.2 mL) was stirred

vigorously for 24 h. Then, water was added and the mixture was extracted by DCM (2 mL × 3). The combined organic layers were dried over anhydrous Na_2SO_4, filtered, and concentrated to afford the target product as a white solid (43 mg, 73% yield).

^{1}H NMR (400 MHz, DMSO-d_6) δ 7.63 (d, J = 1.3 Hz, 4H), 7.55 (s, 2H), 5.90 (s, 1H), 3.35 (t, J = 7.0 Hz, 6H), 3.08 (t, J = 7.0 Hz, 6H), 1.37 (s, 36H) ppm.

^{13}C NMR (101 MHz, DMSO- d_6) δ 151.3, 131.6, 125.7, 123.6, 81.8, 51.0, 45.0, 34.7, 31.1 ppm.

^{19}F NMR (376 MHz, DMSO- d_6) δ 148.3 ppm.

HRMS (ES+) Calcd for $C_{35}H_{55}N_2^+$ [M-Cl]$^+$: 503.4360, Found: 503.4364.

V. Reaction Condition Optimization

Table S1. Optimization of Other Parameters

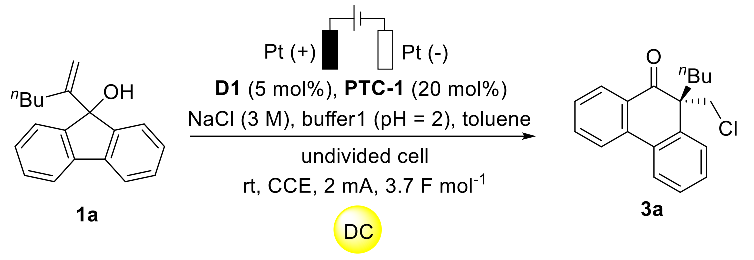

entry[a]	deviation from above	yield[b]	ee[c]
1	none	99%	80%
2[d]	buffer2 (pH = 6.4) instead of buffer1	25%	55%
3[e]	buffer3 (pH = 4.5) instead of buffer1	15%	80%
4[f]	buffer4 (pH = 7.0) instead of buffer1	18%	75%
5	LiCl instead of NaCl	90%	73%
6	KCl instead of NaCl	96%	74%

[a]Reaction conditions: **1a** (0.02 mmol), **D1** (5 mol%), **PTC-1** (20 mol%), NaCl (3 M aq.), buffer (2 mL), toluene (1.5 mL), Pt anode and cathode (10 mm × 25 mm × 0.1 mm), stirring speed: 1500 r/min, 2 mA, rt, 1 h. [b]The yields were determined by ^{1}H NMR analysis of the crude reaction mixture with CH_2Br_2 as internal standard. [c]The ee values were determined by chiral HPLC. [d]Buffer2: $c(NaH_2PO_4)/c(Na_2HPO_4)$ = 0.5 M/0.5 M (pH = 6.4). [e]Buffer3: *c*(HOAc)/*c*(NaOAc) = 0.5 M/0.5 M (pH = 4.5). [f]Buffer4 (pH = 7) was commercially available from ThermoFisher®.

Table S2. Effect of Current Densities

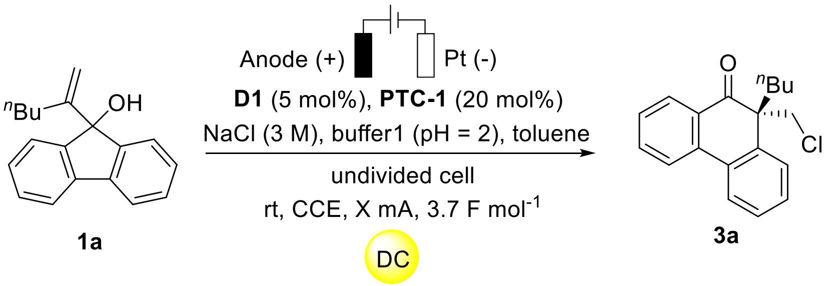

entry[a]	X mA	yield[b]	ee[c]
1	0.5 mA	99%	78%
2	1 mA	99%	80%
3	2 mA	99%	80%
4	3 mA	99%	81%

[a]Reaction conditions: **1a** (0.02 mmol), **D1** (5 mol%), **PTC-1** (20 mol%), NaCl (3 M aq.), buffer1 (2 mL), toluene (1.5 mL), corresponding anode and platinum cathode (10 mm × 25 mm × 0.1 mm), stirring speed: 1500 r/min, CCE, X mA, rt, 3.7 F·mol^{-1}. [b]The yields were determined by ^{1}H NMR analysis of the crude reaction mixture with CH_2Br_2 as internal standard. [c]The ee values were determined by chiral HPLC.

Table S3. Evaluation of PTCs[a,b,c]

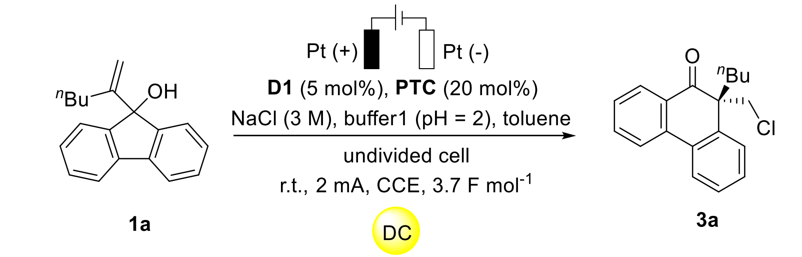

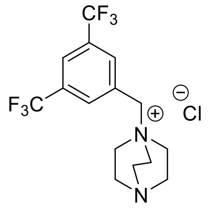

PTC-1
99% yield, 80% ee

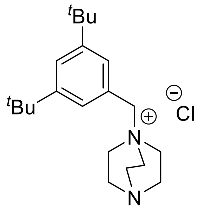

PTC-2
99% yield, 91% ee

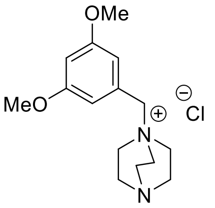

PTC-3
99% yield, 72% ee

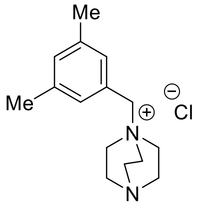

PTC-4
99% yield, 60% ee

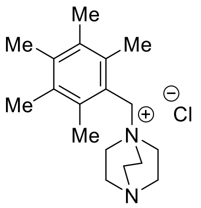

PTC-5
99% yield, 80% ee

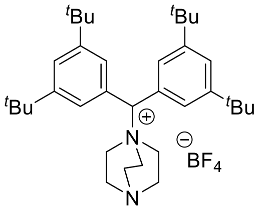

PTC-6
99% yield, 85.5% ee

[a]Reaction conditions: **1a** (0.02 mmol), **D1** (5 mol%), **PTC** (20 mol%), NaCl (3 M aq.), buffer1 (2 mL), toluene (1.5 mL), Pt anode and cathode (10 mm × 25 mm × 0.1 mm), stirring speed: 1500 r/min, CCE, 2 mA, rt, 1 h. [b]The yields were determined by ^{1}H NMR analysis of the crude reaction mixture with CH_2Br_2 as internal standard. [c]The ee values were determined by chiral HPLC.

Table S4. Effect of the Anodes

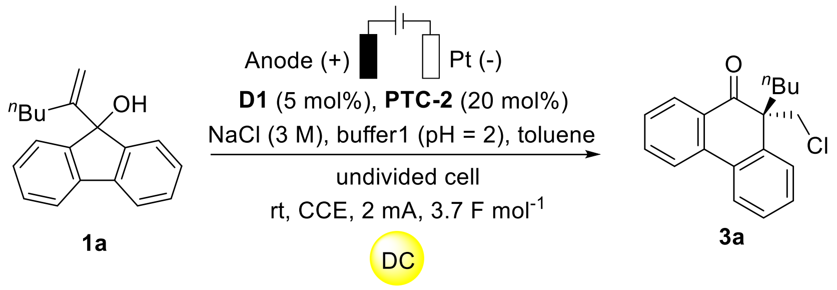

entry[a]	anode	yield[b]	ee[c]
1	Pt	99%	91%
2	GC	99%	86%
3	CF	7%	86%
4	graphite	56%	79%

[a]Reaction conditions: **1a** (0.02 mmol), **D1** (5 mol%), **PTC-2** (20 mol%), NaCl (3 M aq.), buffer1 (2 mL), toluene (1.5 mL), corresponding anode and platinum cathode (10 mm × 25 mm × 0.1 mm), stirring speed: 1500 r/min, CCE, 2 mA, rt, 1 h. [b]The yields were determined by ^{1}H NMR analysis of the crude reaction mixture with CH_2Br_2 as internal standard. [c]The ee values were determined by chiral HPLC.

Table S5. Optimization of Solvents

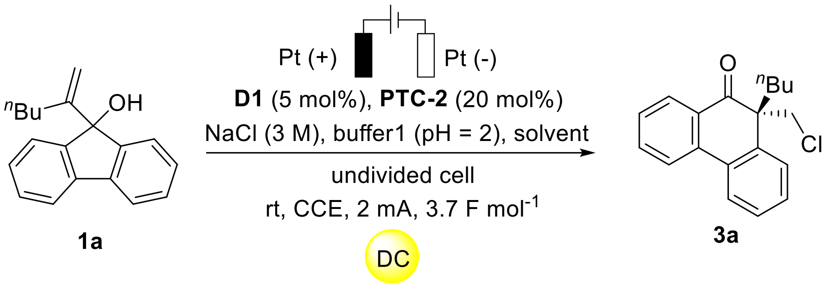

entry[a]	solvent	yield[b]	ee[c]
1	toluene	99%	91%
2	DCM	99%	0
3	EtOAc	79%	0
4	MeCN	56%	0
5	THF	74%	0
6	*n*-hexane	72%	38%
7	acetone	90%	0

[a]Reaction conditions: **1a** (0.02 mmol), **D1** (5 mol%), **PTC-2** (20 mol%), NaCl (3 M aq.), buffer1 (2 mL), solvent (1.5 mL), platinum anode and cathode (10 mm × 25 mm × 0.1 mm), stirring speed: 1500 r/min, CCE, 2 mA, rt, 1 h. [b]The yields were determined by ^{1}H NMR analysis of the crude reaction mixture with CH_2Br_2 as internal standard. [c]The ee values were determined by chiral HPLC.

Table S6. Effect of the pH Values within A Narrow Range

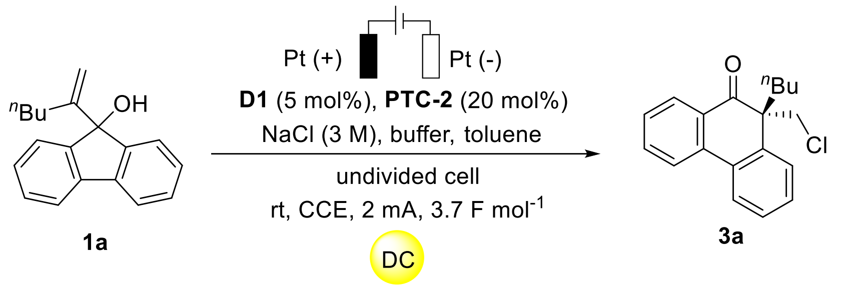

entry[a]	bufferX	yield[b]	ee[c]
1	buffer5 (pH =1.7)	92%	92%
2	buffer6 (pH = 2.6)	96%	92%
3	buffer7 (pH = 3.0)	90%	93%

[a]Reaction conditions: **1a** (0.02 mmol), (*R*)-**D1** (5 mol%), **PTC-2** (20 mol%), NaCl (3 M aq.), buffer (2 mL), toluene (1.5 mL), platinum anode and cathode (10 mm × 25 mm × 0.1 mm), stirring speed: 1500 r/min, CCE, 2 mA, rt, 1 h. [b]The yields were determined by ^{1}H NMR analysis of the crude reaction mixture with CH_2Br_2 as internal standard. [c]The ee values were determined by chiral HPLC.

Table S7. Optimization for AC Condition under 0.3-mmol Scale.

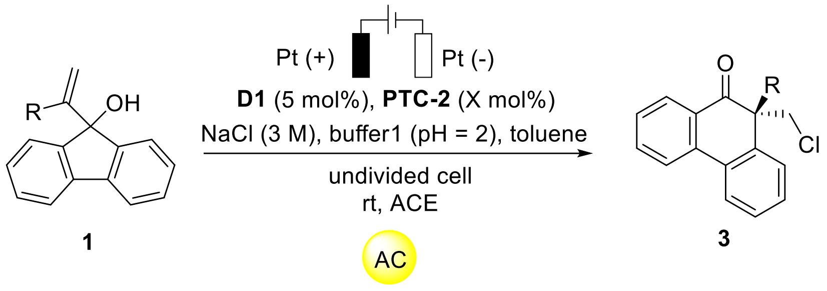

entry[a]	R	X	wave form	yield[b]	ee[c]
1	Ph (**1k**)	20	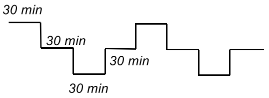 30 min; 2 mA, 0 mA, -2 mA	96%	96%
2	Ph (**1k**)	10	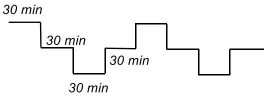 30 min; 2 mA, 0 mA, -2 mA	72%	95%
3	Ph (**1k**)	10	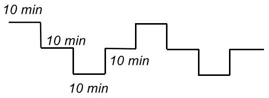 10 min; 2 mA, 0 mA, -2 mA	96%	96%
4	Ph (**1k**)	5	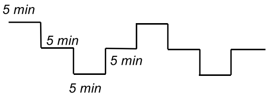 5 min; 2 mA, 0 mA, -2 mA	94.5%	95.7%
5	Ph (**1k**)	2	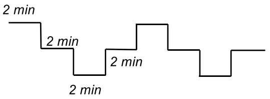 2 min; 2 mA, 0 mA, -2 mA	93%	94.4%
6	Ph (**1k**)	1	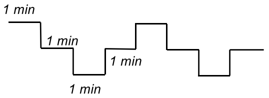 1 min; 2 mA, 0 mA, -2 mA	92.5%	92%
7	Ph (**1k**)	0.5	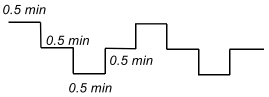 0.5 min; 2 mA, 0 mA, -2 mA	78%	90.3%
8	^{n}Bu (**1a**)	5	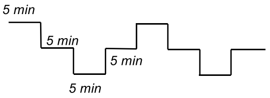 5 min; 2 mA, 0 mA, -2 mA	41%	89%
9	*p*-ClC$_6$H$_4$ (**1p**)	5	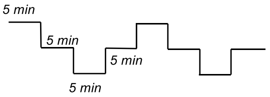 5 min; 2 mA, 0 mA, -2 mA	29%	93%
10	*p*-ClC$_6$H$_4$ (**1p**)	10	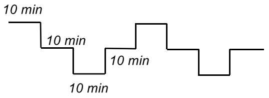 10 min; 2 mA, 0 mA, -2 mA	47%	91%
11	*p*-ClC$_6$H$_4$ (**1p**)	10	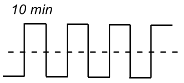 10 min; 2 mA, 0 mA, -2 mA	89%	99%

12	Cy (**1l**)	5	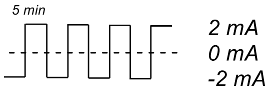	99%	92.4%
13	Cy (**1l**)	10	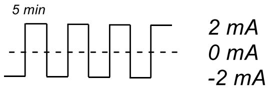	99%	93%
14	Ph (**1k**)	10	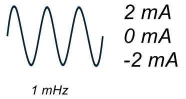	94%	96%

[a]Reaction conditions: **1** (0.3 mmol), **D1** (5 mol%), **PTC-2** (0.5–20 mol%), NaCl (3 M aq.), buffer1 (H_3PO_4/NaH_2PO_4=0.5 M/0.5 M, pH = 2, 10 mL), toluene (7.5 mL), Pt anode and cathode (15 mm × 15 mm × 0.2 mm), stirring speed: 1500 r/min, AC, rt, maximum 3.7 F/mol. [b]Isolated yield was obtained. [c]Ee value was determined by chiral HPLC.

At certain stage of the optimization, a white anodic residue was observed and was identified as an analogue of **PTC-2**. Then, the alternating current (AC) electrolysis was hypothesized to solve the problem. Initially, we used a square-wave AC with a resting period between two reversed currents for electrolysis. It was efficient for substrate **1k** when 20 mol% of **PTC-2** was used (Table S7, entry 1). Then, we gradually reduced the loading of **PTC-2**. However, sole decreasing the **PTC-2** loading from 20 mol% to 10 mol%, without modification of the electrolysis condition, led to incomplete conversion (entry 2). When the electrolysis frequency increased together with the decreased loading, the reaction went completion again (entry 3). With this logic, we further decreased the loading of **PTC-2**, which indicated that a slight decrease in both yield and ee was observed, but the reaction remained overall efficient even at 1 mol% loading of **PTC-2** (entries 4–7). Other substrates, such as **1a** and **1q**, were then evaluated with 5 mol% of **PTC-2**. However, these reactions halted again before completion (entries 8–9). Therefore, with **1q** as the model substrate, we continued to optimize the 0.3-mmol scale reaction. Increasing the PTC loading showed no effect on the outcome (entry 10), but eliminating the resting time during electrolysis could lead to complete conversion (entry 11). The similar strategy was evaluated with substrate

1l by utilization of both 5 mol% and 10 mol% loadings of PTC, which worked equally well in both cases (entries 12–13). Because different substrates showed different reaction rates, a 10 mol% **PTC-2** loading was chosen uniformly as the standard condition for substrate scope evaluation (entry 13). We also tested sine-wave electrolysis under other optimized conditions. At 1 mHz frequency and 2 mA amplitude with the same charge maintained in a single period, similarly high yield and enantioselectivity could be obtained (entry 14).

VI. Electrochemical Chlorinative Semipinacol Rearrangement

General Procedure E

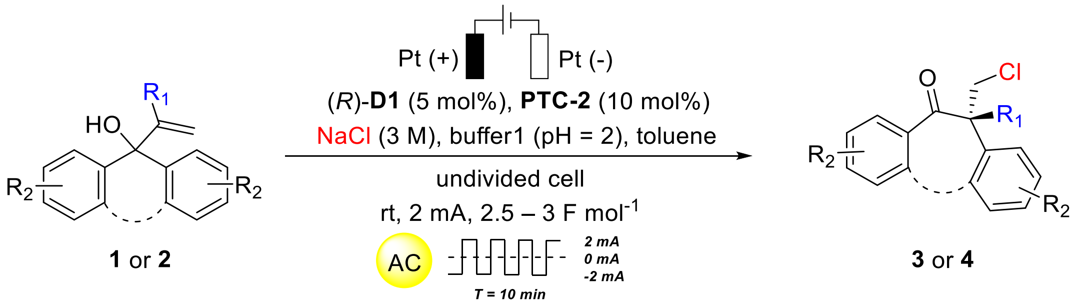

To a 25-mL vial equipped with a platinum anode (15 × 15 × 0.2 mm), a platinum cathode (15 × 15 × 0.2 mm), and two magnetic stir bars (cylindrical, Ø6 × 10 mm) were added the substrate **1** or **2** (0.3 mmol), (*R*)-**D1** (11.5 mg, 0.015 mmol, 5 mol%), **PTC-2** (10.6 mg, 0.03 mmol, 10 mol%), NaCl (1.75 g), toluene (7.5 mL) and the H_3PO_4–NaH_2PO_4 buffer1 ($c(H_3PO_4) = c(NaH_2PO_4) = 0.5$ M, pH = 2, 10 mL). The vial was placed on a stir plate with a stirring speed of 1500 r/min (caution: The stirring must allow full mixing of the two phases but avoid contact between the stir bars and electrodes). The electrolysis was carried out with a current of 2 mA and alternating phase every 5 min and the total electrolysis lasted for about 10 – 12 h (2.5 – 3.0 F·mol^{-1}) until substrate **1** or **2** disappeared by TLC analysis. The layers were separated and the aqueous layer was extracted with EtOAc (5 mL × 3). The combined organic layers were dried over anhydrous Na_2SO_4, filtered, and concentrated. The residue was purified by silica gel chromatography to yield the desired product **3** or **4**.

General Procedure F

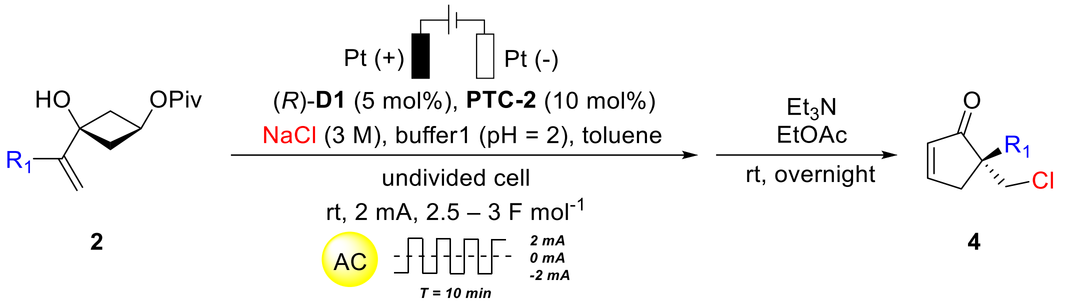

2 **4**

To a 25-mL vial equipped with a platinum anode (15 × 15 × 0.2 mm), a platinum cathode (15 × 15 × 0.2 mm), and two magnetic stir bars (cylindrical, Ø6 × 10 mm) were added the substrate **2** (0.3 mmol), (*R*)-**D1** (11.5 mg, 0.015 mmol, 5 mol%), **PTC-2** (10.6 mg, 0.03 mmol, 10 mol%), NaCl (1.75 g), toluene (7.5 mL), and the H_3PO_4–NaH_2PO_4 buffer1 ($c(H_3PO_4) = c(NaH_2PO_4) = 0.5$ M, pH = 2, 10 mL). The vial was placed on a stir plate with a stirring speed of 1500 r/min (caution: The stirring must allow full mixing of the two phases but avoid contact between the stir bars and electrodes). The electrolysis was carried out with a current of 2 mA and alternating phase every 5 min and the total electrolysis lasted for about 10 – 12 h (2.5 – 3.0 F·mol^{-1}) until substrate **2** disappeared by TLC analysis. The layers were separated, and the aqueous layer was extracted with EtOAc (5 mL × 3). The combined organic layers were dried over anhydrous Na_2SO_4, filtered, and concentrated. The residue was dissolved in EtOAc (15 mL) and Et_3N (1.5 mL), and the mixture was stirred at room temperature overnight. Then, the solvent was removed by evaporation in vacuum directly. The residue was purified by silica gel chromatography to yield the desired product **4**.

Unless otherwise noted, the racemic samples were prepared according to the General Procedure E or F in the absence of a chiral phosphoric acid catalyst.

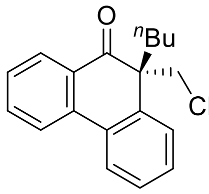

3a

(*R*)-10-Butyl-10-(chloromethyl)phenanthren-9(10*H*)-one (3a) was prepared as a colorless oil from **1a** (79.3 mg, 0.3 mmol) according to the General Procedure E (11 h, eluent: *n*-hexane/EtOAc = 30:1, 90.1 mg, 99% yield, 92% ee).

$[\alpha]_D^{25}$: +77.1 (*c* = 1.0, CH_2Cl_2). HPLC analysis of product: Daicel CHIRALCEL® OD-3 column; 5% *i*-PrOH in *n*-hexane; 1.0 mL/min; retention time: 6.9 min (major), 8.4 min (minor).

^{1}H NMR (400 MHz, $CDCl_3$) δ 8.20 (dd, J_1 = 7.8, J_2 = 1.4 Hz, 1H), 8.12 – 8.08 (m, 2H), 7.72 – 7.67 (m, 1H), 7.49 – 7.42 (m, 4H), 4.35 (d, *J* = 10.5 Hz, 1H), 3.88 (d, *J* = 10.5 Hz, 1H), 2.14 (td, J_1 = 12.6, J_2 = 4.8 Hz, 1H), 1.79 (td, *J* = 12.6, 4.6 Hz, 1H), 1.18 – 1.00 (m, 2H), 0.95 – 0.78 (m, 2H), 0.68 (t, *J* = 7.3 Hz, 3H) ppm.

^{13}C NMR (101 MHz, $CDCl_3$) δ 199.6, 138.8, 137.2, 135.0, 131.2, 129.5, 129.3, 128.3, 127.6 (2C), 126.7, 123.7, 123.2, 56.4, 51.4, 41.5, 26.3, 22.8, 13.6 ppm.

HRMS (CI+) Calcd for $C_{19}H_{20}ClO^+$ $[M+H]^+$: 299.1197, Found: 299.1203.

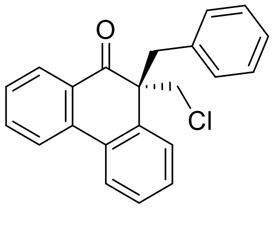

3b

(*R*)-10-Benzyl-10-(chloromethyl)phenanthren-9(10*H*)-one (3b) was prepared as a white solid from **1b** (89.5 mg, 0.3 mmol) according to the General Procedure E (11 h, eluent: *n*-hexane/EtOAc = 30:1, 94.6 mg, 95% yield, 95% ee).

$[\alpha]_D^{25}$: +168.2 (*c* = 1.0, CH_2Cl_2). HPLC analysis of product: Daicel CHIRALPAK® AS-3 column; 3% *i*-PrOH in *n*-hexane; 1.0 mL/min; retention time: 9.6 min (minor), 11.0 min (major).

^{1}**H NMR** (400 MHz, $CDCl_3$) δ 8.11 (dd, *J*$_1$ = 7.8, *J*$_2$ = 1.3 Hz, 1H), 7.99 – 7.94 (m, 1H), 7.86 (d, *J* = 8.1 Hz, 1H), 7.59 – 7.54 (m, 1H), 7.47 – 7.33 (m, 4H), 6.99 – 6.89 (m, 3H), 6.57 – 6.54 (m, 2H), 4.62 (d, *J* = 10.7 Hz, 1H), 4.06 (d, *J* = 10.7 Hz, 1H), 3.25 (d, *J* = 12.9 Hz, 1H), 3.02 (d, *J* = 12.9 Hz, 1H) ppm.

^{13}**C NMR** (101 MHz, $CDCl_3$) δ 198.7, 137.3, 136.9, 134.7, 134.2, 131.4, 129.9, 129.3, 128.9, 128.1, 127.8, 127.6 (2C), 127.5, 126.8, 123.8, 122.9, 58.1, 48.6, 48.2 ppm.

HRMS (ES+) Calcd for $C_{22}H_{17}ClNaO^+$ [M+Na]$^+$: 355.0860, Found: 355.0869.

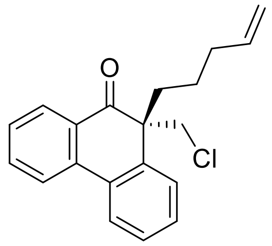

3c

(*R*)-10-(Chloromethyl)-10-(pent-4-en-1-yl)phenanthren-9(10*H*)-one (3c) was prepared as a colorless oil from **1c** (82.3 mg, 0.3 mmol) according to the General Procedure E (11 h, eluent: *n*-hexane/EtOAc = 30:1, 71.4 mg, 77% yield, 91% ee).

$[\alpha]_D^{25}$: +66.1 (*c* = 1.0, CH_2Cl_2). HPLC analysis of product: Daicel CHIRALPAK® IB N-3 column; 5% *i*-PrOH in *n*-hexane; 1.0 mL/min; retention time: 7.5 min (major), 8.4 min (minor).

^{1}**H NMR** (400 MHz, $CDCl_3$) δ 8.19 (dd, *J*$_1$ = 7.8, *J*$_2$ = 1.2 Hz, 1H), 8.12 – 8.08 (m, 2H), 7.70 (ddd, *J*$_1$ = 8.2, *J*$_2$ = 7.3, *J*$_3$ = 1.5 Hz 1H), 7.49 – 7.42 (m, 4H), 5.59 – 5.49 (m, 1H), 4.85 (t, *J* = 1.2 Hz, 1H), 4.82 (ddd, *J*$_1$ = 5.0, *J*$_2$ = 2.7, *J*$_3$ = 1.3 Hz, 1H), 4.33 (d, *J* = 10.5 Hz, 1H), 3.86 (d, *J* = 10.5 Hz, 1H), 2.15 (td, *J*$_1$ = 12.6, *J*$_2$ = 4.8 Hz, 1H), 1.89 – 1.77 (m, 3H), 1.09 – 0.88 (m, 2H) ppm.

^{13}**C NMR** (101 MHz, $CDCl_3$) δ 199.5, 138.7, 137.7, 137.2, 135.0, 130.5, 131.3, 129.5, 129.4, 128.3, 127.7, 126.8, 123.8, 123.2, 115.1, 56.4, 51.4, 40.9, 33.7, 23.5 ppm.

HRMS (ES+) Calcd for $C_{20}H_{19}ClNaO^+$ [M+Na]$^+$: 333.1017, Found: 333.1024.

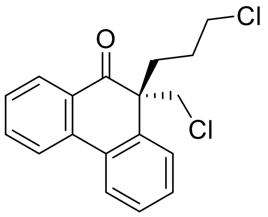

3d

(*R*)-10-(Chloromethyl)-10-(3-chloropropyl)phenanthren-9(10*H*)-one (3d) was prepared as a white solid from **1d** (85.4 mg, 0.3 mmol) according to the General Procedure E (11 h, eluent: *n*-hexane/EtOAc = 30:1, 76.9 mg, 80% yield, 93% ee).

$[\alpha]_D^{25}$: +47.3 (c = 1.0, CH_2Cl_2). HPLC analysis of product: Daicel CHIRALCEL® OD-3 column; 5% *i*-PrOH in *n*-hexane; 1.0 mL/min; retention time: 11.3 min (major), 13.8 min (minor).

^{1}H NMR (400 MHz, $CDCl_3$) δ 8.18 (d, J = 7.8 Hz, 1H), 8.10 (t, J = 7.2 Hz, 2H), 7.73 – 7.69 (m, 1H), 7.48 – 7.43 (m, 4H), 4.26 (d, J = 10.6 Hz, 1H), 3.85 (d, J = 10.6 Hz, 1H), 3.36 – 3.27 (m, 2H), 2.32 (td, J_1 = 12.5, J_2 = 4.7 Hz, 1H), 2.03 (td, J_1 = 12.5, J_2 = 4.4 Hz, 1H), 1.49 – 1.26 (m, 2H) ppm.

^{13}C NMR (101 MHz, $CDCl_3$) δ 199.1, 137.9, 137.2, 135.2, 131.3, 129.6, 129.3, 128.5, 128.0, 127.8, 126.9, 123.9, 123.3, 55.8, 51.7, 44.6, 37.7, 27.5 ppm.

HRMS (ES+) Calcd for $C_{18}H_{17}Cl_2O^+$ $[M+H]^+$: 319.0651, Found: 319.0657.

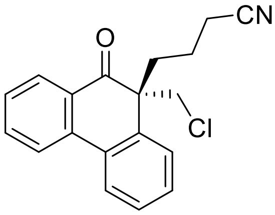

3e

(*R*)-4-(9-(Chloromethyl)-10-oxo-9,10-dihydrophenanthren-9-yl)butanenitrile (3e) was prepared as a white solid from **1e** (82.6 mg, 0.3 mmol) according to the General Procedure E (11 h, eluent: *n*-hexane/EtOAc = 10:1, 96.7 mg, 99% yield, 82% ee).

$[\alpha]_D^{25}$: +33.8 (c = 1.0, CH_2Cl_2). HPLC analysis of product: Daicel CHIRALPAK® IE-3 column; 20% *i*-PrOH in *n*-hexane; 1.0 mL/min; retention time: 13.2 min (minor), 14.6

min (major).

^{1}H NMR (400 MHz, $CDCl_3$) δ 8.17 (dd, J_1 = 7.8, J_2 = 1.4 Hz, 1H), 8.13 – 8.08 (m, 2H), 7.75 – 7.71 (m, 1H), 7.54 – 7.45 (m, 4H), 4.20 (d, J = 10.6 Hz, 1H), 3.83 (d, J = 10.6 Hz, 1H), 2.40 – 2.32 (m, 1H), 2.23 – 2.10 (m, 2H), 2.01 (td, J_1 = 12.6, J_2 = 4.8 Hz, 1H), 1.37 – 1.18 (m, 2H) ppm.

^{13}C NMR (101 MHz, $CDCl_3$) δ 198.9, 137.4, 137.1, 135.4, 131.2, 129.7, 129.2, 128.6, 128.3, 127.8, 126.7, 124.1, 123.2, 118.9, 55.7, 51.8, 38.7, 20.6, 17.3 ppm.

HRMS (ES+) Calcd for $C_{19}H_{16}ClNNaO^+$ $[M+Na]^+$: 332.0813, Found: 332.0826.

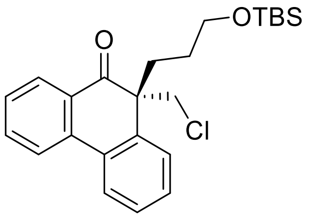

3f

(*R*)-10-(3-((*tert*-Butyldimethylsilyl)oxy)propyl)-10-(chloromethyl)phenanth-ren-9(10*H*)-one (3f) was prepared as a white solid from **1f** (114.2 mg, 0.3 mmol) according to the General Procedure E (11 h, eluent: *n*-hexane/EtOAc = 30:1, 121.7 mg, 98% yield, 92% ee).

$[\alpha]_D^{25}$: +56.0 (c = 1.0, CH_2Cl_2). HPLC analysis of product: Daicel CHIRALPAK® IB N-3 column; 5% *i*-PrOH in *n*-hexane; 1.0 mL/min; retention time: 6.0 min (major), 6.4 min (minor).

^{1}H NMR (400 MHz, $CDCl_3$) δ 8.19 (dd, J_1 = 7.8, J_2 = 1.3 Hz, 1H), 8.11 – 8.07 (m, 2H), 7.69 (td, J_1 = 8.7, J_2 = 4.4 Hz, 1H), 7.46 – 7.41 (m, 4H), 4.33 (d, J = 10.5 Hz, 1H), 3.88 (d, J = 10.5 Hz, 1H), 3.41 – 3.32 (m, 2H), 2.21 – 2.13 (m, 1H), 1.93 (td, J_1 = 12.5, J_2 = 4.4 Hz, 1H), 1.21 – 1.02 (m, 2H), 0.80 (s, 9H), -0.09 (d, J = 9.0 Hz, 6H) ppm.

^{13}C NMR (101 MHz, $CDCl_3$) δ 199.4, 138.5, 137.2, 135.0, 131.2, 129.5, 129.4, 128.3, 127.7 (2C), 126.9, 123.8, 123.2, 56.1, 51.4, 37.7, 27.6, 25.9, 18.2, -5.4 (2C) ppm.

HRMS (ES+) Calcd for $C_{24}H_{31}ClNaO_2Si^+$ $[M+Na]^+$: 437.1674, Found: 337.1682.

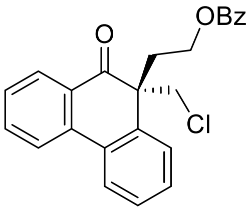

3g

(*R*)-2-(9-(Chloromethyl)-10-oxo-9,10-dihydrophenanthren-9-yl)ethyl benzoate (3g) was prepared as a white solid from **1g** (107 mg, 0.3 mmol) according to the General Procedure E (11 h, eluent: *n*-hexane/EtOAc = 10:1, 126.6 mg, 99% yield, 91% ee).

$[\alpha]_D^{25}$: -124.5 (*c* = 1.0, CH_2Cl_2). HPLC analysis of product: Daicel CHIRALCEL® OD-3 column; 10% *i*-PrOH in *n*-hexane; 1.0 mL/min; retention time: 14.4 min (major), 16.3 min (minor).

^{1}H NMR (400 MHz, $CDCl_3$) δ 8.10 (dd, J_1 = 12.3, J_2 = 4.3 Hz, 2H), 8.03 (d, *J* = 8.1 Hz, 1H), 7.64 – 7.60 (m, 3H), 7.50 – 7.41 (m, 4H), 7.32 – 7.26 (m, 3H), 4.30 (d, *J* = 10.4 Hz, 1H), 4.12 (dt, J_1 = 11.3, J_2 = 5.6 Hz, 1H), 3.93 – 3.87 (m, 2H), 2.85 (ddd, J_1 = 14.5, J_2 = 8.6, J_3 = 6.2 Hz, 1H), 2.33 (dt, J_1 = 13.9, J_2 = 5.3 Hz, 1H) ppm.

^{13}C NMR (101 MHz, $CDCl_3$) δ 198.4, 166.0, 137.3, 136.9, 135.0, 132.8, 131.3, 129.5 (3C), 129.1, 128.2, 128.1 (2C), 128.0, 126.9, 124.1, 123.2, 61.0, 54.2, 52.6, 38.9 ppm.

HRMS (ES+) Calcd for $C_{24}H_{19}ClNaO_3^+$ $[M+Na]^+$: 413.0915, Found: 413.0922.

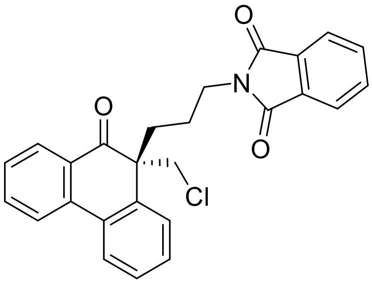

3h

(*R*)-2-(3-(9-(Chloromethyl)-10-oxo-9,10-dihydrophenanthren-9-yl)propyl)-isoindoline-1,3-dione (3h) was prepared as a white solid from **1h** (118.6 mg, 0.3 mmol) according to the General Procedure E (11 h, eluent: *n*-hexane/EtOAc = 10:1, 127.2 mg, 99% yield, 90% ee).

$[\alpha]_D^{25}$: +32.8 (c = 1.0, CH_2Cl_2). HPLC analysis of product: Daicel CHIRALCEL® OD-3 column; 20% *i*-PrOH in *n*-hexane; 1.0 mL/min; retention time: 16.8 min (minor), 18.0 min (major).

^{1}H NMR (400 MHz, $CDCl_3$) δ 8.14 (dd, J_1 = 7.8, J_2 = 0.9 Hz, 1H), 8.07 (t, J = 6.8 Hz, 2H), 7.80 – 7.76 (m, 2H), 7.71 – 7.66 (m, 3H), 7.44 – 7.36 (m, 4H), 4.26 (d, J = 10.6 Hz, 1H), 3.84 (d, J = 10.5 Hz, 1H), 3.47 (t, J = 6.8 Hz, 2H), 2.23 (td, J_1 = 12.6, J_2 = 4.9 Hz, 1H), 1.86 (td, J_1 = 12.7, J_2 = 4.2 Hz, 1H), 1.40 – 1.17 (m, 2H) ppm

^{13}C NMR (101 MHz, $CDCl_3$) δ 198.9, 168.2, 138.1, 137.1, 135.1, 134.0, 132.0, 131.3, 129.5, 129.3, 128.4, 127.9, 127.8, 126.7, 123.9, 123.3, 123.2, 55.9, 51.4, 38.3, 37.8, 23.7 ppm.

HRMS (ES+) Calcd for $C_{26}H_{20}ClNNaO_3^+$ $[M+Na]^+$: 452.1024, Found: 452.1031.

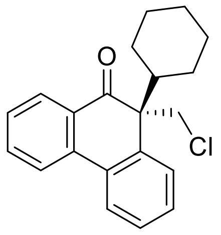

3i

(*R*)-10-(Chloromethyl)-10-cyclohexylphenanthren-9(10*H*)-one (3i) was prepared as a colorless oil from **1i** (87.2 mg, 0.3 mmol) according to the General Procedure E (11 h, eluent: *n*-hexane/EtOAc = 30:1, 98.5 mg, 99% yield, 93% ee).

$[\alpha]_D^{25}$: +241.1 (c = 1.0, CH_2Cl_2). HPLC analysis of product: Daicel CHIRALPAK® AD-3 column; 5% *i*-PrOH in *n*-hexane; 1.0 mL/min; retention time: 7.5 min (minor), 10.6 min (major).

^{1}H NMR (400 MHz, $CDCl_3$) δ 8.13 (dd, J_1 = 7.8, J_2 = 1.1 Hz, 1H), 8.07 – 8.04 (m, 2H), 7.69 – 7.65 (m, 1H), 7.46 – 7.41 (m, 4H), 4.72 (d, J = 10.6 Hz, 1H), 4.05 (d, J = 10.6 Hz, 1H), 1.79 (t, J = 11.6 Hz, 1H), 1.64 (d, J = 8.8 Hz, 2H), 1.55 – 1.43 (m, 3H), 1.12 – 0.87 (m, 4H), 0.80 (qd, J_1 = 12.3, J_2 = 3.3 Hz, 1H) ppm.

^{13}C NMR (101 MHz, $CDCl_3$) δ 199.1, 138.5, 137.3, 134.7, 131.7, 130.3, 128.7, 128.3, 127.7, 127.5, 127.3, 123.7, 123.1, 60.5, 60.0, 47.8, 28.2, 27.4, 26.9, 26.5, 25.9 ppm.

HRMS (ES+) Calcd for $C_{21}H_{21}ClNaO^+$ $[M+Na]^+$: 347.1173, Found: 347.1177.

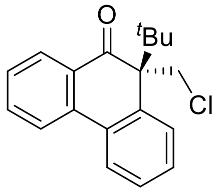

3j

(*R*)-10-(*tert*-Butyl)-10-(chloromethyl)phenanthren-9(10*H*)-one (3j) was prepared as a white solid from **1j** (77.0 mg, 0.29 mmol) according to the General Procedure E (11 h, eluent: *n*-hexane/EtOAc = 30:1, 84.2 mg, 98% yield, 94% ee).

$[\alpha]_D^{25}$: +279.8 (c = 1.0, CH_2Cl_2). HPLC analysis of product: Daicel CHIRALPAK® AD-3 column; 10% *i*-PrOH in *n*-hexane; 1.0 mL/min; retention time: 5.4 min (minor), 5.9 min (major).

^{1}H NMR (400 MHz, $CDCl_3$) δ 8.08 – 7.98 (m, 3H), 7.67 – 7.63 (m, 1H), 7.46 – 7.39 (m, 4H), 4.82 (d, J = 10.8 Hz, 1H), 4.17 (d, J = 10.8 Hz, 1H), 0.83 (s, 9H) ppm.

^{13}C NMR (101 MHz, $CDCl_3$) δ 198.8, 137.8, 135.9, 134.5, 132.7, 131.5, 130.0, 128.4, 127.7, 127.6, 127.1, 124.0, 122.8, 63.3, 45.2, 40.0, 26.9 ppm.

HRMS (CI+) Calcd for $C_{19}H_{19}ClNaO^+$ $[M+Na]^+$: 321.1017, Found: 321.1026

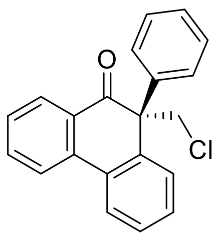

3k

(*R*)-10-(Chloromethyl)-10-phenylphenanthren-9(10*H*)-one (3k) was prepared as a white solid from **1k** (85.3 mg, 0.3 mmol) according to the General Procedure E (11 h, eluent: *n*-hexane/EtOAc = 30:1, 88.4 mg, 93% yield, 95% ee).

$[\alpha]_D^{25}$: +108.8 (c = 1.0, CH_2Cl_2). HPLC analysis of product: Daicel CHIRALPAK® AS-3 column; 5% *i*-PrOH in *n*-hexane; 1.0 mL/min; retention time: 7.4 min (minor), 9.1 min (major).

^{1}H NMR (400 MHz, $CDCl_3$) δ 8.12 – 8.09 (m, 1H), 8.02 (dd, J_1 = 12.7, J_2 = 7.9 Hz, 2H),

7.63 (t, J = 7.7 Hz, 1H), 7.54 – 7.47 (m, 2H), 7.43 – 7.41 (m, 1H), 7.36 (t, J = 7.5 Hz, 1H), 7.22 – 7.17 (m, 3H), 7.07 – 7.04 (m, 2H), 5.05 (d, J = 10.9 Hz, 1H), 4.16 (d, J = 10.9 Hz, 1H) ppm.

^{13}C NMR (101 MHz, $CDCl_3$) δ 196.9, 139.7, 137.2, 136.9, 134.8, 131.7, 129.3, 129.1, 129.0, 128.8, 128.4, 128.3 (2C), 128.1, 127.3, 124.4, 123.1, 61.3, 47.6 ppm.

HRMS (ES+) Calcd for $C_{21}H_{15}ClNaO^+$ $[M+Na]^+$: 341.0704, Found: 341.0715.

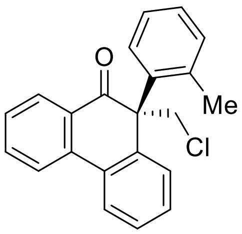

3l

(*R*)-10-(Chloromethyl)-10-(*o*-tolyl)phenanthren-9(10*H*)-one (3l) was prepared as a white solid from **1l** (89.5 mg, 0.3 mmol) according to the General Procedure E (11 h, eluent: *n*-hexane/EtOAc = 30:1, 98.6 mg, 99% yield, 93% ee).

$[\alpha]_D^{25}$: -55.1 (c = 1.0, CH_2Cl_2). HPLC analysis of product: Daicel CHIRALPAK® AD-3 column; 10% *i*-PrOH in *n*-hexane; 1.0 mL/min; retention time: 15.4 min (major), 19.0 min (minor).

^{1}H NMR (400 MHz, $CDCl_3$) δ 8.22 (d, J = 7.8 Hz, 1H), 8.16 (d, J = 8.0 Hz, 2H), 8.11 (d, J = 8.0 Hz, 1H), 7.76 (td, J_1 = 8.1 Hz, J_2 = 1.5 Hz, 1H), 7.66 (d, J = 7.8 Hz, 1H), 7.52 – 7.48 (m, 1H), 7.43 – 7.39 (m, 1H), 7.36 (t, J = 7.6 Hz, 1H), 7.27 (td, J_1 = 7.5 Hz, J_2 = 1.0 Hz, 1H), 7.22 (td, J_1 = 7.9 Hz, J_2 = 1.2 Hz, 1H), 7.11 (d, J = 7.4 Hz, 1H), 6.82 (d, J = 7.8 Hz, 1H), 4.39 (d, J = 10.8 Hz, 1H), 4.05 (d, J = 10.8 Hz, 1H), 1.56 (s, 3H) ppm.

^{13}C NMR (101 MHz, $CDCl_3$) δ 197.7, 140.1, 140.0, 136.8 (2C), 134.8, 132.1, 130.7, 129.8, 129.2, 128.7, 128.6, 128.0 (2C), 127.7, 127.4, 125.6, 123.5, 123.3, 59.8, 52.0, 21.0 ppm.**HRMS** (ES+) Calcd for $C_{22}H_{17}ClNaO^+$ $[M+Na]^+$: 355.0860, Found: 355.0868.

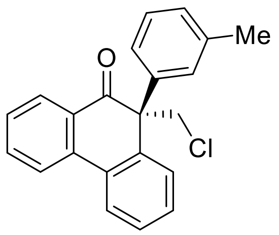

3m

(*R*)-10-(Chloromethyl)-10-(*m*-tolyl)phenanthren-9(10*H*)-one (3m) was prepared as a white solid from **1m** (89.5 mg, 0.3 mmol) according to the General Procedure E (11 h, eluent: *n*-hexane/EtOAc = 30:1, 88.0 mg, 88% yield, 91% ee).

$[\alpha]_D^{25}$: +88.1 (c = 1.0, CH_2Cl_2). HPLC analysis of product: Daicel CHIRALPAK® AS-3 column; 5% *i*-PrOH in *n*-hexane; 1.0 mL/min; retention time: 6.2 min (minor), 7.8 min (major).

^{1}H NMR (400 MHz, $CDCl_3$) δ 8.11 – 8.09 (m, 1H), 8.02 (dd, J_1 = 13.9 Hz, J_2 = 7.9 Hz, 2H), 7.63 (t, J = 7.7 Hz, 1H), 7.50 (dq, J_1 = 7.2 Hz, J_2 = 5.9 Hz, 2H), 7.42 – 7.35 (m, 2H), 7.06 (t, J = 7.7 Hz, 1H), 6.99 (d, J = 7.5 Hz, 1H), 6.88 (s, 1H), 6.79 (d, J = 7.8 Hz, 1H), 5.03 (d, J = 10.9 Hz, 1H), 4.14 (d, J = 10.9 Hz, 1H), 2.21 (s, 3H) ppm.

^{13}C NMR (101 MHz, $CDCl_3$) δ 197.2, 140.0, 138.8, 137.6, 137.3, 135.0, 132.0, 129.6, 129.4, 129.2, 128.9, 128.7 (2C), 128.6, 128.5 128.1, 124.8, 124.7, 123.4, 61.5, 48.1, 21.9 ppm.

HRMS (ES+) Calcd for $C_{22}H_{17}ClNaO^+$ $[M+Na]^+$: 355.0860, Found: 355.0867.

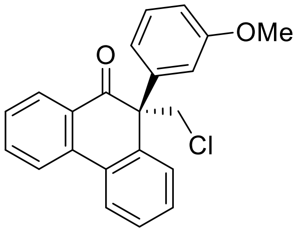

3n

(*R*)-10-(Chloromethyl)-10-(3-methoxyphenyl)phenanthren-9(10*H*)-one (3n) was prepared as a white solid from **1n** (94.3 mg, 0.3 mmol) according to the General Procedure E (11 h, eluent: *n*-hexane/EtOAc = 30:1, 109.1 mg, 99% yield, 94% ee).

$[\alpha]_D^{25}$: +86.7 (c = 1.0, CH_2Cl_2). HPLC analysis of product: Daicel CHIRALPAK® AS-3 column; 15% *i*-PrOH in *n*-hexane; 1.0 mL/min; retention time: 7.1 min (minor), 8.7 min

(major).

^{1}H NMR (400 MHz, $CDCl_3$) δ 8.10 – 8.07 (m, 1H), 8.04 – 7.98 (m, 2H), 7.63 (dd, J_1 = 11.1 Hz, J_2 = 4.2 Hz, 1H), 7.52 – 7.45 (m, 2H), 7.43 – 7.41 (m, 1H), 7.36 (t, J = 7.5 Hz, 1H), 7.11 (t, J = 8.1 Hz, 1H), 6.71 (dd, J_1 = 8.2 Hz, J_2 = 2.3, 1H), 6.64 (d, J = 7.9 Hz, 1H), 6.59 (t, J = 2.0 Hz, 1H), 5.01 (d, J = 10.9 Hz, 1H), 4.15 (d, J = 10.9 Hz, 1H), 3.66 (s, 3H) ppm.

^{13}C NMR (101 MHz, $CDCl_3$) δ 196.7, 159.8, 141.3, 137.2, 137.0, 134.8, 131.7, 129.7, 129.3, 129.1 (2C), 128.4, 128.3 (2C), 124.4, 123.1, 119.7, 114.0, 112.9, 61.3, 55.2, 47.6 ppm.

HRMS (ES+) Calcd for $C_{22}H_{17}ClNaO_2^+$ $[M+Na]^+$: 371.0809, Found: 371.0815.

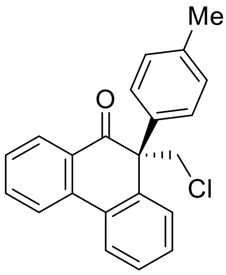

3o

(*R*)-10-(Chloromethyl)-10-(*p*-tolyl)phenanthren-9(10*H*)-one (3o) was prepared as a white solid from **1o** (89.5 mg, 0.3 mmol) according to the General Procedure E (11 h, eluent: *n*-hexane/EtOAc = 30:1, 87.9 mg, 88% yield, 93% ee).

$[\alpha]_D^{25}$: +123.4 (c = 1.0, CH_2Cl_2). HPLC analysis of product: Daicel CHIRALPAK® IB N-3 column; 5% *i*-PrOH in *n*-hexane; 1.0 mL/min; retention time: 9.5 min (major), 10.5 min (minor).

^{1}H NMR (400 MHz, $CDCl_3$) δ 8.11 – 8.08 (m, 1H), 8.04 – 7.98 (m, 2H), 7.62 (td, J_1 = 7.8 Hz, J_2 = 1.5 Hz, 1H), 7.54 – 7.46 (m, 2H), 7.44 – 7.41 (m, 1H), 7.38 – 7.34 (m, 1H), 6.99 (d, J = 8.3 Hz, 2H), 6.92 (d, J = 8.4 Hz, 2H), 5.02 (d, J = 11.0 Hz, 1H), 4.13 (d, J = 10.9 Hz, 1H), 2.22 (s, 3H) ppm.

^{13}C NMR (101 MHz, $CDCl_3$) δ 197.2, 138.3, 137.6, 137.2, 137.0, 135.0 (2C), 132.0, 129.8, 129.5, 129.4, 128.7, 128.6, 128.5, 127.5, 124.7, 123.4, 61.4, 47.8, 21.2 ppm.

HRMS (ES+) Calcd for $C_{22}H_{17}ClNaO^+$ $[M+Na]^+$: 355.0860, Found: 355.0869.

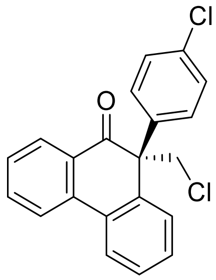

3p

(*R*)-10-(Chloromethyl)-10-(4-chlorophenyl)phenanthren-9(10*H*)-one (3p) was prepared as a white solid from **1p** (95.5 mg, 0.3 mmol) according to the General Procedure E (11 h, eluent: *n*-hexane/EtOAc = 30:1, 99.6 mg, 94% yield, 96% ee).

$[\alpha]_D^{25}$: +98.2 (c = 1.0, CH_2Cl_2). HPLC analysis of product: Daicel CHIRALPAK® IB N-3 column; 5% *i*-PrOH in *n*-hexane; 1.0 mL/min; retention time: 10.1 min (major), 12.1 min (minor).

^{1}H NMR (400 MHz, $CDCl_3$) δ 8.10 (dd, J_1 = 7.7 Hz, J_2 = 1.4 Hz, 1H), 8.03 – 7.99 (m, 2H), 7.67 – 7.63 (m, 1H), 7.50 (dtd, J_1 = 16.6 Hz, J_2 = 7.3 Hz, J_3 = 1.5 Hz, 2H), 7.38 (t, J = 7.9 Hz, 2H), 7.18 – 7.14 (m, 2H), 7.00 – 6.96 (m, 2H), 4.96 (d, J = 11.0 Hz, 1H), 4.11 (d, J = 11.0 Hz, 1H) ppm.

^{13}C NMR (101 MHz, $CDCl_3$) δ 196.6, 138.3, 136.8 (2C), 135.0, 134.2, 131.7, 129.3, 129.2, 128.9, 128.8 (2C), 128.6, 128.5, 128.3, 124.5, 123.2, 60.8, 47.5 ppm.

HRMS (ES+) Calcd for $C_{21}H_{14}Cl_2NaO^+$ $[M+Na]^+$: 375.0314, Found: 375.0319.

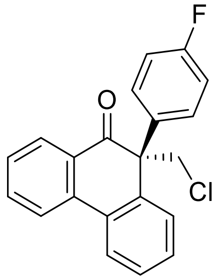

3q

(*R*)-10-(Chloromethyl)-10-(4-fluorophenyl)phenanthren-9(10*H*)-one (3q) was prepared as a white solid from **1q** (90.7 mg, 0.3 mmol) according to the General Procedure E (11 h, eluent: *n*-hexane/EtOAc = 30:1, 85.9 mg, 85% yield, 94% ee).

$[\alpha]_D^{25}$: +89.3 (c = 1.0, CH_2Cl_2). HPLC analysis of product: Daicel CHIRALPAK® IB N-3 column; 5% *i*-PrOH in *n*-hexane; 1.0 mL/min; retention time: 9.9 min (major), 11.2 min (minor).

^{1}H NMR (400 MHz, $CDCl_3$) δ 8.10 (dd, J_1 = 7.7 Hz, J_2 = 1.5 Hz, 1H), 8.04 – 7.99 (m, 2H), 7.67 – 7.62 (m, 1H), 7.55 – 7.47 (m, 2H), 7.41 – 7.36 (m, 2H), 7.04 – 7.01 (m, 2H), 6.90 – 6.85 (m, 2H), 4.99 (d, J = 11.0 Hz, 1H), 4.12 (d, J = 11.0 Hz, 1H) ppm.

^{13}C NMR (101 MHz, $CDCl_3$) δ 196.8, 162.3 (d, J = 249.5 Hz), 136.9 (d, J = 12.6 Hz), 135.4 (d, J = 3.2 Hz), 134.9, 131.7, 129.2 (4C), 128.8, 128.5 (2C), 128.3, 124.6, 123.1, 115.7 (d, J = 21.5 Hz), 60.7, 47.5 ppm.

^{19}F NMR (376 MHz, $CDCl_3$) 113.8 ppm.

HRMS (ES+) Calcd for $C_{21}H_{14}ClFNaO^+$ $[M+Na]^+$: 359.0609, Found: 359.0616.

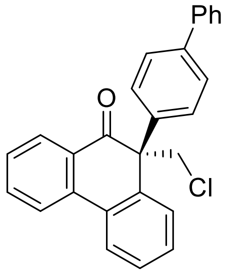

3r

(*R*)-10-([1,1'-Biphenyl]-4-yl)-10-(chloromethyl)phenanthren-9(10*H*)-one (3r) was prepared as a white solid from **1r** (108.1 mg, 0.3 mmol) according to the General Procedure E (11 h, eluent: *n*-hexane/EtOAc = 30:1, 95.1 mg, 80% yield, 96% ee).

$[\alpha]_D^{25}$: +110.5 (c = 1.0, CH_2Cl_2). HPLC analysis of product: Daicel CHIRALPAK® AD-3 column; 5% *i*-PrOH in *n*-hexane; 1.0 mL/min; retention time: 25.7 min (major), 29.7 min (minor).

^{1}H NMR (400 MHz, $CDCl_3$) δ 8.14 – 8.12 (m, 1H), 8.08 – 8.02 (m, 2H), 7.65 (ddd, J_1 = 8.1 Hz, J_2 = 7.3 Hz, J_3 = 1.5 Hz, 1H), 7.56 – 7.49 (m, 2H), 7.48 – 7.45 (m, 3H), 7.43 – 7.36 (m, 5H), 7.33 – 7.29 (m, 1H), 7.14 – 7.10 (m, 2H), 5.08 (d, J = 10.9 Hz, 1H), 4.19 (d, J = 10.9 Hz, 1H) ppm.

^{13}C NMR (101 MHz, $CDCl_3$) δ 196.8, 140.9, 140.1, 138.7, 137.2, 137.0, 134.9, 131.7, 129.3,

129.2, 129.0, 128.8, 128.5, 128.4, 128.3, 127.8, 127.6, 127.5, 127.0, 124.5, 123.2, 61.2, 47.7 ppm.

HRMS (ES+) Calcd for $C_{27}H_{19}ClNaO^+$ $[M+Na]^+$: 417.1017, Found: 417.1022.

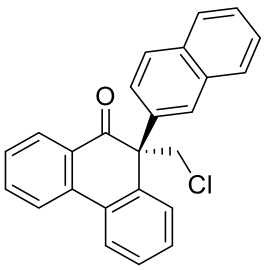

3s

(*R*)-10-(Chloromethyl)-10-(naphthalen-2-yl)phenanthren-9(10*H*)-one (3s) was prepared as a white solid from **1s** (100.3 mg, 0.3 mmol) according to the General Procedure E (11 h, eluent: *n*-hexane/EtOAc = 30:1, 87.2 mg, 79% yield, 97% ee).

$[\alpha]_D^{25}$: +126.5 (*c* = 1.0, CH_2Cl_2). HPLC analysis of product: Daicel CHIRALPAK® AD-3 column; 10% *i*-PrOH in *n*-hexane; 1.0 mL/min; retention time: 20.0 min (major), 22.3 min (minor).

^{1}H NMR (400 MHz, $CDCl_3$) δ 8.15 – 8.13 (m, 1H), 8.06 – 8.01 (m, 2H), 7.73 – 7.61 (m, 4H), 7.57 – 7.49 (m, 3H), 7.47 – 7.40 (m, 3H), 7.38 – 7.34 (m, 1H), 7.15 (dd, J_1 = 8.7 Hz, J_2 = 2.0 Hz, 1H), 5.14 (d, *J* = 10.9 Hz, 1H), 4.24 (d, *J* = 10.9 Hz, 1H) ppm.

^{13}C NMR (101 MHz, $CDCl_3$) δ 196.9, 137.3, 137.2, 137.0, 134.9, 133.1, 132.7, 131.8, 129.4, 129.2, 129.1, 128.6, 128.5, 128.4, 128.3 (2C), 127.4, 126.7, 126.6, 126.4, 124.9, 124.5, 123.2, 61.4, 47.8 ppm.

HRMS (ES+) Calcd for $C_{25}H_{17}ClNaO^+$ $[M+Na]^+$: 391.0860, Found: 391.0872.

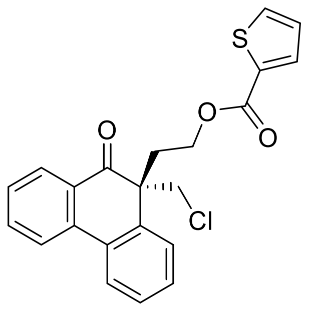

3t

(*R*)-2-(9-(Chloromethyl)-10-oxo-9,10-dihydrophenanthren-9-yl)ethylthiophe--ne-2-carboxylate (3t) was prepared as a white solid from **1t** (108.6 mg, 0.3 mmol) according to the General Procedure E (11 h, eluent: *n*-hexane/EtOAc = 30:1, 122.5 mg, 99% yield, 91% ee).

$[\alpha]_D^{25}$: -77.6 (c = 1.0, CH_2Cl_2). HPLC analysis of product: Daicel CHIRALCEL® OD-3 column; 10% *i*-PrOH in *n*-hexane; 1.0 mL/min; retention time: 18.9 min (major), 21.7 min (minor).

^{1}H NMR (400 MHz, $CDCl_3$) δ 8.14 – 8.04 (m, 3H), 7.67 – 7.63 (m, 1H), 7.50 – 7.41 (m, 4H), 7.39 – 7.33 (m, 2H), 6.96 (dd, J_1 = 4.9 Hz, J_2 = 3.8 Hz, 1H), 4.28 (d, J = 10.5 Hz, 1H), 4.08 (ddd, J_1 = 11.5 Hz, J_2 = 6.5 Hz, J_3 = 5.2 Hz, 1H), 3.93 – 3.87 (m, 2H), 2.81 (ddd, J_1 = 14.5 Hz, J_2 = 8.2 Hz, J_3 = 6.6 Hz, 1H), 2.31 (dt, J_1 = 13.9 Hz, J_2 = 5.6 Hz, 1H) ppm.

^{13}C NMR (101 MHz, $CDCl_3$) δ 198.2, 161.6, 137.1, 137.0, 135.0, 133.4, 133.1, 132.4, 131.2, 129.5, 129.0, 128.2, 128.1, 128.0, 127.5, 126.9, 124.1, 123.2, 61.1, 54.2, 52.4, 38.6 ppm.

HRMS (ES+) Calcd for $C_{22}H_{17}ClNaO_3S^+$ $[M+Na]^+$: 419.0479, Found: 419.0487.

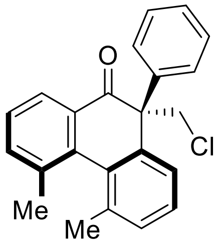

3u

(*R*)-10-(Chloromethyl)-4,5-dimethyl-10-phenylphenanthren-9(10*H*)-one (3u) was prepared as a white solid from **1u** (94.0 mg, 0.3 mmol) according to the General Procedure E (11 h, eluent: *n*-hexane/EtOAc = 30:1, 99.4 mg, 96% yield, 94% ee, 20:1 dr).

$[\alpha]_D^{25}$: +177.9 (c = 1.0, CH_2Cl_2). HPLC analysis of product: Daicel CHIRALPAK® AD-3 column; 10% *i*-PrOH in *n*-hexane; 1.0 mL/min; retention time: 20.0 min (major), 22.3 min (minor).

^{1}H NMR (400 MHz, $CDCl_3$) δ 7.57 – 7.55 (m, 2H), 7.44 (t, J = 7.6 Hz, 1H), 7.37 (dd, J_1 = 7.7 Hz, J_2 = 0.6 Hz, 1H), 7.26 (d, J = 6.9 Hz, 1H), 7.16 (d, J = 9.4 Hz, 1H), 7.11 – 7.07 (m, 3H), 7.01 – 6.99 (m, 2H), 4.63 (d, J = 11.6 Hz, 1H), 4.11 (d, J = 9.7 Hz, 1H), 2.28 (s, 3H), 2.17 (s, 3H) ppm.

^{13}C NMR (101 MHz, $CDCl_3$) δ 197.7, 139.5, 136.5, 136.4, 135.8, 135.6, 133.5, 132.8, 132.7, 130.7, 128.4, 127.8, 127.4, 127.3, 126.7, 125.5, 124.5, 62.3, 46.3, 20.9, 20.0 ppm.

HRMS (ES+) Calcd for $C_{23}H_{19}ClNaO^+$ $[M+Na]^+$: 369.1017, Found: 369.1026.

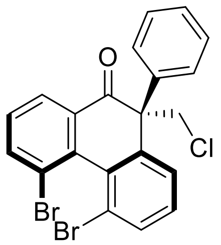

3v

(*R*)-4,5-Dibromo-10-(chloromethyl)-10-phenylphenanthren-9(10*H*)-one (3v) was prepared as a white solid from **1v** (124.4 mg, 0.28 mmol) according to the General Procedure E (11 h, eluent: *n*-hexane/EtOAc = 30:1, 98.2 mg, 74% yield, 97% ee, 20:1 dr). $[\alpha]_D^{25}$: +265.3 (c = 1.0, CH_2Cl_2). HPLC analysis of product: Daicel CHIRALPAK® AD-3 column; 10% *i*-PrOH in *n*-hexane; 1.0 mL/min; retention time: 20.0 min (major), 22.3 min (minor).

^{1}H NMR (400 MHz, $CDCl_3$) δ 7.76 (dd, J_1 = 8.0 Hz, J_2 = 0.8 Hz, 1H), 7.68 (d, J = 7.6 Hz, 1H), 7.61 (t, J = 8.0 Hz, 2H), 7.41 (t, J = 7.9 Hz, 1H), 7.15 – 7.11 (m, 4H), 6.96 (dd, J_1 = 6.5 Hz, J_2 = 3.1 Hz, 2H), 4.60 (d, J = 11.2 Hz, 1H), 4.09 (d, J = 11.7 Hz, 1H) ppm.

^{13}C NMR (101 MHz, $CDCl_3$) δ 194.6, 138.2, 138.1 (2C), 137.1, 135.3, 134.0, 133.9 (2C), 129.6, 129.5, 128.8, 128.3, 126.9, 126.6, 125.6, 123.5, 122.5, 62.8 ppm.

HRMS (ES+) Calcd for $C_{21}H_{13}Br_2ClNaO^+$ $[M+Na]^+$: 496.8914, Found: 496.8918.

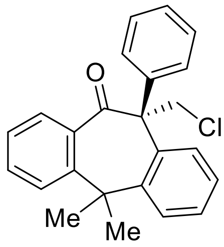

3w

(*R*)-11-(Chloromethyl)-5,5-dimethyl-11-phenyl-5,11-dihydro-10*H*-dibenzo [*a*,*d*][7]annulen-10-one (3w) was prepared as a white solid from **1w** (97.9 mg, 0.3 mmol) according to the General Procedure E, except that the CPA was changed to **D2** and the solvent was changed to chlorobenzene (11 h, eluent: *n*-hexane/EtOAc = 30:1, 89.0 mg, 82% yield, 84% ee).

$[\alpha]_D^{25}$: +178.4 (c = 1.0, CH_2Cl_2). HPLC analysis of product: Daicel CHIRALPAK® AD-3 column; 3% *i*-PrOH in *n*-hexane; 1.0 mL/min; retention time: 7.7 min (minor), 9.1 min (major).

^{1}H NMR (400 MHz, $CDCl_3$) δ 7.66 (d, J = 8.1 Hz, 1H), 7.41 (d, J = 8.0 Hz, 1H), 7.33 – 6.93 (m, 10H), 6.53 (d, J = 7.4 Hz, 1H), 5.46 (d, J = 10.9 Hz, 1H), 4.30 (d, J = 10.9 Hz, 1H), 1.99 (s, 3H), 1.77 (s, 3H) ppm.

^{13}C NMR (101 MHz, $CDCl_3$) δ 204.8, 146.6, 146.1, 142.5, 139.4, 138.7, 132.7, 130.4, 129.8, 128.1 (2C), 127.6 (2C), 126.7, 126.0, 125.2, 123.9, 69.0, 49.2, 42.1, 36.2, 30.0 ppm.

HRMS (ES+) Calcd for $C_{24}H_{21}ClNaO^+$ $[M+Na]^+$: 383.1173, Found: 383.1183.

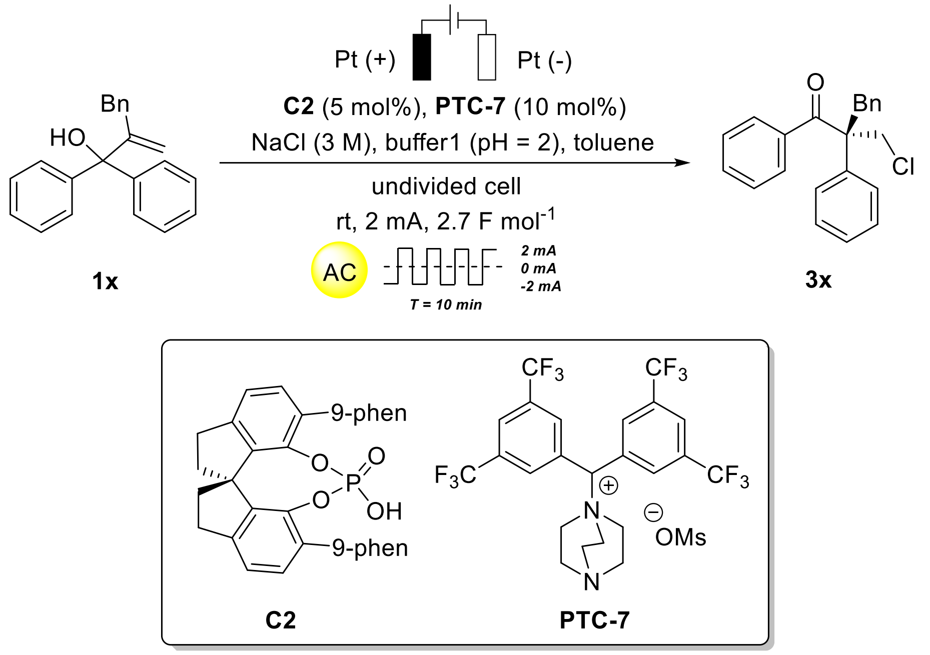

(*R*)-2-Benzyl-3-chloro-1,2-diphenylpropan-1-one (3x) was prepared as a white solid from **1x** (90.4 mg, 0.3 mmol) according to the General Procedure E with **C2** and **PTC-7** as catalysts (11 h, eluent: *n*-hexane/EtOAc = 30:1, 74.1 mg, 80% yield, 58% ee).

$[\alpha]_D^{25}$: -3.6 (c = 1.0, CH_2Cl_2). HPLC analysis of product: Daicel CHIRALPAK® IE-3 column; 5% *i*-PrOH in *n*-hexane; 1.0 mL/min; retention time: 5.9 min (major), 7.0 min (minor).

^{1}H NMR (400 MHz, $CDCl_3$) δ 7.46 – 7.36 (m, 6H), 7.29 – 7.25 (m, 2H), 7.19 – 7.10 (m, 5H), 6.73 – 6.71 (m, 2H), 4.13 (d, *J* = 11.5 Hz, 1H), 3.99 (d, *J* = 11.3 Hz, 1H), 3.76 – 3.48 (m, 2H) ppm.

^{13}C NMR (101 MHz, $CDCl_3$) δ 200.4, 139.7, 136.7, 135.8, 132.1, 130.3, 129.4, 129.1, 128.2, 127.9 (2C), 126.8 (2C), 60.0, 48.6, 39.4 ppm.

HRMS (ES+) Calcd for $C_{21}H_{19}ClNaO^+$ $[M+Na]^+$: 357.1017, Found: 357.1012.

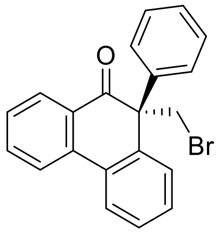

3y

(*R*)-10-(Bromomethyl)-10-phenylphenanthren-9(10H)-one (3y) was prepared as a white solid from **1k** (85.3 mg, 0.3 mmol) according to the General Procedure E except that NaCl was replaced by NaBr (11 h, eluent: *n*-hexane/EtOAc = 30:1, 70.1 mg, 64% yield, 82% ee).

$[\alpha]_D^{25}$: +72.3 (c = 1.0, CH_2Cl_2). HPLC analysis of product: Daicel CHIRALPAK® AS-3 column; 3% *i*-PrOH in *n*-hexane; 1.0 mL/min; retention time: 9.4 min (minor), 12.4 min (major).

^{1}H NMR (400 MHz, $CDCl_3$) δ 8.11 (dd, J_1 = 7.5, J_2 = 1.9 Hz, 1H), 8.05 – 8.00 (m, 2H), 7.64 (ddd, J_1 = 8.0, J_2 = 7.2, J_3 = 1.5 Hz, 1H), 7.51 (pd, J_1 = 7.3, J_2 =1.6 Hz, 2H), 7.41 – 7.35 (m, 2H), 7.20 – 7.17 (m, 3H), 7.08 – 7.04 (m, 2H), 4.91 (d, J = 10.1 Hz, 1H), 3.99 (d, J = 10.1 Hz, 1H) ppm.

^{13}C NMR (101 MHz, $CDCl_3$) δ 196.7, 140.1, 137.9, 136.9, 134.8, 131.5, 129.2, 129.1, 128.9, 128.8, 128.4, 128.3 (2C), 128.0, 127.3, 124.3, 123.1, 60.6, 36.2 ppm.

HRMS (ES+) Calcd for $C_{21}H_{15}BrNaO^+$ $[M+Na]^+$: 385.0198, Found: 385.0203.

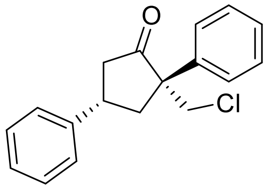

4a

(2*S*,4*S*)-2-(Chloromethyl)-2,4-diphenylcyclopentan-1-one (4a) was prepared as a colorless oil from **2a** (68.1 mg, 0.27 mmol) according to the General Procedure E (11 h, eluent: *n*-hexane/EtOAc = 30:1, 71.2 mg, 93% yield, 91% ee, 10:1 dr).

$[\alpha]_D^{25}$: +0.4 (c = 1.0, CH_2Cl_2). HPLC analysis of product: Daicel CHIRALPAK® ID-3 column; 3% *i*-PrOH in *n*-hexane; 1.0 mL/min; retention time: 7.0 min (major), 8.5 min

(minor).

^{1}H NMR (400 MHz, $CDCl_3$) δ 7.49 – 7.46 (m, 2H), 7.44 – 7.40 (m, 2H), 7.38 – 7.33 (m, 3H), 7.31 – 7.25 (m, 3H), 4.02 (d, *J* = 11.1 Hz, 1H), 3.69 (d, *J* = 11.1 Hz, 1H), 3.36 – 3.27 (m, 1H), 2.97 (ddd, J_1 = 13.3 Hz, J_2 = 5.7 Hz, J_3 = 2.6 Hz, 1H), 2.83 (ddd, J_1 = 19.2 Hz, J_2 = 8.1 Hz, J_3 = 2.6 Hz, 1H), 2.70 (t, *J* = 13.0 Hz, 1H), 2.43 (dd, J_1 = 19.2 Hz, J_2 = 11.7 Hz, 1H) ppm.

^{13}C NMR (101 MHz, $CDCl_3$) δ 214.7, 142.4, 136.4, 129.2, 128.7, 128.2, 126.9, 126.8 (2C), 60.5, 49.5, 45.7, 38.9, 37.4 ppm.

HRMS (CI+) Calcd for $C_{18}H_{17}ClO^+$ (M^+): 284.0962, Found: 284.0964.

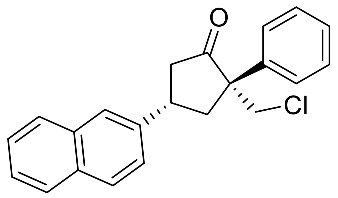

4b

(2*S*,4*S*)-2-(Chloromethyl)-4-(naphthalen-2-yl)-2-phenylcyclopentan-1-one (4b) was prepared as a white solid from **2b** (90.1 mg, 0.3 mmol) according to the General Procedure E (11 h, eluent: *n*-hexane/EtOAc = 30:1, 90.5 mg, 90% yield, 93% ee, 7:1 dr). $[\alpha]_D^{25}$: -28.2 (*c* = 1.0, CH_2Cl_2). HPLC analysis of product: Daicel CHIRALPAK® IC-3 column; 3% *i*-PrOH in *n*-hexane; 1.0 mL/min; retention time: 10.9 min (major), 14.2 min (minor).

^{1}H NMR (400 MHz, $CDCl_3$) δ 7.86 – 7.80 (m, 3H), 7.71 (s 1H), 7.52 – 7.34 (m, 8H), 4.05 (dd, J_1 = 11.1 Hz, J_2 = 1.1 Hz, 1H), 3.72 (dd, J_1 = 11.1 Hz, J_2 = 0.8 Hz, 1H), 3.49 (tdd, J_1 = 12.5 Hz, J_2 = 8.1 Hz, J_3 = 5.7 Hz, 1H), 3.05 (ddd, J_1 = 13.3 Hz, J_2 = 5.7 Hz, J_3 = 2.6 Hz, 1H), 2.91 (ddd, J_1 = 19.2 Hz, J_2 = 8.1 Hz, J_3 = 2.6 Hz, 1H), 2.81 (t, *J* = 12.9 Hz, 1H), 2.54 (dd, J_1 = 19.2 Hz, J_2 = 11.6 Hz, 1H) ppm.

^{13}C NMR (101 MHz, $CDCl_3$) δ 214.7, 139.7, 136.4, 133.4, 132.4, 129.2, 128.5, 128.2, 127.6 (2C), 126.9, 126.3, 125.7, 125.2 (2C), 60.6, 49.5, 45.6, 38.9, 37.6 ppm.

HRMS (ES+) Calcd for $C_{22}H_{19}ClNaO^+$ $[M+Na]^+$: 357.1017, Found: 357.1046.

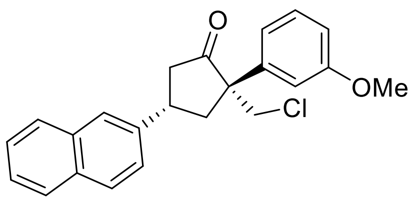

4c

(2*S*,4*S*)-2-(Chloromethyl)-2-(3-methoxyphenyl)-4-(naphthalen-2-yl)cyclopentan-1-one (4c) was prepared as a white solid from **2c** (99.1 mg, 0.3 mmol) according to the General Procedure E (11 h, eluent: *n*-hexane/EtOAc = 30:1, 86.4 mg, 79% yield, 96% ee, 11:1 dr).

$[\alpha]_D^{25}$: -48.0 (c = 1.0, CH_2Cl_2). HPLC analysis of product: Daicel CHIRALCEL® OZ-3 column; 5% *i*-PrOH in *n*-hexane; 1.0 mL/min; retention time: 8.9 min (minor), 10.8 min (major).

^{1}H NMR (400 MHz, $CDCl_3$) δ 7.86 – 7.80 (m, 3H), 7.71 (s, 1H), 7.52 – 7.45 (m, 2H), 7.41 (dd, J_1 = 8.5 Hz, J_2 = 1.8 Hz, 1H), 7.36 (t, J = 8.0 Hz, 1H), 7.10 – 7.08 (m, 1H), 7.06 – 7.05 (m, 1H), 6.92 – 6.89 (m, 1H), 4.05 (d, J = 11.1 Hz, 1H), 3.84 (s, 3H), 3.72 (d, J = 11.1 Hz, 1H), 3.55 – 3.45 (m, 1H), 3.01 (ddd, J_1 = 13.2 Hz, J_2 = 5.7 Hz, J_3 = 2.6 Hz, 1H), 2.91 (ddd, J_1 = 19.1 Hz, J_2 = 8.1 Hz, J_3 = 2.6 Hz 1H), 2.79 (t, J = 13.0 Hz, 1H), 2.53 (dd, J_1 = 19.2 Hz, J_2 = 11.6 Hz, 1H) ppm.

^{13}C NMR (101 MHz, $CDCl_3$) δ 214.5, 160.2, 139.7, 138.0, 133.4, 132.4, 130.2, 128.5, 127.6 (2C), 126.3, 125.7, 125.2 (2C), 119.0, 113.3, 113.1, 60.6, 55.3, 49.4, 45.6, 39.0, 37.6 ppm.

HRMS (ES+) Calcd for $C_{23}H_{21}ClNaO_2^+$ $[M+Na]^+$: 387.1122, Found: 387.1114.

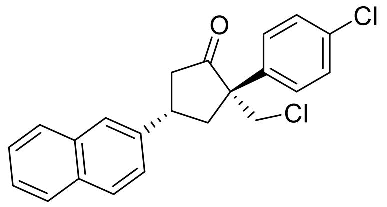

4d

(2*S*,4*S*)-2-(Chloromethyl)-2-(4-chlorophenyl)-4-(naphthalen-2-yl)cyclopentan-1-one (4d) was prepared as a white solid from **2d** (100.5 mg, 0.3 mmol) according to the General Procedure E (11 h, eluent: *n*-hexane/EtOAc = 30:1, 97.7 mg, 88% yield, 92.5%

ee, 8:1 dr).

[α]$_D^{25}$: -79.4 (*c* = 1.0, CH_2Cl_2). HPLC analysis of product: Daicel CHIRALCEL® OZ-3 column; 5% *i*-PrOH in *n*-hexane; 1.0 mL/min; retention time: 8,4 min (minor), 11.1 min (major).

^{1}H NMR (400 MHz, $CDCl_3$) δ 7.86 – 7.80 (m, 3H), 7.70 (s, 1H), 7.52 – 7.39 (m, 7H), 3.99 (d, *J* = 11.2 Hz, 1H), 3.70 (d, *J* = 11.2 Hz, 1H), 3.50 – 3.41 (m, 1H), 3.02 (ddd, J_1 = 13.4 Hz, J_2 = 5.6 Hz, J_3 = 2.5 Hz, 1H), 2.90 (ddd, J_1 = 19.2 Hz, J_2 = 8.1 Hz, J_3 = 2.5 Hz, 1H), 2.77 (t, *J* = 13.0 Hz, 1H), 2.54 (dd, J_1 = 19.2 Hz, J_2 = 11.7 Hz, 1H) ppm.

^{13}C NMR (101 MHz, $CDCl_3$) δ 214.4, 139.4, 134.8, 134.4, 133.4, 132.5, 129.4, 128.6, 128.4, 127.7, 127.6, 126.4, 125.8, 125.2, 125.0, 60.0, 49.6, 45.6, 39.0, 37.6 ppm.

HRMS (ES+) Calcd for $C_{22}H_{18}Cl_2NaO^+$ [M+Na]$^+$: 391.0627, Found: 391.0615.

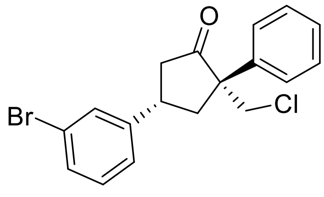

4e

(2*S*,4*S*)-4-(3-Bromophenyl)-2-(chloromethyl)-2-phenylcyclopentan-1-one (4e) was prepared as a colorless oil from **2e** (81.7 mg, 0.25 mmol) according to the General Procedure E (11 h, eluent: *n*-hexane/EtOAc = 30:1, 85.6 mg, 95% yield, 95% ee, 13:1 dr).

[α]$_D^{25}$: -10.5 (*c* = 1.0, CH_2Cl_2). HPLC analysis of product: Daicel CHIRALCEL® OD-3 column; 15% *i*-PrOH in *n*-hexane; 1.0 mL/min; retention time: 9.6 min (major), 11.7 min (minor).

^{1}H NMR (400 MHz, $CDCl_3$) δ 7.47 – 7.17 (m, 10H), 4.01 (d, *J* = 11.2 Hz, 1H), 3.68 (d, *J* = 11.2 Hz, 1H), 3.32 – 3.23 (m, 1H), 2.95 (ddd, J_1 = 13.2 Hz, J_2 = 5.7 Hz, J_3 = 2.6 Hz, 1H), 2.82 (ddd, J_1 = 19.1 Hz, J_2 = 8.1 Hz, J_3 = 2.6 Hz, 1H), 2.66 (t, *J* = 13.0 Hz, 1H), 2.39 (dd, J_1 = 19.1 Hz, J_2 = 11.7 Hz, 1H) ppm.

^{13}C NMR (101 MHz, $CDCl_3$) δ 214.0, 144.8, 136.2, 130.3, 130.1, 130.0, 129.3, 128.3, 126.8, 125.5, 122.8, 60.5, 49.4, 45.4, 38.8, 37.1 ppm.

HRMS (ES+) Calcd for $C_{18}H_{16}BrClNaO^+$ [M+Na]$^+$: 384.9965, Found: 384.9981.

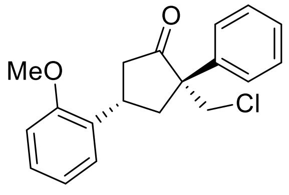

4f

(2*S*,4*S*)-2-(Chloromethyl)-4-(2-methoxyphenyl)-2-phenylcyclopentan-1-one (4f) was prepared as a colorless oil from **2f** (84.1 mg, 0.3 mmol) according to the General Procedure E (11 h, eluent: *n*-hexane/EtOAc = 30:1, 88.4 mg, 94% yield, 86.5% ee, 8:1 dr). $[\alpha]_D^{25}$: -2.0 (*c* = 1.0, CH_2Cl_2). HPLC analysis of product: Daicel CHIRALCEL® IB N-3 column; 1% *i*-PrOH in *n*-hexane; 1.0 mL/min; retention time: 12.3 min (major), 16.4 min (minor).

^{1}H NMR (400 MHz, $CDCl_3$) δ 7.57 – 7.48 (m, 2H), 7.43 – 7.40 (m, 2H), 7.37 – 7.30 (m, 1H), 7.28 – 7.24 (m, 2H), 6.97 (t, *J* = 7.4, 1H), 6.89 (d, *J* = 8.2 Hz, 1H), 4.01 (d, *J* = 11.2 Hz, 1H), 3.82 (s, 3H), 3.71 (d, *J* = 11.1 Hz, 1H), 3.61 – 3.52 (m, 1H), 2.95 (ddd, J_1 = 13.2 Hz, J_2 = 5.7 Hz, J_3 = 2.5 Hz, 1H), 2.83 – 2.70 (m, 2H), 2.45 (dd, J_1 = 19.2 Hz, J_2 = 11.5 Hz, 1H) ppm.

^{13}C NMR (101 MHz, $CDCl_3$) δ 215.4, 157.6, 136.5, 130.3, 129.1, 128.0, 127.9, 127.0, 126.7, 120.6, 110.5, 60.1, 55.2, 50.0, 43.8, 36.5, 32.6 ppm.

HRMS (ES+) Calcd for $C_{19}H_{19}ClNaO_2^+$ $[M+Na]^+$: 337.0966, Found: 337.0971.

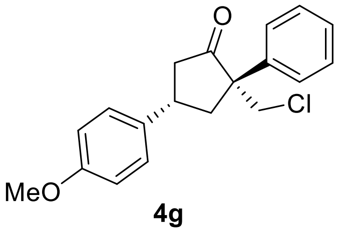

4g

(2*S*,4*S*)-2-(Chloromethyl)-4-(4-methoxyphenyl)-2-phenylcyclopentan-1-one (4g) was prepared as a colorless oil from **2g** (84.1 mg, 0.3 mmol) according to the General Procedure E (11 h, eluent: *n*-hexane/EtOAc = 30:1, 80.1 mg, 85% yield, 93% ee, 8:1 dr). $[\alpha]_D^{25}$: -9.2 (*c* = 1.0, CH_2Cl_2). HPLC analysis of product: Daicel CHIRALCEL® OX-3 column; 10% *i*-PrOH in *n*-hexane; 1.0 mL/min; retention time: 12.0 min (minor), 16.7

min (major).

^{1}H NMR (400 MHz, $CDCl_3$) δ 7.48 – 7.33 (m, 5H), 7.22 – 7.16 (m, 2H), 6.91 – 6.87 (m, 2H), 4.00 (d, *J* = 11.2 Hz, 1H), 3.81 (s, 3H), 3.68 (d, *J* = 11.1 Hz, 1H), 3.26 (tdd, J_1 = 12.6 Hz, J_2 = 8.1 Hz, J_3 = 5.7 Hz, 1H), 2.93 (ddd, J_1 = 13.3 Hz, J_2 = 5.7 Hz, J_3 = 2.6 Hz, 1H), 2.80 (ddd, J_1 = 19.1 Hz, J_2 = 8.1 Hz, J_3 = 2.6 Hz, 1H), 2.64 (t, *J* = 13.0 Hz, 1H), 2.37 (dd, J_1 = 19.2 Hz, J_2 = 11.7 Hz, 1H) ppm.

^{13}C NMR (101 MHz, $CDCl_3$) δ 215.0, 158.5, 136.4, 134.4, 129.2, 128.2, 127.8, 127.7, 126.9, 126.6, 114.1, 60.5, 55.3, 49.6, 46.0, 39.2, 36.6 ppm.

HRMS (ES+) Calcd for $C_{19}H_{19}ClNaO_2^+$ $[M+Na]^+$: 337.0966, Found: 337.0938.

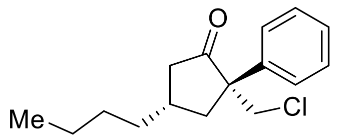

4h

(2*S*,4*S*)-4-Butyl-2-(chloromethyl)-2-phenylcyclopentan-1-one (4h) was prepared as a colorless oil from **2h** (64.5 mg, 0.28 mmol) according to the General Procedure E (11 h, eluent: *n*-hexane/EtOAc = 30:1, 69.1 mg, 93% yield, 82% ee, 5:1 dr).

$[\alpha]_D^{25}$: +13.8 (*c* = 1.0, CH_2Cl_2). HPLC analysis of major diastereomer product: Daicel CHIRALCEL® OX-3 column; 1% *i*-PrOH in *n*-hexane; 1.0 mL/min; retention time: 7.8 min (minor), 9.7 min (major).

^{1}H NMR (400 MHz, $CDCl_3$) major diastereomer δ 7.46 – 7.27 (m, 5H), 3.91 (d, *J* = 11.1 Hz, 1H), 3.63 (d, *J* = 11.1 Hz, 1H), 2.74 – 2.69 (m, 1H), 2.56 – 2.49 (m, 1H), 2.12 – 2.03 (m, 2H), 1.88 (dd, J_1 = 19.0 Hz, J_2 = 10.5 Hz, 1H), 1.56 – 1.41 (m, 2H), 1.38 – 1.25 (m, 4H), 0.90 (t, *J* = 6.9 Hz, 3H) ppm.

^{13}C NMR (101 MHz, $CDCl_3$) major diastereomer δ 215.9, 136.8, 129.0, 127.9, 126.8, 60.1, 49.8, 44.7, 38.1, 35.4, 32.2, 30.0, 22.8, 14.0 ppm.

HRMS (ES+) Calcd for $C_{16}H_{21}ClONa^+$ $[M+Na]^+$: 287.1173, Found: 287.1180.

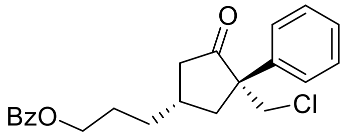

4i

3-((*1S,3S*)-3-(Chloromethyl)-4-oxo-3-phenylcyclopentyl)propyl benzoate (4i) was prepared as a colorless oil from **2i** (100.9 mg, 0.3 mmol) according to the General Procedure E (11 h, eluent: *n*-hexane/EtOAc = 30:1, 83.6 mg, 75% yield, 82% ee, 4:1 dr). $[\alpha]_D^{25}$: +8.6 (*c* = 1.0, CH_2Cl_2). HPLC analysis of major diastereomer product: Daicel CHIRALDAL® IE-3 column; 10% *i*-PrOH in *n*-hexane; 1.0 mL/min; retention time: 16.7 min (major), 21.4 min (minor).

^{1}H NMR (400 MHz, $CDCl_3$) major diastereomer δ 8.04 – 8.01 (m, 2H), 7.58 – 7.54 (m, 1H), 7.46 – 7.26 (m, 7H), 4.33 (td, J_1 = 6.5 Hz, J_2 = 1.9 Hz, 2H), 3.92 (d, *J* = 11.1 Hz, 1H), 3.63 (d, *J* = 11.1 Hz, 1H), 2.81 – 2.72 (m, 1H), 2.61 – 2.54 (m, 1H), 2.18 – 2.08 (m, 2H), 1.96 – 1.56 (m, 5H) ppm.

^{13}C NMR (101 MHz, $CDCl_3$) major diastereomer δ 215.3, 166.5, 136.5, 132.9, 130.2, 129.5, 129.1, 128.3, 128.0, 126.8, 64.7, 60.2, 49.6, 44.5, 37.9, 32.1, 32.0, 27.1 ppm.

HRMS (ES+) Calcd for $C_{22}H_{23}ClNaO_3^+$ $[M+Na]^+$: 393.1228, Found: 393.1234.

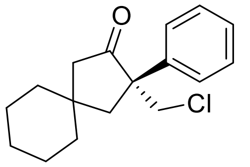

4j

(*S*)-3-(Chloromethyl)-3-phenylspiro[4.5]decan-2-one (4j) was prepared as a colorless oil from **2j** (72.7 mg, 0.3 mmol) according to the General Procedure E (11 h, eluent: *n*-hexane/EtOAc = 30:1, 58.8 mg, 71% yield, 78% ee).

$[\alpha]_D^{25}$: +15.7 (*c* = 1.0, CH_2Cl_2). HPLC analysis of product: Daicel CHIRALPAK® IC-3 column; 3% *i*-PrOH in *n*-hexane; 1.0 mL/min; retention time: 6.3 min (major), 7.2 min (minor).

^{1}H NMR (400 MHz, $CDCl_3$) δ 7.44 – 7.42 (m, 2H), 7.37 – 7.31 (m, 2H), 7.27 (dt, J_1 = 7.2

Hz, J_2 = 2.9 Hz, 1H), 3.81 (d, J = 10.9 Hz, 1H), 3.53 (d, J = 10.9 Hz, 1H), 2.68 (dd, J_1 = 13.9 Hz, J_2 = 1.5 Hz, 1H), 2.46 – 2.41 (m, 2H), 2.16 (d, J = 18.7 Hz, 1H), 1.61 – 1.51 (m, 3H), 1.43 – 1.28 (m, 4H), 1.27 – 1.17 (m, 1H), 1.12 – 1.09 (m, 2H) ppm.

^{13}C NMR (101 MHz, $CDCl_3$) δ 217.2, 139.7, 128.8, 127.6, 126.6, 58.5, 51.2, 39.8, 37.7, 36.9, 25.7, 23.3, 22.5 ppm.

HRMS (CI+) Calcd for $C_{17}H_{21}ClO^+$ (M^+): 276.1275, Found: 276.1280.

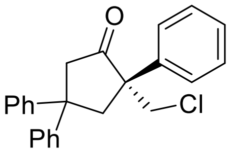

4k

(*S*)-2-(Chloromethyl)-2,4,4-triphenylcyclopentan-1-one (4k) was prepared as a colorless oil from **2k** (98.0 mg, 0.3 mmol) according to the General Procedure E (11 h, eluent: *n*-hexane/EtOAc = 30:1, 103.8 mg, 96% yield, 78% ee).

$[\alpha]_D^{25}$: -57.6 (c = 1.0, CH_2Cl_2). HPLC analysis of product: Daicel CHIRALPAK® IB N-3 column; 5% *i*-PrOH in *n*-hexane; 1.0 mL/min; retention time: 11.3 min (major), 12.7 min (minor).

^{1}H NMR (400 MHz, $CDCl_3$) δ 7.39 – 7.31 (m, 4H), 7.27 – 7.20 (m, 3H), 7.14 – 6.93 (m, 8H), 3.61 – 3.51 (m, 3H), 3.42 (dd, J_1 = 15.8 Hz, J_2 = 2.2 Hz, 2H), 3.11 (dd, J_1 = 18.2 Hz, J_2 = 0.9 Hz, 1H) ppm.

^{13}C NMR (101 MHz, $CDCl_3$) δ 214.9, 147.0, 145.9, 138.7, 128.7, 128.2, 128.1, 127.0, 126.8, 126.6, 126.5, 126.4, 126.1, 57.7, 52.0, 51.2, 48.6, 43.4 ppm.

HRMS (ES+) Calcd for $C_{24}H_{21}ClNaO^+$ $[M+Na]^+$: 383.1173, Found: 383.1179.

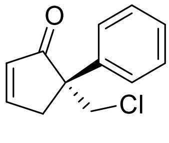

4l

(*S*)-5-(Chloromethyl)-5-phenylcyclopent-2-en-1-one (4l) was prepared as a colorless

oil from **2l** (82.3 mg, 0.3 mmol) according to the General Procedure F (11 h, eluent: *n*-hexane/EtOAc = 30:1, 57.6 mg, 93% yield, 90% ee).

$[\alpha]_D^{25}$: +30.1 (*c* = 1.0, CH_2Cl_2). HPLC analysis of product: Daicel CHIRALPAK® ID-3 column; 5% *i*-PrOH in *n*-hexane; 1.0 mL/min; retention time: 13.5 min (major), 14.7 min (minor).

^{1}H NMR (400 MHz, $CDCl_3$) δ 7.82 (dt, J_1 = 5.7 Hz, J_2 = 2.7 Hz, 1H), 7.38 – 7.32 (m, 4H), 7.31 – 7.25 (m, 1H), 6.31 (dt, J_1 = 5.8 Hz, J_2 = 2.2 Hz, 1H), 3.99 (d, J = 10.8 Hz, 1H), 3.87 (d, J = 10.8 Hz, 1H), 3.37 (dt, J_1 = 19.3 Hz, J_2 = 2.5 Hz, 1H), 3.16 (ddd, J_1 = 19.3 Hz, J_2 = 2.8 Hz, J_3 = 2.1 Hz, 1H) ppm.

^{13}C NMR (101 MHz, $CDCl_3$) δ 207.7, 163.5, 139.4, 133.6, 128.8, 127.6, 126.6, 55.3, 48.9, 41.8 ppm.

HRMS (CI+) Calcd for $C_{12}H_{12}ClO^+$ $[M+H]^+$: 207.0571, Found: 207.0571.

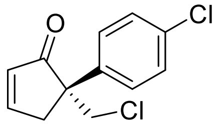

4m

(*S*)-5-(Chloromethyl)-5-(4-chlorophenyl)cyclopent-2-en-1-one (4m) was prepared as a colorless oil from **2m** (68.9 mg, 0.22 mmol) according to the General Procedure F (11 h, eluent: *n*-hexane/EtOAc = 30:1, 34.1 mg, 64% yield, 87% ee).

$[\alpha]_D^{25}$: +11.7 (*c* = 1.0, CH_2Cl_2). HPLC analysis of product: Daicel CHIRALPAK® IB N-3 column; 5% *i*-PrOH in *n*-hexane; 1.0 mL/min; retention time: 10.6 min (major), 11.4 min (minor).

^{1}H NMR (400 MHz, $CDCl_3$) δ 7.83 (dt, J_1 = 5.7 Hz, J_2 = 2.7 Hz, 1H), 7.31 (s, 4H), 6.30 (dt, J_1 = 5.8 Hz, J_2 = 2.1 Hz, 1H), 3.92 (d, J = 10.9 Hz, 1H), 3.82 (d, J = 10.9 Hz, 1H), 3.35 (dt, J_1 = 19.4 Hz, J_2 = 2.5 Hz, 1H), 3.15 – 3.09 (m, 1H) ppm.

^{13}C NMR (101 MHz, $CDCl_3$) δ 207.2, 163.5, 137.7, 133.6, 133.4, 128.9, 128.1, 54.8, 48.9, 41.7 ppm.

HRMS (CI+) Calcd for $C_{12}H_{10}Cl_2O^+$ (M^+): 240.0103, Found: 240.0109.

Other examples:

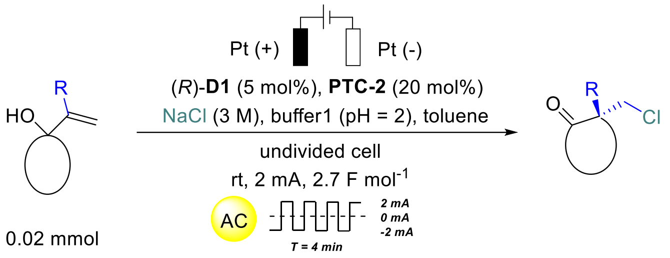

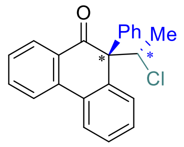

81%, 20% ee, 2:1 dr
from *trans*- substrate

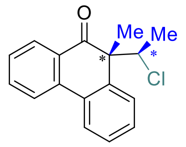

75%, 30% ee, 5:1 dr
from *cis*- substrate

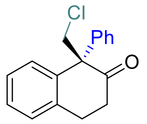

71%, 53% ee

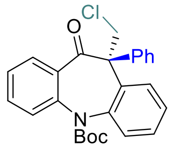

95%, 50% ee
D2 as catalyst

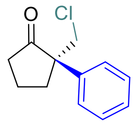

99%, 38% ee

VII. Mechanistic Studies

(1) Investigation on the role of PTC

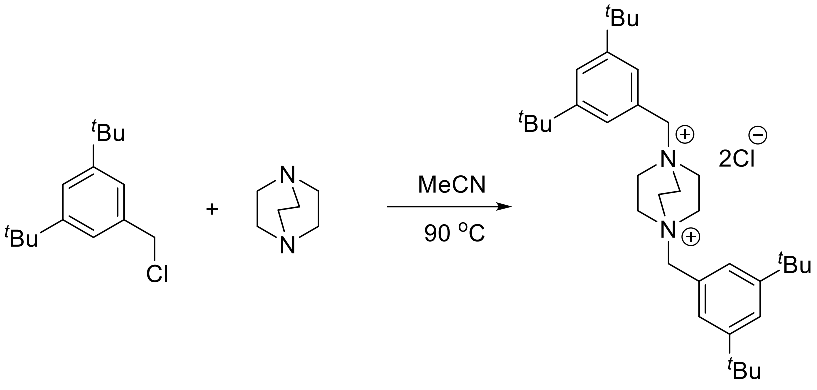

PTC-2'

1,4-Bis(3,5-di-*tert*-butylbenzyl)-1,4-diazabicyclo[2.2.2]octane-1,4-diium dichloride (PTC-2′). A solution of 1,3-di-*tert*-butyl-5-(chloromethyl)benzene (238.8 mg, 1.0 mmol, 5.0 equiv) and DABCO (22.4 mg, 0.2 mmol, 1.0 equiv) in MeCN (4 mL) was heated to 90 °C for 48 h. The resultant mixture was filtered and repeatedly washed with Et_2O to give the pure product as a white solid (54.4 mg, 46% yield).

^{1}H NMR (400 MHz, DMSO-*d*$_6$) δ 7.60 (s, 2H), 6.36 (d, *J* = 1.4 Hz, 4H), 4.79 (s, 4H), 3.88 (s, 12H), 1.34 (s, 36H) ppm.

^{13}C NMR (101 MHz, DMSO-*d*$_6$) δ 151.2, 127.1, 125.7, 124.4, 67.1, 50.2, 34.6, 31.1 ppm.

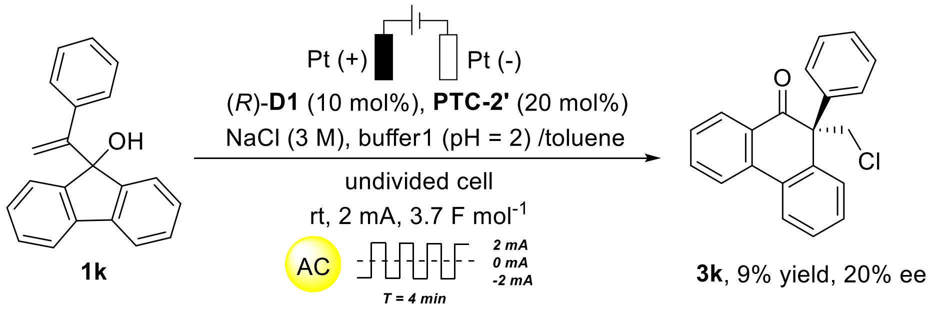

1k **3k**, 9% yield, 20% ee

To a 10-mL tube equipped with a platinum anode, a platinum cathode, and a magnetic stir bars were added the substrate **1k** (0.02 mmol), (*R*)-**D1** (1.5 mg, 0.002 mmol, 10 mol%), **PTC-2′** (1.4 mg, 0.004 mmol, 20 mol%), NaCl (3M aq.), toluene (1.5 mL) and H_3PO_4–NaH_2PO_4 buffer1 ($c(H_3PO_4)$ = $c(NaH_2PO_4)$ = 0.5 M, pH = 2, 2 mL). The

electrolysis was carried out with an alternating current of 2 mA which alternates its direction every 2 min and the total electrolysis lasted for 1 h. The yield was determined to be 9% by ^{1}H NMR with CH_2Br_2 as the internal standard, and the ee value was determined to be 20% by chiral HPLC.

(2) Investigation of the residue on the anode

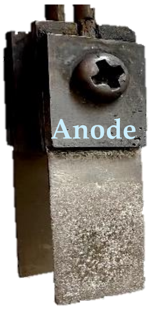

Figure S2. The white residue on the anode.

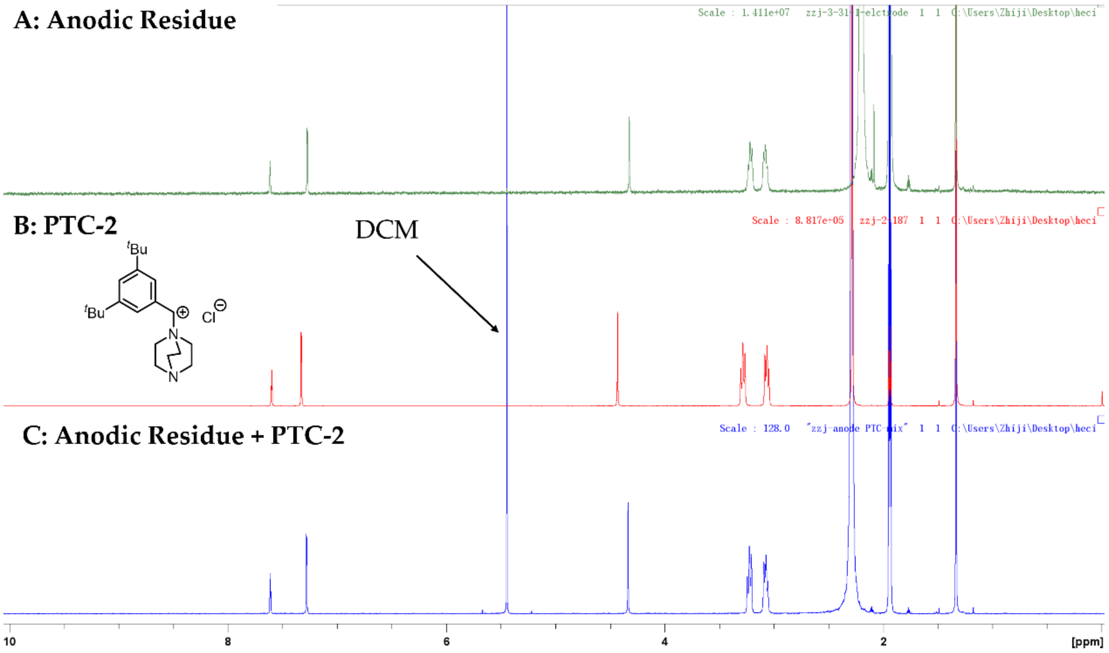

Figure S3. NMR study to investigate the structure of anodic residue under direct current electrolysis. Solvent: MeCN-d_3.

The ^{1}H NMR spectra in Figure S3 showed that the anodic residue had a tiny difference

from **PTC-2** (Figure S3A–B). However, a mixed sample of this residue and **PTC-2** showed that they have the same set of NMR signals (Figure S3C), indicating the anodic residue should have the same ammonium structure. The anion motif might be different, causing minor shifts of cation signals. Another possibility is that these two structures have fast equilibrium between each other. Currently we think it is likely the former situation.

(3) Evidence for the generation of Cl_2

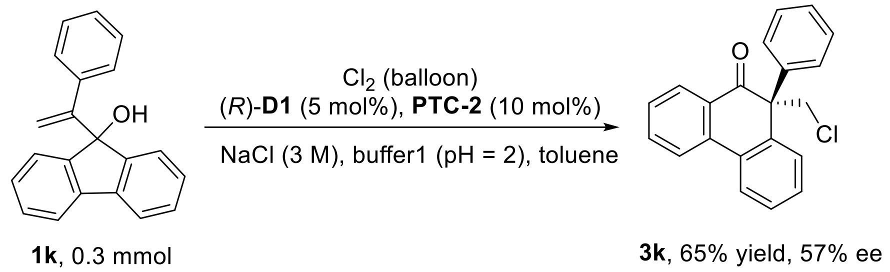

To a 50-mL flask equipped with a rubber cap was added $KMnO_4$ (3 g). A solution of HCl (2 M, 20 mL) was slowly added via a syringe. A syringe needle was inserted into the cap to release the pressure in the flask. When the flask was filled with chlorine gas (detected by a starch-KI test paper), a balloon was attached to the needle to collect the chlorine gas. The balloon was then attached to a 20-mL vial charged with **1k** (0.3 mmol), (*R*)-**D1** (11.5 mg, 0.015 mmol, 5 mol%), **PTC-2** (10.6 mg, 0.03 mmol, 10 mol%), NaCl (3 M aq.), toluene (7.5 mL) and the H_3PO_4–NaH_2PO_4 buffer1 ($c(H_3PO_4)$ = $c(NaH_2PO_4)$ = 0.5 M, pH = 2, 10 mL). The reaction was stopped until **1k** disappeared based on TLC analysis. A solution of aqueous NaOH (2 M) was then added to quench the reaction. The organic layer of the reaction mixture was separated, and the aqueous layer was extracted with EtOAc (10 mL x 3). The combined organic layers were dried over anhydrous Na_2SO_4, filtered, and concentrated. The residue was purified by silica gel chromatography (eluent: *n*-hexane/EtOAc = 10:1) to yield the desired product **3k** (62 mg, 65% yield, 57% ee).

Table S8. Asymmetric Chemical Chlorinative Semipinacol Rearrangement Using *in-situ* Prepared Chlorine Gas

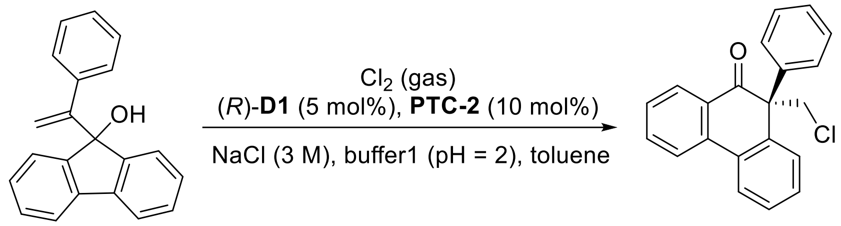

1k, 0.3 mmol **3k**

entry	reaction time	yield, ee
1	30 min	78%, 71%
2	4 h	98%, 95%

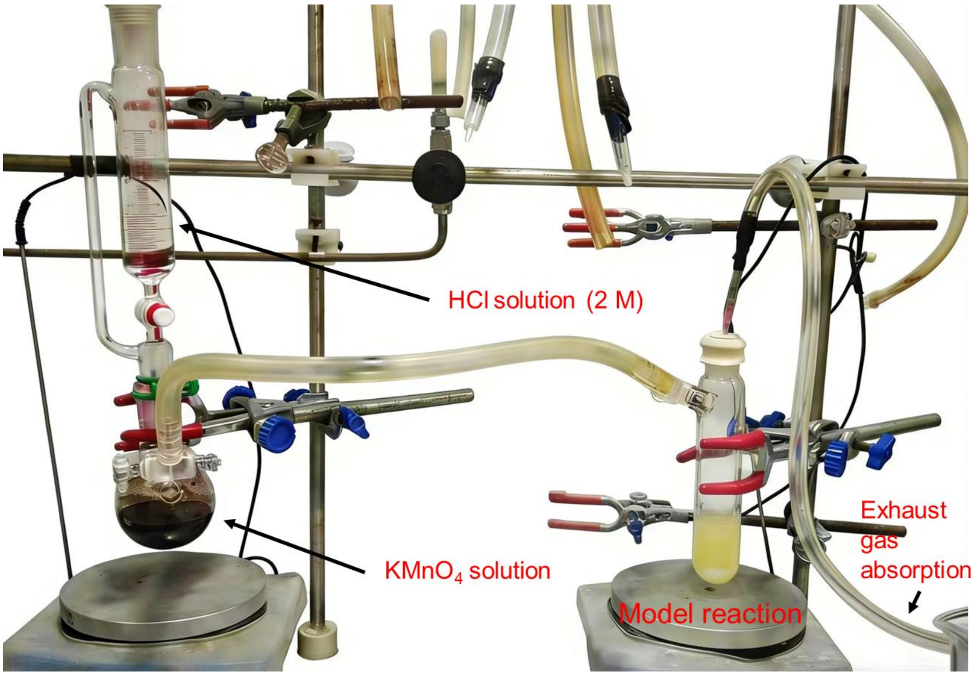

Figure S4. Reaction setup for asymmetric chemical chlorinative semipinacol rearrangement using *in-situ* prepared chlorine gas.

To a 50-mL Schlenk tube were added the substrate **1k** (0.3 mmol), (*R*)-**D1** (11.5 mg, 0.015 mmol, 5 mol%), **PTC-2** (10.6 mg, 0.03 mmol, 10 mol%), NaCl (3 M aq.), toluene (7.5 mL) and the H_3PO_4–NaH_2PO_4 buffer1 ($c(H_3PO_4) = c(NaH_2PO_4) = 0.5$ M, pH = 2, 10 mL). A solution of aqueous HCl (2 M, 20 mL) was added slowly to a $KMnO_4$ solution

(3 g in 10 mL buffer1) by a constant pressure dropping funnel within certain time. The *in-situ* generated chlorine gas was introduced into the Schlenk tube within a rubber tube. The reaction was stopped until **1k** disappeared based on TLC analysis. A solution of aqueous NaOH (2 M) was then added to quench the reaction. The organic layer of the reaction mixture was separated, and the aqueous layer was extracted with EtOAc (10 mL x 3). The combined organic layers were dried over anhydrous Na_2SO_4, filtered, and concentrated. The residue was purified by silica gel chromatography (eluent: *n*-hexane/EtOAc = 10:1) to yield the desired product **3k** (74 mg & 94 mg for the 30-min- & 4-h reactions, respectively).

(4) Reaction progress over time with and without (*R*)-D1

Two parallel reactions were performed in the presence and absence of CPA (*R*)-**D1** to investigate the possible acceleration by this catalyst. The reactions were run according to the General Procedure E. At different stages of the reaction, aliquots of the organic phase were taken from the reaction mixture and quenched by a saturated $Na_2S_2O_3$ solution. The organic phase was separated, and the aqueous layer was extracted with EtOAc (2 mL x 3). The combined organic layers were dried over anhydrous Na_2SO_4, filtered, and concentrated. The residue was subjected to ^{1}H NMR to determine the yield of the product **3k**.

(a) Without **(*R*)-D1**

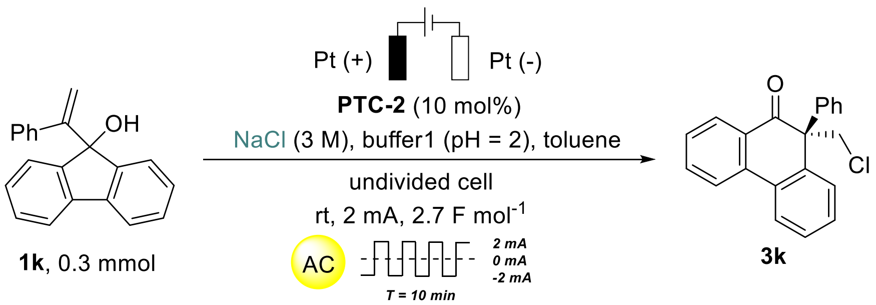

entry	time	yield[a]
1	0.5 h	2%

2	1 h	5%
3	2 h	11%
4	3.5 h	24%
5	5 h	36%
6	8 h	74%
7	11 h	77%

[a]The yields were determined by ^{1}H NMR analysis of the crude reaction mixture.

(b) With **(*R*)-D1**

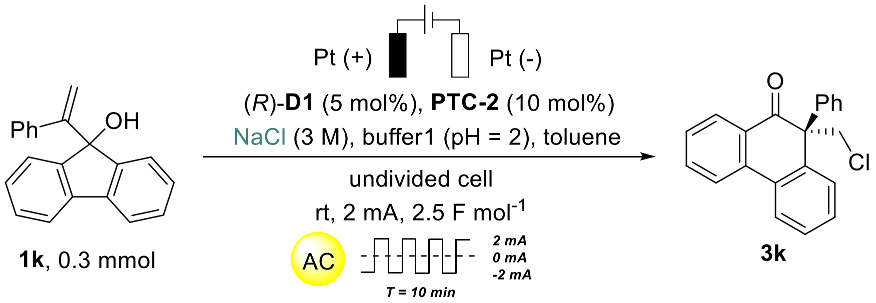

entry	time	yield[a]
1	0.5 h	2%
2	1 h	7%
3	2 h	14%
4	3.5 h	29%
5	5 h	46%
6	7.5 h	78%
7	9 h	95%
8	10 h	99%

[a]The yields were determined by ^{1}H NMR analysis of the crude reaction mixture by ratio of the substrate and product.

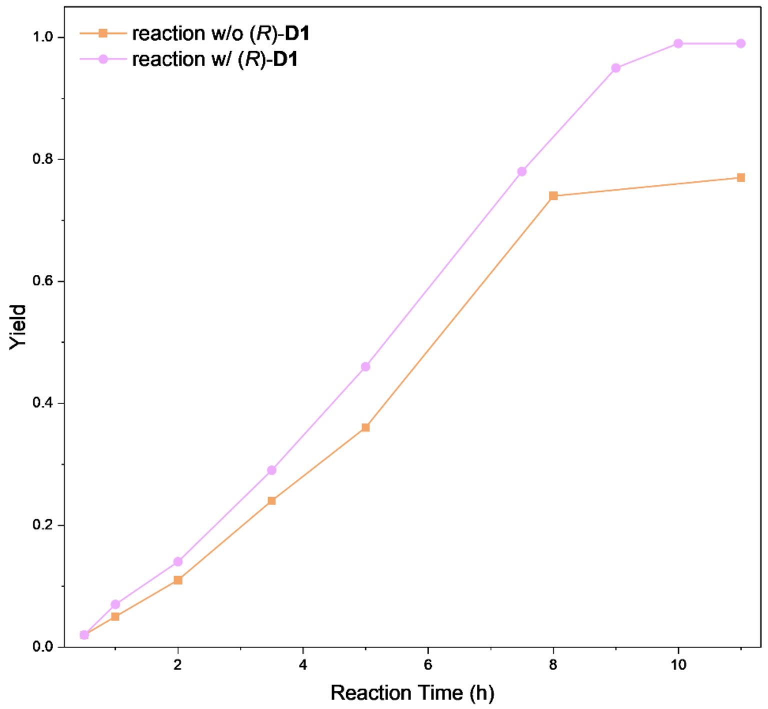

Figure S5. The reaction progress over time with and without (*R*)-**D1**.

(5) Monitoring the pH value and product ee during the reaction progress

A standard reaction of **1k** was performed according to General Procedure E. At different conversions, the pH value of the aqueous phase was recorded, and an aliquot of the organic phase was taken from the reaction system and quenched by a saturated $Na_2S_2O_3$ solution. The organic phase was separated, and the aqueous layer was extracted with EtOAc. The combined organic layers were dried over anhydrous Na_2SO_4, filtered, and concentrated. The residue was subjected to ^1H NMR analysis to determine the conversion and yield of the product **3k**. The ee value was determined by chiral HPLC.

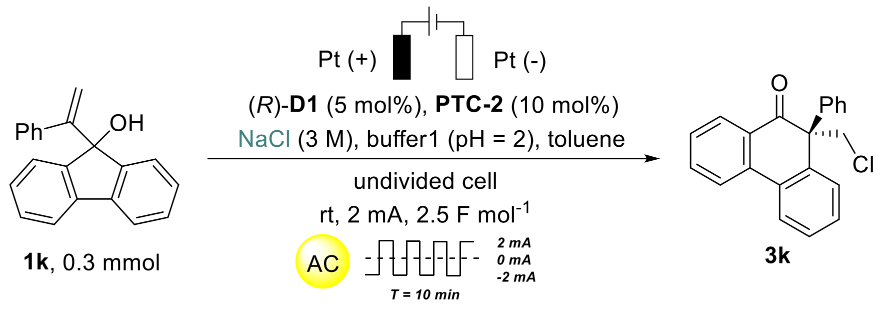

entry	conversion[a]	pH	ee
1	0	1.1	-
2	19%	1.1	95%
3	47%	1.2	96%
4	88%	1.3	95%
5	100%	1.2	96%

[a]Determined by ^{1}H NMR analysis of the crude reaction mixture.

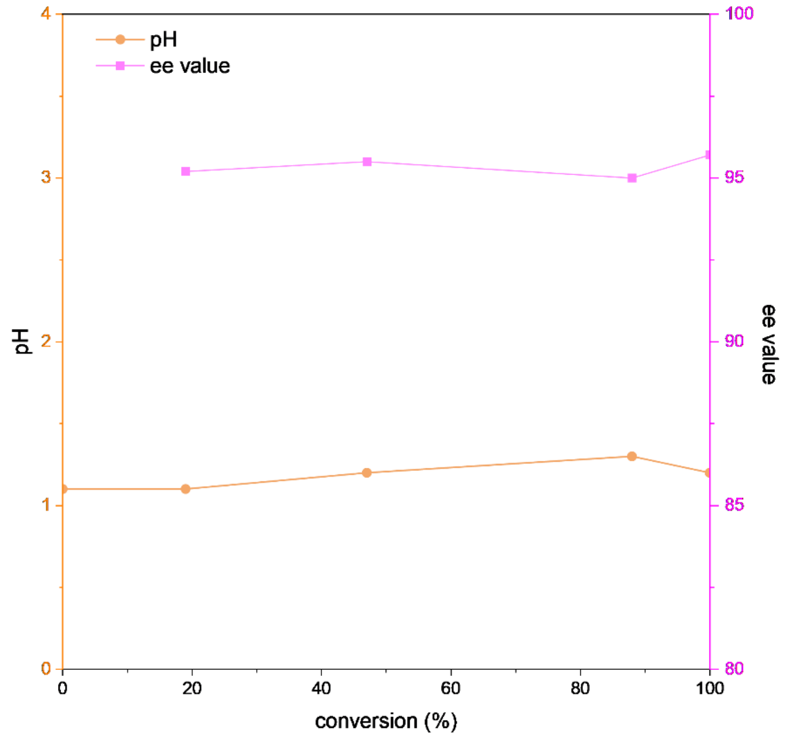

Figure S6. Plots of pH and product ee values at different conversions.

(6) Cyclic Voltammetry

The cyclic voltammetry measurements were carried out using BioLogic VMP3 Multichannel Potentiostat. For all the experiments, a glassy-carbon (GC) electrode (3-mm-diameter, disc-electrode) was used as the working electrode, a platinum wire was used as the counter electrode and an SCE reference electrode was employed. The measurements were recorded at a scan rate of 50 mV/s unless otherwise noted.

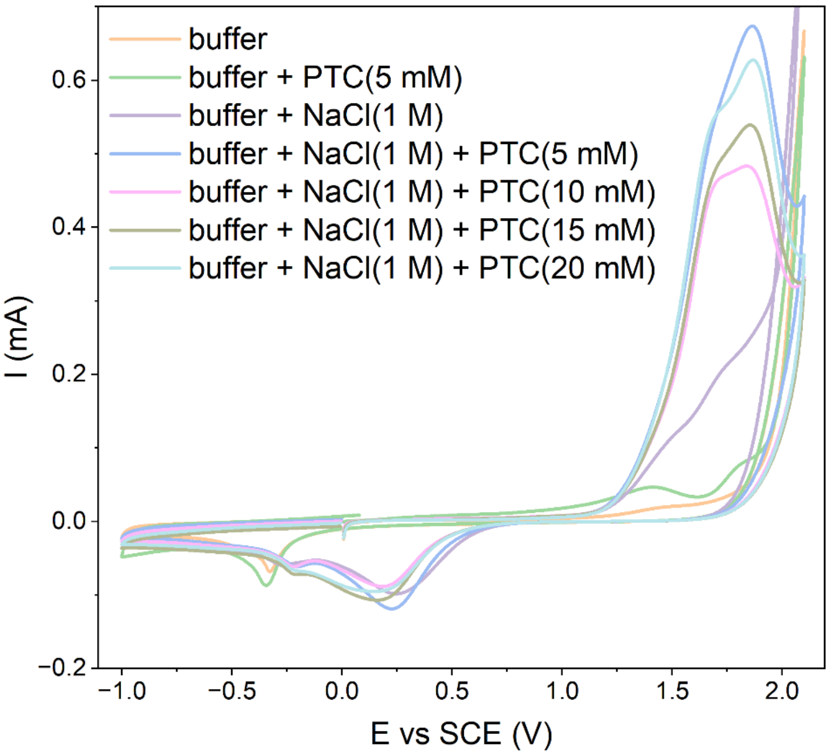

Figure S7. Cyclic voltammetry measurements in buffer with 1 M NaCl and different concentration of **PTC-2**.

For an anodic process, the oxidative current I_P is controlled by both absorption and diffusion. If this process is primarily diffusion control, then $I_P \propto v^{1/2}$ (v is scan rate). If this process is primarily absorption control, then $I_P \propto v$. As shown in Figure S8, we recorded five CV curves with various scan rates. The oxidative current I_P was read from the diagram and a linear fitting between oxidative current I_P and scan rates v was conducted (Figure S9). The results showed that the I_P has a good linear relationship with scan rate v. Thus, the deposition of species onto the electrode surface likely dictates the electrochemical behavior of this system.

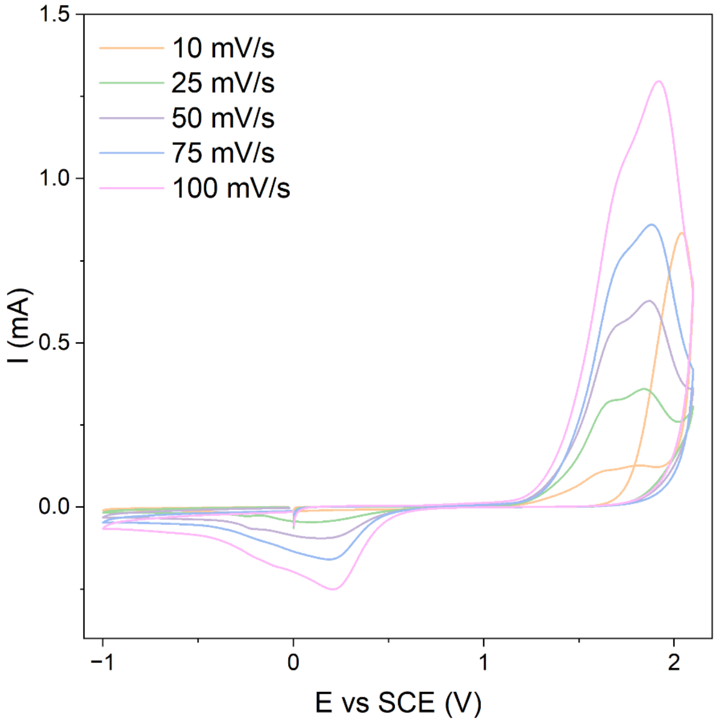

Figure S8. Cyclic voltammetry measurements in buffer with 1 M NaCl and 20 mM **PTC-2** under different scan rates.

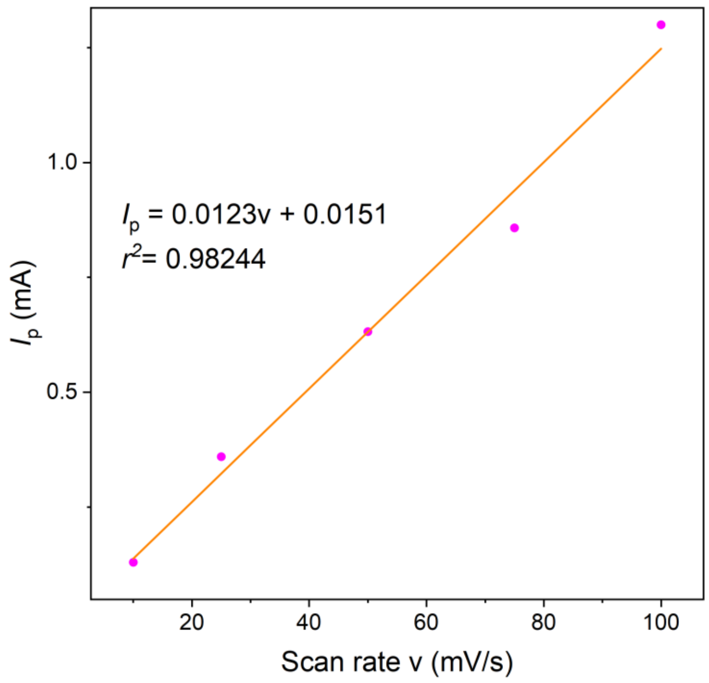

Figure S9. Linear fitting between oxidative peak I_p and scan rate v.

VIII. Large-Scale Synthesis and Product Derivatizations

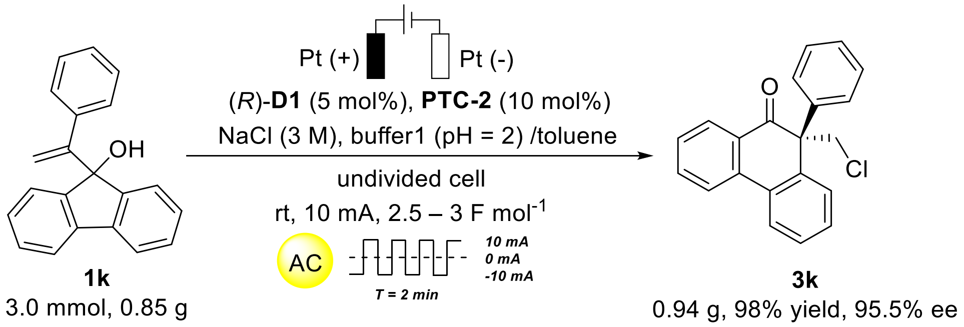

To an undivided bottle (cylindrical, 200 mL) equipped with a screw cap, platinum anode (30 × 40 × 0.2 mm), platinum cathode (30 × 40 × 0.2 mm), and a magnetic stir bar (cylindrical, Ø10 x 30 mm) were added the substrate **1k** (0.85 g, 3.0 mmol, 1.0 equiv), (*R*)-**D1** (115 mg, 0.15 mmol, 5 mol%), **PTC-2** (105 mg, 0.3 mmol, 10 mol%), NaCl (17.5 g), toluene (67 mL) and H_3PO_4–NaH_2PO_4 buffer1 ($c(H_3PO_4) = c(NaH_2PO_4) = 0.5$ M, pH = 2, 90 mL). The vial was placed on a stir plate with a stirring speed of 1500 r/min (caution: The stirring must allow full mixing of the two phases but avoid contact between the stir bars and electrodes). The electrolysis was carried out with an alternating current of 10 mA which alternates its direction every 1 min and the total electrolysis lasted for about 100 – 120 h (2.5 – 3.0 F·mol^{-1}) until substrate **1k** disappeared by TLC analysis. The organic layer was separated, and the aqueous layer was extracted with EtOAc (5 mL × 3). The combined organic layers were dried over anhydrous Na_2SO_4, filtered, and concentrated. The residue was purified by silica gel chromatography (eluent: *n*-hexane/EtOAc = 10:1) to yield the desired product **3k** (0.94 g, 98% yield, 95.5% ee).

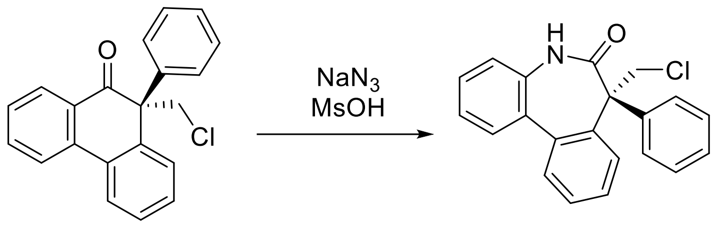

3k, 95.5% ee **5**, 50% yield, 94% ee

(*R*)-7-(Chloromethyl)-7-phenyl-5,7-dihydro-6*H*-dibenzo[b,d]azepin-6-one (5). Under N_2, to a 10-mL Schlenk tube were added **3k** (31.8 mg, 0.1 mmol, 1.0 equiv), NaN_3 (39 mg, 0.6 mmol, 6.0 equiv) and methanesulfonic acid (3 mL). The mixture was stirred at 60 °C for 10 h. The mixture was cooled to room temperature and quenched by addition of ice water dropwise. The organic layer was separated, and the aqueous layer was extracted with DCM (5 mL × 3). The combined organic layers were dried over anhydrous Na_2SO_4, filtered, and concentrated. The residue was purified by flash column chromatography (eluent: n-hexane/ethyl acetate = 1:1) on silica gel to afford the desired product **5** as a white solid (17 mg, 50% yield, 94% ee).

$[\alpha]_D^{25}$: +115.6 (*c* = 1.0, CH_2Cl_2). HPLC analysis of product: Daicel CHIRALPAK® ID-3 column; 20% *i*-PrOH in *n*-hexane; 1.0 mL/min; retention time: 7.4 min (minor), 9.1 min (major).

^{1}H NMR (400 MHz, $CDCl_3$) δ 8.23 (s, 1H), 7.77 (d, *J* = 7.8 Hz, 1H), 7.59 – 7.48 (m, 3H), 7.27 (dd, J_1 = 7.3, J_2 = 1.7 Hz, 1H), 7.19 (d, *J* = 6.5 Hz, 1H), 7.05 – 7.01 (m, 2H), 6.96 – 6.92 (m, 2H), 6.81 (t, *J* = 7.0 Hz, 1H), 6.72 (dd, J_1 = 7.9, J_2 = 1.0 Hz, 1H), 6.47 (d, *J* = 6.7 Hz, 1H), 4.77 (d, *J* = 11.9 Hz, 1H), 4.35 (d, *J* = 11.9 Hz, 1H) ppm.

^{13}C NMR (101 MHz, $CDCl_3$) 173.1, 137.7, 136.9, 136.7, 134.3, 134.2, 131.0, 129.1, 129.0, 128.3, 128.2, 128.1, 127.4, 127.1, 126.6, 124.6, 120.3, 59.6, 50.3 ppm.

HRMS (ES+) Calcd for $C_{21}H_{16}ClNNaO^+$ $[M+Na]^+$: 356.0813, Found: 356.0808.

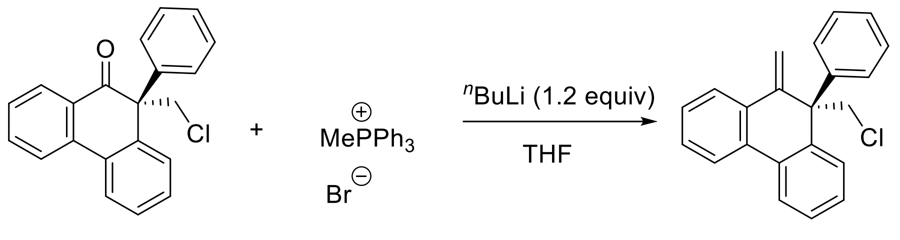

3k, 95.5% ee

6, 81% yield, 95% ee

(*S*)-9-(Chloromethyl)-10-methylene-9-phenyl-9,10-dihydrophenanthrene (6). Under N_2 at 0 °C, to a flask charged with a solution of methyltriphenylphosphonium bromide (85.7 mg, 0.24 mmol, 1.2 equiv) in dry THF (16 mL) was added ^{n}BuLi (0.22 mL, 0.22 mmol, 1.1 equiv, 1.6 M in *n*-hexane) dropwise. The mixture was stirred at the same temperature for 30 h. **3k** (63.6 mg, 0.2 mmol, 1.0 equiv) was then dissolved in dry THF (4 mL) and added slowly. The reaction was allowed to warm to room temperature and stirred overnight before quenched by slow addition of a saturated solution of NH_4Cl (5 mL). The organic layer was separated, and the aqueous layer was extracted with EtOAc (20 mL × 3). The combined organic layers were dried over anhydrous Na_2SO_4, filtered, and concentrated. The residue was purified by flash column chromatography (eluent: n-hexane/ethyl acetate = 10:1) on silica gel to afford the desired product **6** as a white solid (51.1 mg, 81% yield, 95% ee).

$[\alpha]_D^{25}$: +2.9 (*c* = 1.0, CH_2Cl_2). HPLC analysis of product: Daicel CHIRALPAK® IB N-3 column; 1% *i*-PrOH in *n*-hexane; 1.0 mL/min; retention time: 14.4 min (minor), 24.0 min (major).

^{1}H NMR (400 MHz, $CDCl_3$) δ 7.89 (dd, J_1 = 7.9 Hz, J_2 = 1.1 Hz, 1H), 7.79 (dd, J_1 = 7.8 Hz, J_2 = 1.0 Hz, 1H), 7.59 (dd, J_1 = 7.6 Hz, J_2 = 1.3 Hz, 1H), 7.39 – 7.21 (m, 9H), 7.07 (dd, J_1 = 7.9 Hz, J_2 = 1.1 Hz, 1H), 5.79 (s, 1H), 5.09 (s, 1H), 4.11 (d, *J* = 12.0 Hz, 1H), 3.97 (d, *J* = 12.0 Hz, 1H) ppm.

^{13}C NMR (101 MHz, $CDCl_3$) δ 146.8, 142.4, 138.9, 134.1, 133.4, 131.6, 129.7, 129.4, 128.8, 128.3, 127.8, 127.4, 127.0, 126.4, 124.0, 123.2, 116.7, 55.3, 48.3, 30.3 ppm.

HRMS (CI+) Calcd for $C_{22}H_{17}Cl^+$ (M^+): 316.1013, Found: 316.1021.

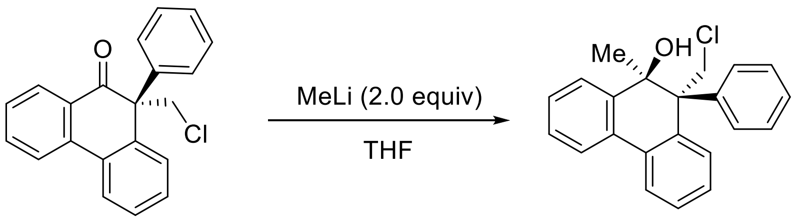

3k, 95.5% ee

7, 67% yield
93% ee, 10:1 dr

(9*R*,10*R*)-10-(Chloromethyl)-9-methyl-10-phenyl-9,10-dihydrophenanthren-9-ol (7). Under N_2 at –78 °C, to a 10-mL Schlenk tube charged with a solution of **3k** (63.6 mg, 0.2 mmol, 1.0 equiv) in dry THF (2 mL) was added methyl lithium (0.25 mL, 0.4 mmol, 2.0 equiv, 1.6 M in THF) dropwise via a syringe. The mixture was stirred at the same temperature for 5 h and quenched by slow addition of a saturated solution of NH_4Cl at the same temperature. The organic layer was separated, and the aqueous layer was extracted with Et_2O (5 mL × 3). The combined organic layers were dried over anhydrous Na_2SO_4, filtered, and concentrated. The residue was purified by flash column chromatography on silica gel (eluent: n-hexane/ethyl acetate = 20:1) to afford the desired product **7** as a white solid (46.0 mg, 67% yield, 93% ee, 10:1 dr).

$[\alpha]_D^{25}$: -62.9 (*c* = 1.0, CH_2Cl_2). HPLC analysis of product: Daicel CHIRALPAK® ID-3 column; 5% *i*-PrOH in *n*-hexane; 1.0 mL/min; retention time: 9.7 min (minor), 10.3 min (major).

^{1}H NMR (400 MHz, $CDCl_3$) major diastereomer δ 7.87 (d, *J* = 7.7 Hz, 1H), 7.76 (d, J_1 = 7.9, 1H), 7.46 (td, J_1 = 7.5 Hz, J_2 = 1.0 Hz, 1H), 7.41 – 7.32 (m, 4H), 7.24 – 7.20 (m, 6H), 4.70 (brs, 1H), 4.21 (brs, 1H), 2.13 (s, 1H), 1.38 (s, 3H) ppm.

^{13}C NMR (101 MHz, $CDCl_3$) major diastereomer δ 137.4, 132.6, 130.2, 128.6, 128.3, 128.2, 127.6, 127.0, 124.2, 123.9, 123.3, 76.2, 56.6 47.9, 23.5 ppm.

HRMS (CI+) Calcd for $C_{22}H_{19}ClO^+$ (M^+): 334.1119, Found: 334.1122.

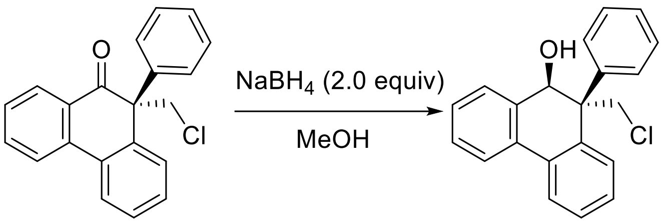

3k, 95.5% ee

8, 94% yield
97% ee, 17:1 dr

(9*R*,10*R*)-10-(Chloromethyl)-10-phenyl-9,10-dihydrophenanthren-9-ol (8). Under N_2 at –78 °C, to a 10-mL Schlenk tube charged with a solution of **3k** (63.6 mg, 0.2 mmol, 1.0 equiv) in MeOH (2 mL) was added $NaBH_4$ (25.1 mg, 0.4 mmol, 2.0 equiv) in one portion. The mixture was stirred at the same temperature for 5 h before it was warmed to –40 °C over another 3 h. The reaction was quenched by slow addition of H_2O (5 mL) and DCM (5 mL). The organic layer was separated and the aqueous layer was extracted with DCM (5 mL × 3). The combined organic layers were dried over anhydrous Na_2SO_4, filtered, and concentrated. The residue was purified by flash column chromatography on silica gel (eluent: n-hexane/ethyl acetate = 10:1) to afford the desired product **8** as a white solid (60.2 mg, 94% yield, 97% ee, 17:1 dr).

$[\alpha]_D^{25}$: +11.9 (*c* = 1.0, CH_2Cl_2). HPLC analysis of product: Daicel CHIRALPAK® AD-3 column; 5% *i*-PrOH in *n*-hexane; 1.0 mL/min; retention time: 17.8 min (minor), 19.8 min (major).

^{1}H NMR (400 MHz, $CDCl_3$) δ 7.87 – 7.85 (m, 1H), 7.65 – 7.61 (m, 1H), 7.56 – 7.52 (m, 1H), 7.50 – 7.42 (m, 3H), 7.29 – 7.24 (m, 2H), 7.19 – 7.16 (m, 2H), 7.11 – 7.07 (m, 3H), 5.50 (d, *J* = 7.4 Hz, 1H), 4.55 (d, *J* = 11.8 Hz, 1H), 4.24 (d, *J* = 11.8 Hz, 1H), 2.39 (d, *J* = 7.5 Hz, 1H) ppm.

^{13}C NMR (101 MHz, $CDCl_3$) δ 139.4, 137.3, 136.7, 134.6, 132.4, 129.0, 128.1 (2C), 127.9 (2C), 127.6 (2C), 127.1, 124.6 (2C), 123.6, 71.8, 53.4, 47.3 ppm.

HRMS (CI+) Calcd for $C_{21}H_{17}ClO^+$ (M^+): 320.0962, Found: 320.0966.

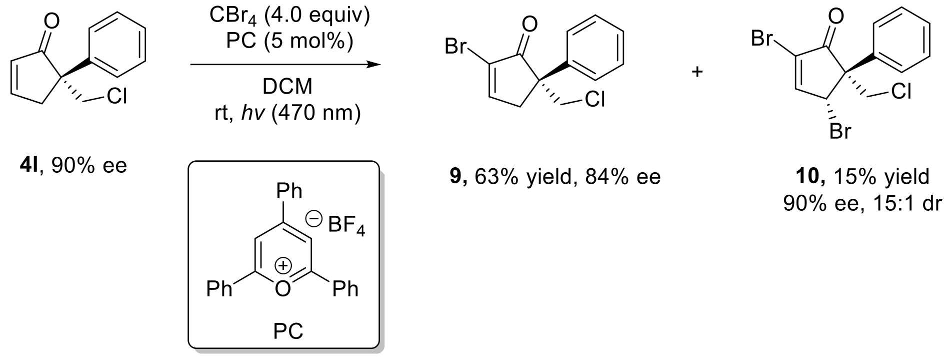

(*S*)-2-Bromo-5-(chloromethyl)-5-phenylcyclopent-2-en-1-one (9) & (4*R*,5*R*)-2,4-dibromo-5-(chloromethyl)-5-phenylcyclopent-2-en-1-one (10). Under N_2, to a 10-mL Schlenk tube were added **4l** (62 mg, 0.3 mmol, 1.0 equiv), CBr_4 (398 mg, 1.2 mmol, 4.0 equiv), 2,4,6-triphenylpyrylium tetrafluoroborate (6.0 mg, 0.015 mmol, 5 mol%) and DCM (2 mL). The mixture was irradiated with 470 nm blue light (50 W) under room temperature for 48 h (A fan was used to keep the reaction cool). After the reaction, brine was added and the organic layer was separated and the aqueous layer was extracted with DCM (5 mL × 3). The combined organic layers were dried over anhydrous Na_2SO_4, filtered, and concentrated. The residue was purified by flash column chromatography (eluent: n-hexane/ethyl acetate = 10:1) on silica gel to afford the desired product **9** (54 mg, 63% yield, 84% ee) & **10** (16 mg, 15% yield, 90% ee, 15:1 dr) as both colorless oils.

Analytic data for **9**: $[\alpha]_D^{25}$: +55.6 (*c* = 1.0, CH_2Cl_2). HPLC analysis of product: Daicel CHIRALPAK® ID-3 column; 5% *i*-PrOH in *n*-hexane; 1.0 mL/min; retention time: 13.0 min (major), 16.2 min (minor).

^{1}H NMR (400 MHz, $CDCl_3$) δ 7.87 (t, *J* = 3.0 Hz, 1H), 7.38 – 7.27 (m, 5H), 4.02 (d, *J* = 10.9 Hz, 1H), 3.86 (d, *J* = 10.9 Hz, 1H), 3.34 (dd, *J* = 19.1, 2.9 Hz, 1H), 3.15 (dd, *J* = 19.1, 3.2 Hz, 1H) ppm.

^{13}C NMR (101 MHz, $CDCl_3$) 200.2, 160.6, 138.4, 129.0, 128.0, 126.5, 124.7, 54.6, 48.9, 41.0 ppm.

HRMS (ES+) Calcd for $C_{12}H_{14}BrClNO^+$ ($M+NH_4^+$): 301.9942, Found: 301.1424.

Analytic data for **10**. $[\alpha]_D^{25}$: -77.4 (*c* = 1.0, CH_2Cl_2). HPLC analysis of product: Daicel CHIRALPAK® AS-3 column; 5% *i*-PrOH in *n*-hexane; 1.0 mL/min; retention time: 8.2

min (minor), 11.5 min (major).

^{1}H NMR (400 MHz, $CDCl_3$) δ 7.80 (d, *J* = 2.8 Hz, 1H), 7.43 – 7.33 (m, 5H), 5.49 (d, *J* = 2.8 Hz, 1H), 4.15 (d, *J* = 11.3 Hz, 1H), 4.09 (d, *J* = 11.4 Hz, 1H) ppm.

^{13}C NMR (101 MHz, $CDCl_3$) 195.8, 157.8, 137.1, 129.3, 128.7, 127.4, 126.3, 57.5, 54.0, 49.8 ppm.

HRMS (CI+) Calcd for $C_{12}H_9Br_2ClO^+$ (M^+): 361.8703, Found: 361.9077.

IX. Determination of Products Stereochemistry

The structure and absolute stereochemistry of **3k**, **3u**, **3w**, and **4a'** were determined by single crystal X-ray crystallography. The X-ray data have been deposited at the Cambridge Crystallographic Data Center (CCDC 2441283, 2441583, 2441584 and 2441725) and are available free of charge at www.ccdc.cam.ac.uk/conts/retrieving.html.

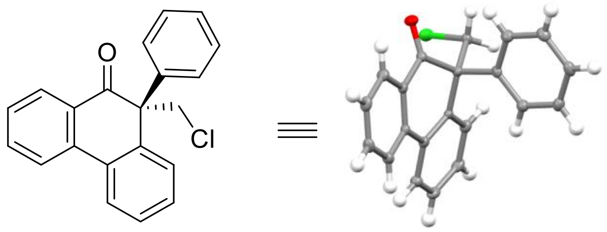

3k CCDC 2441283

Table S9. Crystal Data and Structure Refinement for 3k

Identification code	**3k**
Empirical formula	$C_{21}H_{15}ClO$
Formula weight	318.78
Temperature/K	99.97(11)
Crystal system	monoclinic
Space group	$P2_1$
a/Å	9.0535(3)
b/Å	8.9517(3)
c/Å	9.7504(3)
α/°	90
β/°	100.931(3)
γ/°	90
Volume/Å^3	775.87(4)
Z	2

ρ_{calc}g/cm^3	1.365
μ/mm^{-1}	0.248
F(000)	332.0
Crystal size/mm^3	0.32 × 0.3 × 0.25
Radiation	MoKα (λ = 0.71073)
2Θ range for data collection/°	5.632 to 59.904
Index ranges	-9 ≤ h ≤ 11, -10 ≤ k ≤ 11, -10 ≤ l ≤ 12
Reflections collected	5586
Independent reflections	3480 [R_{int} = 0.0179, R_{sigma} = 0.0359]
Data/restraints/parameters	3480/1/208
Goodness-of-fit on F^2	1.036
Final R indexes [I>=2σ (I)]	R_1 = 0.0304, wR_2 = 0.0668
Final R indexes [all data]	R_1 = 0.0336, wR_2 = 0.0690
Largest diff. peak/hole / e Å^{-3}	0.25/-0.21
Flack parameter	-0.03(2)

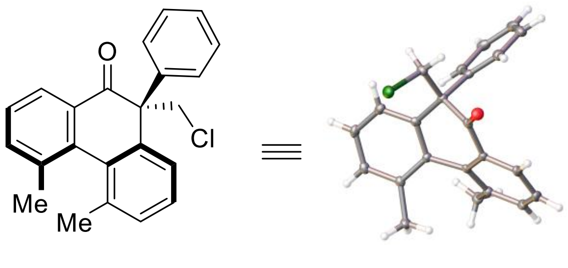

3u CCDC 2441583

Table S10. Crystal Data and Structure Refinement for 3u

Identification code	**3u**
Empirical formula	$C_{23}H_{19}ClO$
Formula weight	346.83
Temperature/K	100.00
Crystal system	orthorhombic

Space group	$P2_12_12_1$
a/Å	10.5746(3)
b/Å	12.5803(4)
c/Å	13.0213(4)
α/°	90
β/°	90
γ/°	90
Volume/Å^3	1732.24(9)
Z	4
ρ_{calc}g/cm^3	1.330
μ/mm^{-1}	1.312
F(000)	728.0
Crystal size/mm^3	0.16 × 0.15 × 0.08
Radiation	GaKα (λ = 1.34139)
2Θ range for data collection/°	8.502 to 118.81
Index ranges	-13 ≤ h ≤ 13, -16 ≤ k ≤ 16, -16 ≤ l ≤ 16
Reflections collected	31843
Independent reflections	3822 [R_{int} = 0.0829, R_{sigma} = 0.0369]
Data/restraints/parameters	3822/0/228
Goodness-of-fit on F^2	1.048
Final R indexes [I>=2σ (I)]	R_1 = 0.0349, wR_2 = 0.0879
Final R indexes [all data]	R_1 = 0.0358, wR_2 = 0.0886
Largest diff. peak/hole / e Å^{-3}	0.32/-0.20
Flack parameter	0.008(8)

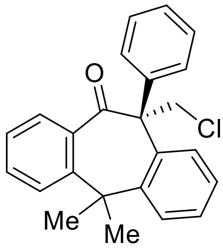

3w

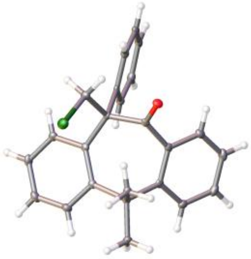

CCDC 2441584

Table S11. Crystal Data and Structure Refinement for 3w

Identification code	**3w**
Empirical formula	$C_{24}H_{21}ClO$
Formula weight	360.86
Temperature/K	100.00
Crystal system	orthorhombic
Space group	$P2_12_12_1$
a/Å	7.0853(2)
b/Å	14.8168(5)
c/Å	16.8627(5)
α/°	90
β/°	90
γ/°	90
Volume/Å^3	1770.27(9)
Z	4
ρ_{calc}g/cm^3	1.354
μ/mm^{-1}	1.297
F(000)	760.0
Crystal size/mm^3	0.08 × 0.06 × 0.05
Radiation	GaKα (λ = 1.34139)
2Θ range for data collection/°	6.91 to 116.21

Index ranges	-8 ≤ h ≤ 8, -18 ≤ k ≤ 18, -21 ≤ l ≤ 21
Reflections collected	49423
Independent reflections	3746 [R_{int} = 0.0686, R_{sigma} = 0.0269]
Data/restraints/parameters	3746/0/237
Goodness-of-fit on F^2	1.074
Final R indexes [I>=2σ (I)]	R_1 = 0.0362, wR_2 = 0.0893
Final R indexes [all data]	R_1 = 0.0377, wR_2 = 0.0903
Largest diff. peak/hole / e Å^{-3}	0.37/-0.20
Flack parameter	0.004(7)

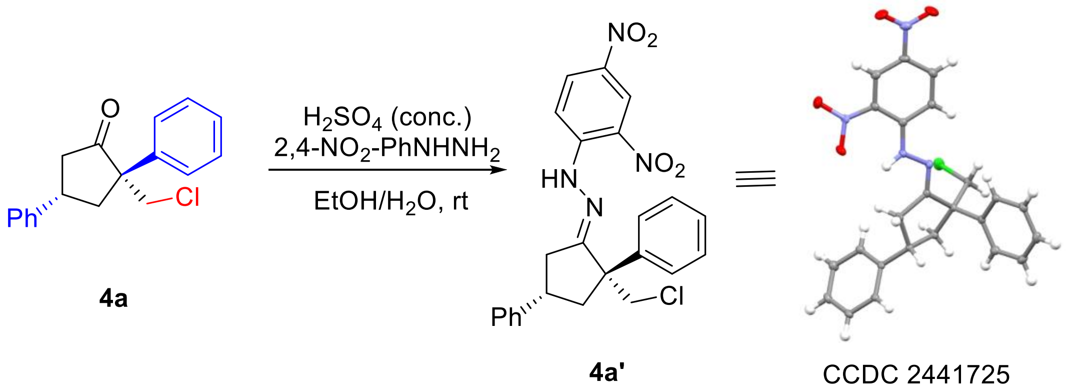

Table S12. Crystal Data and Structure Refinement for 4a′

Identification code	**4a′**
Empirical formula	$C_{24}H_{21}ClN_4O_4$
Formula weight	464.90
Temperature/K	100.00(10)
Crystal system	orthorhombic
Space group	$P2_12_12_1$
a/Å	7.6843(2)
b/Å	13.6011(5)
c/Å	21.3044(7)

α/°	90
β/°	90
γ/°	90
Volume/Å^3	2226.63(12)
Z	4
ρ_{calc}g/cm^3	1.387
μ/mm^{-1}	1.214
F(000)	968.0
Crystal size/mm^3	0.135 × 0.055 × 0.041
Radiation	GaKα (λ = 1.34139)
2Θ range for data collection/°	6.708 to 127.166
Index ranges	-8 ≤ h ≤ 10, -17 ≤ k ≤ 18, -27 ≤ l ≤ 28
Reflections collected	42422
Independent reflections	5530 [R_{int} = 0.0574, R_{sigma} = 0.0383]
Data/restraints/parameters	5530/239/381
Goodness-of-fit on F^2	1.071
Final R indexes [I>=2σ (I)]	R_1 = 0.0309, wR_2 = 0.0776
Final R indexes [all data]	R_1 = 0.0323, wR_2 = 0.0782
Largest diff. peak/hole / e Å^{-3}	0.24/-0.19
Flack parameter	0.015(5)

The structure and relative stereochemistry of **8** were determined NOSEY spectrum. The structure and relative stereochemistry of **7** and **10** were determined by analogy.

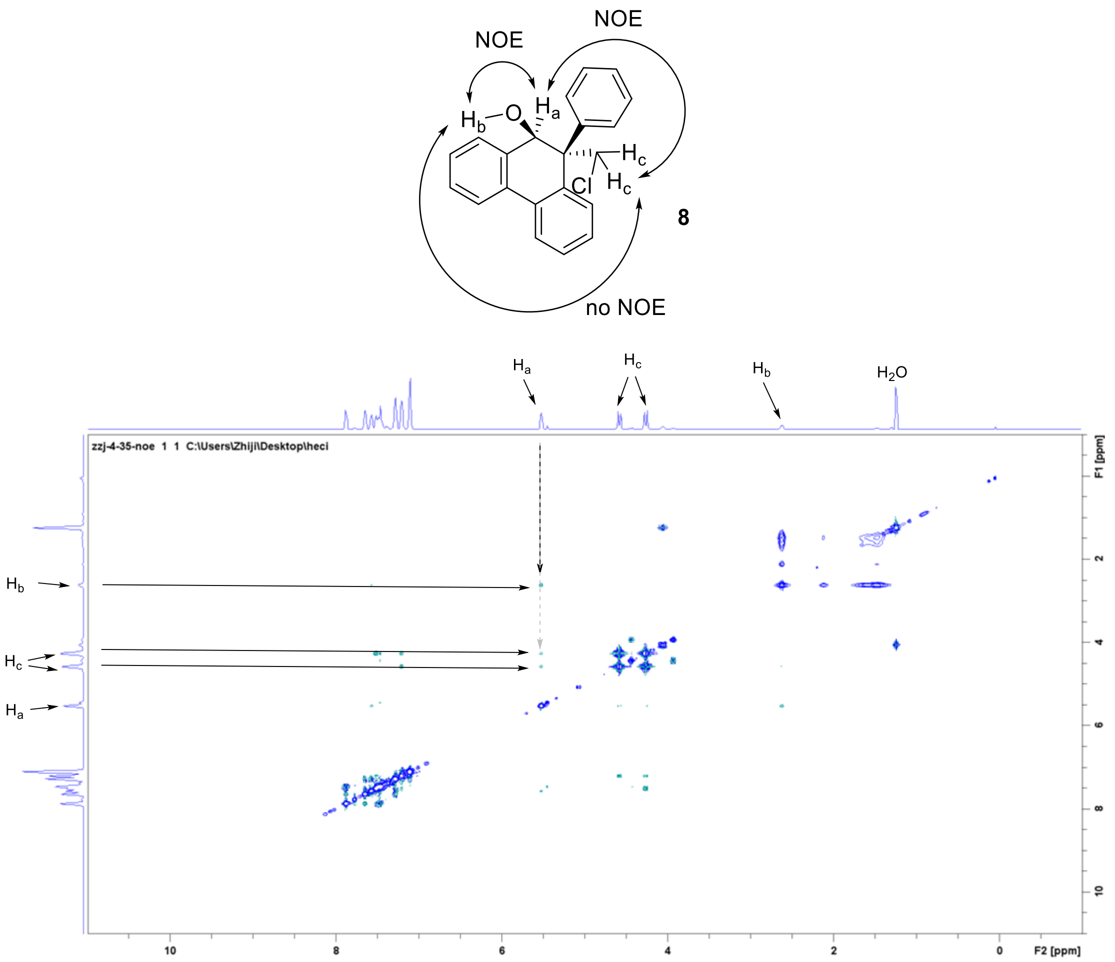

Figure S10. NOESY for compound**8**.

X. DFT Calculations

All structures were optimized and characterized in toluene or water with the SMD[7] solvent model (SCRF = SMD) at M06-2X[8]/6-31G(d) level. Harmonic frequency analysis calculations at the same level were performed to verify the optimized geometries to be minima (no imaginary frequency). The energies were further improved by M06-2X/6-311+G(d, p)//M06-2X/6-31G(d) single-point calculations with solvent effects accounted by the SMD solvent model, using the experimental solvent (toluene or water). All DFT calculations were carried out using Gaussian 16 program.[9] Computed structures are illustrated using the CYLview.[10]

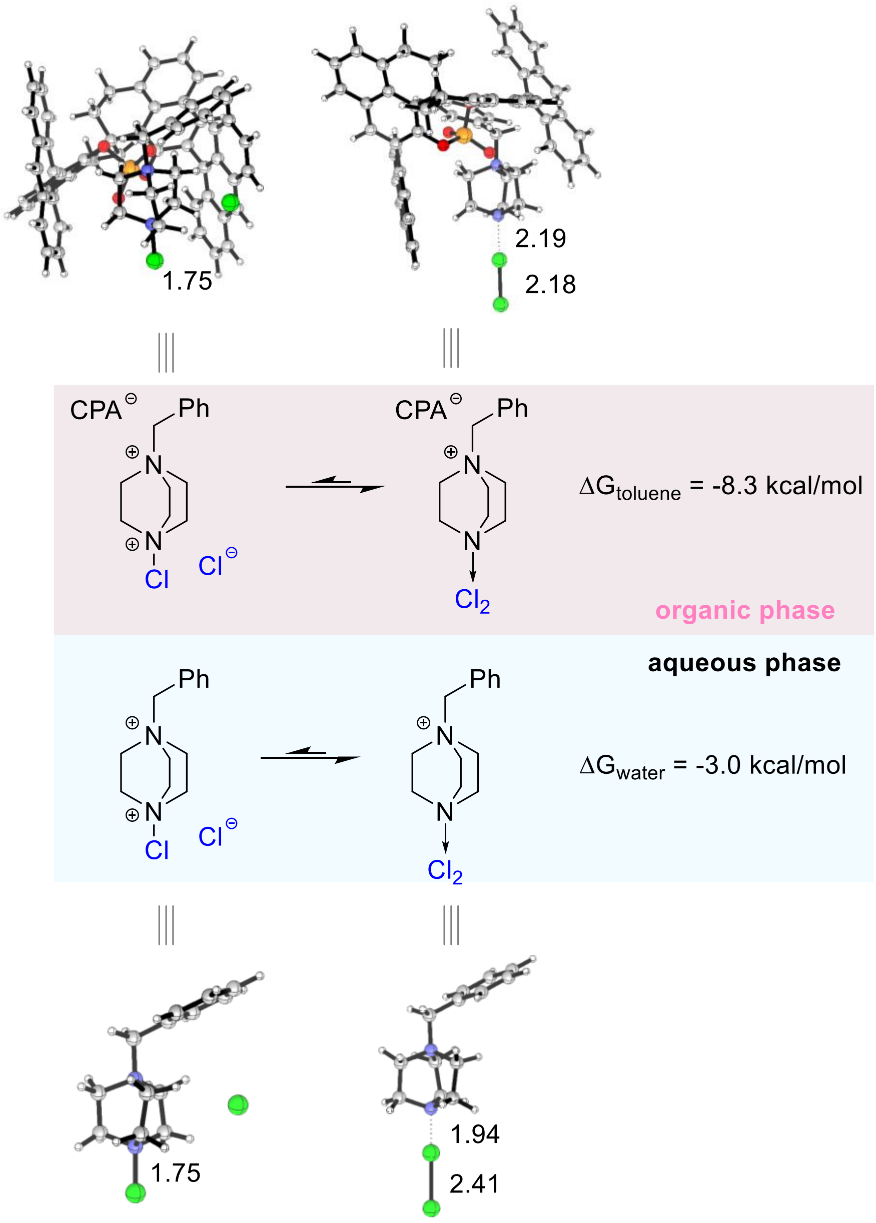

Figure S11. DFT calculations comparing the energies of possible PTC-bonded species. The results revealed that the formation of a loosely associated PTC•Cl_2 species is thermodynamically more favorable than forming a covalent N–Cl bond (e.g., $[R_3N–Cl]^+Cl^-$) in both organic and aqueous phases. CPA was (*R*)-**D1**. The key bond lengths in the 3D structures are given in angstroms.

Cartesian Coordinates in Å, SCF Energies and Free Energies (in a.u.) at 298.15 K and 1 atm for the Optimized Structures [BSI= 6-31G(d), BSII=6-311+G(d,p)]

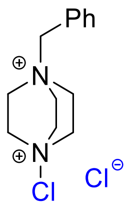

M06-2X/BSI SCF energy in water: -1536.180539 a.u.

M06-2X/BSII SCF energy in water: -1536.426947 a.u.

M06-2X/BSII free energy in water: -1536.152616 a.u.

C	-2.78892800	-1.44581300	0.18512700
C	-1.45451500	-2.16712100	-0.05636500
H	-3.15774300	-1.57218300	1.20355300
H	-3.53837300	-1.76394200	-0.53855200
H	-1.29774100	-2.92875000	0.70768900
H	-1.41339400	-2.61847700	-1.04857200
C	-1.71035000	0.55383000	1.13151500
H	-1.41682800	1.57486300	0.87344100
H	-2.32084700	0.52333300	2.03335100
C	-0.48173200	-0.35559200	1.26736900
H	0.40275700	0.27535800	1.37168000
H	-0.56913300	-1.05006700	2.10432600
C	-0.39884300	-0.27437600	-1.17177600
H	0.30125100	0.54821400	-1.00541300
H	-0.12796600	-0.85743500	-2.05290900
C	-1.82944300	0.26598900	-1.31342200
H	-1.81208800	1.34217600	-1.48857000
H	-2.40246100	-0.24565200	-2.08737000
N	-0.32354200	-1.18099200	0.02313100

N	-2.55003400	0.03108500	-0.00815400
Cl	0.71407500	2.74334600	0.03192900
Cl	-4.08372900	0.87536500	-0.03488800
C	0.98752700	-1.95861700	0.04675100
H	0.94703500	-2.55434300	0.96119600
H	0.93916200	-2.61913200	-0.82199000
C	2.20641800	-1.08136100	0.01087400
C	2.75780300	-0.68471700	-1.21133100
C	2.80717100	-0.66627400	1.20310400
C	3.87664700	0.14289700	-1.23979900
H	2.30747000	-1.02615500	-2.13971500
C	3.92729800	0.16046700	1.17454500
H	2.39403400	-0.99421500	2.15370800
C	4.45907300	0.56962100	-0.04705200
H	4.29663500	0.45000400	-2.19206000
H	4.38647100	0.47996700	2.10433900
H	5.33214700	1.21409600	-0.06990300

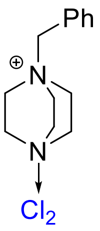

M06-2X/BSI SCF energy in water: -1536.18876 a.u.

M06-2X/BSII SCF energy in water: -1536.42973 a.u.

M06-2X/BSII free energy in water: -1536.157373 a.u.

C	1.63857300	1.70910300	0.11790000
C	0.19228900	2.15424700	-0.15410600
H	1.96570200	1.97030700	1.12529000

H	2.31704300	2.14461600	-0.61603100
H	-0.12723700	2.90823500	0.56538900
H	0.06120500	2.53137500	-1.16950900
C	0.99013400	-0.38916000	1.14609600
H	0.92365100	-1.46043600	0.95131000
H	1.57669800	-0.21376100	2.04826400
C	-0.39941100	0.26028400	1.27320900
H	-1.16100300	-0.49960500	1.44526800
H	-0.43542900	1.01112700	2.06469400
C	-0.45970600	0.02780900	-1.15809200
H	-0.99030800	-0.90052700	-0.94545700
H	-0.85852200	0.48530500	-2.06464800
C	1.05900400	-0.19579900	-1.27356900
H	1.27668700	-1.25261400	-1.42949800
H	1.49959600	0.39448600	-2.07804000
N	-0.72549600	0.96729100	-0.01356800
N	1.69761400	0.23078000	-0.00220300
Cl	5.85653900	-1.05447700	0.01772700
Cl	3.55128100	-0.34065500	0.00624500
C	-2.15875400	1.47722900	-0.02294900
H	-2.23651400	2.11906000	0.85730900
H	-2.23440800	2.08948400	-0.92459100
C	-3.20031100	0.39368300	-0.00751700
C	-3.68535800	-0.13598000	-1.20732800
C	-3.72078900	-0.06621800	1.20597700
C	-4.66079500	-1.12911900	-1.19272200
H	-3.30040800	0.23720600	-2.15277400
C	-4.69660300	-1.05962500	1.22085600
H	-3.36308600	0.36246200	2.13881300

C	-5.16440700	-1.59388900	0.02153000
H	-5.03222900	-1.53455700	-2.12822800
H	-5.09523800	-1.41069200	2.16706100
H	-5.92715200	-2.36610700	0.03254600

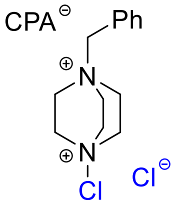

M06-2X/BSI SCF energy in toluene: -4217.952237 a.u.

M06-2X/BSII SCF energy in toluene: -4218.839805 a.u.

M06-2X/BSII free energy in toluene: -4217.880714 a.u.

C	-4.46394300	3.03959500	2.55832000
C	-4.66852000	4.09074400	3.42535700
C	-3.80839500	5.20626000	3.45658500
C	-2.73605600	5.25021300	2.60491300
C	-2.48255000	4.18441400	1.70543600
C	-3.34418900	3.04721400	1.67021800
H	-5.52015900	4.05258000	4.10059300
H	-3.99841800	6.01879700	4.15089500
H	-2.05732600	6.09891300	2.60368300
C	-3.06574000	1.96562500	0.75231800
C	-1.37618300	4.25812600	0.82328400
C	-1.08466800	3.23030500	-0.03069900
C	-1.94215300	2.08635700	-0.03430000
H	-0.74936300	5.14647500	0.83843900
C	-5.43776600	1.89165600	2.54346400
H	-6.33164300	2.18566600	1.97649400

H	-5.77991500	1.68447900	3.56331500
C	-4.83017200	0.63940900	1.93447000
H	-5.59681400	-0.13541700	1.83290100
H	-4.05576200	0.23799000	2.60005800
C	-6.19874700	-0.60662900	-0.97296200
C	-7.18077700	-1.32044500	-1.62388200
C	-7.01700800	-2.68037900	-1.95395700
C	-5.85082300	-3.31459900	-1.61693300
C	-4.81245100	-2.61395300	-0.95292700
C	-4.96061800	-1.23243700	-0.62713000
H	-8.10635100	-0.81361300	-1.88643100
H	-7.81027900	-3.21387700	-2.46816800
H	-5.69709400	-4.36465700	-1.85052200
C	-3.88094200	-0.53021400	0.03118100
C	-3.63176900	-3.30097400	-0.58185800
C	-2.60655000	-2.65255400	0.05085500
C	-2.74782000	-1.25767600	0.32736400
H	-3.55088100	-4.36329000	-0.79910400
C	-6.44301400	0.83274700	-0.61316300
H	-7.03400700	0.88008000	0.31175700
H	-7.05122000	1.31057300	-1.38873800
C	-5.13940300	1.58910100	-0.43320600
H	-5.34293300	2.60882300	-0.09266000
H	-4.62206600	1.67191400	-1.39713800
C	-4.16394700	0.89653900	0.55649400
O	-1.66168300	-0.64792600	0.90756300
O	-1.58555500	1.07276600	-0.88991200
P	-0.59346900	-0.04966500	-0.20620700
O	-0.31191900	-1.03949800	-1.28799300

O	0.51157200	0.56093300	0.59040500
C	1.00917600	-5.46756400	-2.35975000
C	1.25455700	-5.62787100	-1.02797900
C	0.47573800	-4.93024500	-0.04637900
C	-0.59509400	-4.07261900	-0.47769600
C	-0.79077000	-3.90645600	-1.89007200
C	-0.01884900	-4.57721500	-2.79402500
C	0.73866200	-5.07398400	1.31698700
C	-1.40013700	-3.41957500	0.48223500
C	-1.12197900	-3.57638000	1.85800400
C	-0.02448300	-4.40579800	2.27533100
C	0.25183400	-4.55347400	3.67275200
H	1.08695400	-5.18411300	3.96709500
C	-0.51730700	-3.92973400	4.60797700
C	-1.62309700	-3.12584500	4.20100600
C	-1.91617000	-2.95901500	2.87976300
H	1.54385600	-5.73162800	1.63995000
H	1.59666000	-6.00854600	-3.09585900
H	2.03757200	-6.29780100	-0.68034900
H	-1.54131100	-3.20224200	-2.22732700
H	-0.18146200	-4.42868700	-3.85699700
H	-0.30165900	-4.04878500	5.66528000
H	-2.23828100	-2.64567600	4.95535600
H	-2.76818300	-2.35449300	2.58949800
C	-1.58485400	3.28218100	-4.29067200
C	-1.41917900	3.27723700	-2.93731400
C	-0.11266400	3.29848500	-2.34764700
C	1.01839900	3.34468600	-3.23202500
C	0.80279000	3.34089100	-4.64773200

C	-0.45708700	3.30777100	-5.16410200
C	0.08851400	3.29437400	-0.95074600
C	2.30612700	3.40241100	-2.69961400
C	2.51976800	3.42801700	-1.32047500
C	1.39648400	3.37096700	-0.42476500
C	1.66895700	3.38255900	0.98369900
H	0.84081300	3.29124700	1.67577000
C	2.94603900	3.48974000	1.45350200
C	4.05847600	3.53036600	0.56034000
C	3.84818700	3.48732300	-0.78540300
H	-2.58502700	3.26765500	-4.71255300
H	-2.28800000	3.26731900	-2.28755600
H	1.67302900	3.37208700	-5.29834200
H	-0.61125400	3.30801100	-6.23876300
H	3.16255300	3.43971100	-3.37035900
H	3.12090900	3.52429300	2.52564000
H	5.07144800	3.56163600	0.94981400
H	4.68922900	3.47664000	-1.47320200
C	2.31366300	-2.30260600	-1.27734200
C	2.44219800	-2.03613900	0.22405600
H	2.67820000	-3.28692200	-1.57590800
H	1.27622900	-2.13898300	-1.58377700
H	2.51620700	-2.97600300	0.77474000
H	1.60054400	-1.44267200	0.59115400
C	4.62676600	-1.64549700	-1.76053400
H	5.23939100	-0.81440200	-2.12824700
H	4.82844000	-2.57135100	-2.29946000
C	4.83944900	-1.81491600	-0.24692900
H	5.73909500	-1.25376400	0.02224800

H	4.91245600	-2.86365400	0.05078400
C	3.45446800	0.17895100	-0.00884100
H	4.41975000	0.69374500	0.01678400
H	2.69565000	0.65375400	0.61938200
C	2.91997800	0.10087100	-1.44670100
H	3.46400200	0.79669100	-2.08710000
H	1.84018100	0.25381400	-1.49909200
N	3.67928000	-1.22560800	0.49867200
N	3.18273800	-1.29245700	-1.98225500
Cl	6.57917400	0.94126700	-1.21066000
Cl	2.82036300	-1.32584200	-3.69566300
C	3.91793100	-1.24691600	2.00204600
H	4.14299500	-2.29101900	2.23927500
H	2.95162400	-0.98271000	2.43994000
C	4.99985200	-0.31851800	2.46792100
C	4.66111100	0.96666300	2.89491500
C	6.33702800	-0.72302100	2.49560400
C	5.65067400	1.84901600	3.31574700
H	3.61824800	1.27227900	2.89317100
C	7.32737500	0.16206600	2.90706800
H	6.60554900	-1.73255000	2.19297400
C	6.98489700	1.44951000	3.31339200
H	5.38058100	2.84833700	3.64426900
H	8.36548500	-0.15268200	2.91028600
H	7.75885600	2.14020900	3.63219400

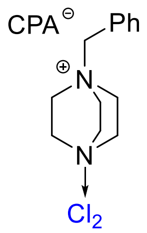

M06-2X/BSI SCF energy in toluene: -4217.964169 a.u.

M06-2X/BSII SCF energy in toluene: -4218.845678 a.u.

M06-2X/BSII free energy in toluene: -4217.893924 a.u.

C	5.32757200	-2.08094200	1.97589500
C	5.89923400	-2.80825300	2.99675000
C	5.20387600	-3.84588600	3.64884000
C	3.92388200	-4.14368000	3.26087100
C	3.29437600	-3.41429000	2.22123100
C	3.98723000	-2.35439300	1.56167900
H	6.91564400	-2.57058900	3.30184400
H	5.68325000	-4.40051000	4.44948200
H	3.36615900	-4.94299100	3.74153900
C	3.32573900	-1.60818400	0.51630000
C	1.98217000	-3.76225900	1.81175400
C	1.34434800	-3.06162100	0.82543300
C	2.03198500	-1.96822600	0.21447300
H	1.48729500	-4.60479500	2.28892000
C	6.12874600	-1.00688500	1.28943500
H	6.80043600	-1.47109400	0.55415600
H	6.77550400	-0.50626600	2.01820700
C	5.23511500	0.01034500	0.60048600
H	5.84454800	0.71727800	0.02852300
H	4.68745100	0.59409300	1.35101100
C	5.41113400	0.35786100	-2.83025900

C	6.03057200	0.85296200	-3.95657300
C	5.61370600	2.05258800	-4.56697100
C	4.56573900	2.75058500	-4.02719300
C	3.89587800	2.27240600	-2.87298200
C	4.30058000	1.05069900	-2.25571000
H	6.86413100	0.29817000	-4.38073000
H	6.12256400	2.41528200	-5.45460600
H	4.22692500	3.68274100	-4.47128700
C	3.58994800	0.56611700	-1.09457300
C	2.84298400	3.03296100	-2.30681900
C	2.17339200	2.59448900	-1.19722500
C	2.55023400	1.33937100	-0.62728400
H	2.58063000	3.98641400	-2.75892600
C	5.91811500	-0.90950300	-2.19762700
H	6.78439000	-0.67791200	-1.56261500
H	6.28253100	-1.59069500	-2.97415000
C	4.83980300	-1.58883900	-1.37197300
H	5.25448600	-2.46298000	-0.86041400
H	4.04239700	-1.95332900	-2.03167200
C	4.18165400	-0.64203900	-0.33309700
O	1.79173400	0.90636700	0.43204200
O	1.31602100	-1.25818000	-0.71614600
P	0.48959200	0.00932000	-0.05672100
O	-0.22062800	0.65199100	-1.19797700
O	-0.20885100	-0.37289900	1.20800900
C	-2.37648800	4.61498600	-2.66194100
C	-2.11897600	5.14824700	-1.43350200
C	-0.96048900	4.75176000	-0.68681500
C	-0.04180200	3.80612200	-1.26180000

C	-0.37543700	3.24280800	-2.53877100
C	-1.49862600	3.63142600	-3.21106900
C	-0.71360700	5.26892000	0.58507900
C	1.13072700	3.45324000	-0.55887700
C	1.36977400	3.99140000	0.72569300
C	0.42041000	4.89880100	1.30883600
C	0.66716400	5.43014000	2.61517400
H	-0.06741500	6.10957100	3.03968000
C	1.79381800	5.09837300	3.30472400
C	2.75271400	4.21908600	2.72096400
C	2.55084800	3.68974900	1.48057200
H	-1.41141400	5.98467900	1.01581300
H	-3.25092400	4.92826400	-3.22517800
H	-2.78146200	5.89200200	-0.99683200
H	0.26587000	2.46943700	-2.94331900
H	-1.73100800	3.18788200	-4.17450800
H	1.97214400	5.50562000	4.29509300
H	3.65400000	3.97171700	3.27305200
H	3.29731600	3.03232500	1.04894500
C	0.75567700	-4.52785200	-3.15091100
C	0.93471900	-4.06473300	-1.88066500
C	-0.16805600	-3.93563000	-0.97416700
C	-1.46591600	-4.33403900	-1.44241200
C	-1.61409300	-4.80881500	-2.78476500
C	-0.53953500	-4.90038600	-3.61688700
C	-0.01413300	-3.45281600	0.34445600
C	-2.56046100	-4.26478200	-0.57977500
C	-2.41952400	-3.81035000	0.73092100
C	-1.13031500	-3.38267900	1.20321900

C	-1.04514600	-2.87740400	2.54332200
H	-0.09315300	-2.49440000	2.89147400
C	-2.13937700	-2.84708700	3.35745900
C	-3.41231100	-3.29765700	2.89322700
C	-3.54604800	-3.75593700	1.61661500
H	1.60722600	-4.61529100	-3.81851600
H	1.92900900	-3.79520600	-1.54100500
H	-2.60701100	-5.09649900	-3.12031200
H	-0.66084800	-5.26107100	-4.63356300
H	-3.54023500	-4.57864400	-0.93521500
H	-2.04943100	-2.46798800	4.37031700
H	-4.26755000	-3.27266500	3.56228600
H	-4.50672200	-4.10508400	1.24459600
C	-4.02205800	1.10263800	-1.75547300
C	-3.00858400	1.67183100	-0.74430200
H	-4.70279000	1.86984400	-2.13430800
H	-3.49966700	0.65033300	-2.60120600
H	-3.00299000	2.76417700	-0.75363700
H	-1.99140700	1.29985700	-0.91279500
C	-5.70067200	0.69004600	-0.08617900
H	-6.18844000	-0.11007500	0.47534400
H	-6.46976800	1.27117500	-0.59936200
C	-4.85799900	1.59601600	0.84138000
H	-5.10990600	1.44039000	1.89081600
H	-4.96252900	2.65644400	0.59571500
C	-3.26814800	-0.25425500	0.78379400
H	-3.76167800	-0.53348700	1.71823500
H	-2.19920800	-0.48504800	0.85769600
C	-3.93496200	-0.90763000	-0.44215900

H	-4.52241200	-1.77706200	-0.13938100
H	-3.19064000	-1.22764100	-1.17600000
N	-3.41359100	1.24728300	0.65022100
N	-4.82864000	0.06814100	-1.09063600
Cl	-7.36663800	-1.97104100	-4.00786100
Cl	-6.09068800	-0.95263900	-2.55687600
C	-2.50442700	1.97735500	1.62543300
H	-2.64819500	3.04142500	1.41587700
H	-1.49192100	1.68212100	1.33732700
C	-2.75742100	1.66514300	3.07437400
C	-2.03853000	0.64128200	3.69883900
C	-3.65836500	2.43237400	3.82042600
C	-2.23810300	0.38467700	5.05382000
H	-1.31599000	0.07089000	3.11656100
C	-3.86173200	2.16662300	5.17098800
H	-4.18918400	3.25366800	3.34318700
C	-3.15030700	1.13932700	5.78803600
H	-1.66566800	-0.39798300	5.54224600
H	-4.56033100	2.76863600	5.74341100
H	-3.29724100	0.93720200	6.84457600

XI. References

(1) Li, H.; Zhang, F.-M.; Tu, Y.-Q.; Zhang, Q.-W.; Chen, Z.-M.; Chen, Z.-H.; Li, J. Enantioselective Bromination/Semipinacol Rearrangement for the Synthesis of β-Bromoketones Containing an All-α-Carbon Quaternary Center. *Chem. Sci.* **2011**, *2*, 1839–1841.

(2) Azzi, E.; Rodríguez-Martínez, M.; Kolusu, S. R. N.; Scarfiello, J.; Varela, J. A.; Nappi, M. Photolysis of CO_2 Carbamate for Hydrocarboxylation Reactions. *J. Am. Chem. Soc.* **2026**, *148*, 3801–3810.

(3) Yin, Q.; You, S.-L. Asymmetric chlorination/ring expansion for the synthesis of α-quaternary cycloalkanones. *Org. Lett.* **2014**, *16*, 1810–1813.

(4) Tan, X.; Wang, Q.; Sun, J. Electricity-driven asymmetric bromocyclization enabled by chiral phosphate anion phase-transfer catalysis. *Nat. Commun.* **2023**, *14*, 357.

(5) Wang, Y.-M.; Wu, J.; Hoong, C., Rauniyar, V.; Toste, F. D. Enantioselective halocyclization using reagents tailored for chiral anion phase-transfer catalysis. *J. Am. Chem. Soc.* **2012**, *134*, 12928–12931.

(6) Li, D.-H.; Yang, L.-P.; Chai, H.; Jia, F.; Ke, H.; Jiang, W. Temperature-induced large amplitude conformational change in the complex of oxatub[4]arene revealed via rotaxane synthesis. *Org. Chem. Front.* **2019**, *6*, 1027–1031.

(7) Marenich, A. V.; Cramer, C. J.; Truhlar, D. G. Universal Solvation Model Based on Solute Electron Density and on a Continuum Model of the Solvent Defined by the Bulk Dielectric Constant and Atomic Surface Tensions. *J. Phys. Chem. B.* **2009**, *113*, 6378–6396.

(8) (a) Zhao, Y.; Truhlar, D. G. Benchmark Energetic Data in a Model System for Grubbs II Metathesis Catalysis and Their Use for the Development, Assessment, and Validation of Electronic Structure Methods. *J. Chem. Theory Comput.* **2009**, *5*, 324–333; (b) Zhao, Y.; Truhlar, D. G. The M06 suite of density functionals for main group thermochemistry, thermochemical kinetics, noncovalent

interactions, excited states, and transition elements: two new functionals and systematic testing of four M06-class functionals and 12 other functionals. *Theor. Chem. Acc.* **2008**, *120*, 215–241; (c) Zhao, Y.; Truhlar, D. G. Density Functionals with Broad Applicability in Chemistry. *Acc. Chem. Res.* **2008**, *41*, 157–167.

(9) Frisch, M. J.; Trucks, G. W.; Schlegel, H. B.; Scuseria, G. E.; Robb, M. A.; Cheeseman, J. R.; Scalmani, G.; Barone, V.; Petersson, G. A.; Nakatsuji, H.; Li, X.; Caricato, M.; Marenich, A. V.; Bloino, J.; Janesko, B. G.; Gomperts, R.; Mennucci, B.; Hratchian, H. P.; Ortiz, J. V.; Izmaylov, A. F.; Sonnenberg, J. L.; Williams-Young, D.; Ding, F.; Lipparini, F.; Egidi, F.; Goings, J.; Peng, B.; Petrone, A.; Henderson, T.; Ranasinghe, D.; Zakrzewski, V. G.; Gao, J.; Rega, N.; Zheng, G.; Liang, W.; Hada, M.; Ehara, M.; Toyota, K.; Fukuda, R.; Hasegawa, J.; Ishida, M.; Nakajima, T.; Honda, Y.; Kitao, O.; Nakai, H.; Vreven, T.; Throssell, K.; Montgomery, J. A., Jr.; Peralta, J. E.; Ogliaro, F.; Bearpark, M. J.; Heyd, J. J.; Brothers, E. N.; Kudin, K. N.; Staroverov, V. N.; Keith, T. A.; Kobayashi, R.; Normand, J.; Raghavachari, K.; Rendell, A. P.; Burant, J. C.; Iyengar, S. S.; Tomasi, J.; Cossi, M.; Millam, J. M.; Klene, M.; Adamo, C.; Cammi, R.; Ochterski, J. W.; Martin, R. L.; Morokuma, K.; Farkas, O.; Foresman, J. B.; Fox, D. J. Gaussian 16, Revision A.03, Gaussian, Inc.: Wallingford, CT, 2016.

(10) Legault, C. Y. *CYLView, version 1.0 b*; Université de Sherbrooke: Sherbrooke, Quebec, Canada, 2009. http://www.cylview.org.

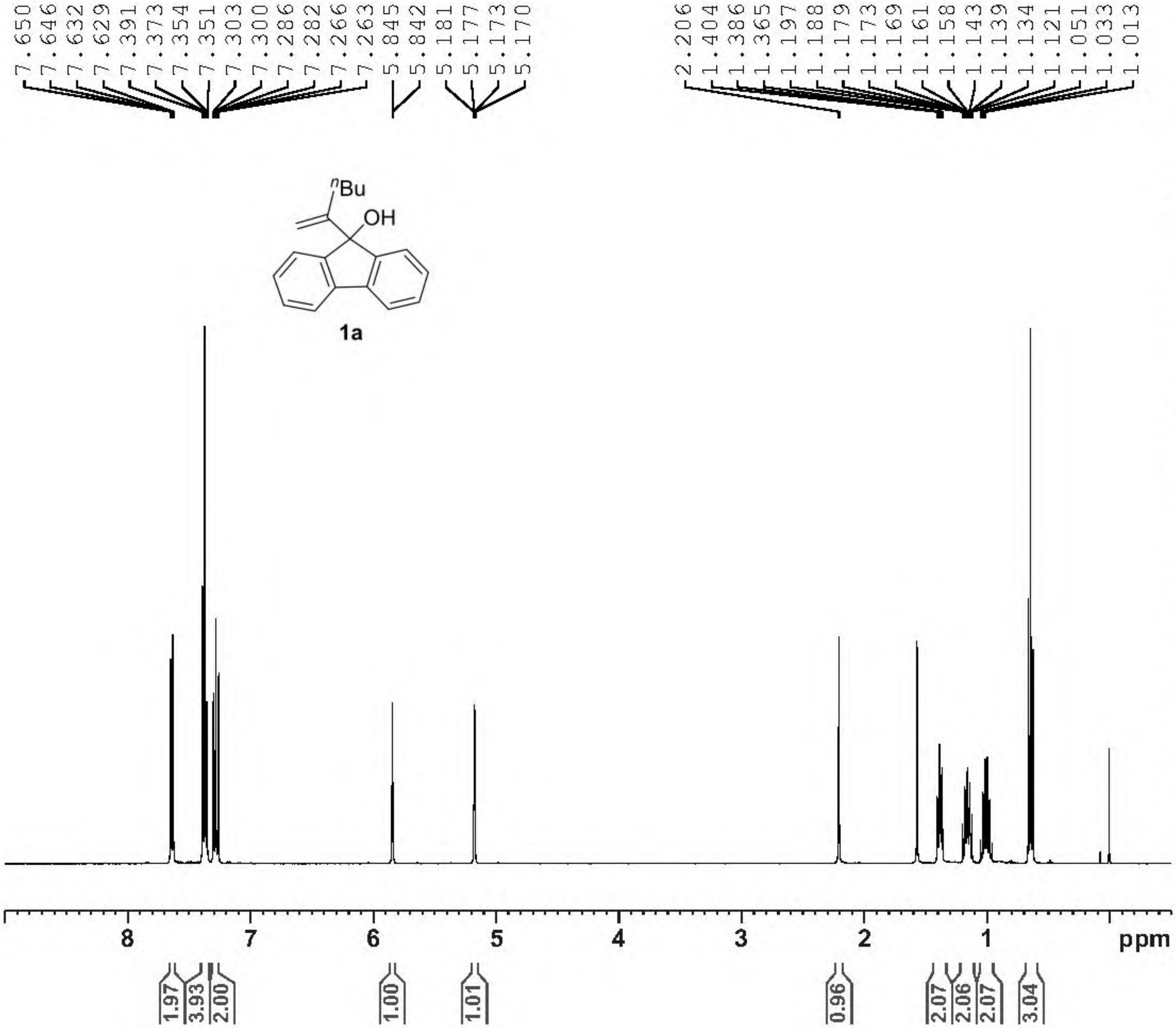

Current Data Parameters
NAME zzj-SM-Bu-H
EXPNO 1
PROCNO 1

F2 - Acquisition Parameters
Date_ 20240711
Time 0.16
INSTRUM spect
PROBHD 5 mm PABBO BB/
PULPROG zg30
TD 65536
SOLVENT CDCl3
NS 16
DS 2
SWH 8012.820 Hz
FIDRES 0.122266 Hz
AQ 4.0894465 sec
RG 88.84
DW 62.400 usec
DE 6.50 usec
TE 296.3 K
D1 1.00000000 sec
TD0 1

======== CHANNEL f1 ========
SFO1 400.1324710 MHz
NUC1 1H
P1 14.50 usec
PLW1 11.99499989 W

F2 - Processing parameters
SI 65536
SF 400.1300099 MHz
WDW EM
SSB 0
LB 0.30 Hz
GB 0
PC 1.00

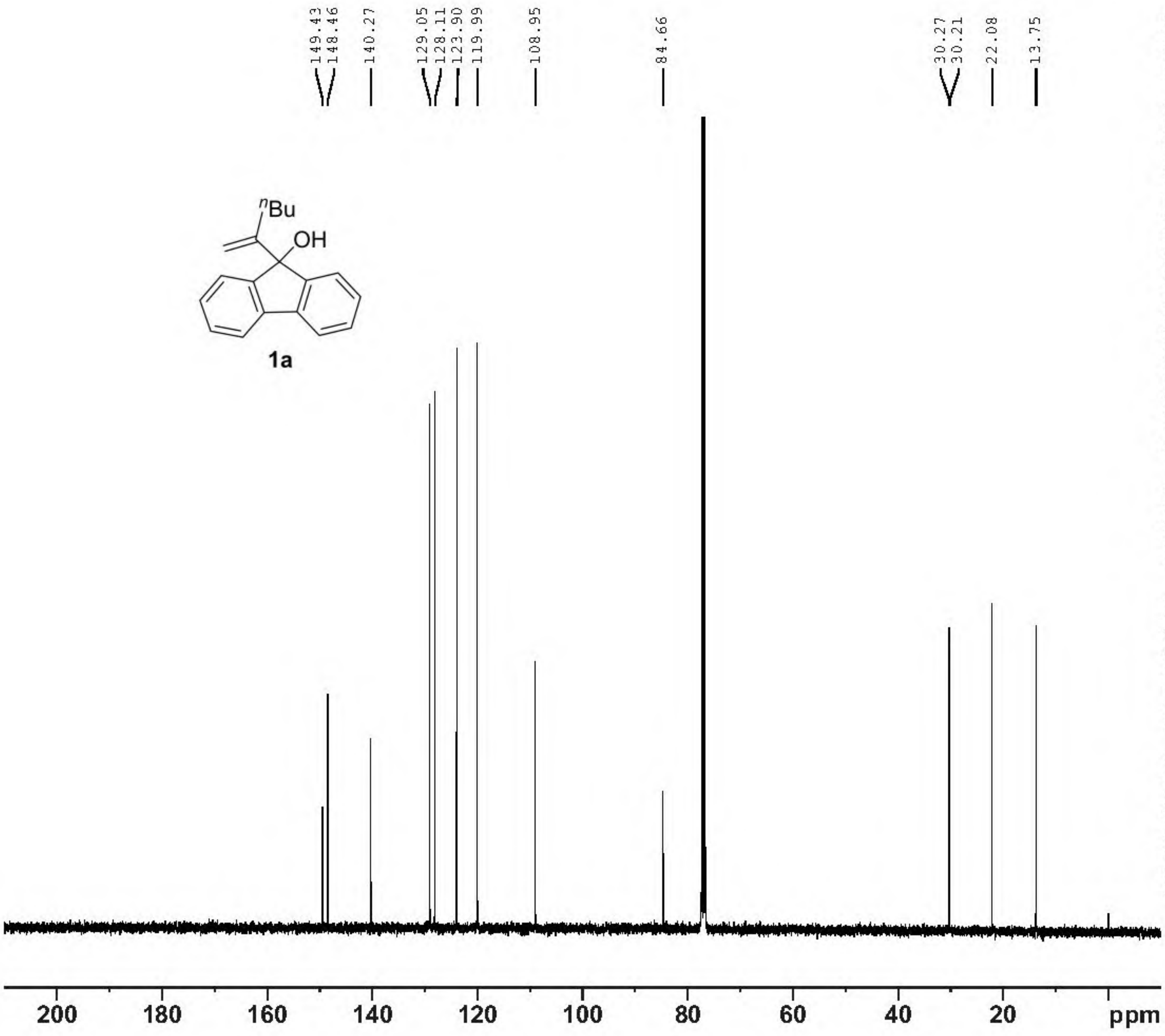


```
Current Data Parameters
NAME          zzj-SM-Bu-C
EXPNO                   1
PROCNO                  1

F2 - Acquisition Parameters
Date_            20240711
Time                 1.14
INSTRUM             spect
PROBHD   5 mm PABBO BB/
PULPROG           zgpg30
TD                 65536
SOLVENT            CDCl3
NS                  1000
DS                     2
SWH            24038.461 Hz
FIDRES          0.366798 Hz
AQ             1.3631488 sec
RG                196.92
DW                20.800 usec
DE                  6.50 usec
TE                 297.2 K
D1            2.00000000 sec
D11           0.03000000 sec
TD0                    1

======== CHANNEL f1 ========
SFO1         100.6228298 MHz
NUC1                 13C
P1                  9.70 usec
PLW1         46.98899841 W

======== CHANNEL f2 ========
SFO2         400.1316005 MHz
NUC2                  1H
CPDPRG[2          waltz16
PCPD2              90.00 usec
PLW2         11.99499989 W
PLW12         0.34213999 W
PLW13         0.27713001 W

F2 - Processing parameters
SI                 32768
SF           100.6127718 MHz
WDW                   EM
SSB                    0
LB                  1.00 Hz
GB                     0
PC                  1.40
```

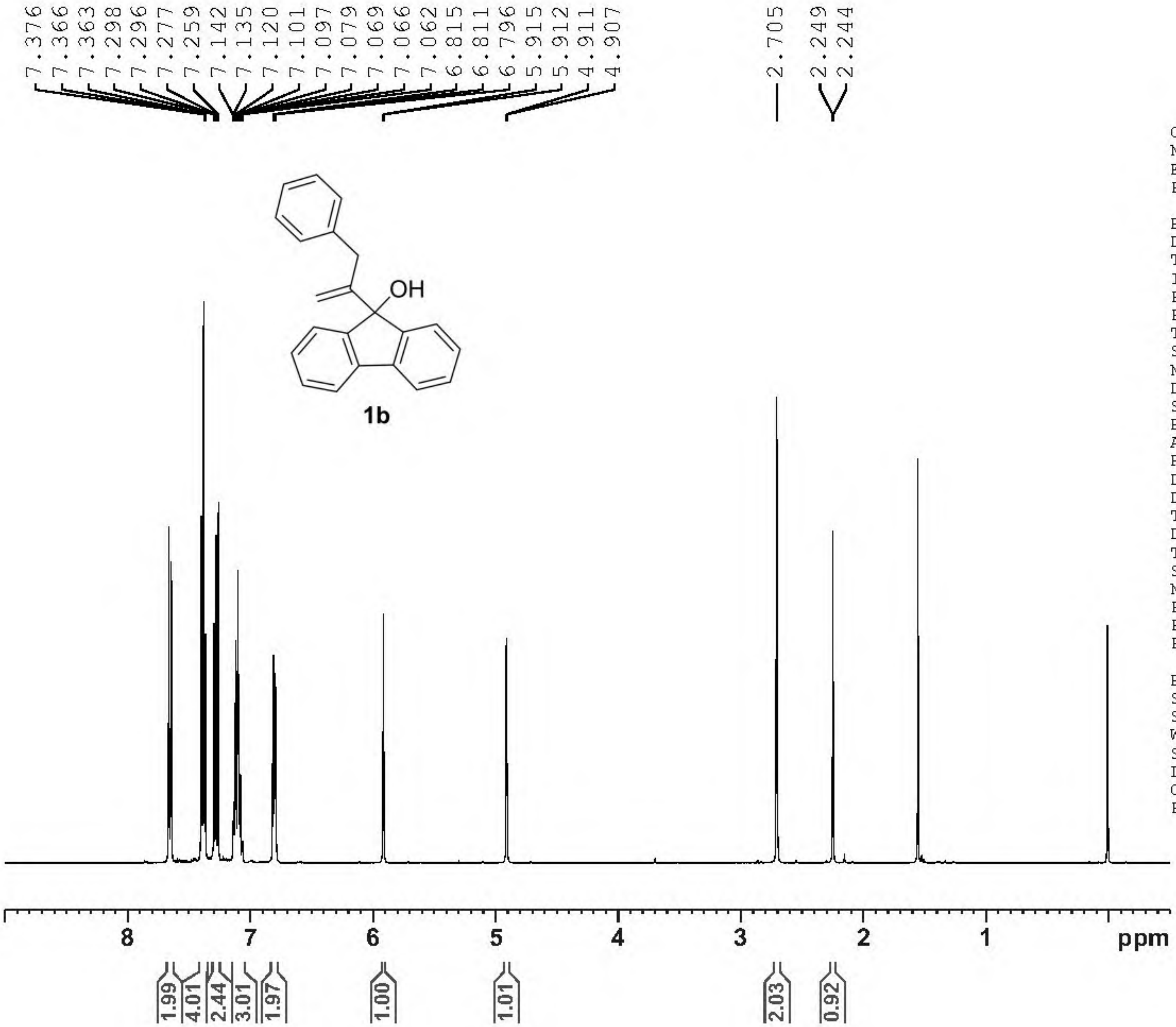

1b

Current Data Parameters
NAME SM-Bn-H
EXPNO 1
PROCNO 1

F2 - Acquisition Parameters
Date_ 20240109
Time_ 11.10 h
INSTRUM AvanceNeo 400MHz
PROBHD Z163739_0629 (
PULPROG zg30
TD 65536
SOLVENT CDCl3
NS 16
DS 2
SWH 8196.722 Hz
FIDRES 0.250144 Hz
AQ 3.9976959 sec
RG 101
DW 61.000 usec
DE 13.89 usec
TE 297.0 K
D1 1.00000000 sec
TD0 1
SFO1 400.1824711 MHz
NUC1 1H
P0 2.67 usec
P1 8.00 usec
PLW1 21.26700020 W

F2 - Processing parameters
SI 65536
SF 400.1800099 MHz
WDW EM
SSB 0
LB 0.30 Hz
GB 0
PC 1.00

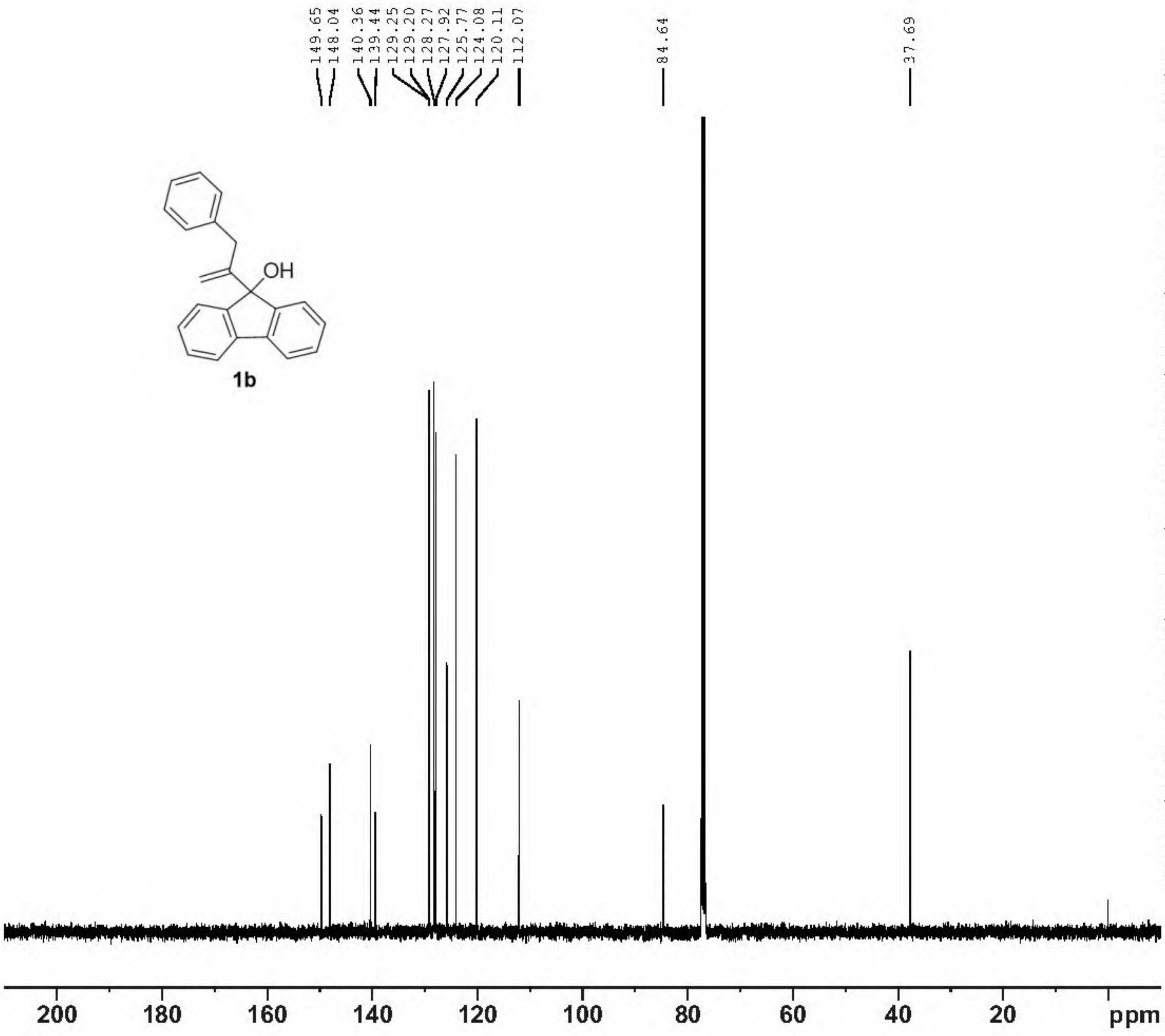


```
Current Data Parameters
NAME              SM-Bn-C
EXPNO                   3
PROCNO                  1

F2 - Acquisition Parameters
Date_            20240109
Time                12.09 h
INSTRUM  AvanceNeo 400MHz
PROBHD   Z163739_0629 (
PULPROG            zgpg30
TD                  65536
SOLVENT             CDCl3
NS                   1000
DS                      4
SWH             23809.523 Hz
FIDRES           0.726609 Hz
AQ              1.3762560 sec
RG                     10
DW                 21.000 usec
DE                   6.50 usec
TE                  297.6 K
D1             2.00000000 sec
D11            0.03000000 sec
TD0                     1
SFO1          100.6354036 MHz
NUC1                  13C
P0                   2.67 usec
P1                   8.00 usec
PLW1          85.25399780 W
SFO2          400.1816007 MHz
NUC2                   1H
CPDPRG[2          waltz65
PCPD2               90.00 usec
PLW2          21.26700020 W
PLW12          0.16802999 W
PLW13          0.08452000 W

F2 - Processing parameters
SI                  32768
SF            100.6253410 MHz
WDW                    EM
SSB                     0
LB                   1.00 Hz
GB                      0
PC                   1.40
```

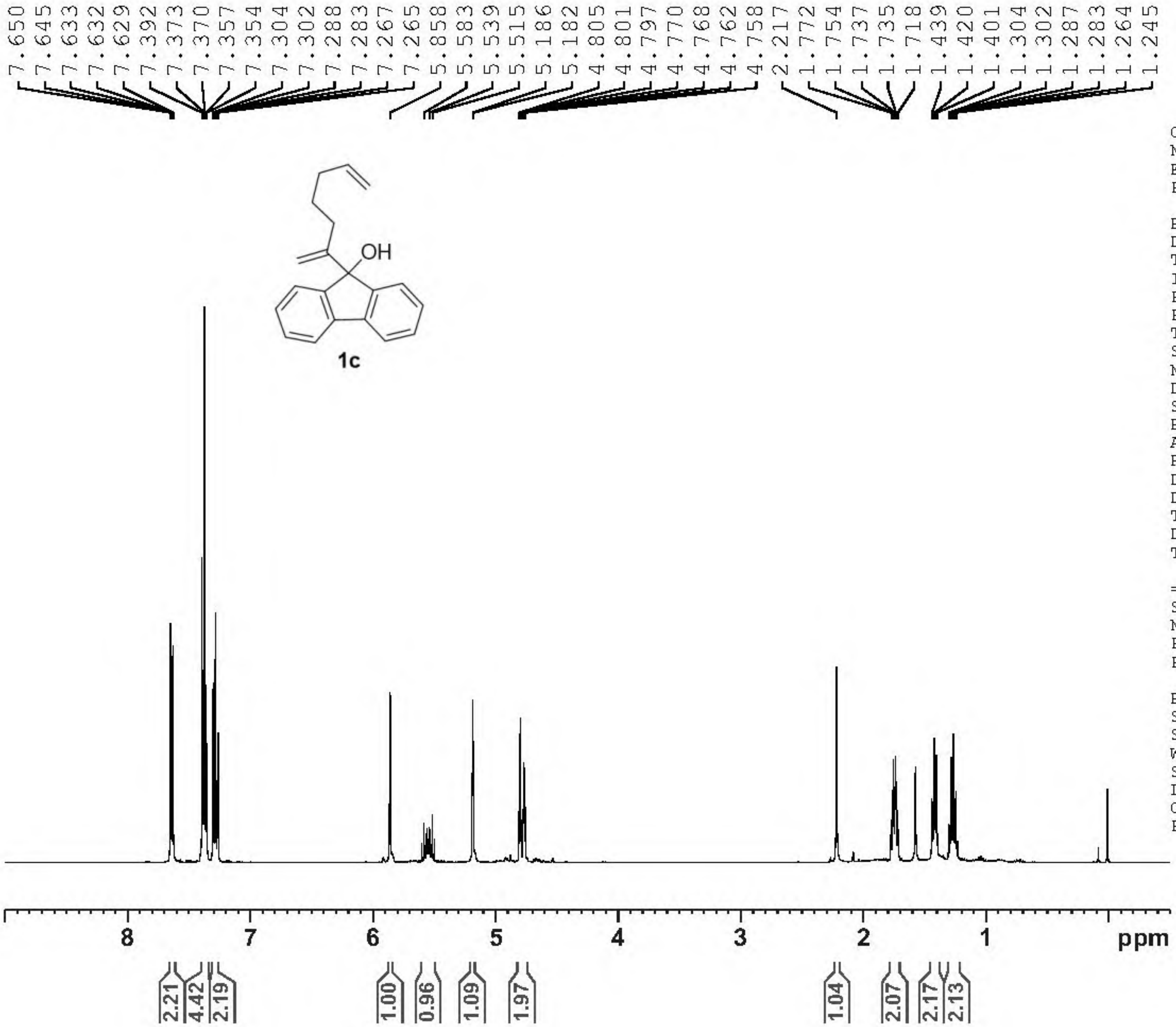


```
Current Data Parameters
NAME      zzj-SM-Alkene-H
EXPNO                   1
PROCNO                  1

F2 - Acquisition Parameters
Date_            20240711
Time                 3.23
INSTRUM             spect
PROBHD   5 mm PABBO BB/
PULPROG              zg30
TD                  65536
SOLVENT             CDCl3
NS                     16
DS                      2
SWH            8012.820 Hz
FIDRES         0.122266 Hz
AQ            4.0894465 sec
RG                  70.97
DW               62.400 usec
DE                 6.50 usec
TE                296.3 K
D1           1.00000000 sec
TD0                     1

======== CHANNEL f1 ========
SFO1        400.1324710 MHz
NUC1                   1H
P1                14.50 usec
PLW1        11.99499989 W

F2 - Processing parameters
SI                  65536
SF          400.1300098 MHz
WDW                    EM
SSB                     0
LB                   0.30 Hz
GB                      0
PC                   1.00
```

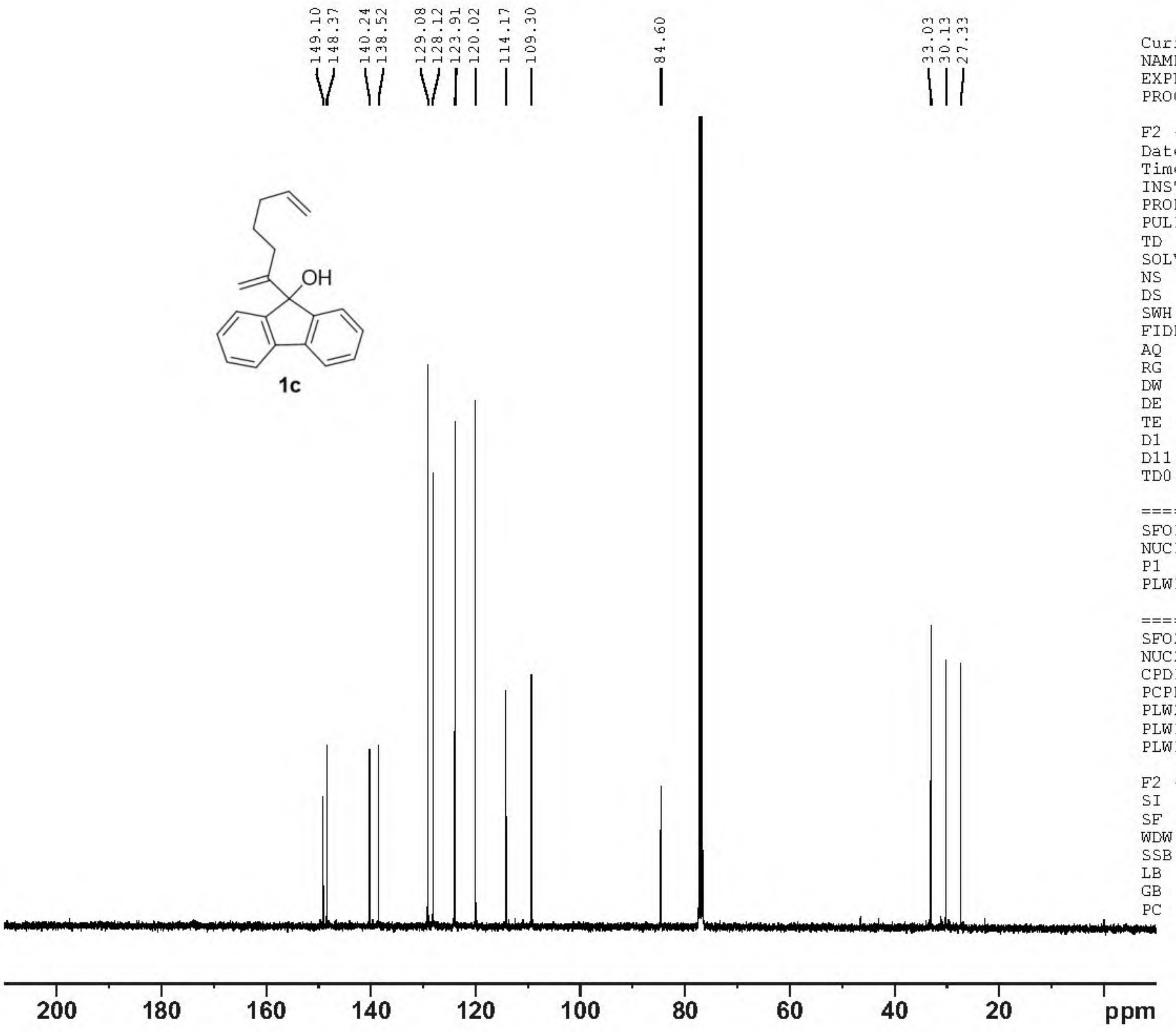


```
Current Data Parameters
NAME      zzj-SM-Alkene-C
EXPNO                   1
PROCNO                  1

F2 - Acquisition Parameters
Date_            20240711
Time                 4.21
INSTRUM             spect
PROBHD    5 mm PABBO BB/
PULPROG           zgpg30
TD                 65536
SOLVENT            CDCl3
NS                  1000
DS                     2
SWH            24038.461 Hz
FIDRES          0.366798 Hz
AQ             1.3631488 sec
RG                196.92
DW                20.800 usec
DE                  6.50 usec
TE                 297.2 K
D1            2.00000000 sec
D11           0.03000000 sec
TD0                    1

======== CHANNEL f1 ========
SFO1         100.6228298 MHz
NUC1                 13C
P1                  9.70 usec
PLW1         46.98899841 W

======== CHANNEL f2 ========
SFO2         400.1316005 MHz
NUC2                  1H
CPDPRG[2         waltz16
PCPD2              90.00 usec
PLW2         11.99499989 W
PLW12         0.34213999 W
PLW13         0.27713001 W

F2 - Processing parameters
SI                 32768
SF           100.6127728 MHz
WDW                   EM
SSB                    0
LB                  1.00 Hz
GB                     0
PC                  1.40
```

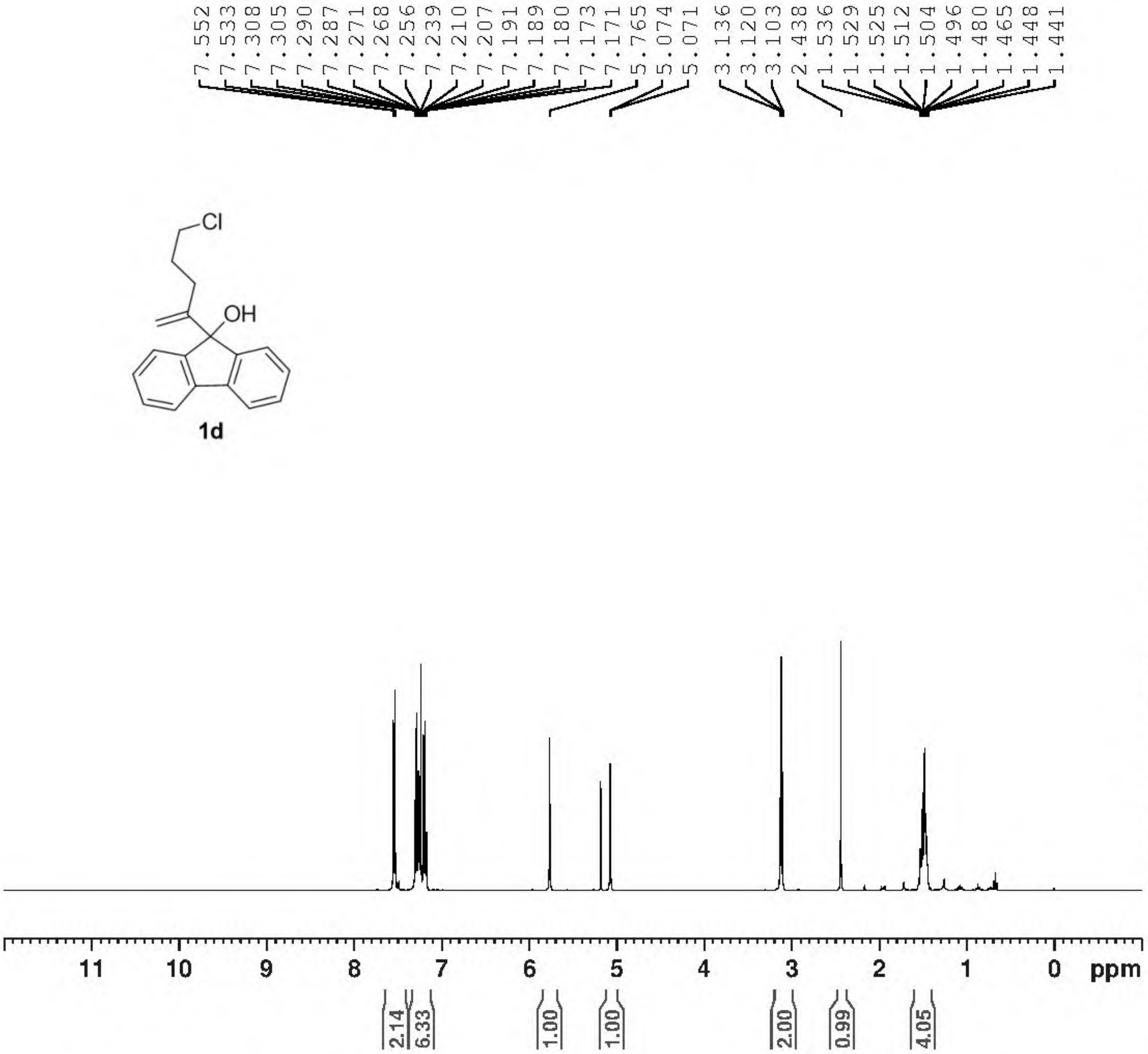

Current Data Parameters	
NAME	yql-5-113
EXPNO	1
PROCNO	1
F2 - Acquisition Parameters	
Date_	20230318
Time	15.12
INSTRUM	spect
PROBHD	5 mm DUL 13C-1
PULPROG	zg30
TD	65536
SOLVENT	CDCl3
NS	3
DS	0
SWH	8223.685 Hz
FIDRES	0.125483 Hz
AQ	3.9845889 sec
RG	64
DW	60.800 usec
DE	6.00 usec
TE	292.8 K
D1	1.00000000 sec
TD0	1
======== CHANNEL f1 ========	
NUC1	1H
P1	15.80 usec
PL1	-1.00 dB
PL1W	12.17476940 W
SFO1	400.1324710 MHz
F2 - Processing parameters	
SI	32768
SF	400.1300415 MHz
WDW	EM
SSB	0
LB	0.30 Hz
GB	0
PC	1.00

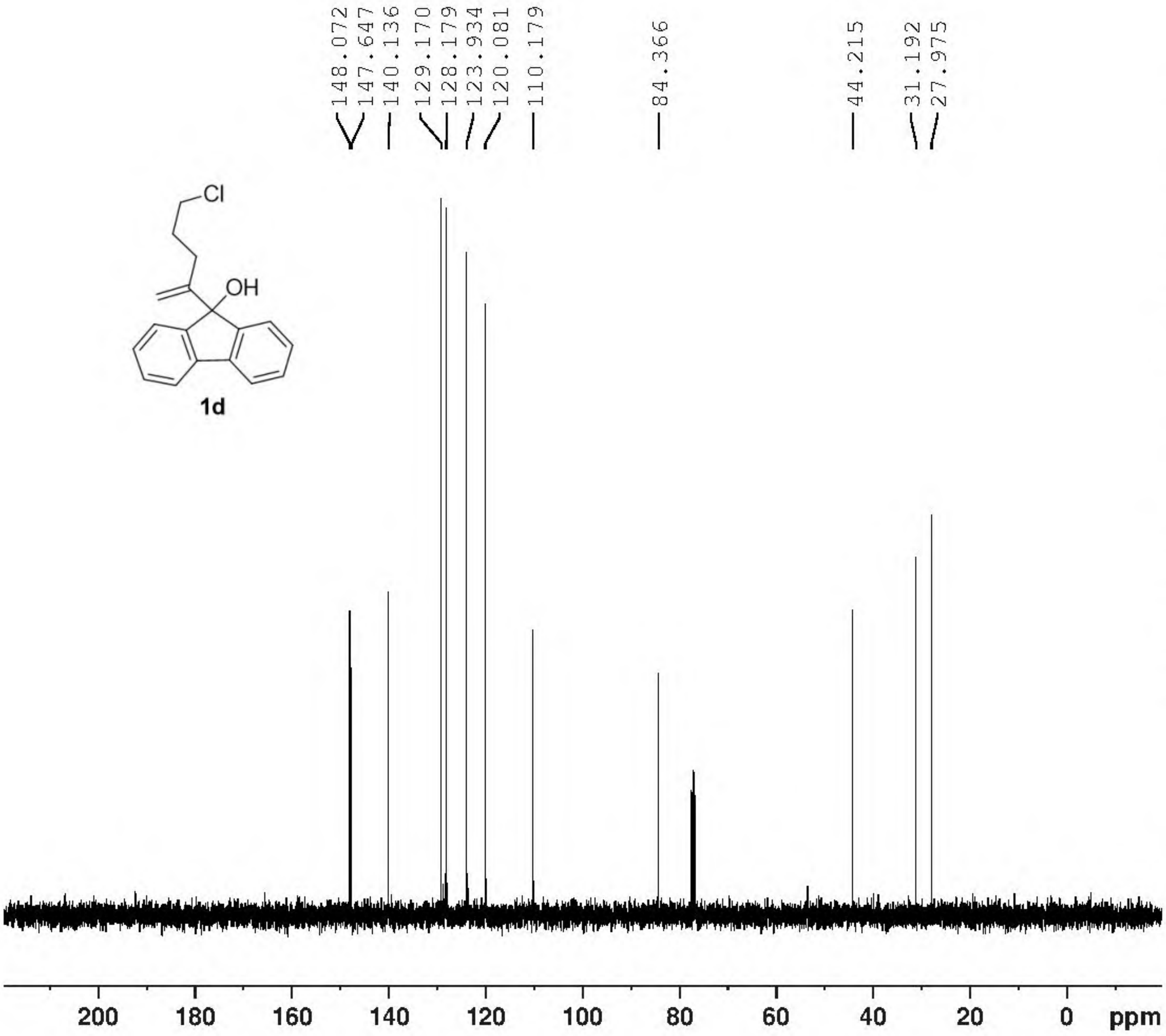


```
Current Data Parameters
NAME            yql-5-113
EXPNO                   2
PROCNO                  1

F2 - Acquisition Parameters
Date_            20230318
Time                15.22
INSTRUM             spect
PROBHD   5 mm DUL 13C-1
PULPROG           zgpg30
TD                  65536
SOLVENT             CDCl3
NS                     10
DS                      1
SWH             24038.461 Hz
FIDRES           0.366798 Hz
AQ              1.3631488 sec
RG                   2050
DW                 20.800 usec
DE                   6.00 usec
TE                  292.9 K
D1             2.00000000 sec
D11            0.03000000 sec
TD0                     1

======== CHANNEL f1 ========
NUC1                  13C
P1                  40.00 usec
PL1                 -3.00 dB
PL1W          60.64365387 W
SFO1          100.6228298 MHz

======== CHANNEL f2 ========
CPDPRG[2          waltz16
NUC2                   1H
PCPD2               80.00 usec
PL2                 -1.00 dB
PL12                14.39 dB
PL13                18.00 dB
PL2W          12.17476940 W
PL12W          0.35193357 W
PL13W          0.15327126 W
SFO2          400.1316005 MHz

F2 - Processing parameters
SI                  32768
SF            100.6127786 MHz
WDW                    EM
SSB                     0
LB                   1.00 Hz
GB                      0
PC                   1.40
```

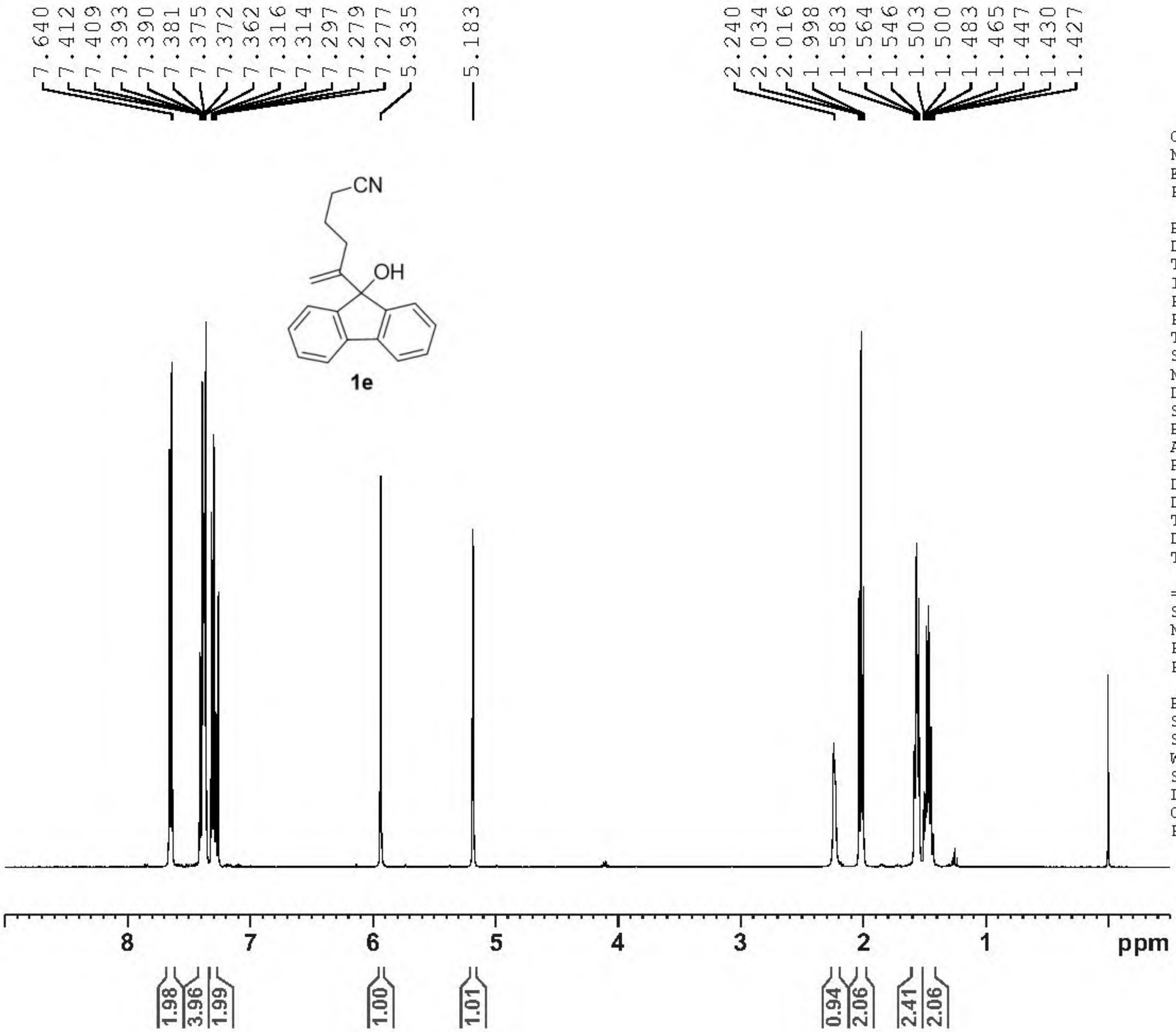


```
Current Data Parameters
NAME          zzj-SM-CN-H
EXPNO                   1
PROCNO                  1

F2 - Acquisition Parameters
Date_            20240723
Time                 4.09
INSTRUM             spect
PROBHD   5 mm PABBO BB/
PULPROG              zg30
TD                  65536
SOLVENT             CDCl3
NS                     16
DS                      2
SWH              8012.820 Hz
FIDRES           0.122266 Hz
AQ              4.0894465 sec
RG                 103.52
DW                 62.400 usec
DE                   6.50 usec
TE                  296.4 K
D1             1.00000000 sec
TD0                     1

======== CHANNEL f1 ========
SFO1         400.1324710 MHz
NUC1                   1H
P1                  14.50 usec
PLW1          11.99499989 W

F2 - Processing parameters
SI                  65536
SF           400.1300099 MHz
WDW                    EM
SSB                     0
LB                   0.30 Hz
GB                      0
PC                   1.00
```

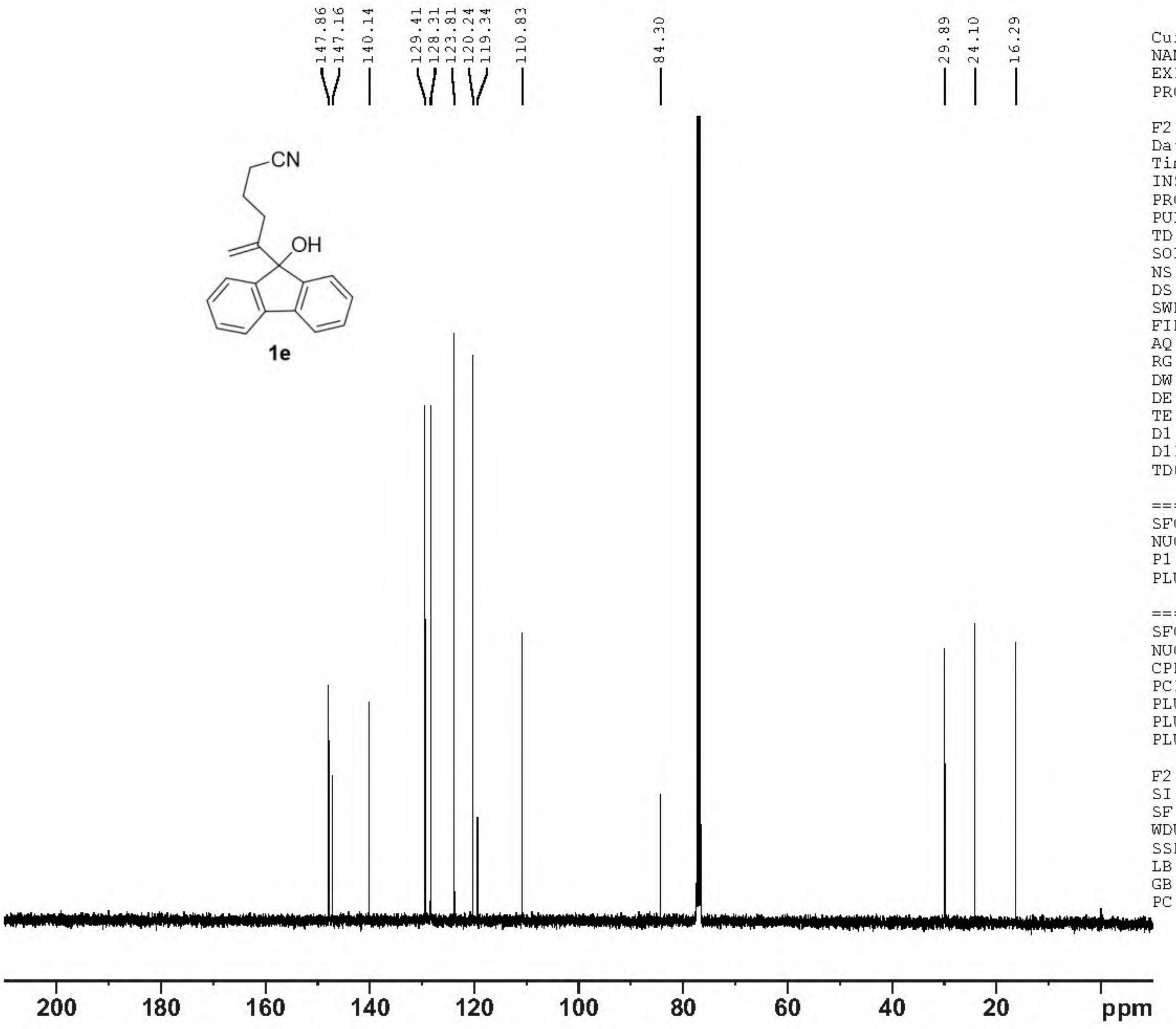

Current Data Parameters
NAME zzj-SM-CN-C
EXPNO 1
PROCNO 1

F2 - Acquisition Parameters
Date_ 20240723
Time_ 4.56
INSTRUM spect
PROBHD 5 mm PABBO BB/
PULPROG zgpg30
TD 65536
SOLVENT CDCl3
NS 800
DS 2
SWH 24038.461 Hz
FIDRES 0.366798 Hz
AQ 1.3631488 sec
RG 196.92
DW 20.800 usec
DE 6.50 usec
TE 297.4 K
D1 2.00000000 sec
D11 0.03000000 sec
TD0 1

======== CHANNEL f1 ========
SFO1 100.6228298 MHz
NUC1 13C
P1 9.70 usec
PLW1 46.98899841 W

======== CHANNEL f2 ========
SFO2 400.1316005 MHz
NUC2 1H
CPDPRG[2 waltz16
PCPD2 90.00 usec
PLW2 11.99499989 W
PLW12 0.34213999 W
PLW13 0.27713001 W

F2 - Processing parameters
SI 32768
SF 100.6127733 MHz
WDW EM
SSB 0
LB 1.00 Hz
GB 0
PC 1.40

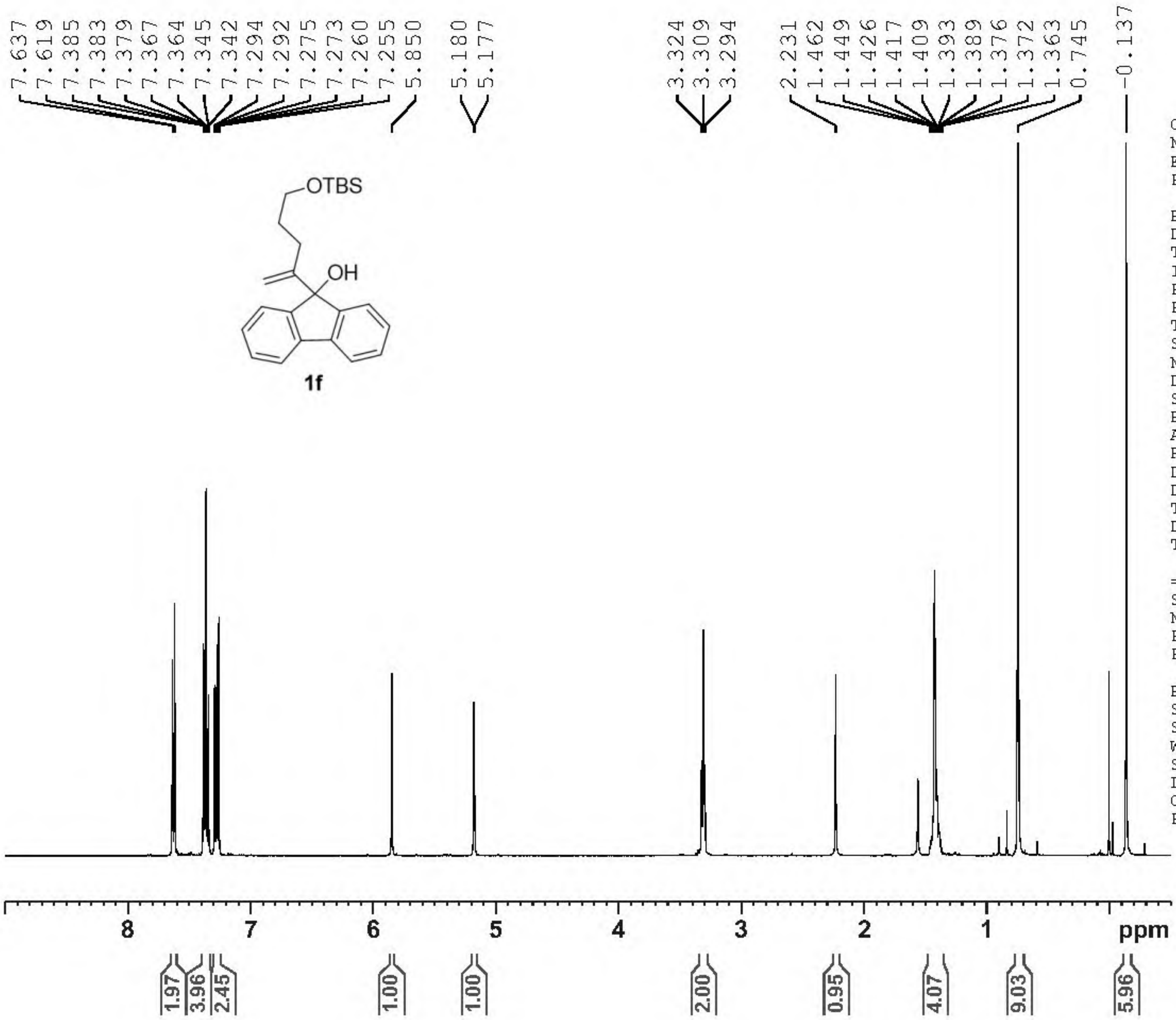


```
Current Data Parameters
NAME        zzj-SM-OTBS-H
EXPNO                   1
PROCNO                  1

F2 - Acquisition Parameters
Date_            20240723
Time                 3.18
INSTRUM             spect
PROBHD    5 mm PABBO BB/
PULPROG              zg30
TD                  65536
SOLVENT             CDCl3
NS                     16
DS                      2
SWH             8012.820 Hz
FIDRES          0.122266 Hz
AQ             4.0894465 sec
RG                  88.84
DW                 62.400 usec
DE                   6.50 usec
TE                  296.1 K
D1             1.00000000 sec
TD0                     1

======== CHANNEL f1 ========
SFO1         400.1324710 MHz
NUC1                   1H
P1                  14.50 usec
PLW1          11.99499989 W

F2 - Processing parameters
SI                  65536
SF           400.1300097 MHz
WDW                    EM
SSB                     0
LB                   0.30 Hz
GB                      0
PC                   1.00
```

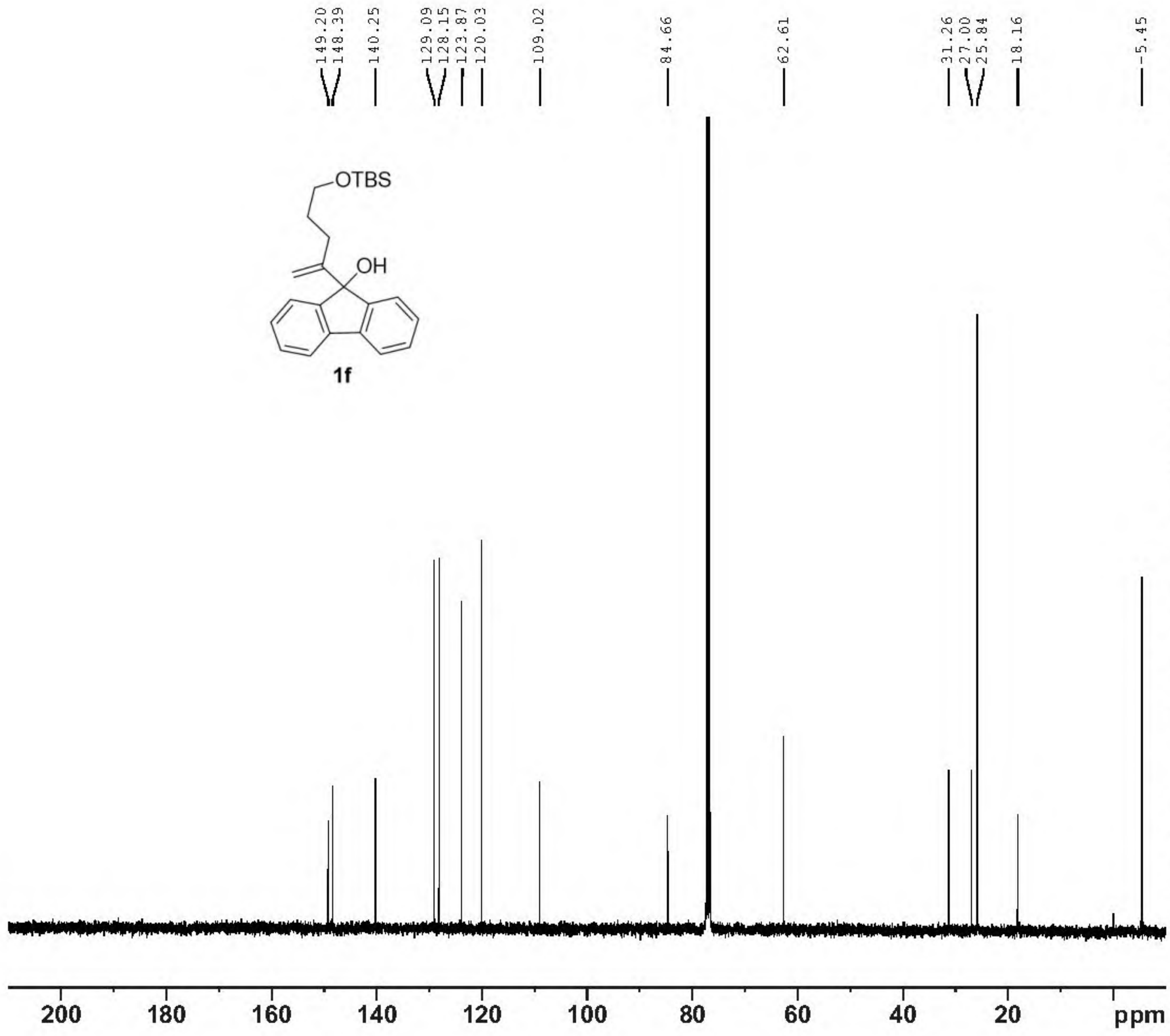

Current Data Parameters
NAME zzj-SM-OTBS-C
EXPNO 1
PROCNO 1

F2 - Acquisition Parameters
Date_ 20240723
Time_ 4.05
INSTRUM spect
PROBHD 5 mm PABBO BB/
PULPROG zgpg30
TD 65536
SOLVENT CDCl3
NS 800
DS 2
SWH 24038.461 Hz
FIDRES 0.366798 Hz
AQ 1.3631488 sec
RG 196.92
DW 20.800 usec
DE 6.50 usec
TE 297.3 K
D1 2.00000000 sec
D11 0.03000000 sec
TD0 1

======== CHANNEL f1 ========
SFO1 100.6228298 MHz
NUC1 13C
P1 9.70 usec
PLW1 46.98899841 W

======== CHANNEL f2 ========
SFO2 400.1316005 MHz
NUC2 1H
CPDPRG[2 waltz16
PCPD2 90.00 usec
PLW2 11.99499989 W
PLW12 0.34213999 W
PLW13 0.27713001 W

F2 - Processing parameters
SI 32768
SF 100.6127712 MHz
WDW EM
SSB 0
LB 1.00 Hz
GB 0
PC 1.40

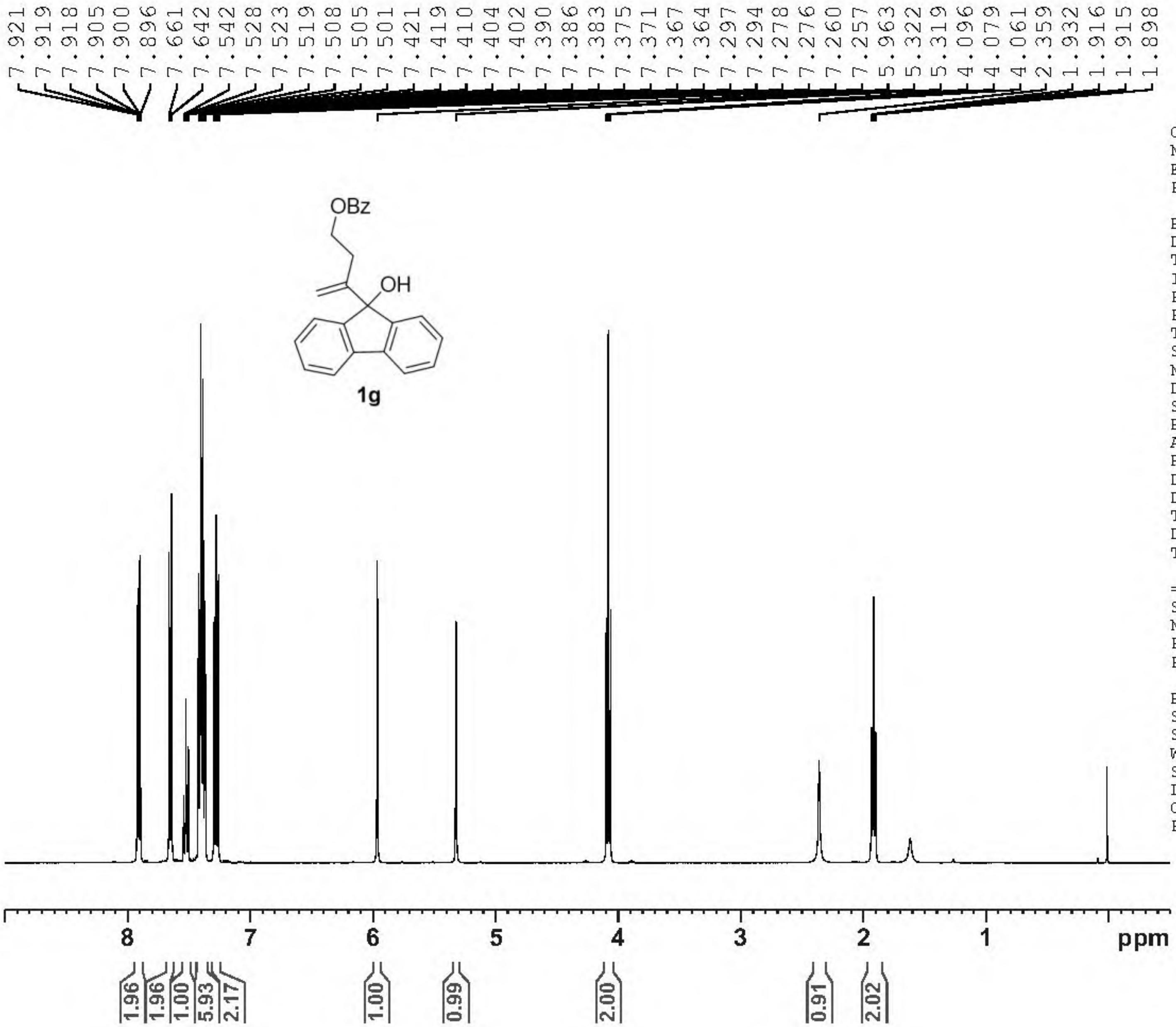


```
Current Data Parameters
NAME          zzj-SM-OBz-H
EXPNO                    1
PROCNO                   1

F2 - Acquisition Parameters
Date_             20240711
Time                  4.26
INSTRUM              spect
PROBHD   5 mm PABBO BB/
PULPROG               zg30
TD                   65536
SOLVENT              CDCl3
NS                      16
DS                       2
SWH              8012.820 Hz
FIDRES           0.122266 Hz
AQ              4.0894465 sec
RG                   70.97
DW                  62.400 usec
DE                    6.50 usec
TE                   296.3 K
D1              1.00000000 sec
TD0                      1

======== CHANNEL f1 ========
SFO1          400.1324710 MHz
NUC1                    1H
P1                   14.50 usec
PLW1          11.99499989 W

F2 - Processing parameters
SI                   65536
SF            400.1300097 MHz
WDW                     EM
SSB                      0
LB                    0.30 Hz
GB                       0
PC                    1.00
```

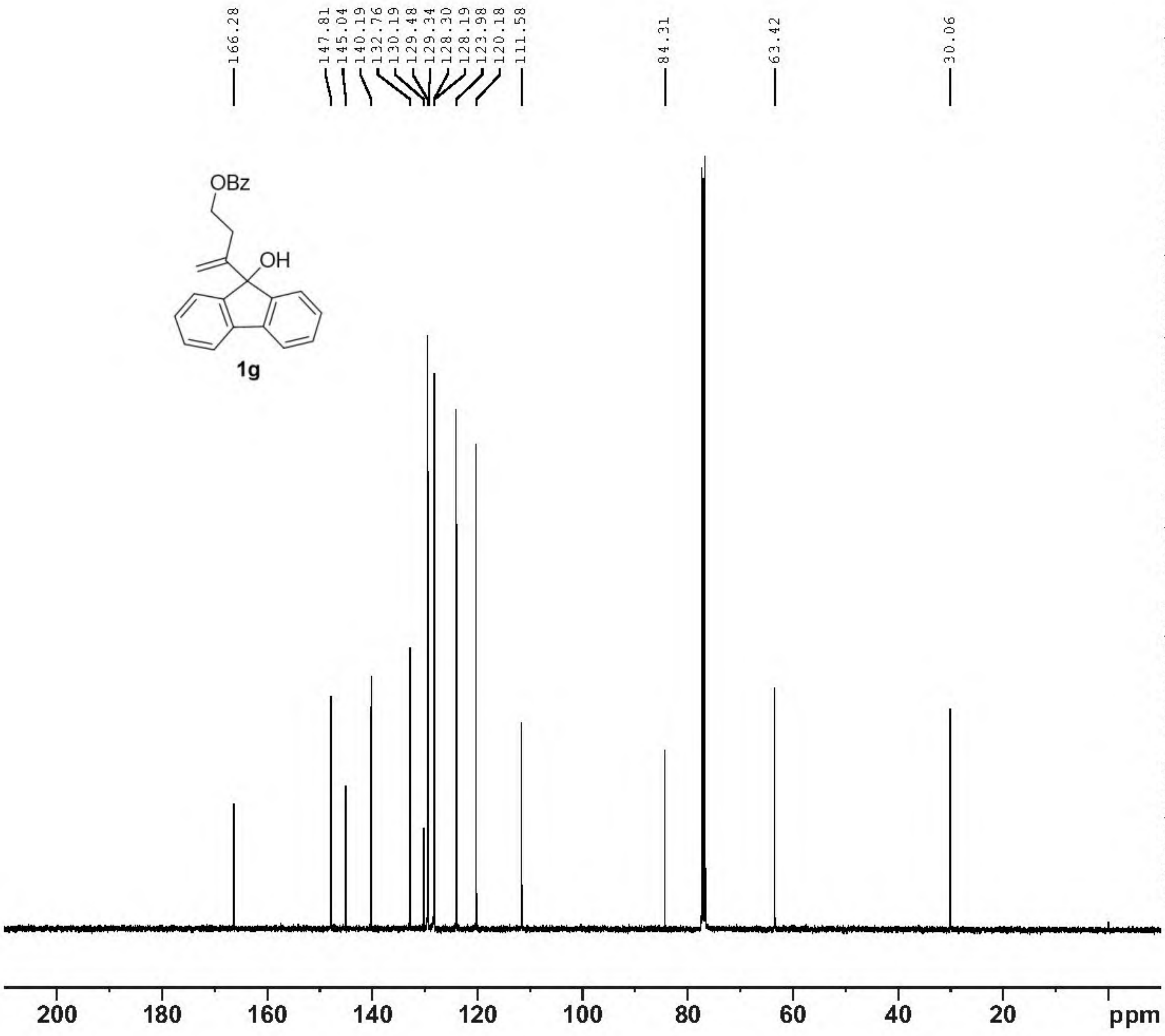


```
Current Data Parameters
NAME        zzj-SM-OBz-C
EXPNO                  1
PROCNO                 1

F2 - Acquisition Parameters
Date_           20240711
Time                5.25
INSTRUM            spect
PROBHD   5 mm PABBO BB/
PULPROG          zgpg30
TD                 65536
SOLVENT            CDCl3
NS                  1000
DS                     2
SWH            24038.461 Hz
FIDRES          0.366798 Hz
AQ             1.3631488 sec
RG                196.92
DW                20.800 usec
DE                  6.50 usec
TE                 297.2 K
D1            2.00000000 sec
D11           0.03000000 sec
TD0                    1

======== CHANNEL f1 ========
SFO1         100.6228298 MHz
NUC1                 13C
P1                  9.70 usec
PLW1         46.98899841 W

======== CHANNEL f2 ========
SFO2         400.1316005 MHz
NUC2                  1H
CPDPRG[2         waltz16
PCPD2              90.00 usec
PLW2         11.99499989 W
PLW12         0.34213999 W
PLW13         0.27713001 W

F2 - Processing parameters
SI                 32768
SF           100.6127744 MHz
WDW                   EM
SSB                    0
LB                  1.00 Hz
GB                     0
PC                  1.40
```

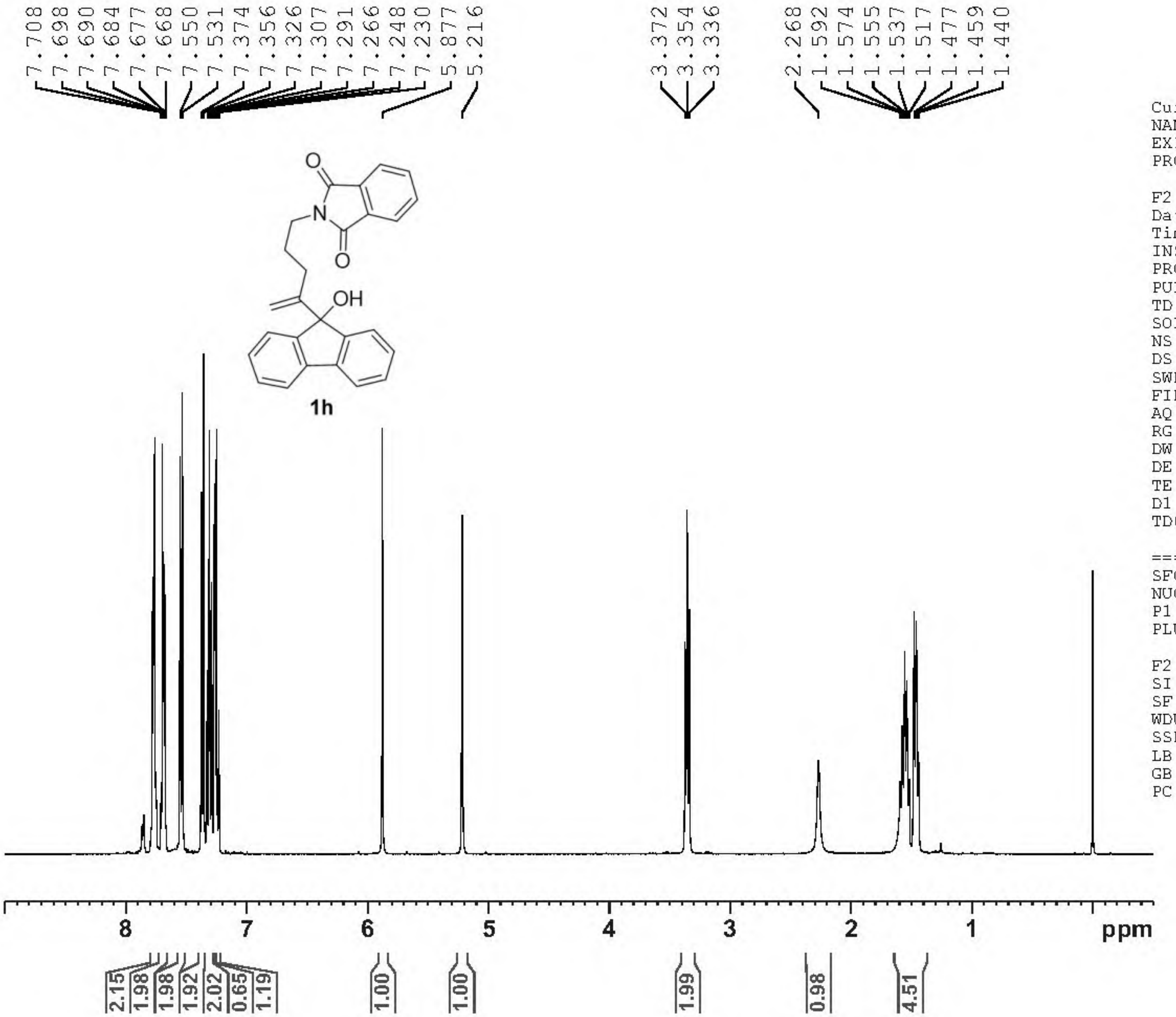


```
Current Data Parameters
NAME      zzj-SM-Nthph-H
EXPNO                  1
PROCNO                 1

F2 - Acquisition Parameters
Date_           20240723
Time                5.51
INSTRUM            spect
PROBHD   5 mm PABBO BB/
PULPROG             zg30
TD                 65536
SOLVENT            CDCl3
NS                    16
DS                     2
SWH            8012.820 Hz
FIDRES         0.122266 Hz
AQ            4.0894465 sec
RG                112.31
DW                62.400 usec
DE                  6.50 usec
TE                 296.5 K
D1            1.00000000 sec
TD0                    1

======== CHANNEL f1 ========
SFO1        400.1324710 MHz
NUC1                  1H
P1                 14.50 usec
PLW1         11.99499989 W

F2 - Processing parameters
SI                 65536
SF           400.1300097 MHz
WDW                   EM
SSB                    0
LB                  0.30 Hz
GB                     0
PC                  1.00
```

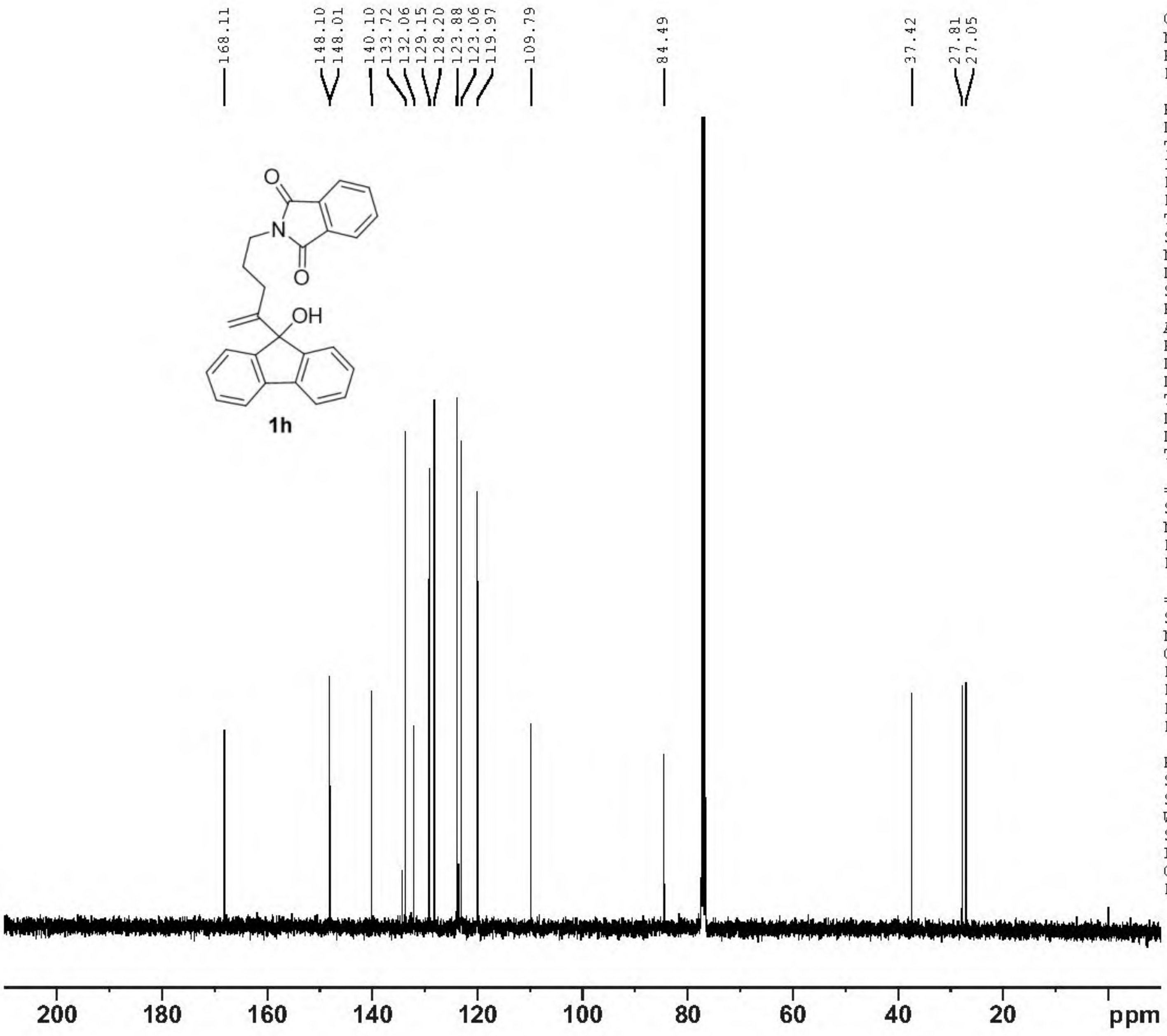


```
Current Data Parameters
NAME       zzj-SM-Nthph-H
EXPNO                   2
PROCNO                  1

F2 - Acquisition Parameters
Date_            20240723
Time                 6.38
INSTRUM             spect
PROBHD    5 mm PABBO BB/
PULPROG           zgpg30
TD                 65536
SOLVENT            CDCl3
NS                   800
DS                     2
SWH            24038.461 Hz
FIDRES          0.366798 Hz
AQ             1.3631488 sec
RG                196.92
DW                20.800 usec
DE                  6.50 usec
TE                 297.4 K
D1            2.00000000 sec
D11           0.03000000 sec
TD0                    1

======== CHANNEL f1 ========
SFO1         100.6228298 MHz
NUC1                 13C
P1                  9.70 usec
PLW1         46.98899841 W

======== CHANNEL f2 ========
SFO2         400.1316005 MHz
NUC2                  1H
CPDPRG[2         waltz16
PCPD2              90.00 usec
PLW2         11.99499989 W
PLW12         0.34213999 W
PLW13         0.27713001 W

F2 - Processing parameters
SI                 32768
SF           100.6127724 MHz
WDW                   EM
SSB                    0
LB                  1.00 Hz
GB                     0
PC                  1.40
```

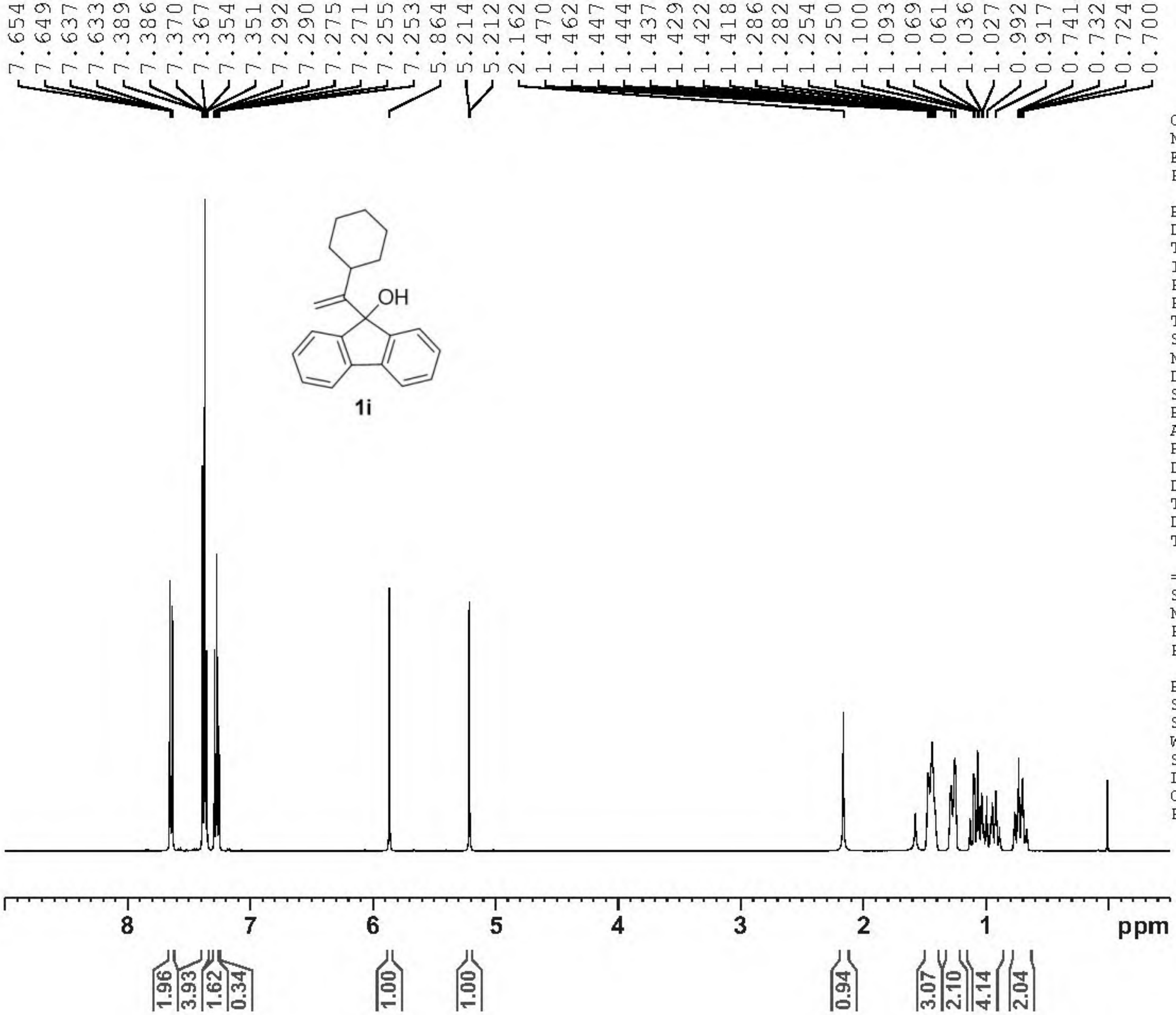


```
Current Data Parameters
NAME          zzj-SM-Cy-H
EXPNO                   1
PROCNO                  1

F2 - Acquisition Parameters
Date_            20240711
Time                 2.21
INSTRUM             spect
PROBHD   5 mm PABBO BB/
PULPROG              zg30
TD                  65536
SOLVENT             CDCl3
NS                     16
DS                      2
SWH              8012.820 Hz
FIDRES           0.122266 Hz
AQ              4.0894465 sec
RG                  62.93
DW                 62.400 usec
DE                   6.50 usec
TE                  296.3 K
D1             1.00000000 sec
TD0                     1

======== CHANNEL f1 ========
SFO1          400.1324710 MHz
NUC1                   1H
P1                  14.50 usec
PLW1          11.99499989 W

F2 - Processing parameters
SI                  65536
SF            400.1300098 MHz
WDW                    EM
SSB                     0
LB                   0.30 Hz
GB                      0
PC                   1.00
```

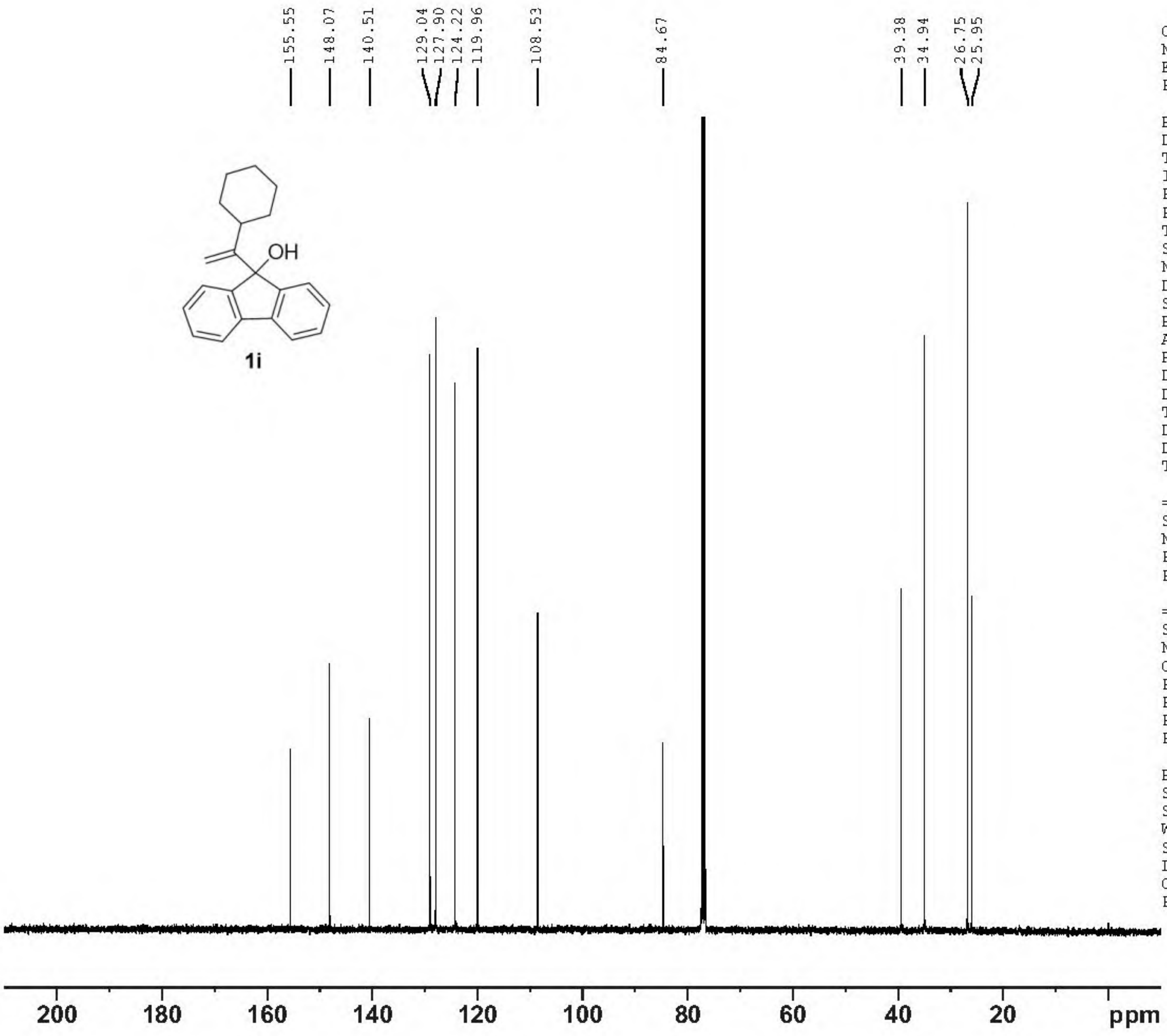

Current Data Parameters
NAME zzj-SM-Cy-C
EXPNO 1
PROCNO 1

F2 - Acquisition Parameters
Date_ 20240711
Time 3.19
INSTRUM spect
PROBHD 5 mm PABBO BB/
PULPROG zgpg30
TD 65536
SOLVENT CDCl3
NS 1000
DS 2
SWH 24038.461 Hz
FIDRES 0.366798 Hz
AQ 1.3631488 sec
RG 196.92
DW 20.800 usec
DE 6.50 usec
TE 297.2 K
D1 2.00000000 sec
D11 0.03000000 sec
TD0 1

======== CHANNEL f1 ========
SFO1 100.6228298 MHz
NUC1 13C
P1 9.70 usec
PLW1 46.98899841 W

======== CHANNEL f2 ========
SFO2 400.1316005 MHz
NUC2 1H
CPDPRG[2 waltz16
PCPD2 90.00 usec
PLW2 11.99499989 W
PLW12 0.34213999 W
PLW13 0.27713001 W

F2 - Processing parameters
SI 32768
SF 100.6127730 MHz
WDW EM
SSB 0
LB 1.00 Hz
GB 0
PC 1.40

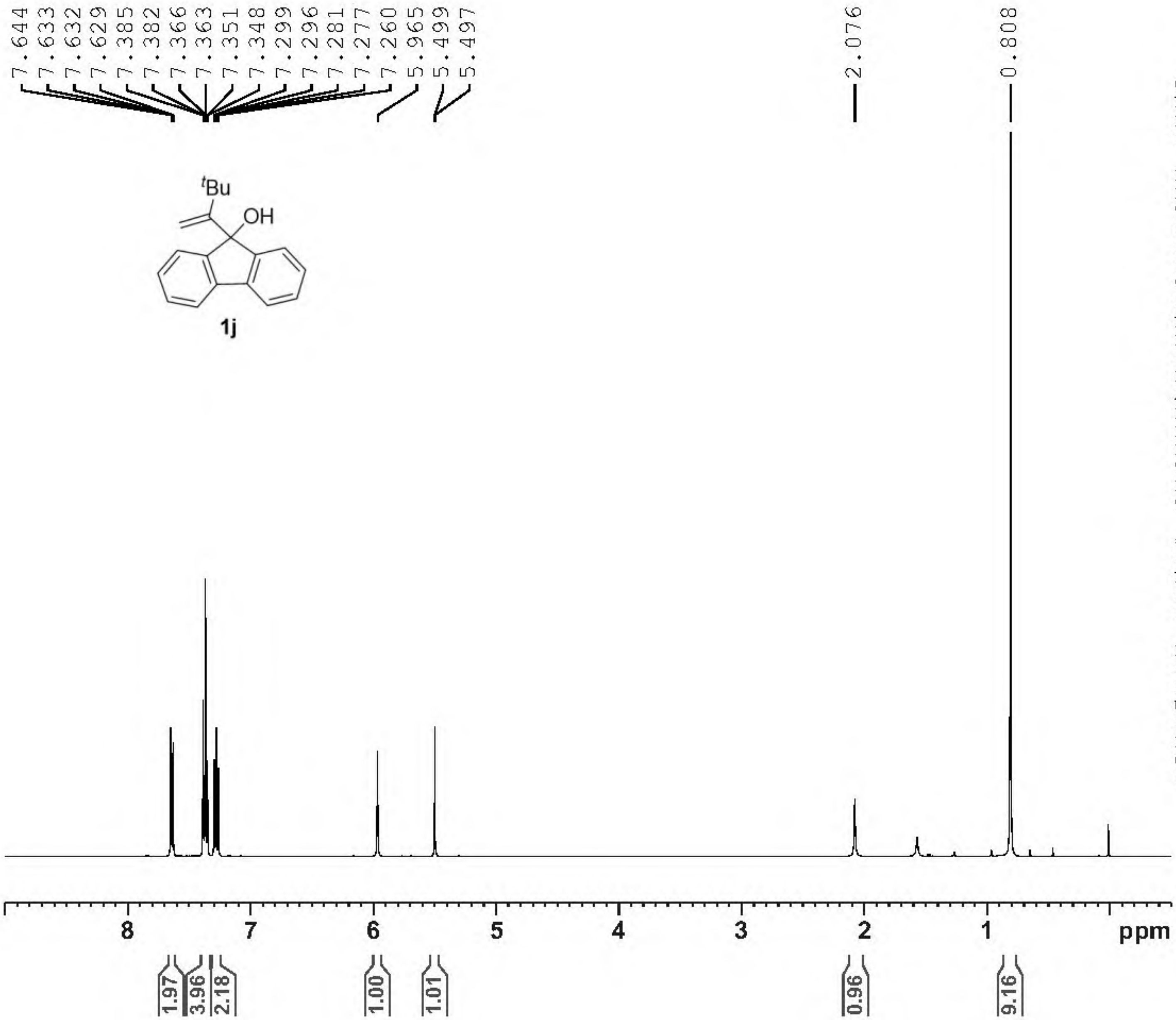


```
Current Data Parameters
NAME         zzj-SM-tBu-H
EXPNO                   1
PROCNO                  1

F2 - Acquisition Parameters
Date_            20240711
Time                 1.19
INSTRUM             spect
PROBHD   5 mm PABBO BB/
PULPROG              zg30
TD                  65536
SOLVENT             CDCl3
NS                     16
DS                      2
SWH            8012.820 Hz
FIDRES         0.122266 Hz
AQ            4.0894465 sec
RG                  70.97
DW               62.400 usec
DE                 6.50 usec
TE                296.3 K
D1           1.00000000 sec
TD0                     1

======== CHANNEL f1 ========
SFO1        400.1324710 MHz
NUC1                   1H
P1                14.50 usec
PLW1        11.99499989 W

F2 - Processing parameters
SI                  65536
SF          400.1300098 MHz
WDW                    EM
SSB                     0
LB                  0.30 Hz
GB                      0
PC                   1.00
```

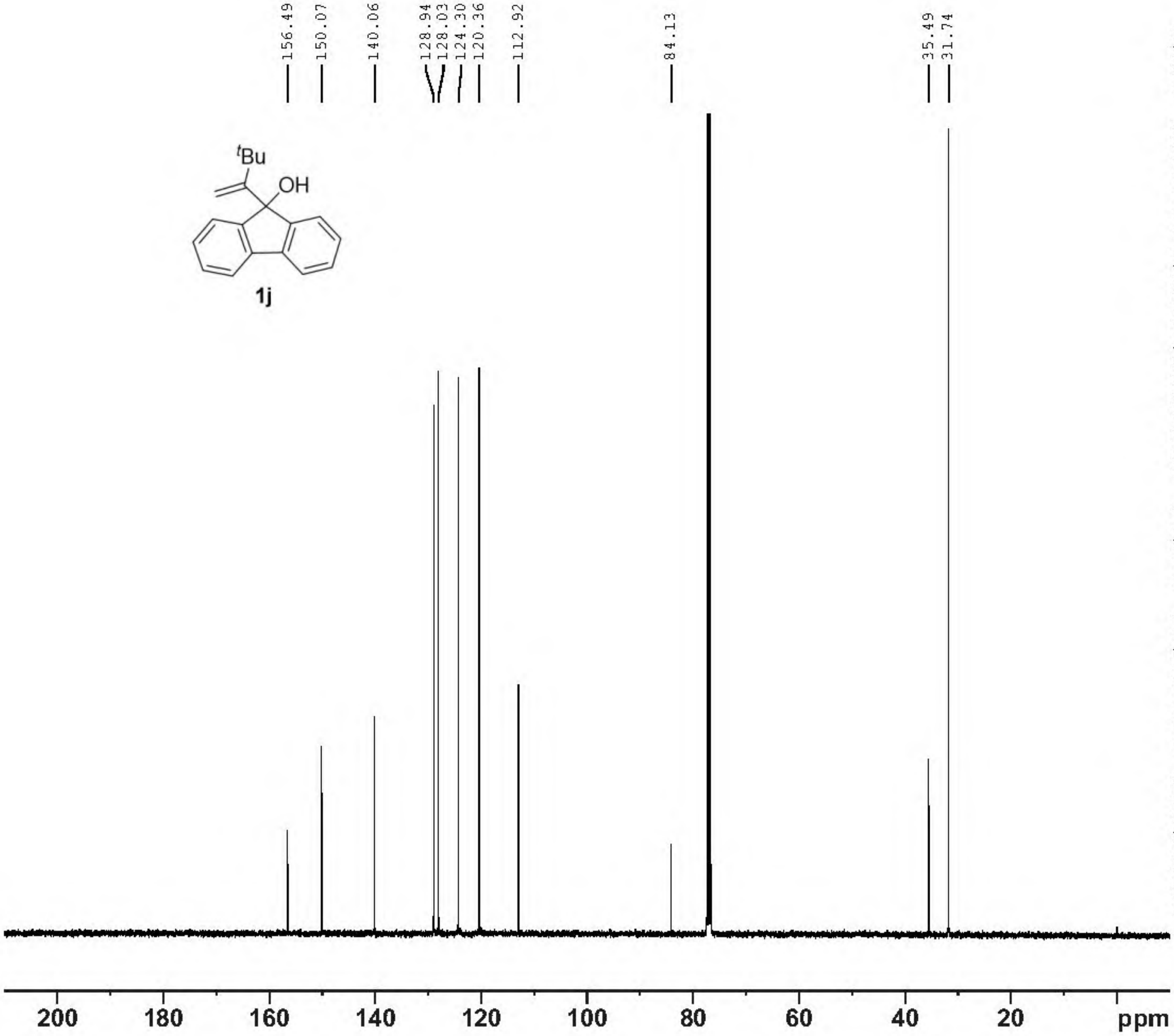


```
Current Data Parameters
NAME          zzj-SM-tBu-C
EXPNO                    1
PROCNO                   1

F2 - Acquisition Parameters
Date_             20240711
Time                  2.17
INSTRUM              spect
PROBHD   5 mm PABBO BB/
PULPROG            zgpg30
TD                   65536
SOLVENT              CDCl3
NS                    1000
DS                       2
SWH              24038.461 Hz
FIDRES            0.366798 Hz
AQ               1.3631488 sec
RG                  196.92
DW                  20.800 usec
DE                    6.50 usec
TE                   297.2 K
D1              2.00000000 sec
D11             0.03000000 sec
TD0                      1

======== CHANNEL f1 ========
SFO1         100.6228298 MHz
NUC1                     13C
P1                      9.70 usec
PLW1          46.98899841 W

======== CHANNEL f2 ========
SFO2         400.1316005 MHz
NUC2                      1H
CPDPRG[2             waltz16
PCPD2                  90.00 usec
PLW2          11.99499989 W
PLW12          0.34213999 W
PLW13          0.27713001 W

F2 - Processing parameters
SI                     32768
SF            100.6127730 MHz
WDW                       EM
SSB                        0
LB                      1.00 Hz
GB                         0
PC                      1.40
```

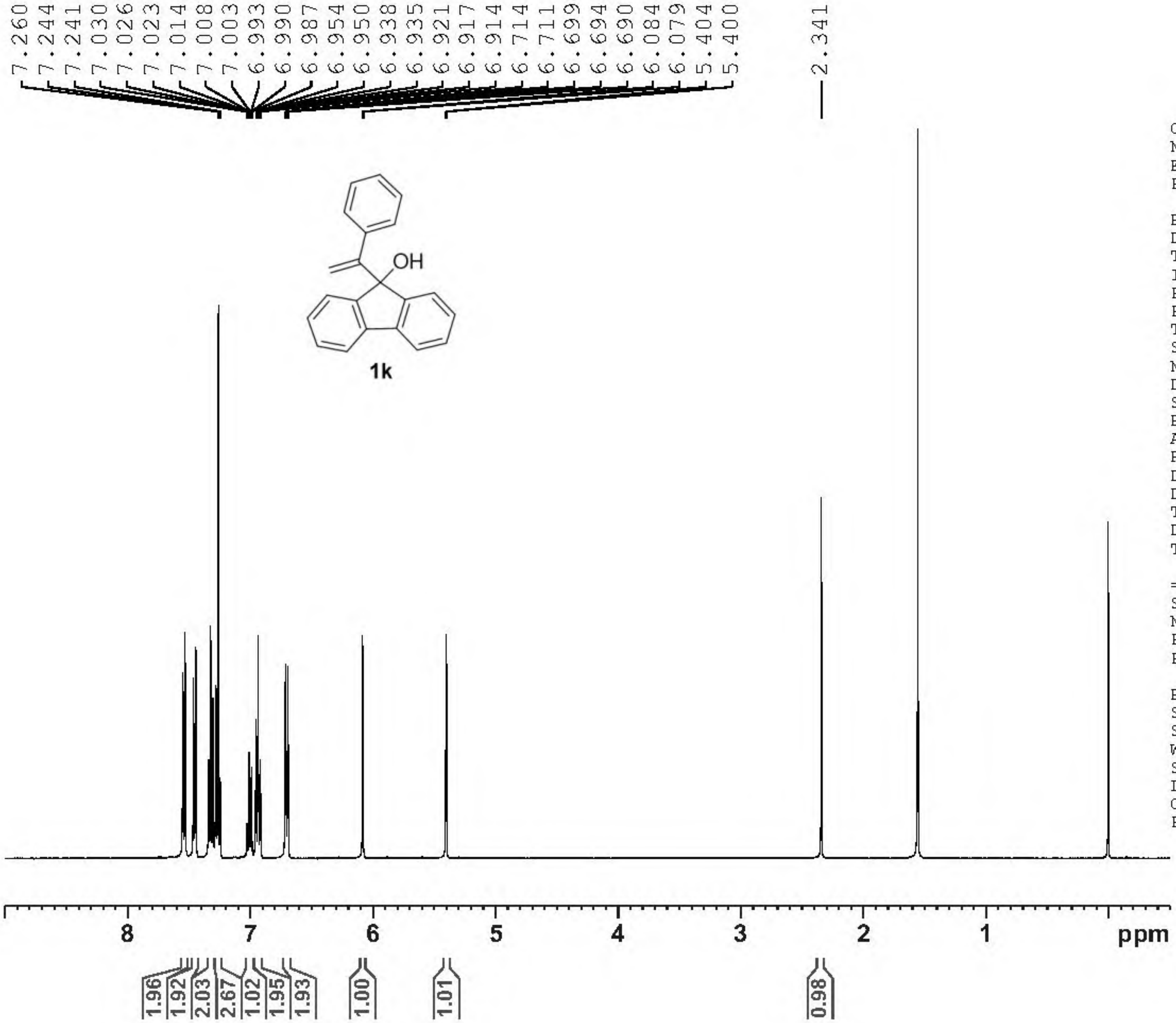


```
Current Data Parameters
NAME          zzj-SM-Ph-H
EXPNO                   1
PROCNO                  1

F2 - Acquisition Parameters
Date_            20240819
Time                22.32
INSTRUM             spect
PROBHD   5 mm PABBO BB/
PULPROG              zg30
TD                  65536
SOLVENT             CDCl3
NS                     16
DS                      2
SWH              8012.820 Hz
FIDRES           0.122266 Hz
AQ              4.0894465 sec
RG                 164.33
DW                 62.400 usec
DE                   6.50 usec
TE                  296.0 K
D1             1.00000000 sec
TD0                     1

======== CHANNEL f1 ========
SFO1          400.1324710 MHz
NUC1                   1H
P1                  14.50 usec
PLW1          11.99499989 W

F2 - Processing parameters
SI                  65536
SF            400.1300098 MHz
WDW                    EM
SSB                     0
LB                   0.30 Hz
GB                      0
PC                   1.00
```

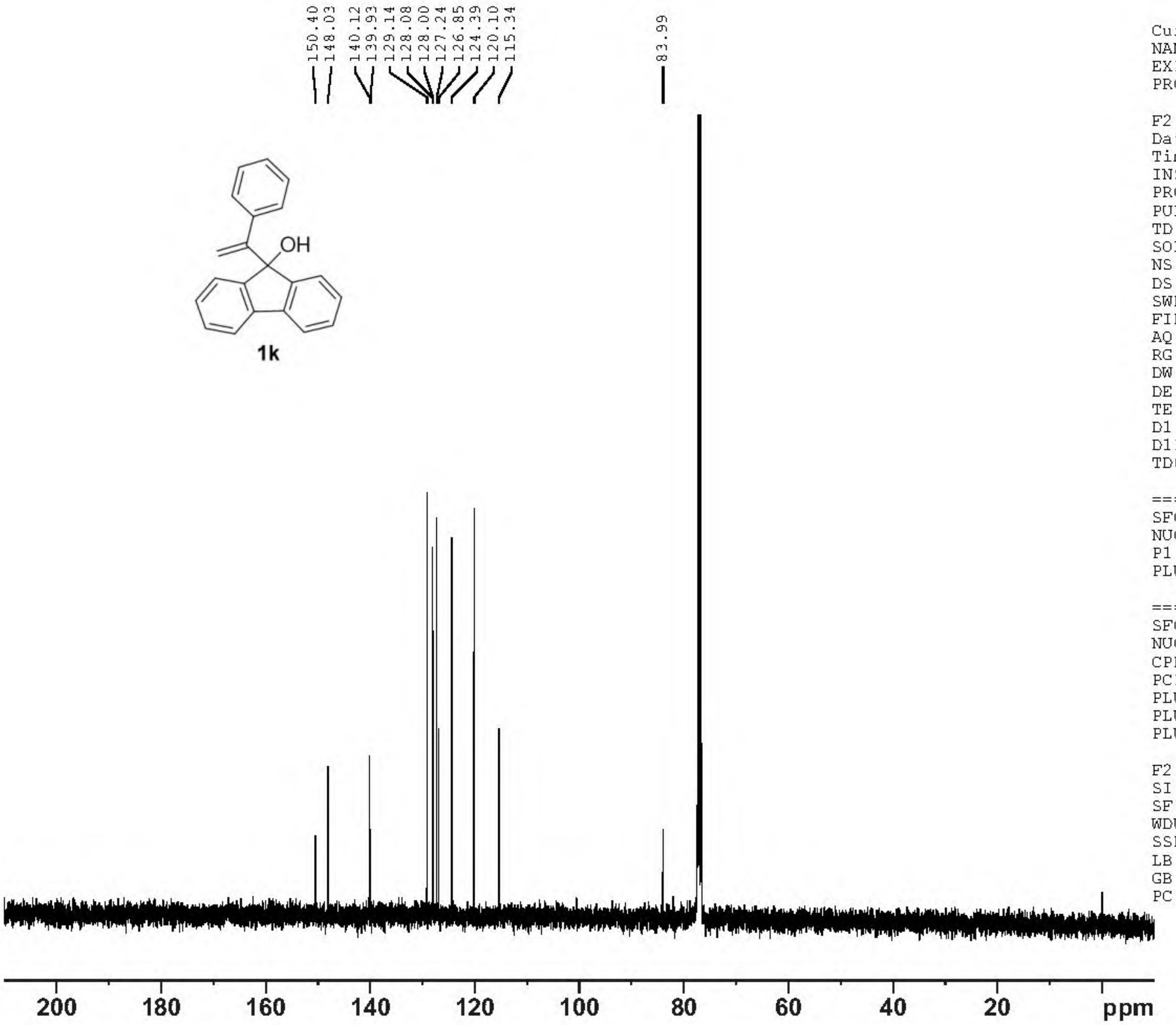

Current Data Parameters
NAME zzj-SM-Ph-C
EXPNO 1
PROCNO 1

F2 - Acquisition Parameters
Date_ 20240819
Time 23.30
INSTRUM spect
PROBHD 5 mm PABBO BB/
PULPROG zgpg30
TD 65536
SOLVENT CDCl3
NS 1000
DS 2
SWH 24038.461 Hz
FIDRES 0.366798 Hz
AQ 1.3631488 sec
RG 196.92
DW 20.800 usec
DE 6.50 usec
TE 297.3 K
D1 2.00000000 sec
D11 0.03000000 sec
TD0 1

======== CHANNEL f1 ========
SFO1 100.6228298 MHz
NUC1 13C
P1 9.70 usec
PLW1 46.98899841 W

======== CHANNEL f2 ========
SFO2 400.1316005 MHz
NUC2 1H
CPDPRG[2 waltz16
PCPD2 90.00 usec
PLW2 11.99499989 W
PLW12 0.34213999 W
PLW13 0.27713001 W

F2 - Processing parameters
SI 32768
SF 100.6127709 MHz
WDW EM
SSB 0
LB 1.00 Hz
GB 0
PC 1.40

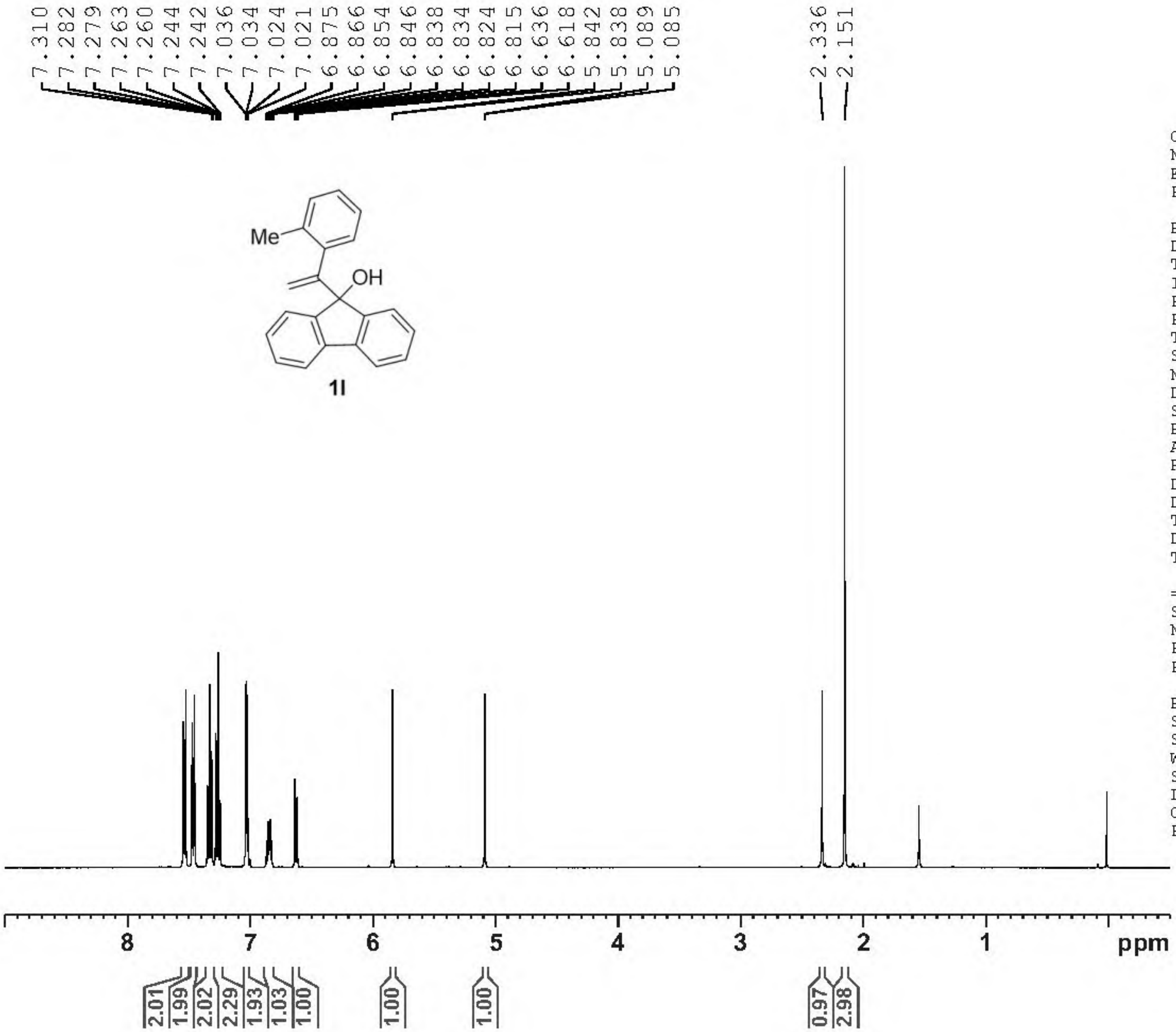

1l


```
Current Data Parameters
NAME      zzj-SM-o-Methyl-H
EXPNO                     1
PROCNO                    1

F2 - Acquisition Parameters
Date_              20240723
Time                   6.42
INSTRUM               spect
PROBHD    5 mm PABBO BB/
PULPROG                zg30
TD                    65536
SOLVENT               CDCl3
NS                       16
DS                        2
SWH                8012.820 Hz
FIDRES             0.122266 Hz
AQ                4.0894465 sec
RG                    82.92
DW                   62.400 usec
DE                     6.50 usec
TE                    296.5 K
D1               1.00000000 sec
TD0                       1

======== CHANNEL f1 ========
SFO1          400.1324710 MHz
NUC1                     1H
P1                    14.50 usec
PLW1            11.99499989 W

F2 - Processing parameters
SI                    65536
SF            400.1300102 MHz
WDW                      EM
SSB                       0
LB                     0.30 Hz
GB                        0
PC                     1.00
```

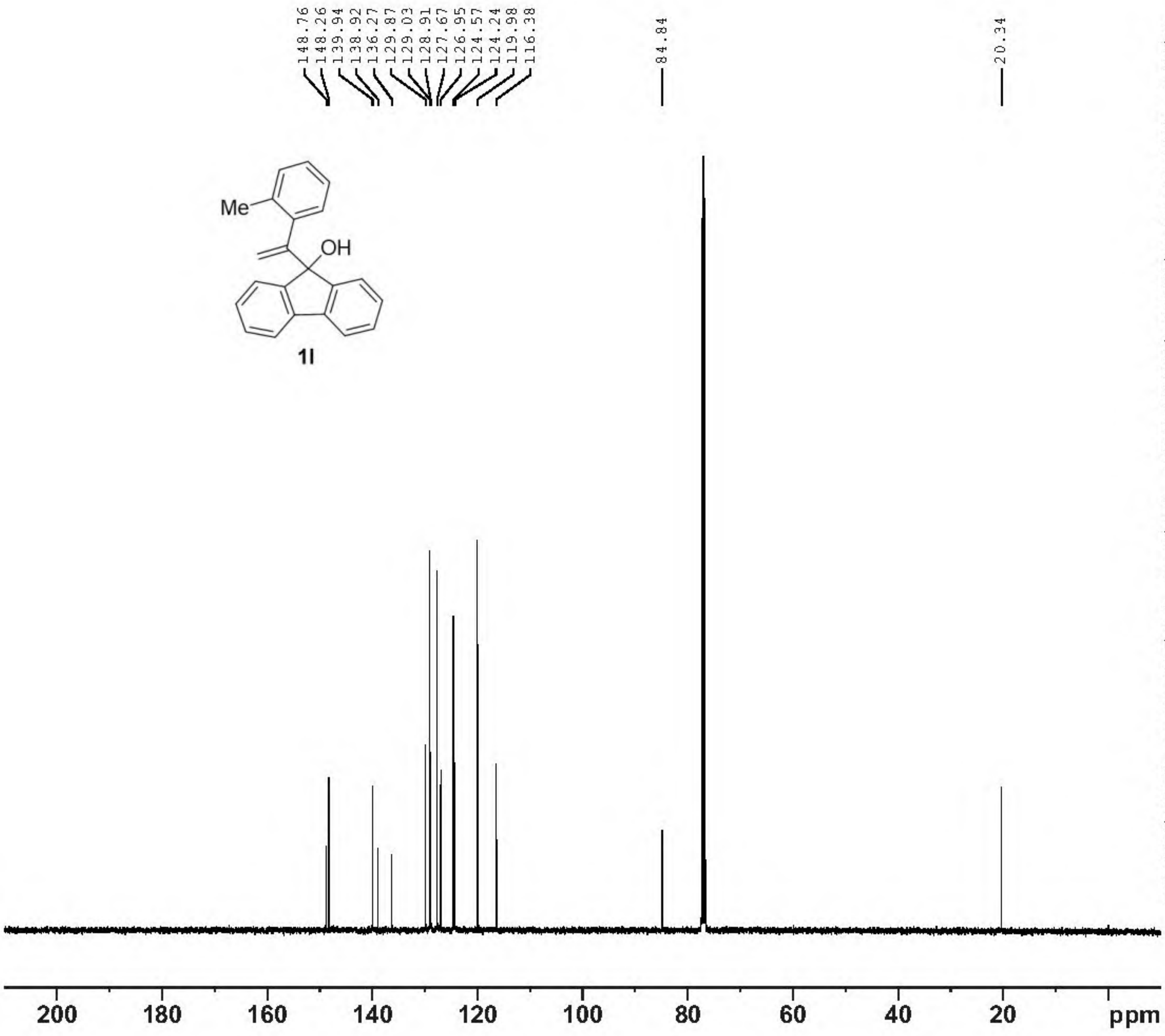


```
Current Data Parameters
NAME      zzj-SM-o-Methyl-H
EXPNO                    2
PROCNO                   1

F2 - Acquisition Parameters
Date_             20240723
Time                  7.28
INSTRUM              spect
PROBHD   5 mm PABBO BB/
PULPROG            zgpg30
TD                  65536
SOLVENT             CDCl3
NS                    800
DS                      2
SWH             24038.461 Hz
FIDRES           0.366798 Hz
AQ              1.3631488 sec
RG                 196.92
DW                 20.800 usec
DE                   6.50 usec
TE                  297.4 K
D1             2.00000000 sec
D11            0.03000000 sec
TD0                     1

======== CHANNEL f1 ========
SFO1          100.6228298 MHz
NUC1                  13C
P1                   9.70 usec
PLW1          46.98899841 W

======== CHANNEL f2 ========
SFO2          400.1316005 MHz
NUC2                   1H
CPDPRG[2          waltz16
PCPD2               90.00 usec
PLW2          11.99499989 W
PLW12          0.34213999 W
PLW13          0.27713001 W

F2 - Processing parameters
SI                  32768
SF            100.6127734 MHz
WDW                    EM
SSB                     0
LB                   1.00 Hz
GB                      0
PC                   1.40
```

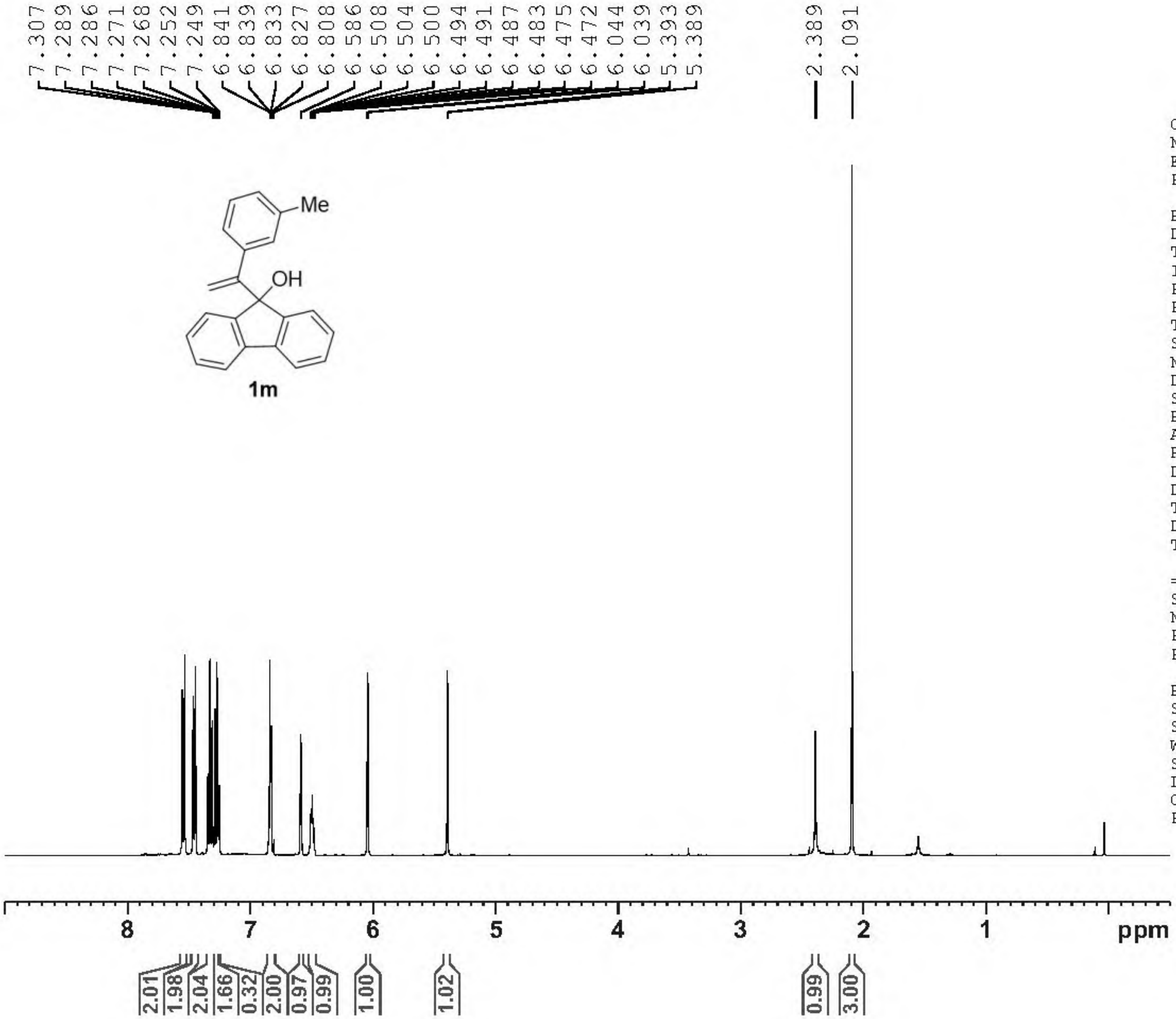

Current Data Parameters
NAME zzj-SM-m-Me-H
EXPNO 1
PROCNO 1

F2 - Acquisition Parameters
Date_ 20240808
Time 0.45
INSTRUM spect
PROBHD 5 mm PABBO BB/
PULPROG zg30
TD 65536
SOLVENT CDCl3
NS 16
DS 2
SWH 8012.820 Hz
FIDRES 0.122266 Hz
AQ 4.0894465 sec
RG 34.77
DW 62.400 usec
DE 6.50 usec
TE 296.5 K
D1 1.00000000 sec
TD0 1

======== CHANNEL f1 ========
SFO1 400.1324710 MHz
NUC1 1H
P1 14.50 usec
PLW1 11.99499989 W

F2 - Processing parameters
SI 65536
SF 400.1300097 MHz
WDW EM
SSB 0
LB 0.30 Hz
GB 0
PC 1.00

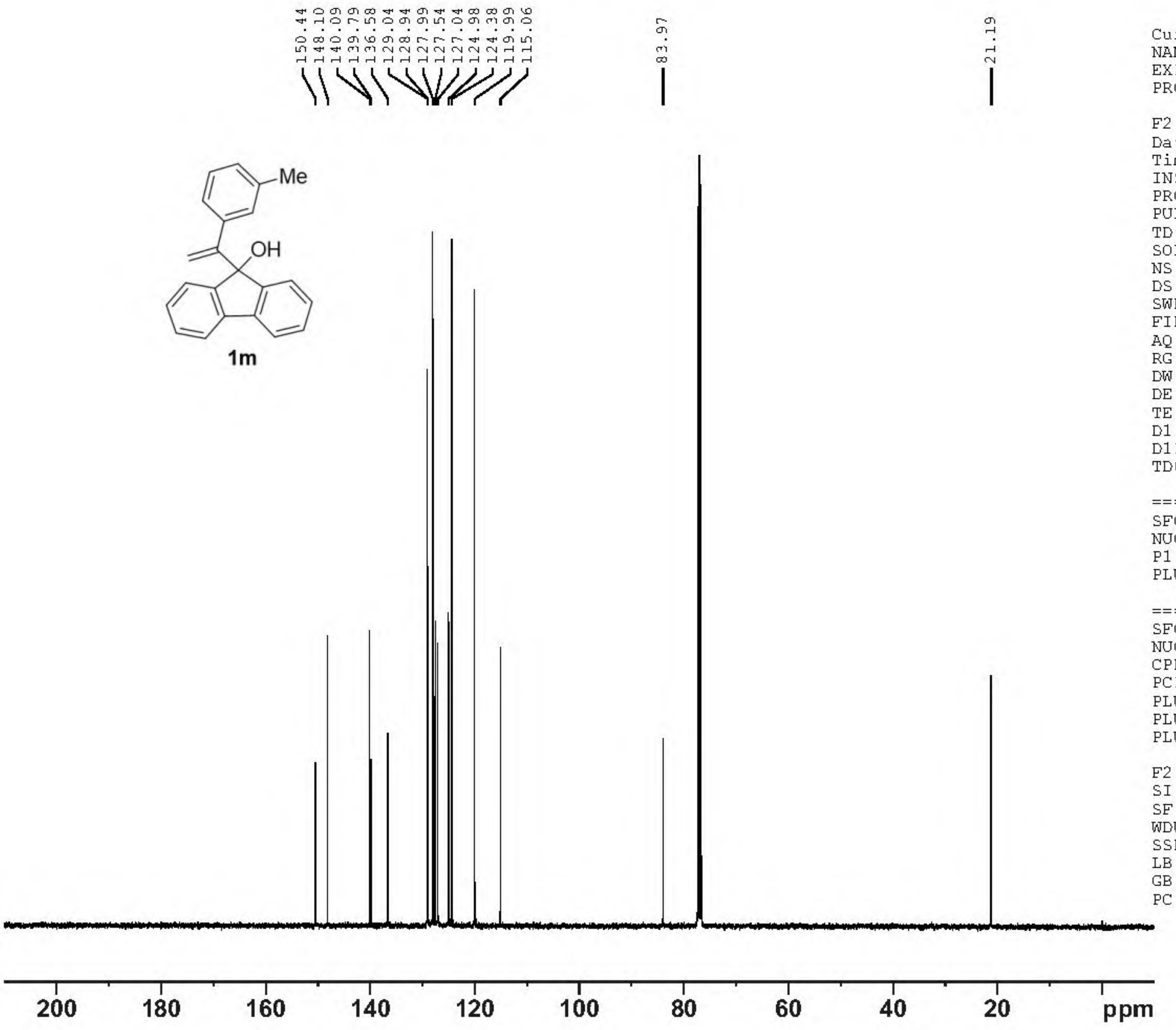


```
Current Data Parameters
NAME        zzj-SM-m-Me-C
EXPNO                   1
PROCNO                  1

F2 - Acquisition Parameters
Date_            20240808
Time                 1.43
INSTRUM             spect
PROBHD    5 mm PABBO BB/
PULPROG           zgpg30
TD                  65536
SOLVENT             CDCl3
NS                   1000
DS                      2
SWH             24038.461 Hz
FIDRES           0.366798 Hz
AQ              1.3631488 sec
RG                 196.92
DW                 20.800 usec
DE                   6.50 usec
TE                  297.5 K
D1             2.00000000 sec
D11            0.03000000 sec
TD0                     1

======== CHANNEL f1 ========
SFO1          100.6228298 MHz
NUC1                  13C
P1                   9.70 usec
PLW1          46.98899841 W

======== CHANNEL f2 ========
SFO2          400.1316005 MHz
NUC2                   1H
CPDPRG[2          waltz16
PCPD2               90.00 usec
PLW2          11.99499989 W
PLW12          0.34213999 W
PLW13          0.27713001 W

F2 - Processing parameters
SI                  32768
SF            100.6127766 MHz
WDW                    EM
SSB                     0
LB                   1.00 Hz
GB                      0
PC                   1.40
```

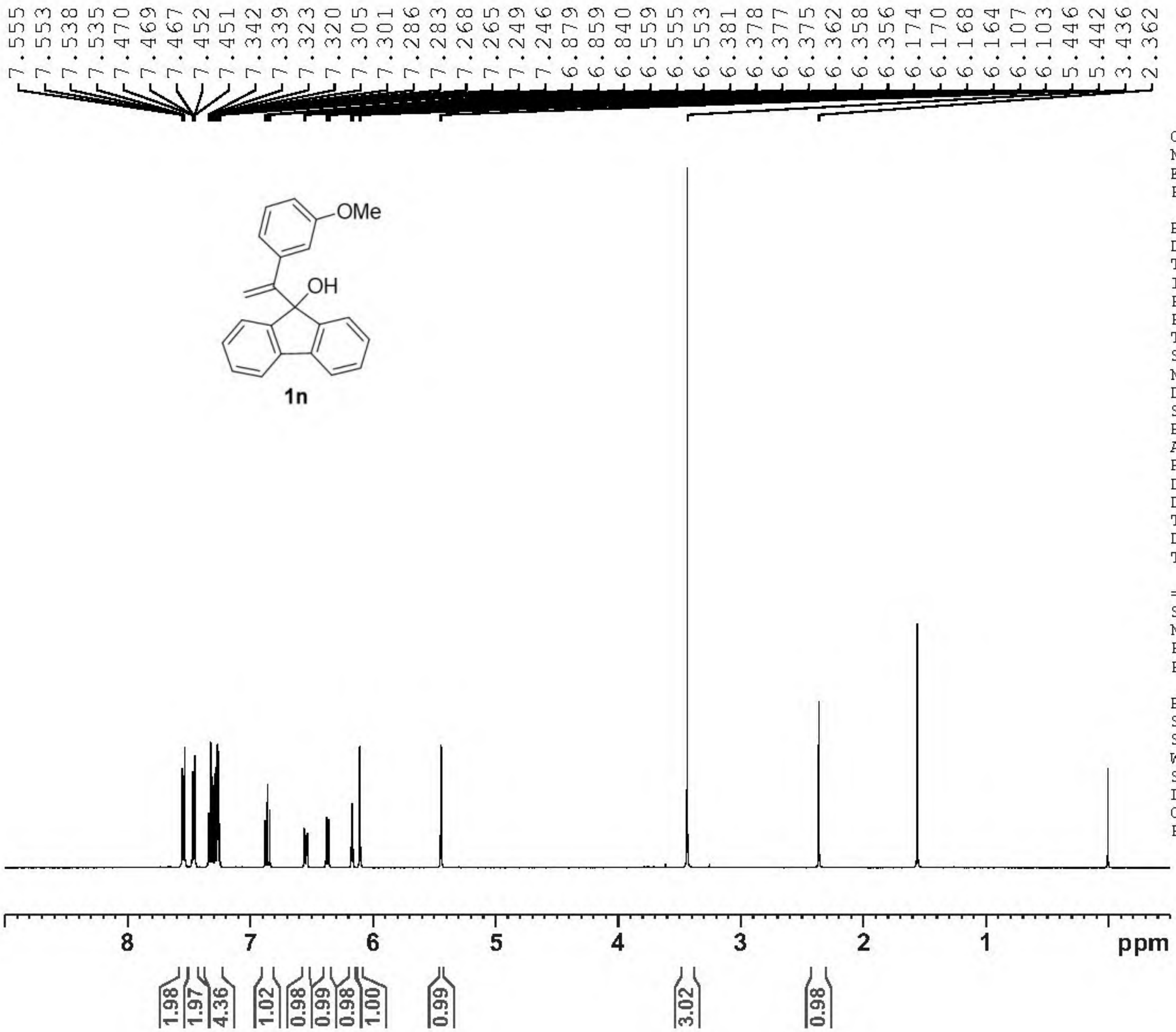


```
Current Data Parameters
NAME          zzj-SM-OMe-H
EXPNO                    1
PROCNO                   1

F2 - Acquisition Parameters
Date_             20240820
Time                  0.37
INSTRUM              spect
PROBHD   5 mm PABBO BB/
PULPROG               zg30
TD                   65536
SOLVENT              CDCl3
NS                      16
DS                       2
SWH              8012.820 Hz
FIDRES           0.122266 Hz
AQ              4.0894465 sec
RG                  126.97
DW                  62.400 usec
DE                    6.50 usec
TE                   296.5 K
D1              1.00000000 sec
TD0                      1

======== CHANNEL f1 ========
SFO1         400.1324710 MHz
NUC1                    1H
P1                   14.50 usec
PLW1           11.99499989 W

F2 - Processing parameters
SI                   65536
SF           400.1300098 MHz
WDW                     EM
SSB                      0
LB                    0.30 Hz
GB                       0
PC                    1.00
```

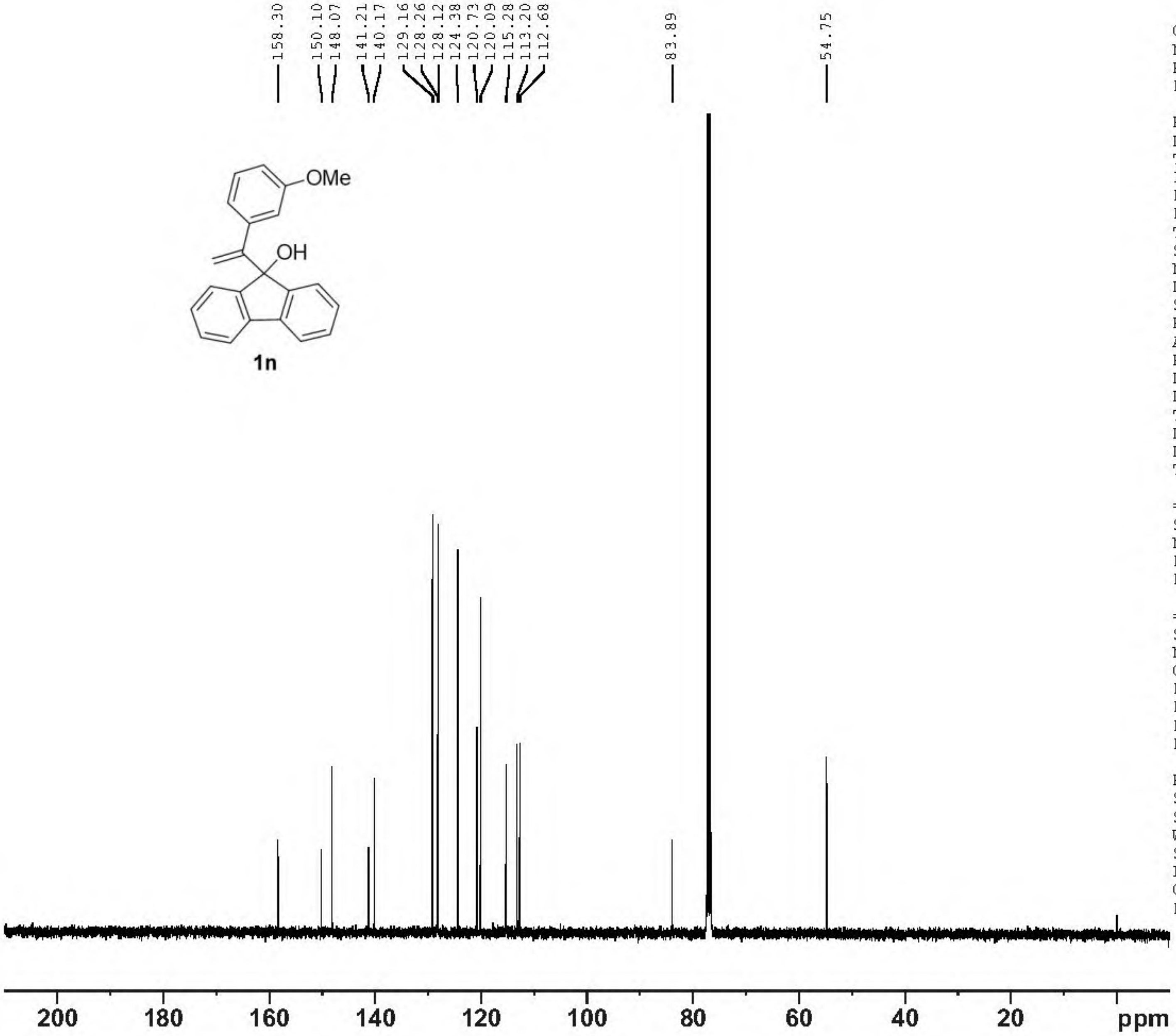


```
Current Data Parameters
NAME        zzj-SM-OMe-C
EXPNO                  1
PROCNO                 1

F2 - Acquisition Parameters
Date_           20240820
Time                1.35
INSTRUM            spect
PROBHD   5 mm PABBO BB/
PULPROG           zgpg30
TD                 65536
SOLVENT            CDCl3
NS                  1000
DS                     2
SWH            24038.461 Hz
FIDRES          0.366798 Hz
AQ             1.3631488 sec
RG                196.92
DW                20.800 usec
DE                  6.50 usec
TE                 297.4 K
D1            2.00000000 sec
D11           0.03000000 sec
TD0                    1

======== CHANNEL f1 ========
SFO1         100.6228298 MHz
NUC1                 13C
P1                  9.70 usec
PLW1         46.98899841 W

======== CHANNEL f2 ========
SFO2         400.1316005 MHz
NUC2                  1H
CPDPRG[2         waltz16
PCPD2              90.00 usec
PLW2         11.99499989 W
PLW12         0.34213999 W
PLW13         0.27713001 W

F2 - Processing parameters
SI                 32768
SF           100.6127718 MHz
WDW                   EM
SSB                    0
LB                  1.00 Hz
GB                     0
PC                  1.40
```

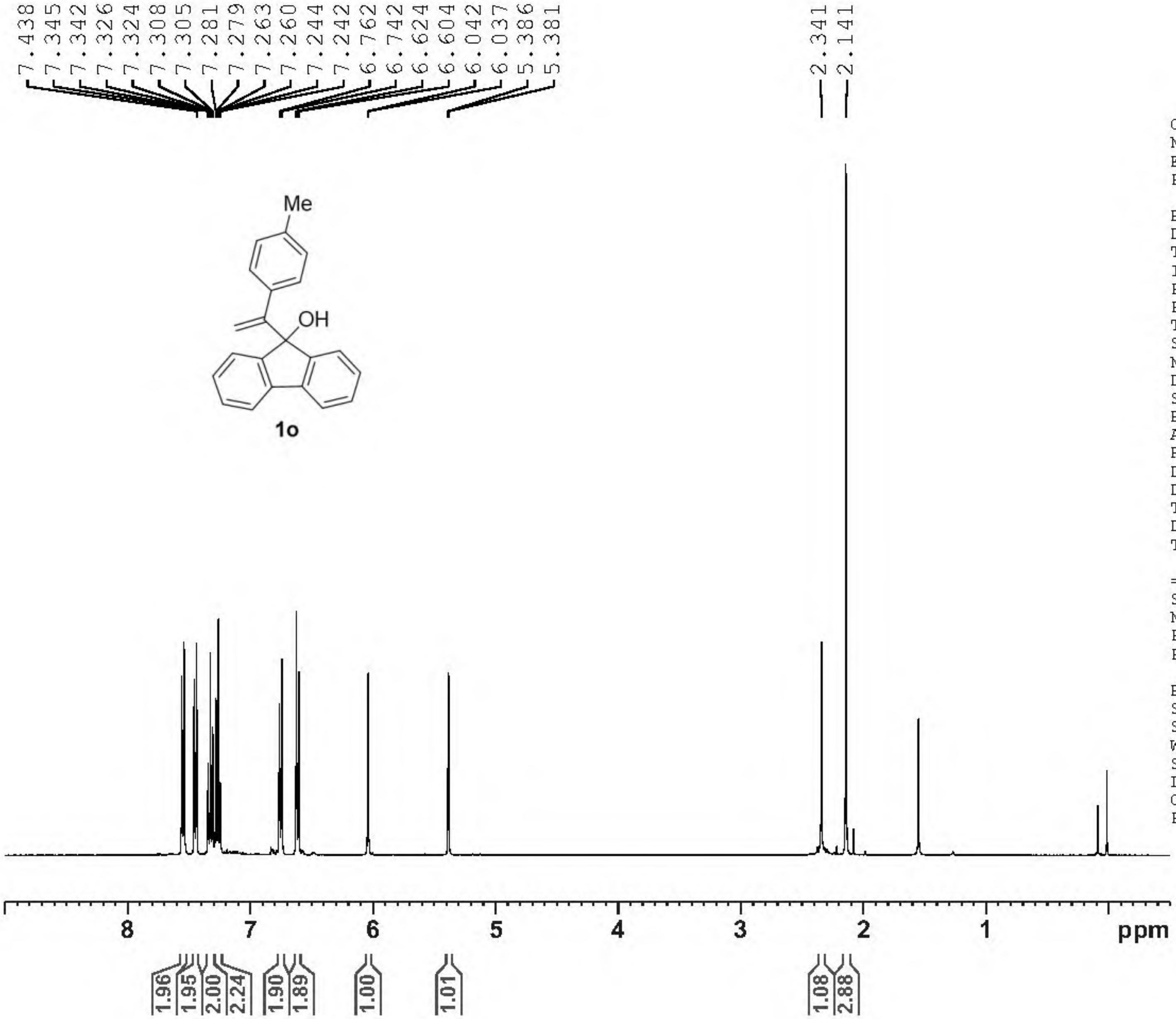


```
Current Data Parameters
NAME        zzj-SM-p-Me-H
EXPNO                   1
PROCNO                  1

F2 - Acquisition Parameters
Date_            20240808
Time                 1.47
INSTRUM             spect
PROBHD   5 mm PABBO BB/
PULPROG              zg30
TD                  65536
SOLVENT             CDCl3
NS                     16
DS                      2
SWH             8012.820 Hz
FIDRES          0.122266 Hz
AQ             4.0894465 sec
RG                  88.84
DW                 62.400 usec
DE                   6.50 usec
TE                  296.6 K
D1            1.00000000 sec
TD0                     1

======== CHANNEL f1 ========
SFO1         400.1324710 MHz
NUC1                   1H
P1                  14.50 usec
PLW1          11.99499989 W

F2 - Processing parameters
SI                  65536
SF           400.1300103 MHz
WDW                    EM
SSB                     0
LB                   0.30 Hz
GB                      0
PC                   1.00
```

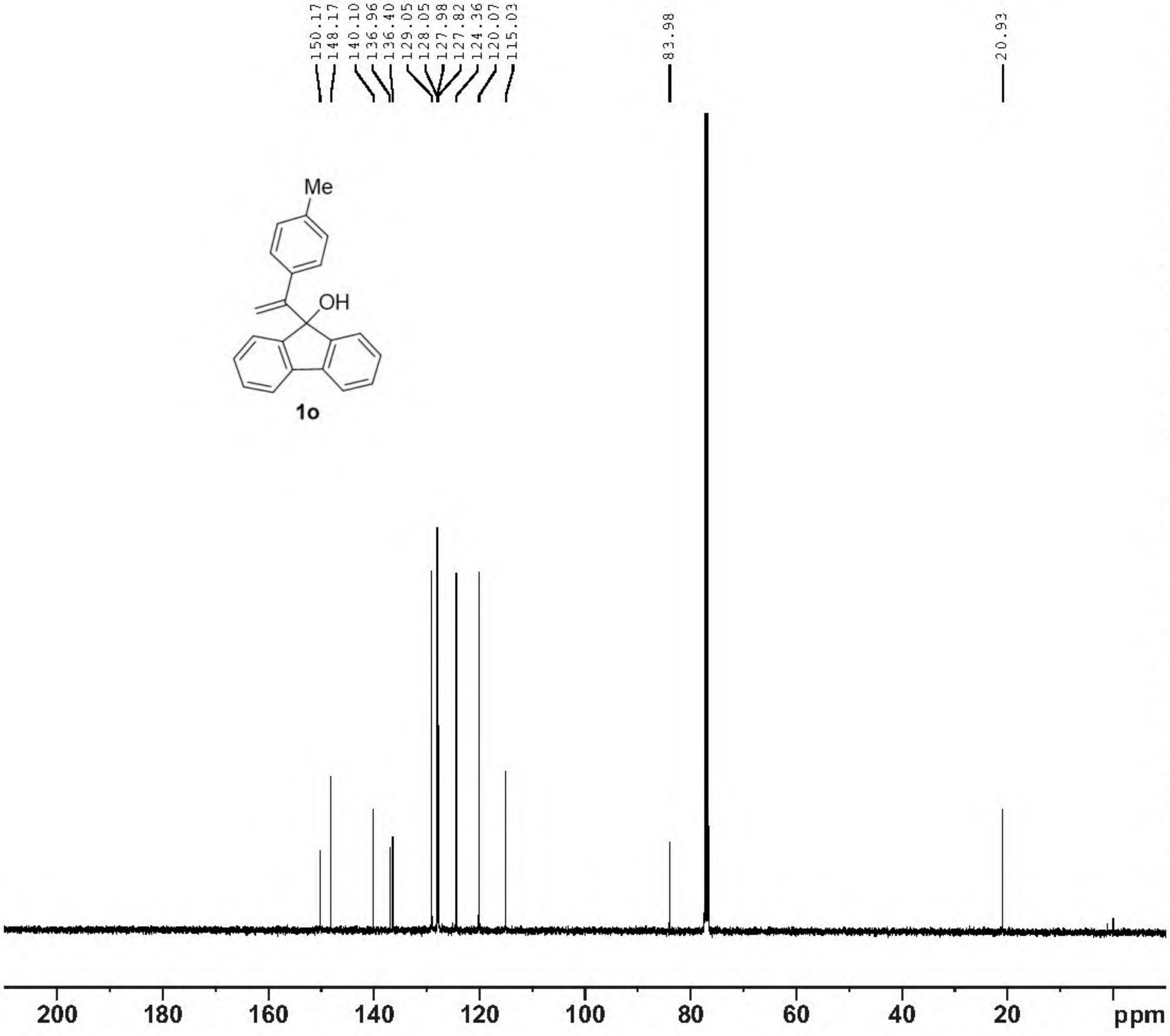


```
Current Data Parameters
NAME       zzj-SM-p-Me-C
EXPNO                  1
PROCNO                 1

F2 - Acquisition Parameters
Date_           20240808
Time                2.45
INSTRUM            spect
PROBHD   5 mm PABBO BB/
PULPROG          zgpg30
TD                 65536
SOLVENT            CDCl3
NS                  1000
DS                     2
SWH           24038.461 Hz
FIDRES         0.366798 Hz
AQ            1.3631488 sec
RG                196.92
DW                20.800 usec
DE                  6.50 usec
TE                 297.5 K
D1            2.00000000 sec
D11           0.03000000 sec
TD0                    1

======== CHANNEL f1 ========
SFO1        100.6228298 MHz
NUC1                 13C
P1                  9.70 usec
PLW1         46.98899841 W

======== CHANNEL f2 ========
SFO2        400.1316005 MHz
NUC2                  1H
CPDPRG[2         waltz16
PCPD2              90.00 usec
PLW2         11.99499989 W
PLW12         0.34213999 W
PLW13         0.27713001 W

F2 - Processing parameters
SI                 32768
SF           100.6127735 MHz
WDW                   EM
SSB                    0
LB                  1.00 Hz
GB                     0
PC                  1.40
```

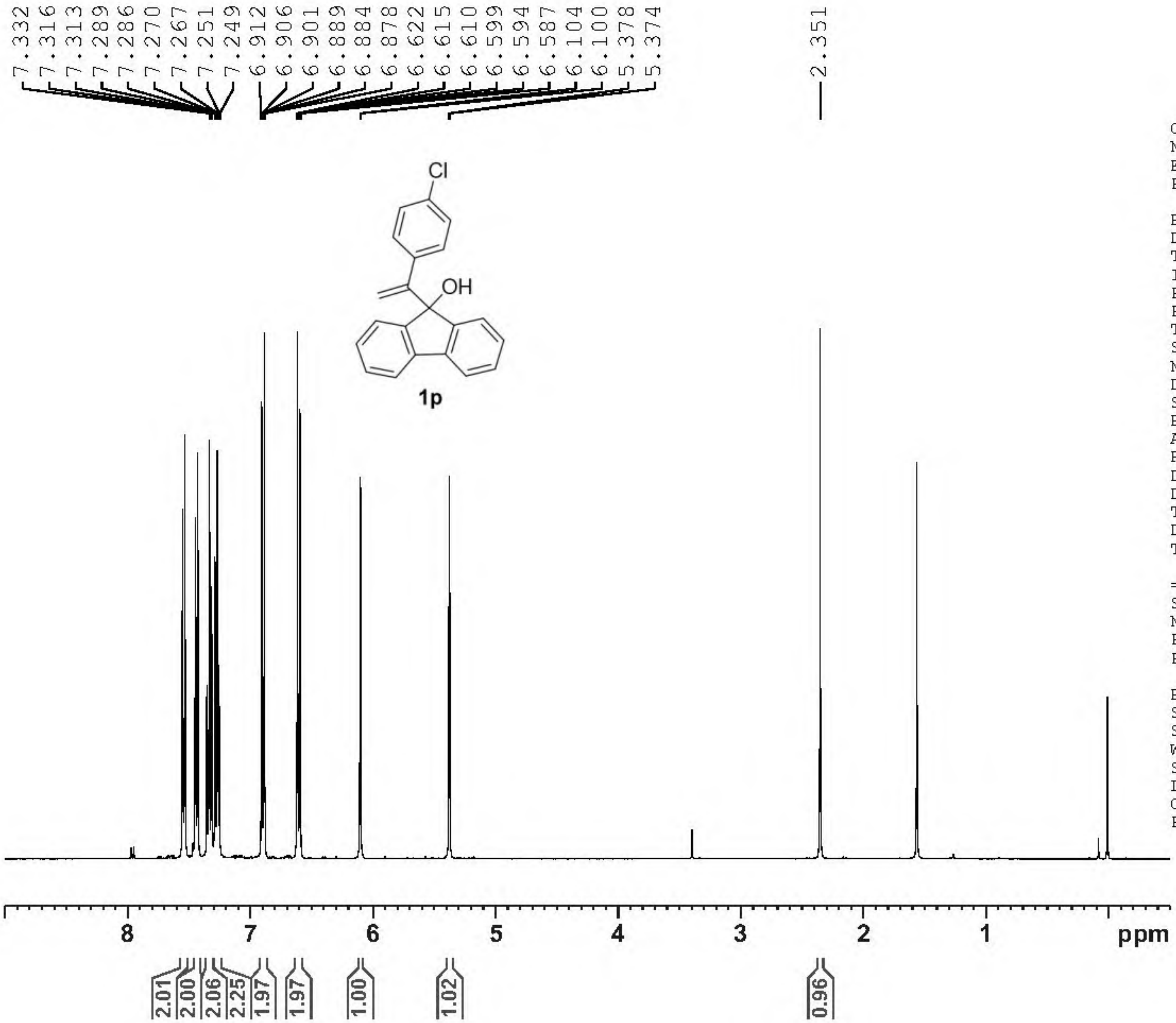

Current Data Parameters
NAME zzj-SM-p-Cl-Ph-H
EXPNO 1
PROCNO 1

F2 - Acquisition Parameters
Date_ 20240807
Time 22.41
INSTRUM spect
PROBHD 5 mm PABBO BB/
PULPROG zg30
TD 65536
SOLVENT CDCl3
NS 16
DS 2
SWH 8012.820 Hz
FIDRES 0.122266 Hz
AQ 4.0894465 sec
RG 103.52
DW 62.400 usec
DE 6.50 usec
TE 296.1 K
D1 1.00000000 sec
TD0 1

======== CHANNEL f1 ========
SFO1 400.1324710 MHz
NUC1 1H
P1 14.50 usec
PLW1 11.99499989 W

F2 - Processing parameters
SI 65536
SF 400.1300096 MHz
WDW EM
SSB 0
LB 0.30 Hz
GB 0
PC 1.00

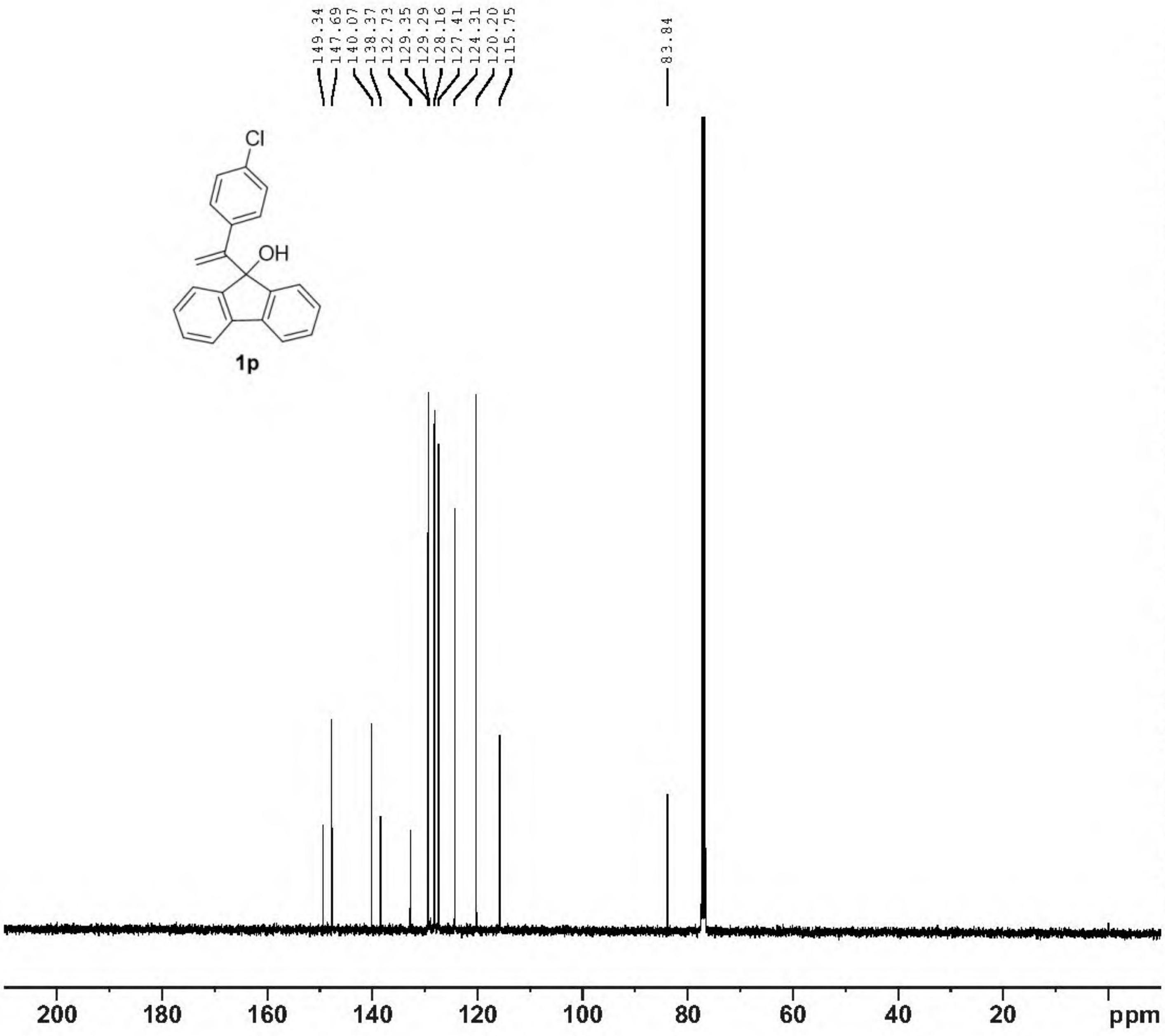


```
Current Data Parameters
NAME      zzj-SM-p-Cl-Ph-C
EXPNO                    1
PROCNO                   1

F2 - Acquisition Parameters
Date_             20240807
Time                 23.39
INSTRUM              spect
PROBHD   5 mm PABBO BB/
PULPROG            zgpg30
TD                   65536
SOLVENT              CDCl3
NS                    1000
DS                       2
SWH           24038.461 Hz
FIDRES         0.366798 Hz
AQ            1.3631488 sec
RG               196.92
DW               20.800 usec
DE                 6.50 usec
TE                297.4 K
D1           2.00000000 sec
D11          0.03000000 sec
TD0                      1

======== CHANNEL f1 ========
SFO1        100.6228298 MHz
NUC1                   13C
P1                  9.70 usec
PLW1         46.98899841 W

======== CHANNEL f2 ========
SFO2        400.1316005 MHz
NUC2                    1H
CPDPRG[2           waltz16
PCPD2              90.00 usec
PLW2         11.99499989 W
PLW12         0.34213999 W
PLW13         0.27713001 W

F2 - Processing parameters
SI                   32768
SF          100.6127724 MHz
WDW                     EM
SSB                      0
LB                  1.00 Hz
GB                       0
PC                    1.40
```

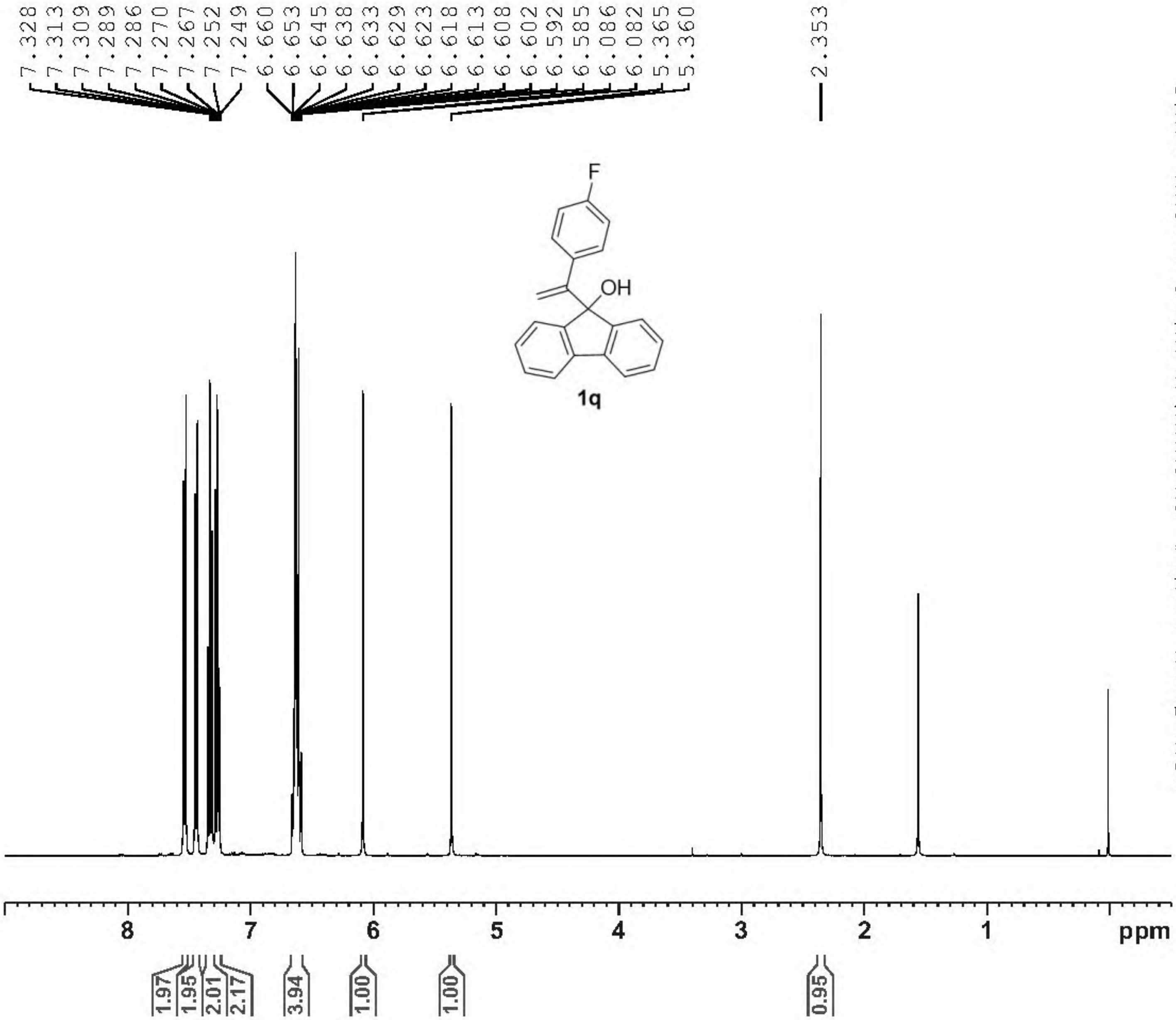

Current Data Parameters
NAME zzj-SM-p-F-Ph-H
EXPNO 1
PROCNO 1

F2 - Acquisition Parameters
Date_ 20240808
Time 5.03
INSTRUM spect
PROBHD 5 mm PABBO BB/
PULPROG zg30
TD 65536
SOLVENT CDCl3
NS 16
DS 2
SWH 8012.820 Hz
FIDRES 0.122266 Hz
AQ 4.0894465 sec
RG 88.84
DW 62.400 usec
DE 6.50 usec
TE 296.5 K
D1 1.00000000 sec
TD0 1

======== CHANNEL f1 ========
SFO1 400.1324710 MHz
NUC1 1H
P1 14.50 usec
PLW1 11.99499989 W

F2 - Processing parameters
SI 65536
SF 400.1300098 MHz
WDW EM
SSB 0
LB 0.30 Hz
GB 0
PC 1.00

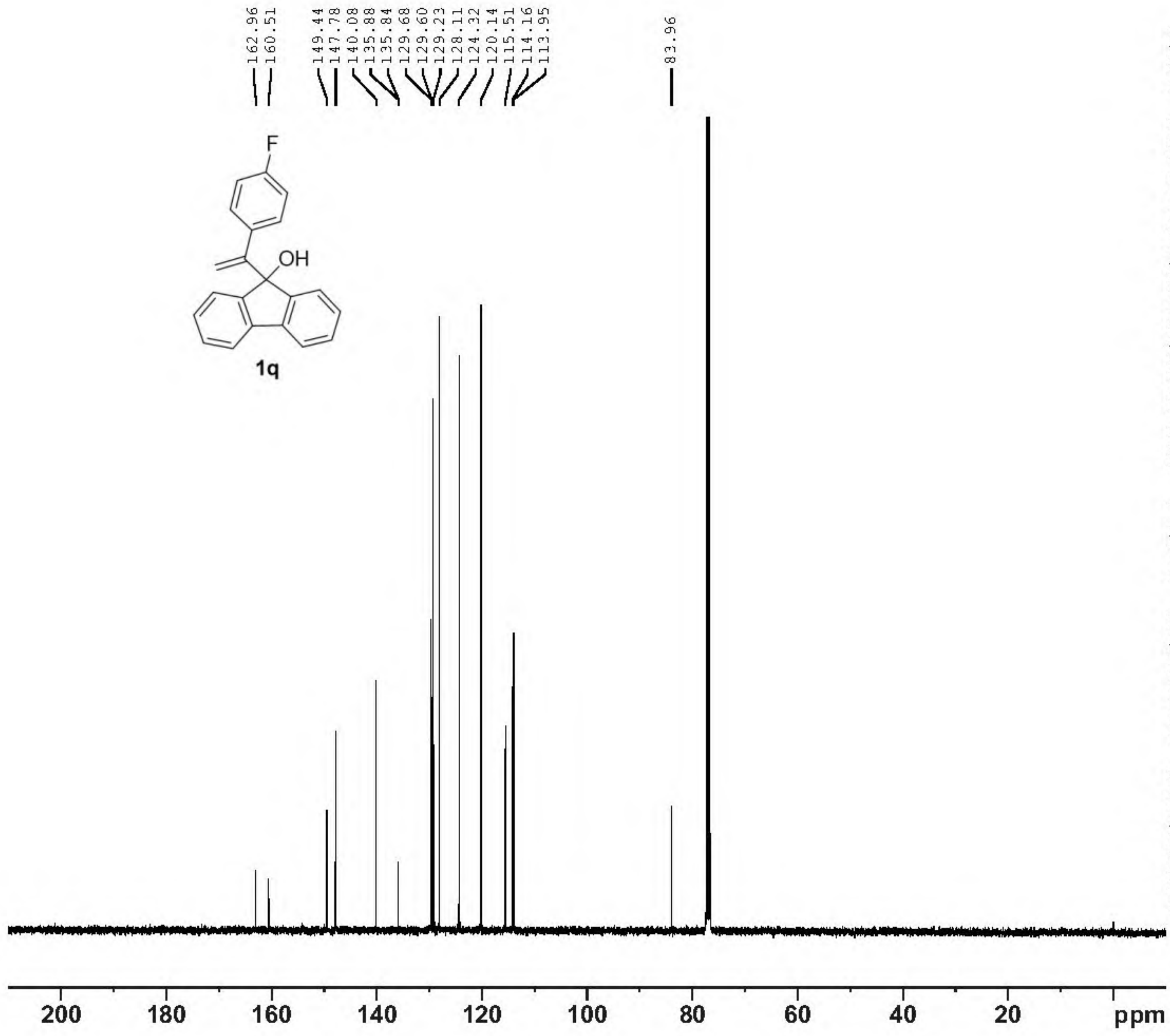


```
Current Data Parameters
NAME        zzj-SM-p-F-Ph-C
EXPNO                     1
PROCNO                    1

F2 - Acquisition Parameters
Date_              20240808
Time                   6.01
INSTRUM               spect
PROBHD     5 mm PABBO BB/
PULPROG              zgpg30
TD                    65536
SOLVENT               CDCl3
NS                     1000
DS                        2
SWH               24038.461 Hz
FIDRES             0.366798 Hz
AQ                1.3631488 sec
RG                   196.92
DW                   20.800 usec
DE                     6.50 usec
TE                    297.5 K
D1               2.00000000 sec
D11              0.03000000 sec
TD0                       1

======== CHANNEL f1 ========
SFO1            100.6228298 MHz
NUC1                    13C
P1                     9.70 usec
PLW1            46.98899841 W

======== CHANNEL f2 ========
SFO2            400.1316005 MHz
NUC2                     1H
CPDPRG[2            waltz16
PCPD2                 90.00 usec
PLW2            11.99499989 W
PLW12            0.34213999 W
PLW13            0.27713001 W

F2 - Processing parameters
SI                    32768
SF              100.6127729 MHz
WDW                      EM
SSB                       0
LB                     1.00 Hz
GB                        0
PC                     1.40
```

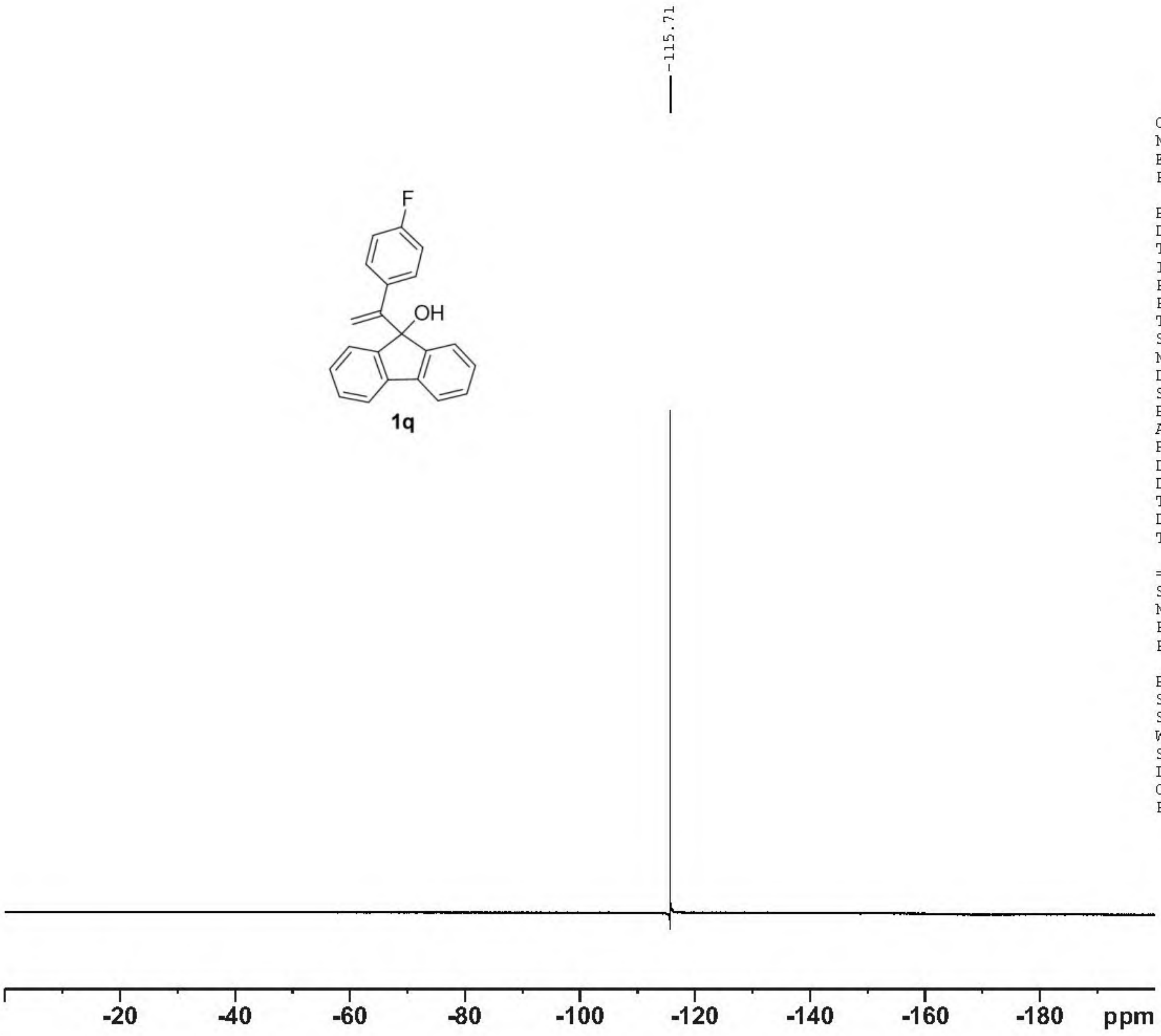


```
Current Data Parameters
NAME      zzj-SM-p-F-Ph-F
EXPNO                   1
PROCNO                  1

F2 - Acquisition Parameters
Date_            20240808
Time                 6.03
INSTRUM             spect
PROBHD   5 mm PABBO BB/
PULPROG           zgflqn
TD                 131072
SOLVENT             CDCl3
NS                     16
DS                      4
SWH            89285.711 Hz
FIDRES          0.681196 Hz
AQ             0.7340032 sec
RG                 196.92
DW                  5.600 usec
DE                   6.50 usec
TE                  296.9 K
D1            1.00000000 sec
TD0                     1

======== CHANNEL f1 ========
SFO1         376.4607164 MHz
NUC1                  19F
P1                  14.70 usec
PLW1         15.99600029 W

F2 - Processing parameters
SI                  65536
SF           376.4983660 MHz
WDW                    EM
SSB                     0
LB                   0.30 Hz
GB                      0
PC                   1.00
```

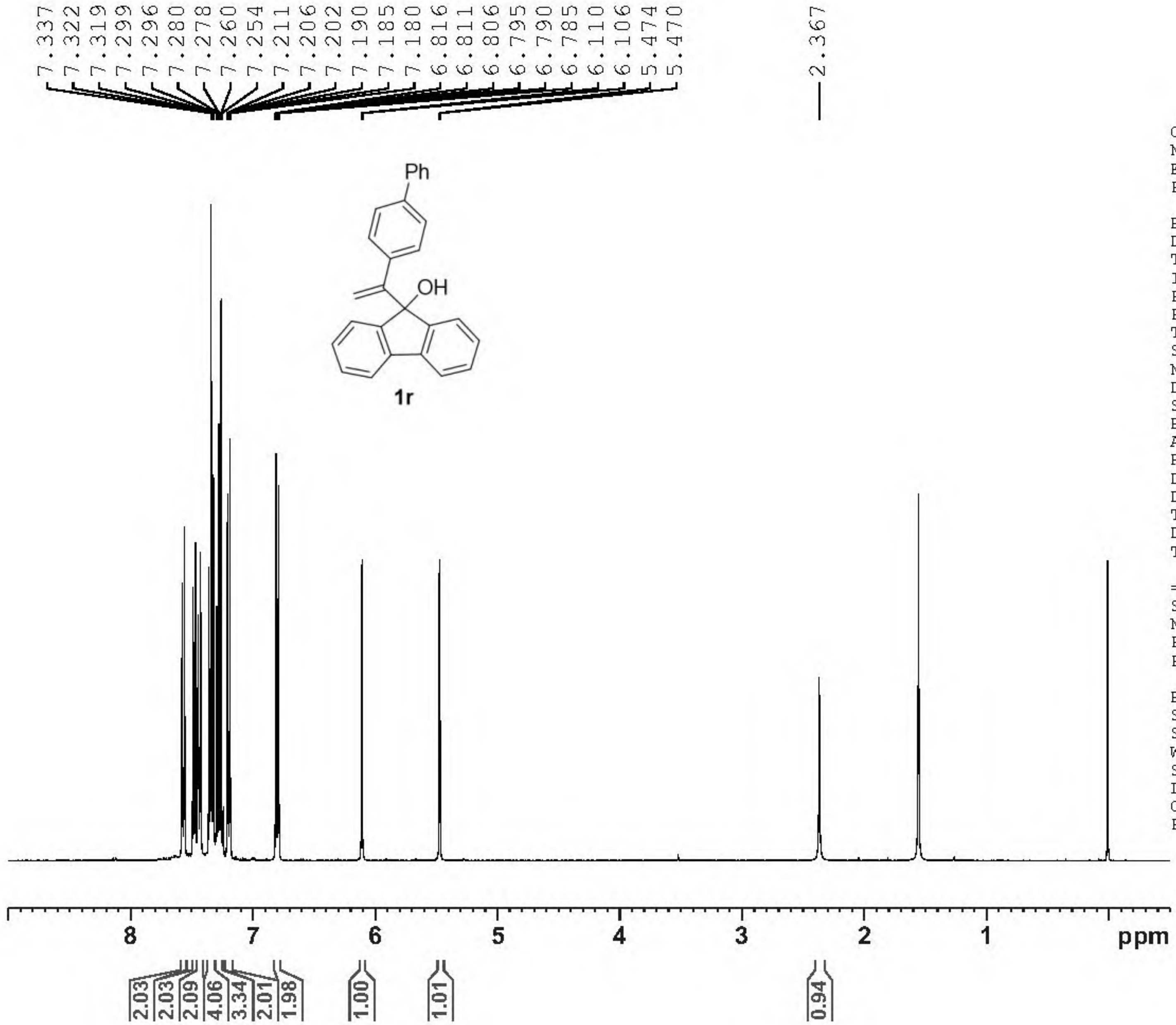


```
Current Data Parameters
NAME       zzj-SM-Ph-Ph-H
EXPNO                   1
PROCNO                  1

F2 - Acquisition Parameters
Date_            20240819
Time                23.35
INSTRUM             spect
PROBHD   5 mm PABBO BB/
PULPROG              zg30
TD                  65536
SOLVENT             CDCl3
NS                     16
DS                      2
SWH              8012.820 Hz
FIDRES           0.122266 Hz
AQ              4.0894465 sec
RG                 126.97
DW                 62.400 usec
DE                   6.50 usec
TE                  296.4 K
D1             1.00000000 sec
TD0                     1

======== CHANNEL f1 ========
SFO1          400.1324710 MHz
NUC1                   1H
P1                  14.50 usec
PLW1          11.99499989 W

F2 - Processing parameters
SI                  65536
SF            400.1300096 MHz
WDW                    EM
SSB                     0
LB                   0.30 Hz
GB                      0
PC                   1.00
```

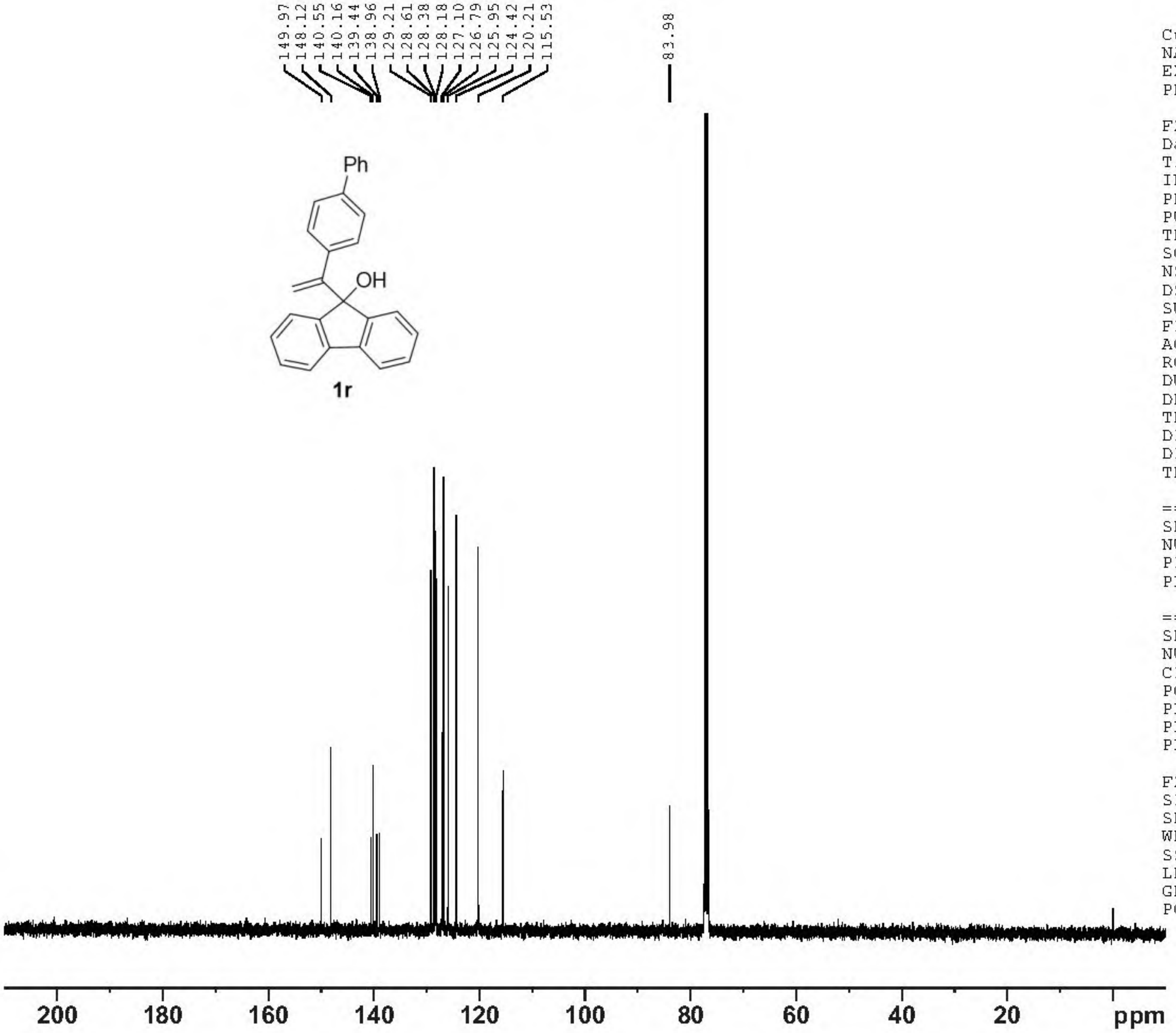

Current Data Parameters
NAME zzj-SM-Ph-Ph-C
EXPNO 1
PROCNO 1

F2 - Acquisition Parameters
Date_ 20240820
Time 0.33
INSTRUM spect
PROBHD 5 mm PABBO BB/
PULPROG zgpg30
TD 65536
SOLVENT CDCl3
NS 1000
DS 2
SWH 24038.461 Hz
FIDRES 0.366798 Hz
AQ 1.3631488 sec
RG 196.92
DW 20.800 usec
DE 6.50 usec
TE 297.4 K
D1 2.00000000 sec
D11 0.03000000 sec
TD0 1

======== CHANNEL f1 ========
SFO1 100.6228298 MHz
NUC1 13C
P1 9.70 usec
PLW1 46.98899841 W

======== CHANNEL f2 ========
SFO2 400.1316005 MHz
NUC2 1H
CPDPRG[2 waltz16
PCPD2 90.00 usec
PLW2 11.99499989 W
PLW12 0.34213999 W
PLW13 0.27713001 W

F2 - Processing parameters
SI 32768
SF 100.6127712 MHz
WDW EM
SSB 0
LB 1.00 Hz
GB 0
PC 1.40

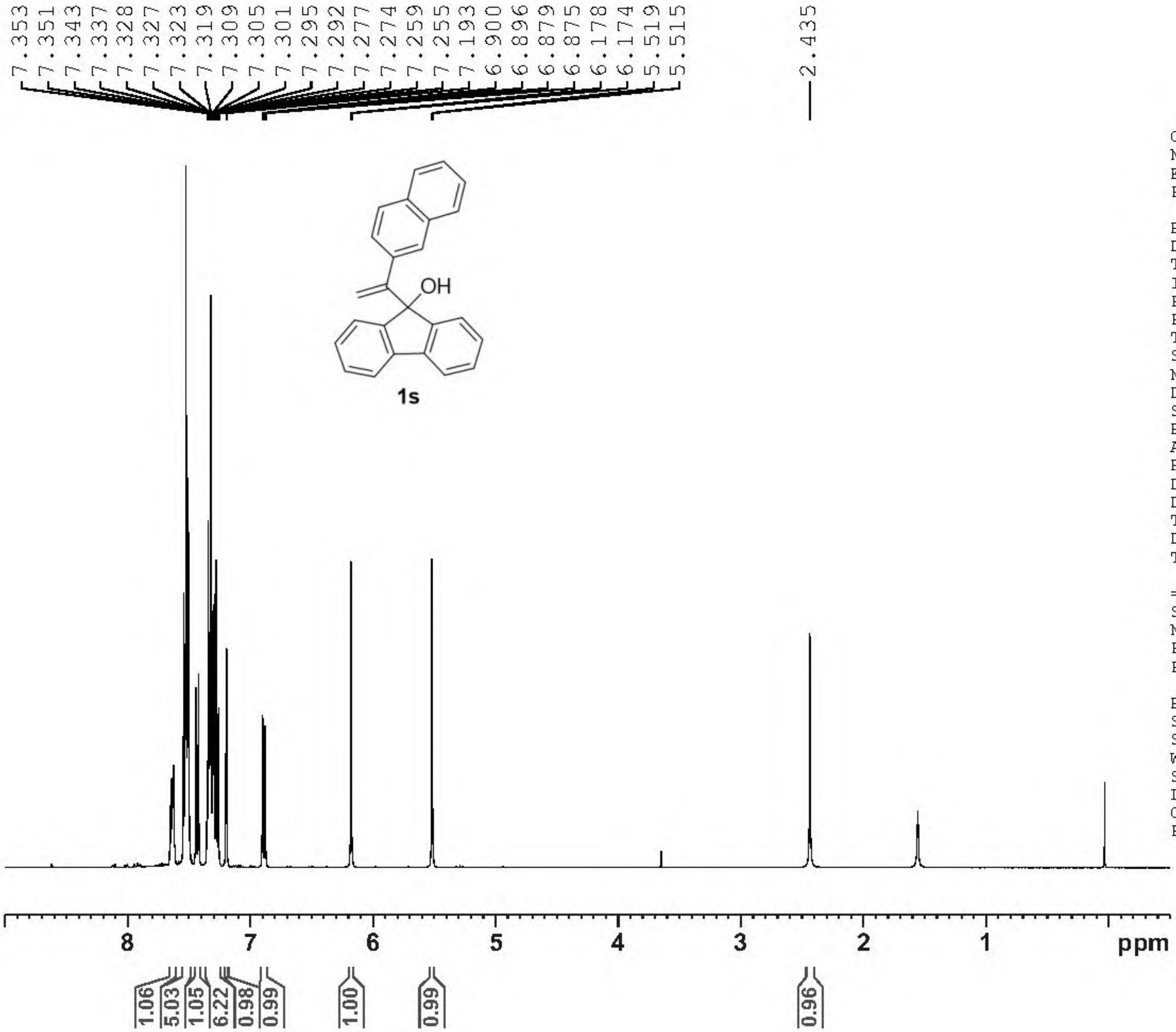


```
Current Data Parameters
NAME          zzj-SM-2-Nap-H
EXPNO                      1
PROCNO                     1

F2 - Acquisition Parameters
Date_               20240807
Time                   23.43
INSTRUM                spect
PROBHD     5 mm PABBO BB/
PULPROG                 zg30
TD                     65536
SOLVENT                CDCl3
NS                        16
DS                         2
SWH                 8012.820 Hz
FIDRES              0.122266 Hz
AQ                 4.0894465 sec
RG                     70.97
DW                    62.400 usec
DE                      6.50 usec
TE                     296.5 K
D1                1.00000000 sec
TD0                        1

======== CHANNEL f1 ========
SFO1             400.1324710 MHz
NUC1                      1H
P1                     14.50 usec
PLW1             11.99499989 W

F2 - Processing parameters
SI                     65536
SF               400.1300098 MHz
WDW                       EM
SSB                        0
LB                      0.30 Hz
GB                         0
PC                      1.00
```

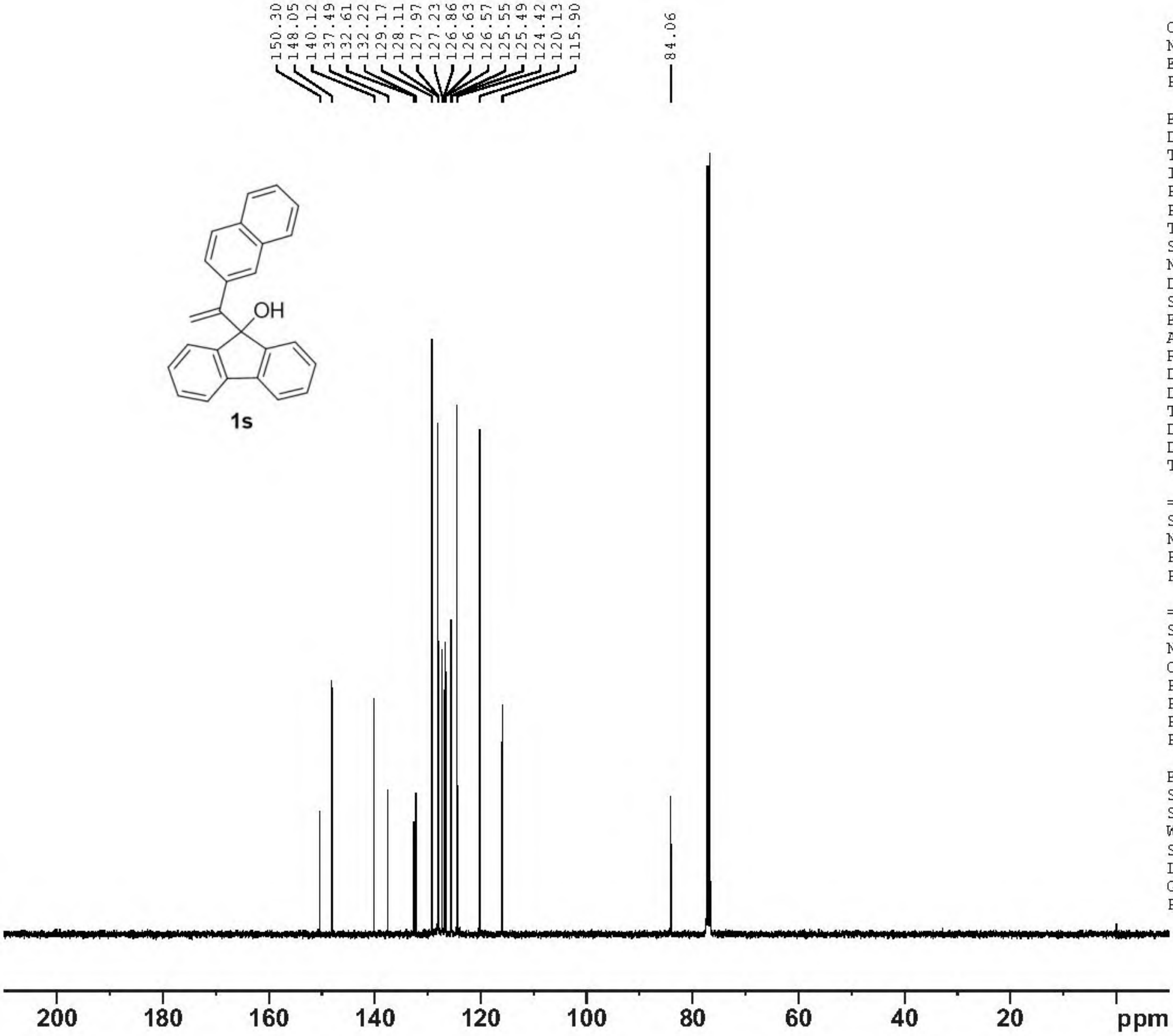

Current Data Parameters
NAME zzj-SM-2-Nap-C
EXPNO 1
PROCNO 1

F2 - Acquisition Parameters
Date_ 20240808
Time 0.41
INSTRUM spect
PROBHD 5 mm PABBO BB/
PULPROG zgpg30
TD 65536
SOLVENT CDCl3
NS 1000
DS 2
SWH 24038.461 Hz
FIDRES 0.366798 Hz
AQ 1.3631488 sec
RG 196.92
DW 20.800 usec
DE 6.50 usec
TE 297.4 K
D1 2.00000000 sec
D11 0.03000000 sec
TD0 1

======== CHANNEL f1 ========
SFO1 100.6228298 MHz
NUC1 13C
P1 9.70 usec
PLW1 46.98899841 W

======== CHANNEL f2 ========
SFO2 400.1316005 MHz
NUC2 1H
CPDPRG[2 waltz16
PCPD2 90.00 usec
PLW2 11.99499989 W
PLW12 0.34213999 W
PLW13 0.27713001 W

F2 - Processing parameters
SI 32768
SF 100.6127752 MHz
WDW EM
SSB 0
LB 1.00 Hz
GB 0
PC 1.40

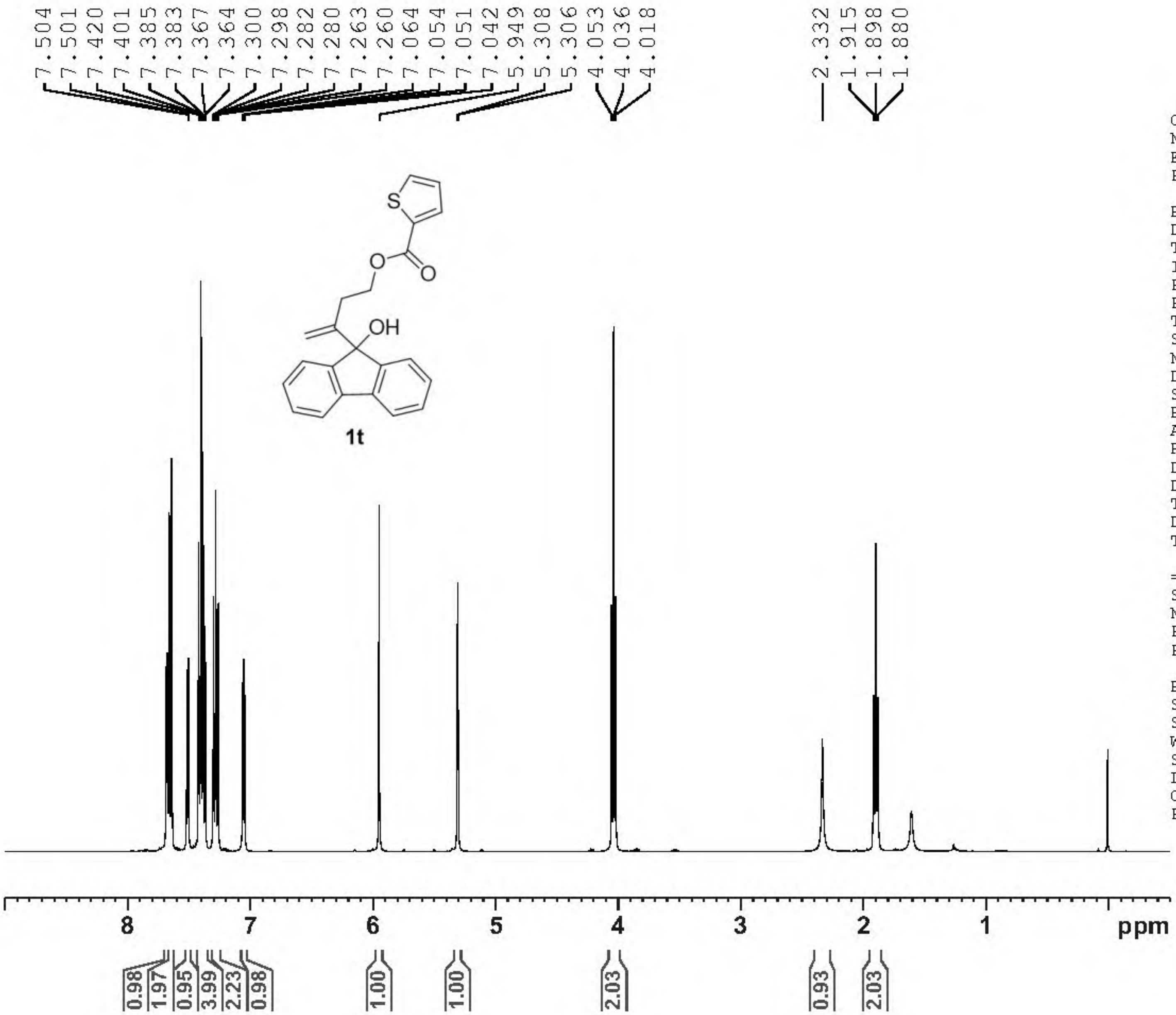

Current Data Parameters
NAME zzj-SM-thio-H
EXPNO 1
PROCNO 1

F2 - Acquisition Parameters
Date_ 20240711
Time_ 5.29
INSTRUM spect
PROBHD 5 mm PABBO BB/
PULPROG zg30
TD 65536
SOLVENT CDCl3
NS 16
DS 2
SWH 8012.820 Hz
FIDRES 0.122266 Hz
AQ 4.0894465 sec
RG 88.84
DW 62.400 usec
DE 6.50 usec
TE 296.3 K
D1 1.00000000 sec
TD0 1

======== CHANNEL f1 ========
SFO1 400.1324710 MHz
NUC1 1H
P1 14.50 usec
PLW1 11.99499989 W

F2 - Processing parameters
SI 65536
SF 400.1300098 MHz
WDW EM
SSB 0
LB 0.30 Hz
GB 0
PC 1.00

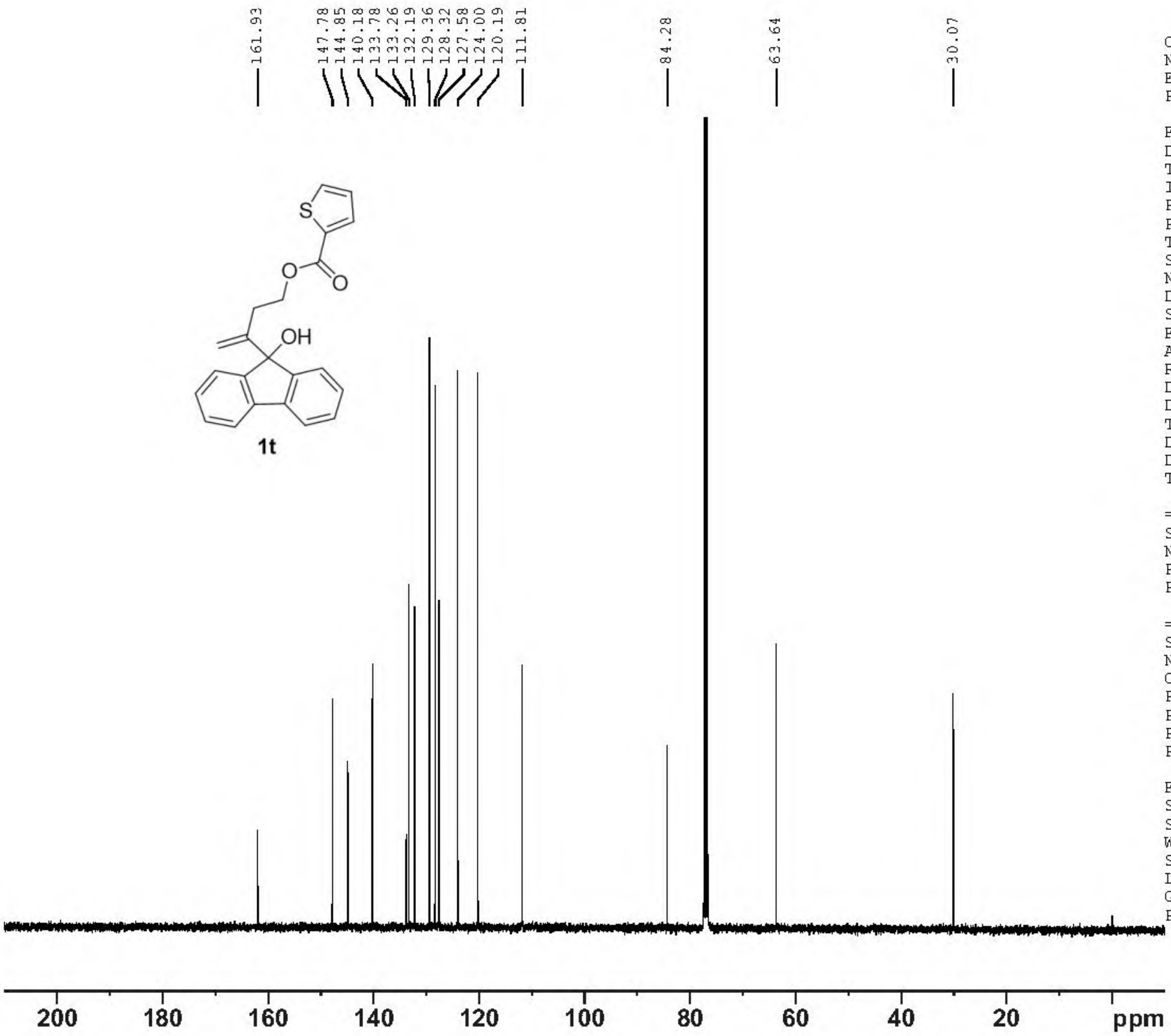

Current Data Parameters
NAME zzj-SM-thio-C
EXPNO 1
PROCNO 1

F2 - Acquisition Parameters
Date_ 20240711
Time_ 6.27
INSTRUM spect
PROBHD 5 mm PABBO BB/
PULPROG zgpg30
TD 65536
SOLVENT CDCl3
NS 1000
DS 2
SWH 24038.461 Hz
FIDRES 0.366798 Hz
AQ 1.3631488 sec
RG 196.92
DW 20.800 usec
DE 6.50 usec
TE 297.2 K
D1 2.00000000 sec
D11 0.03000000 sec
TD0 1

======== CHANNEL f1 ========
SFO1 100.6228298 MHz
NUC1 13C
P1 9.70 usec
PLW1 46.98899841 W

======== CHANNEL f2 ========
SFO2 400.1316005 MHz
NUC2 1H
CPDPRG[2 waltz16
PCPD2 90.00 usec
PLW2 11.99499989 W
PLW12 0.34213999 W
PLW13 0.27713001 W

F2 - Processing parameters
SI 32768
SF 100.6127739 MHz
WDW EM
SSB 0
LB 1.00 Hz
GB 0
PC 1.40

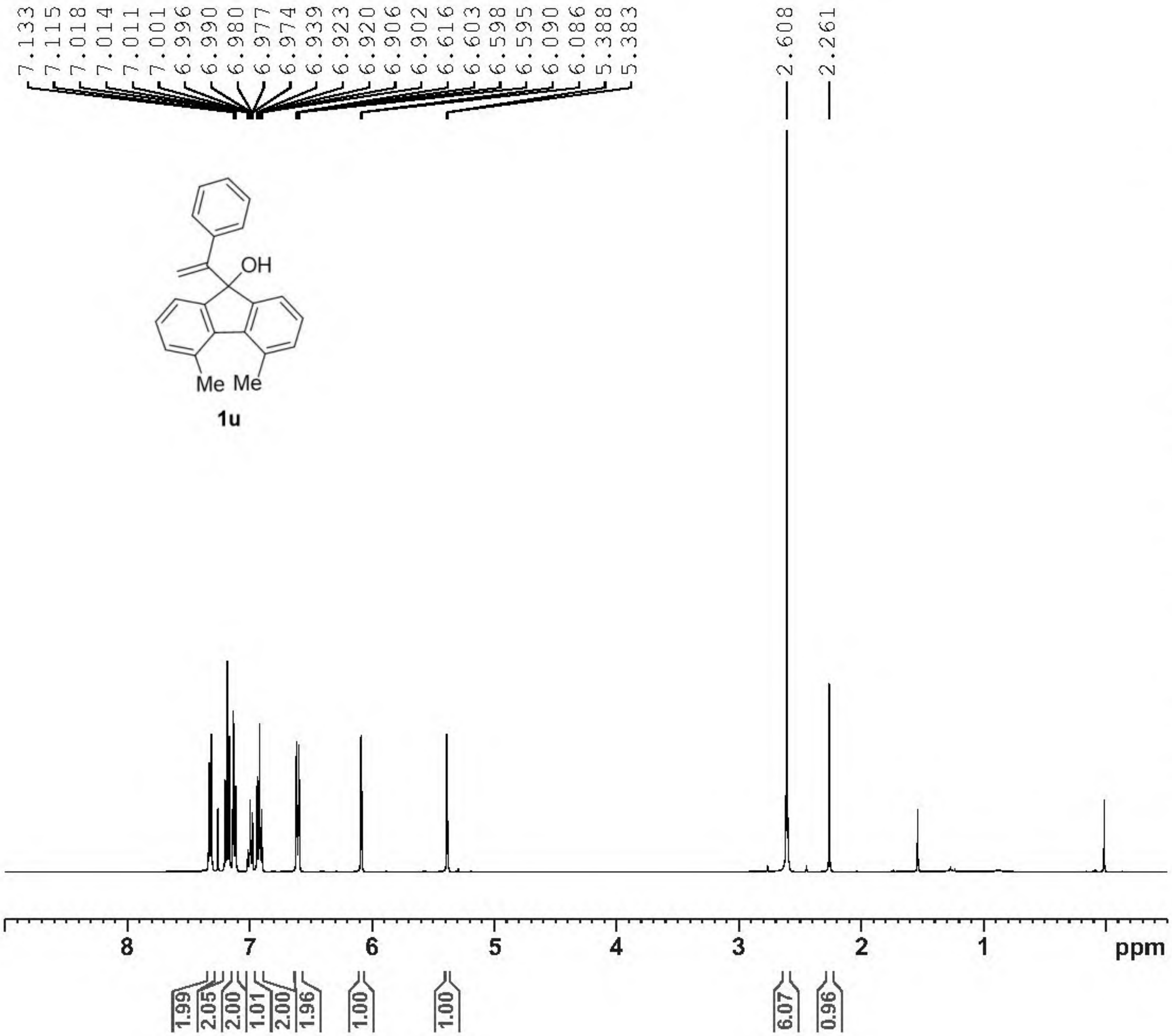

1u

```
Current Data Parameters
NAME      zzj-SM-axial-Me-H
EXPNO                    1
PROCNO                   1

F2 - Acquisition Parameters
Date_             20241019
Time                 11.17 h
INSTRUM   AvanceNeo 400MHz
PROBHD    Z163739_0629 (
PULPROG               zg30
TD                   65536
SOLVENT              CDCl3
NS                      16
DS                       2
SWH               8196.722 Hz
FIDRES            0.250144 Hz
AQ               3.9976959 sec
RG                     101
DW                  61.000 usec
DE                   13.89 usec
TE                   297.3 K
D1              1.00000000 sec
TD0                      1
SFO1           400.1824711 MHz
NUC1                    1H
P0                    2.67 usec
P1                    8.00 usec
PLW1           21.26700020 W

F2 - Processing parameters
SI                   65536
SF             400.1800097 MHz
WDW                     EM
SSB                      0
LB                    0.30 Hz
GB                       0
PC                    1.00
```

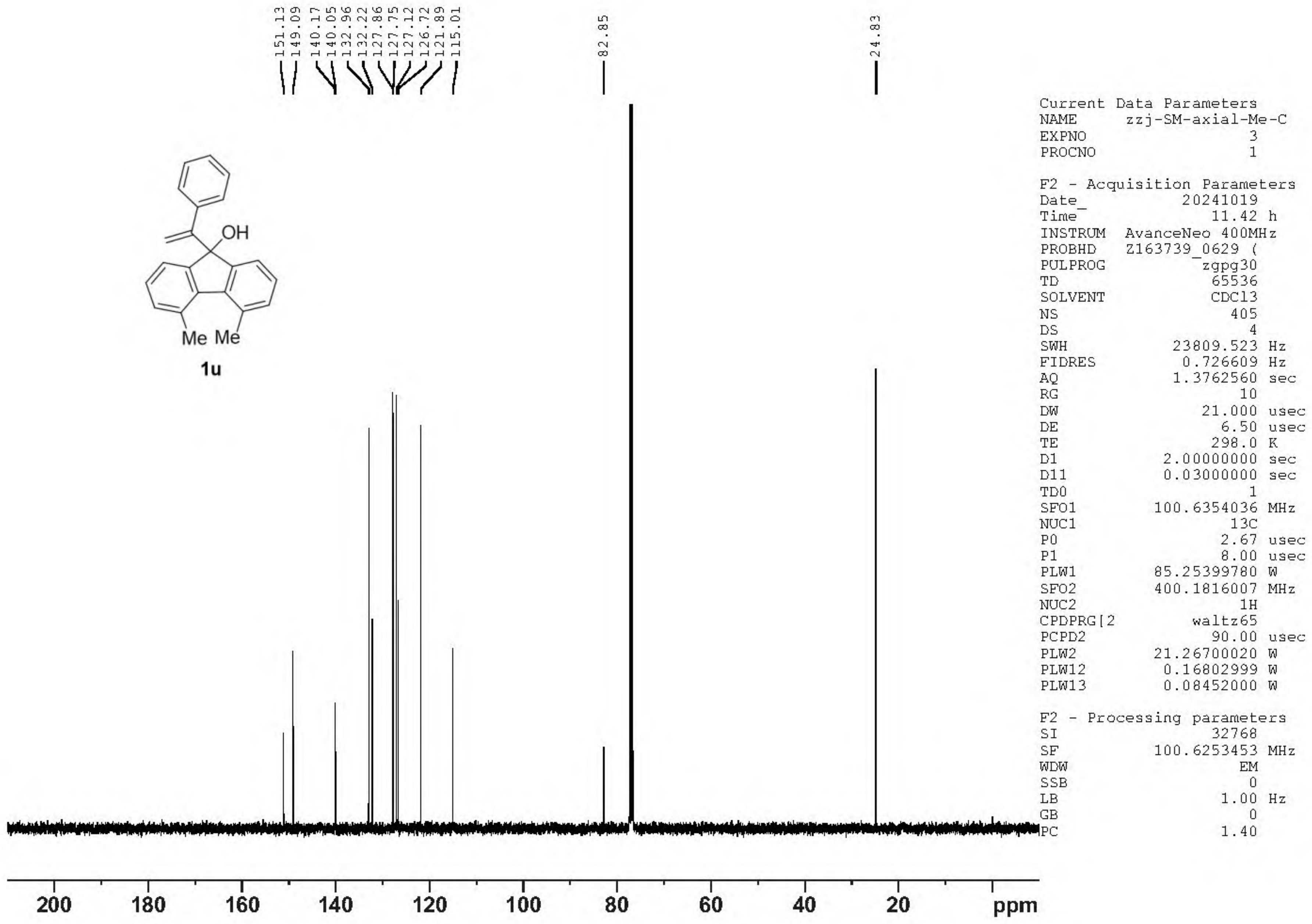
151.13
149.09
140.17
140.05
132.96
132.22
127.86
127.75
127.12
126.72
121.89
115.01
82.85
24.83
OH
Me Me
1u
200
180
160
140
120
100
80
60
40
20
ppm
Current Data Parameters
NAME zzj-SM-axial-Me-C
EXPNO 3
PROCNO 1
F2 - Acquisition Parameters
Date_ 20241019
Time_ 11.42 h
INSTRUM AvanceNeo 400MHz
PROBHD Z163739_0629 (
PULPROG zgpg30
TD 65536
SOLVENT CDCl3
NS 405
DS 4
SWH 23809.523 Hz
FIDRES 0.726609 Hz
AQ 1.3762560 sec
RG 10
DW 21.000 usec
DE 6.50 usec
TE 298.0 K
D1 2.00000000 sec
D11 0.03000000 sec
TD0 1
SFO1 100.6354036 MHz
NUC1 13C
P0 2.67 usec
P1 8.00 usec
PLW1 85.25399780 W
SFO2 400.1816007 MHz
NUC2 1H
CPDPRG[2 waltz65
PCPD2 90.00 usec
PLW2 21.26700020 W
PLW12 0.16802999 W
PLW13 0.08452000 W
F2 - Processing parameters
SI 32768
SF 100.6253453 MHz
WDW EM
SSB 0
LB 1.00 Hz
GB 0
PC 1.40

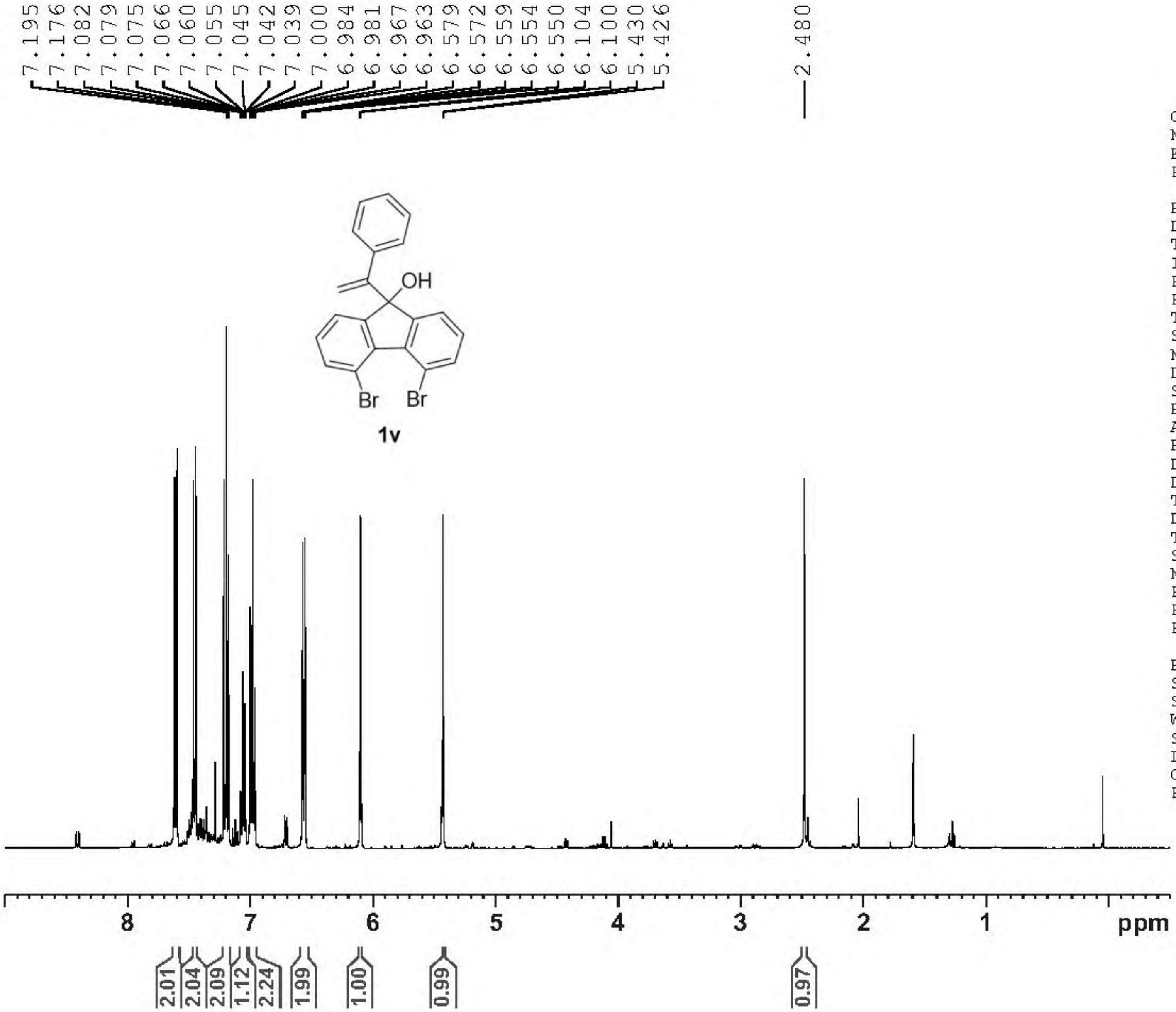

Current Data Parameters
NAME zzj-SM-axial-Br-C
EXPNO 1
PROCNO 1

F2 - Acquisition Parameters

Date_	20240821	
Time	20.56	h
INSTRUM	AvanceNeo 400MHz	
PROBHD	Z163739_0629 (	
PULPROG	zg30	
TD	65536	
SOLVENT	CDCl3	
NS	7	
DS	2	
SWH	8196.722	Hz
FIDRES	0.250144	Hz
AQ	3.9976959	sec
RG	101	
DW	61.000	usec
DE	13.89	usec
TE	296.7	K
D1	1.00000000	sec
TD0	1	
SFO1	400.1824711	MHz
NUC1	1H	
P0	2.67	usec
P1	8.00	usec
PLW1	21.26700020	W

F2 - Processing parameters

SI	65536	
SF	400.1800000	MHz
WDW	EM	
SSB	0	
LB	0.30	Hz
GB	0	
PC	1.00	

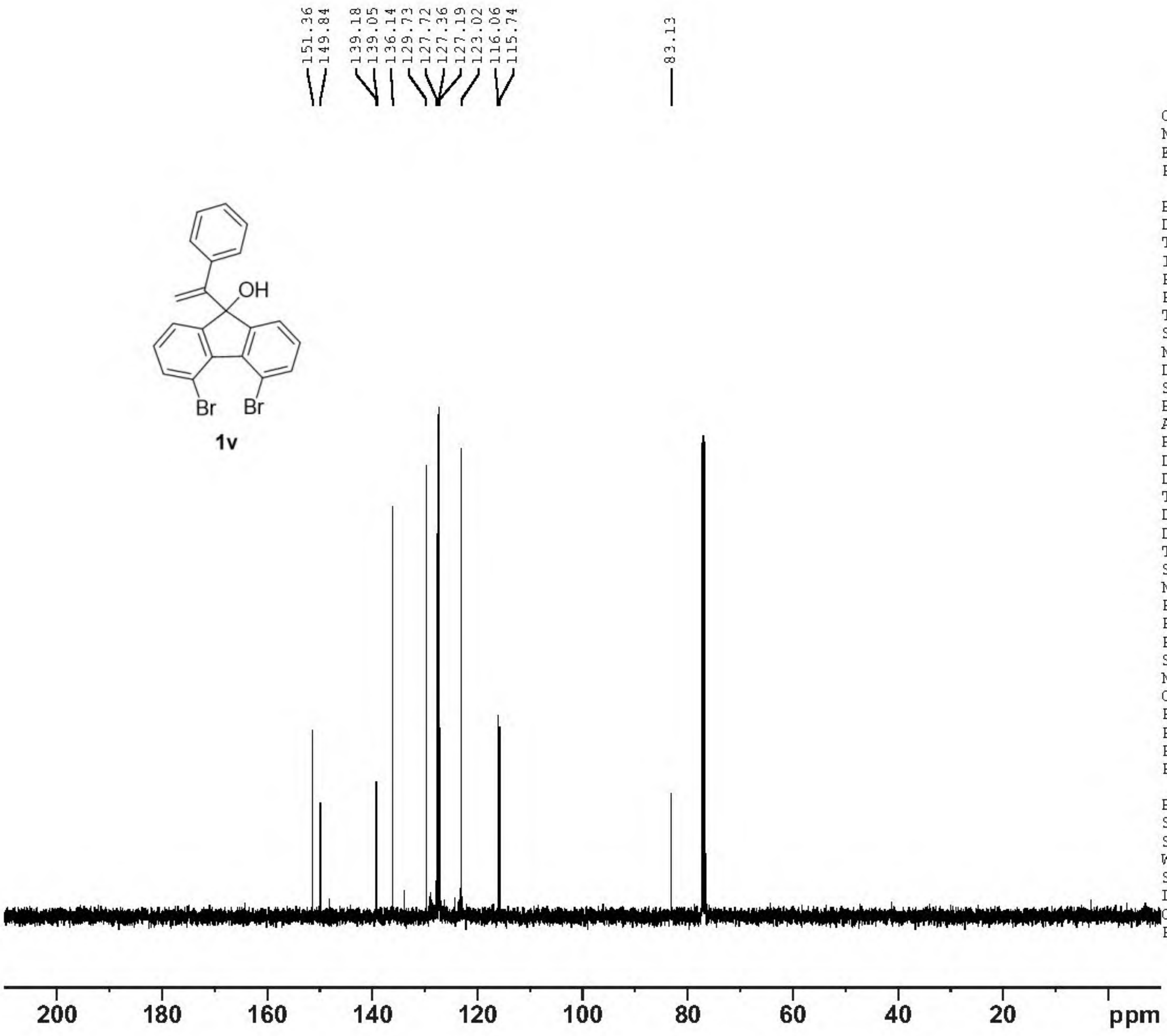


```
Current Data Parameters
NAME        zzj-SM-axial-Br-H
EXPNO                       3
PROCNO                      1

F2 - Acquisition Parameters
Date_              20240821
Time                  21.01 h
INSTRUM     AvanceNeo 400MHz
PROBHD      Z163739_0629 (
PULPROG             zgpg30
TD                   65536
SOLVENT              CDCl3
NS                      38
DS                       4
SWH              23809.523 Hz
FIDRES            0.726609 Hz
AQ               1.3762560 sec
RG                      10
DW                  21.000 usec
DE                    6.50 usec
TE                   297.3 K
D1              2.00000000 sec
D11             0.03000000 sec
TD0                      1
SFO1           100.6354036 MHz
NUC1                   13C
P0                    2.67 usec
P1                    8.00 usec
PLW1           85.25399780 W
SFO2           400.1816007 MHz
NUC2                    1H
CPDPRG[2           waltz65
PCPD2                90.00 usec
PLW2           21.26700020 W
PLW12           0.16802999 W
PLW13           0.08452000 W

F2 - Processing parameters
SI                   32768
SF             100.6253500 MHz
WDW                     EM
SSB                      0
LB                    1.00 Hz
GB                       0
PC                    1.40
```

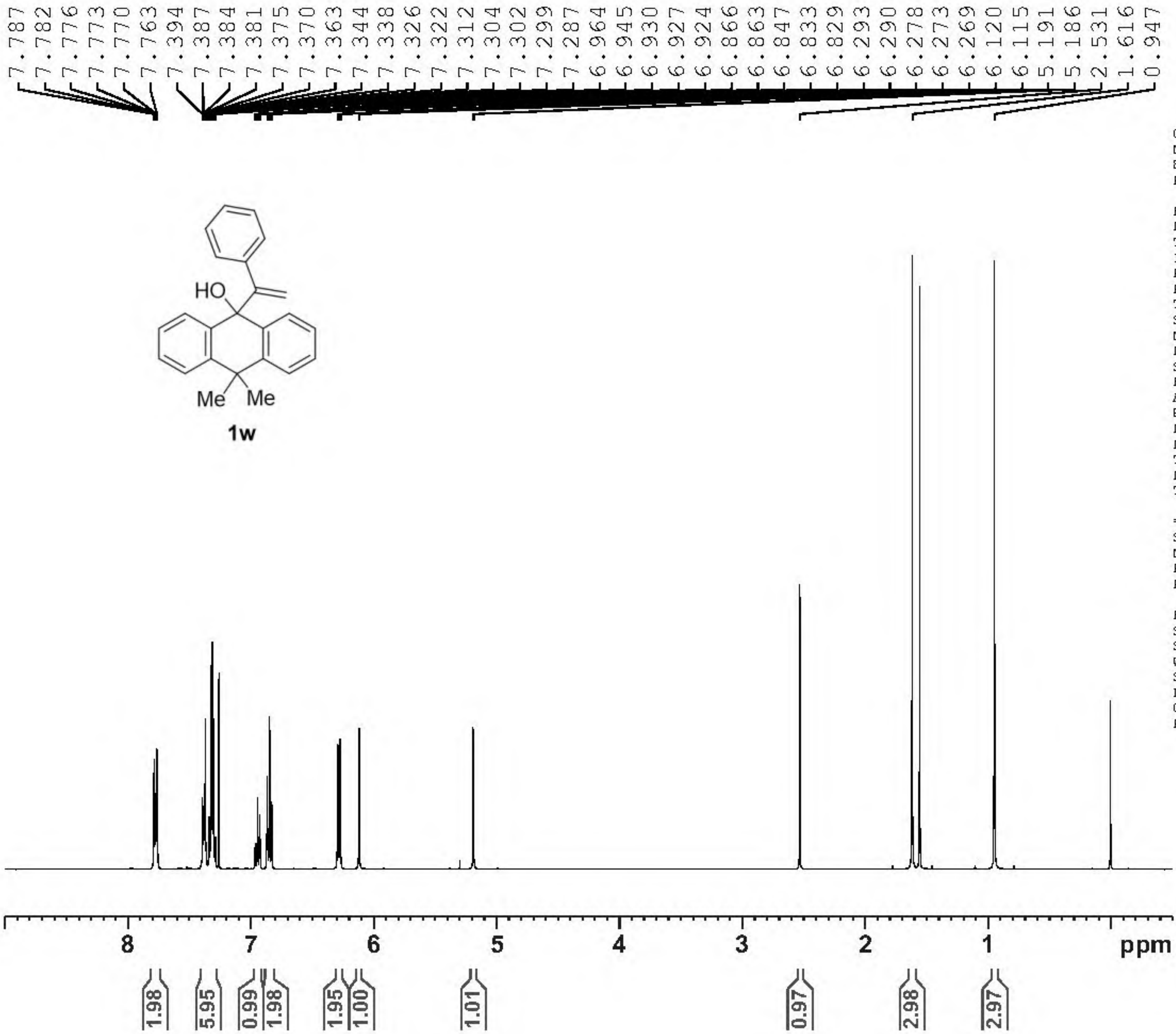

1w

```
Current Data Parameters
NAME      zzj-sm-7-membered ring-H
EXPNO                    1
PROCNO                   1

F2 - Acquisition Parameters
Date_             20240816
Time                 10.18
INSTRUM              spect
PROBHD   5 mm PABBO BB/
PULPROG               zg30
TD                   65536
SOLVENT              CDCl3
NS                      16
DS                       2
SWH               8012.820 Hz
FIDRES            0.122266 Hz
AQ               4.0894465 sec
RG                  126.97
DW                  62.400 usec
DE                    6.50 usec
TE                   296.1 K
D1              1.00000000 sec
TD0                      1

======== CHANNEL f1 ========
SFO1           400.1324710 MHz
NUC1                    1H
P1                   14.50 usec
PLW1           11.99499989 W

F2 - Processing parameters
SI                   65536
SF             400.1300094 MHz
WDW                     EM
SSB                      0
LB                    0.30 Hz
GB                       0
PC                    1.00
```

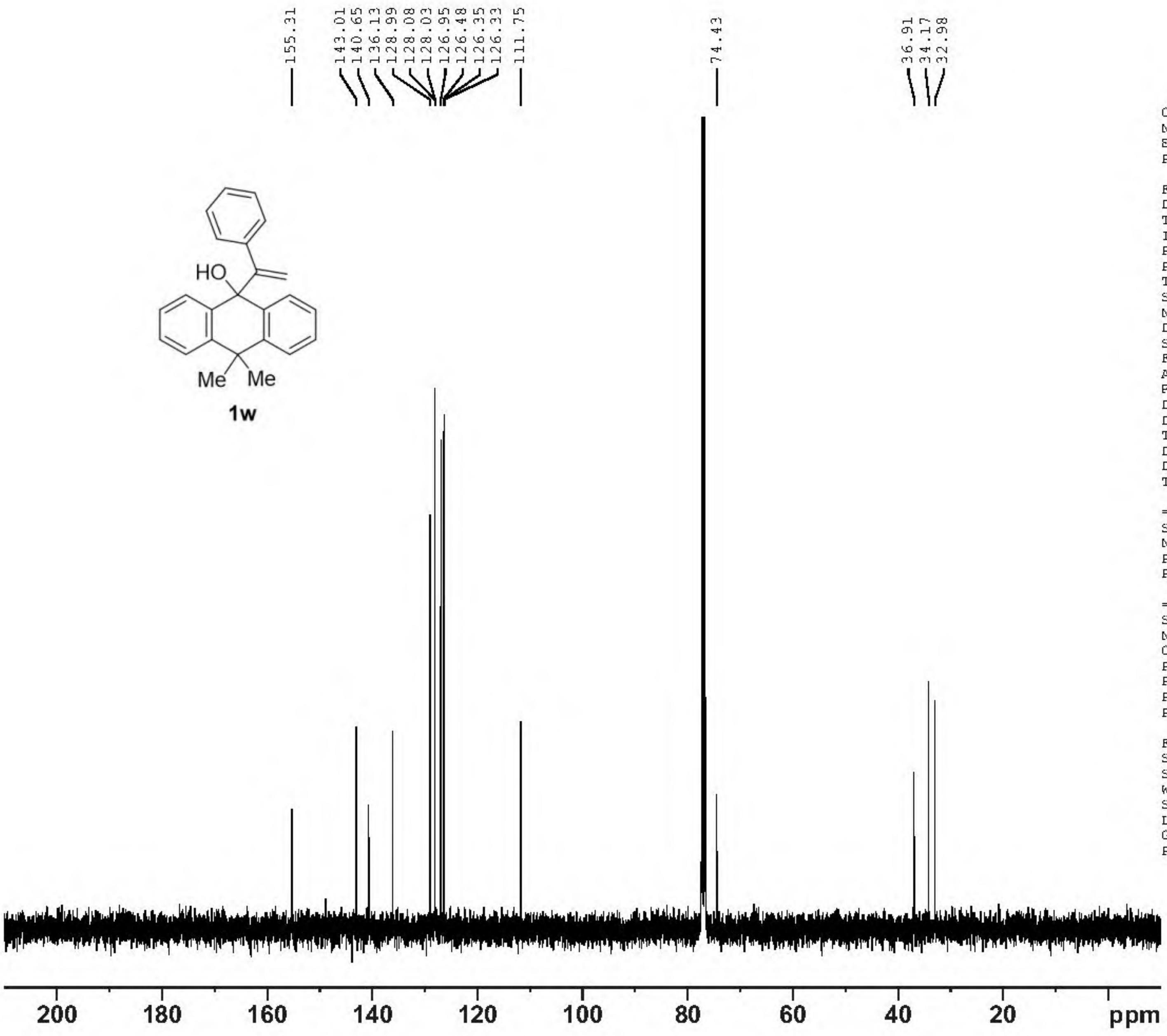

Current Data Parameters
NAME zzj-sm-7-membered ring-C
EXPNO 1
PROCNO 1

F2 - Acquisition Parameters
Date_ 20240816
Time 10.42
INSTRUM spect
PROBHD 5 mm PABBO BB/
PULPROG zgpg30
TD 65536
SOLVENT CDCl3
NS 400
DS 2
SWH 24038.461 Hz
FIDRES 0.366798 Hz
AQ 1.3631488 sec
RG 196.92
DW 20.800 usec
DE 6.50 usec
TE 297.2 K
D1 2.00000000 sec
D11 0.03000000 sec
TD0 1

======== CHANNEL f1 ========
SFO1 100.6228298 MHz
NUC1 13C
P1 9.70 usec
PLW1 46.98899841 W

======== CHANNEL f2 ========
SFO2 400.1316005 MHz
NUC2 1H
CPDPRG[2 waltz16
PCPD2 90.00 usec
PLW2 11.99499989 W
PLW12 0.34213999 W
PLW13 0.27713001 W

F2 - Processing parameters
SI 32768
SF 100.6127713 MHz
WDW EM
SSB 0
LB 1.00 Hz
GB 0
PC 1.40

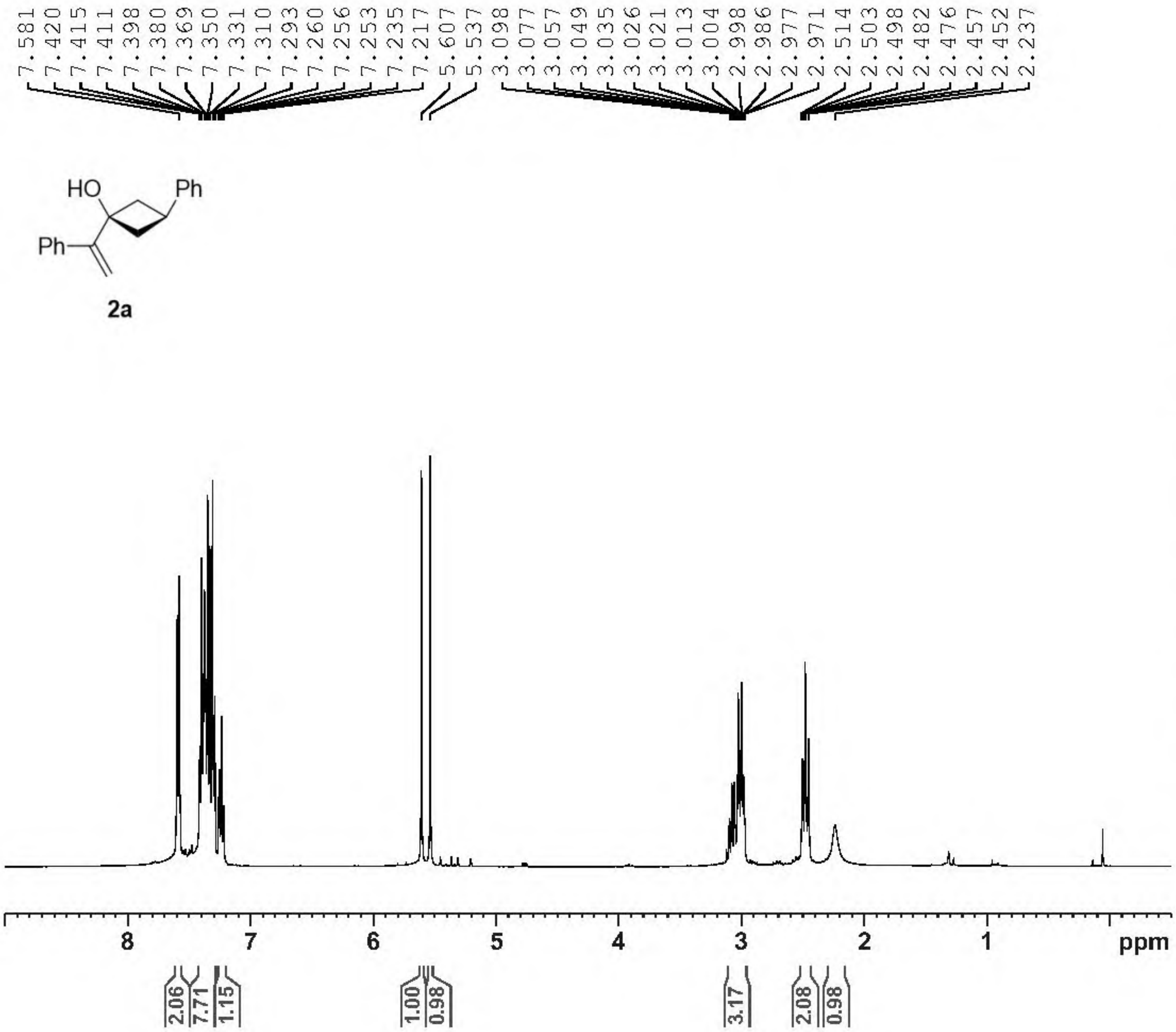


```
Current Data Parameters
NAME       zzj-4-mem-SM-Ph-H
EXPNO                      1
PROCNO                     1

F2 - Acquisition Parameters
Date_              20241216
Time                  22.49
INSTRUM               spect
PROBHD    5 mm PABBO BB/
PULPROG                zg30
TD                    65536
SOLVENT               CDCl3
NS                       16
DS                        2
SWH             8012.820 Hz
FIDRES          0.122266 Hz
AQ             4.0894465 sec
RG                    27.78
DW               62.400 usec
DE                 6.50 usec
TE                  295.2 K
D1            1.00000000 sec
TD0                       1

======== CHANNEL f1 ========
SFO1        400.1324710 MHz
NUC1                     1H
P1                14.50 usec
PLW1          11.99499989 W

F2 - Processing parameters
SI                    65536
SF          400.1300096 MHz
WDW                      EM
SSB                       0
LB                  0.30 Hz
GB                        0
PC                     1.00
```

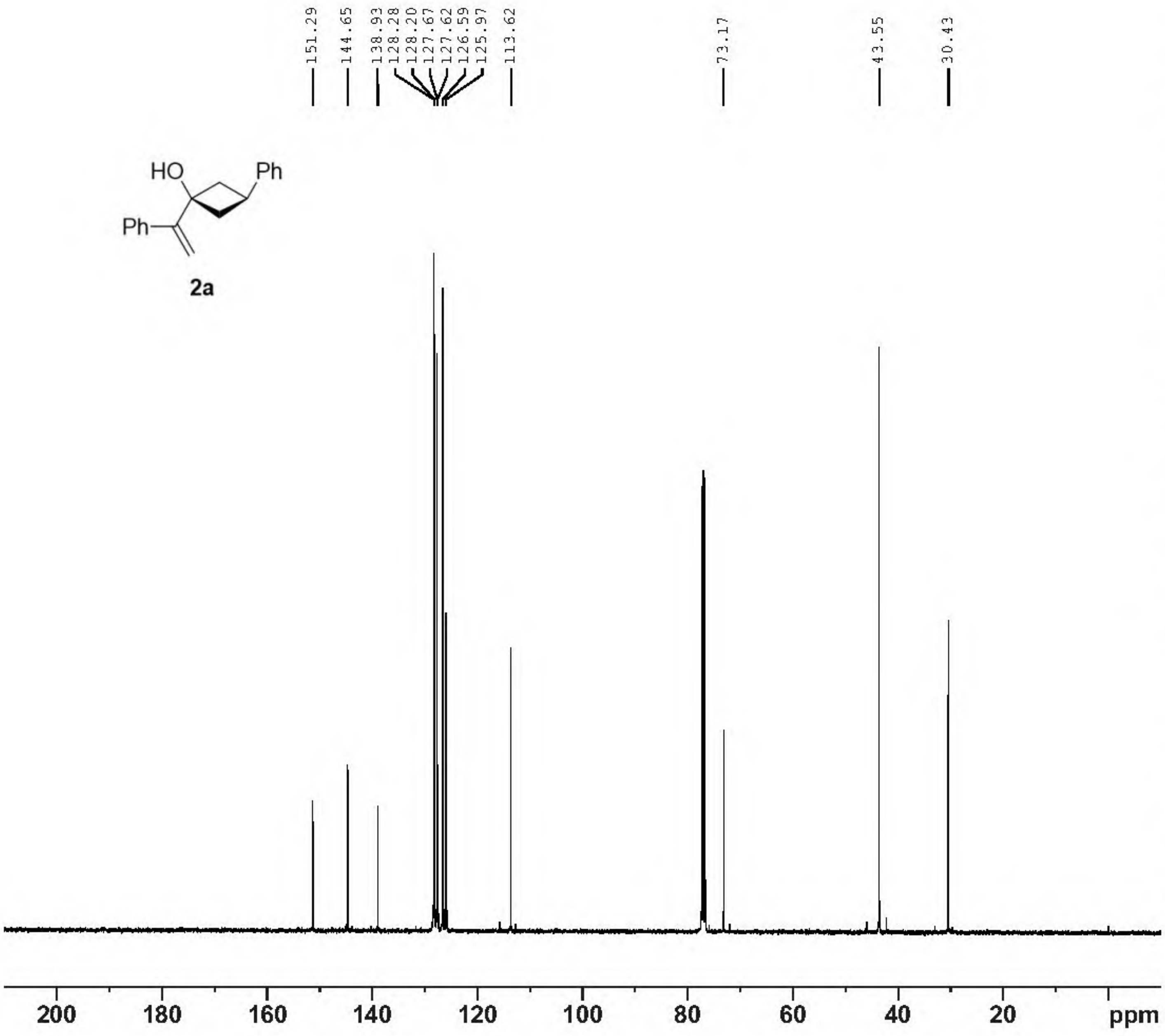


```
Current Data Parameters
NAME      zzj-4-mem-SM-Ph-C
EXPNO                     1
PROCNO                    1

F2 - Acquisition Parameters
Date_              20241216
Time                  23.36
INSTRUM               spect
PROBHD    5 mm PABBO BB/
PULPROG             zgpg30
TD                   65536
SOLVENT              CDCl3
NS                     800
DS                       2
SWH              24038.461 Hz
FIDRES            0.366798 Hz
AQ               1.3631488 sec
RG                  196.92
DW                  20.800 usec
DE                    6.50 usec
TE                   296.5 K
D1              2.00000000 sec
D11             0.03000000 sec
TD0                      1

======== CHANNEL f1 ========
SFO1           100.6228298 MHz
NUC1                   13C
P1                     9.70 usec
PLW1           46.98899841 W

======== CHANNEL f2 ========
SFO2           400.1316005 MHz
NUC2                    1H
CPDPRG[2           waltz16
PCPD2                90.00 usec
PLW2           11.99499989 W
PLW12           0.34213999 W
PLW13           0.27713001 W

F2 - Processing parameters
SI                   32768
SF             100.6127812 MHz
WDW                     EM
SSB                      0
LB                    1.00 Hz
GB                       0
PC                    1.40
```

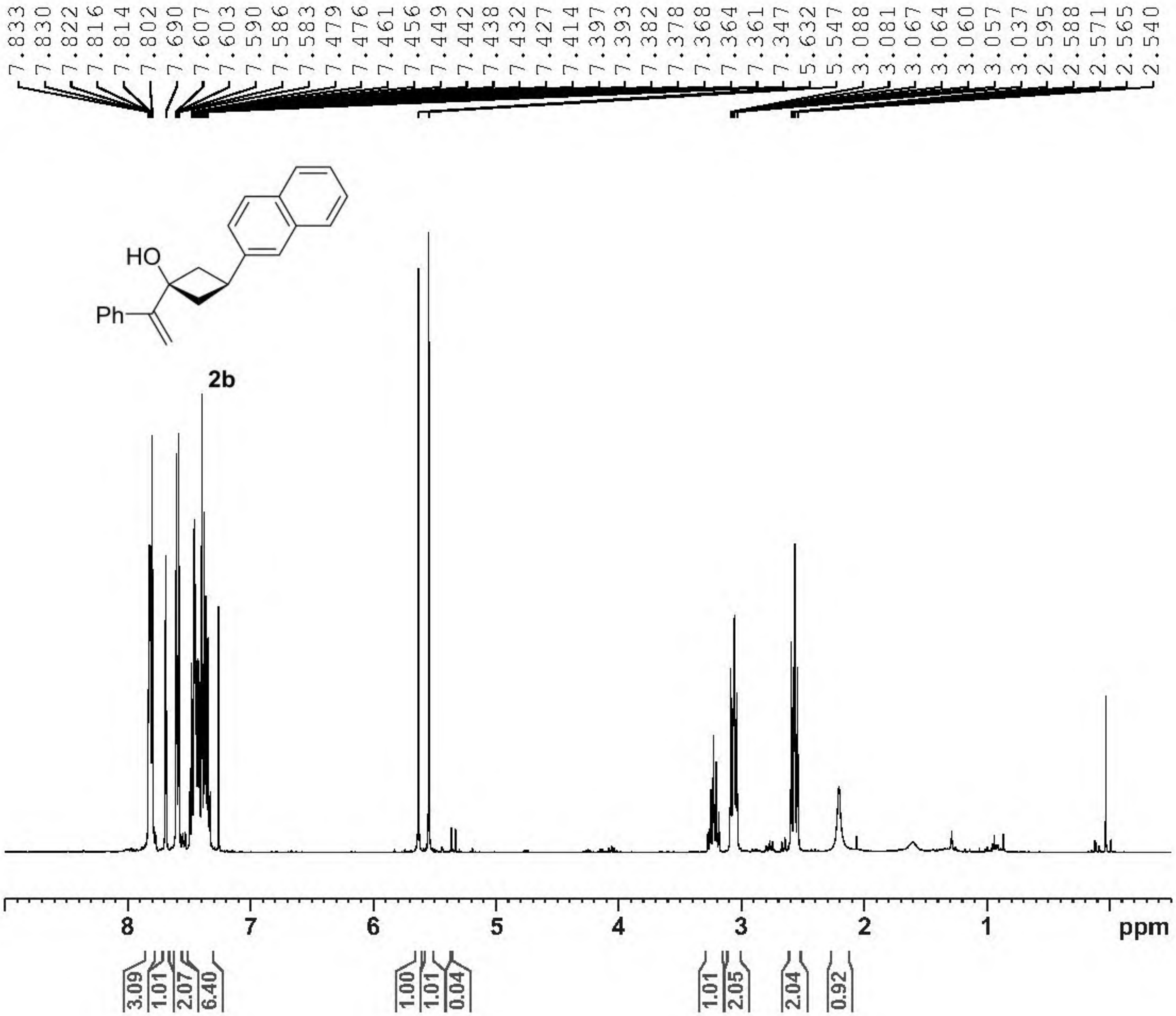

Current Data Parameters
NAME zzj-4-mem-SM-Ph-Nap-H
EXPNO 1
PROCNO 1

F2 - Acquisition Parameters
Date_ 20260509
Time 11.35
INSTRUM spect
PROBHD 5 mm PABBO BB/
PULPROG zg30
TD 65536
SOLVENT CDCl3
NS 16
DS 2
SWH 8012.820 Hz
FIDRES 0.122266 Hz
AQ 4.0894465 sec
RG 70.97
DW 62.400 usec
DE 6.50 usec
TE 297.0 K
D1 1.00000000 sec
TD0 1

======== CHANNEL f1 ========
SFO1 400.1324710 MHz
NUC1 1H
P1 14.50 usec
PLW1 11.99499989 W

F2 - Processing parameters
SI 65536
SF 400.1300096 MHz
WDW EM
SSB 0
LB 0.30 Hz
GB 0
PC 1.00

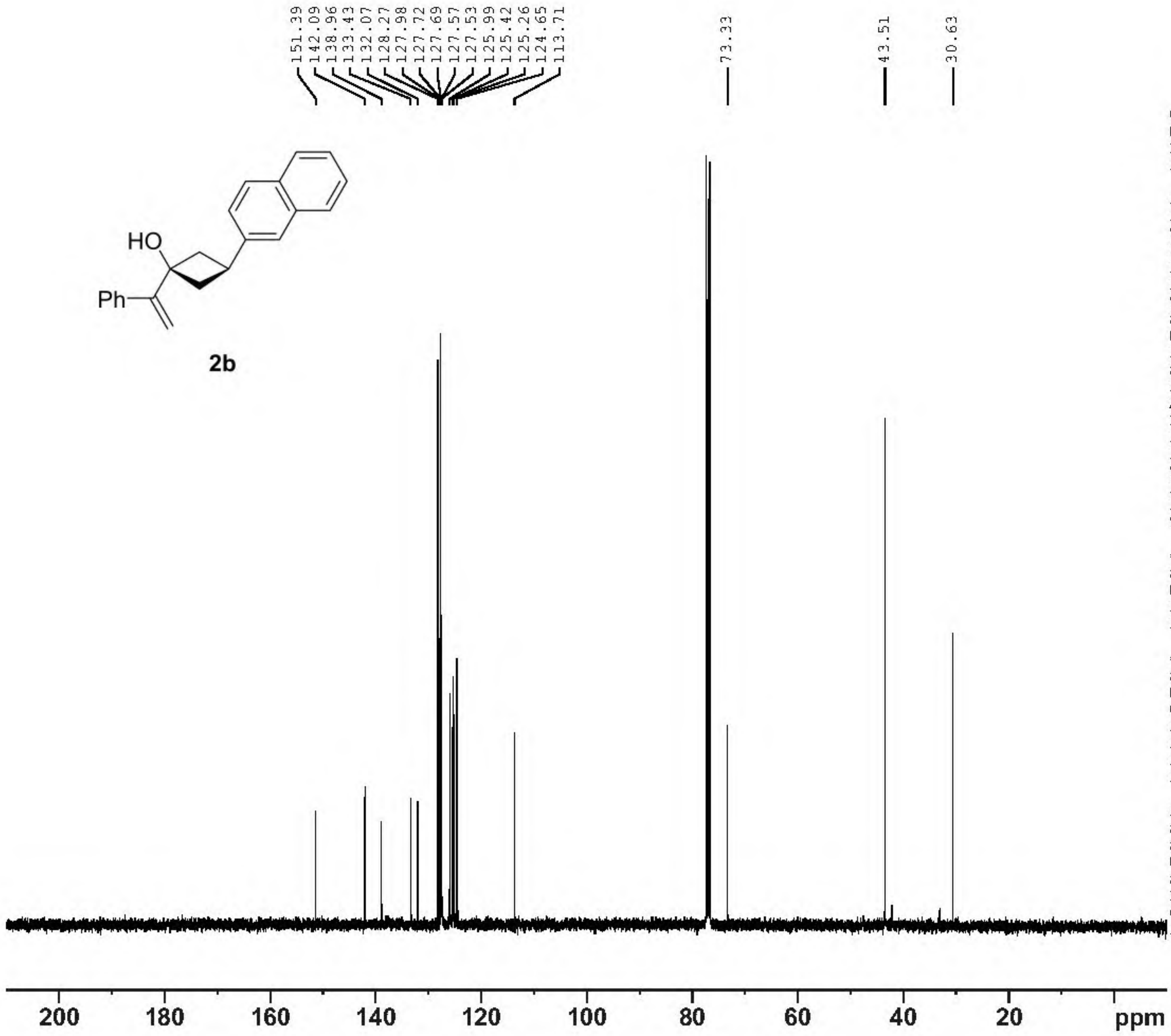

2b

Current Data Parameters
NAME zzj-4-mem-SM-Ph-Nap-C
EXPNO 1
PROCNO 1

F2 - Acquisition Parameters
Date_ 20260509
Time_ 11.47
INSTRUM spect
PROBHD 5 mm PABBO BB/
PULPROG zgpg30
TD 65536
SOLVENT CDCl3
NS 200
DS 2
SWH 24038.461 Hz
FIDRES 0.366798 Hz
AQ 1.3631488 sec
RG 196.92
DW 20.800 usec
DE 6.50 usec
TE 297.7 K
D1 2.00000000 sec
D11 0.03000000 sec
TD0 1

======== CHANNEL f1 ========
SFO1 100.6228298 MHz
NUC1 13C
P1 9.70 usec
PLW1 46.98899841 W

======== CHANNEL f2 ========
SFO2 400.1316005 MHz
NUC2 1H
CPDPRG[2 waltz16
PCPD2 90.00 usec
PLW2 11.99499989 W
PLW12 0.34213999 W
PLW13 0.27713001 W

F2 - Processing parameters
SI 32768
SF 100.6127756 MHz
WDW EM
SSB 0
LB 1.00 Hz
GB 0
PC 1.40

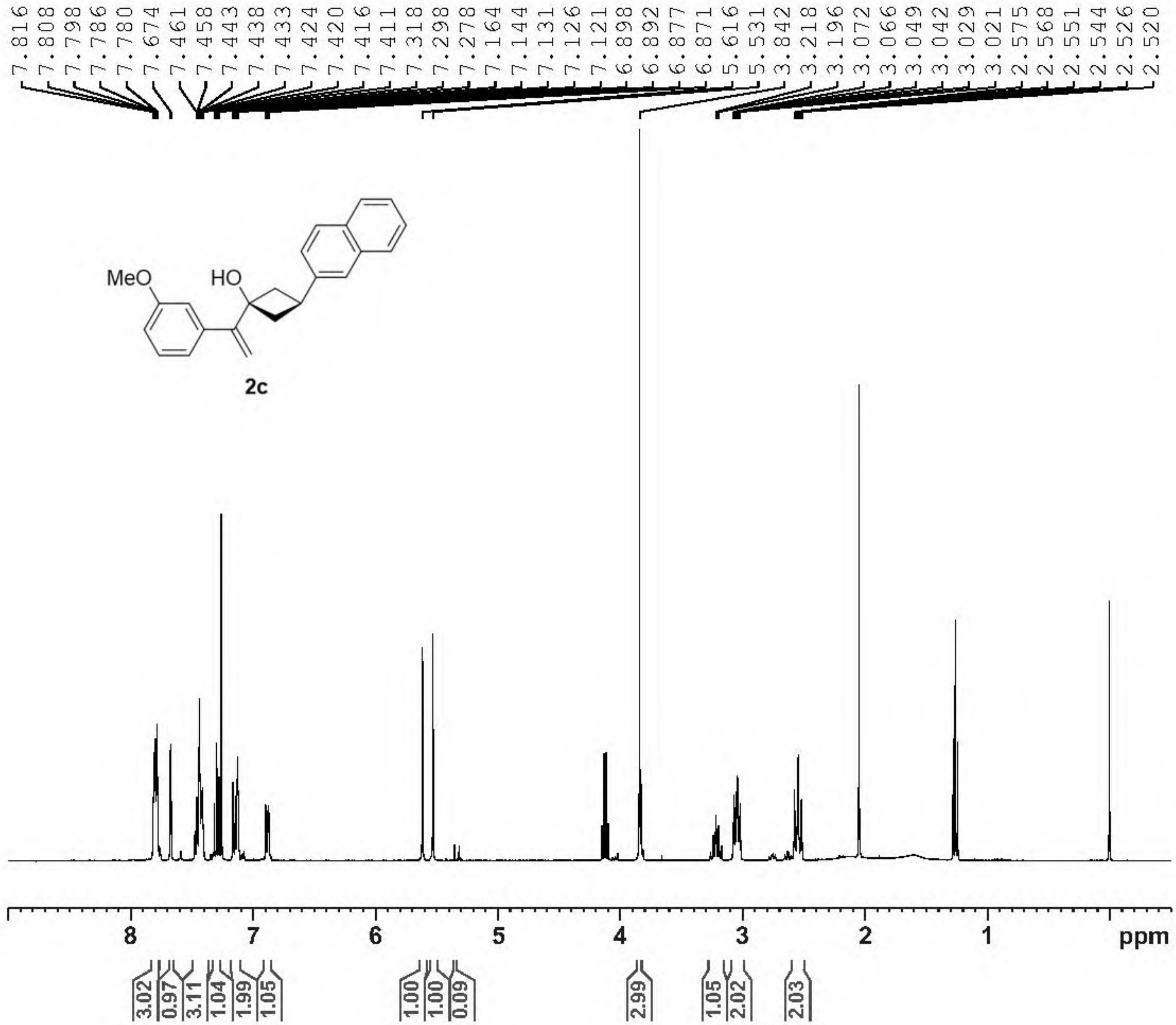

Current Data Parameters	
NAME	zzj-7-86-H
EXPNO	1
PROCNO	1
F2 - Acquisition Parameters	
Date_	20260314
Time	1.18
INSTRUM	spect
PROBHD	5 mm PABBO BB/
PULPROG	zg30
TD	65536
SOLVENT	CDCl3
NS	16
DS	2
SWH	8012.820 Hz
FIDRES	0.122266 Hz
AQ	4.0894465 sec
RG	126.97
DW	62.400 usec
DE	6.50 usec
TE	295.8 K
D1	1.00000000 sec
TD0	1
======== CHANNEL f1 ========	
SFO1	400.1324710 MHz
NUC1	1H
P1	14.50 usec
PLW1	11.99499989 W
F2 - Processing parameters	
SI	65536
SF	400.1300098 MHz
WDW	EM
SSB	0
LB	0.30 Hz
GB	0
PC	1.00

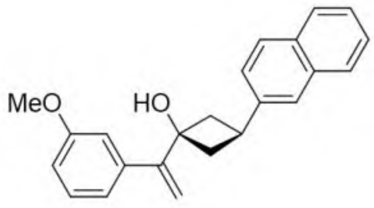

2c

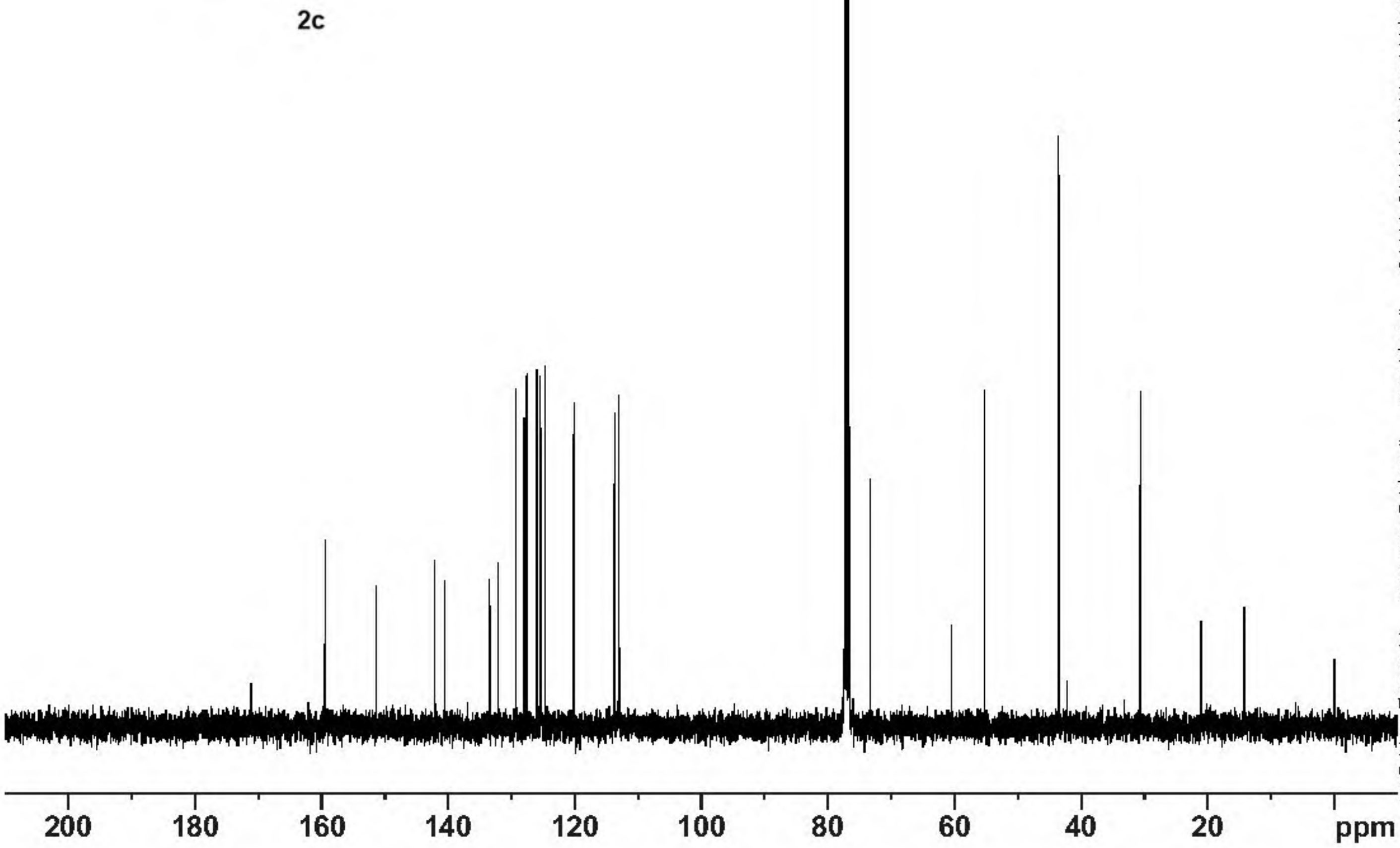


```
Current Data Parameters
NAME          zzj-7-86-C
EXPNO                  1
PROCNO                 1

F2 - Acquisition Parameters
Date_           20260314
Time_               2.16
INSTRUM            spect
PROBHD   5 mm PABBO BB/
PULPROG          zgpg30
TD                 65536
SOLVENT            CDCl3
NS                  1000
DS                     2
SWH            24038.461 Hz
FIDRES          0.366798 Hz
AQ             1.3631488 sec
RG                196.92
DW                20.800 usec
DE                  6.50 usec
TE                 296.7 K
D1            2.00000000 sec
D11           0.03000000 sec
TD0                    1

======== CHANNEL f1 ========
SFO1         100.6228298 MHz
NUC1                 13C
P1                  9.70 usec
PLW1         46.98899841 W

======== CHANNEL f2 ========
SFO2         400.1316005 MHz
NUC2                  1H
CPDPRG[2         waltz16
PCPD2              90.00 usec
PLW2         11.99499989 W
PLW12         0.34213999 W
PLW13         0.27713001 W

F2 - Processing parameters
SI                 32768
SF           100.6127721 MHz
WDW                   EM
SSB                    0
LB                  1.00 Hz
GB                     0
PC                  1.40
```

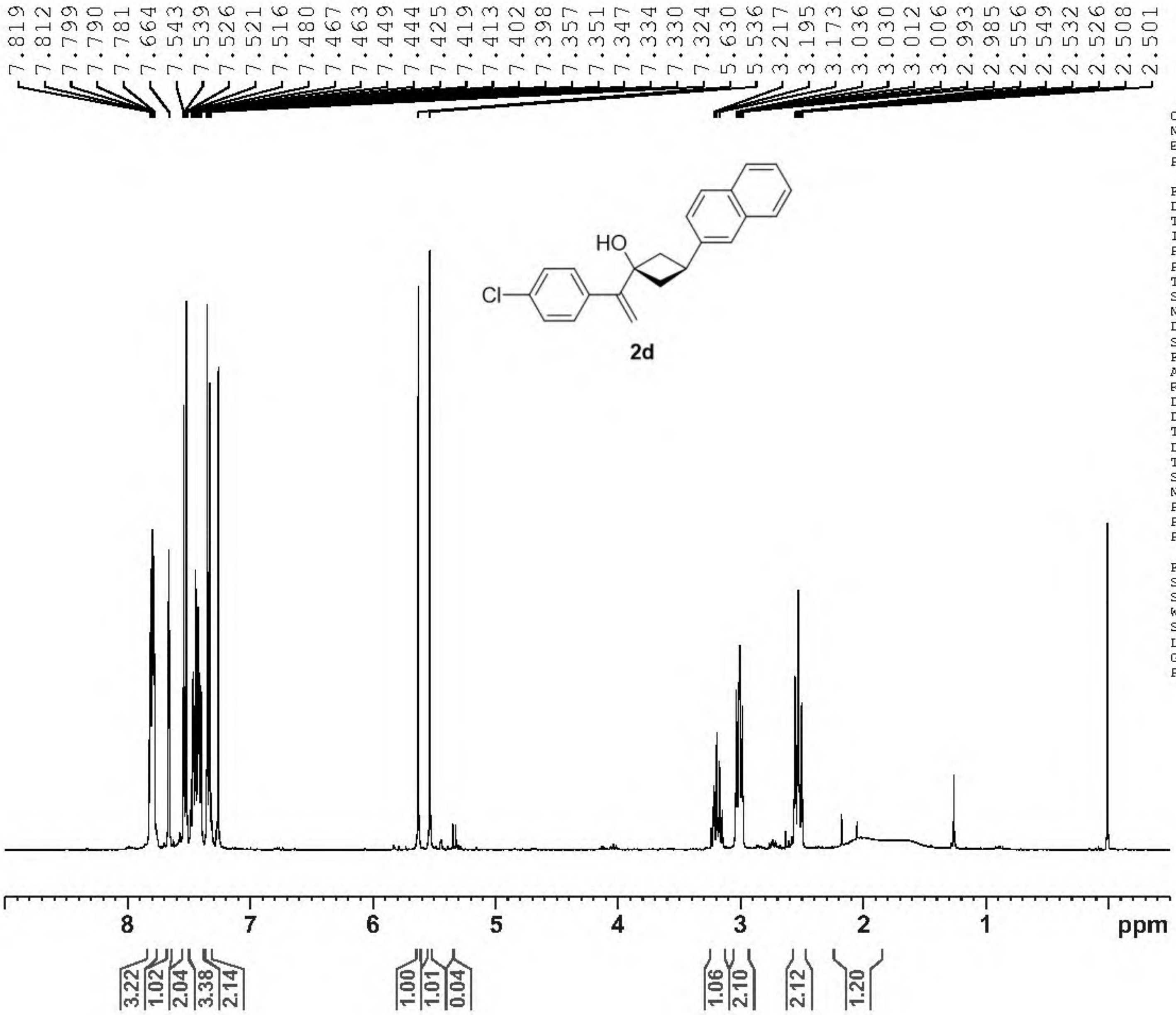


```
Current Data Parameters
NAME      zzj-4-mem-SM-pCl-Ph-Nap-H
EXPNO                  1
PROCNO                 1

F2 - Acquisition Parameters
Date_           20260508
Time               20.12 h
INSTRUM  AvanceNeo 400MHz
PROBHD   Z163739_0629 (
PULPROG             zg30
TD                 65536
SOLVENT            CDCl3
NS                     7
DS                     2
SWH            8196.722 Hz
FIDRES         0.250144 Hz
AQ            3.9976959 sec
RG                   101
DW                61.000 usec
DE                 13.89 usec
TE                 297.0 K
D1            1.00000000 sec
TD0                    1
SFO1       400.1824711 MHz
NUC1                  1H
P0                  2.67 usec
P1                  8.00 usec
PLW1         21.26700020 W

F2 - Processing parameters
SI                 65536
SF         400.1800098 MHz
WDW                   EM
SSB                    0
LB                  0.30 Hz
GB                     0
PC                  1.00
```

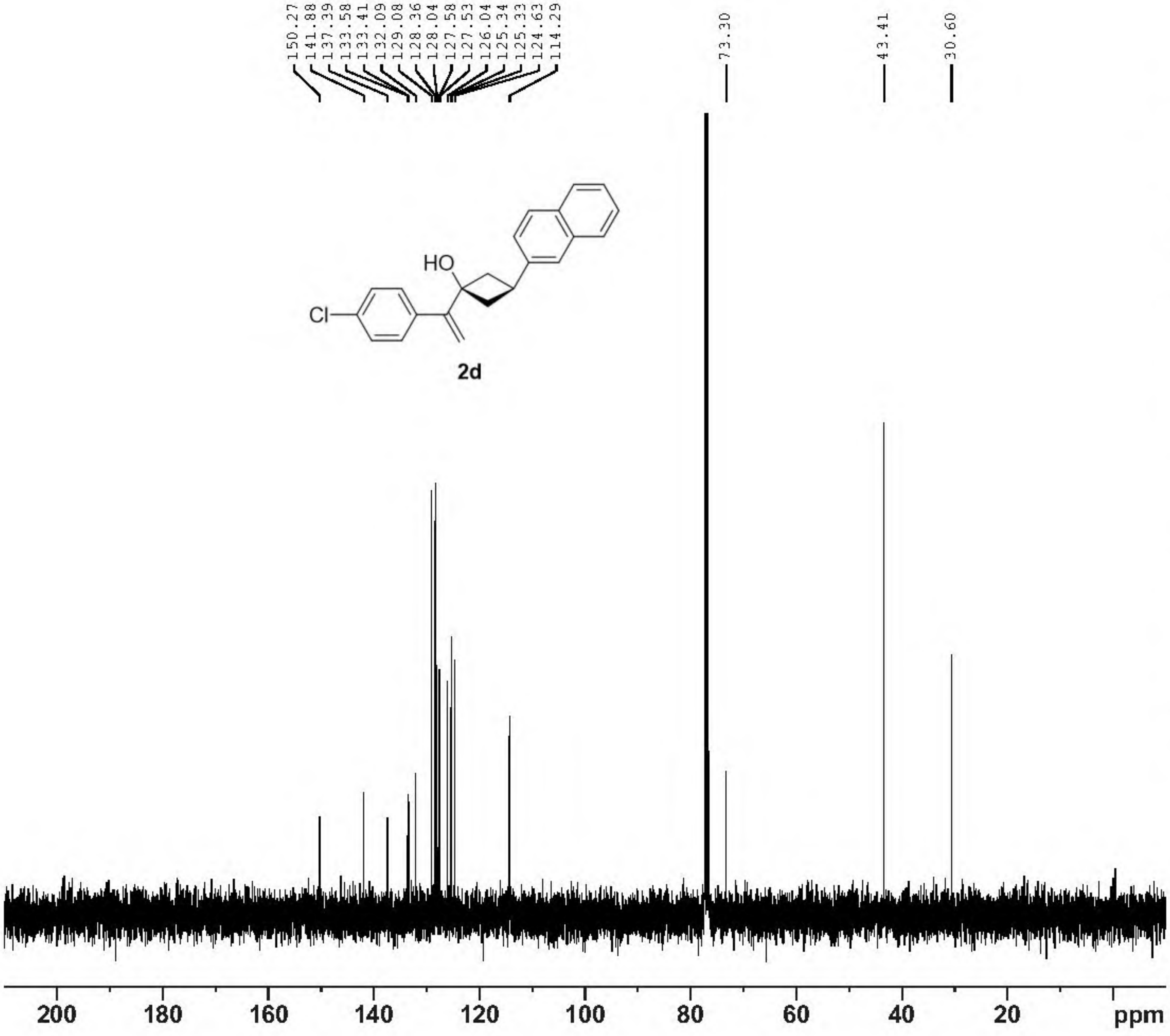


```
Current Data Parameters
NAME     zzj-4-mem-SM-pCl-Ph-Nap-C
EXPNO                  3
PROCNO                 1

F2 - Acquisition Parameters
Date_          20260508
Time              20.17 h
INSTRUM  AvanceNeo 400MHz
PROBHD   Z163739_0629 (
PULPROG          zgpg30
TD                65536
SOLVENT           CDCl3
NS                   70
DS                    4
SWH           23809.523 Hz
FIDRES         0.726609 Hz
AQ            1.3762560 sec
RG                   16
DW               21.000 usec
DE                 6.50 usec
TE                297.5 K
D1           2.00000000 sec
D11          0.03000000 sec
TD0                   1
SFO1        100.6354036 MHz
NUC1                13C
P0                 2.67 usec
P1                 8.00 usec
PLW1        85.25399780 W
SFO2        400.1816007 MHz
NUC2                 1H
CPDPRG[2        waltz65
PCPD2             90.00 usec
PLW2        21.26700020 W
PLW12        0.16802999 W
PLW13        0.08452000 W

F2 - Processing parameters
SI                32768
SF          100.6253457 MHz
WDW                  EM
SSB                   0
LB                 1.00 Hz
GB                    0
PC                 1.40
```

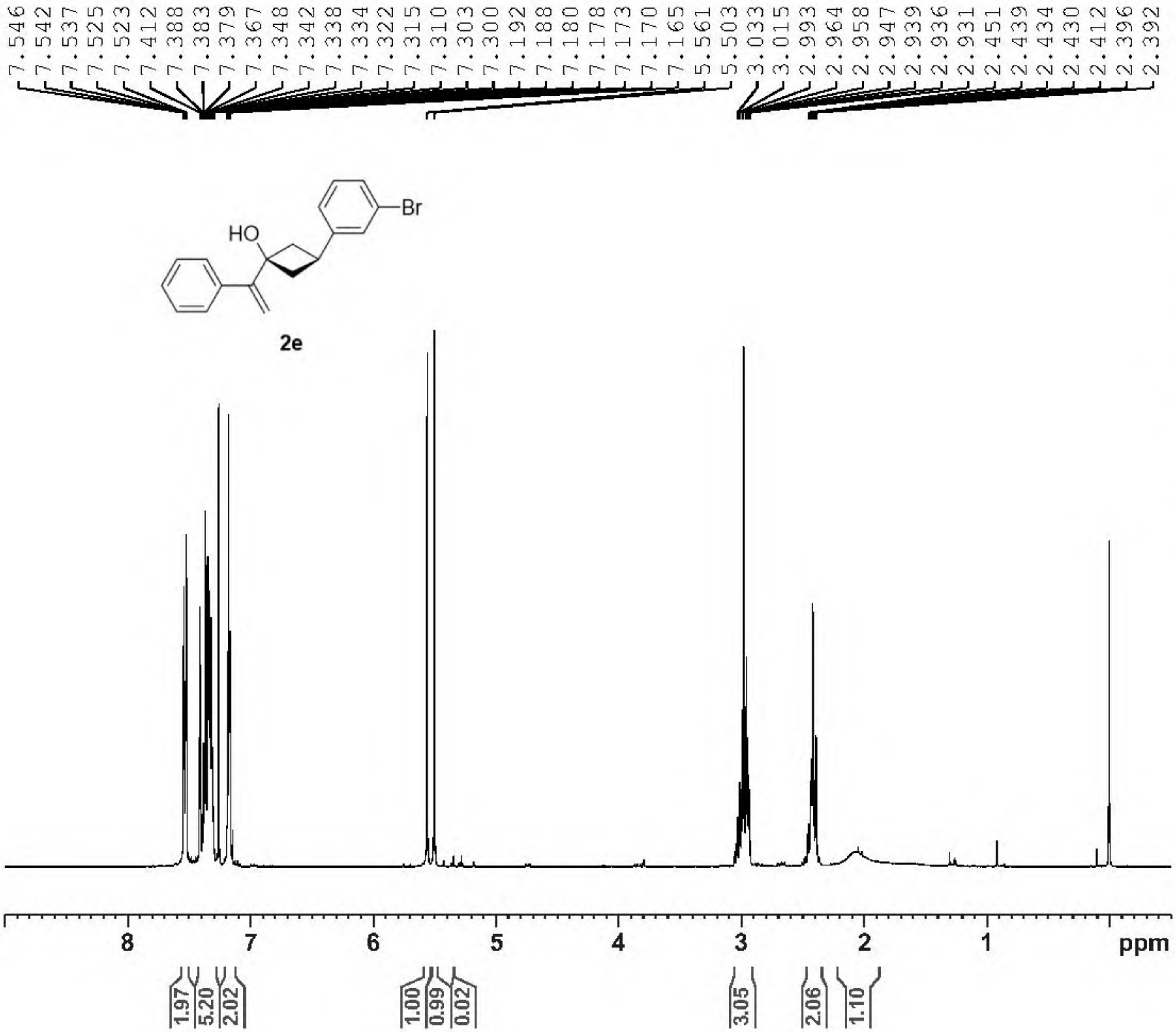


```
Current Data Parameters
NAME      zzj-4-mem-SM-alkene Ph-m-Br-Ph-H
EXPNO                  1
PROCNO                 1

F2 - Acquisition Parameters
Date_           20260402
Time               19.42
INSTRUM            spect
PROBHD   5 mm PABBO BB/
PULPROG             zg30
TD                 65536
SOLVENT            CDCl3
NS                    16
DS                     2
SWH             8012.820 Hz
FIDRES          0.122266 Hz
AQ             4.0894465 sec
RG                126.97
DW                62.400 usec
DE                  6.50 usec
TE                 296.1 K
D1            1.00000000 sec
TD0                    1

======== CHANNEL f1 ========
SFO1         400.1324710 MHz
NUC1                  1H
P1                 14.50 usec
PLW1         11.99499989 W

F2 - Processing parameters
SI                 65536
SF           400.1300099 MHz
WDW                   EM
SSB                    0
LB                  0.30 Hz
GB                     0
PC                  1.00
```

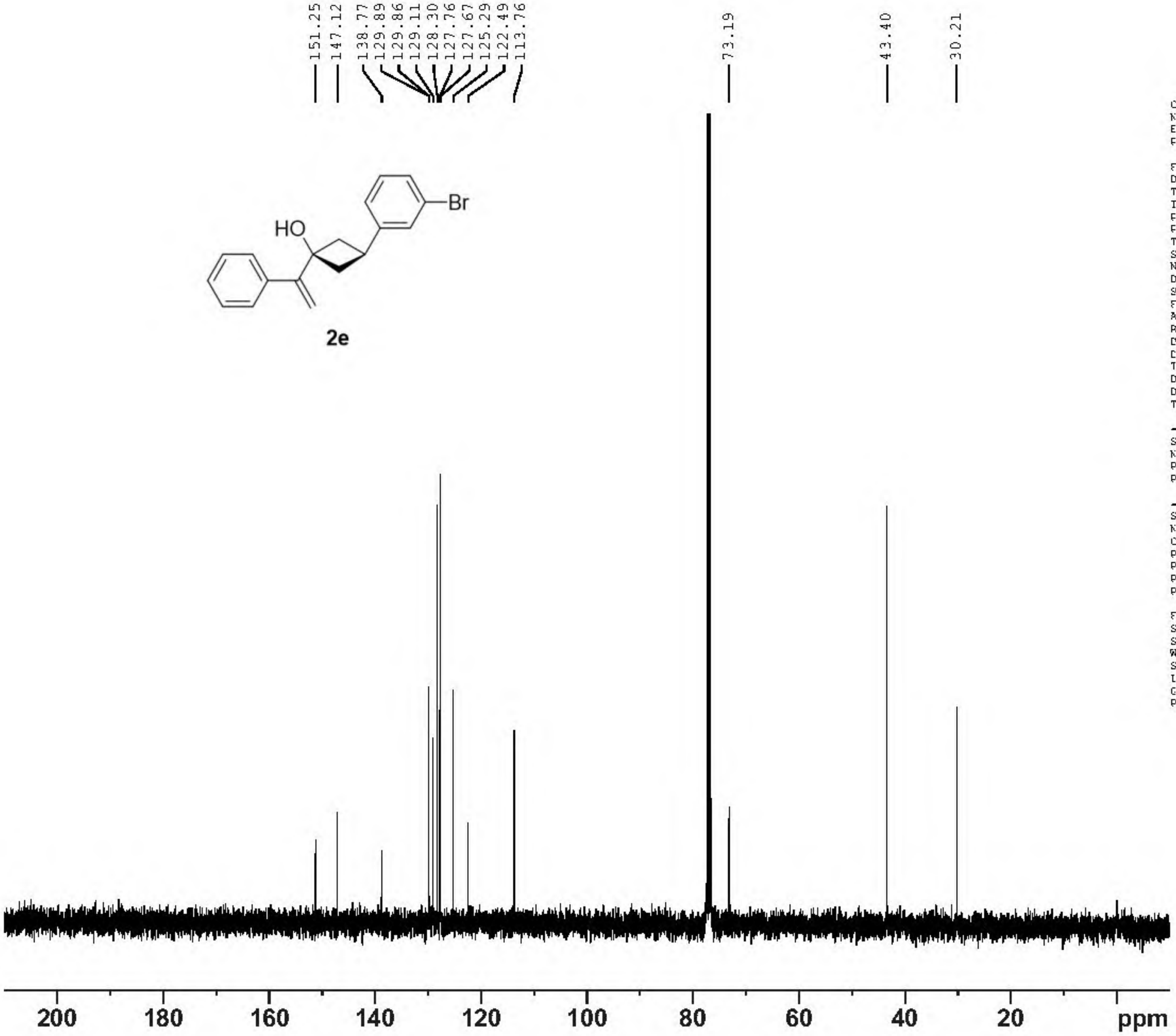


```
Current Data Parameters
NAME     zzj-4-mem-SM-alkene Ph-m-Br-Ph-C
EXPNO                  1
PROCNO                 1

F2 - Acquisition Parameters
Date_           20260402
Time               19.47
INSTRUM            spect
PROBHD   5 mm PABBO BB/
PULPROG          zgpg30
TD                 65536
SOLVENT            CDCl3
NS                   275
DS                     2
SWH           24038.461 Hz
FIDRES         0.366798 Hz
AQ            1.3631488 sec
RG                196.92
DW                20.800 usec
DE                  6.50 usec
TE                 296.8 K
D1            2.00000000 sec
D11           0.03000000 sec
TD0                    1

======== CHANNEL f1 ========
SFO1         100.6228298 MHz
NUC1                 13C
P1                  9.70 usec
PLW1         46.98899841 W

======== CHANNEL f2 ========
SFO2         400.1316005 MHz
NUC2                  1H
CPDPRG[2         waltz16
PCPD2              90.00 usec
PLW2         11.99499989 W
PLW12         0.34213999 W
PLW13         0.27713001 W

F2 - Processing parameters
SI                 32768
SF           100.6127720 MHz
WDW                   EM
SSB                    0
LB                  1.00 Hz
GB                     0
PC                  1.40
```

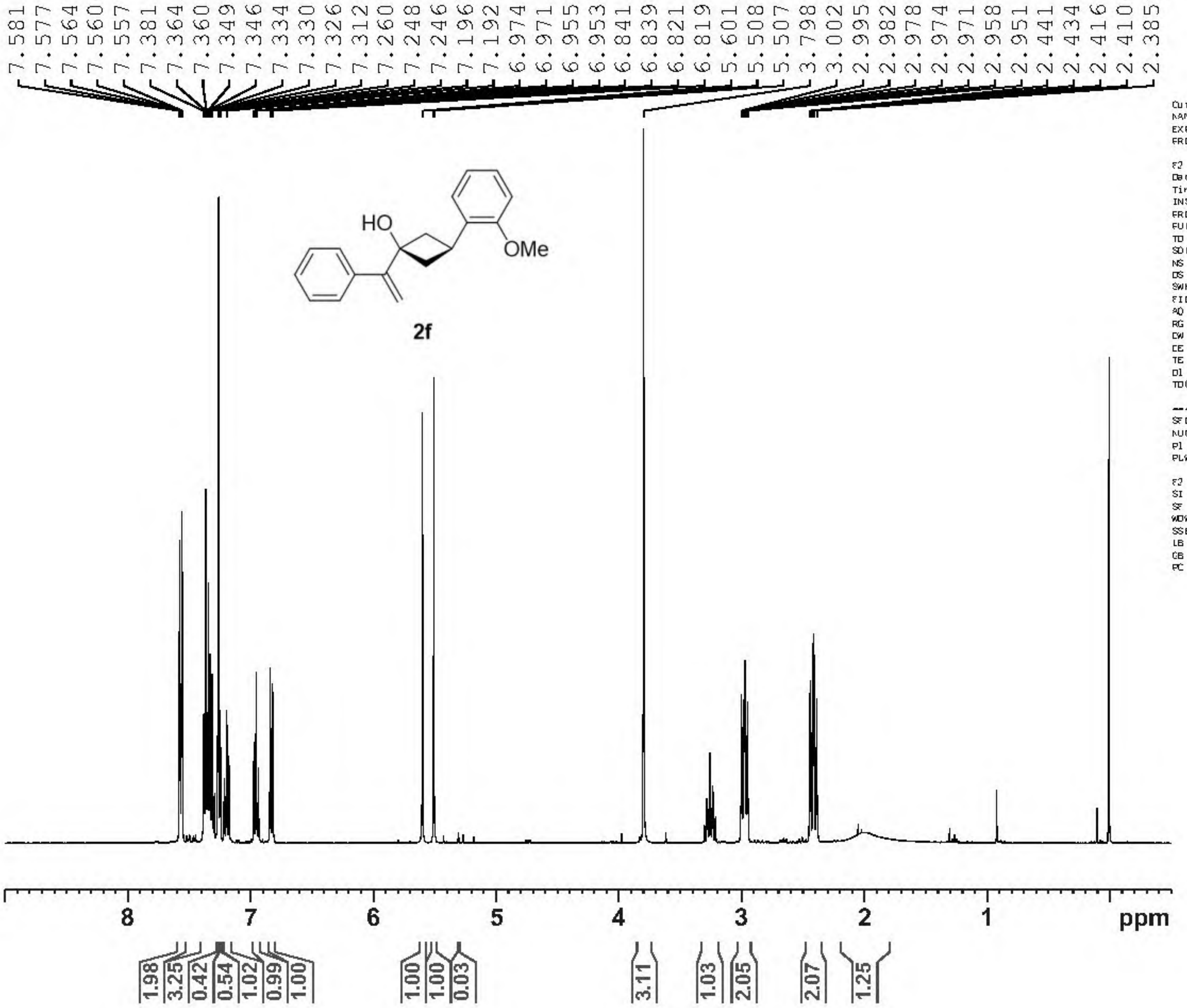


```
Current Data Parameters
NAME     zzj-4-mem-SM-alkene Ph-o-OMe-Ph-H
EXPNO                    1
PROCNO                   1

F2 - Acquisition Parameters
Date_             20260402
Time                 19.25
INSTRUM              spect
PROBHD   5 mm PABBO BB/
PULPROG               zg30
TD                   65536
SOLVENT              CDCl3
NS                      16
DS                       2
SWH             8012.820 Hz
FIDRES          0.122266 Hz
AQ             4.0894465 sec
RG                  112.31
DW                62.400 usec
DE                  6.50 usec
TE                 296.1 K
D1            1.00000000 sec
TD0                      1

-------- CHANNEL f1 --------
SFO1          400.1324710 MHz
NUC1                    1H
P1                   14.50 usec
PLW1           11.99499989 W

F2 - Processing parameters
SI                   65536
SF            400.1300097 MHz
WDW                     EM
SSB                      0
LB                    0.30 Hz
GB                       0
PC                    1.00
```

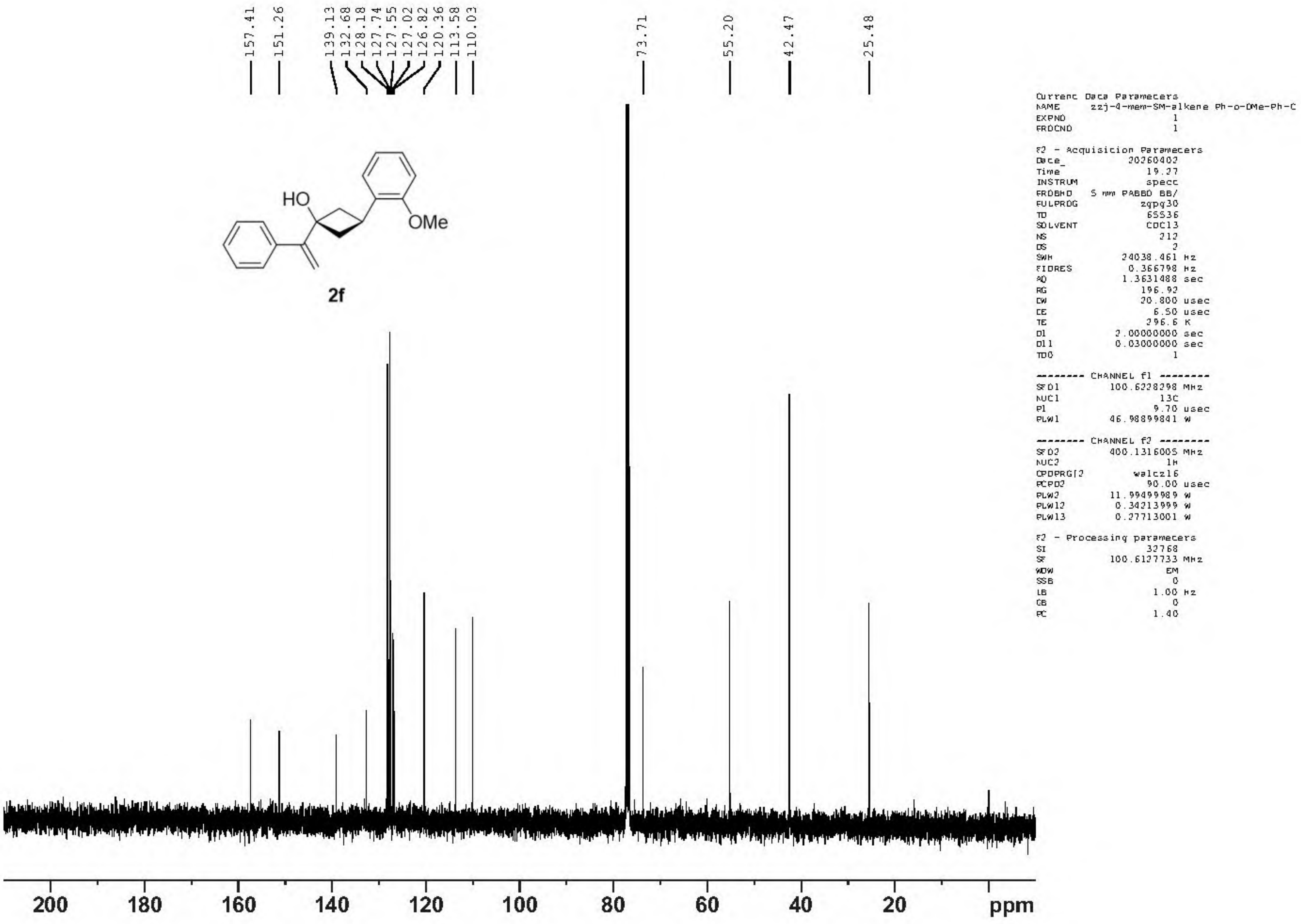
157.41
151.26
139.13
132.68
128.18
127.74
127.55
127.02
126.82
120.36
113.58
110.03
73.71
55.20
42.47
25.48
HO
OMe
2f
200
180
160
140
120
100
80
60
40
20
ppm
Current Data Parameters
NAME zzj-4-mem-SM-alkene Ph-o-OMe-Ph-C
EXPNO 1
PROCNO 1
F2 - Acquisition Parameters
Date_ 20260402
Time 19.27
INSTRUM spect
PROBHD 5 mm PABBO BB/
PULPROG zgpg30
TD 65536
SOLVENT CDCl3
NS 212
DS 2
SWH 24038.461 Hz
FIDRES 0.366798 Hz
AQ 1.3631488 sec
RG 196.92
DW 20.800 usec
DE 6.50 usec
TE 296.6 K
D1 2.00000000 sec
D11 0.03000000 sec
TD0 1
======== CHANNEL f1 ========
SFO1 100.6228298 MHz
NUC1 13C
P1 9.70 usec
PLW1 46.98899841 W
======== CHANNEL f2 ========
SFO2 400.1316005 MHz
NUC2 1H
CPDPRG[2 waltz16
PCPD2 90.00 usec
PLW2 11.99499989 W
PLW12 0.34213999 W
PLW13 0.27713001 W
F2 - Processing parameters
SI 32768
SF 100.6127733 MHz
WDW EM
SSB 0
LB 1.00 Hz
GB 0
PC 1.40

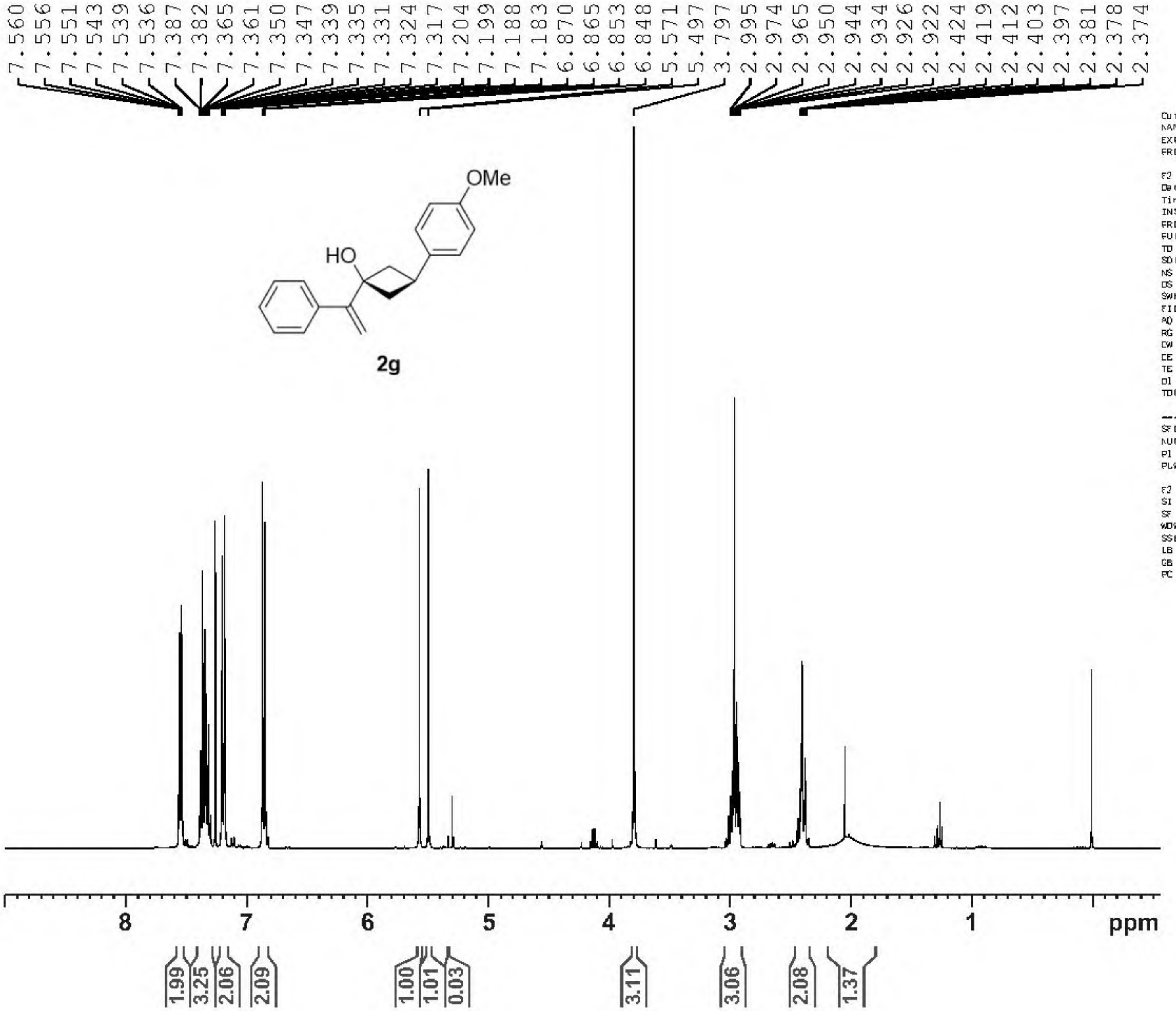


```
Current Data Parameters
NAME      zzj-4-mem-SM-alkene Ph-p-OMe-Ph-H
EXPNO                    1
PROCNO                   1

F2 - Acquisition Parameters
Date_             20260402
Time                 19.07
INSTRUM              spect
PROBHD   5 mm PABBO BB/
PULPROG               zg30
TD                   65536
SOLVENT              CDCl3
NS                      16
DS                       2
SWH             8012.820 Hz
FIDRES          0.122266 Hz
AQ             4.0894465 sec
RG                  112.31
DW                  62.400 usec
DE                    6.50 usec
TE                   295.9 K
D1            1.00000000 sec
TD0                      1

======== CHANNEL f1 ========
SFO1         400.1324710 MHz
NUC1                    1H
P1                   14.50 usec
PLW1          11.99499989 W

F2 - Processing parameters
SI                   65536
SF           400.1300097 MHz
WDW                     EM
SSB                      0
LB                    0.30 Hz
GB                       0
PC                    1.00
```

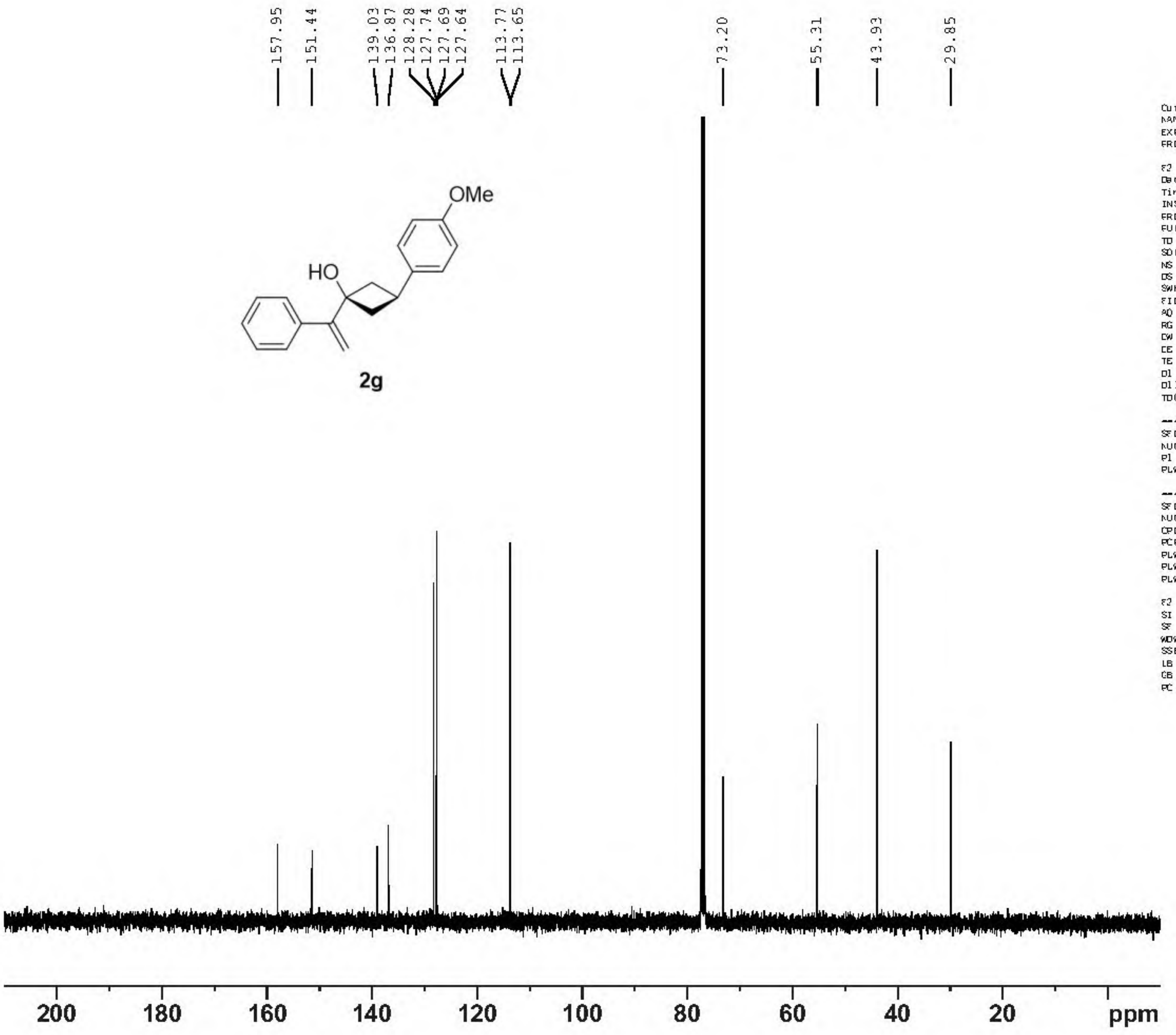


```
Current Data Parameters
NAME     zzj-4-mem-SM-alkene Ph-p-OMe-Ph-C
EXPNO                 1
PROCNO                1

F2 - Acquisition Parameters
Date_          20260402
Time              19.11
INSTRUM           spect
PROBHD   5 mm PABBO BB/
PULPROG          zgpg30
TD                65536
SOLVENT           CDCl3
NS                  202
DS                    2
SWH           24038.461 Hz
FIDRES         0.366798 Hz
AQ            1.3631488 sec
RG               196.92
DW               20.800 usec
DE                 6.50 usec
TE                296.6 K
D1           2.00000000 sec
D11          0.03000000 sec
TD0                   1

======== CHANNEL f1 ========
SFO1        100.6228298 MHz
NUC1                13C
P1                 9.70 usec
PLW1        46.98899841 W

======== CHANNEL f2 ========
SFO2        400.1316005 MHz
NUC2                 1H
CPDPRG[2        waltz16
PCPD2             90.00 usec
PLW2        11.99499989 W
PLW12        0.34213999 W
PLW13        0.27713001 W

F2 - Processing parameters
SI                32768
SF          100.6127690 MHz
WDW                  EM
SSB                   0
LB                 1.00 Hz
GB                    0
PC                 1.40
```

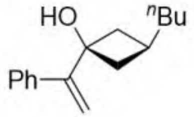

2h

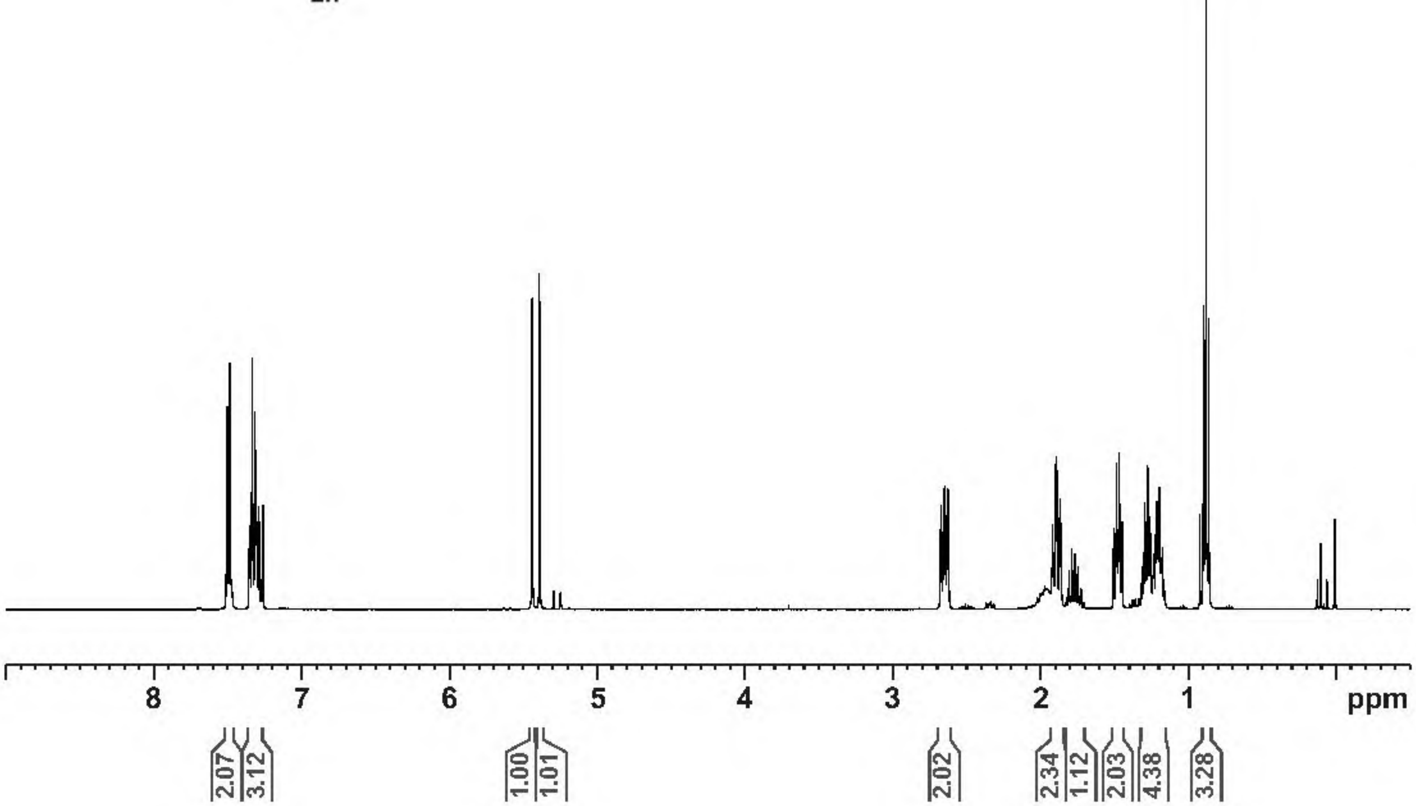


```
Current Data Parameters
NAME      zzj-4-mem-SM-Bu-H
EXPNO                     1
PROCNO                    1

F2 - Acquisition Parameters
Date_              20250217
Time                  11.06
INSTRUM               spect
PROBHD    5 mm PABBO BB/
PULPROG                zg30
TD                    65536
SOLVENT               CDCl3
NS                       16
DS                        2
SWH                8012.820 Hz
FIDRES             0.122266 Hz
AQ                4.0894465 sec
RG                    34.77
DW                   62.400 usec
DE                     6.50 usec
TE                    295.8 K
D1               1.00000000 sec
TD0                       1

======== CHANNEL f1 ========
SFO1         400.1324710 MHz
NUC1                     1H
P1                    14.50 usec
PLW1          11.99499989 W

F2 - Processing parameters
SI                    65536
SF           400.1300096 MHz
WDW                      EM
SSB                       0
LB                     0.30 Hz
GB                        0
PC                     1.00
```

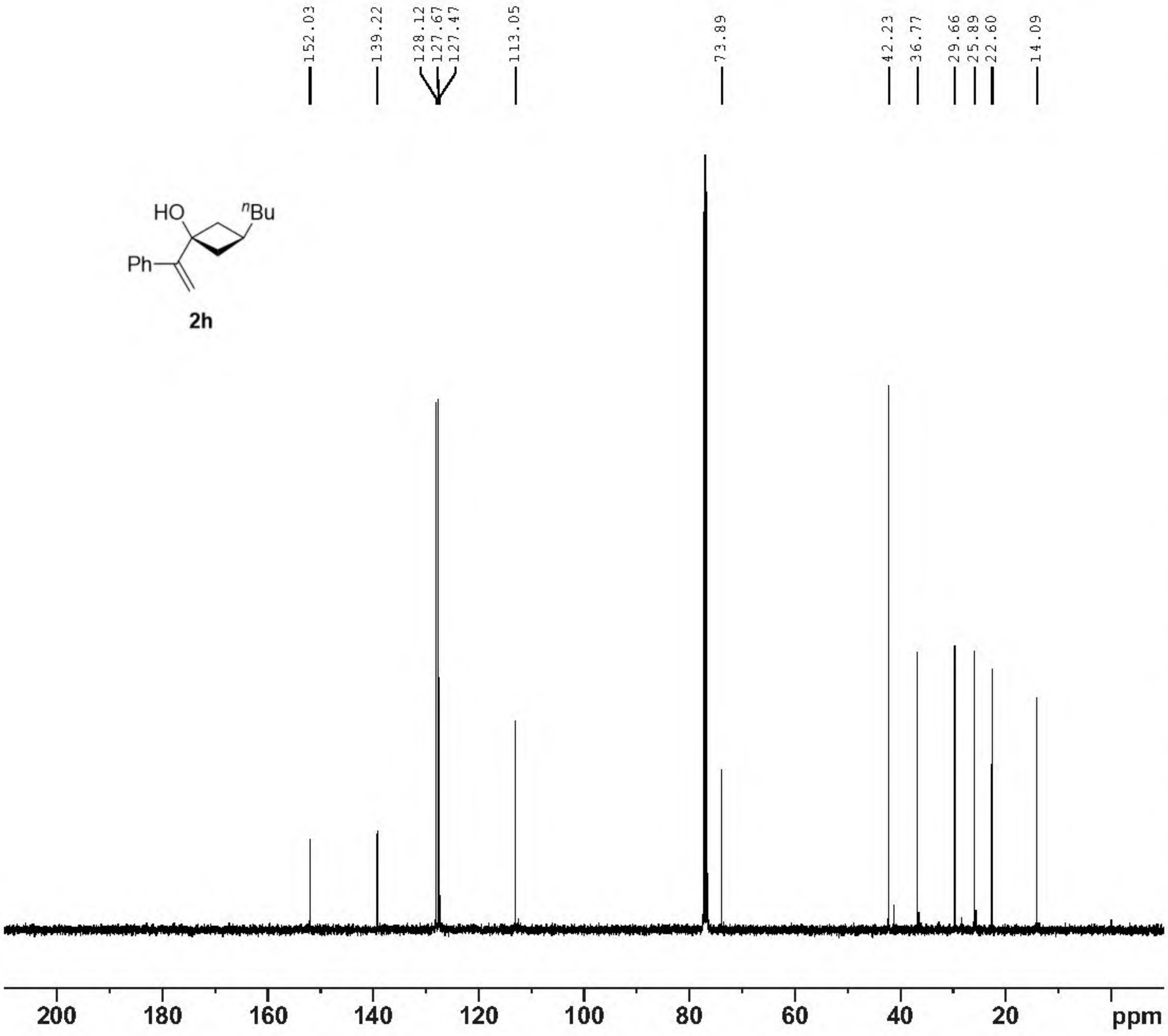

Current Data Parameters
NAME zzj-4-mem-SM-Bu-C
EXPNO 1
PROCNO 1

F2 - Acquisition Parameters
Date_ 20250217
Time 11.30
INSTRUM spect
PROBHD 5 mm PABBO BB/
PULPROG zgpg30
TD 65536
SOLVENT CDCl3
NS 400
DS 2
SWH 24038.461 Hz
FIDRES 0.366798 Hz
AQ 1.3631488 sec
RG 196.92
DW 20.800 usec
DE 6.50 usec
TE 297.0 K
D1 2.00000000 sec
D11 0.03000000 sec
TD0 1

======== CHANNEL f1 ========
SFO1 100.6228298 MHz
NUC1 13C
P1 9.70 usec
PLW1 46.98899841 W

======== CHANNEL f2 ========
SFO2 400.1316005 MHz
NUC2 1H
CPDPRG[2 waltz16
PCPD2 90.00 usec
PLW2 11.99499989 W
PLW12 0.34213999 W
PLW13 0.27713001 W

F2 - Processing parameters
SI 32768
SF 100.6127729 MHz
WDW EM
SSB 0
LB 1.00 Hz
GB 0
PC 1.40

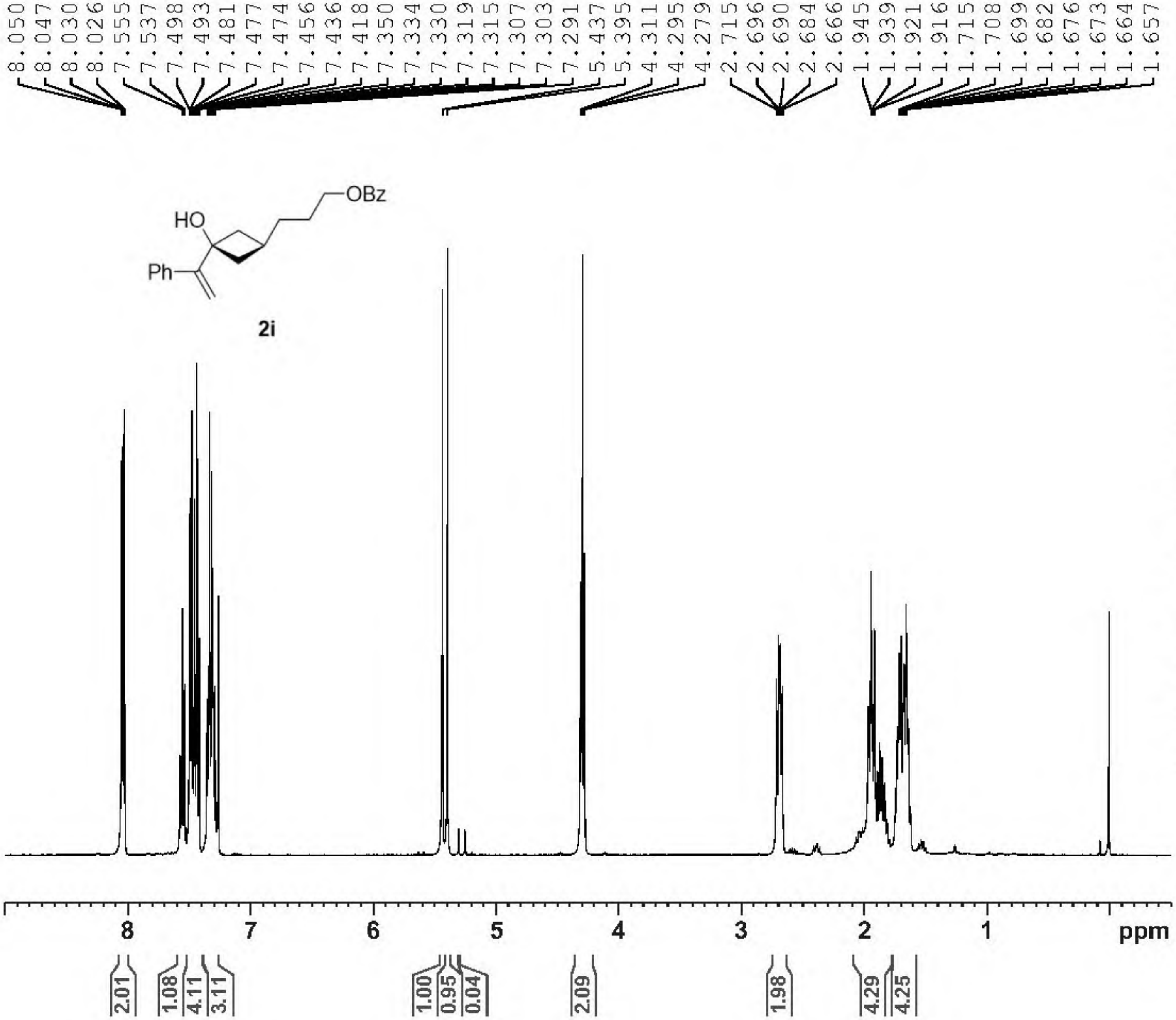


```
Current Data Parameters
NAME      zzj-4-mem-SM-OBz-H
EXPNO                    1
PROCNO                   1

F2 - Acquisition Parameters
Date_             20260509
Time                 11.18
INSTRUM              spect
PROBHD   5 mm PABBO BB/
PULPROG               zg30
TD                   65536
SOLVENT              CDCl3
NS                      16
DS                       2
SWH               8012.820 Hz
FIDRES            0.122266 Hz
AQ               4.0894465 sec
RG                   82.92
DW                  62.400 usec
DE                    6.50 usec
TE                   297.2 K
D1              1.00000000 sec
TD0                      1

======== CHANNEL f1 ========
SFO1          400.1324710 MHz
NUC1                    1H
P1                   14.50 usec
PLW1           11.99499989 W

F2 - Processing parameters
SI                   65536
SF            400.1300090 MHz
WDW                     EM
SSB                      0
LB                    0.30 Hz
GB                       0
PC                    1.00
```

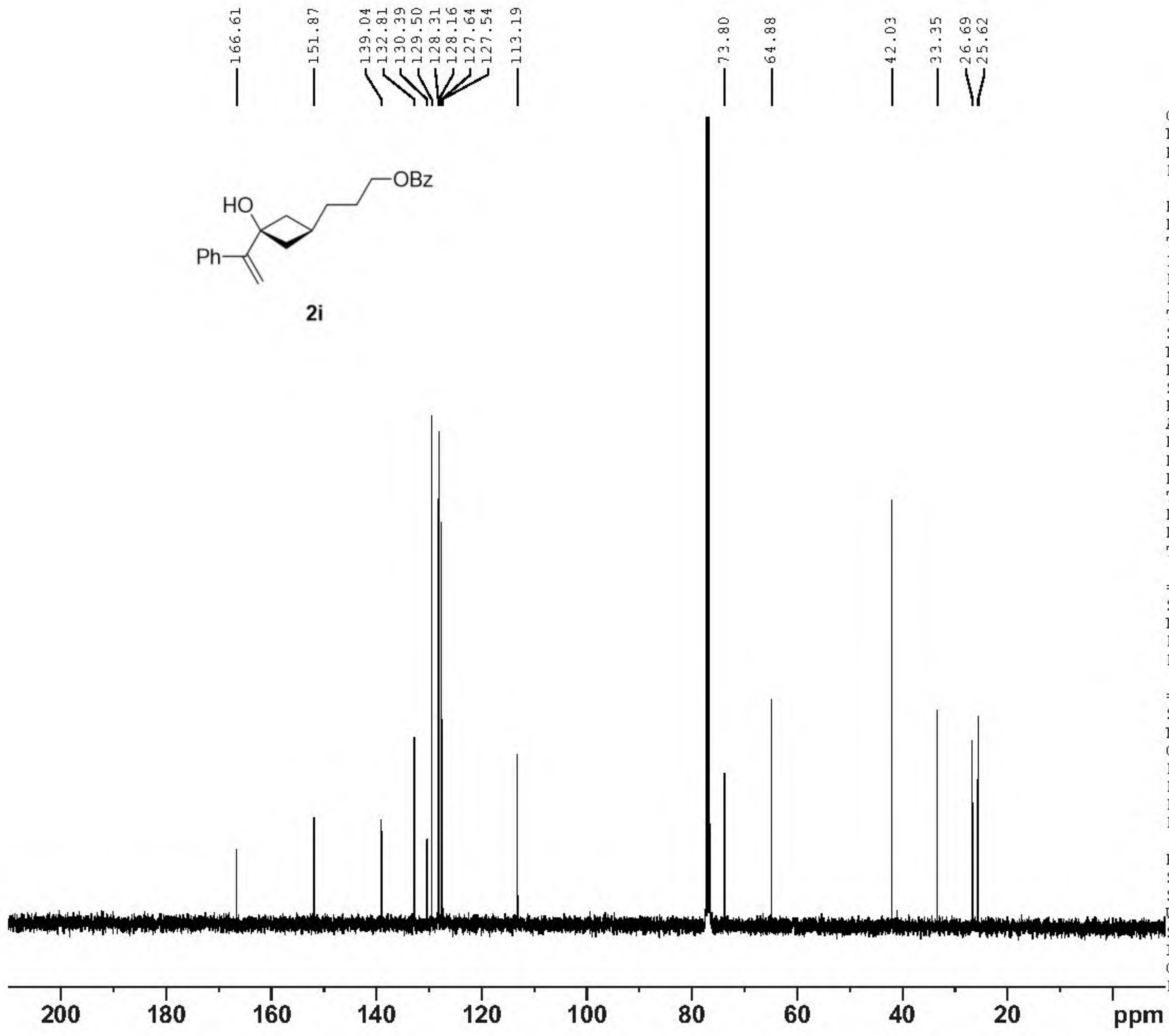


```
Current Data Parameters
NAME      zzj-4-mem-SM-OBz-C
EXPNO                    1
PROCNO                   1

F2 - Acquisition Parameters
Date_             20260509
Time                 11.30
INSTRUM              spect
PROBHD    5 mm PABBO BB/
PULPROG            zgpg30
TD                  65536
SOLVENT             CDCl3
NS                    200
DS                      2
SWH             24038.461 Hz
FIDRES           0.366798 Hz
AQ              1.3631488 sec
RG                 196.92
DW                 20.800 usec
DE                   6.50 usec
TE                  297.9 K
D1             2.00000000 sec
D11            0.03000000 sec
TD0                     1

======== CHANNEL f1 ========
SFO1          100.6228298 MHz
NUC1                  13C
P1                   9.70 usec
PLW1          46.98899841 W

======== CHANNEL f2 ========
SFO2          400.1316005 MHz
NUC2                   1H
CPDPRG[2          waltz16
PCPD2               90.00 usec
PLW2          11.99499989 W
PLW12          0.34213999 W
PLW13          0.27713001 W

F2 - Processing parameters
SI                  32768
SF            100.6127734 MHz
WDW                    EM
SSB                     0
LB                   1.00 Hz
GB                      0
PC                   1.40
```

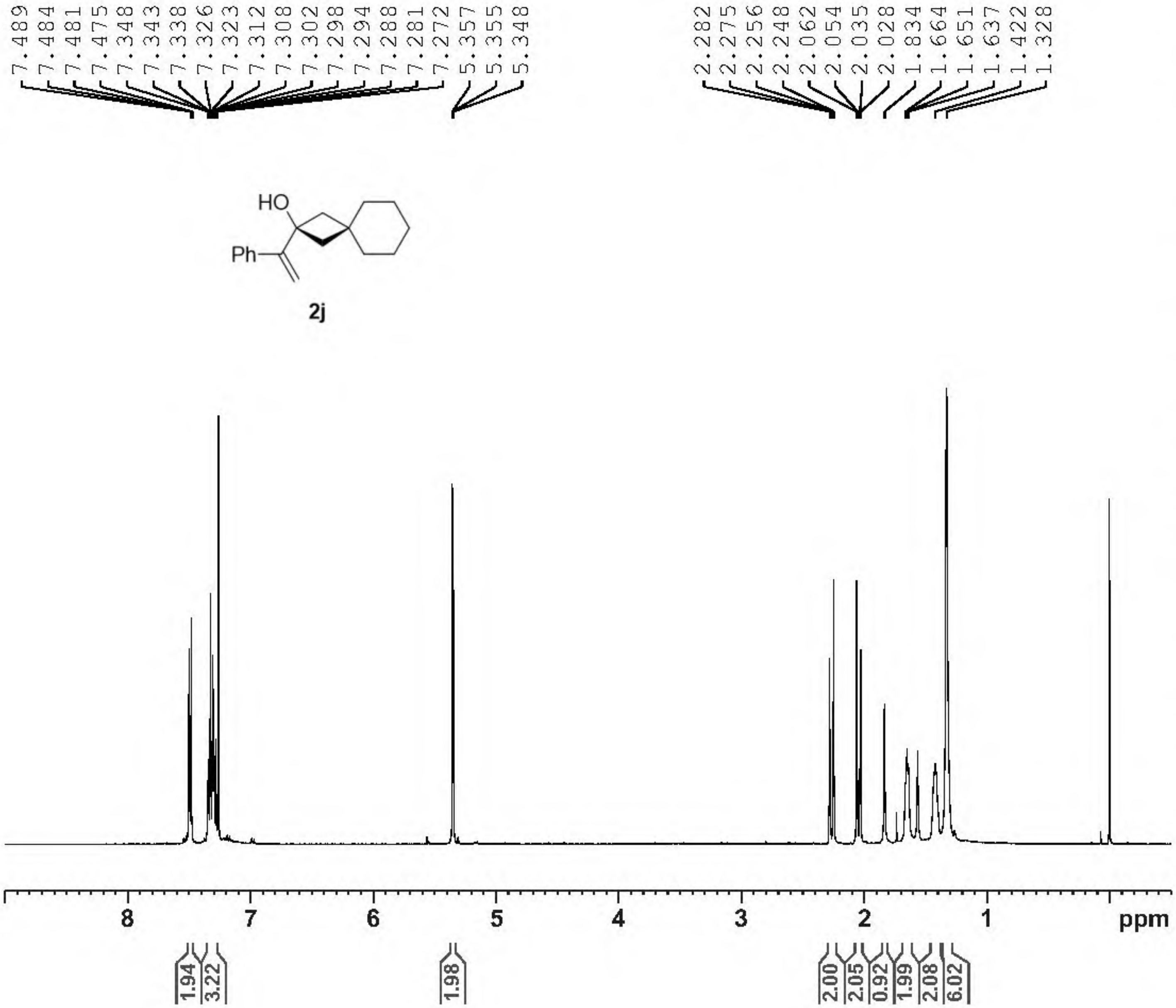

Current Data Parameters
NAME zzj-4-mem-SM-hexane-H
EXPNO 1
PROCNO 1

F2 - Acquisition Parameters
Date_ 20250115
Time 23.07
INSTRUM spect
PROBHD 5 mm PABBO BB/
PULPROG zg30
TD 65536
SOLVENT CDCl3
NS 16
DS 2
SWH 8012.820 Hz
FIDRES 0.122266 Hz
AQ 4.0894465 sec
RG 126.97
DW 62.400 usec
DE 6.50 usec
TE 295.7 K
D1 1.00000000 sec
TD0 1

======== CHANNEL f1 ========
SFO1 400.1324710 MHz
NUC1 1H
P1 14.50 usec
PLW1 11.99499989 W

F2 - Processing parameters
SI 65536
SF 400.1300098 MHz
WDW EM
SSB 0
LB 0.30 Hz
GB 0
PC 1.00

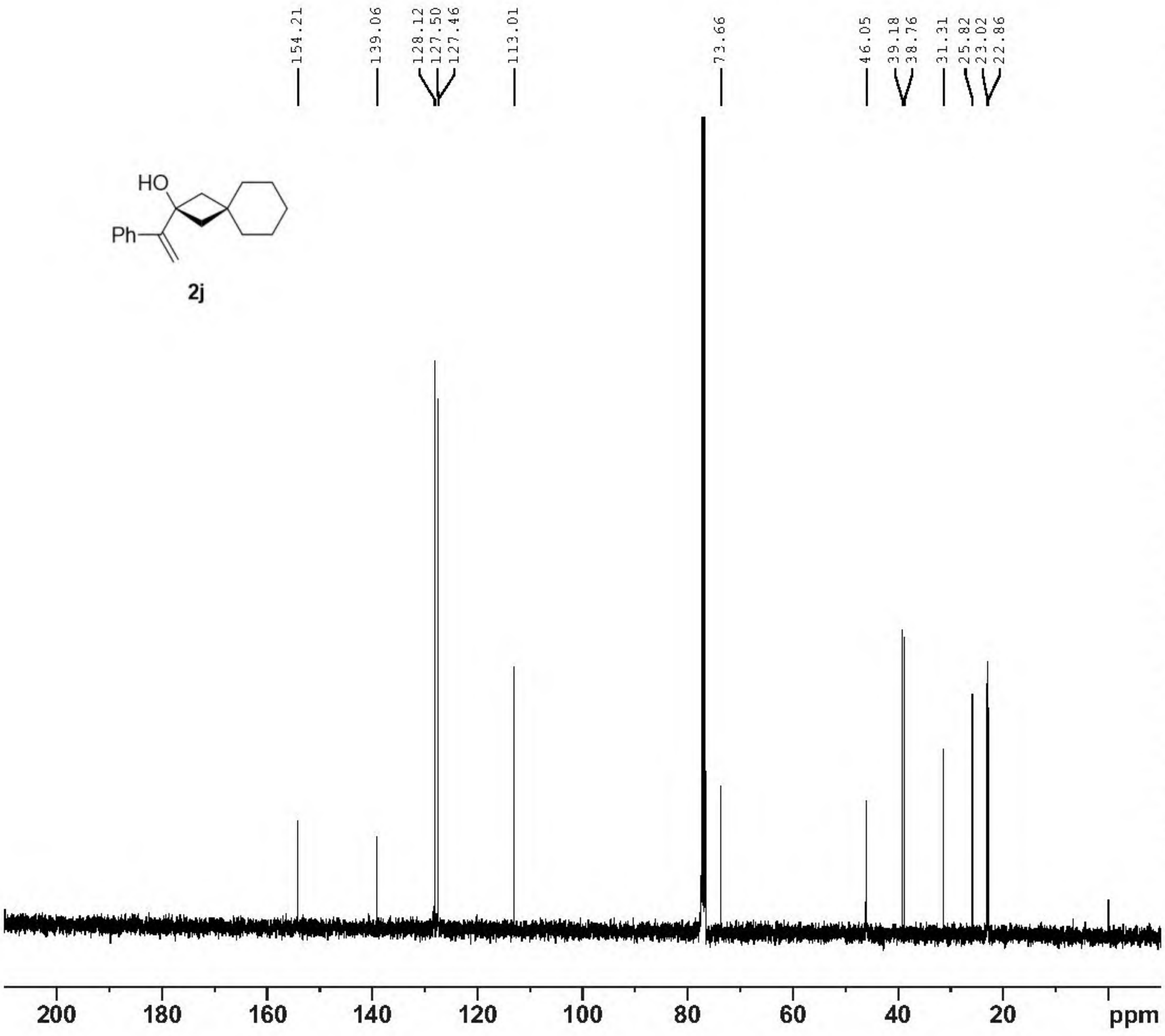


```
Current Data Parameters
NAME      zzj-4-mem-SM-hexane-C
EXPNO                     1
PROCNO                    1

F2 - Acquisition Parameters
Date_              20250116
Time                   0.05
INSTRUM               spect
PROBHD   5 mm PABBO BB/
PULPROG             zgpg30
TD                    65536
SOLVENT               CDCl3
NS                     1000
DS                        2
SWH           24038.461 Hz
FIDRES         0.366798 Hz
AQ            1.3631488 sec
RG                   196.92
DW               20.800 usec
DE                 6.50 usec
TE                296.9 K
D1           2.00000000 sec
D11          0.03000000 sec
TD0                       1

======== CHANNEL f1 ========
SFO1        100.6228298 MHz
NUC1                    13C
P1                 9.70 usec
PLW1        46.98899841 W

======== CHANNEL f2 ========
SFO2        400.1316005 MHz
NUC2                     1H
CPDPRG[2            waltz16
PCPD2             90.00 usec
PLW2        11.99499989 W
PLW12        0.34213999 W
PLW13        0.27713001 W

F2 - Processing parameters
SI                    32768
SF          100.6127716 MHz
WDW                      EM
SSB                       0
LB                   1.00 Hz
GB                        0
PC                     1.40
```

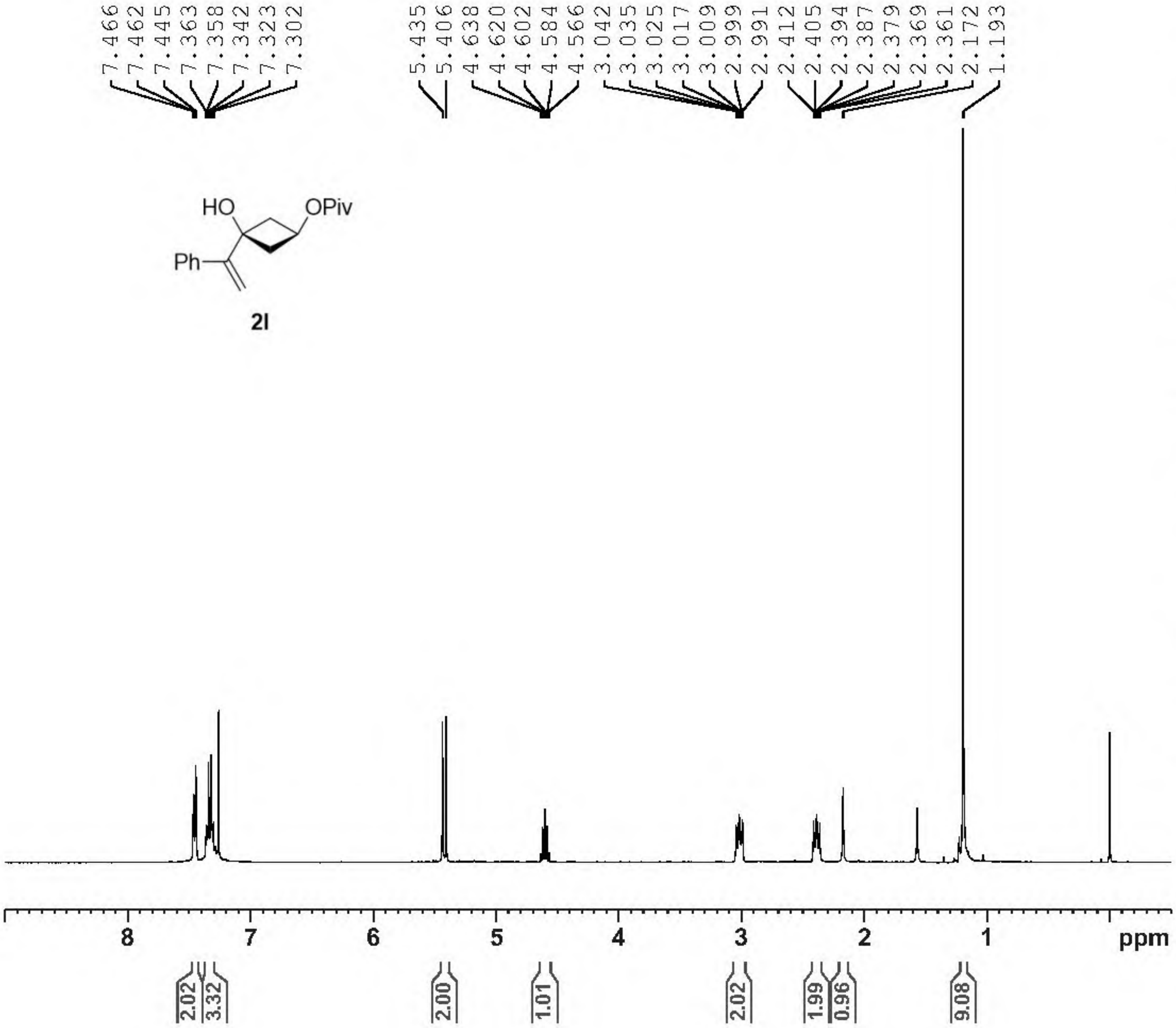


```
Current Data Parameters
NAME      zzj-4-mem-SM-OPiv-H
EXPNO                   1
PROCNO                  1

F2 - Acquisition Parameters
Date_            20241227
Time                23.01
INSTRUM             spect
PROBHD   5 mm PABBO BB/
PULPROG              zg30
TD                  65536
SOLVENT             CDCl3
NS                     16
DS                      2
SWH              8012.820 Hz
FIDRES           0.122266 Hz
AQ              4.0894465 sec
RG                 126.97
DW                 62.400 usec
DE                   6.50 usec
TE                  295.8 K
D1             1.00000000 sec
TD0                     1

======== CHANNEL f1 ========
SFO1         400.1324710 MHz
NUC1                   1H
P1                  14.50 usec
PLW1          11.99499989 W

F2 - Processing parameters
SI                  65536
SF           400.1300097 MHz
WDW                    EM
SSB                     0
LB                   0.30 Hz
GB                      0
PC                   1.00
```

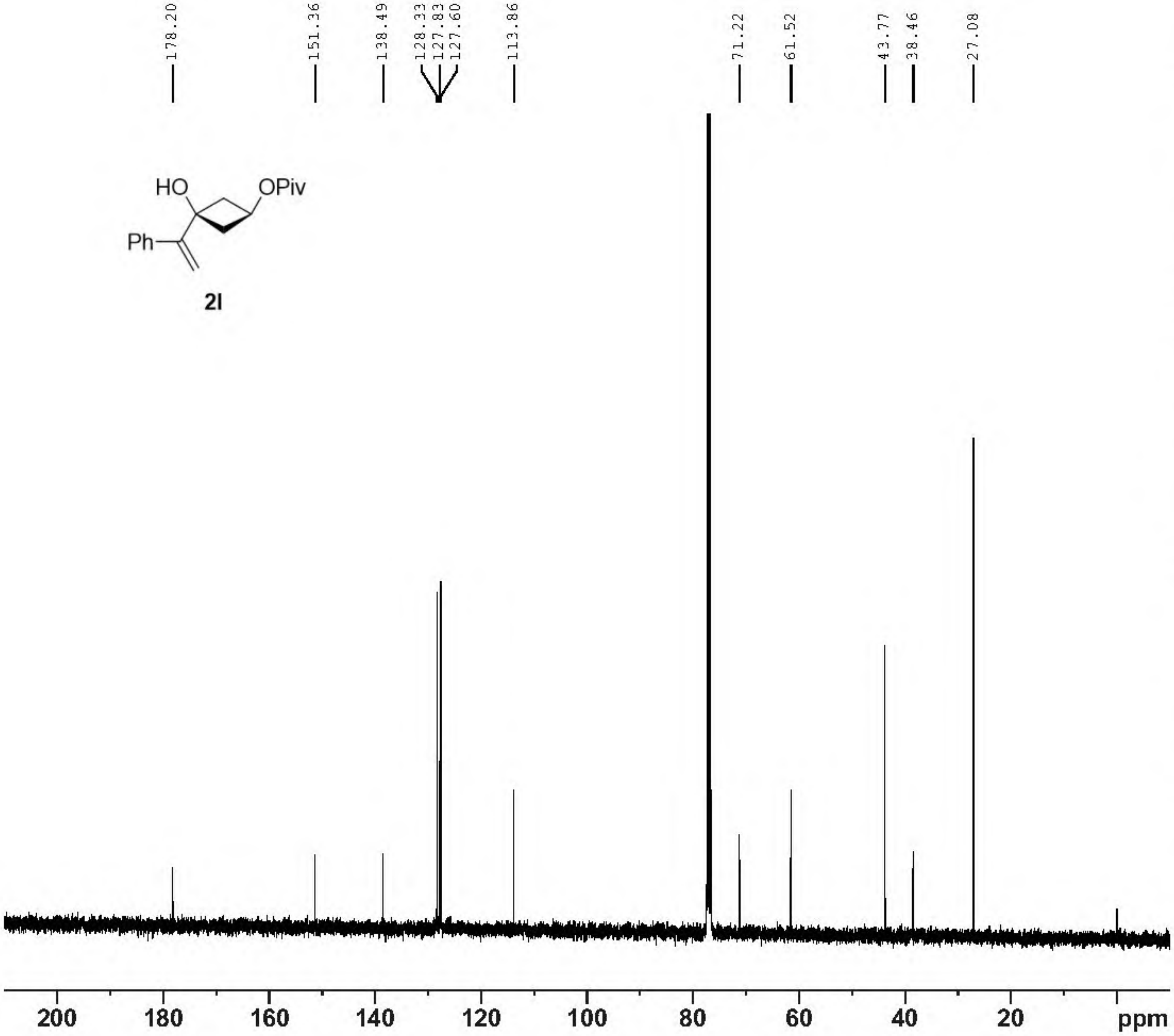


```
Current Data Parameters
NAME      zzj-4-mem-SM-OPiv-C
EXPNO                       1
PROCNO                      1

F2 - Acquisition Parameters
Date_             20241228
Time                     0
INSTRUM              spect
PROBHD    5 mm PABBO BB/
PULPROG            zgpg30
TD                  65536
SOLVENT             CDCl3
NS                   1000
DS                      2
SWH            24038.461 Hz
FIDRES          0.366798 Hz
AQ             1.3631488 sec
RG                 196.92
DW                 20.800 usec
DE                   6.50 usec
TE                  297.1 K
D1            2.00000000 sec
D11           0.03000000 sec
TD0                     1

======== CHANNEL f1 ========
SFO1         100.6228298 MHz
NUC1                  13C
P1                   9.70 usec
PLW1         46.98899841 W

======== CHANNEL f2 ========
SFO2         400.1316005 MHz
NUC2                   1H
CPDPRG[2          waltz16
PCPD2               90.00 usec
PLW2         11.99499989 W
PLW12         0.34213999 W
PLW13         0.27713001 W

F2 - Processing parameters
SI                  32768
SF           100.6127709 MHz
WDW                    EM
SSB                     0
LB                   1.00 Hz
GB                      0
PC                   1.40
```

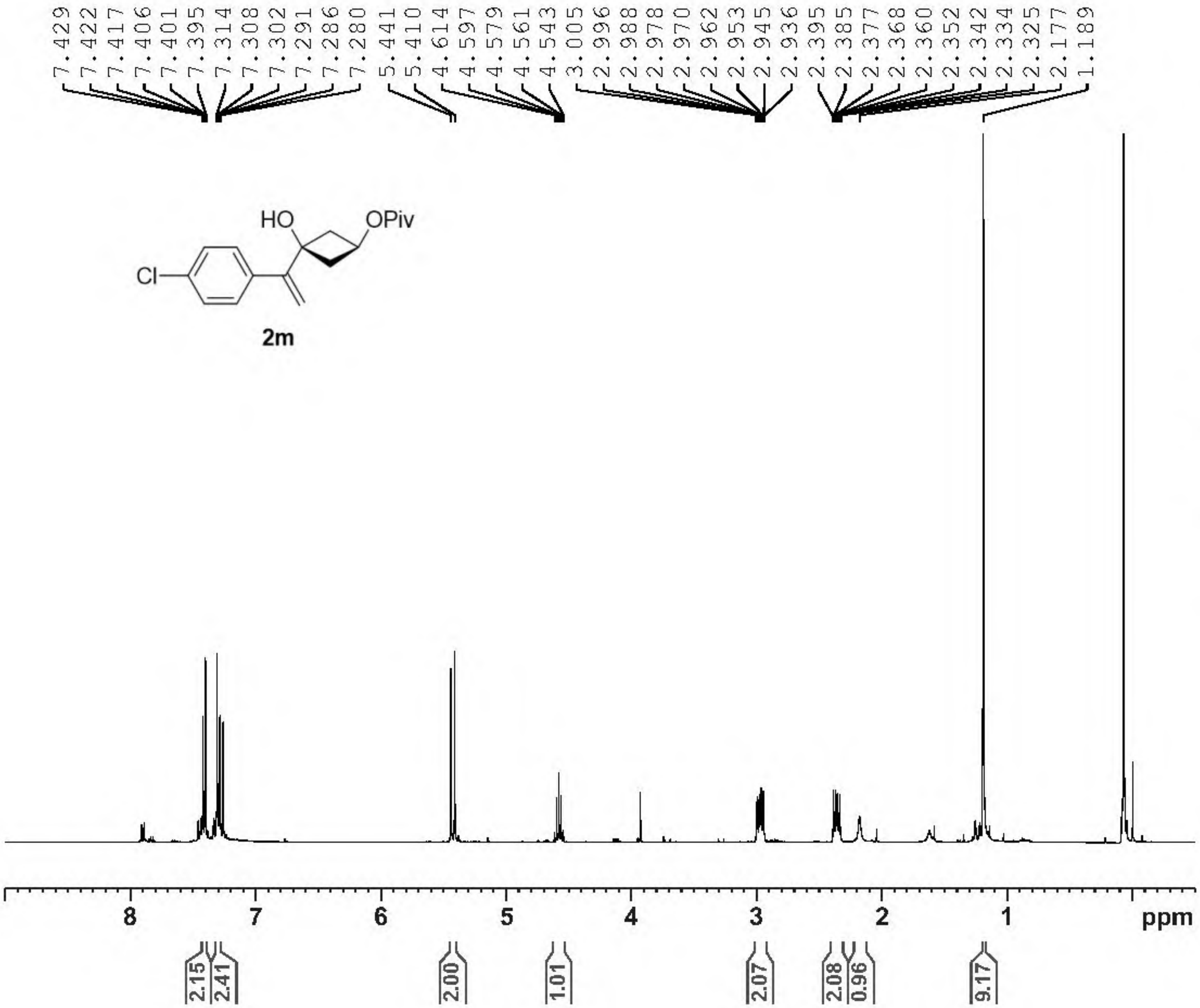


```
Current Data Parameters
NAME     zzj-4-mem-SM-OPiv-pClPh-H
EXPNO                    1
PROCNO                   1

F2 - Acquisition Parameters
Date_             20250307
Time                 10.54
INSTRUM              spect
PROBHD   5 mm PABBO BB/
PULPROG               zg30
TD                   65536
SOLVENT              CDCl3
NS                      16
DS                       2
SWH               8012.820 Hz
FIDRES            0.122266 Hz
AQ               4.0894465 sec
RG                   70.97
DW                  62.400 usec
DE                    6.50 usec
TE                   294.2 K
D1              1.00000000 sec
TD0                      1

-------- CHANNEL f1 --------
SFO1           400.1324710 MHz
NUC1                    1H
P1                   14.50 usec
PLW1           11.99499989 W

F2 - Processing parameters
SI                   65536
SF             400.1300097 MHz
WDW                     EM
SSB                      0
LB                    0.30 Hz
GB                       0
PC                    1.00
```

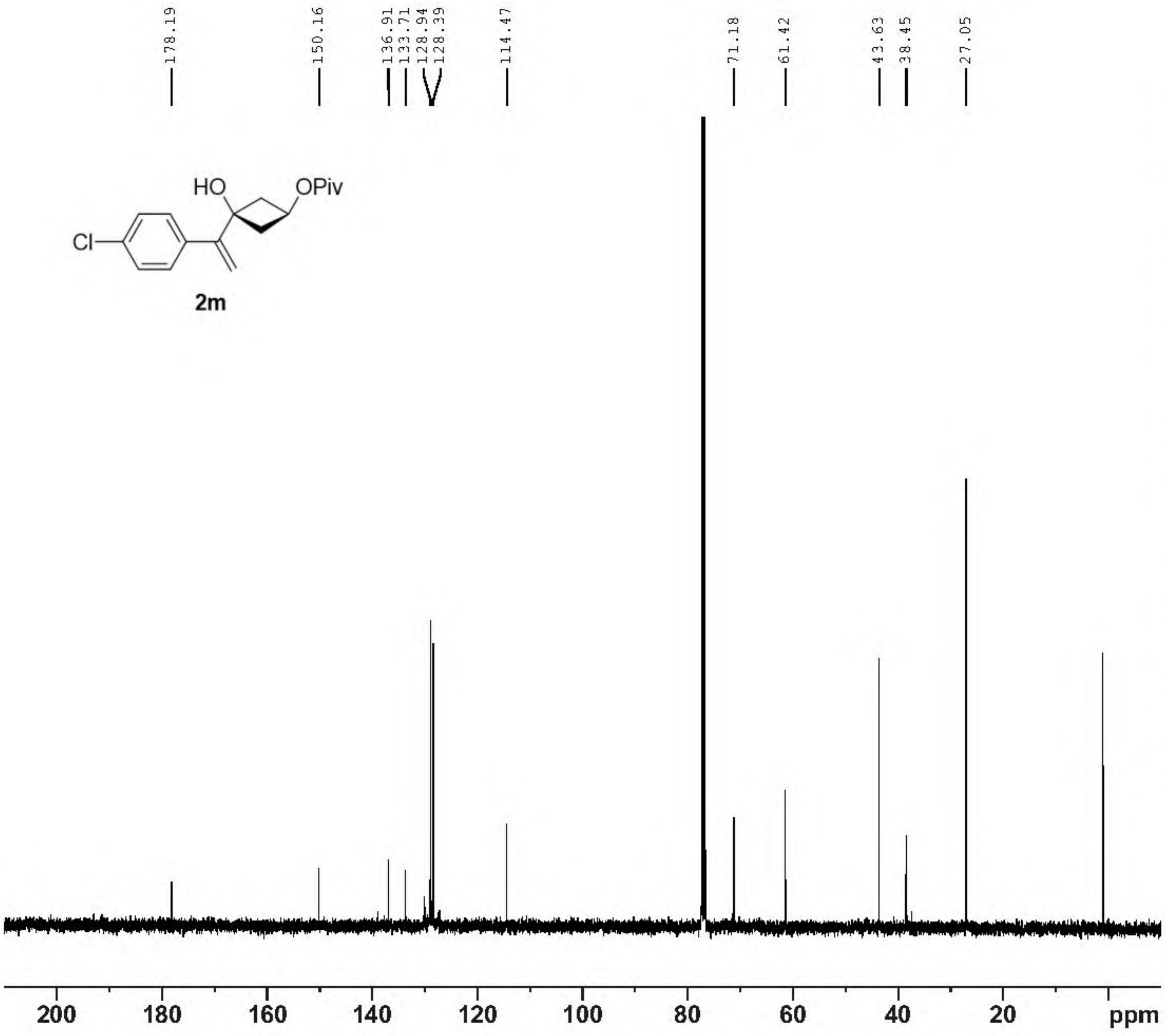


```
Current Data Parameters
NAME      zzj-4-mem-SM-OPiv-pClPh-C
EXPNO                    1
PROCNO                   1

F2 - Acquisition Parameters
Date_             20250307
Time                 10.56
INSTRUM              spect
PROBHD   5 mm PABBO BB/
PULPROG            zgpg30
TD                   65536
SOLVENT              CDCl3
NS                     227
DS                       2
SWH           24038.461 Hz
FIDRES         0.366798 Hz
AQ            1.3631488 sec
RG                 196.92
DW                 20.800 usec
DE                   6.50 usec
TE                  294.5 K
D1           2.00000000 sec
D11          0.03000000 sec
TD0                      1

-------- CHANNEL f1 --------
SFO1        100.6228298 MHz
NUC1                   13C
P1                    9.70 usec
PLW1        46.98899841 W

-------- CHANNEL f2 --------
SFO2        400.1316005 MHz
NUC2                    1H
CPDPRG[2           waltz16
PCPD2                90.00 usec
PLW2        11.99499989 W
PLW12        0.34213999 W
PLW13        0.27713001 W

F2 - Processing parameters
SI                   32768
SF          100.6127727 MHz
WDW                     EM
SSB                      0
LB                    1.00 Hz
GB                       0
PC                    1.40
```

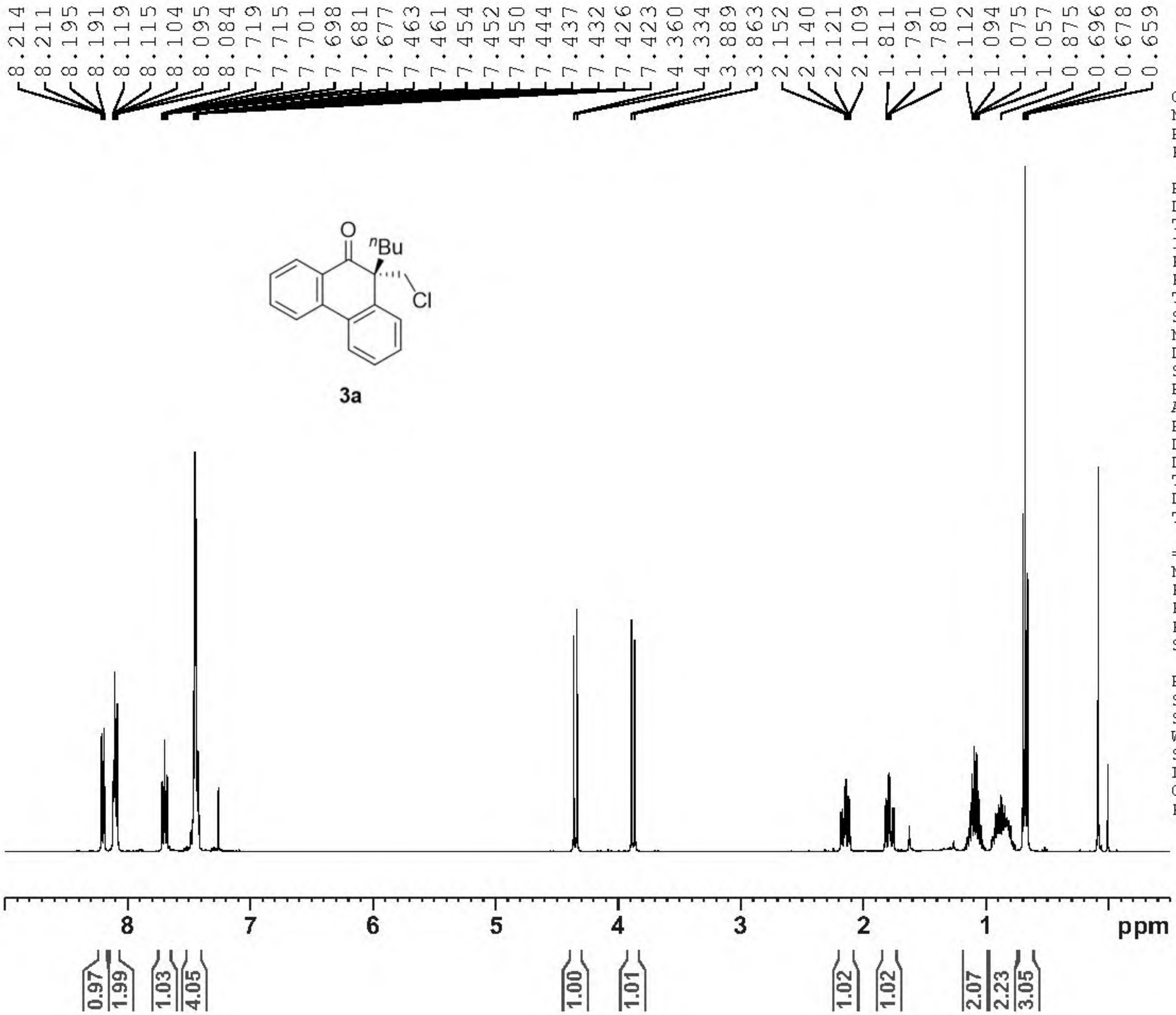


```
Current Data Parameters
NAME              pdt-Bu-H
EXPNO                    1
PROCNO                   1

F2 - Acquisition Parameters
Date_             20240312
Time                 18.10
INSTRUM              spect
PROBHD   5 mm DUL 13C-1
PULPROG               zg30
TD                   65536
SOLVENT              CDCl3
NS                      16
DS                       2
SWH              8223.685 Hz
FIDRES           0.125483 Hz
AQ              3.9845889 sec
RG                     203
DW                  60.800 usec
DE                    6.00 usec
TE                   292.3 K
D1             1.00000000 sec
TD0                      1

======== CHANNEL f1 ========
NUC1                    1H
P1                   15.80 usec
PL1                  -1.00 dB
PL1W           12.17476940 W
SFO1           400.1324710 MHz

F2 - Processing parameters
SI                   32768
SF             400.1300098 MHz
WDW                     EM
SSB                      0
LB                    0.30 Hz
GB                       0
PC                    1.00
```

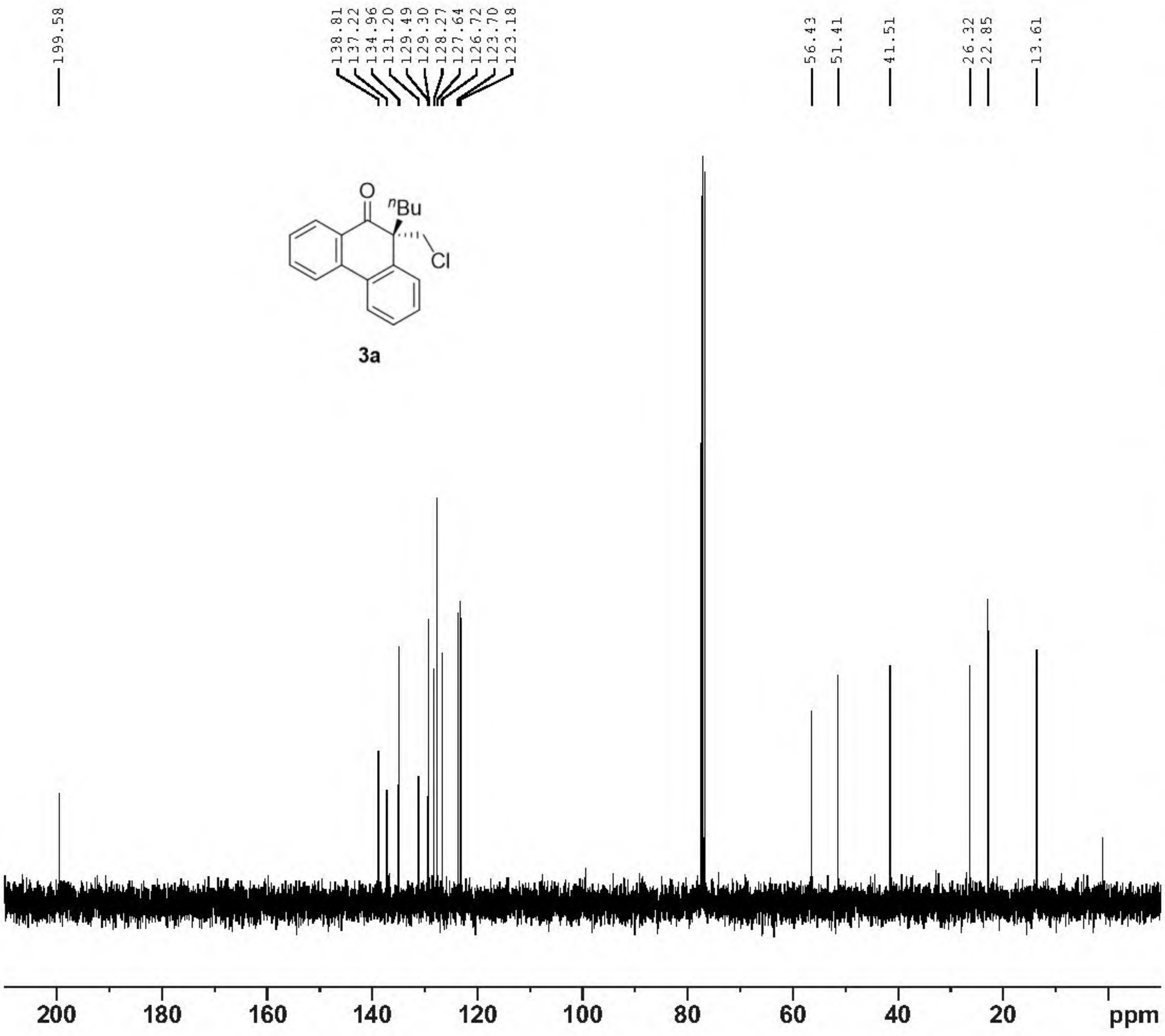


```
Current Data Parameters
NAME             pdt-Bu-C
EXPNO                   1
PROCNO                  1

F2 - Acquisition Parameters
Date_            20240312
Time                18.16
INSTRUM             spect
PROBHD   5 mm DUL 13C-1
PULPROG           zgpg30
TD                 65536
SOLVENT            CDCl3
NS                   144
DS                     1
SWH           24038.461 Hz
FIDRES         0.366798 Hz
AQ            1.3631488 sec
RG                  71.8
DW                20.800 usec
DE                  6.00 usec
TE                 292.7 K
D1            2.00000000 sec
D11           0.03000000 sec
TD0                    1

======== CHANNEL f1 ========
NUC1                 13C
P1                 40.00 usec
PL1                -3.00 dB
PL1W         60.64365387 W
SFO1         100.6228298 MHz

======== CHANNEL f2 ========
CPDPRG[2         waltz16
NUC2                  1H
PCPD2              80.00 usec
PL2                -1.00 dB
PL12               14.39 dB
PL13               18.00 dB
PL2W         12.17476940 W
PL12W         0.35193357 W
PL13W         0.15327126 W
SFO2         400.1316005 MHz

F2 - Processing parameters
SI                 32768
SF           100.6127699 MHz
WDW                   EM
SSB                    0
LB                  1.00 Hz
GB                     0
PC                  1.40
```

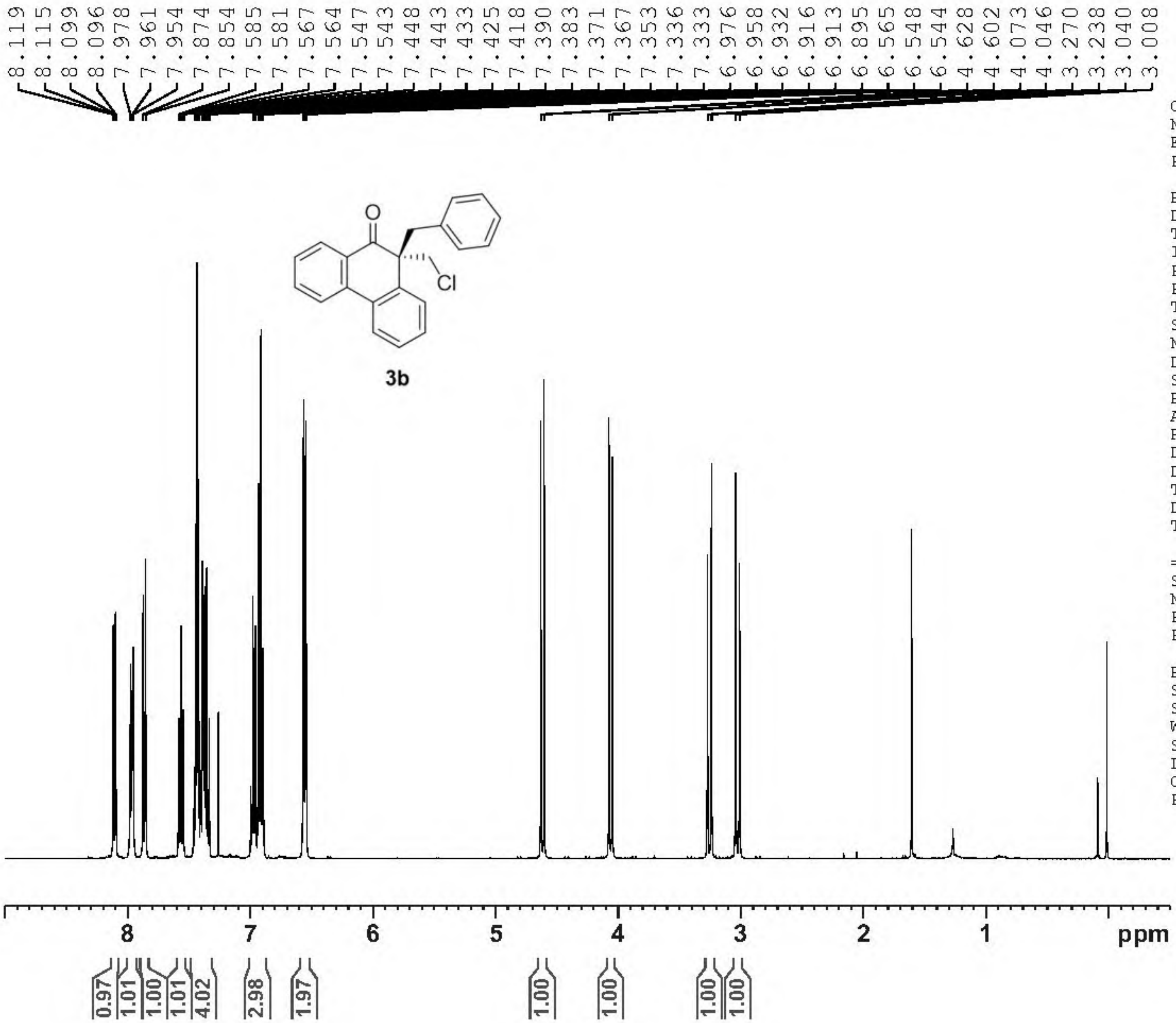

Current Data Parameters
NAME pdt-Bn-H
EXPNO 1
PROCNO 1

F2 - Acquisition Parameters
Date_ 20240122
Time 19.09
INSTRUM spect
PROBHD 5 mm PABBO BB/
PULPROG zg30
TD 65536
SOLVENT CDCl3
NS 5
DS 2
SWH 8012.820 Hz
FIDRES 0.122266 Hz
AQ 4.0894465 sec
RG 70.97
DW 62.400 usec
DE 6.50 usec
TE 295.3 K
D1 1.00000000 sec
TD0 1

======== CHANNEL f1 ========
SFO1 400.1324710 MHz
NUC1 1H
P1 14.50 usec
PLW1 11.99499989 W

F2 - Processing parameters
SI 65536
SF 400.1300098 MHz
WDW EM
SSB 0
LB 0.30 Hz
GB 0
PC 1.00

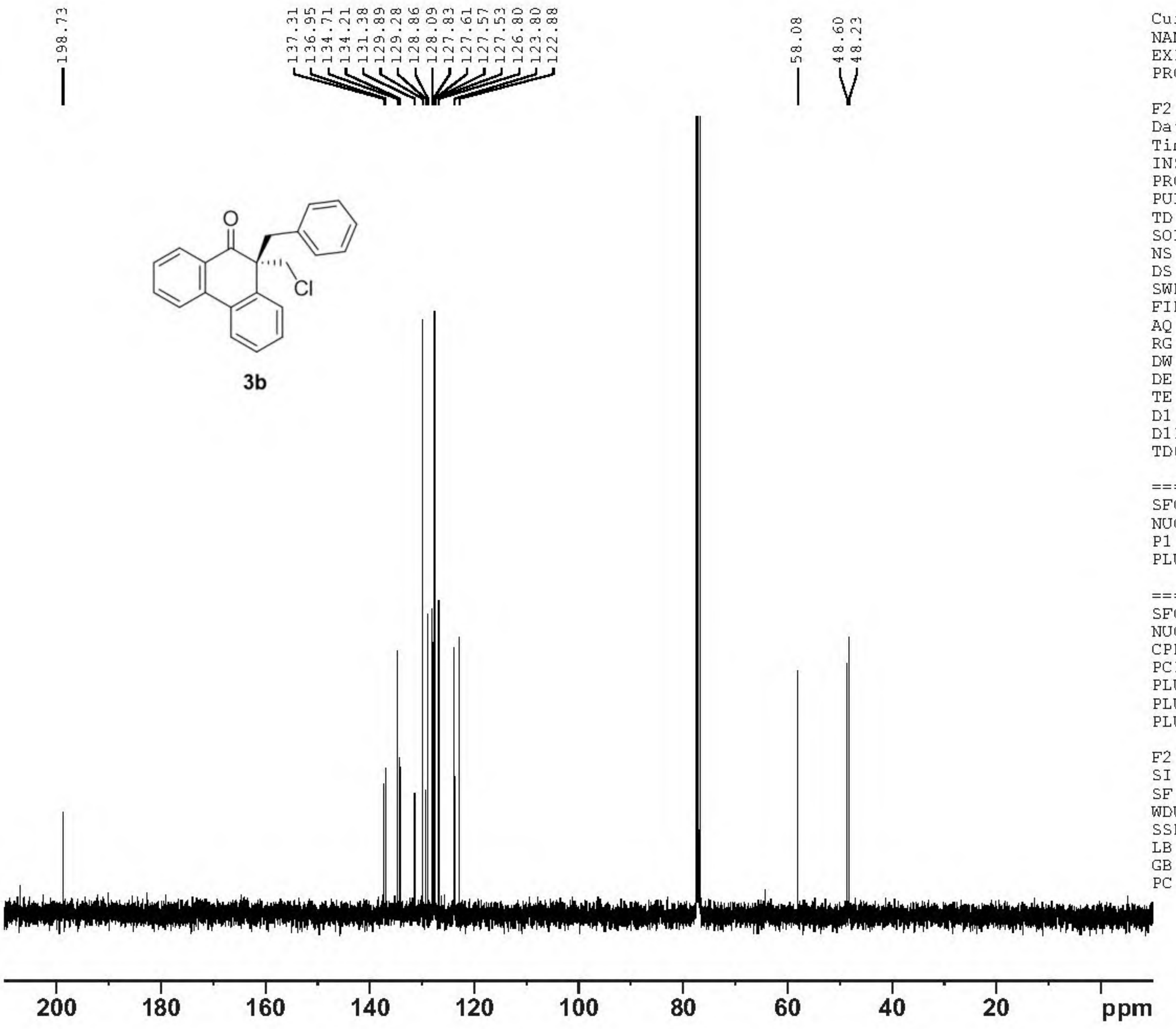


```
Current Data Parameters
NAME              pdt-Bn-C
EXPNO                    1
PROCNO                   1

F2 - Acquisition Parameters
Date_             20240122
Time                 19.11
INSTRUM              spect
PROBHD   5 mm PABBO BB/
PULPROG            zgpg30
TD                   65536
SOLVENT              CDCl3
NS                      79
DS                       2
SWH            24038.461 Hz
FIDRES          0.366798 Hz
AQ             1.3631488 sec
RG                  196.92
DW                  20.800 usec
DE                    6.50 usec
TE                   295.8 K
D1              2.00000000 sec
D11             0.03000000 sec
TD0                      1

======== CHANNEL f1 ========
SFO1         100.6228298 MHz
NUC1                   13C
P1                    9.70 usec
PLW1           46.98899841 W

======== CHANNEL f2 ========
SFO2         400.1316005 MHz
NUC2                    1H
CPDPRG[2           waltz16
PCPD2                90.00 usec
PLW2           11.99499989 W
PLW12           0.34213999 W
PLW13           0.27713001 W

F2 - Processing parameters
SI                   32768
SF           100.6127695 MHz
WDW                     EM
SSB                      0
LB                    1.00 Hz
GB                       0
PC                    1.40
```

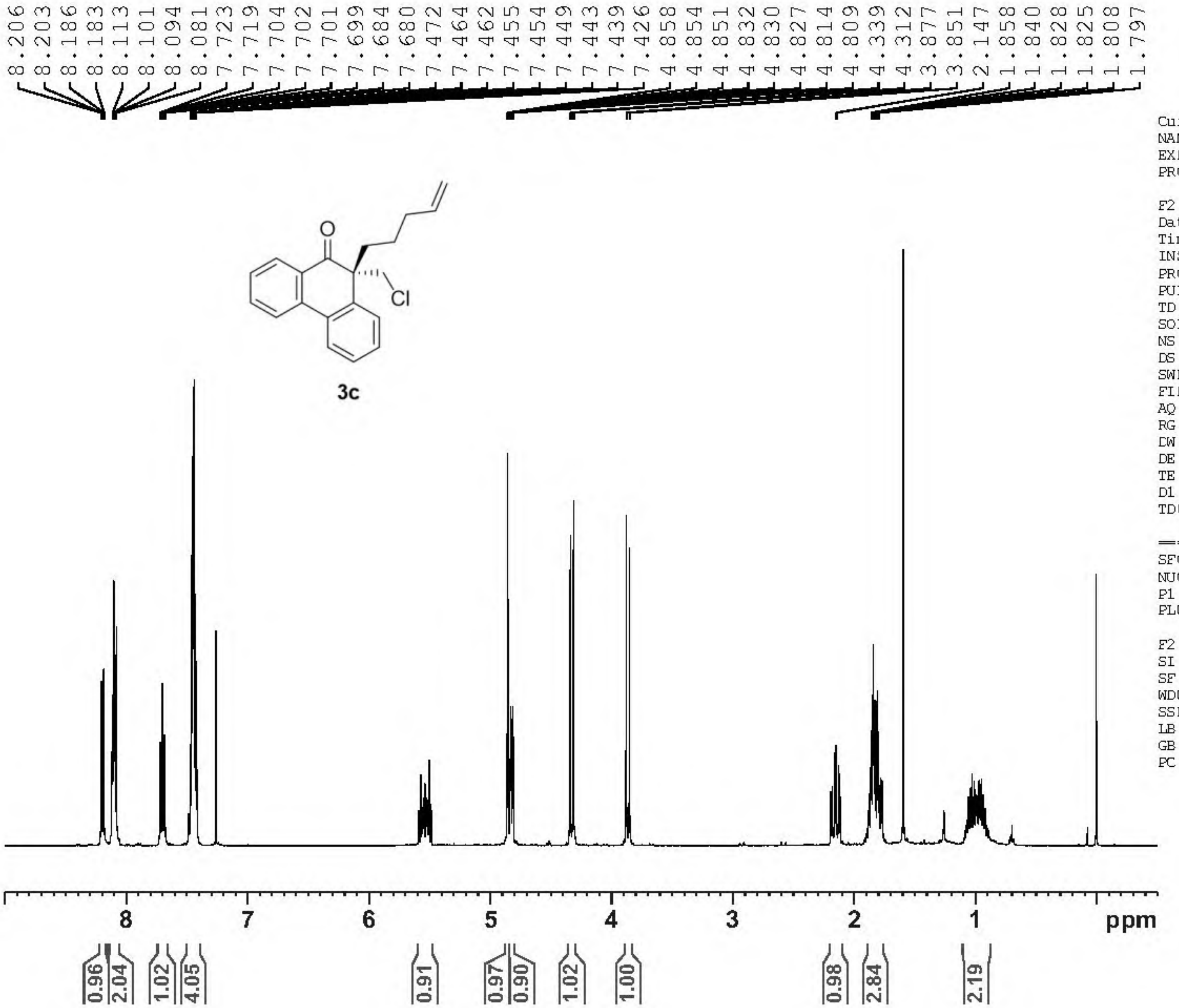

Current Data Parameters
NAME pdt-terminal alkene-H
EXPNO 1
PROCNO 1

F2 - Acquisition Parameters
Date_ 20240422
Time_ 22.48
INSTRUM spect
PROBHD 5 mm PABBO BB/
PULPROG zg30
TD 65536
SOLVENT CDCl3
NS 16
DS 2
SWH 8012.820 Hz
FIDRES 0.122266 Hz
AQ 4.0894465 sec
RG 112.31
DW 62.400 usec
DE 6.50 usec
TE 296.7 K
D1 1.00000000 sec
TD0 1

======= CHANNEL f1 =======
SFO1 400.1324710 MHz
NUC1 1H
P1 14.50 usec
PLW1 11.99499989 W

F2 - Processing parameters
SI 65536
SF 400.1300096 MHz
WDW EM
SSB 0
LB 0.30 Hz
GB 0
PC 1.00

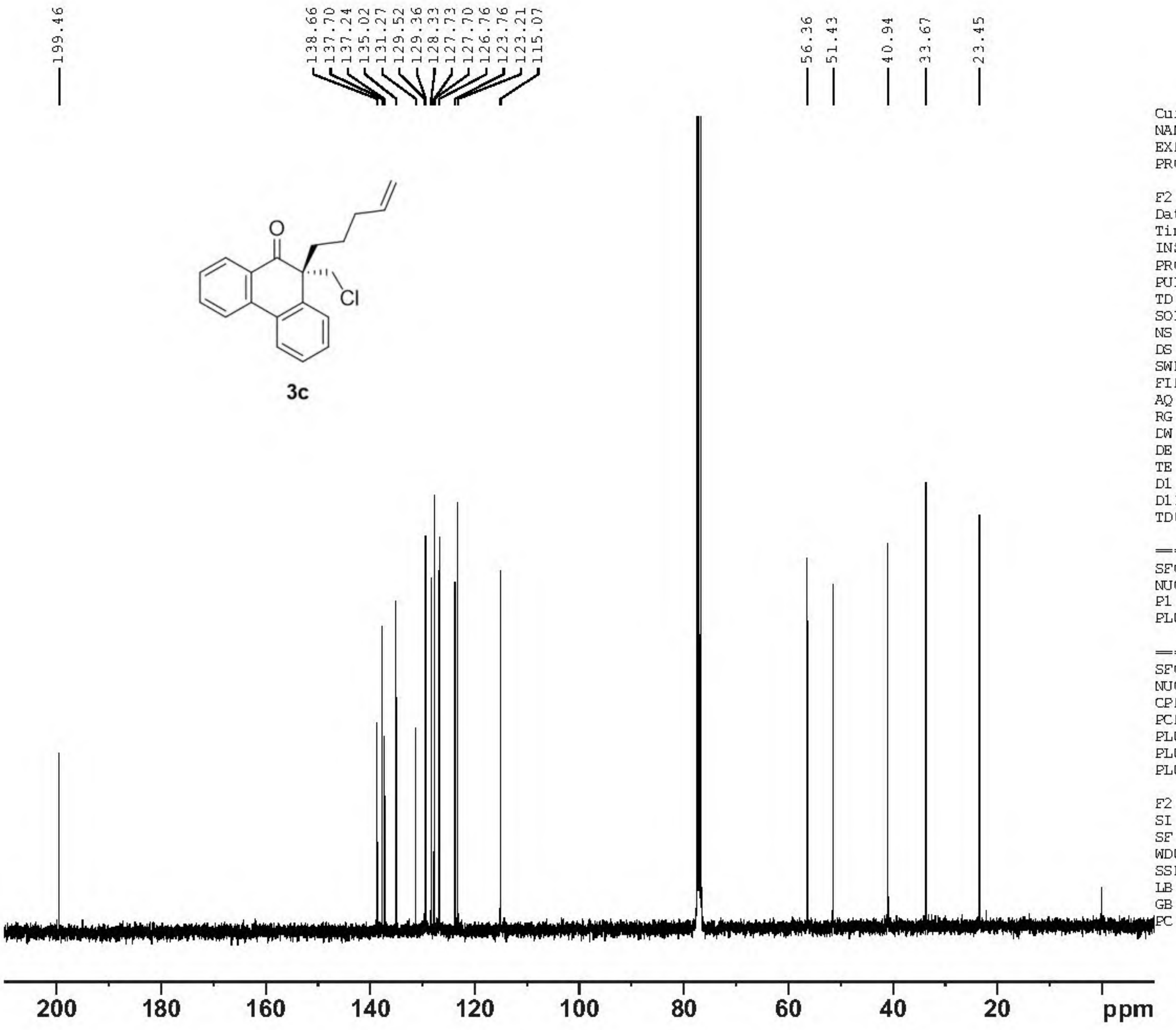


```
Current Data Parameters
NAME      pdt-terminal alkene-C
EXPNO                    1
PROCNO                   1

F2 - Acquisition Parameters
Date_             20240423
Time                  0.44
INSTRUM              spect
PROBHD    5 mm PABBO BB/
PULPROG            zgpg30
TD                   65536
SOLVENT              CDCl3
NS                    2000
DS                       2
SWH             24038.461 Hz
FIDRES           0.366798 Hz
AQ              1.3631488 sec
RG                  196.92
DW                  20.800 usec
DE                    6.50 usec
TE                   298.2 K
D1              2.00000000 sec
D11             0.03000000 sec
TD0                      1

======== CHANNEL f1 ========
SFO1         100.6228298 MHz
NUC1                   13C
P1                    9.70 usec
PLW1          46.98899841 W

======== CHANNEL f2 ========
SFO2         400.1316005 MHz
NUC2                    1H
CPDPRG[2           waltz16
PCPD2                90.00 usec
PLW2          11.99499989 W
PLW12          0.34213999 W
PLW13          0.27713001 W

F2 - Processing parameters
SI                   32768
SF           100.6127663 MHz
WDW                     EM
SSB                      0
LB                    1.00 Hz
GB                       0
PC                    1.40
```

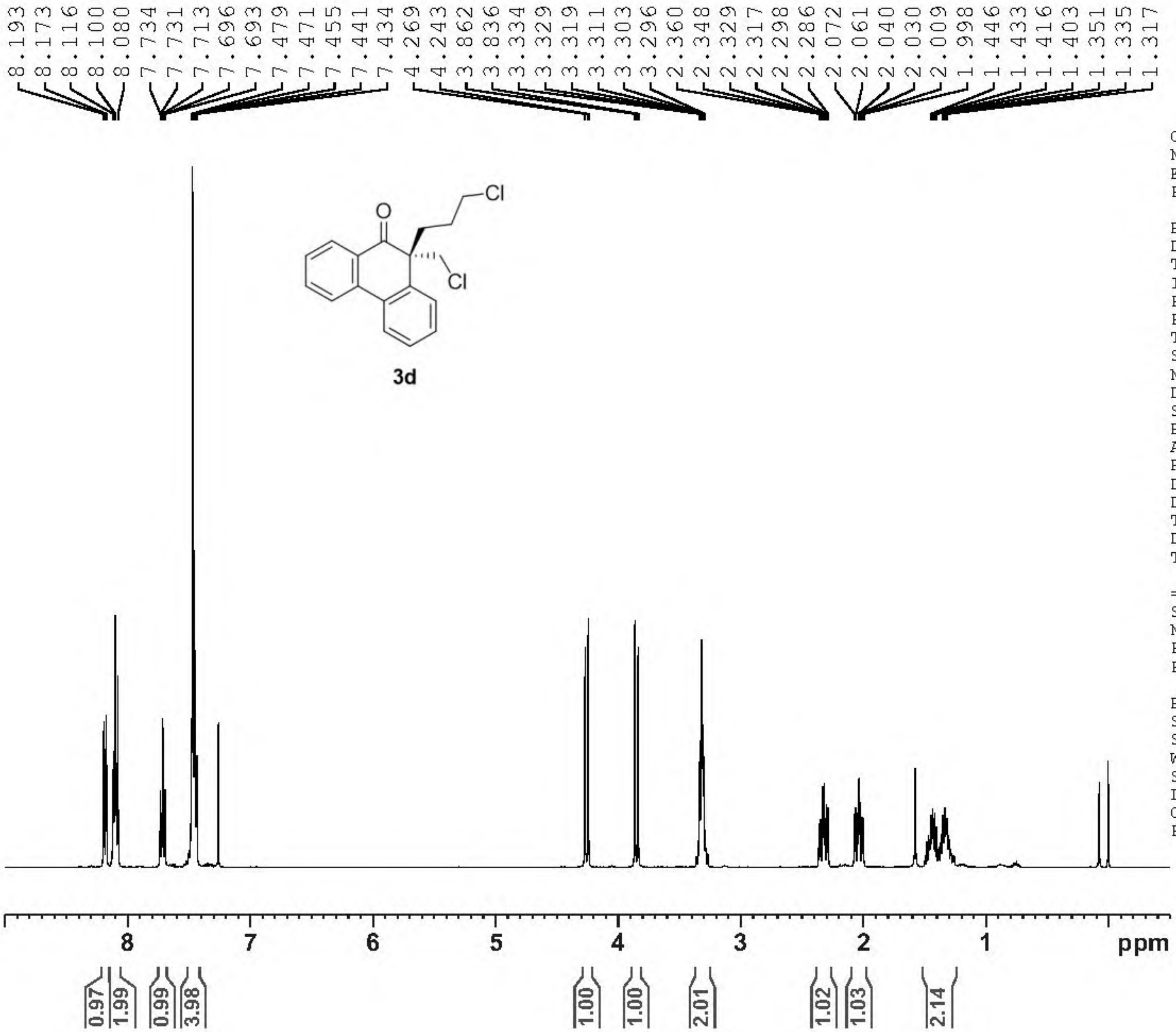


```
Current Data Parameters
NAME              pdt-Cl-H
EXPNO                    1
PROCNO                   1

F2 - Acquisition Parameters
Date_             20240708
Time                 20.07
INSTRUM              spect
PROBHD   5 mm PABBO BB/
PULPROG               zg30
TD                   65536
SOLVENT              CDCl3
NS                      13
DS                       2
SWH               8012.820 Hz
FIDRES            0.122266 Hz
AQ               4.0894465 sec
RG                   88.84
DW                  62.400 usec
DE                    6.50 usec
TE                   297.7 K
D1              1.00000000 sec
TD0                      1

======== CHANNEL f1 ========
SFO1          400.1324710 MHz
NUC1                    1H
P1                   14.50 usec
PLW1           11.99499989 W

F2 - Processing parameters
SI                   65536
SF            400.1300096 MHz
WDW                     EM
SSB                      0
LB                    0.30 Hz
GB                       0
PC                    1.00
```

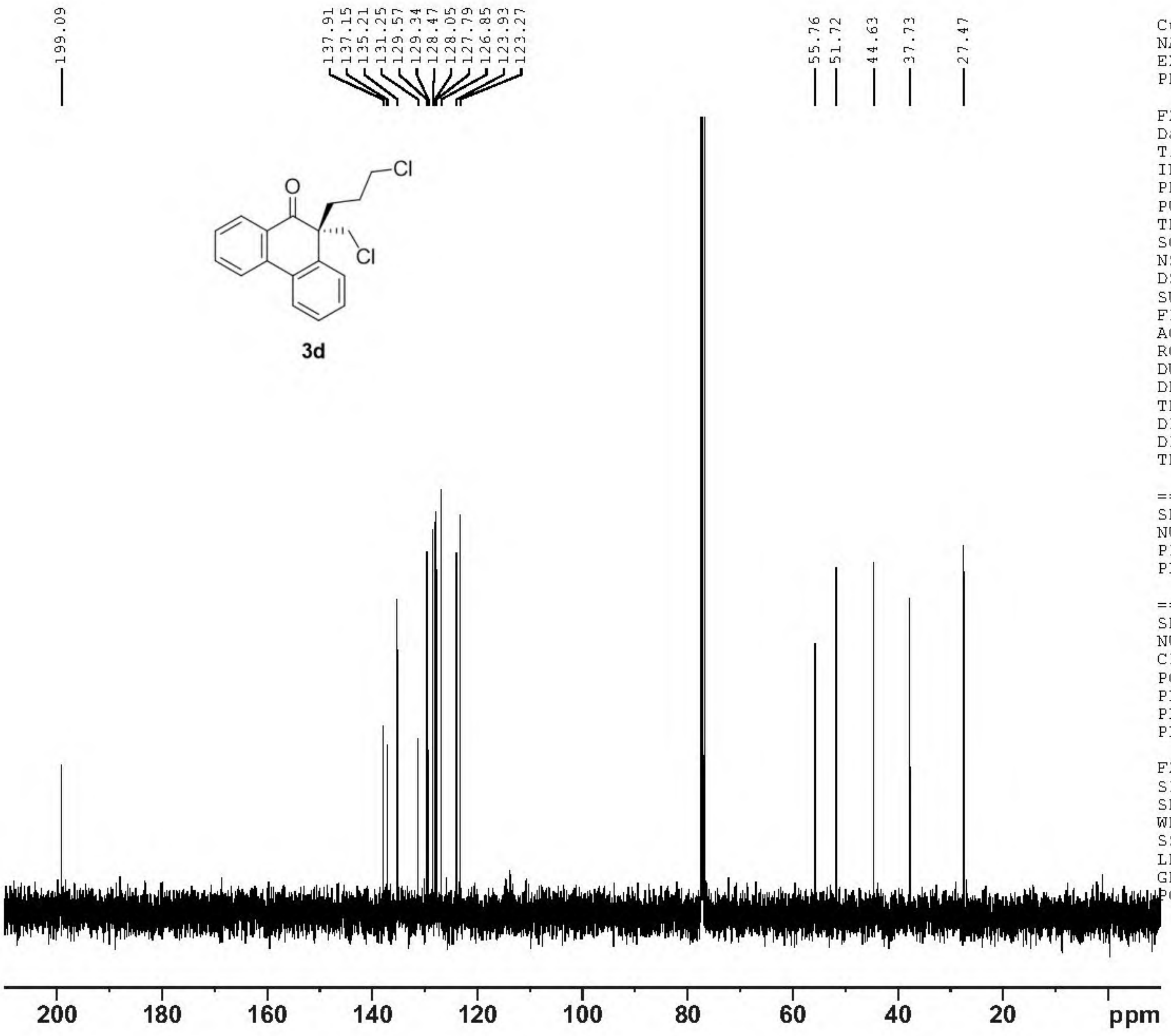


```
Current Data Parameters
NAME             pdt-Cl-C
EXPNO                   1
PROCNO                  1

F2 - Acquisition Parameters
Date_            20240708
Time                20.12
INSTRUM             spect
PROBHD   5 mm PABBO BB/
PULPROG           zgpg30
TD                  65536
SOLVENT             CDCl3
NS                     83
DS                      2
SWH            24038.461 Hz
FIDRES          0.366798 Hz
AQ             1.3631488 sec
RG                 196.92
DW                 20.800 usec
DE                   6.50 usec
TE                  298.4 K
D1             2.00000000 sec
D11            0.03000000 sec
TD0                     1

======== CHANNEL f1 ========
SFO1         100.6228298 MHz
NUC1                  13C
P1                   9.70 usec
PLW1          46.98899841 W

======== CHANNEL f2 ========
SFO2         400.1316005 MHz
NUC2                   1H
CPDPRG[2          waltz16
PCPD2               90.00 usec
PLW2          11.99499989 W
PLW12          0.34213999 W
PLW13          0.27713001 W

F2 - Processing parameters
SI                  32768
SF           100.6127666 MHz
WDW                    EM
SSB                     0
LB                   1.00 Hz
GB                      0
PC                   1.40
```

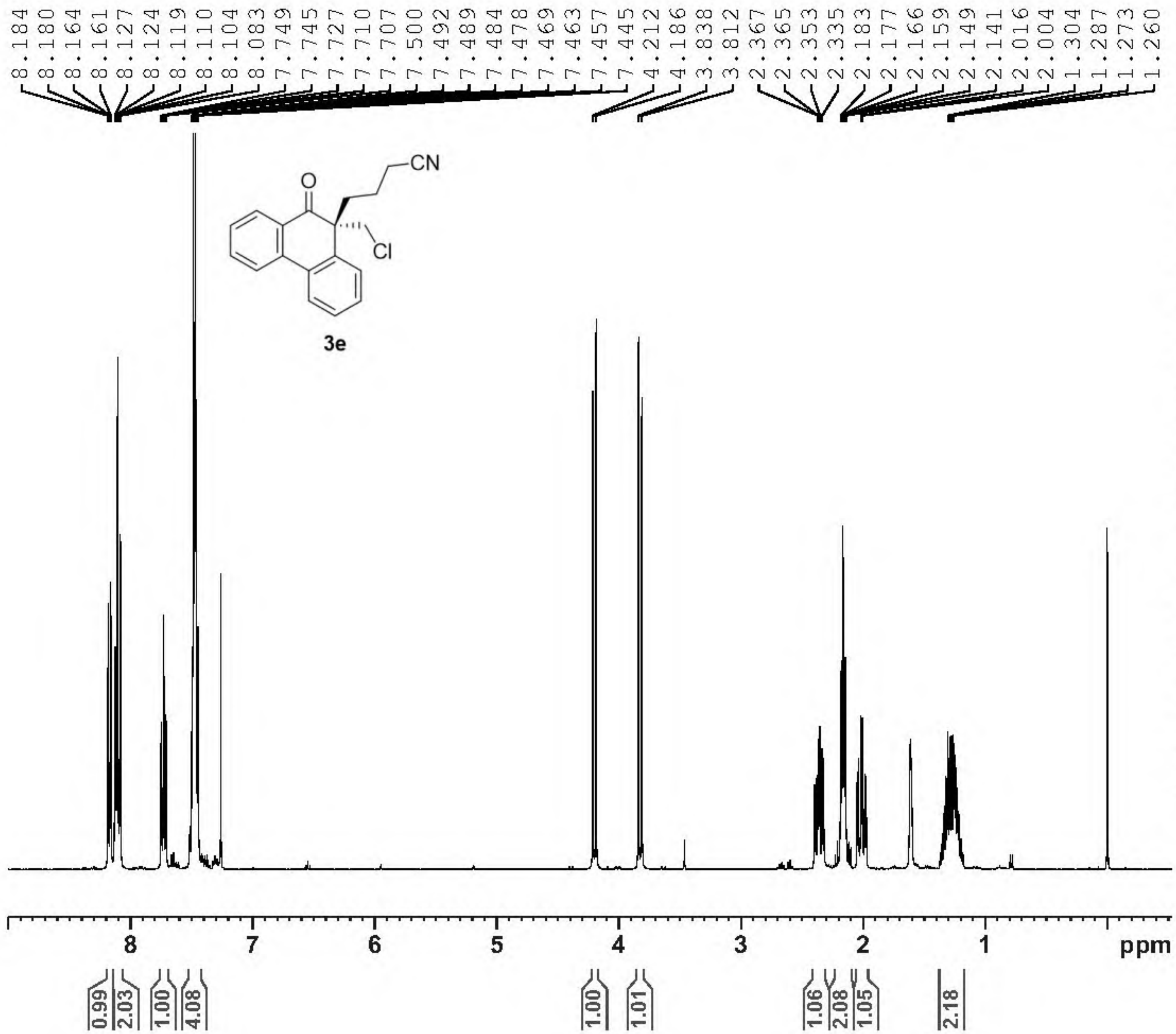

Current Data Parameters
NAME pdt-CN-H
EXPNO 1
PROCNO 1

F2 - Acquisition Parameters
Date_ 20240410
Time 19.09
INSTRUM spect
PROBHD 5 mm DUL 13C-1
PULPROG zg30
TD 65536
SOLVENT CDCl3
NS 8
DS 2
SWH 8223.685 Hz
FIDRES 0.125483 Hz
AQ 3.9845889 sec
RG 362
DW 60.800 usec
DE 6.00 usec
TE 292.4 K
D1 1.00000000 sec
TD0 1

======== CHANNEL f1 ========
NUC1 1H
P1 15.80 usec
PL1 -1.00 dB
PL1W 12.17476940 W
SFO1 400.1324710 MHz

F2 - Processing parameters
SI 32768
SF 400.1300098 MHz
WDW EM
SSB 0
LB 0.30 Hz
GB 0
PC 1.00

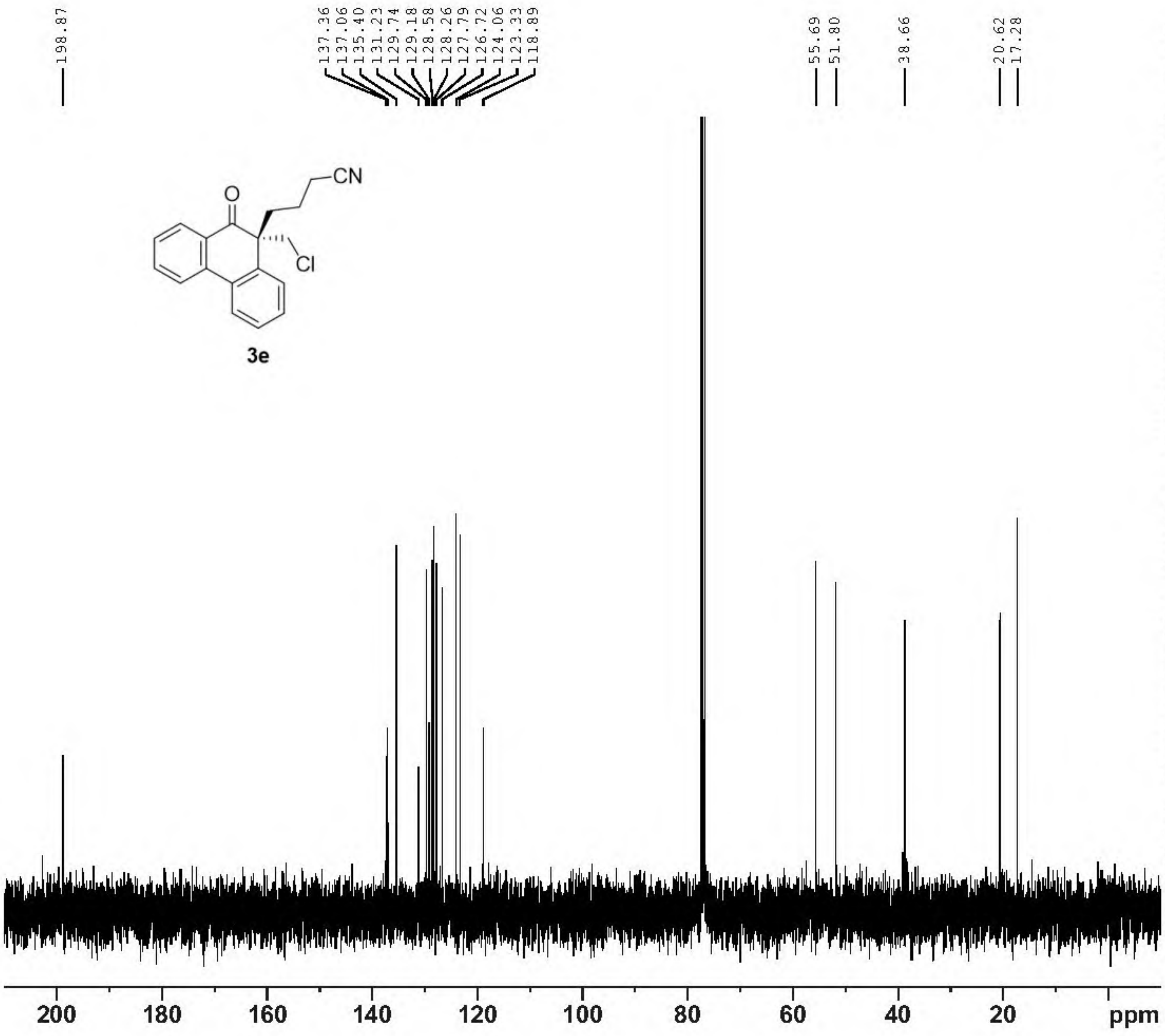


```
Current Data Parameters
NAME              pdt-CN-C
EXPNO                    1
PROCNO                   1

F2 - Acquisition Parameters
Date_             20240410
Time                 19.30
INSTRUM              spect
PROBHD   5 mm DUL 13C-1
PULPROG            zgpg30
TD                   65536
SOLVENT              CDCl3
NS                     329
DS                       1
SWH             24038.461 Hz
FIDRES           0.366798 Hz
AQ              1.3631488 sec
RG                    80.6
DW                  20.800 usec
DE                    6.00 usec
TE                   292.7 K
D1              2.00000000 sec
D11             0.03000000 sec
TD0                      1

======== CHANNEL f1 ========
NUC1                   13C
P1                   40.00 usec
PL1                  -3.00 dB
PL1W           60.64365387 W
SFO1           100.6228298 MHz

======== CHANNEL f2 ========
CPDPRG[2          waltz16
NUC2                    1H
PCPD2                80.00 usec
PL2                  -1.00 dB
PL12                 14.39 dB
PL13                 18.00 dB
PL2W           12.17476940 W
PL12W           0.35193357 W
PL13W           0.15327126 W
SFO2           400.1316005 MHz

F2 - Processing parameters
SI                   32768
SF             100.6127692 MHz
WDW                     EM
SSB                      0
LB                    1.00 Hz
GB                       0
PC                    1.40
```

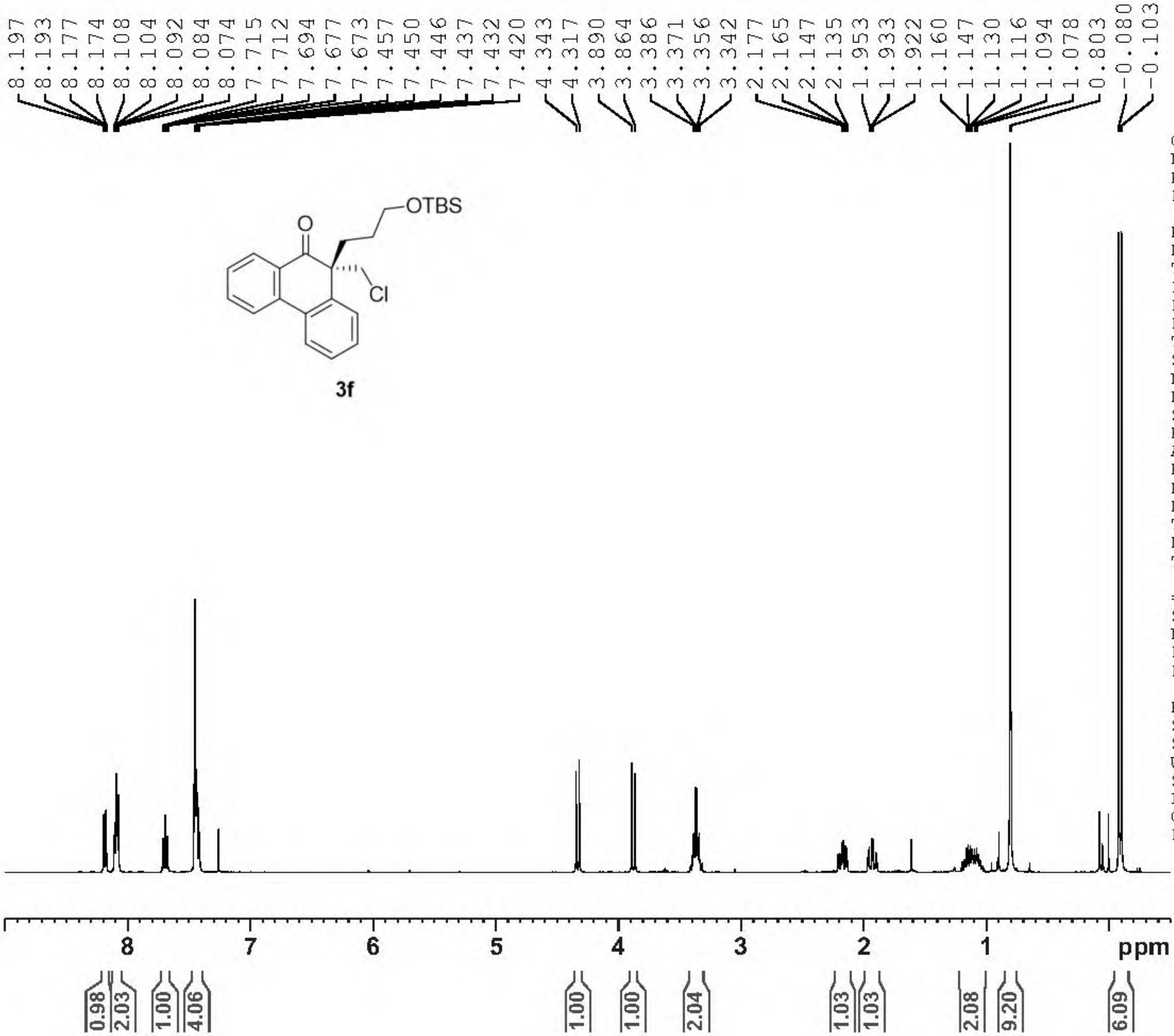

Current Data Parameters
NAME pdt-OTBS-H
EXPNO 1
PROCNO 1

F2 - Acquisition Parameters
Date_ 20240304
Time 19.27
INSTRUM spect
PROBHD 5 mm PABBO BB/
PULPROG zg30
TD 65536
SOLVENT CDCl3
NS 16
DS 2
SWH 8012.820 Hz
FIDRES 0.122266 Hz
AQ 4.0894465 sec
RG 31.55
DW 62.400 usec
DE 6.50 usec
TE 295.6 K
D1 1.00000000 sec
TD0 1

======== CHANNEL f1 ========
SFO1 400.1324710 MHz
NUC1 1H
P1 14.50 usec
PLW1 11.99499989 W

F2 - Processing parameters
SI 65536
SF 400.1300097 MHz
WDW EM
SSB 0
LB 0.30 Hz
GB 0
PC 1.00

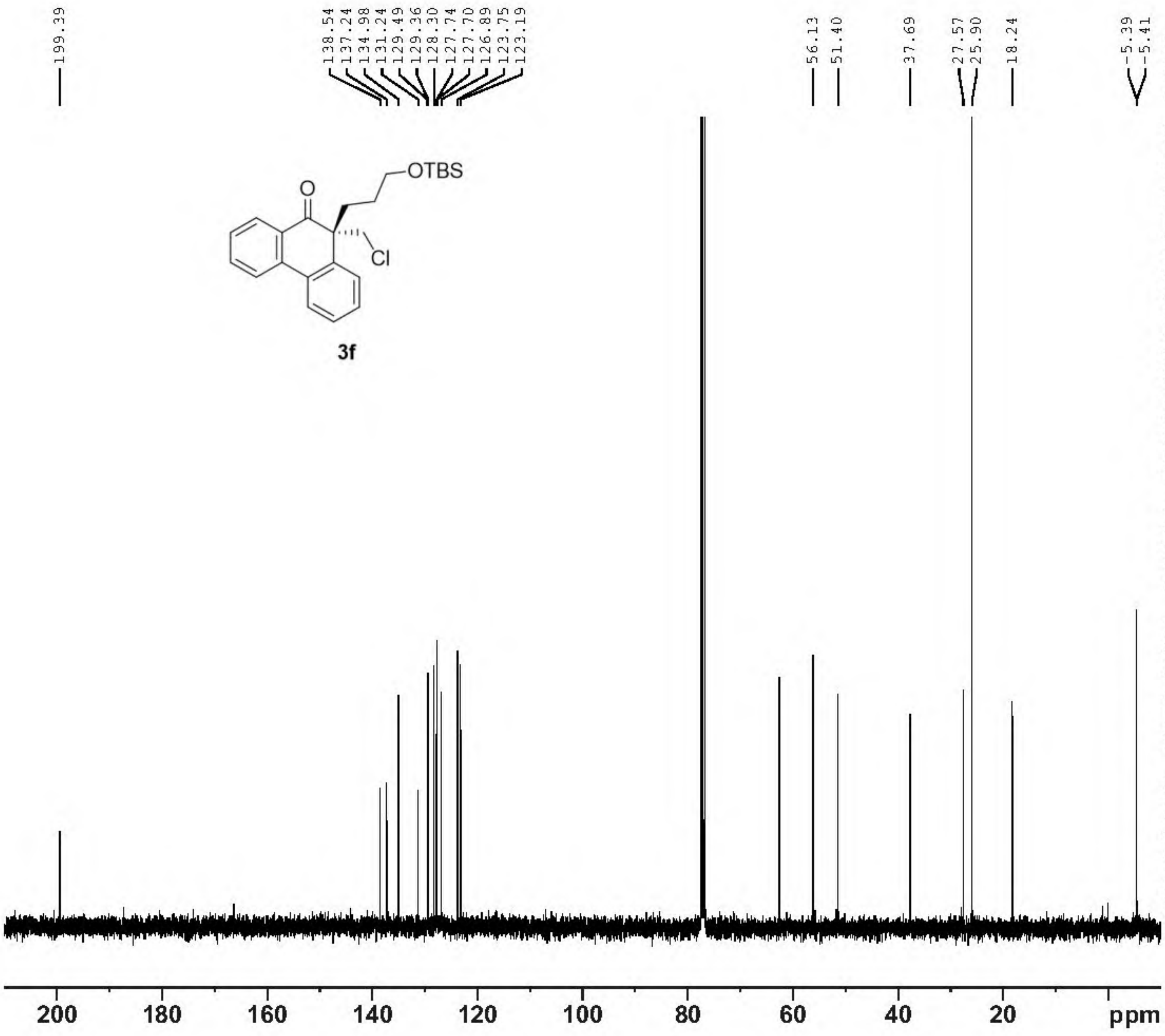

Current Data Parameters
NAME pdt-OTBS-C
EXPNO 1
PROCNO 1

F2 - Acquisition Parameters
Date_ 20240304
Time_ 19.32
INSTRUM spect
PROBHD 5 mm PABBO BB/
PULPROG zgpg30
TD 65536
SOLVENT CDCl3
NS 154
DS 2
SWH 24038.461 Hz
FIDRES 0.366798 Hz
AQ 1.3631488 sec
RG 196.92
DW 20.800 usec
DE 6.50 usec
TE 296.5 K
D1 2.00000000 sec
D11 0.03000000 sec
TD0 1

======== CHANNEL f1 ========
SFO1 100.6228298 MHz
NUC1 13C
P1 9.70 usec
PLW1 46.98899841 W

======== CHANNEL f2 ========
SFO2 400.1316005 MHz
NUC2 1H
CPDPRG[2 waltz16
PCPD2 90.00 usec
PLW2 11.99499989 W
PLW12 0.34213999 W
PLW13 0.27713001 W

F2 - Processing parameters
SI 32768
SF 100.6127674 MHz
WDW EM
SSB 0
LB 1.00 Hz
GB 0
PC 1.40

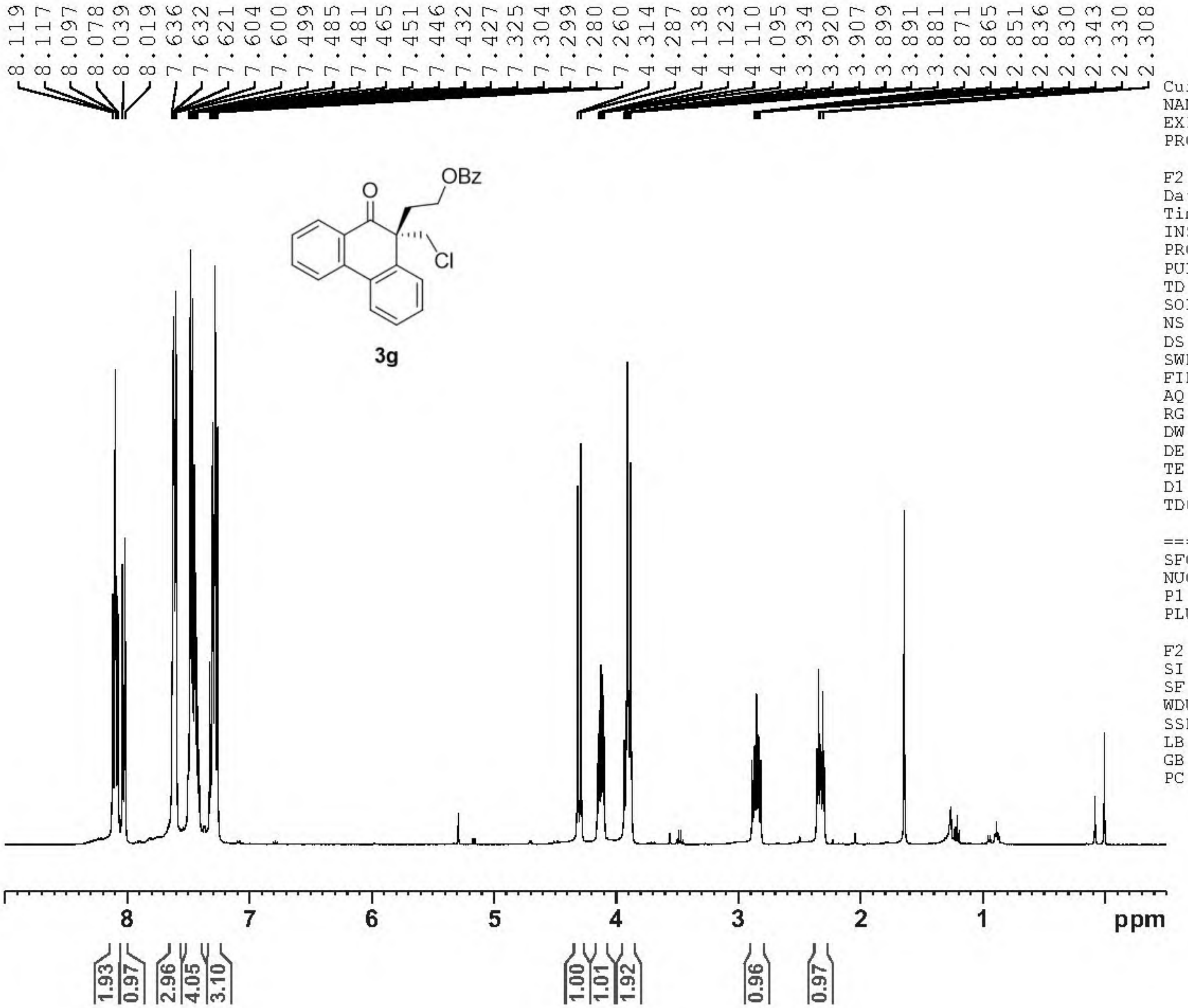

Current Data Parameters
NAME pdt-OBz-H
EXPNO 1
PROCNO 1

F2 - Acquisition Parameters
Date_ 20240531
Time_ 22.58
INSTRUM spect
PROBHD 5 mm PABBO BB/
PULPROG zg30
TD 65536
SOLVENT CDCl3
NS 16
DS 2
SWH 8012.820 Hz
FIDRES 0.122266 Hz
AQ 4.0894465 sec
RG 70.97
DW 62.400 usec
DE 6.50 usec
TE 296.3 K
D1 1.00000000 sec
TD0 1

======== CHANNEL f1 ========
SFO1 400.1324710 MHz
NUC1 1H
P1 14.50 usec
PLW1 11.99499989 W

F2 - Processing parameters
SI 65536
SF 400.1300098 MHz
WDW EM
SSB 0
LB 0.30 Hz
GB 0
PC 1.00

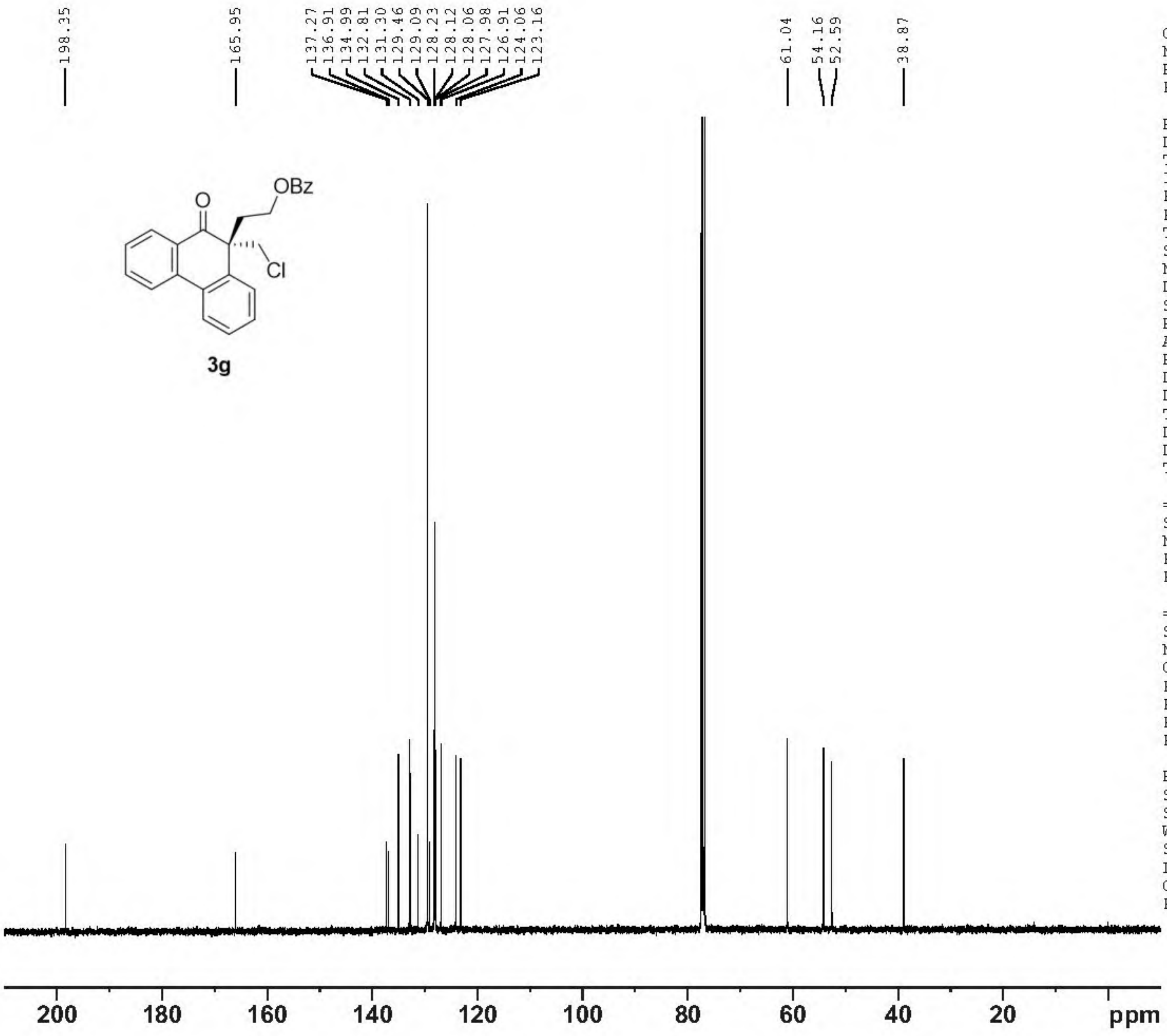

Current Data Parameters
NAME pdt-OBz-C
EXPNO 1
PROCNO 1

F2 - Acquisition Parameters
Date_ 20240531
Time 23.56
INSTRUM spect
PROBHD 5 mm PABBO BB/
PULPROG zgpg30
TD 65536
SOLVENT CDCl3
NS 1000
DS 2
SWH 24038.461 Hz
FIDRES 0.366798 Hz
AQ 1.3631488 sec
RG 196.92
DW 20.800 usec
DE 6.50 usec
TE 297.7 K
D1 2.00000000 sec
D11 0.03000000 sec
TD0 1

======== CHANNEL f1 ========
SFO1 100.6228298 MHz
NUC1 13C
P1 9.70 usec
PLW1 46.98899841 W

======== CHANNEL f2 ========
SFO2 400.1316005 MHz
NUC2 1H
CPDPRG[2 waltz16
PCPD2 90.00 usec
PLW2 11.99499989 W
PLW12 0.34213999 W
PLW13 0.27713001 W

F2 - Processing parameters
SI 32768
SF 100.6127688 MHz
WDW EM
SSB 0
LB 1.00 Hz
GB 0
PC 1.40

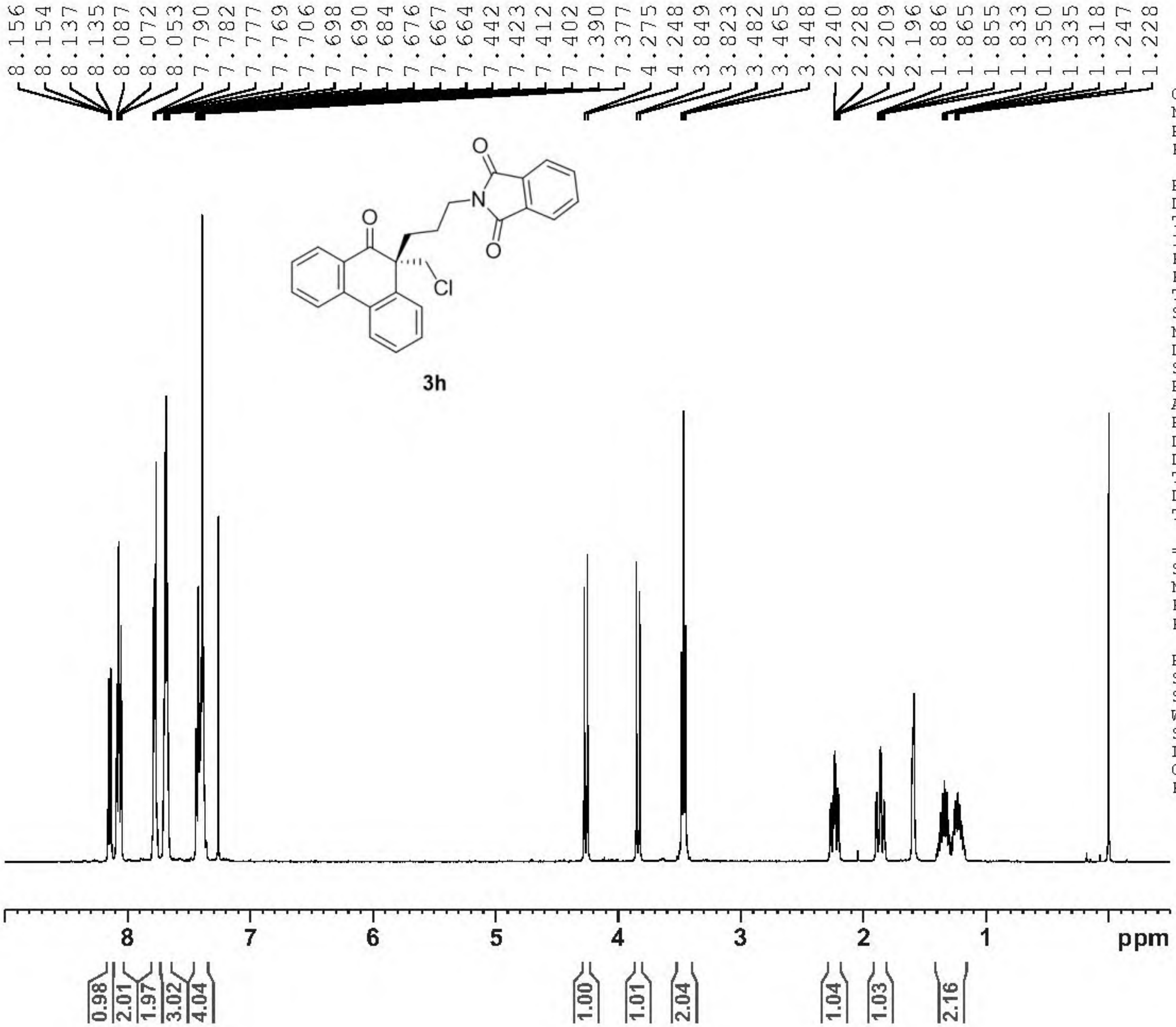


```
Current Data Parameters
NAME          pdt-NthPh-H
EXPNO                   1
PROCNO                  1

F2 - Acquisition Parameters
Date_            20240327
Time                23.00
INSTRUM             spect
PROBHD   5 mm PABBO BB/
PULPROG              zg30
TD                  65536
SOLVENT             CDCl3
NS                     16
DS                      2
SWH              8012.820 Hz
FIDRES           0.122266 Hz
AQ              4.0894465 sec
RG                 126.97
DW                 62.400 usec
DE                   6.50 usec
TE                  296.8 K
D1             1.00000000 sec
TD0                     1

======== CHANNEL f1 ========
SFO1          400.1324710 MHz
NUC1                   1H
P1                  14.50 usec
PLW1          11.99499989 W

F2 - Processing parameters
SI                  65536
SF            400.1300094 MHz
WDW                    EM
SSB                     0
LB                   0.30 Hz
GB                      0
PC                   1.00
```

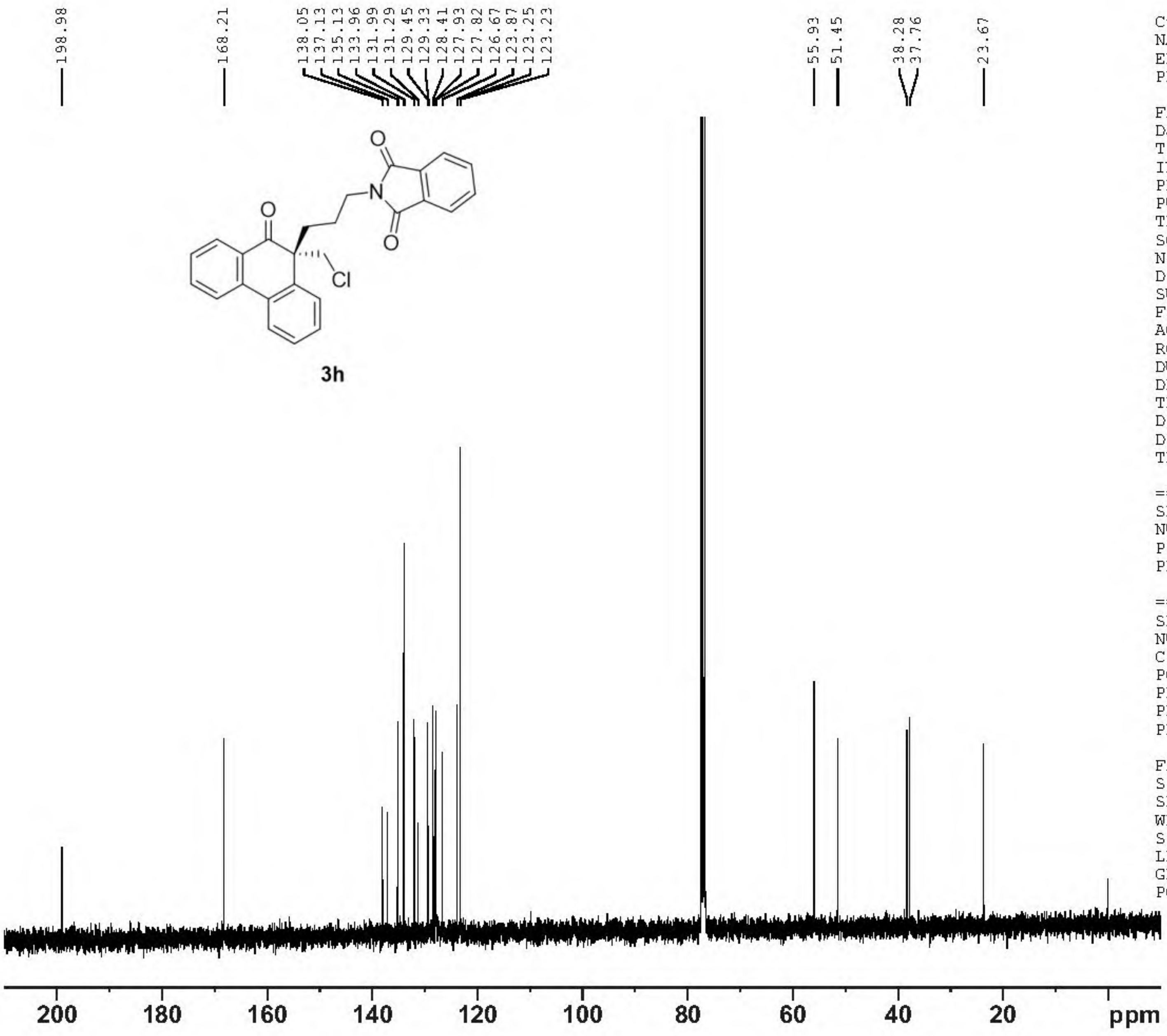

Current Data Parameters
NAME pdt-NthPh-C
EXPNO 1
PROCNO 1

F2 - Acquisition Parameters
Date_ 20240327
Time 23.58
INSTRUM spect
PROBHD 5 mm PABBO BB/
PULPROG zgpg30
TD 65536
SOLVENT CDCl3
NS 1000
DS 2
SWH 24038.461 Hz
FIDRES 0.366798 Hz
AQ 1.3631488 sec
RG 196.92
DW 20.800 usec
DE 6.50 usec
TE 297.9 K
D1 2.00000000 sec
D11 0.03000000 sec
TD0 1

======== CHANNEL f1 ========
SFO1 100.6228298 MHz
NUC1 13C
P1 9.70 usec
PLW1 46.98899841 W

======== CHANNEL f2 ========
SFO2 400.1316005 MHz
NUC2 1H
CPDPRG[2 waltz16
PCPD2 90.00 usec
PLW2 11.99499989 W
PLW12 0.34213999 W
PLW13 0.27713001 W

F2 - Processing parameters
SI 32768
SF 100.6127659 MHz
WDW EM
SSB 0
LB 1.00 Hz
GB 0
PC 1.40

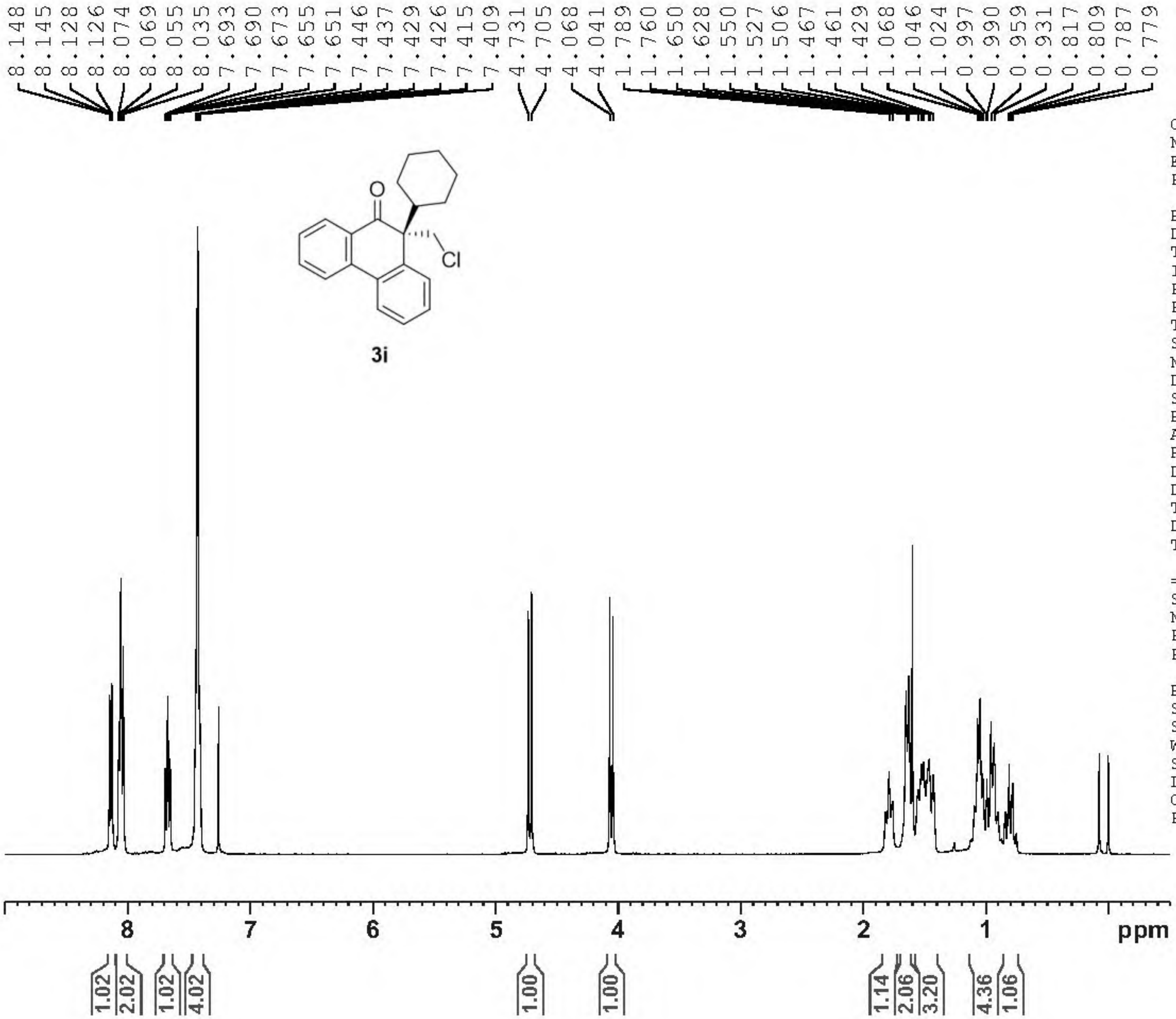

Current Data Parameters
NAME zzj-Cy-H
EXPNO 1
PROCNO 1

F2 - Acquisition Parameters
Date_ 20240601
Time 0
INSTRUM spect
PROBHD 5 mm PABBO BB/
PULPROG zg30
TD 65536
SOLVENT CDCl3
NS 16
DS 2
SWH 8012.820 Hz
FIDRES 0.122266 Hz
AQ 4.0894465 sec
RG 70.97
DW 62.400 usec
DE 6.50 usec
TE 296.8 K
D1 1.00000000 sec
TD0 1

======== CHANNEL f1 ========
SFO1 400.1324710 MHz
NUC1 1H
P1 14.50 usec
PLW1 11.99499989 W

F2 - Processing parameters
SI 65536
SF 400.1300101 MHz
WDW EM
SSB 0
LB 0.30 Hz
GB 0
PC 1.00

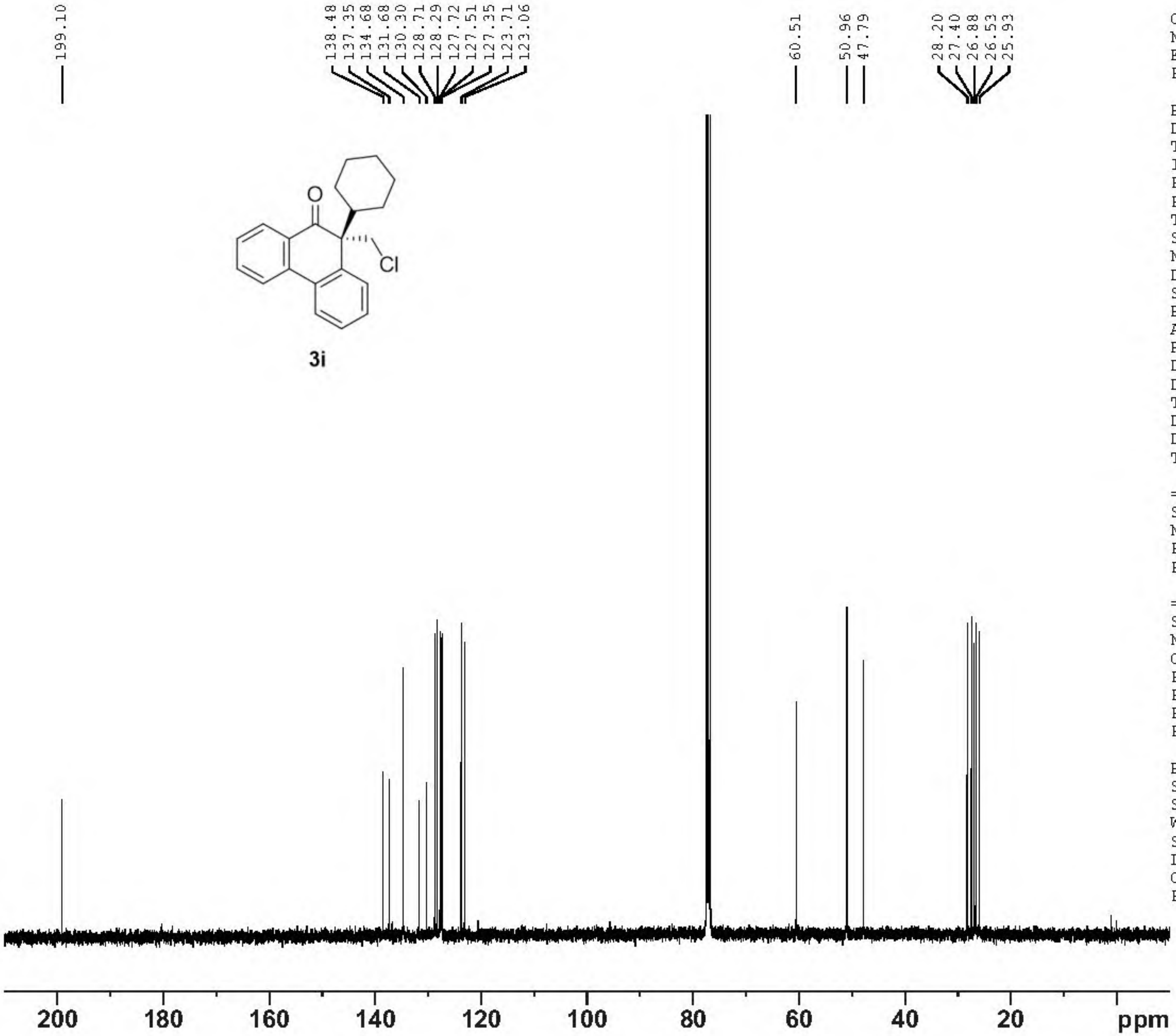

Current Data Parameters
NAME zzj-Cy-C
EXPNO 1
PROCNO 1

F2 - Acquisition Parameters
Date_ 20240601
Time 0.58
INSTRUM spect
PROBHD 5 mm PABBO BB/
PULPROG zgpg30
TD 65536
SOLVENT CDCl3
NS 1000
DS 2
SWH 24038.461 Hz
FIDRES 0.366798 Hz
AQ 1.3631488 sec
RG 196.92
DW 20.800 usec
DE 6.50 usec
TE 297.8 K
D1 2.00000000 sec
D11 0.03000000 sec
TD0 1

======== CHANNEL f1 ========
SFO1 100.6228298 MHz
NUC1 13C
P1 9.70 usec
PLW1 46.98899841 W

======== CHANNEL f2 ========
SFO2 400.1316005 MHz
NUC2 1H
CPDPRG[2 waltz16
PCPD2 90.00 usec
PLW2 11.99499989 W
PLW12 0.34213999 W
PLW13 0.27713001 W

F2 - Processing parameters
SI 32768
SF 100.6127673 MHz
WDW EM
SSB 0
LB 1.00 Hz
GB 0
PC 1.40

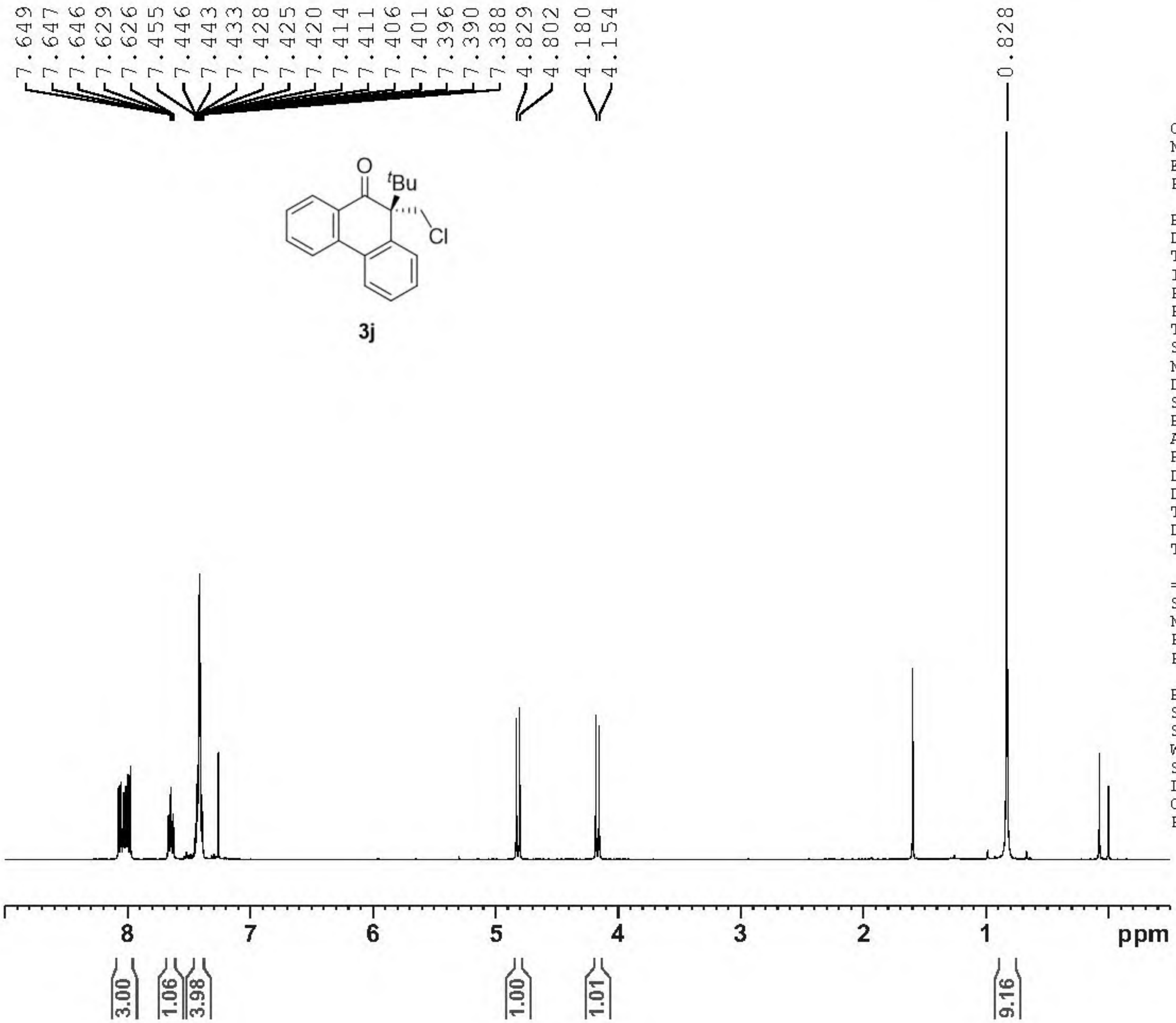


```
Current Data Parameters
NAME            pdt-tBu-H
EXPNO                   1
PROCNO                  1

F2 - Acquisition Parameters
Date_            20240503
Time                22.59
INSTRUM             spect
PROBHD   5 mm PABBO BB/
PULPROG              zg30
TD                  65536
SOLVENT             CDCl3
NS                     16
DS                      2
SWH             8012.820 Hz
FIDRES          0.122266 Hz
AQ             4.0894465 sec
RG                  82.92
DW                 62.400 usec
DE                   6.50 usec
TE                  295.9 K
D1            1.00000000 sec
TD0                     1

======== CHANNEL f1 ========
SFO1         400.1324710 MHz
NUC1                   1H
P1                  14.50 usec
PLW1          11.99499989 W

F2 - Processing parameters
SI                  65536
SF           400.1300098 MHz
WDW                    EM
SSB                     0
LB                   0.30 Hz
GB                      0
PC                   1.00
```

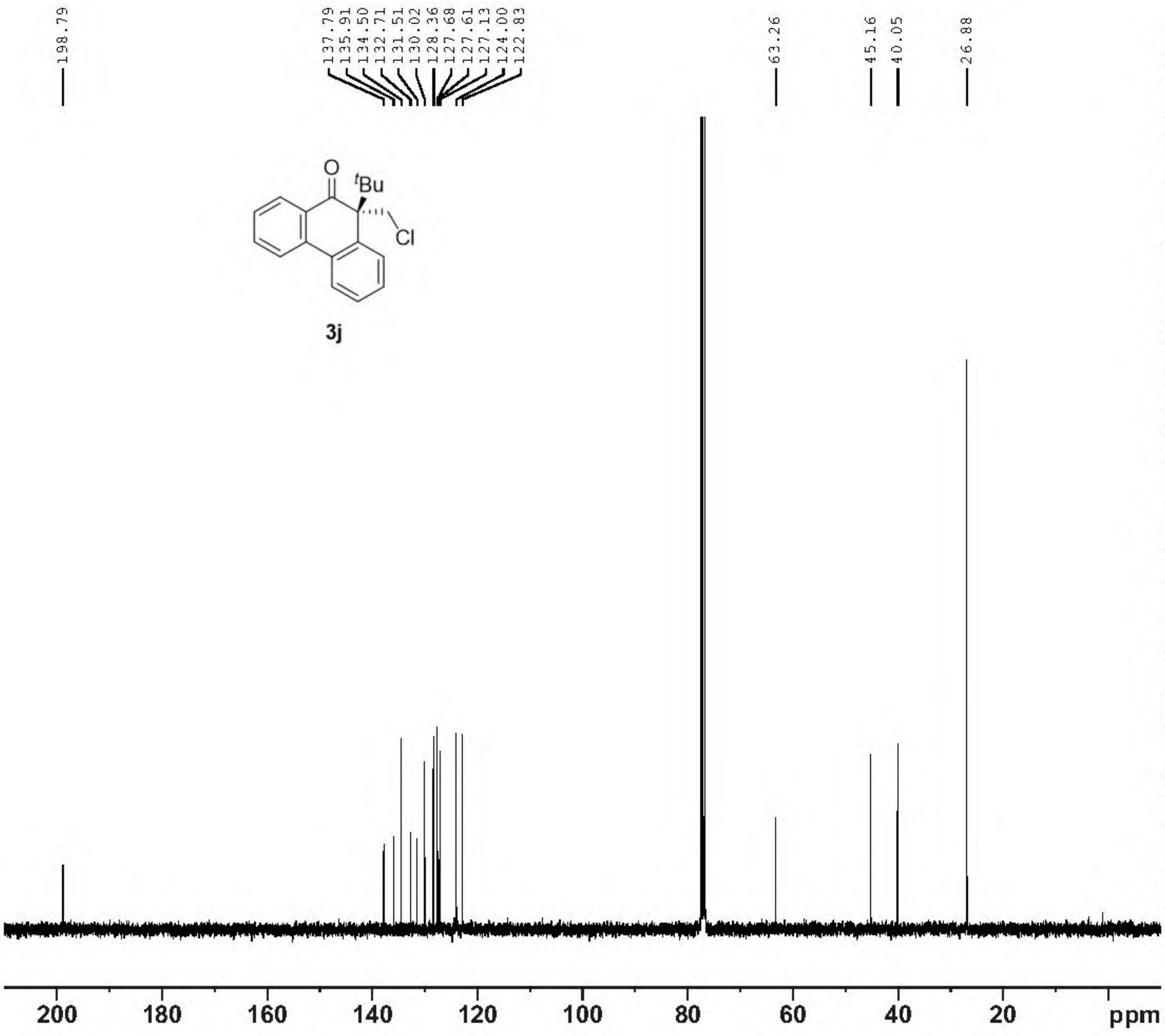

Current Data Parameters
NAME pdt-tBu-C
EXPNO 2
PROCNO 1

F2 - Acquisition Parameters
Date_ 20240504
Time 10.57
INSTRUM spect
PROBHD 5 mm PABBO BB/
PULPROG zgpg30
TD 65536
SOLVENT CDCl3
NS 400
DS 2
SWH 24038.461 Hz
FIDRES 0.366798 Hz
AQ 1.3631488 sec
RG 196.92
DW 20.800 usec
DE 6.50 usec
TE 297.1 K
D1 2.00000000 sec
D11 0.03000000 sec
TD0 1

======== CHANNEL f1 ========
SFO1 100.6228298 MHz
NUC1 13C
P1 9.70 usec
PLW1 46.98899841 W

======== CHANNEL f2 ========
SFO2 400.1316005 MHz
NUC2 1H
CPDPRG[2 waltz16
PCPD2 90.00 usec
PLW2 11.99499989 W
PLW12 0.34213999 W
PLW13 0.27713001 W

F2 - Processing parameters
SI 32768
SF 100.6127666 MHz
WDW EM
SSB 0
LB 1.00 Hz
GB 0
PC 1.40

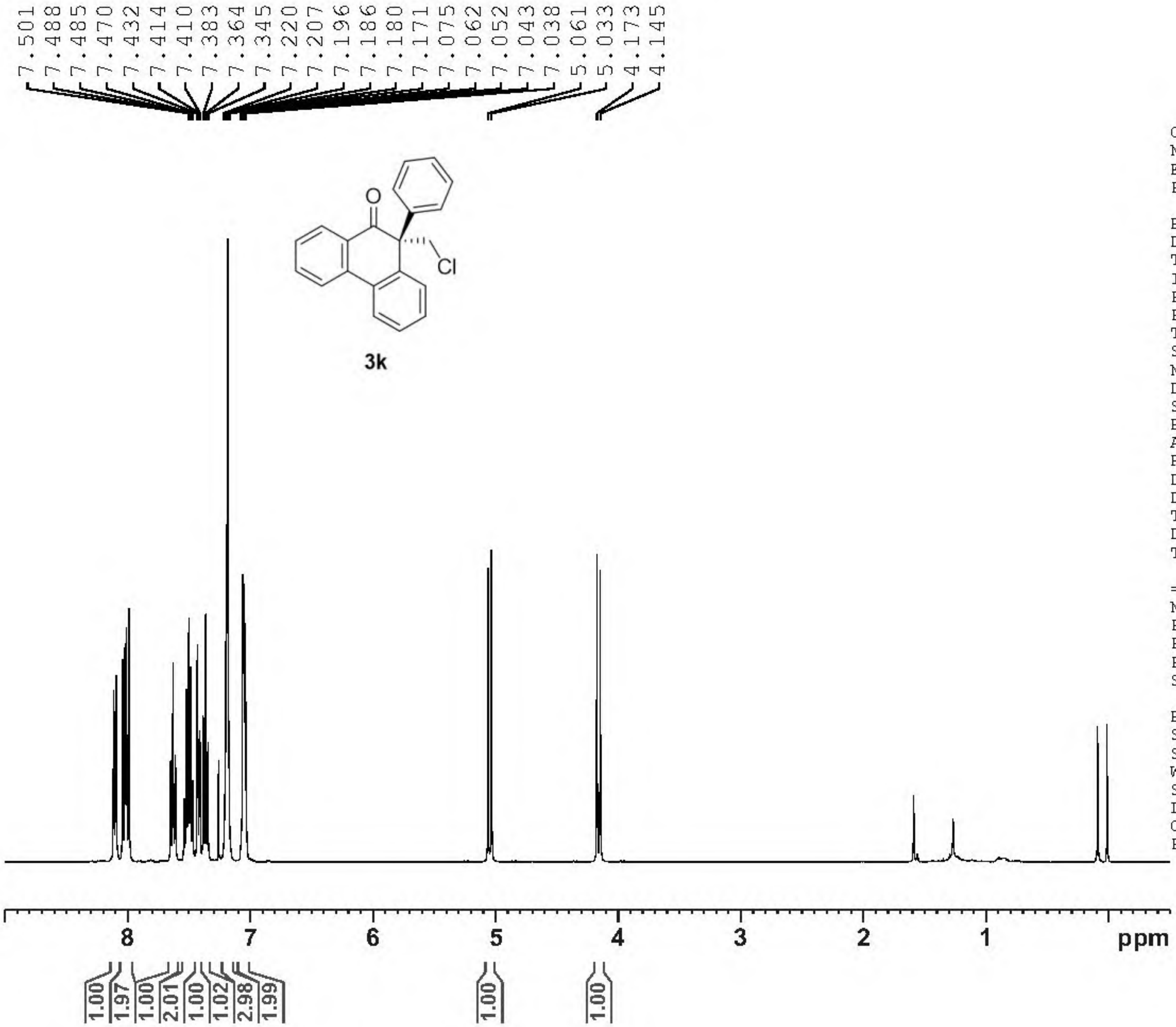

Current Data Parameters
NAME pdt-Ph-H
EXPNO 1
PROCNO 1

F2 - Acquisition Parameters
Date_ 20240313
Time_ 18.16
INSTRUM spect
PROBHD 5 mm DUL 13C-1
PULPROG zg30
TD 65536
SOLVENT CDCl3
NS 7
DS 2
SWH 8223.685 Hz
FIDRES 0.125483 Hz
AQ 3.9845889 sec
RG 322
DW 60.800 usec
DE 6.00 usec
TE 292.3 K
D1 1.00000000 sec
TD0 1

======== CHANNEL f1 ========
NUC1 1H
P1 15.80 usec
PL1 -1.00 dB
PL1W 12.17476940 W
SFO1 400.1324710 MHz

F2 - Processing parameters
SI 32768
SF 400.1300102 MHz
WDW EM
SSB 0
LB 0.30 Hz
GB 0
PC 1.00

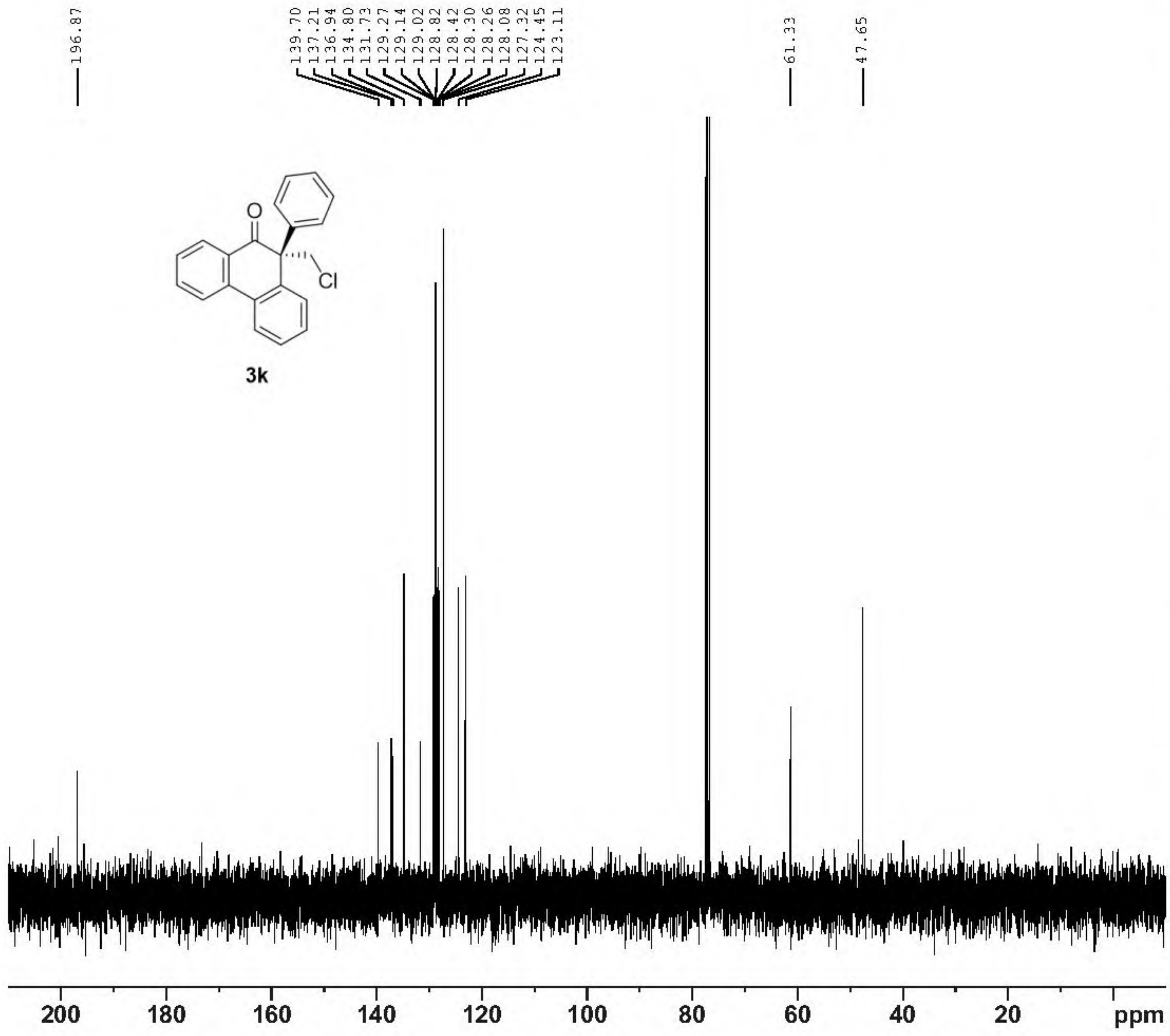


```
Current Data Parameters
NAME             pdt-Ph-C
EXPNO                   1
PROCNO                  1

F2 - Acquisition Parameters
Date_            20240313
Time                18.26
INSTRUM             spect
PROBHD   5 mm DUL 13C-1
PULPROG           zgpg30
TD                  65536
SOLVENT             CDCl3
NS                    133
DS                      1
SWH             24038.461 Hz
FIDRES           0.366798 Hz
AQ              1.3631488 sec
RG                   71.8
DW                 20.800 usec
DE                   6.00 usec
TE                  292.7 K
D1             2.00000000 sec
D11            0.03000000 sec
TD0                     1

======== CHANNEL f1 ========
NUC1                  13C
P1                  40.00 usec
PL1                 -3.00 dB
PL1W          60.64365387 W
SFO1         100.6228298 MHz

======== CHANNEL f2 ========
CPDPRG[2          waltz16
NUC2                   1H
PCPD2               80.00 usec
PL2                 -1.00 dB
PL12                14.39 dB
PL13                18.00 dB
PL2W          12.17476940 W
PL12W          0.35193357 W
PL13W          0.15327126 W
SFO2         400.1316005 MHz

F2 - Processing parameters
SI                  32768
SF           100.6127696 MHz
WDW                    EM
SSB                     0
LB                   1.00 Hz
GB                      0
PC                   1.40
```

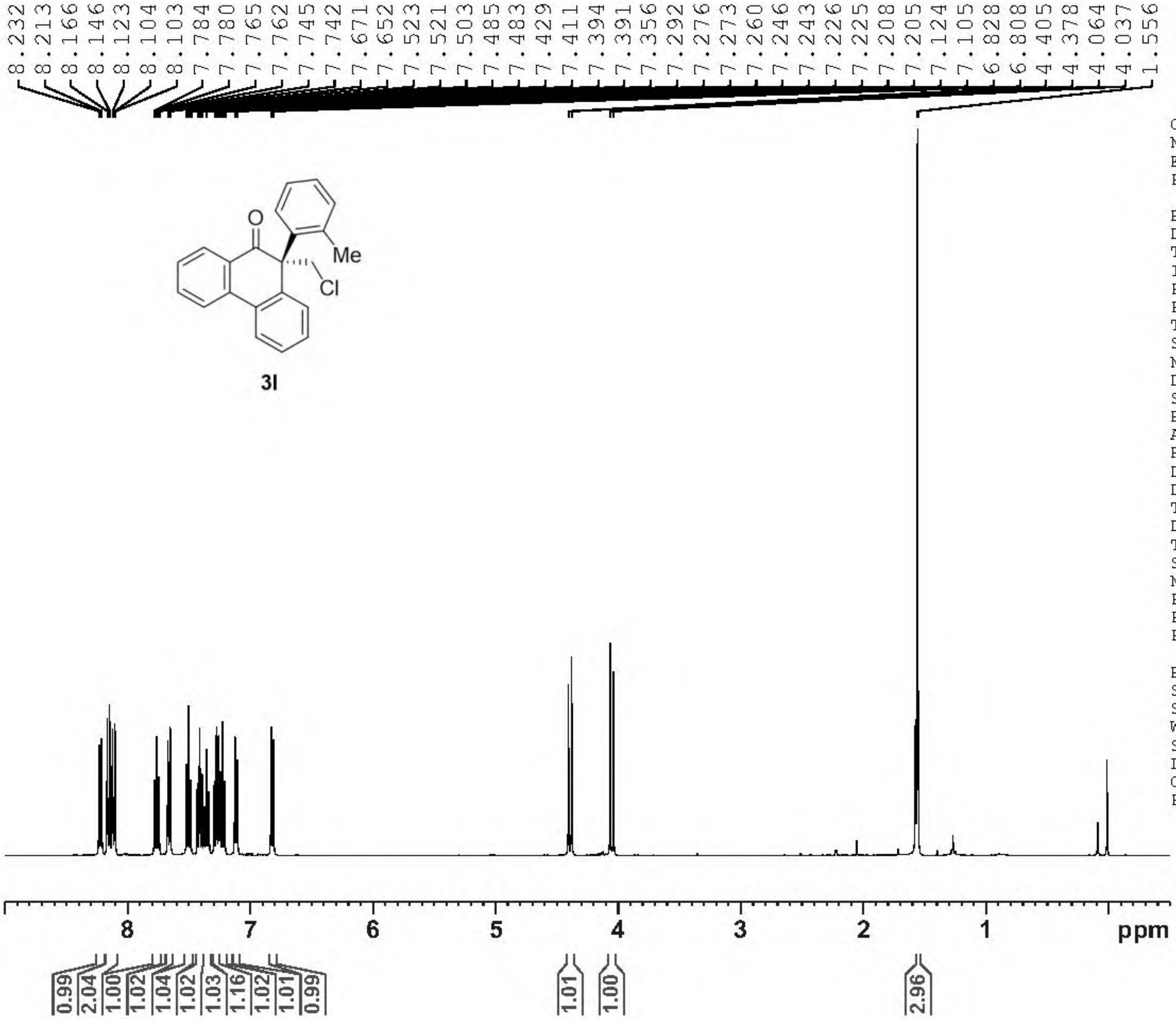

Current Data Parameters
NAME pdt-2-Me-Ph-H
EXPNO 1
PROCNO 1

F2 - Acquisition Parameters
Date_ 20240119
Time_ 23.18 h
INSTRUM AvanceNeo 400MHz
PROBHD Z163739_0629 (
PULPROG zg30
TD 65536
SOLVENT CDCl3
NS 16
DS 2
SWH 8196.722 Hz
FIDRES 0.250144 Hz
AQ 3.9976959 sec
RG 101
DW 61.000 usec
DE 13.89 usec
TE 297.1 K
D1 1.00000000 sec
TD0 1
SFO1 400.1824711 MHz
NUC1 1H
P0 2.67 usec
P1 8.00 usec
PLW1 21.26700020 W

F2 - Processing parameters
SI 65536
SF 400.1800096 MHz
WDW EM
SSB 0
LB 0.30 Hz
GB 0
PC 1.00

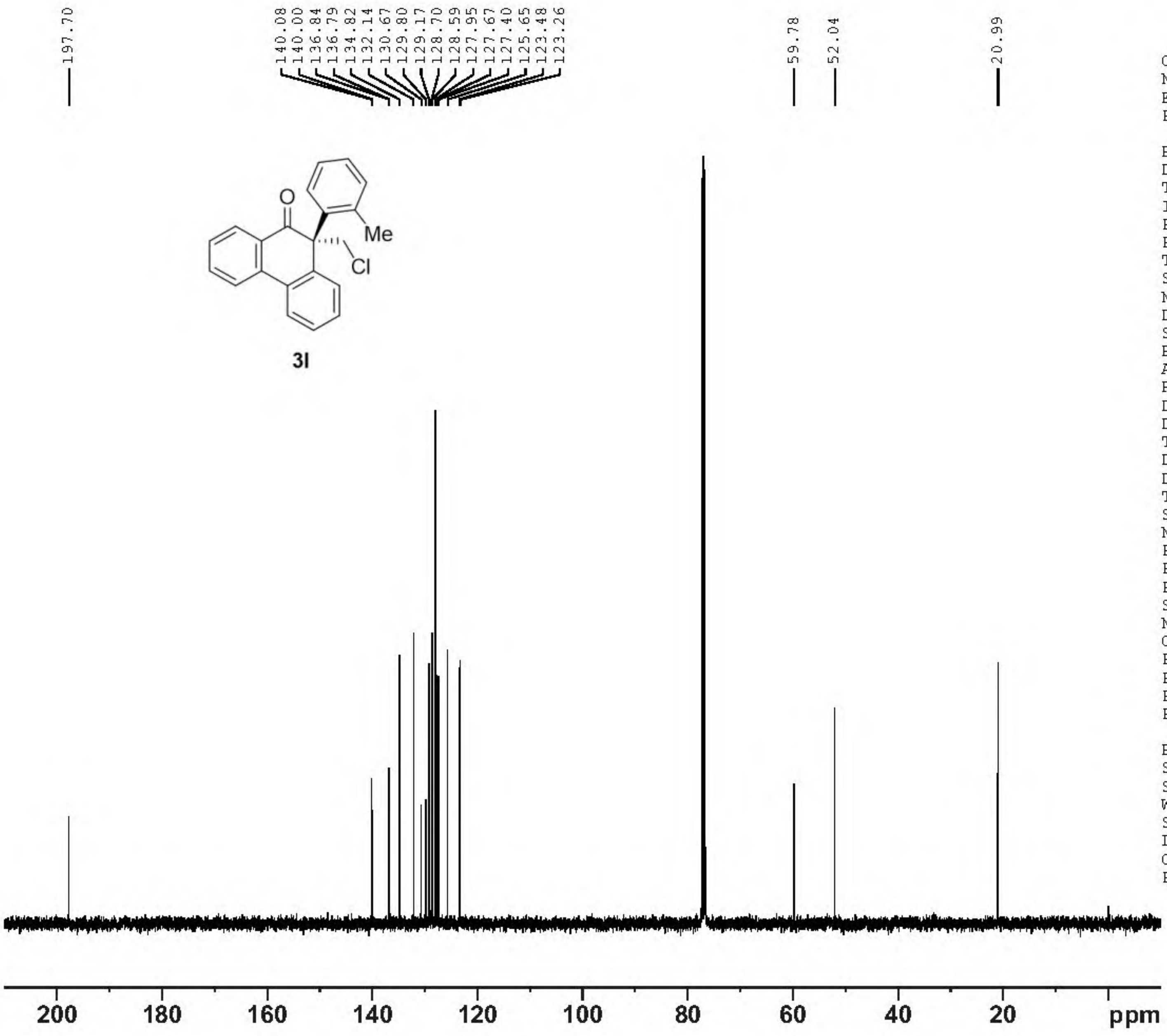

Current Data Parameters
NAME pdt-2-Me-Ph-C
EXPNO 3
PROCNO 1

F2 - Acquisition Parameters
Date_ 20240119
Time_ 23.38 h
INSTRUM AvanceNeo 400MHz
PROBHD Z163739_0629 (
PULPROG zgpg30
TD 65536
SOLVENT CDCl3
NS 304
DS 4
SWH 23809.523 Hz
FIDRES 0.726609 Hz
AQ 1.3762560 sec
RG 10
DW 21.000 usec
DE 6.50 usec
TE 298.1 K
D1 2.00000000 sec
D11 0.03000000 sec
TD0 1
SFO1 100.6354036 MHz
NUC1 13C
P0 2.67 usec
P1 8.00 usec
PLW1 85.25399780 W
SFO2 400.1816007 MHz
NUC2 1H
CPDPRG[2 waltz65
PCPD2 90.00 usec
PLW2 21.26700020 W
PLW12 0.16802999 W
PLW13 0.08452000 W

F2 - Processing parameters
SI 32768
SF 100.6253468 MHz
WDW EM
SSB 0
LB 1.00 Hz
GB 0
PC 1.40

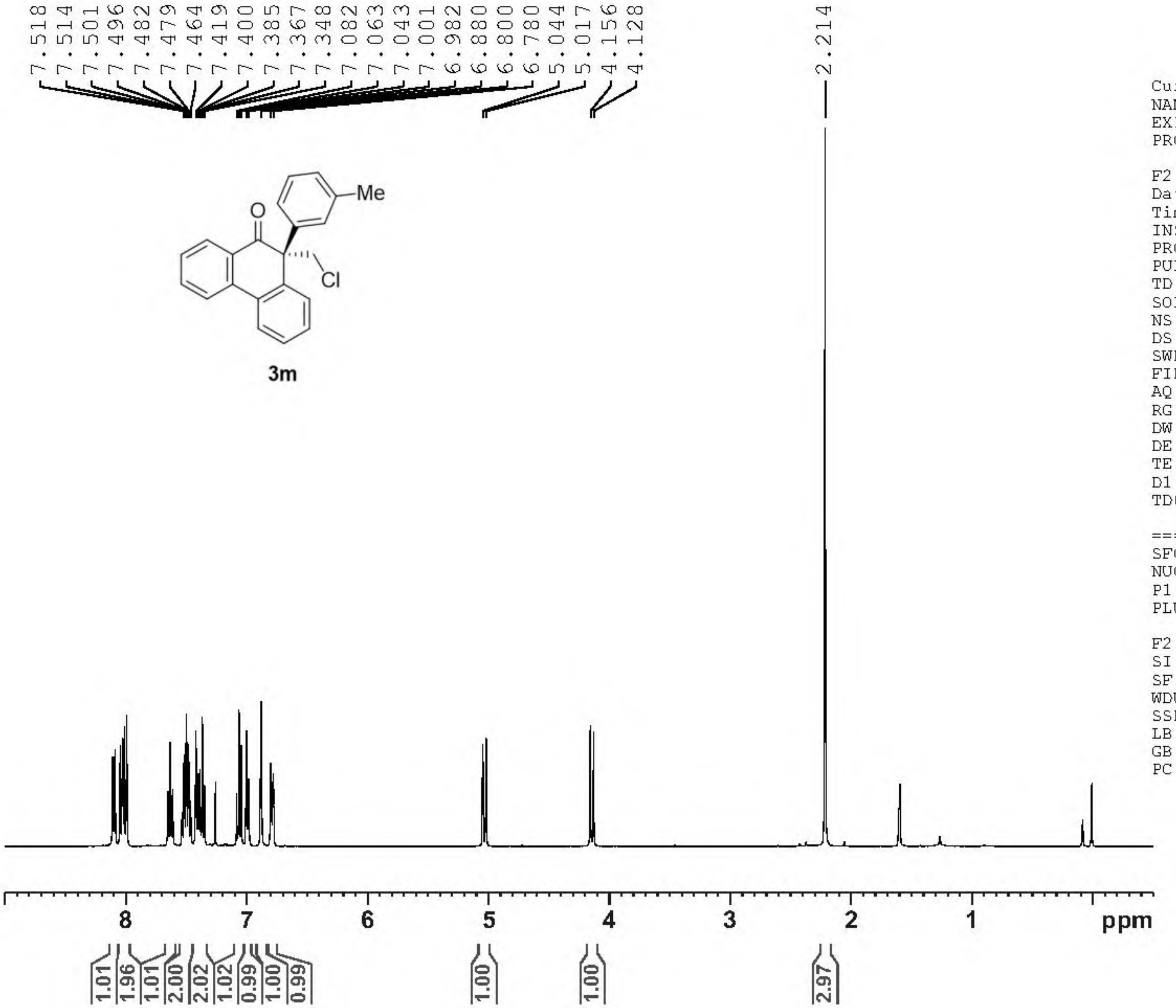

3m

Current Data Parameters
NAME pdt-3-Me-Ph-H
EXPNO 1
PROCNO 1

F2 - Acquisition Parameters
Date_ 20240124
Time 23.00
INSTRUM spect
PROBHD 5 mm PABBO BB/
PULPROG zg30
TD 65536
SOLVENT CDCl3
NS 16
DS 2
SWH 8012.820 Hz
FIDRES 0.122266 Hz
AQ 4.0894465 sec
RG 82.92
DW 62.400 usec
DE 6.50 usec
TE 291.8 K
D1 1.00000000 sec
TD0 1

======== CHANNEL f1 ========
SFO1 400.1324710 MHz
NUC1 1H
P1 14.50 usec
PLW1 11.99499989 W

F2 - Processing parameters
SI 65536
SF 400.1300102 MHz
WDW EM
SSB 0
LB 0.30 Hz
GB 0
PC 1.00

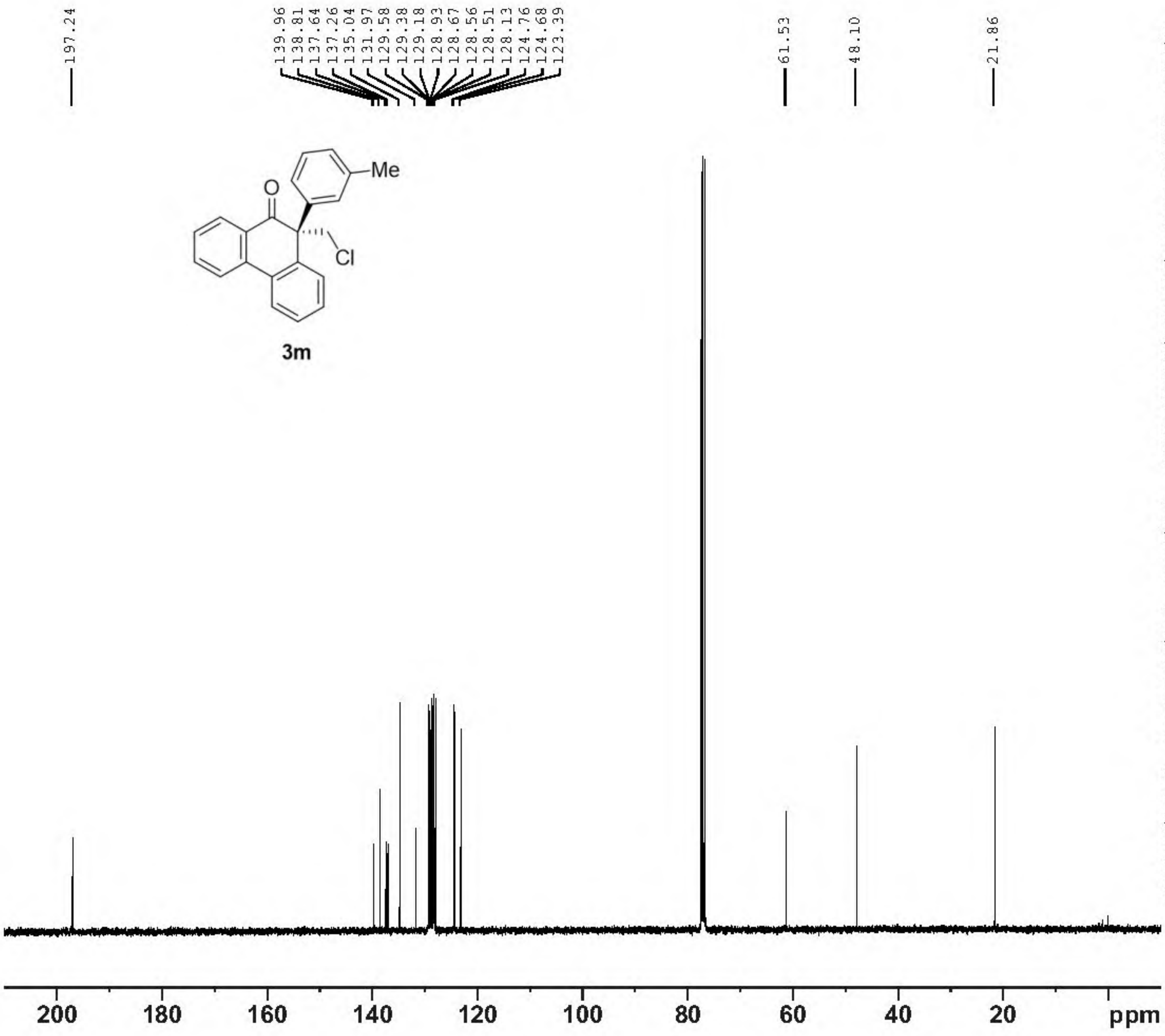

Current Data Parameters
NAME pdt-3-Me-Ph-C
EXPNO 1
PROCNO 1

F2 - Acquisition Parameters
Date_ 20240124
Time 23.58
INSTRUM spect
PROBHD 5 mm PABBO BB/
PULPROG zgpg30
TD 65536
SOLVENT CDCl3
NS 1000
DS 2
SWH 24038.461 Hz
FIDRES 0.366798 Hz
AQ 1.3631488 sec
RG 196.92
DW 20.800 usec
DE 6.50 usec
TE 293.1 K
D1 2.00000000 sec
D11 0.03000000 sec
TD0 1

======== CHANNEL f1 ========
SFO1 100.6228298 MHz
NUC1 13C
P1 9.70 usec
PLW1 46.98899841 W

======== CHANNEL f2 ========
SFO2 400.1316005 MHz
NUC2 1H
CPDPRG[2 waltz16
PCPD2 90.00 usec
PLW2 11.99499989 W
PLW12 0.34213999 W
PLW13 0.27713001 W

F2 - Processing parameters
SI 32768
SF 100.6127691 MHz
WDW EM
SSB 0
LB 1.00 Hz
GB 0
PC 1.40

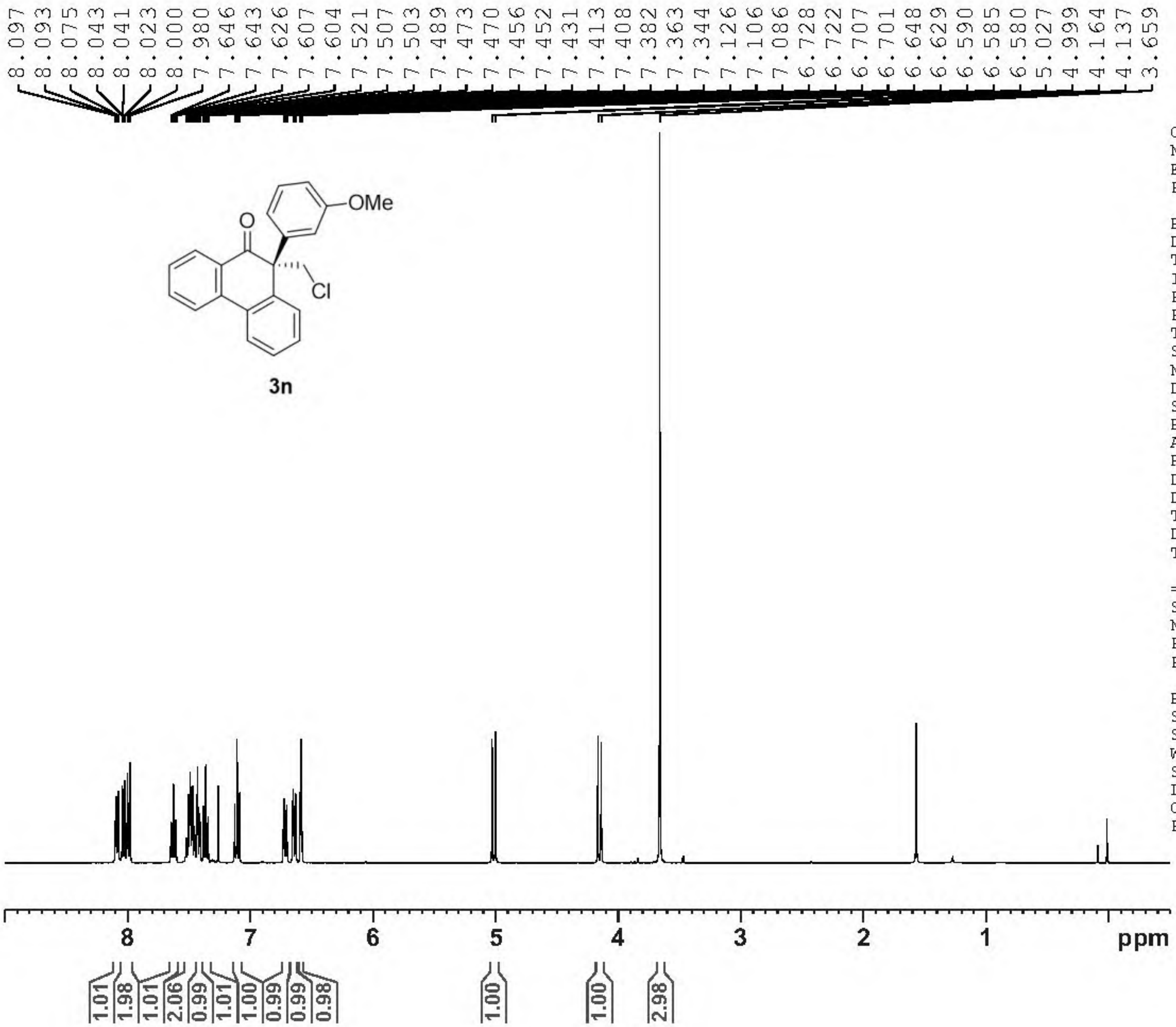


```
Current Data Parameters
NAME      pdt-3-OMe-Ph-H
EXPNO                  1
PROCNO                 1

F2 - Acquisition Parameters
Date_           20240712
Time               20.07
INSTRUM            spect
PROBHD   5 mm PABBO BB/
PULPROG             zg30
TD                 65536
SOLVENT            CDCl3
NS                    13
DS                     2
SWH             8012.820 Hz
FIDRES          0.122266 Hz
AQ             4.0894465 sec
RG                164.33
DW                62.400 usec
DE                  6.50 usec
TE                 303.7 K
D1            1.00000000 sec
TD0                    1

======== CHANNEL f1 ========
SFO1         400.1324710 MHz
NUC1                  1H
P1                 14.50 usec
PLW1         11.99499989 W

F2 - Processing parameters
SI                 65536
SF           400.1300093 MHz
WDW                   EM
SSB                    0
LB                  0.30 Hz
GB                     0
PC                  1.00
```

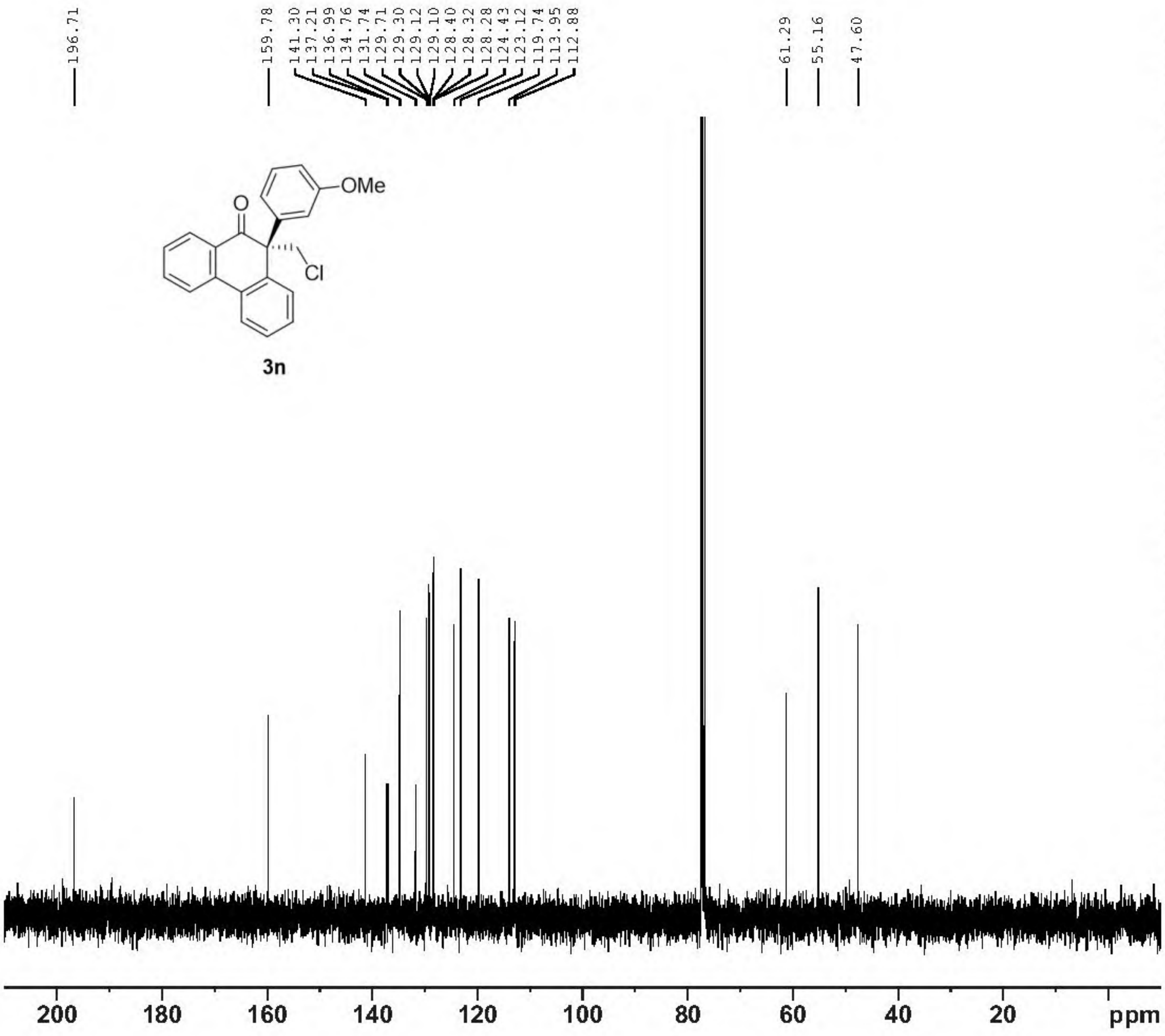

Current Data Parameters
NAME pdt-3-OMe-Ph-C
EXPNO 1
PROCNO 1

F2 - Acquisition Parameters
Date_ 20240712
Time_ 20.10
INSTRUM spect
PROBHD 5 mm PABBO BB/
PULPROG zgpg30
TD 65536
SOLVENT CDCl3
NS 101
DS 2
SWH 24038.461 Hz
FIDRES 0.366798 Hz
AQ 1.3631488 sec
RG 196.92
DW 20.800 usec
DE 6.50 usec
TE 303.4 K
D1 2.00000000 sec
D11 0.03000000 sec
TD0 1

======== CHANNEL f1 ========
SFO1 100.6228298 MHz
NUC1 13C
P1 9.70 usec
PLW1 46.98899841 W

======== CHANNEL f2 ========
SFO2 400.1316005 MHz
NUC2 1H
CPDPRG[2 waltz16
PCPD2 90.00 usec
PLW2 11.99499989 W
PLW12 0.34213999 W
PLW13 0.27713001 W

F2 - Processing parameters
SI 32768
SF 100.6127658 MHz
WDW EM
SSB 0
LB 1.00 Hz
GB 0
PC 1.40

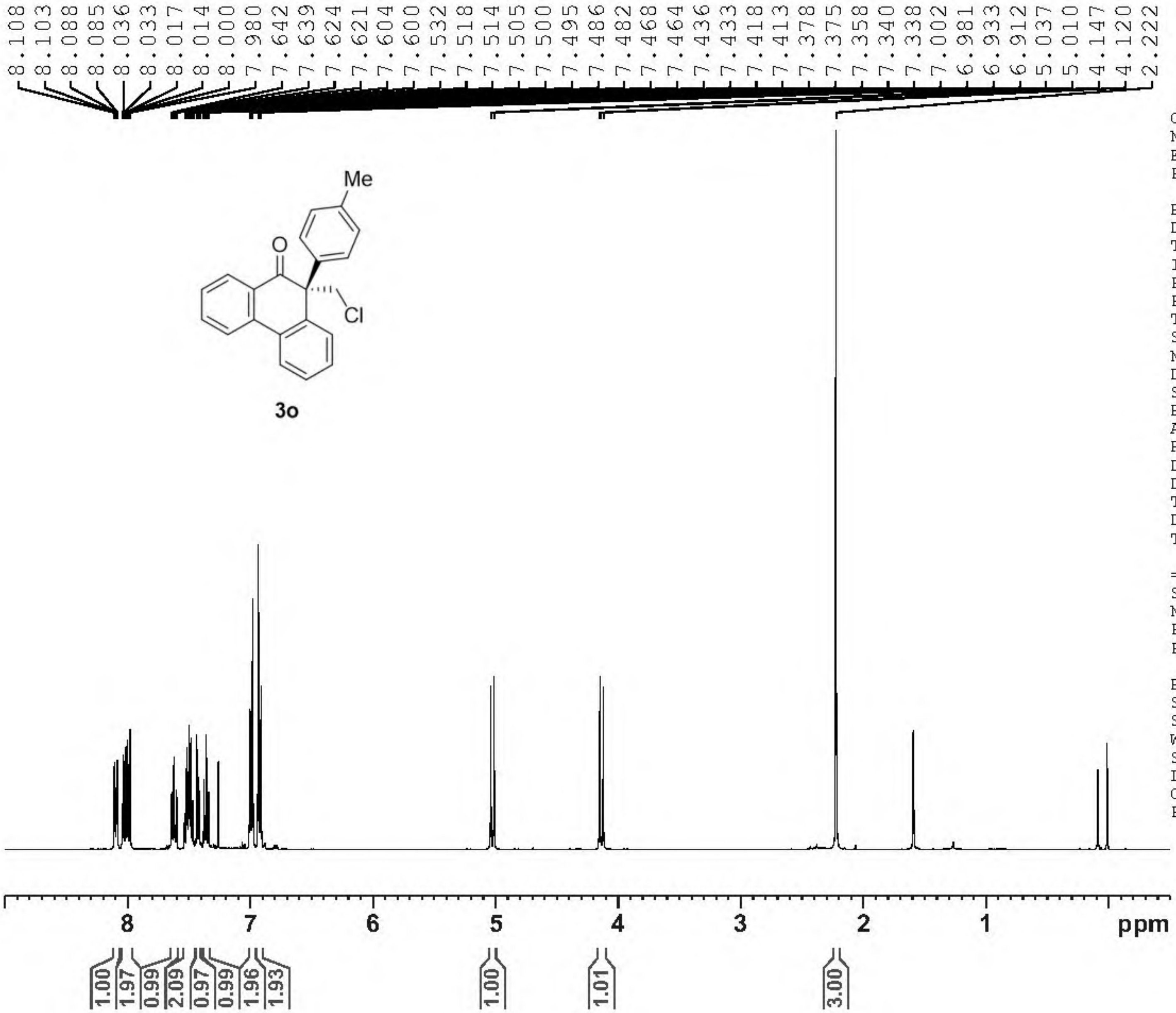


```
Current Data Parameters
NAME       pdt-4-Me-Ph-H
EXPNO                  1
PROCNO                 1

F2 - Acquisition Parameters
Date_           20240120
Time               18.00
INSTRUM            spect
PROBHD   5 mm PABBO BB/
PULPROG             zg30
TD                 65536
SOLVENT            CDCl3
NS                    16
DS                     2
SWH             8012.820 Hz
FIDRES          0.122266 Hz
AQ             4.0894465 sec
RG                 82.92
DW                62.400 usec
DE                  6.50 usec
TE                 296.2 K
D1            1.00000000 sec
TD0                    1

======== CHANNEL f1 ========
SFO1         400.1324710 MHz
NUC1                  1H
P1                 14.50 usec
PLW1         11.99499989 W

F2 - Processing parameters
SI                 65536
SF           400.1300097 MHz
WDW                   EM
SSB                    0
LB                  0.30 Hz
GB                     0
PC                  1.00
```

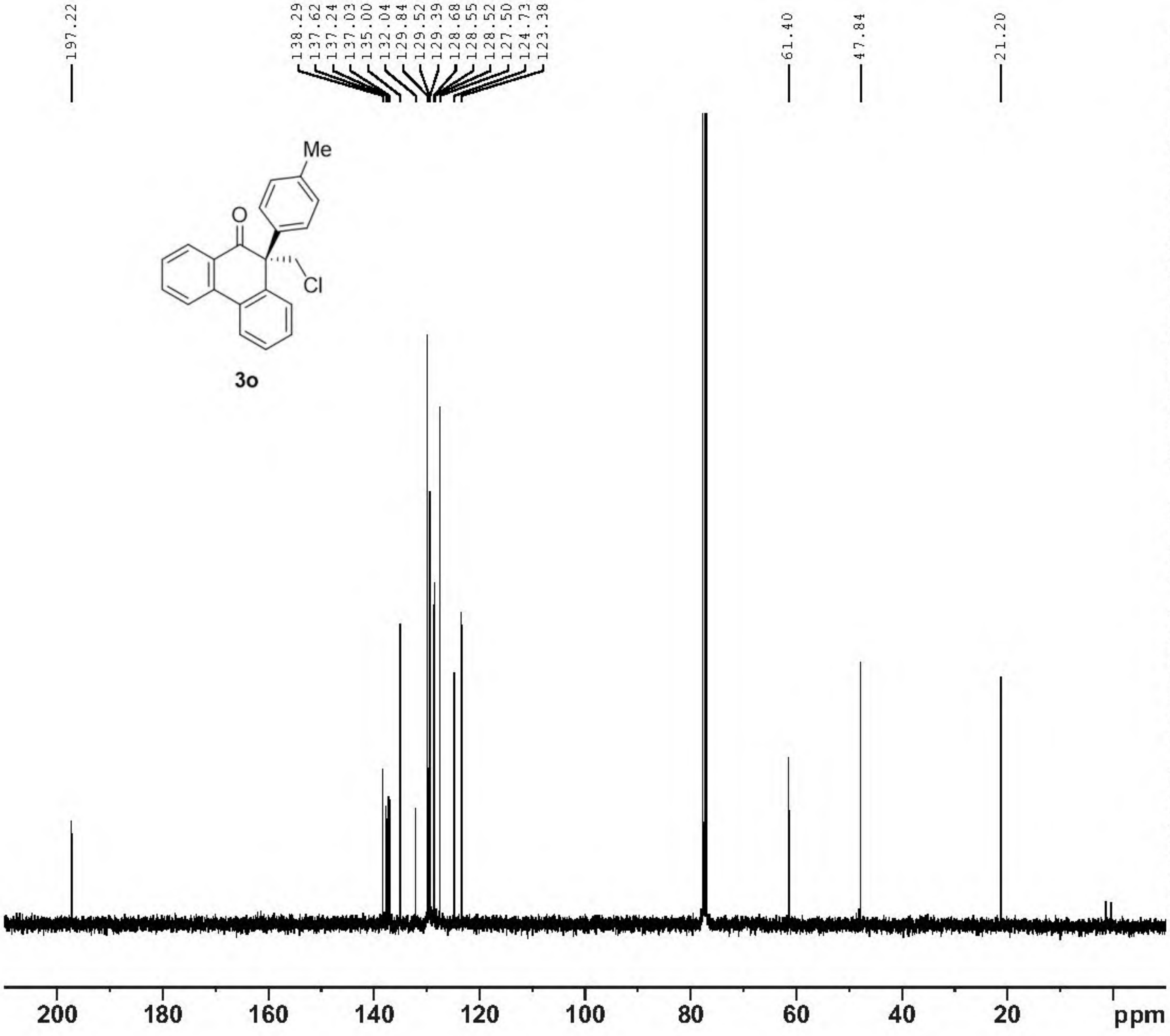


```
Current Data Parameters
NAME        pdt-4-Me-Ph-C
EXPNO                   1
PROCNO                  1

F2 - Acquisition Parameters
Date_            20240120
Time                18.06
INSTRUM             spect
PROBHD   5 mm PABBO BB/
PULPROG           zgpg30
TD                  65536
SOLVENT             CDCl3
NS                    402
DS                      2
SWH            24038.461 Hz
FIDRES          0.366798 Hz
AQ             1.3631488 sec
RG                 196.92
DW                 20.800 usec
DE                   6.50 usec
TE                  297.1 K
D1             2.00000000 sec
D11            0.03000000 sec
TD0                     1

======== CHANNEL f1 ========
SFO1         100.6228298 MHz
NUC1                  13C
P1                   9.70 usec
PLW1          46.98899841 W

======== CHANNEL f2 ========
SFO2         400.1316005 MHz
NUC2                   1H
CPDPRG[2          waltz16
PCPD2               90.00 usec
PLW2          11.99499989 W
PLW12          0.34213999 W
PLW13          0.27713001 W

F2 - Processing parameters
SI                  32768
SF           100.6127375 MHz
WDW                    EM
SSB                     0
LB                   1.00 Hz
GB                      0
PC                   1.40
```

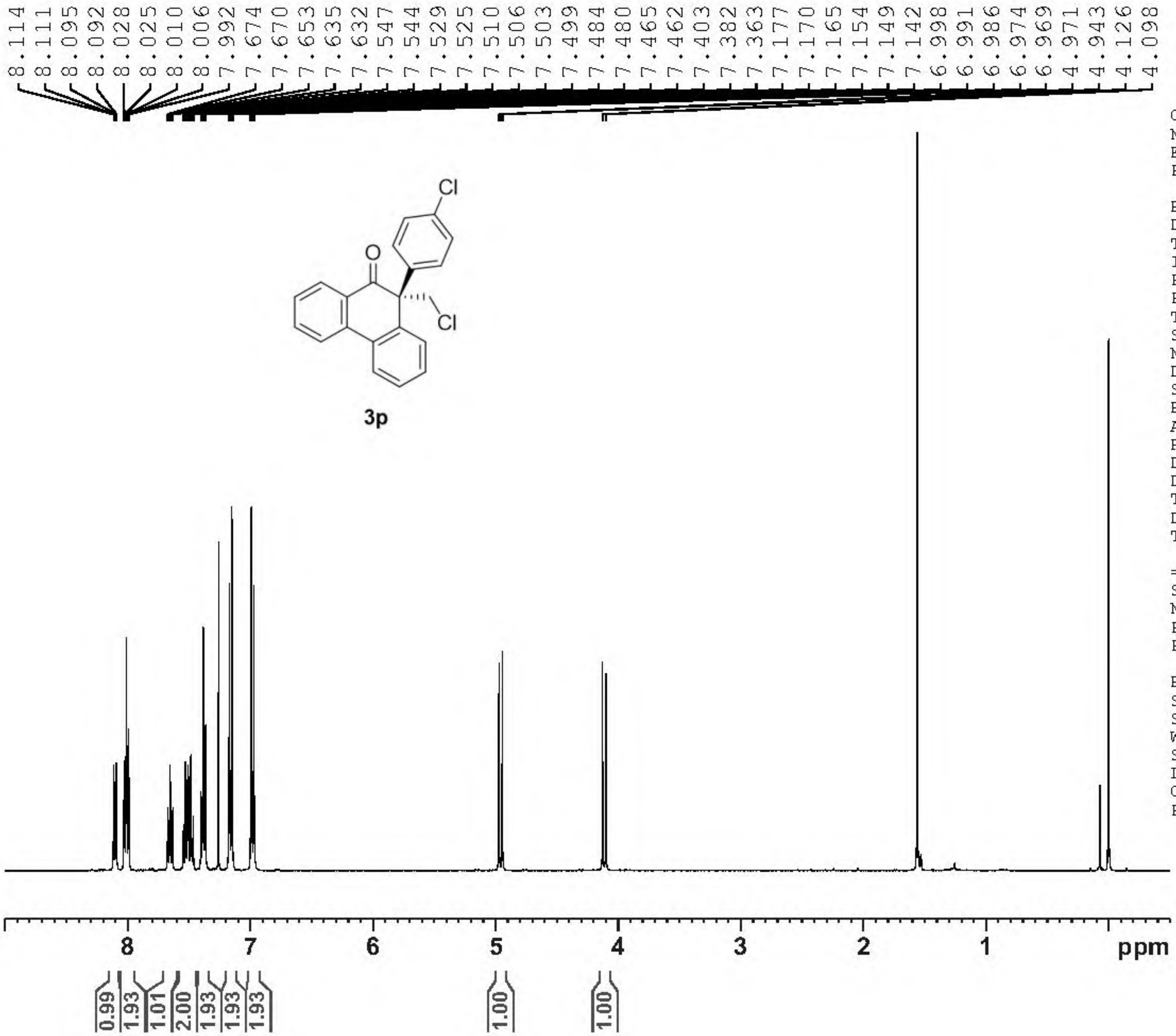


```
Current Data Parameters
NAME        pdt-4-Cl-Ph-H
EXPNO                   1
PROCNO                  1

F2 - Acquisition Parameters
Date_            20240110
Time                23.16
INSTRUM             spect
PROBHD   5 mm PABBO BB/
PULPROG              zg30
TD                  65536
SOLVENT             CDCl3
NS                     16
DS                      2
SWH             8012.820 Hz
FIDRES          0.122266 Hz
AQ             4.0894465 sec
RG                 164.33
DW                 62.400 usec
DE                   6.50 usec
TE                  296.6 K
D1             1.00000000 sec
TD0                     1

======== CHANNEL f1 ========
SFO1        400.1324710 MHz
NUC1                   1H
P1                  14.50 usec
PLW1          11.99499989 W

F2 - Processing parameters
SI                  65536
SF          400.1300103 MHz
WDW                    EM
SSB                     0
LB                   0.30 Hz
GB                      0
PC                   1.00
```

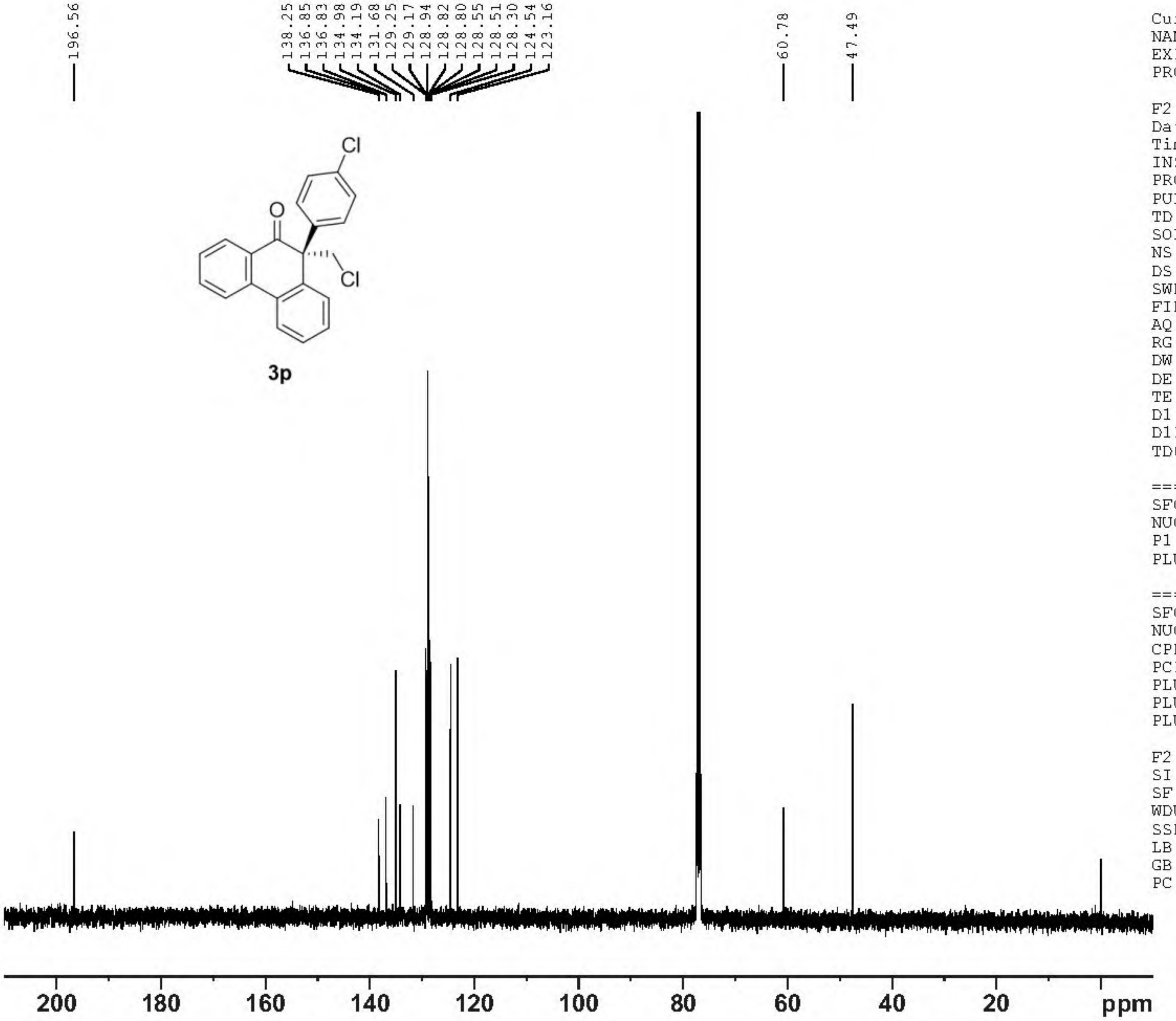

Current Data Parameters
NAME pdt-4-Cl-Ph-C
EXPNO 1
PROCNO 1

F2 - Acquisition Parameters
Date_ 20240111
Time 1.11
INSTRUM spect
PROBHD 5 mm PABBO BB/
PULPROG zgpg30
TD 65536
SOLVENT CDCl3
NS 2000
DS 2
SWH 24038.461 Hz
FIDRES 0.366798 Hz
AQ 1.3631488 sec
RG 196.92
DW 20.800 usec
DE 6.50 usec
TE 297.8 K
D1 2.00000000 sec
D11 0.03000000 sec
TD0 1

======== CHANNEL f1 ========
SFO1 100.6228298 MHz
NUC1 13C
P1 9.70 usec
PLW1 46.98899841 W

======== CHANNEL f2 ========
SFO2 400.1316005 MHz
NUC2 1H
CPDPRG[2 waltz16
PCPD2 90.00 usec
PLW2 11.99499989 W
PLW12 0.34213999 W
PLW13 0.27713001 W

F2 - Processing parameters
SI 32768
SF 100.6127690 MHz
WDW EM
SSB 0
LB 1.00 Hz
GB 0
PC 1.40

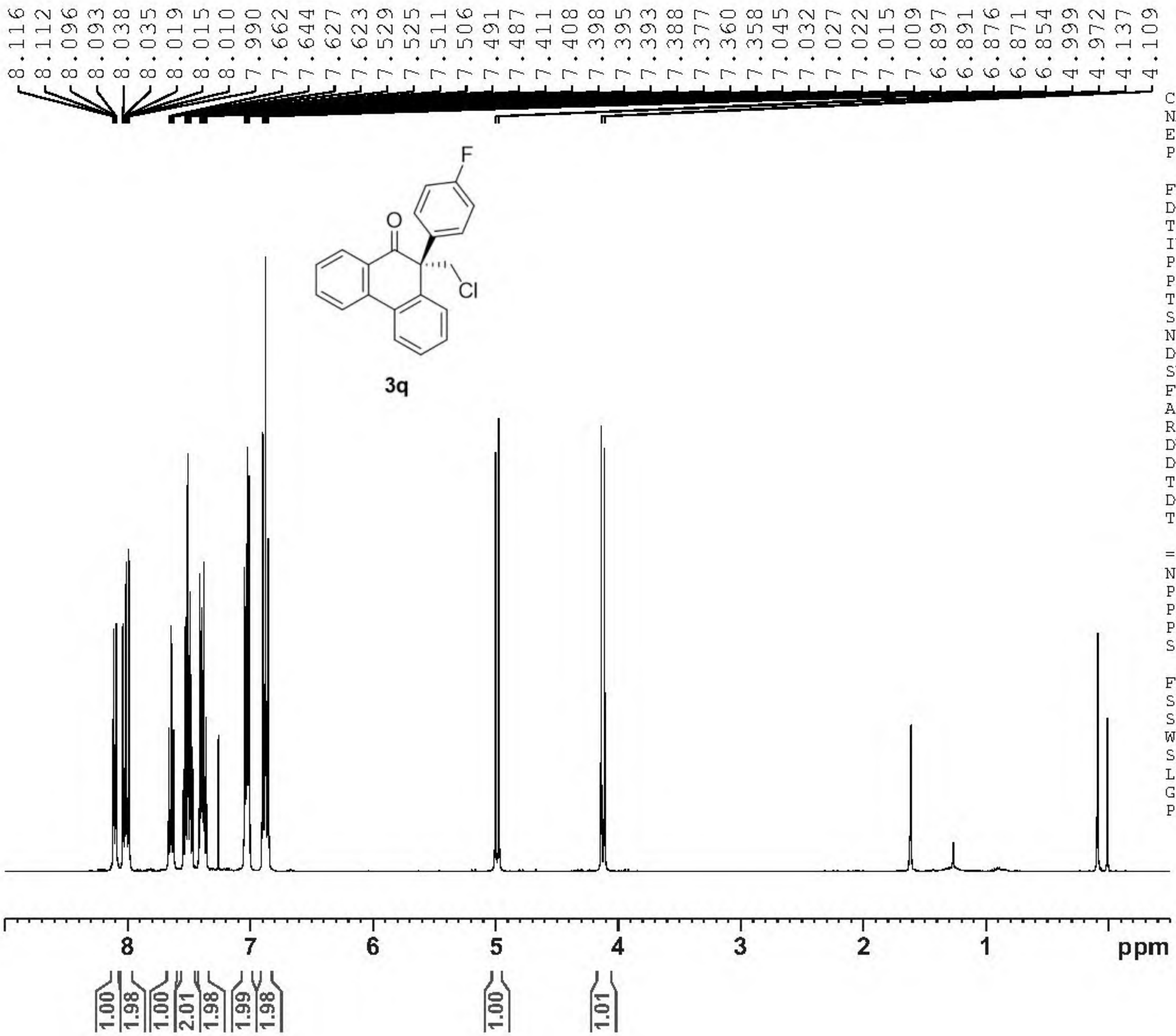

Current Data Parameters
NAME pdt-4-F-Ph-H
EXPNO 1
PROCNO 1

F2 - Acquisition Parameters
Date_ 20240130
Time_ 21.07
INSTRUM spect
PROBHD 5 mm DUL 13C-1
PULPROG zg30
TD 65536
SOLVENT CDCl3
NS 16
DS 2
SWH 8223.685 Hz
FIDRES 0.125483 Hz
AQ 3.9845889 sec
RG 256
DW 60.800 usec
DE 6.00 usec
TE 292.5 K
D1 1.00000000 sec
TD0 1

======== CHANNEL f1 ========
NUC1 1H
P1 15.80 usec
PL1 -1.00 dB
PL1W 12.17476940 W
SFO1 400.1324710 MHz

F2 - Processing parameters
SI 32768
SF 400.1300099 MHz
WDW EM
SSB 0
LB 0.30 Hz
GB 0
PC 1.00

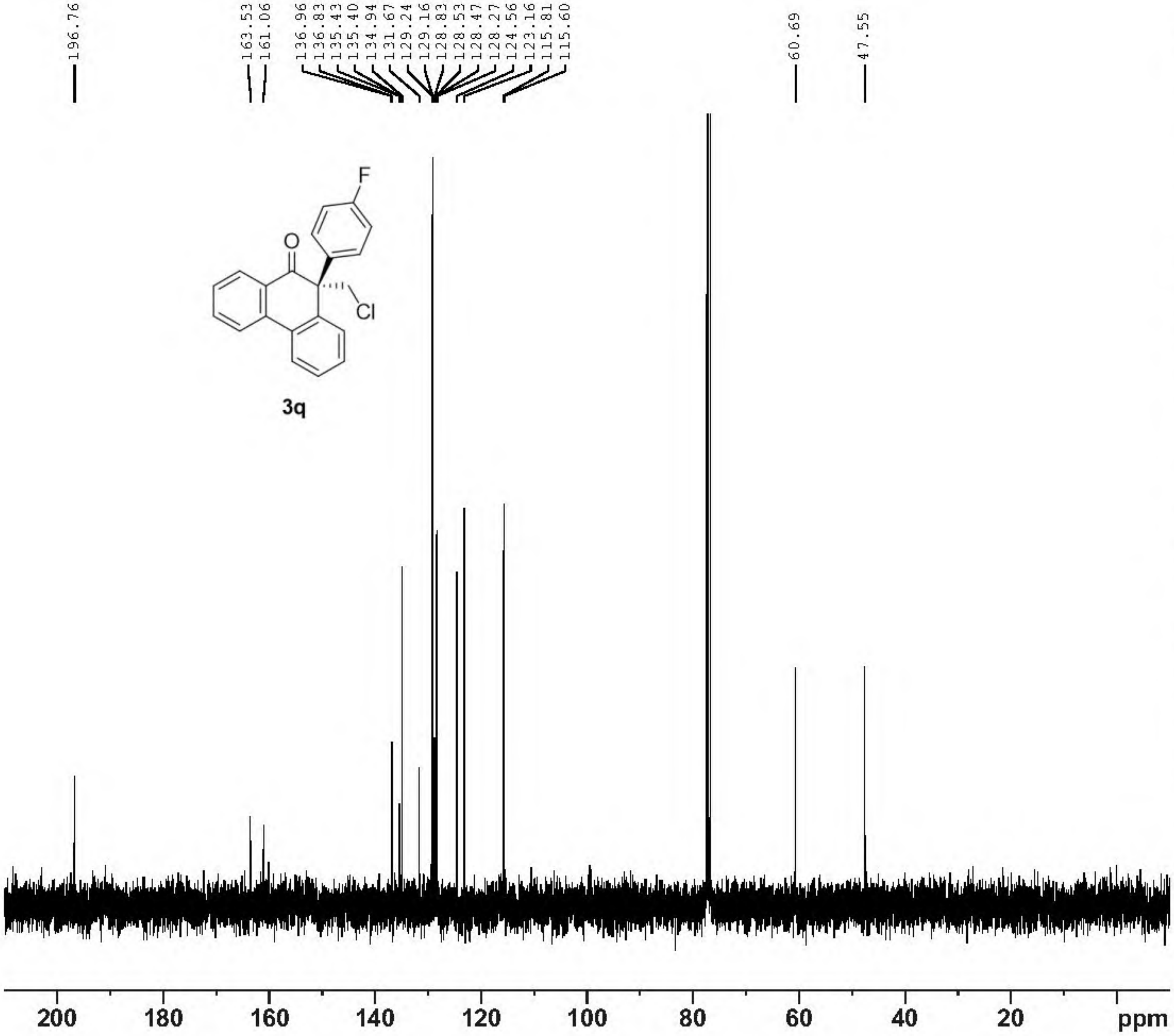

Current Data Parameters
NAME pdt-4-F-Ph-C
EXPNO 1
PROCNO 1

F2 - Acquisition Parameters
Date_ 20240130
Time 20.56
INSTRUM spect
PROBHD 5 mm DUL 13C-1
PULPROG zgpg30
TD 65536
SOLVENT CDCl3
NS 207
DS 1
SWH 24038.461 Hz
FIDRES 0.366798 Hz
AQ 1.3631488 sec
RG 80.6
DW 20.800 usec
DE 6.00 usec
TE 292.7 K
D1 2.00000000 sec
D11 0.03000000 sec
TD0 1

======== CHANNEL f1 ========
NUC1 13C
P1 40.00 usec
PL1 -3.00 dB
PL1W 60.64365387 W
SFO1 100.6228298 MHz

======== CHANNEL f2 ========
CPDPRG[2 waltz16
NUC2 1H
PCPD2 80.00 usec
PL2 -1.00 dB
PL12 14.39 dB
PL13 18.00 dB
PL2W 12.17476940 W
PL12W 0.35193357 W
PL13W 0.15327126 W
SFO2 400.1316005 MHz

F2 - Processing parameters
SI 32768
SF 100.6127699 MHz
WDW EM
SSB 0
LB 1.00 Hz
GB 0
PC 1.40

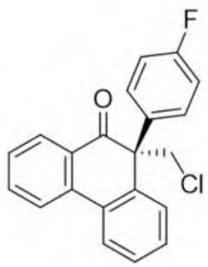

3q

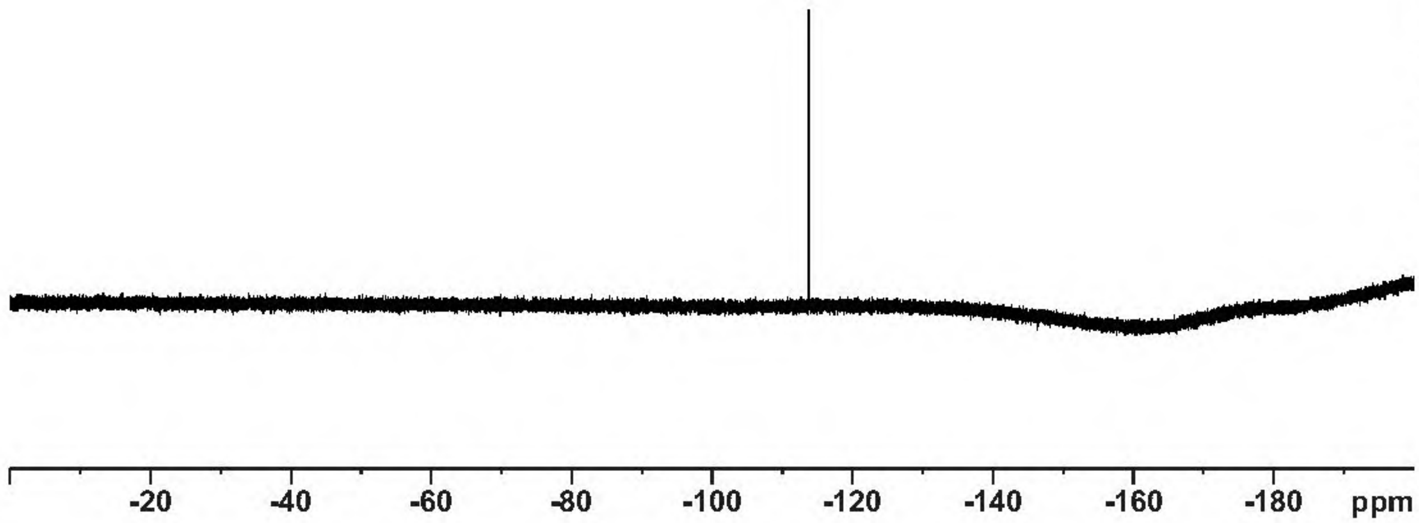


```
Current Data Parameters
NAME      zzj-pdt-p-F-Ph-F
EXPNO                    1
PROCNO                   1

F2 - Acquisition Parameters
Date_             20241011
Time                 20.12
INSTRUM              spect
PROBHD   5 mm PABBO BB/
PULPROG             zgflqn
TD                  131072
SOLVENT              CDCl3
NS                      16
DS                       4
SWH             89285.711 Hz
FIDRES           0.681196 Hz
AQ              0.7340032 sec
RG                  196.92
DW                   5.600 usec
DE                    6.50 usec
TE                   296.1 K
D1             1.00000000 sec
TD0                      1

======== CHANNEL f1 ========
SFO1         376.4607164 MHz
NUC1                   19F
P1                   14.70 usec
PLW1          15.99600029 W

F2 - Processing parameters
SI                   65536
SF           376.4983660 MHz
WDW                     EM
SSB                      0
LB                    0.30 Hz
GB                       0
PC                    1.00
```

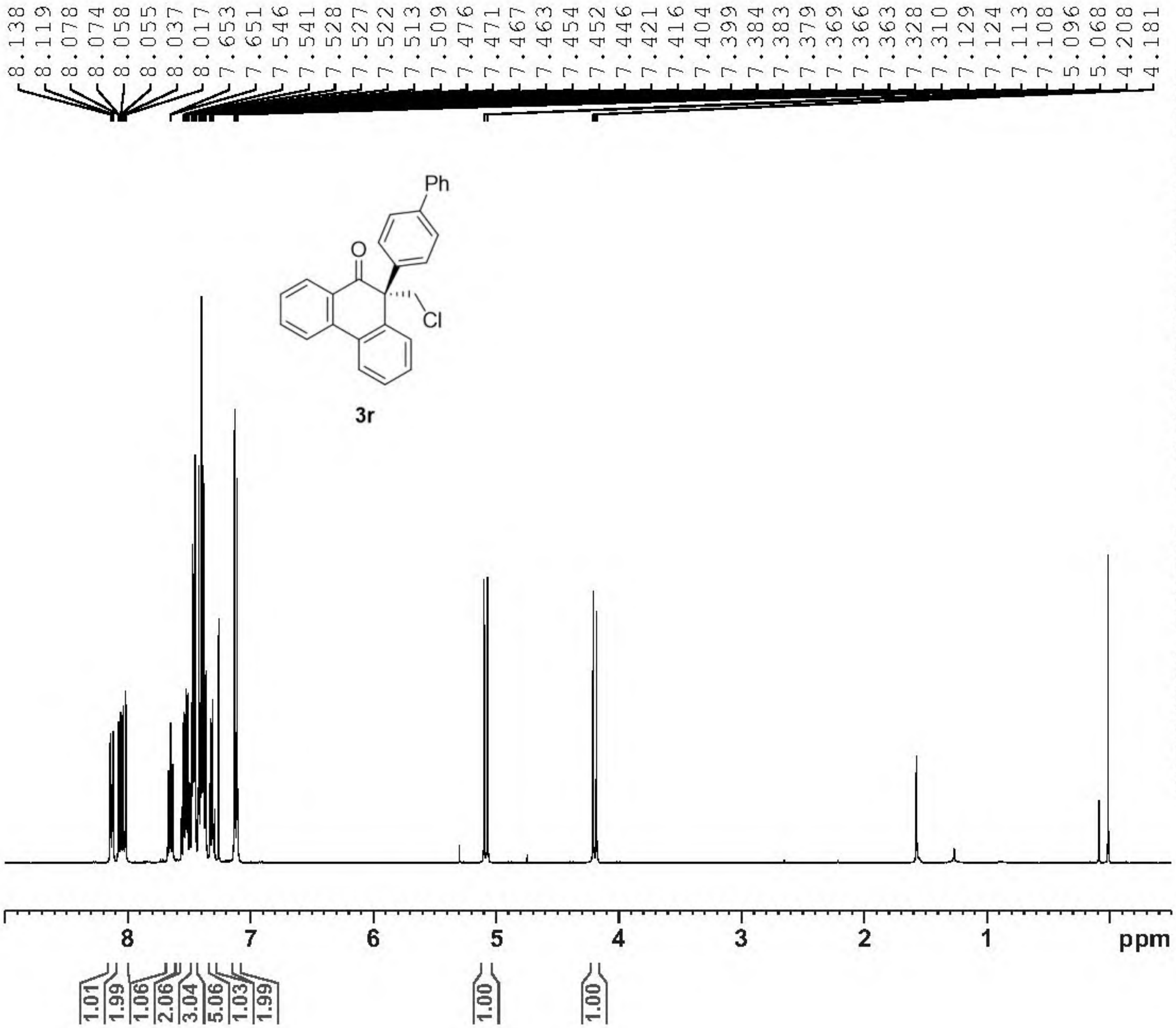

Current Data Parameters
NAME pdt-4-Ph-Ph-H
EXPNO 1
PROCNO 1

F2 - Acquisition Parameters
Date_ 20240308
Time 11.02
INSTRUM spect
PROBHD 5 mm DUL 13C-1
PULPROG zg30
TD 65536
SOLVENT CDCl3
NS 4
DS 2
SWH 8223.685 Hz
FIDRES 0.125483 Hz
AQ 3.9845889 sec
RG 406
DW 60.800 usec
DE 6.00 usec
TE 292.9 K
D1 1.00000000 sec
TD0 1

======== CHANNEL f1 ========
NUC1 1H
P1 15.80 usec
PL1 -1.00 dB
PL1W 12.17476940 W
SFO1 400.1324710 MHz

F2 - Processing parameters
SI 32768
SF 400.1300097 MHz
WDW EM
SSB 0
LB 0.30 Hz
GB 0
PC 1.00

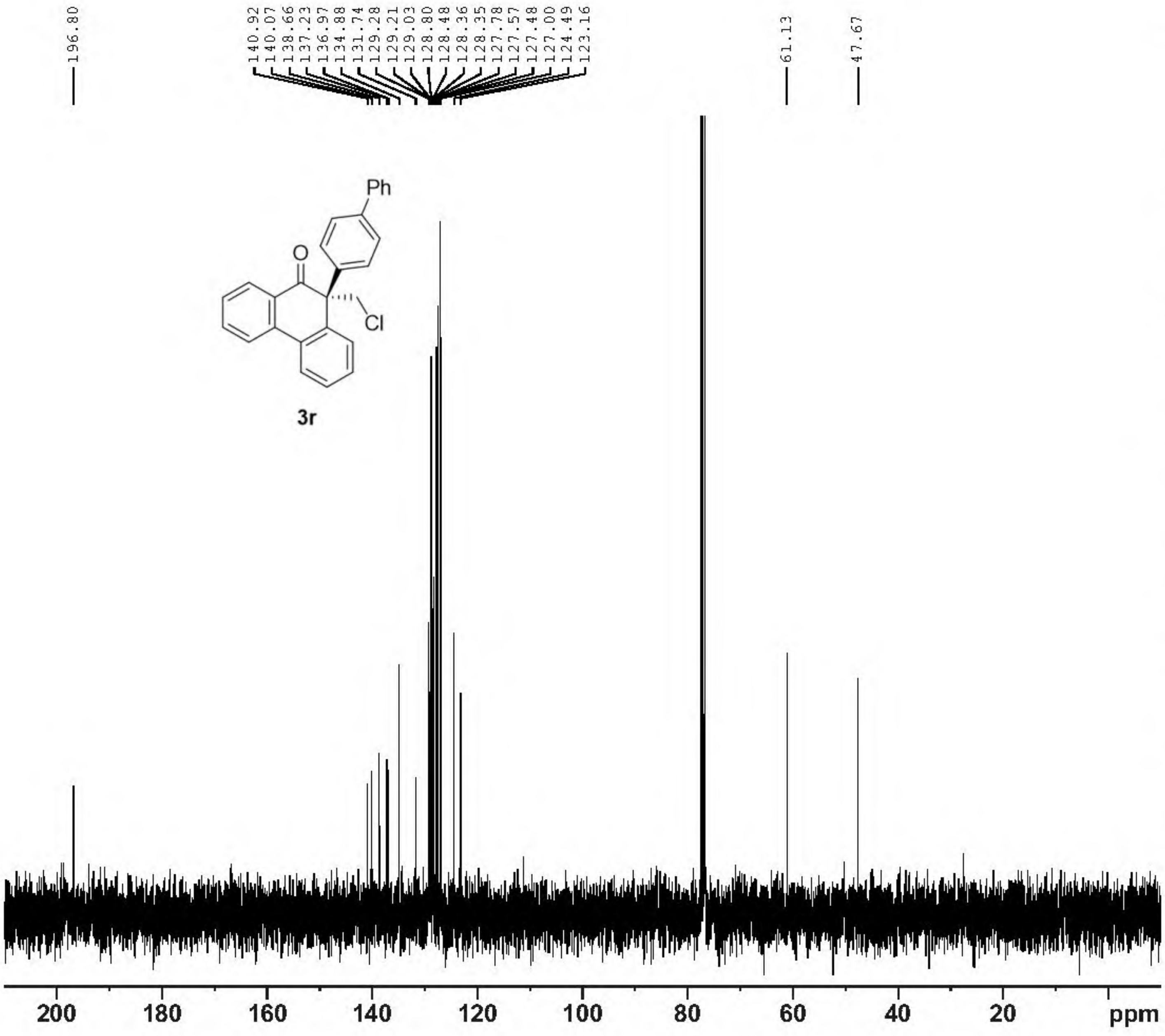


```
Current Data Parameters
NAME       pdt-4-Ph-Ph-C
EXPNO                  1
PROCNO                 1

F2 - Acquisition Parameters
Date_           20240308
Time               11.27
INSTRUM            spect
PROBHD   5 mm DUL 13C-1
PULPROG          zgpg30
TD                 65536
SOLVENT            CDCl3
NS                   400
DS                     1
SWH            24038.461 Hz
FIDRES          0.366798 Hz
AQ             1.3631488 sec
RG                  71.8
DW                20.800 usec
DE                  6.00 usec
TE                 293.2 K
D1            2.00000000 sec
D11           0.03000000 sec
TD0                    1

======== CHANNEL f1 ========
NUC1                 13C
P1                 40.00 usec
PL1                -3.00 dB
PL1W         60.64365387 W
SFO1         100.6228298 MHz

======== CHANNEL f2 ========
CPDPRG[2         waltz16
NUC2                  1H
PCPD2              80.00 usec
PL2                -1.00 dB
PL12               14.39 dB
PL13               18.00 dB
PL2W         12.17476940 W
PL12W         0.35193357 W
PL13W         0.15327126 W
SFO2         400.1316005 MHz

F2 - Processing parameters
SI                 32768
SF           100.6127685 MHz
WDW                   EM
SSB                    0
LB                  1.00 Hz
GB                     0
PC                  1.40
```

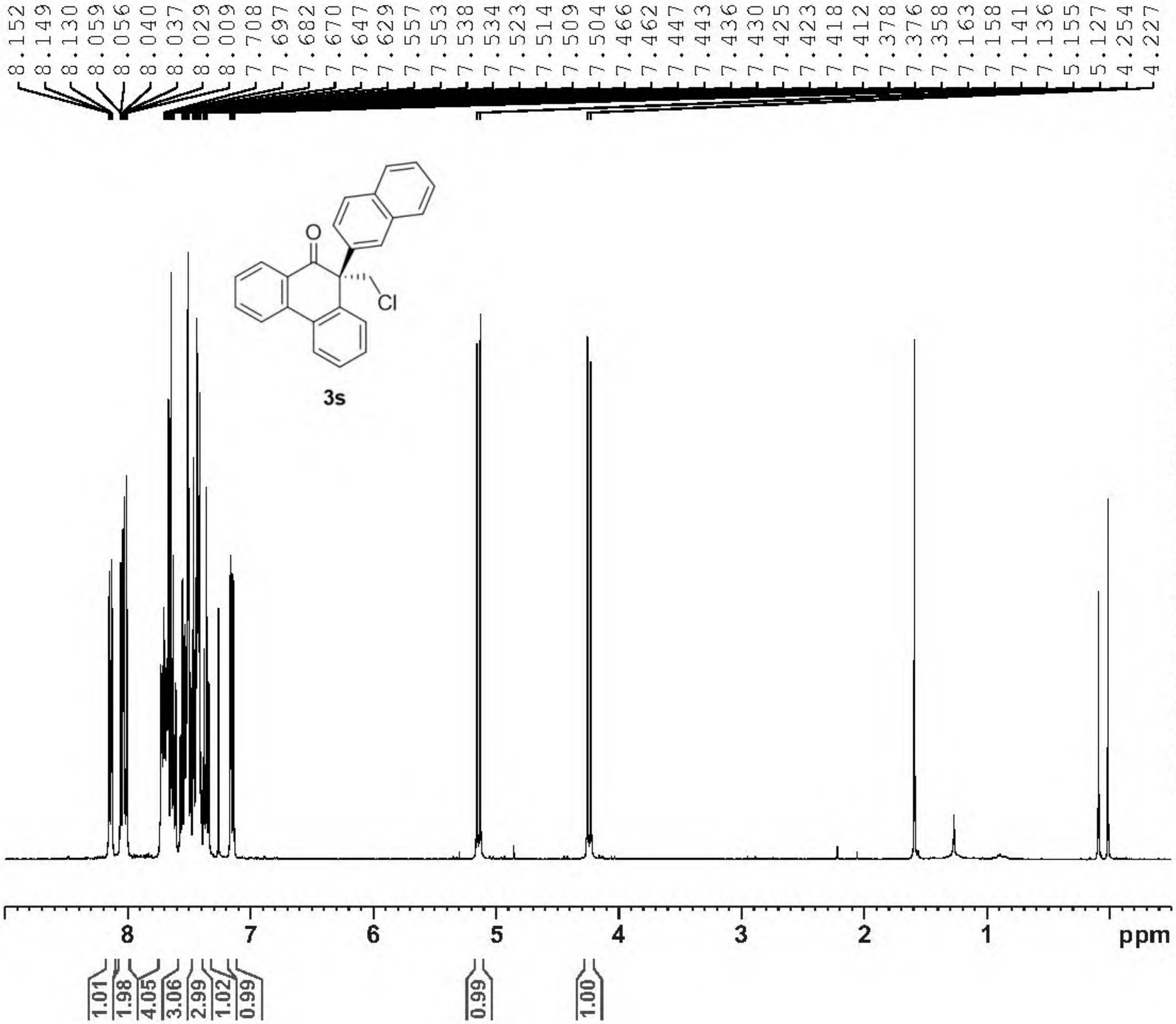

Current Data Parameters
NAME pdt-2-Nap-H
EXPNO 1
PROCNO 1

F2 - Acquisition Parameters
Date_ 20240126
Time_ 0.30
INSTRUM spect
PROBHD 5 mm PABBO BB/
PULPROG zg30
TD 65536
SOLVENT CDCl3
NS 16
DS 2
SWH 8012.820 Hz
FIDRES 0.122266 Hz
AQ 4.0894465 sec
RG 88.84
DW 62.400 usec
DE 6.50 usec
TE 293.4 K
D1 1.00000000 sec
TD0 1

======== CHANNEL f1 ========
SFO1 400.1324710 MHz
NUC1 1H
P1 14.50 usec
PLW1 11.99499989 W

F2 - Processing parameters
SI 65536
SF 400.1300097 MHz
WDW EM
SSB 0
LB 0.30 Hz
GB 0
PC 1.00

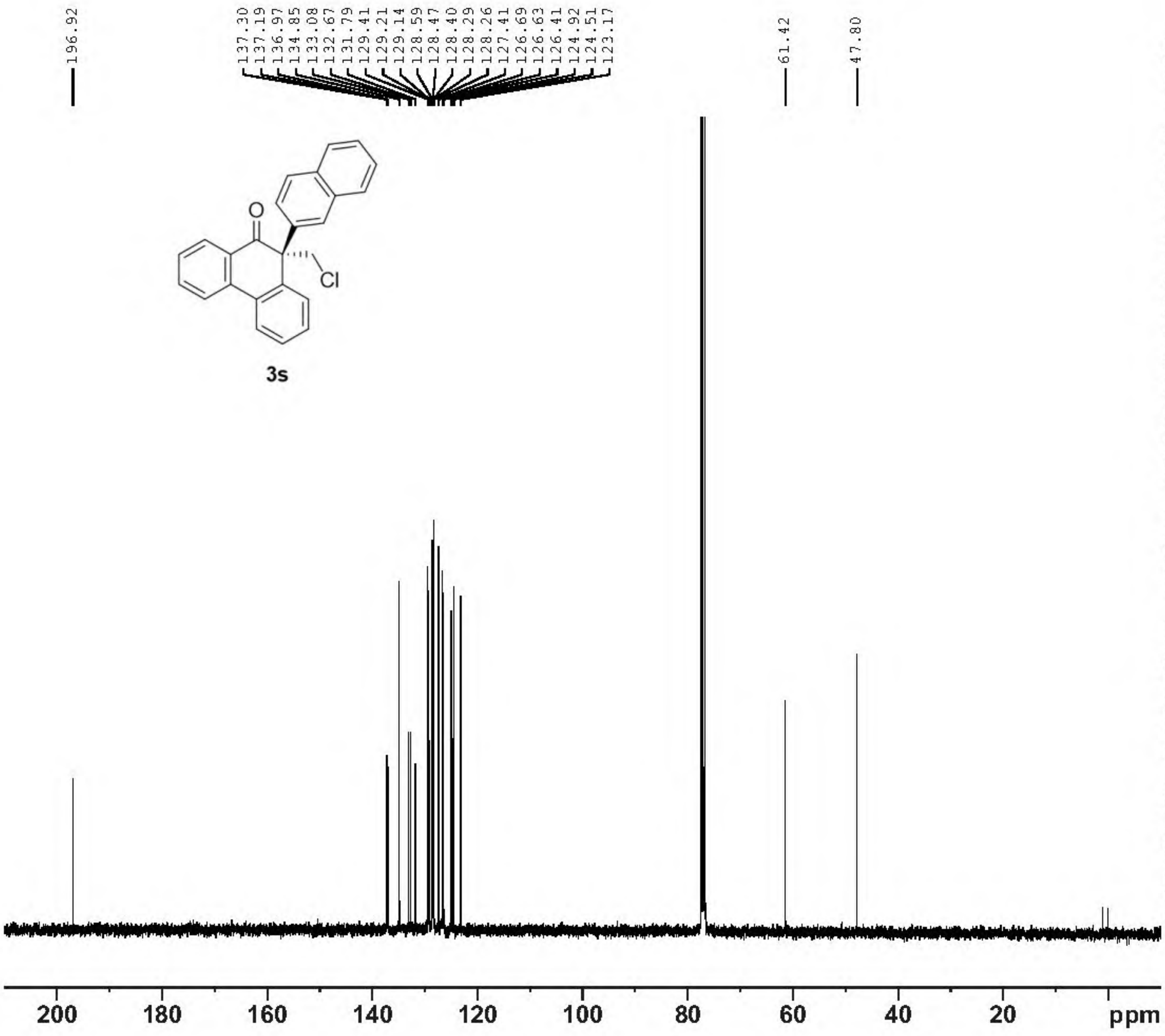

Current Data Parameters
NAME pdt-2-Nap-C
EXPNO 1
PROCNO 1

F2 - Acquisition Parameters
Date_ 20240126
Time 1.28
INSTRUM spect
PROBHD 5 mm PABBO BB/
PULPROG zgpg30
TD 65536
SOLVENT CDCl3
NS 1000
DS 2
SWH 24038.461 Hz
FIDRES 0.366798 Hz
AQ 1.3631488 sec
RG 196.92
DW 20.800 usec
DE 6.50 usec
TE 294.5 K
D1 2.00000000 sec
D11 0.03000000 sec
TD0 1

======== CHANNEL f1 ========
SFO1 100.6228298 MHz
NUC1 13C
P1 9.70 usec
PLW1 46.98899841 W

======== CHANNEL f2 ========
SFO2 400.1316005 MHz
NUC2 1H
CPDPRG[2 waltz16
PCPD2 90.00 usec
PLW2 11.99499989 W
PLW12 0.34213999 W
PLW13 0.27713001 W

F2 - Processing parameters
SI 32768
SF 100.6127688 MHz
WDW EM
SSB 0
LB 1.00 Hz
GB 0
PC 1.40

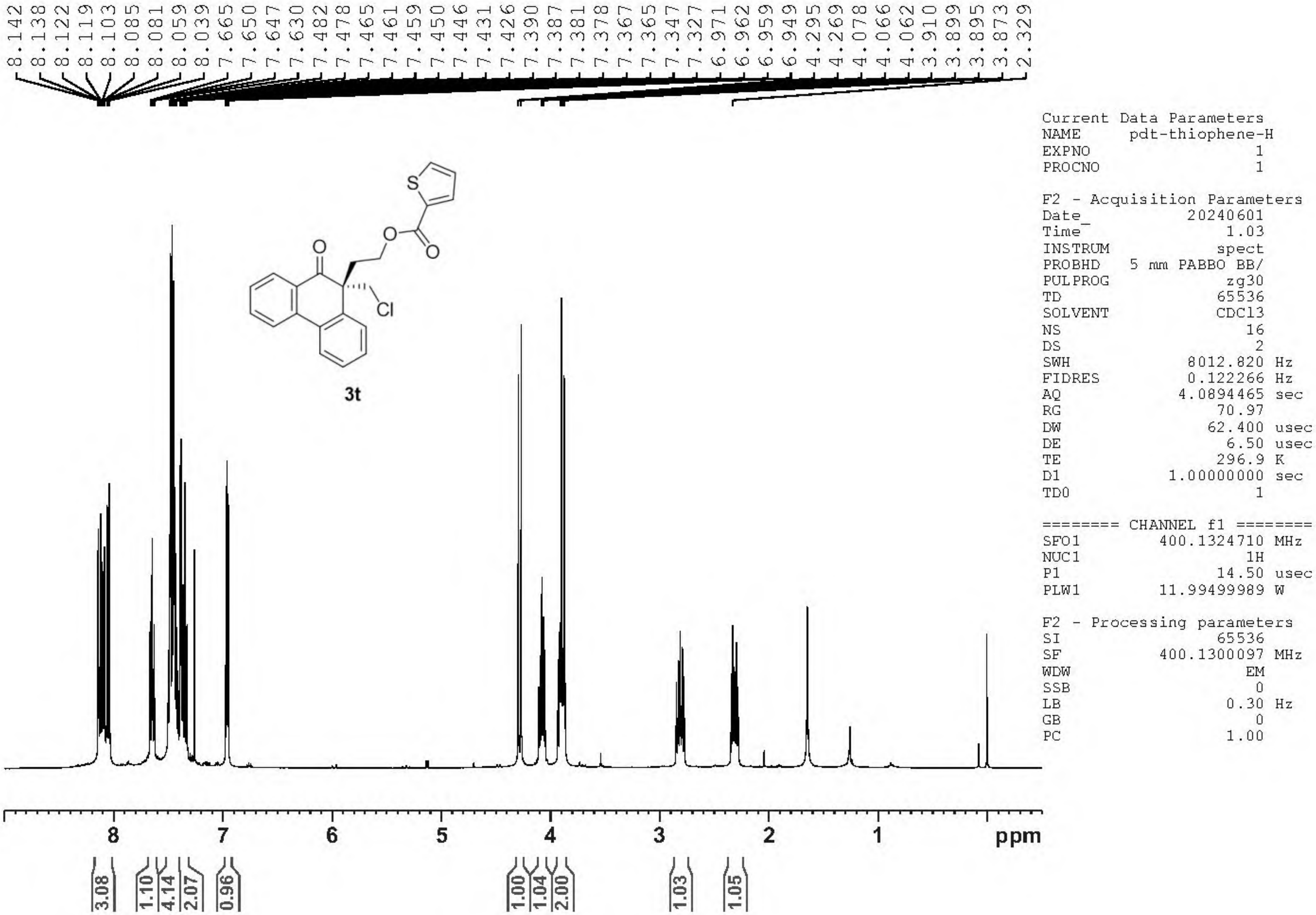
8.142 8.138 8.122 8.119 8.103 8.085 8.081 8.059 8.039 7.665 7.650 7.647 7.630 7.482 7.478 7.465 7.461 7.459 7.450 7.446 7.431 7.426 7.390 7.387 7.381 7.378 7.367 7.365 7.347 7.327 6.971 6.962 6.959 6.949 4.295 4.269 4.078 4.066 4.062 3.910 3.899 3.895 3.873 2.329
S
O
O
O
Cl
3t
Current Data Parameters
NAME pdt-thiophene-H
EXPNO 1
PROCNO 1
F2 - Acquisition Parameters
Date_ 20240601
Time 1.03
INSTRUM spect
PROBHD 5 mm PABBO BB/
PULPROG zg30
TD 65536
SOLVENT CDCl3
NS 16
DS 2
SWH 8012.820 Hz
FIDRES 0.122266 Hz
AQ 4.0894465 sec
RG 70.97
DW 62.400 usec
DE 6.50 usec
TE 296.9 K
D1 1.00000000 sec
TD0 1
======== CHANNEL f1 ========
SFO1 400.1324710 MHz
NUC1 1H
P1 14.50 usec
PLW1 11.99499989 W
F2 - Processing parameters
SI 65536
SF 400.1300097 MHz
WDW EM
SSB 0
LB 0.30 Hz
GB 0
PC 1.00
8 7 6 5 4 3 2 1 ppm
3.08 1.10 4.14 2.07 0.96 1.00 1.04 2.00 1.03 1.05

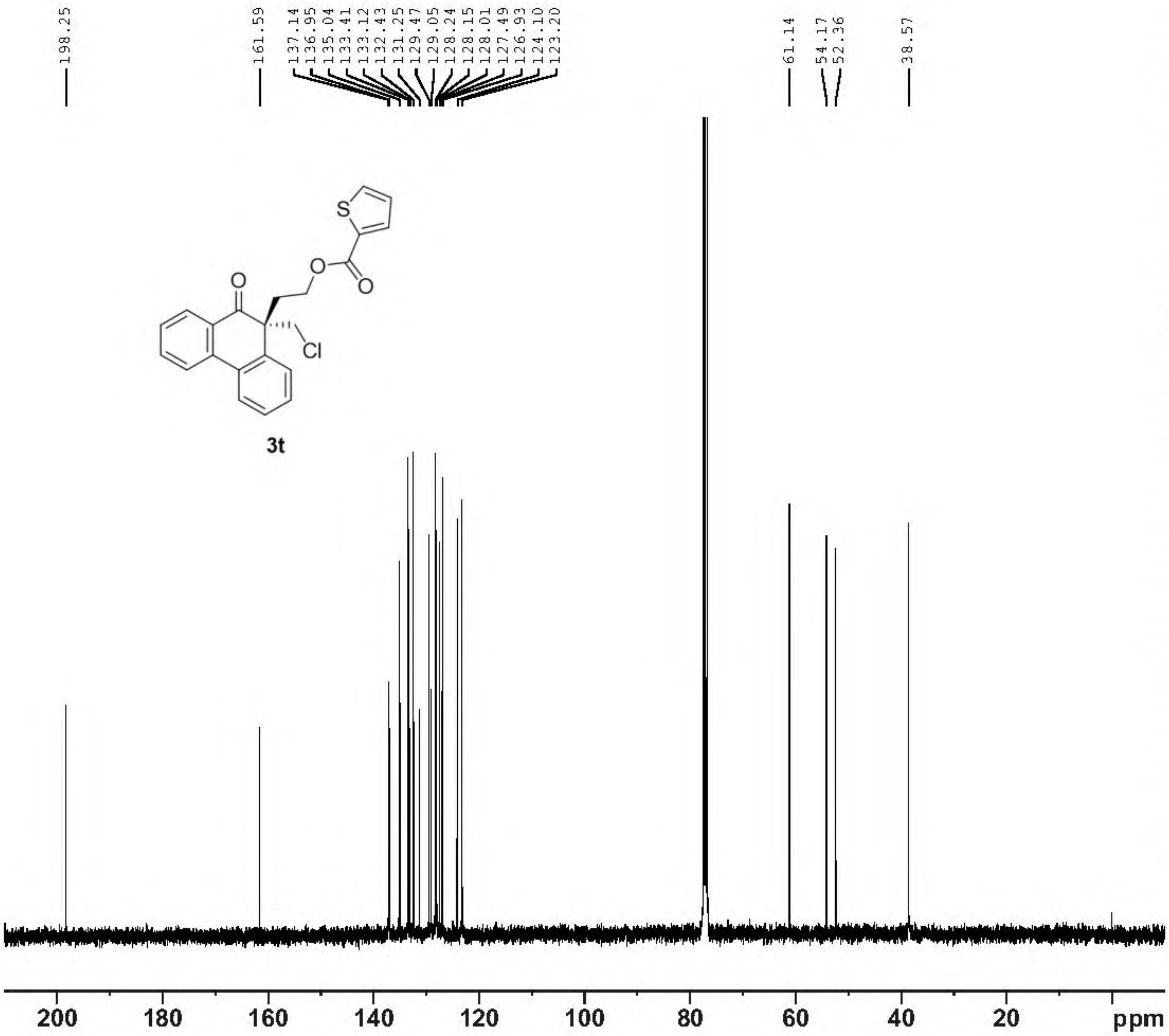


```
Current Data Parameters
NAME     pdt-thiophene-C
EXPNO                  1
PROCNO                 1

F2 - Acquisition Parameters
Date_           20240601
Time                2.01
INSTRUM            spect
PROBHD   5 mm PABBO BB/
PULPROG           zgpg30
TD                 65536
SOLVENT            CDCl3
NS                  1000
DS                     2
SWH            24038.461 Hz
FIDRES          0.366798 Hz
AQ             1.3631488 sec
RG                196.92
DW                20.800 usec
DE                  6.50 usec
TE                 297.6 K
D1            2.00000000 sec
D11           0.03000000 sec
TD0                    1

======== CHANNEL f1 ========
SFO1         100.6228298 MHz
NUC1                 13C
P1                  9.70 usec
PLW1         46.98899841 W

======== CHANNEL f2 ========
SFO2         400.1316005 MHz
NUC2                  1H
CPDPRG[2         waltz16
PCPD2              90.00 usec
PLW2         11.99499989 W
PLW12         0.34213999 W
PLW13         0.27713001 W

F2 - Processing parameters
SI                 32768
SF           100.6127690 MHz
WDW                   EM
SSB                    0
LB                  1.00 Hz
GB                     0
PC                  1.40
```

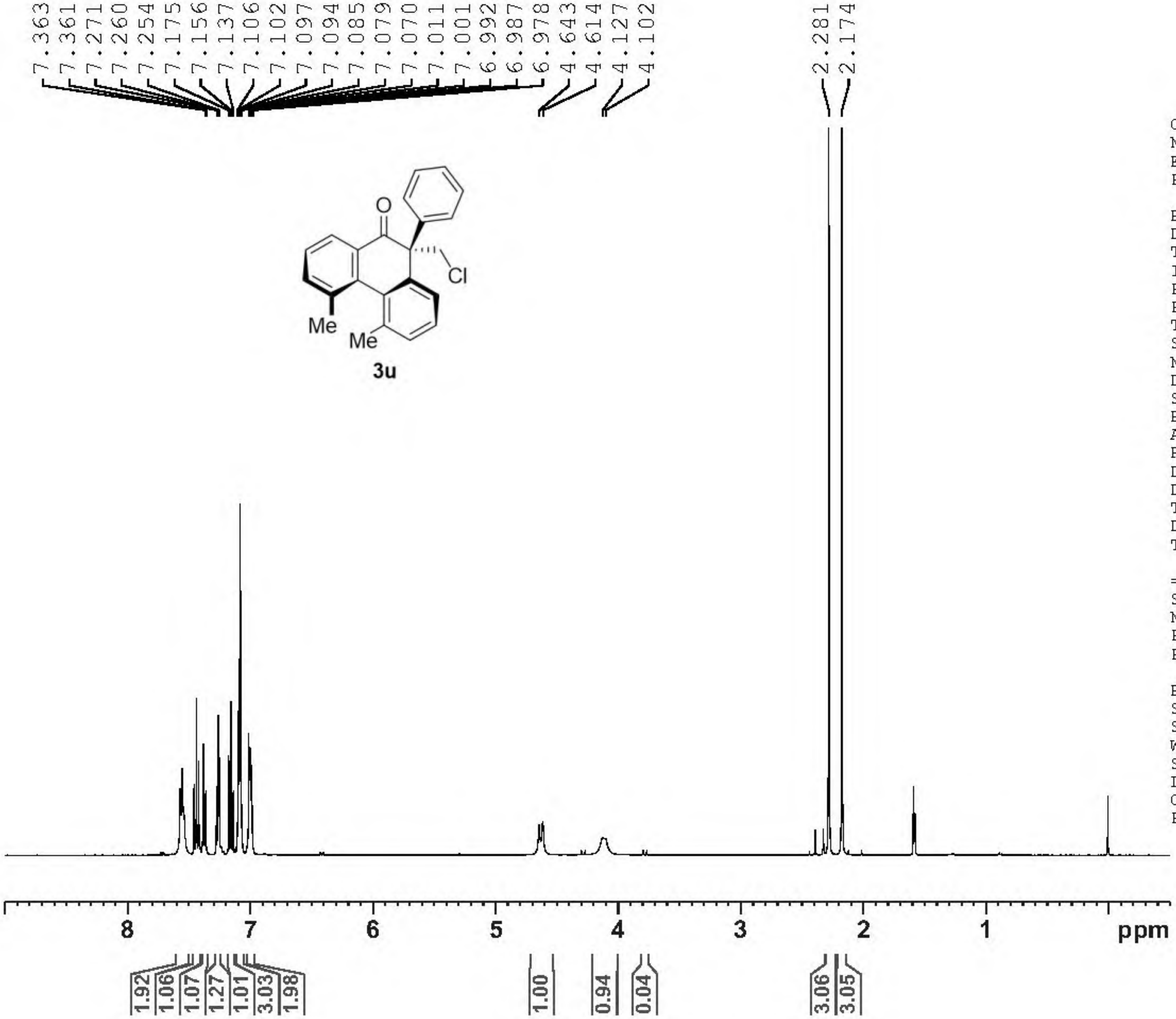

Current Data Parameters
NAME pdt-axial-H
EXPNO 1
PROCNO 1

F2 - Acquisition Parameters
Date_ 20240718
Time_ 10.13
INSTRUM spect
PROBHD 5 mm PABBO BB/
PULPROG zg30
TD 65536
SOLVENT CDCl3
NS 14
DS 2
SWH 8012.820 Hz
FIDRES 0.122266 Hz
AQ 4.0894465 sec
RG 88.84
DW 62.400 usec
DE 6.50 usec
TE 296.0 K
D1 1.00000000 sec
TD0 1

======== CHANNEL f1 ========
SFO1 400.1324710 MHz
NUC1 1H
P1 14.50 usec
PLW1 11.99499989 W

F2 - Processing parameters
SI 65536
SF 400.1300095 MHz
WDW EM
SSB 0
LB 0.30 Hz
GB 0
PC 1.00

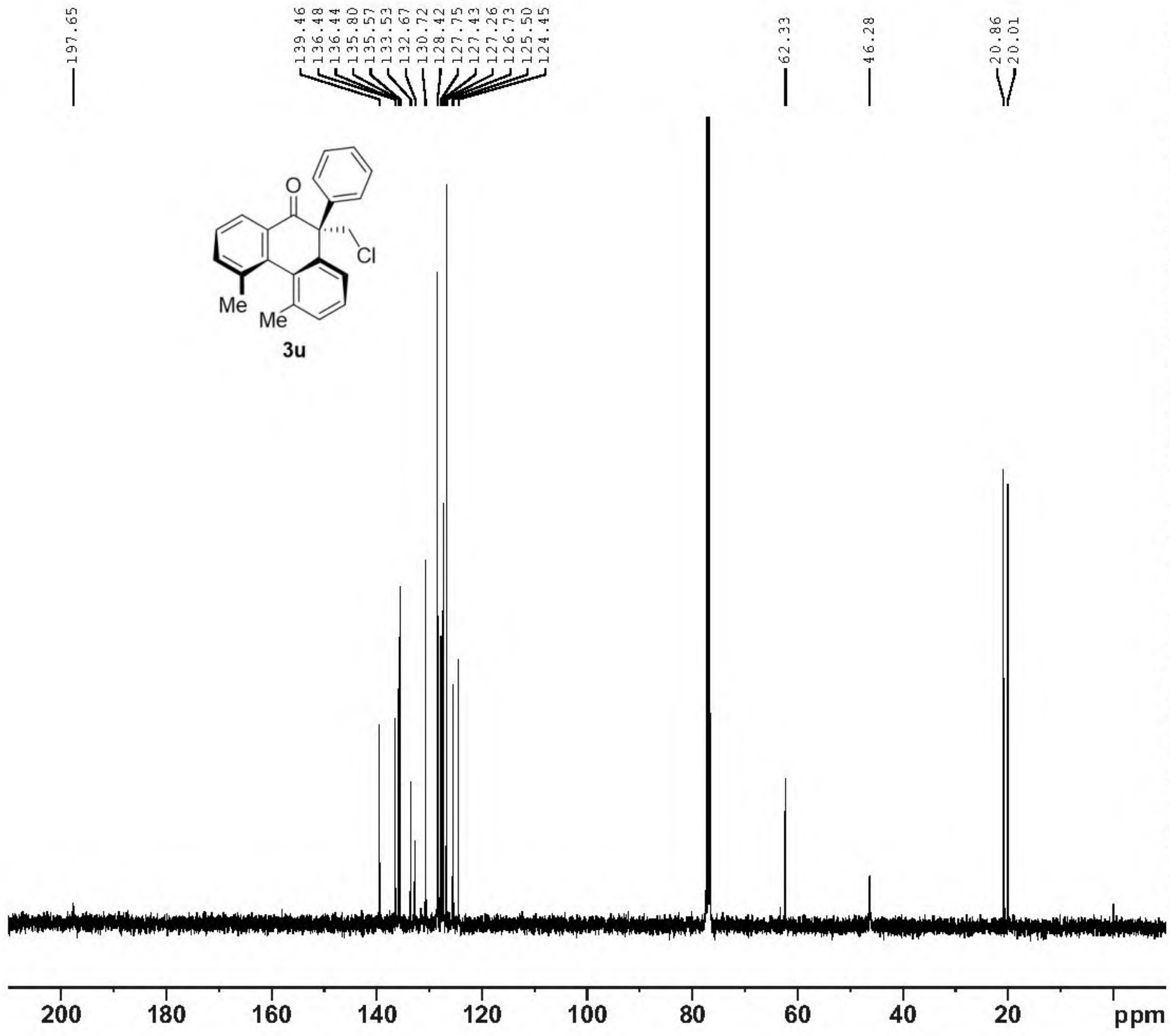

Current Data Parameters
NAME pdt-axial-C
EXPNO 2
PROCNO 1

F2 - Acquisition Parameters
Date_ 20240718
Time_ 11.04
INSTRUM spect
PROBHD 5 mm PABBO BB/
PULPROG zgpg30
TD 65536
SOLVENT CDCl3
NS 682
DS 2
SWH 26041.666 Hz
FIDRES 0.397364 Hz
AQ 1.2582912 sec
RG 196.92
DW 19.200 usec
DE 6.50 usec
TE 297.2 K
D1 2.00000000 sec
D11 0.03000000 sec
TD0 1

======== CHANNEL f1 ========
SFO1 100.6228298 MHz
NUC1 13C
P1 9.70 usec
PLW1 46.98899841 W

======== CHANNEL f2 ========
SFO2 400.1316005 MHz
NUC2 1H
CPDPRG[2 waltz16
PCPD2 90.00 usec
PLW2 11.99499989 W
PLW12 0.34213999 W
PLW13 0.27713001 W

F2 - Processing parameters
SI 32768
SF 100.6127738 MHz
WDW EM
SSB 0
LB 1.00 Hz
GB 0
PC 1.40

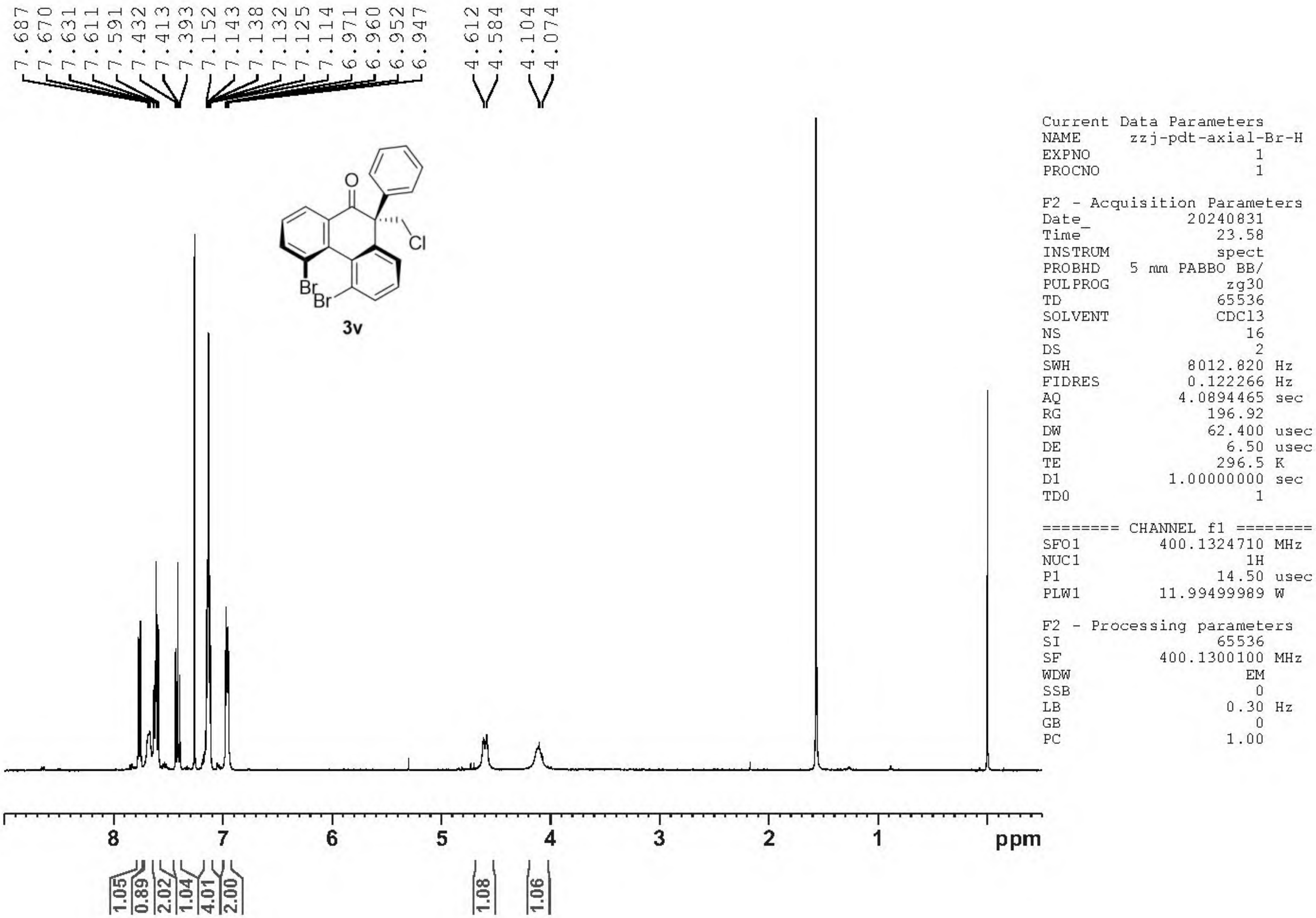
7.687 7.670 7.631 7.611 7.591 7.432 7.413 7.393 7.152 7.143 7.138 7.132 7.125 7.114 6.971 6.960 6.952 6.947
4.612 4.584 4.104 4.074
O
Cl
Br
Br
3v
8 7 6 5 4 3 2 1 ppm
1.05 0.89 2.02 1.04 4.01 2.00 1.08 1.06
Current Data Parameters
NAME zzj-pdt-axial-Br-H
EXPNO 1
PROCNO 1
F2 - Acquisition Parameters
Date_ 20240831
Time_ 23.58
INSTRUM spect
PROBHD 5 mm PABBO BB/
PULPROG zg30
TD 65536
SOLVENT CDCl3
NS 16
DS 2
SWH 8012.820 Hz
FIDRES 0.122266 Hz
AQ 4.0894465 sec
RG 196.92
DW 62.400 usec
DE 6.50 usec
TE 296.5 K
D1 1.00000000 sec
TD0 1
======== CHANNEL f1 ========
SFO1 400.1324710 MHz
NUC1 1H
P1 14.50 usec
PLW1 11.99499989 W
F2 - Processing parameters
SI 65536
SF 400.1300100 MHz
WDW EM
SSB 0
LB 0.30 Hz
GB 0
PC 1.00

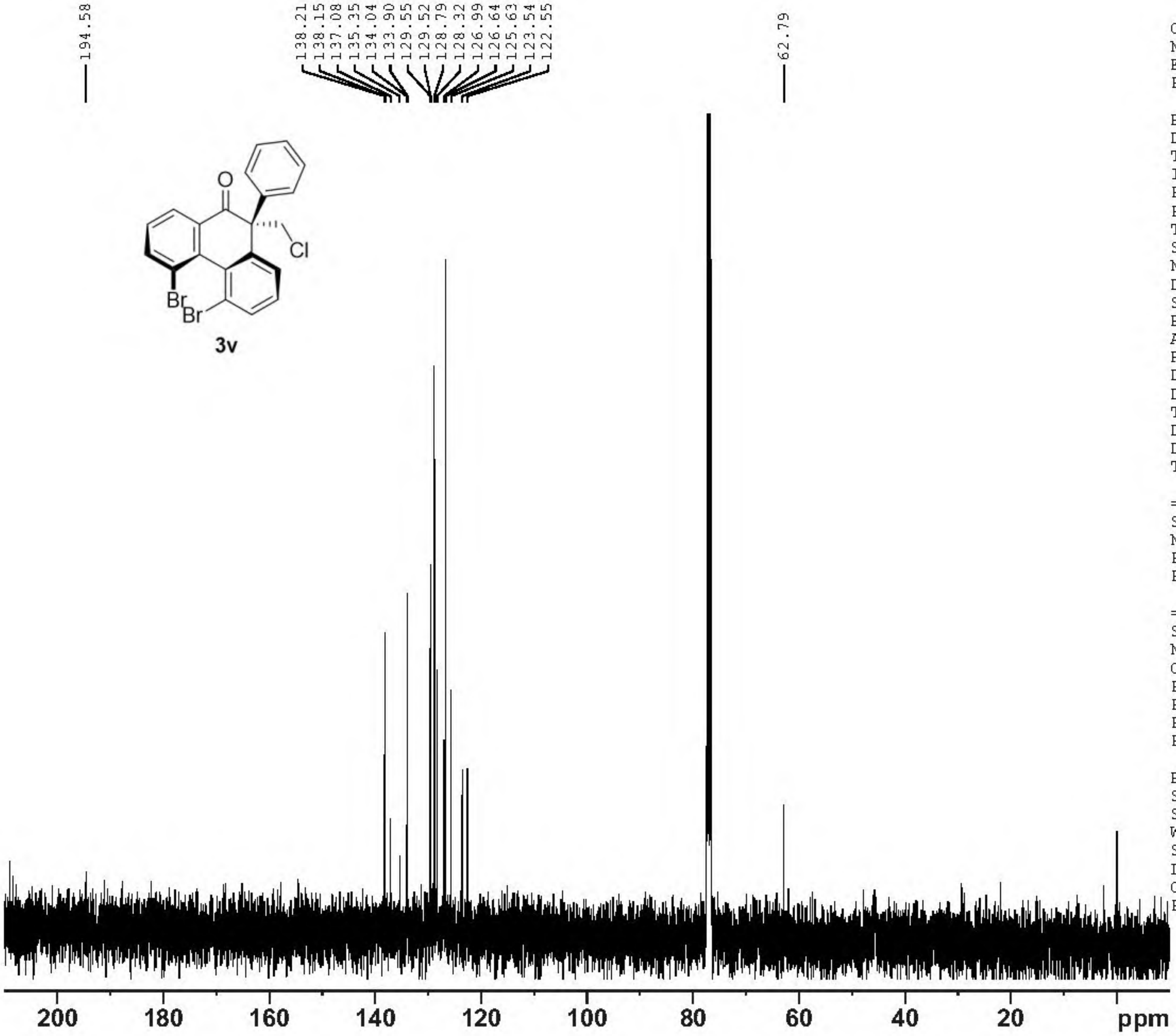


```
Current Data Parameters
NAME      zzj-pdt-axial-Br-C
EXPNO                  1
PROCNO                 1

F2 - Acquisition Parameters
Date_           20240901
Time                0.56
INSTRUM            spect
PROBHD    5 mm PABBO BB/
PULPROG           zgpg30
TD                 65536
SOLVENT            CDCl3
NS                  1000
DS                     2
SWH            24038.461 Hz
FIDRES          0.366798 Hz
AQ             1.3631488 sec
RG                196.92
DW                20.800 usec
DE                  6.50 usec
TE                 297.5 K
D1            2.00000000 sec
D11           0.03000000 sec
TD0                    1

======== CHANNEL f1 ========
SFO1         100.6228298 MHz
NUC1                 13C
P1                  9.70 usec
PLW1         46.98899841 W

======== CHANNEL f2 ========
SFO2         400.1316005 MHz
NUC2                  1H
CPDPRG[2         waltz16
PCPD2              90.00 usec
PLW2         11.99499989 W
PLW12         0.34213999 W
PLW13         0.27713001 W

F2 - Processing parameters
SI                 32768
SF           100.6127710 MHz
WDW                   EM
SSB                    0
LB                  1.00 Hz
GB                     0
PC                  1.40
```

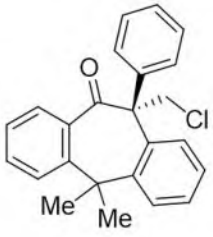

3w

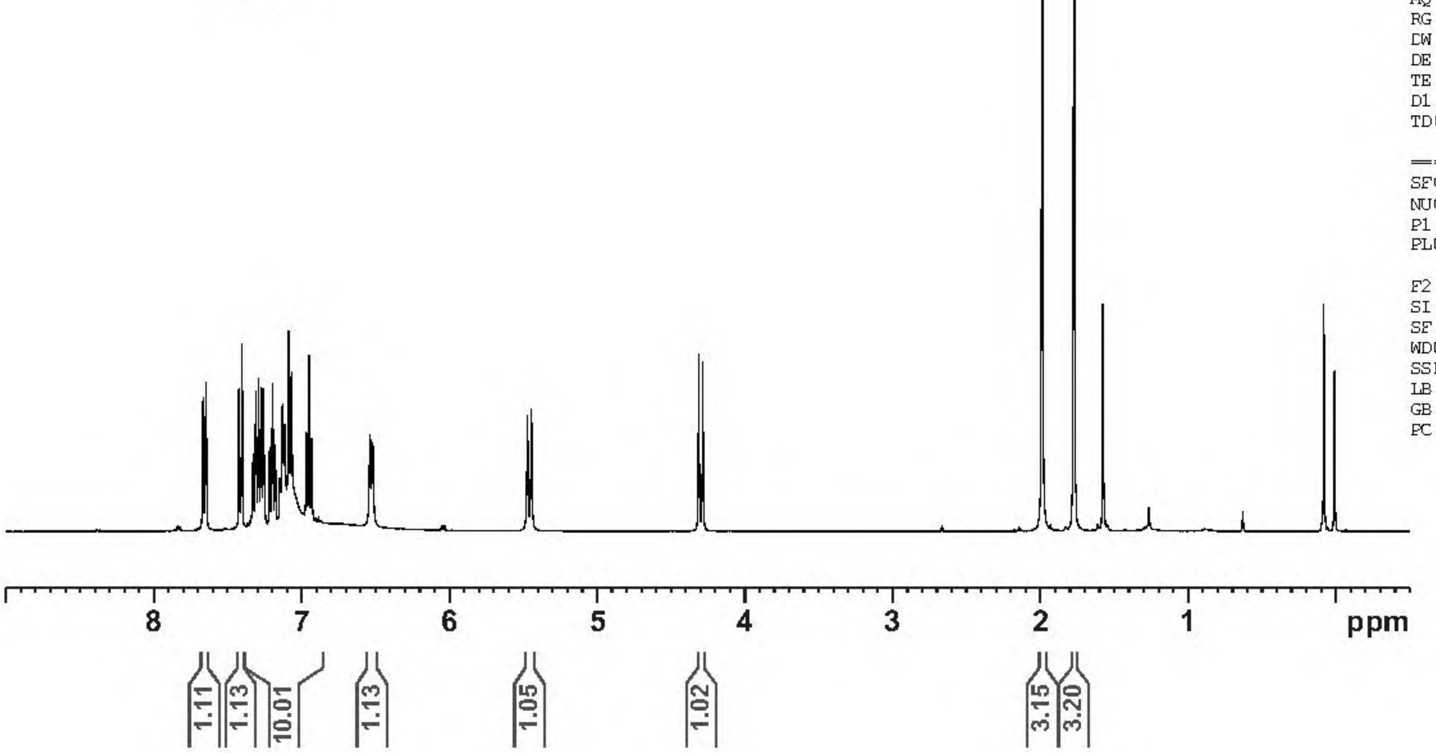


```
Current Data Parameters
NAME     pdt-6 membered ring-H
EXPNO                  1
PROCNO                 1

F2 - Acquisition Parameters
Date_           20240419
Time               22.59
INSTRUM            spect
PROBHD   5 mm PABBO BB/
PULPROG             zg30
TD                 65536
SOLVENT            CDCl3
NS                    16
DS                     2
SWH             8012.820 Hz
FIDRES          0.122266 Hz
AQ             4.0894465 sec
RG                 88.84
DW                62.400 usec
DE                  6.50 usec
TE                 296.8 K
D1            1.00000000 sec
TD0                    1

======== CHANNEL f1 ========
SFO1         400.1324710 MHz
NUC1                  1H
P1                 14.50 usec
PLW1         11.99499989 W

F2 - Processing parameters
SI                 65536
SF           400.1300096 MHz
WDW                   EM
SSB                    0
LB                  0.30 Hz
GB                     0
PC                  1.00
```

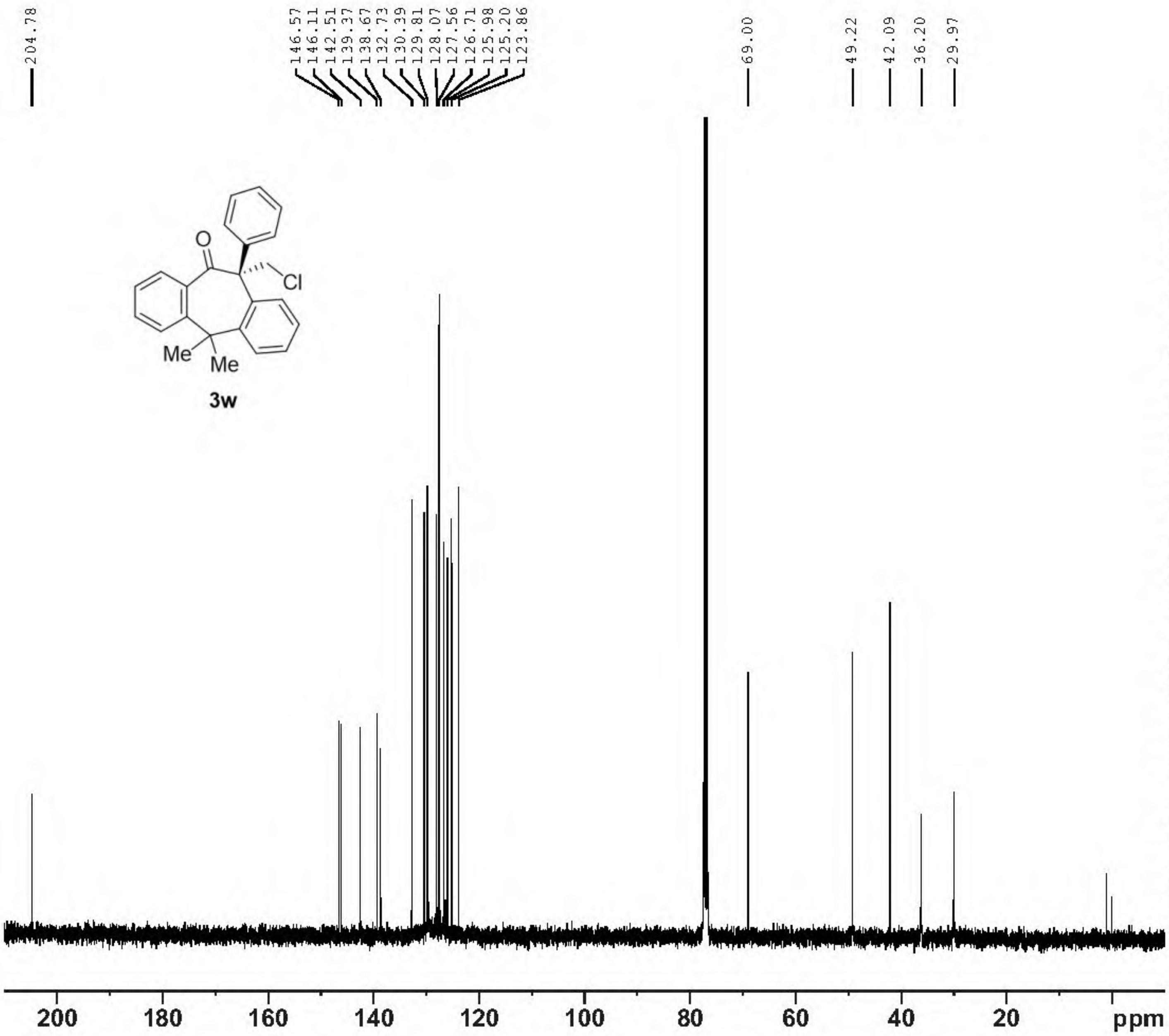


```
Current Data Parameters
NAME        pdt-6 membered ring-C
EXPNO                     1
PROCNO                    1

F2 - Acquisition Parameters
Date_              20240419
Time                  23.57
INSTRUM               spect
PROBHD    5 mm PABBO BB/
PULPROG             zgpg30
TD                    65536
SOLVENT               CDCl3
NS                     1000
DS                        2
SWH              24038.461 Hz
FIDRES            0.366798 Hz
AQ               1.3631488 sec
RG                   196.92
DW                   20.800 usec
DE                     6.50 usec
TE                    298.0 K
D1               2.00000000 sec
D11              0.03000000 sec
TD0                       1

======== CHANNEL f1 ========
SFO1          100.6228298 MHz
NUC1                   13C
P1                     9.70 usec
PLW1           46.98899841 W

======== CHANNEL f2 ========
SFO2          400.1316005 MHz
NUC2                    1H
CPDPRG[2           waltz16
PCPD2                 90.00 usec
PLW2           11.99499989 W
PLW12           0.34213999 W
PLW13           0.27713001 W

F2 - Processing parameters
SI                    32768
SF            100.6127690 MHz
WDW                      EM
SSB                       0
LB                     1.00 Hz
GB                        0
PC                     1.40
```

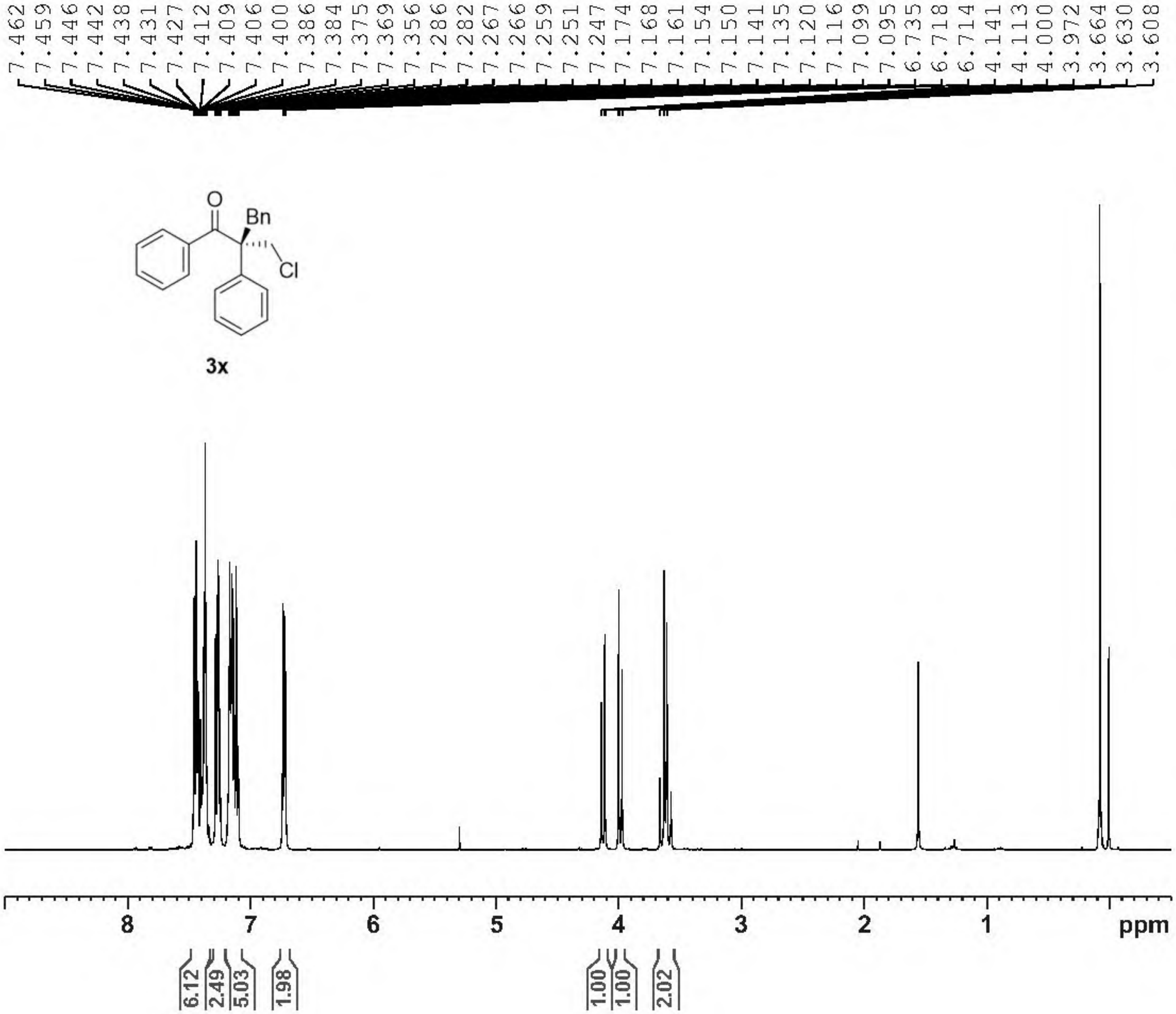

3x

```
Current Data Parameters
NAME        pdt-acyclic-H
EXPNO                   1
PROCNO                  1

F2 - Acquisition Parameters
Date_            20260618
Time                18.26 h
INSTRUM   AvanceNeo 400MHz
PROBHD    Z163739_0629 (
PULPROG              zg30
TD                  65536
SOLVENT             CDCl3
NS                     16
DS                      2
SWH              8196.722 Hz
FIDRES           0.250144 Hz
AQ              3.9976959 sec
RG                    101
DW                 61.000 usec
DE                  13.89 usec
TE                  297.0 K
D1             1.00000000 sec
TD0                     1
SFO1          400.1824711 MHz
NUC1                   1H
P0                   2.67 usec
P1                   8.00 usec
PLW1          21.26700020 W

F2 - Processing parameters
SI                  65536
SF            400.1800098 MHz
WDW                    EM
SSB                     0
LB                   0.30 Hz
GB                      0
PC                   1.00
```

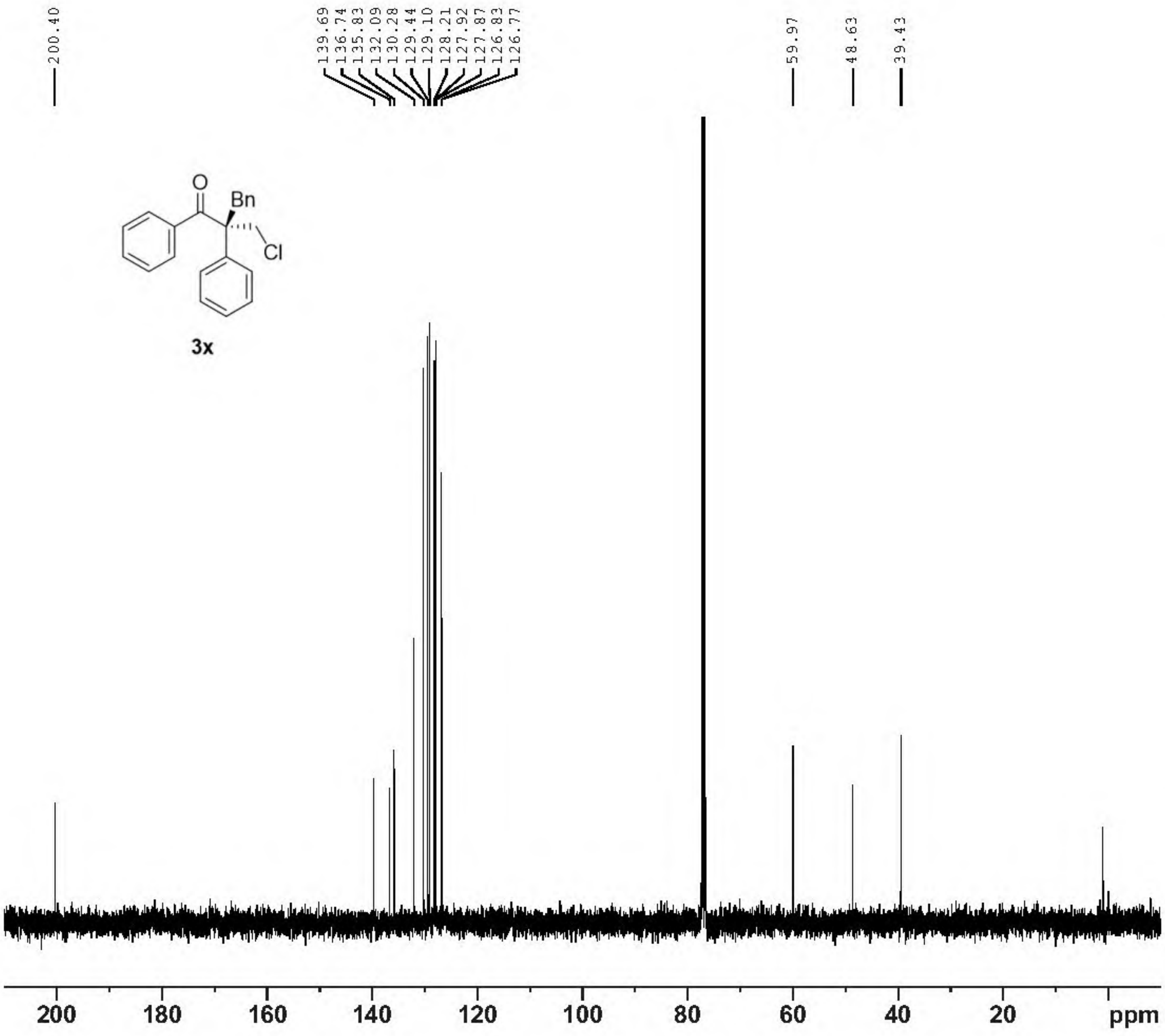

Current Data Parameters	
NAME	pdt-acyclic-C
EXPNO	3
PROCNO	1
**F2 - Acquisition Parameters**	
Date_	20260618
Time	18.36 h
INSTRUM	AvanceNeo 400MHz
PROBHD	Z163739_0629 (
PULPROG	zgpg30
TD	65536
SOLVENT	CDCl3
NS	141
DS	4
SWH	23809.523 Hz
FIDRES	0.726609 Hz
AQ	1.3762560 sec
RG	16
DW	21.000 usec
DE	6.50 usec
TE	297.1 K
D1	2.00000000 sec
D11	0.03000000 sec
TD0	1
SFO1	100.6354036 MHz
NUC1	13C
P0	2.67 usec
P1	8.00 usec
PLW1	85.25399780 W
SFO2	400.1816007 MHz
NUC2	1H
CPDPRG[2	waltz65
PCPD2	90.00 usec
PLW2	21.26700020 W
PLW12	0.16802999 W
PLW13	0.08452000 W
**F2 - Processing parameters**	
SI	32768
SF	100.6253453 MHz
WDW	EM
SSB	0
LB	1.00 Hz
GB	0
PC	1.40

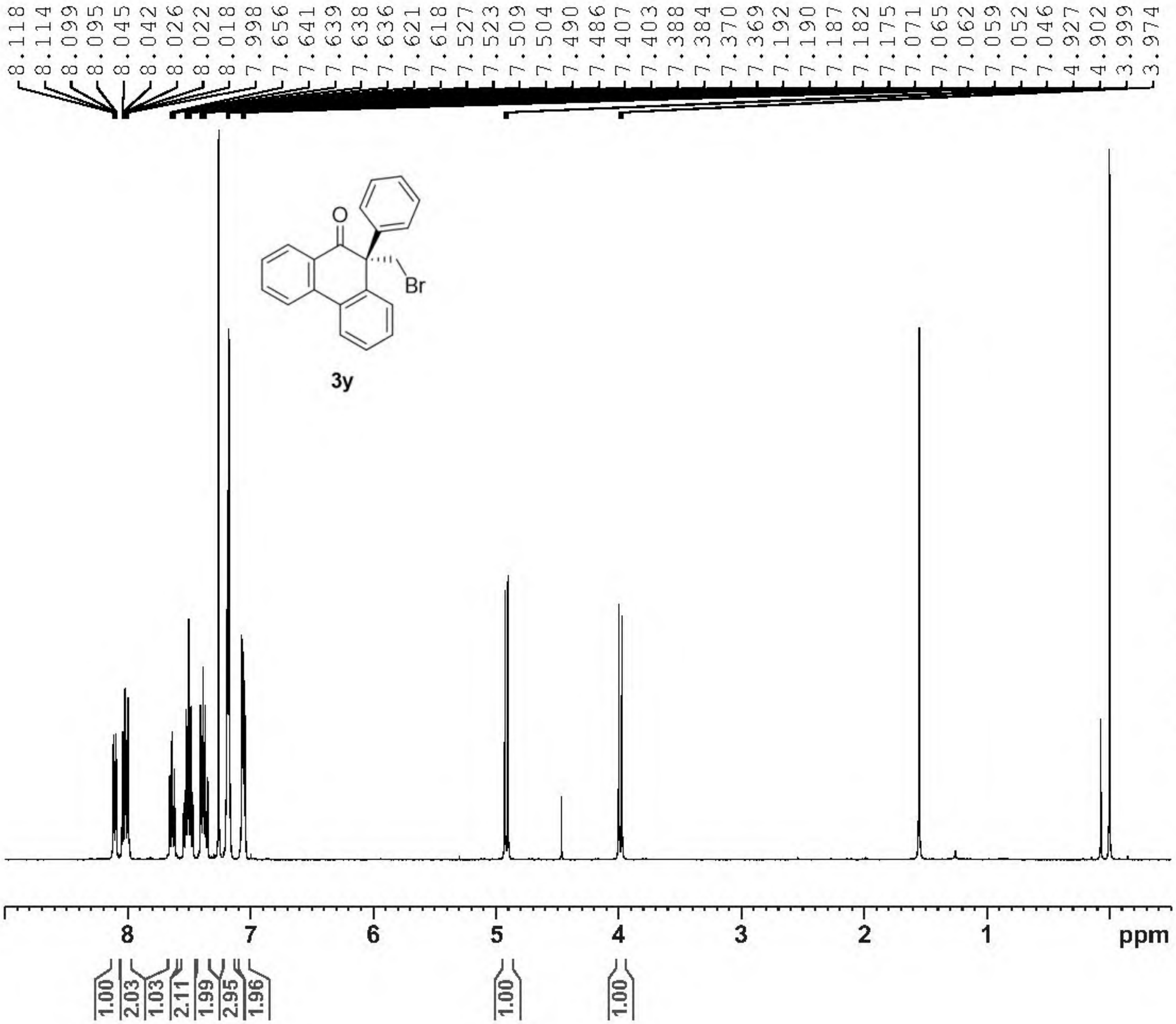

Current Data Parameters
NAME pdt-NaBr-H
EXPNO 1
PROCNO 1

F2 - Acquisition Parameters
Date_ 20260612
Time_ 12.24 h
INSTRUM AvanceNeo 400MHz
PROBHD Z163739_0629 (
PULPROG zg30
TD 65536
SOLVENT CDCl3
NS 16
DS 2
SWH 8196.722 Hz
FIDRES 0.250144 Hz
AQ 3.9976959 sec
RG 101
DW 61.000 usec
DE 13.89 usec
TE 296.8 K
D1 1.00000000 sec
TD0 1
SFO1 400.1824711 MHz
NUC1 1H
P0 2.67 usec
P1 8.00 usec
PLW1 21.26700020 W

F2 - Processing parameters
SI 65536
SF 400.1800097 MHz
WDW EM
SSB 0
LB 0.30 Hz
GB 0
PC 1.00

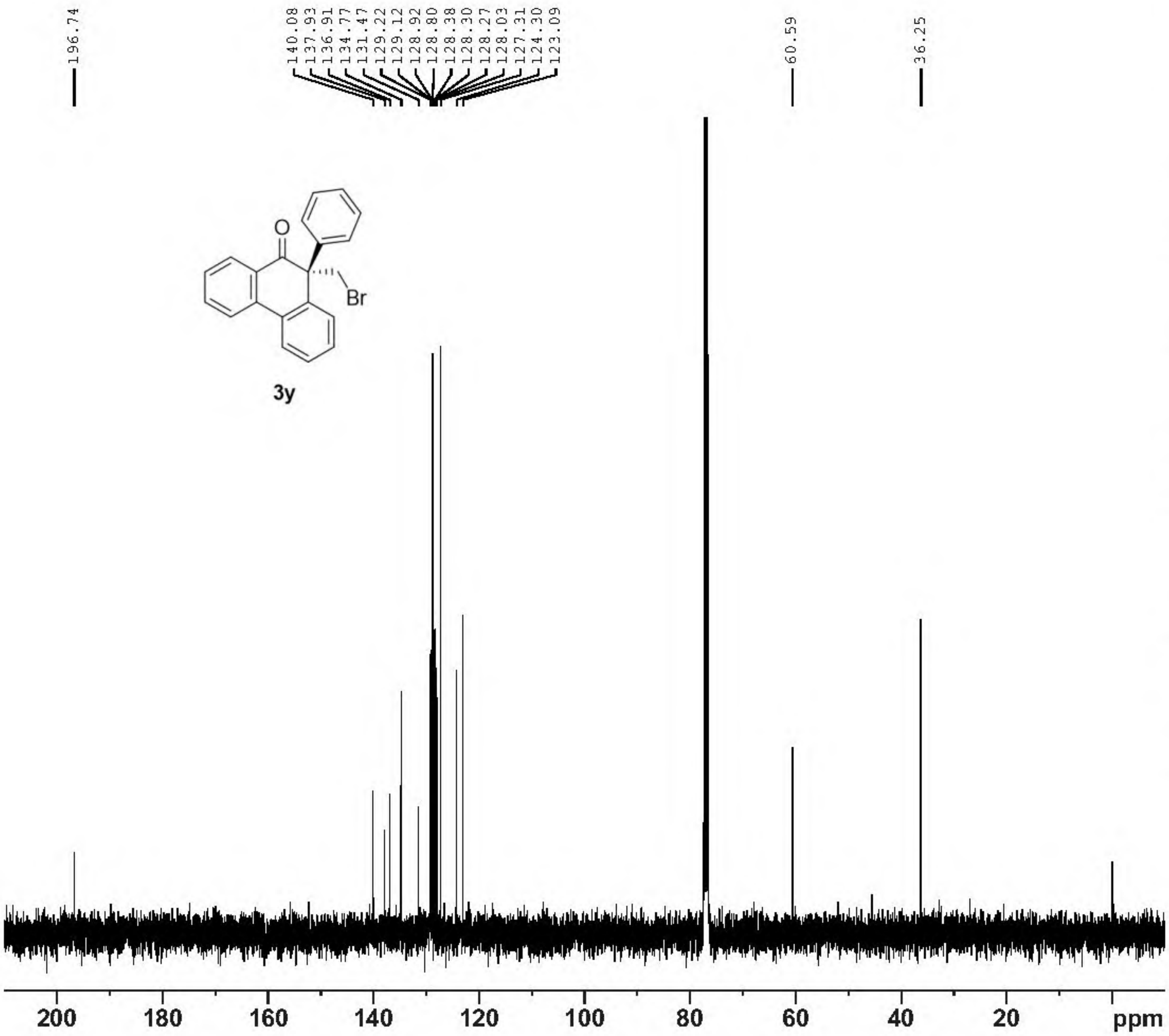

Current Data Parameters
NAME pdt-NaBr-C
EXPNO 3
PROCNO 1

F2 - Acquisition Parameters
Date_ 20260612
Time 12.58 h
INSTRUM AvanceNeo 400MHz
PROBHD Z163739_0629 (
PULPROG zgpg30
TD 65536
SOLVENT CDCl3
NS 555
DS 4
SWH 23809.523 Hz
FIDRES 0.726609 Hz
AQ 1.3762560 sec
RG 16
DW 21.000 usec
DE 6.50 usec
TE 297.5 K
D1 2.00000000 sec
D11 0.03000000 sec
TD0 1
SFO1 100.6354036 MHz
NUC1 13C
P0 2.67 usec
P1 8.00 usec
PLW1 85.25399780 W
SFO2 400.1816007 MHz
NUC2 1H
CPDPRG[2 waltz65
PCPD2 90.00 usec
PLW2 21.26700020 W
PLW12 0.16802999 W
PLW13 0.08452000 W

F2 - Processing parameters
SI 32768
SF 100.6253443 MHz
WDW EM
SSB 0
LB 1.00 Hz
GB 0
PC 1.40

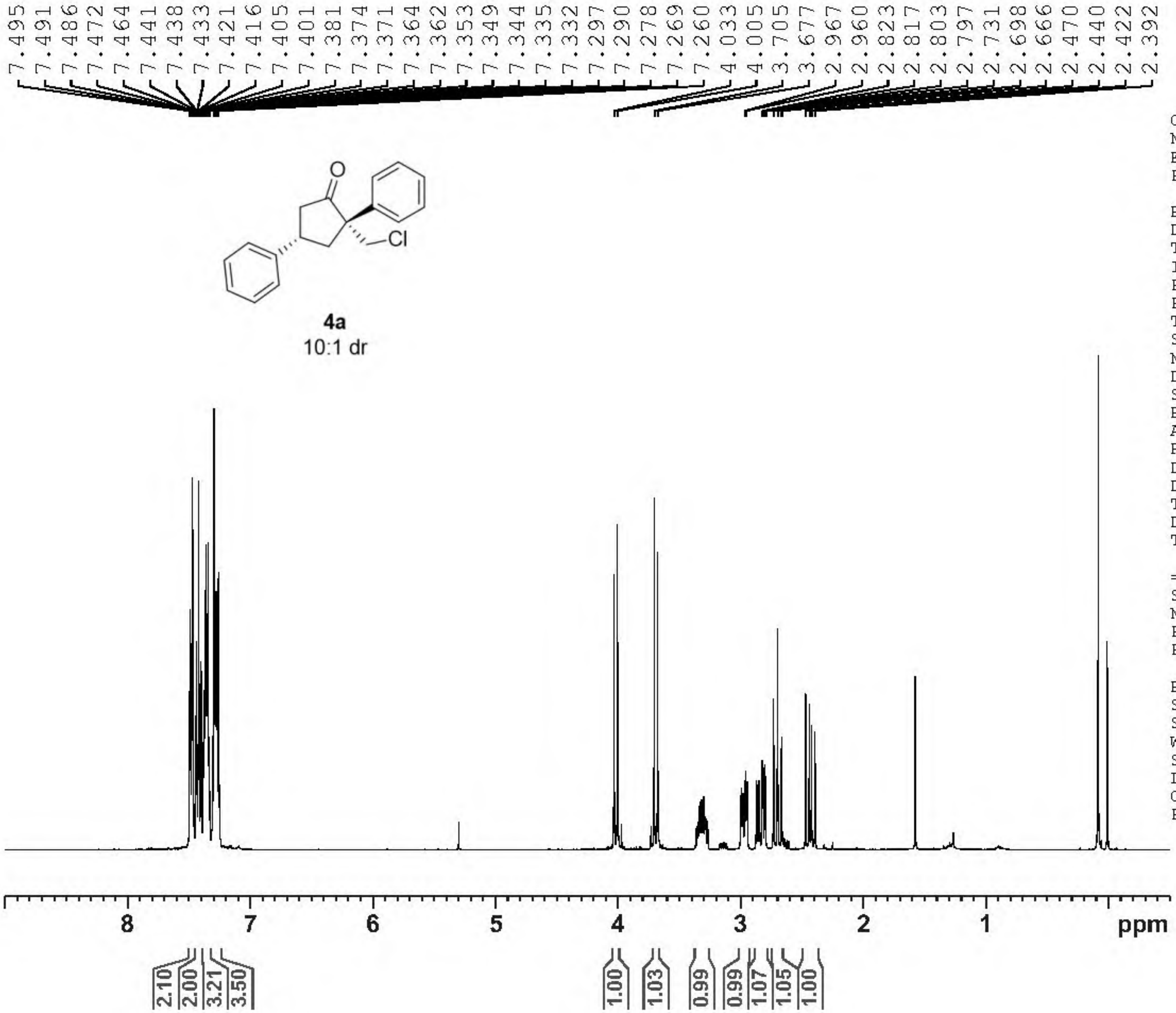

Current Data Parameters
NAME zzj-4-mem-pdt-Ph-H
EXPNO 1
PROCNO 1

F2 - Acquisition Parameters
Date_ 20241228
Time 0.04
INSTRUM spect
PROBHD 5 mm PABBO BB/
PULPROG zg30
TD 65536
SOLVENT CDCl3
NS 16
DS 2
SWH 8012.820 Hz
FIDRES 0.122266 Hz
AQ 4.0894465 sec
RG 82.92
DW 62.400 usec
DE 6.50 usec
TE 296.2 K
D1 1.00000000 sec
TD0 1

======== CHANNEL f1 ========
SFO1 400.1324710 MHz
NUC1 1H
P1 14.50 usec
PLW1 11.99499989 W

F2 - Processing parameters
SI 65536
SF 400.1300098 MHz
WDW EM
SSB 0
LB 0.30 Hz
GB 0
PC 1.00

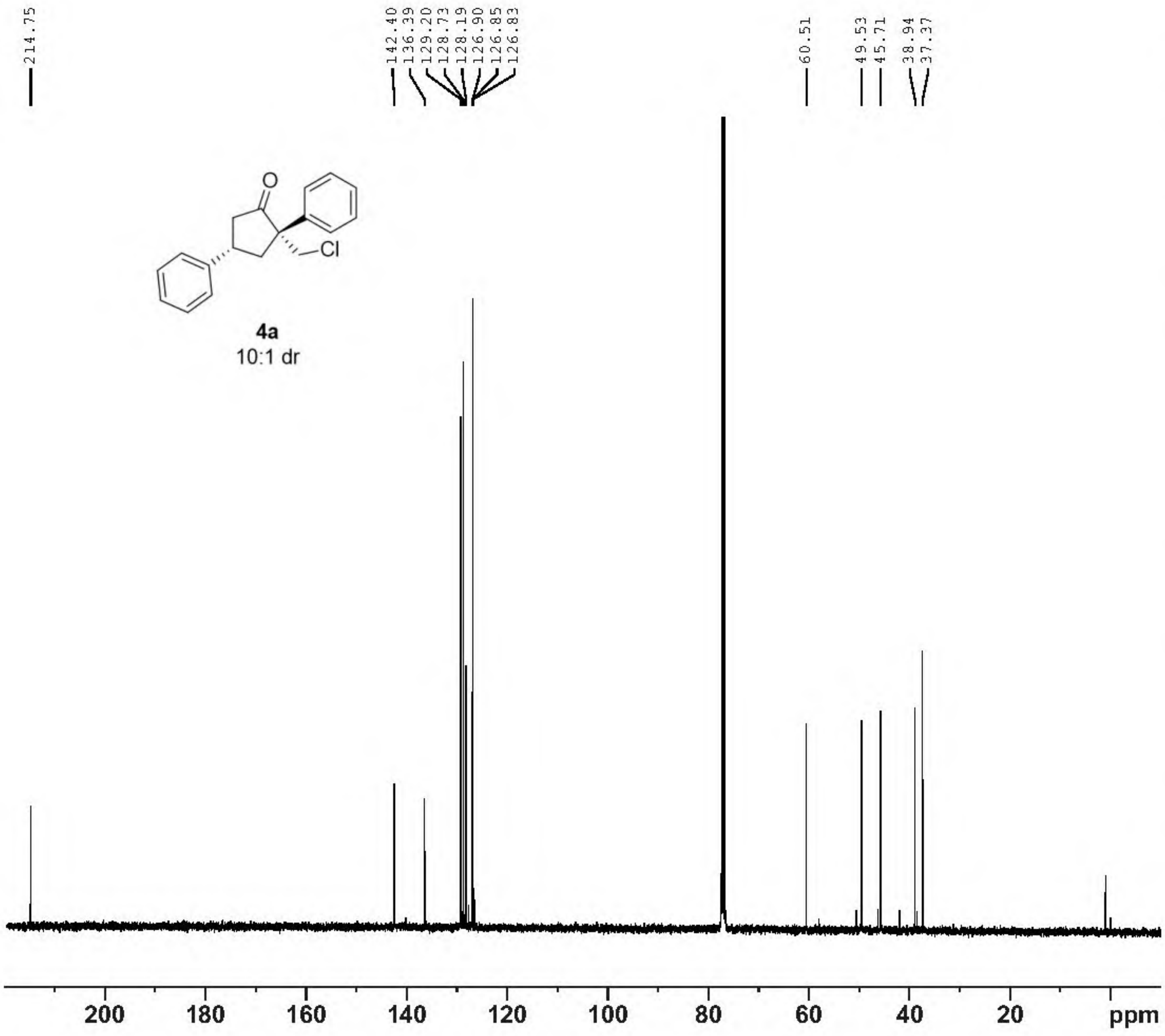


```
Current Data Parameters
NAME        zzj-4-mem-pdt-Ph-C
EXPNO                        1
PROCNO                       1

F2 - Acquisition Parameters
Date_               20241228
Time                    1.02
INSTRUM                spect
PROBHD     5 mm PABBO BB/
PULPROG              zgpg30
TD                     65536
SOLVENT                CDCl3
NS                      1000
DS                         2
SWH                24038.461 Hz
FIDRES              0.366798 Hz
AQ                 1.3631488 sec
RG                    196.92
DW                    20.800 usec
DE                      6.50 usec
TE                     297.1 K
D1                2.00000000 sec
D11               0.03000000 sec
TD0                        1

======== CHANNEL f1 ========
SFO1             100.6228298 MHz
NUC1                     13C
P1                      9.70 usec
PLW1             46.98899841 W

======== CHANNEL f2 ========
SFO2             400.1316005 MHz
NUC2                      1H
CPDPRG[2             waltz16
PCPD2                  90.00 usec
PLW2             11.99499989 W
PLW12             0.34213999 W
PLW13             0.27713001 W

F2 - Processing parameters
SI                     32768
SF               100.6127734 MHz
WDW                       EM
SSB                        0
LB                      1.00 Hz
GB                         0
PC                      1.40
```

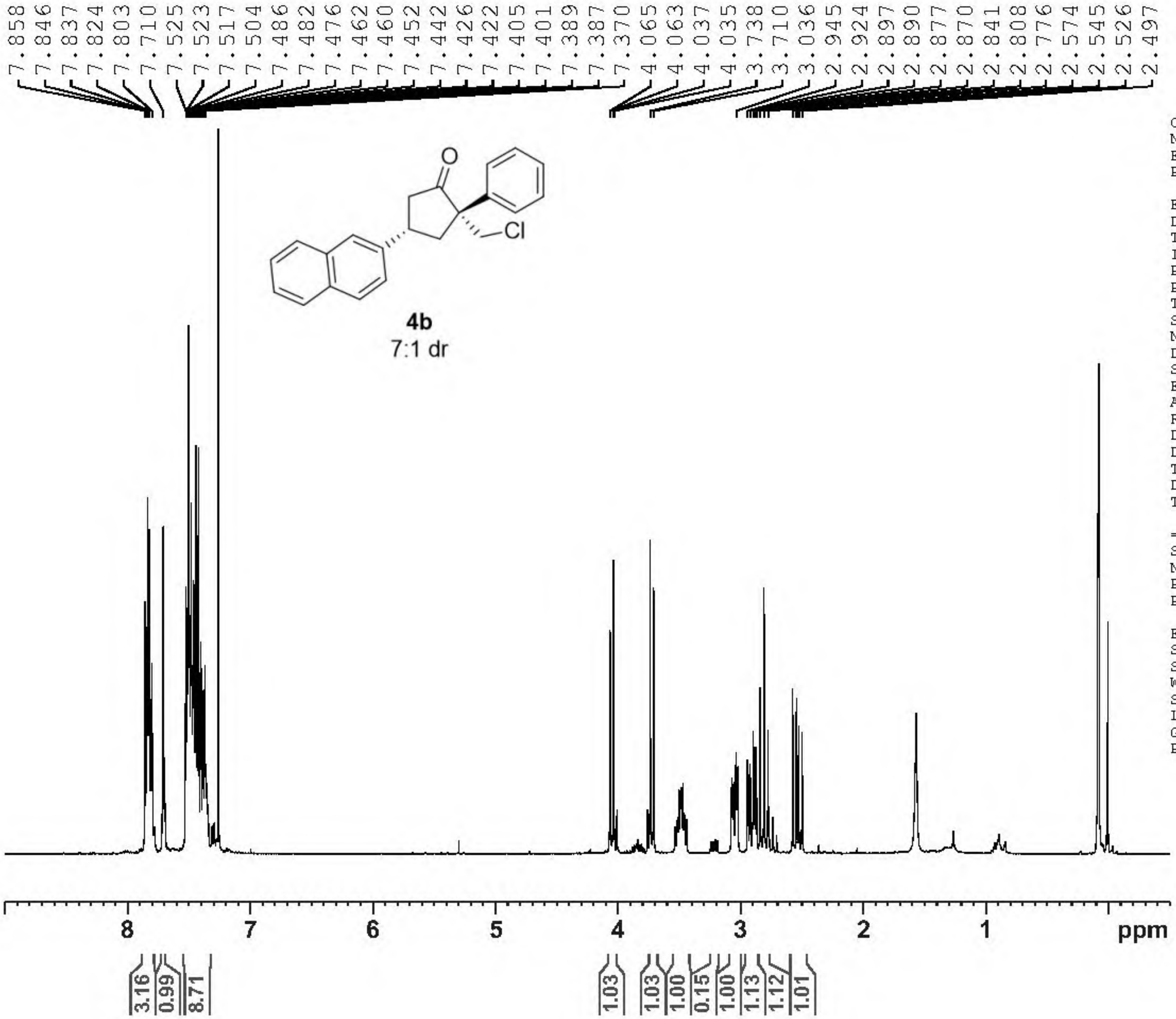

Current Data Parameters
NAME zzj-4-mem-pdt-Ph-Nap-H
EXPNO 1
PROCNO 1

F2 - Acquisition Parameters
Date_ 20260509
Time 11.51
INSTRUM spect
PROBHD 5 mm PABBO BB/
PULPROG zg30
TD 65536
SOLVENT CDCl3
NS 16
DS 2
SWH 8012.820 Hz
FIDRES 0.122266 Hz
AQ 4.0894465 sec
RG 112.31
DW 62.400 usec
DE 6.50 usec
TE 296.9 K
D1 1.00000000 sec
TD0 1

======== CHANNEL f1 ========
SFO1 400.1324710 MHz
NUC1 1H
P1 14.50 usec
PLW1 11.99499989 W

F2 - Processing parameters
SI 65536
SF 400.1300098 MHz
WDW EM
SSB 0
LB 0.30 Hz
GB 0
PC 1.00

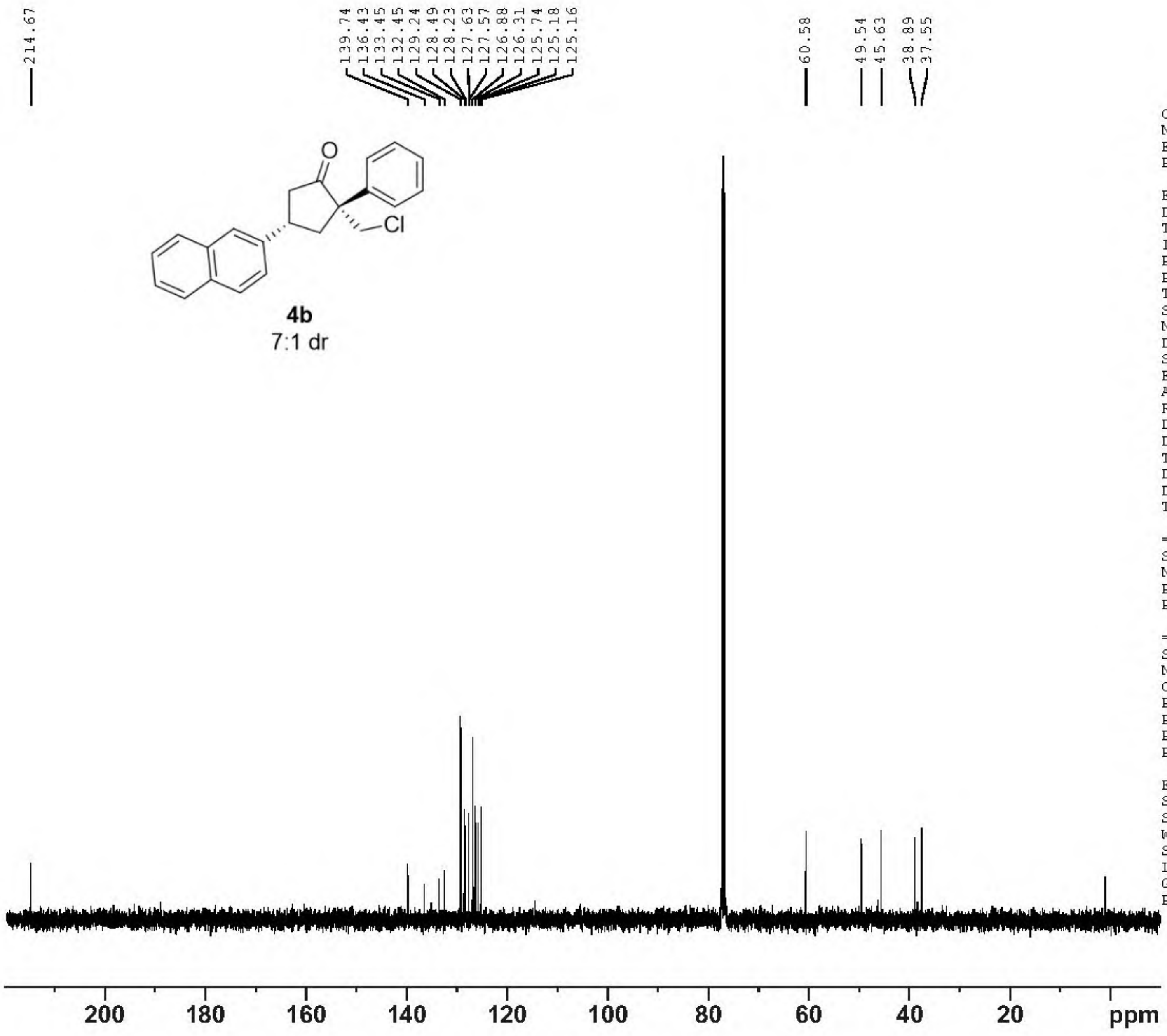

Current Data Parameters
NAME zzj-4-mem-pdt-Ph-Nap-C
EXPNO 1
PROCNO 1

F2 - Acquisition Parameters
Date_ 20260509
Time 11.56
INSTRUM spect
PROBHD 5 mm PABBO BB/
PULPROG zgpg30
TD 65536
SOLVENT CDCl3
NS 122
DS 2
SWH 24038.461 Hz
FIDRES 0.366798 Hz
AQ 1.3631488 sec
RG 196.92
DW 20.800 usec
DE 6.50 usec
TE 297.5 K
D1 2.00000000 sec
D11 0.03000000 sec
TD0 1

======== CHANNEL f1 ========
SFO1 100.6228298 MHz
NUC1 13C
P1 9.70 usec
PLW1 46.98899841 W

======== CHANNEL f2 ========
SFO2 400.1316005 MHz
NUC2 1H
CPDPRG[2 waltz16
PCPD2 90.00 usec
PLW2 11.99499989 W
PLW12 0.34213999 W
PLW13 0.27713001 W

F2 - Processing parameters
SI 32768
SF 100.6127732 MHz
WDW EM
SSB 0
LB 1.00 Hz
GB 0
PC 1.40

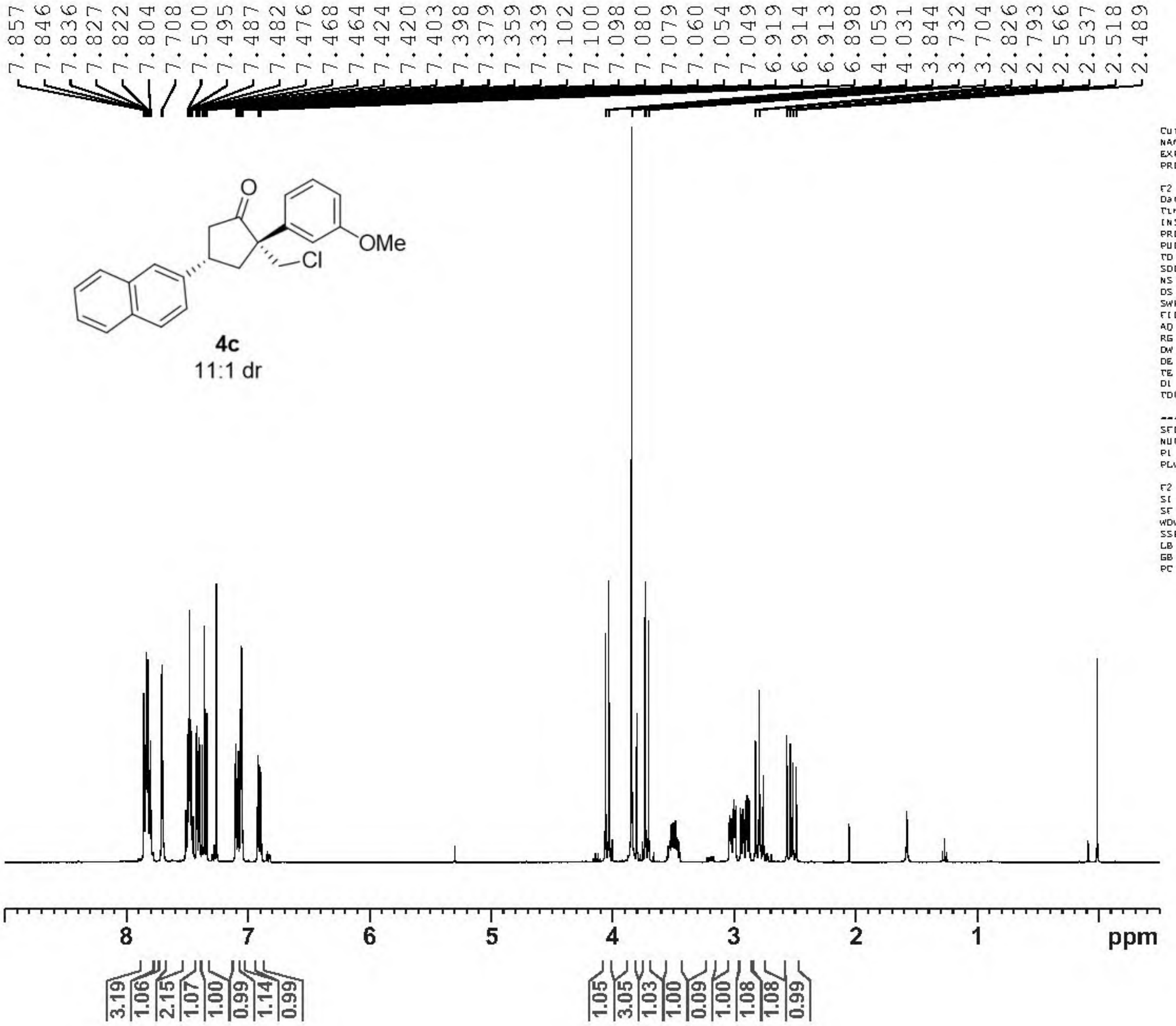

4c
11:1 dr

Current Data Parameters
NAME zzj-4-mem-pdc-alkene-m-OMe-Ph-Nap-H
EXPNO 1
PROCNO 1

F2 - Acquisition Parameters
Date_ 20260328
Time 11.08
INSTRUM spect
PROBHD 5 mm PABBO BB/
PULPROG zg30
TD 65536
SOLVENT CDCl3
NS 16
DS 2
SWH 8012.820 Hz
FIDRES 0.122266 Hz
AQ 4.0894465 sec
RG 82.92
DW 62.400 usec
DE 6.50 usec
TE 296.7 K
D1 1.00000000 sec
TD0 1

======== CHANNEL f1 ========
SFO1 400.1324710 MHz
NUC1 1H
P1 14.50 usec
PLW1 11.99499989 W

F2 - Processing parameters
SI 65536
SF 400.1300096 MHz
WDW EM
SSB 0
LB 0.30 Hz
GB 0
PC 1.00

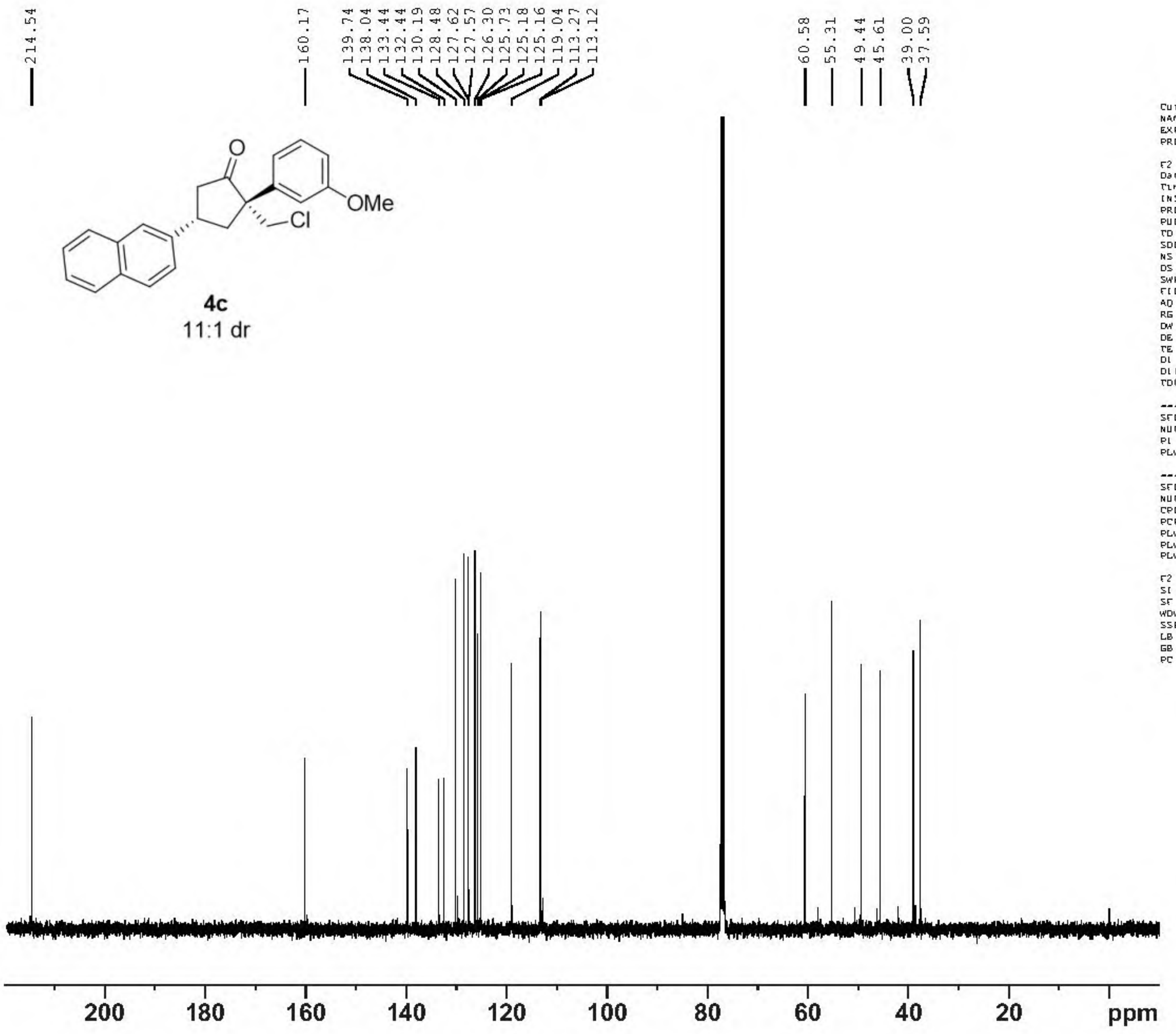


```
Current Data Parameters
NAME     zzj-4-mem-pdc-alkene-m-OMe-Ph-Nap-C
EXPNO                   1
PROCNO                  1

F2 - Acquisition Parameters
Date_          20260328
Time              11.43
INSTRUM           spect
PROBHD   5 mm PABBO BB/
PULPROG          zgpg30
TD                65536
SOLVENT           CDCl3
NS                  600
DS                    2
SWH           24038.461 Hz
FIDRES         0.366798 Hz
AQ            1.3631488 sec
RG               196.92
DW               20.800 usec
DE                 6.50 usec
TE                297.4 K
D1           2.00000000 sec
D11          0.03000000 sec
TD0                   1

======== CHANNEL f1 ========
SFO1        100.6228298 MHz
NUC1                13C
P1                 9.70 usec
PLW1        46.98899841 W

======== CHANNEL f2 ========
SFO2        400.1316005 MHz
NUC2                 1H
CPDPRG[2         waltz16
PCPD2             90.00 usec
PLW2        11.99499989 W
PLW12        0.34213999 W
PLW13        0.27713001 W

F2 - Processing parameters
SI                32768
SF          100.6127738 MHz
WDW                  EM
SSB                   0
LB                 1.00 Hz
GB                    0
PC                 1.40
```

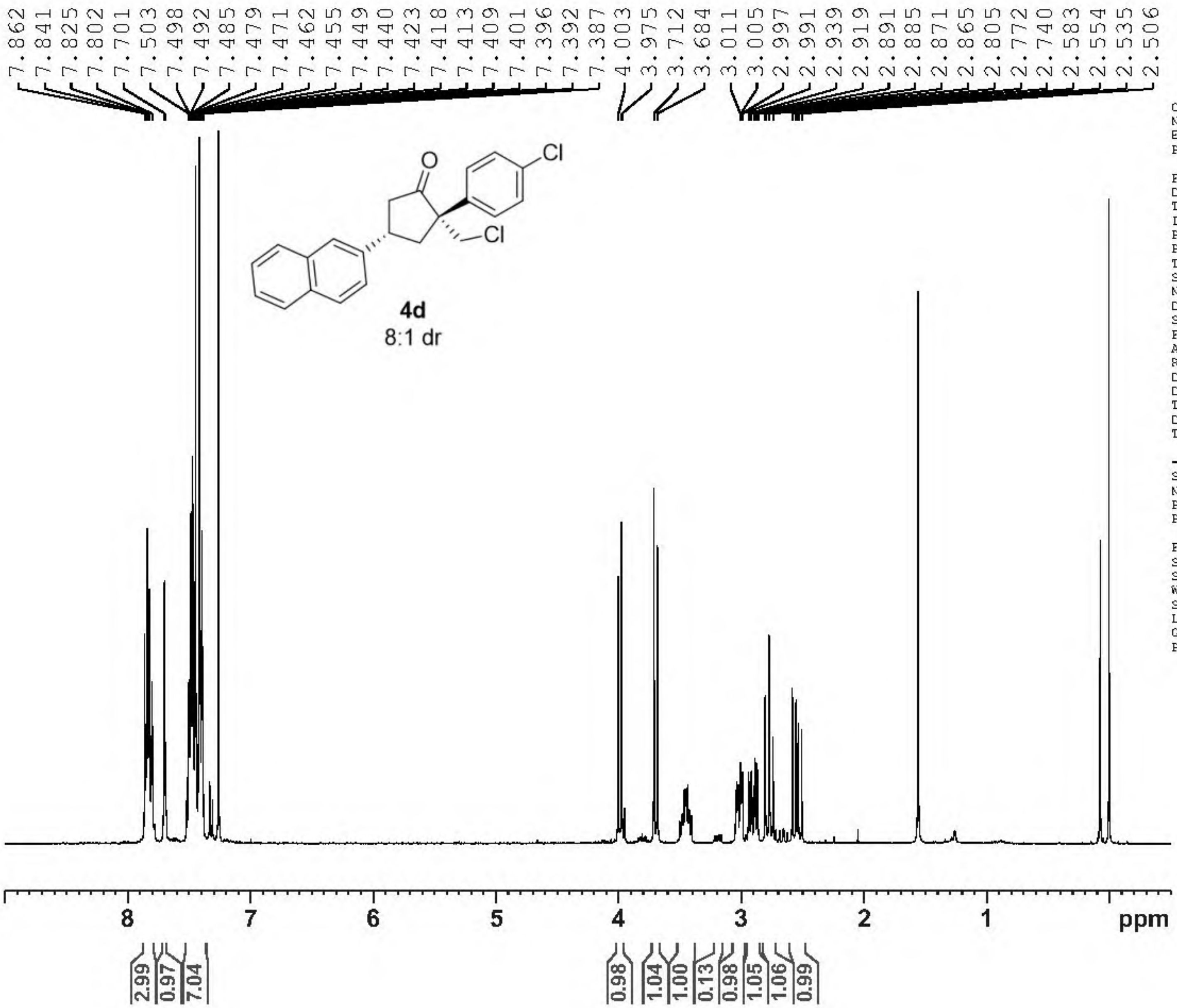


```
Current Data Parameters
NAME      zzj-4-mem-pdt-p-Cl-Ph-Nap-H
EXPNO                 1
PROCNO                1

F2 - Acquisition Parameters
Date_          20260313
Time              23.37
INSTRUM           spect
PROBHD   5 mm PABBO BB/
PULPROG            zg30
TD                65536
SOLVENT           CDCl3
NS                   16
DS                    2
SWH            8012.820 Hz
FIDRES         0.122266 Hz
AQ            4.0894465 sec
RG               142.88
DW               62.400 usec
DE                 6.50 usec
TE                295.6 K
D1           1.00000000 sec
TD0                   1

-------- CHANNEL f1 --------
SFO1        400.1324710 MHz
NUC1                 1H
P1                14.50 usec
PLW1        11.99499989 W

F2 - Processing parameters
SI                65536
SF          400.1300098 MHz
WDW                  EM
SSB                   0
LB                 0.30 Hz
GB                    0
PC                 1.00
```

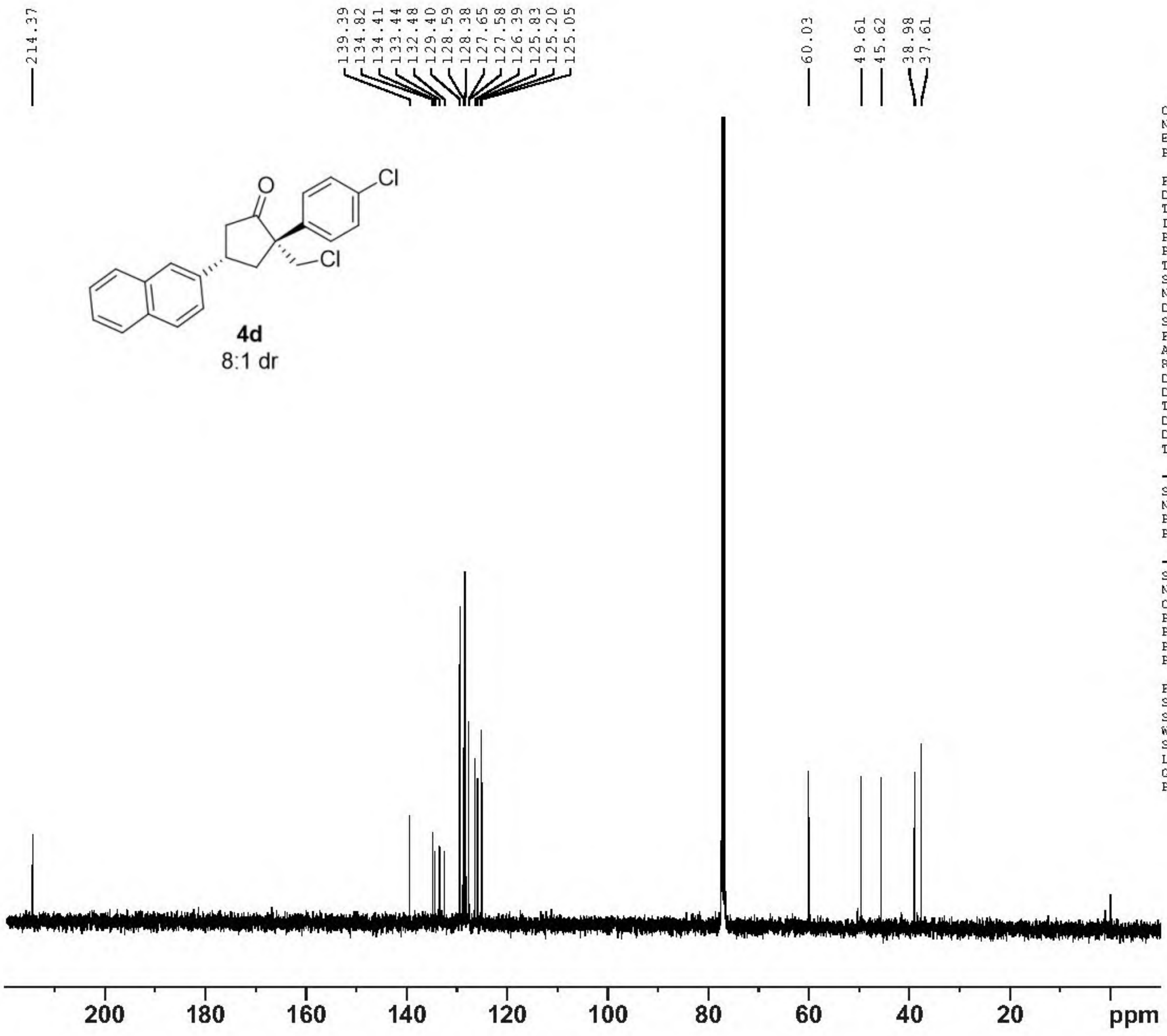


```
Current Data Parameters
NAME      zzj-4-mem-pdt-p-Cl-Ph-Nap-C
EXPNO                    1
PROCNO                   1

F2 - Acquisition Parameters
Date_            20260314
Time                 0.23
INSTRUM             spect
PROBHD    5 mm PABBO BB/
PULPROG            zgpg30
TD                  65536
SOLVENT             CDCl3
NS                    800
DS                      2
SWH             24038.461 Hz
FIDRES           0.366798 Hz
AQ              1.3631488 sec
RG                 196.92
DW                 20.800 usec
DE                   6.50 usec
TE                  296.6 K
D1             2.00000000 sec
D11            0.03000000 sec
TD0                     1

-------- CHANNEL f1 --------
SFO1          100.6228298 MHz
NUC1                  13C
P1                   9.70 usec
PLW1          46.98899841 W

-------- CHANNEL f2 --------
SFO2          400.1316005 MHz
NUC2                   1H
CPDPRG[2          waltz16
PCPD2               90.00 usec
PLW2          11.99499989 W
PLW12          0.34213999 W
PLW13          0.27713001 W

F2 - Processing parameters
SI                  32768
SF            100.6127721 MHz
WDW                    EM
SSB                     0
LB                   1.00 Hz
GB                      0
PC                   1.40
```

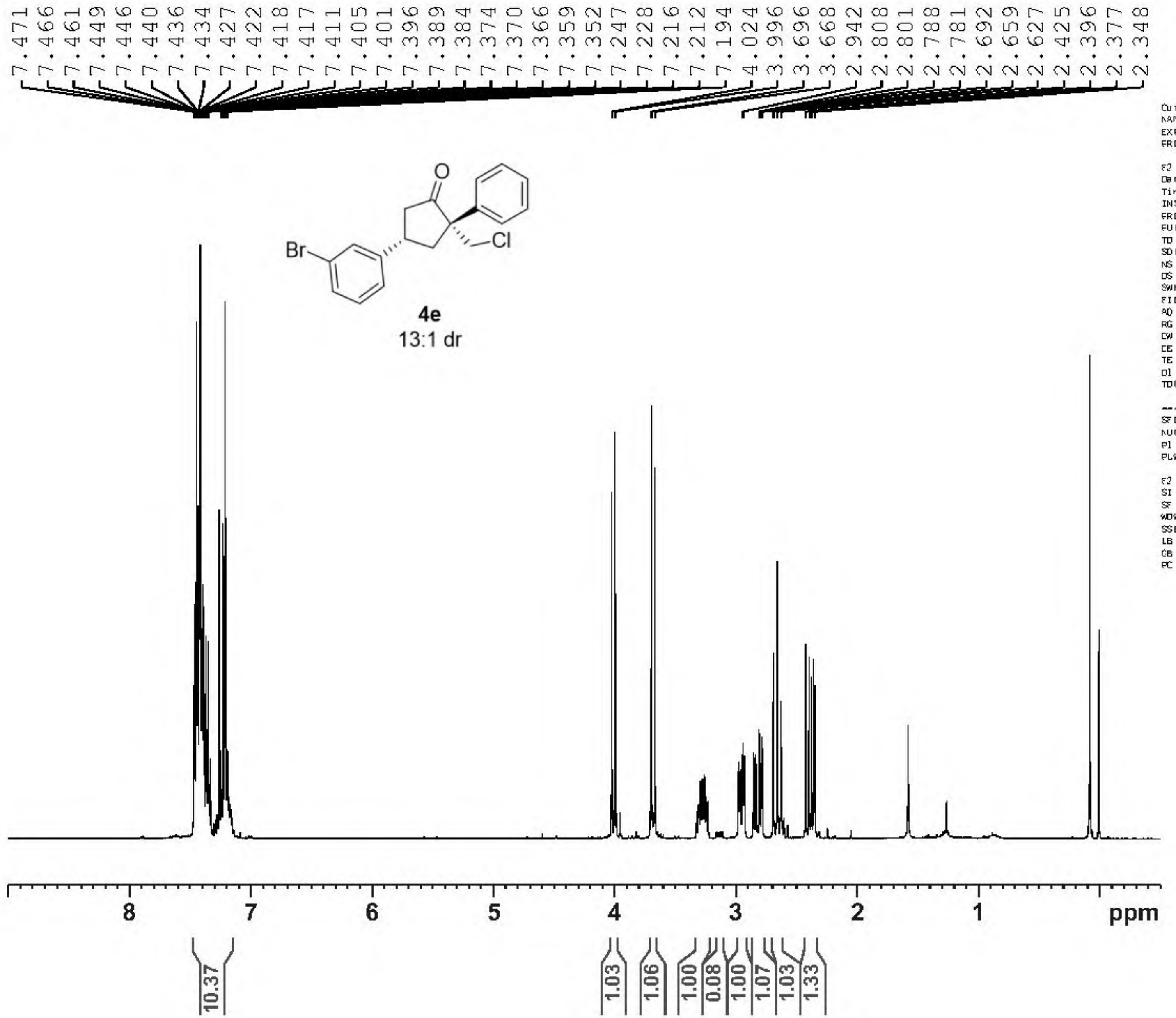


```
Current Data Parameters
NAME       zzj-4-mem-pdc-alkene Ph-m-Br Ph-H
EXPNO                     1
PROCNO                    1

F2 - Acquisition Parameters
Date_              20260423
Time                   2.44
INSTRUM               spect
PROBHD    5 mm PABBO BB/
PULPROG                zg30
TD                    65536
SOLVENT               CDCl3
NS                       16
DS                        2
SWH              8012.820 Hz
FIDRES           0.122266 Hz
AQ              4.0894465 sec
RG                    82.92
DW                   62.400 usec
DE                     6.50 usec
TE                    297.3 K
D1             1.00000000 sec
TD0                       1

======== CHANNEL f1 ========
SFO1          400.1324710 MHz
NUC1                     1H
P1                    14.50 usec
PLW1          11.99499989 W

F2 - Processing parameters
SI                    65536
SF            400.1300097 MHz
WDW                      EM
SSB                       0
LB                     0.30 Hz
GB                        0
PC                     1.00
```

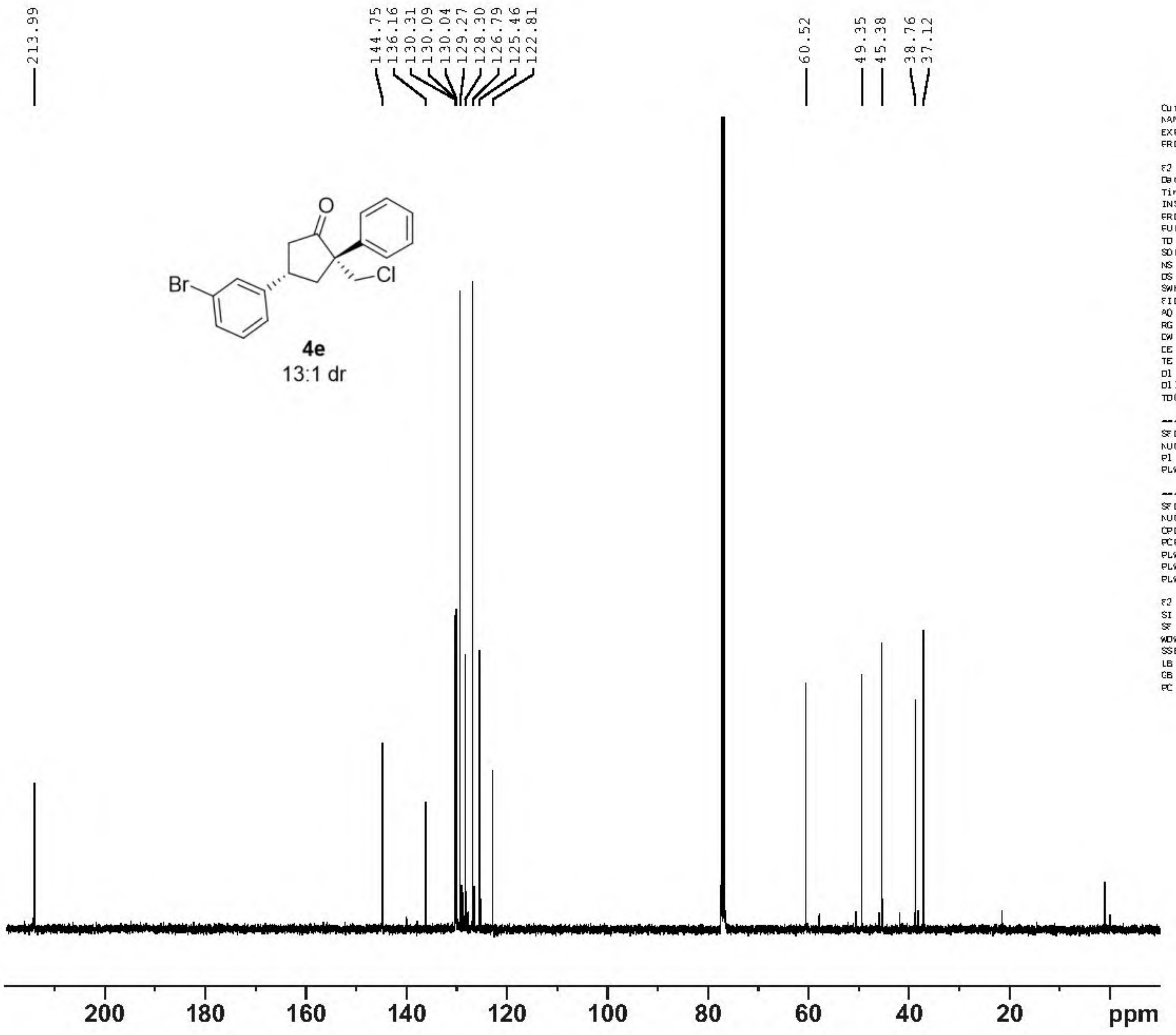


```
Current Data Parameters
NAME      zzj-4-mem-pdc-alkene Ph-m-Br Ph-C
EXPNO                   1
PROCNO                  1

F2 - Acquisition Parameters
Date_            20260423
Time                 3.30
INSTRUM             spect
PROBHD   5 mm PABBO BB/
PULPROG            zgpg30
TD                  65536
SOLVENT             CDCl3
NS                    800
DS                      2
SWH            24038.461 Hz
FIDRES          0.366798 Hz
AQ             1.3631488 sec
RG                 196.92
DW                 20.800 usec
DE                   6.50 usec
TE                  298.3 K
D1            2.00000000 sec
D11           0.03000000 sec
TD0                     1

======== CHANNEL f1 ========
SFO1         100.6228298 MHz
NUC1                  13C
P1                   9.70 usec
PLW1         46.98899841 W

======== CHANNEL f2 ========
SFO2         400.1316005 MHz
NUC2                   1H
CPDPRG[2           waltz16
PCPD2               90.00 usec
PLW2         11.99499989 W
PLW12         0.34213999 W
PLW13         0.27713001 W

F2 - Processing parameters
SI                  32768
SF           100.6127726 MHz
WDW                    EM
SSB                     0
LB                   1.00 Hz
GB                      0
PC                   1.40
```

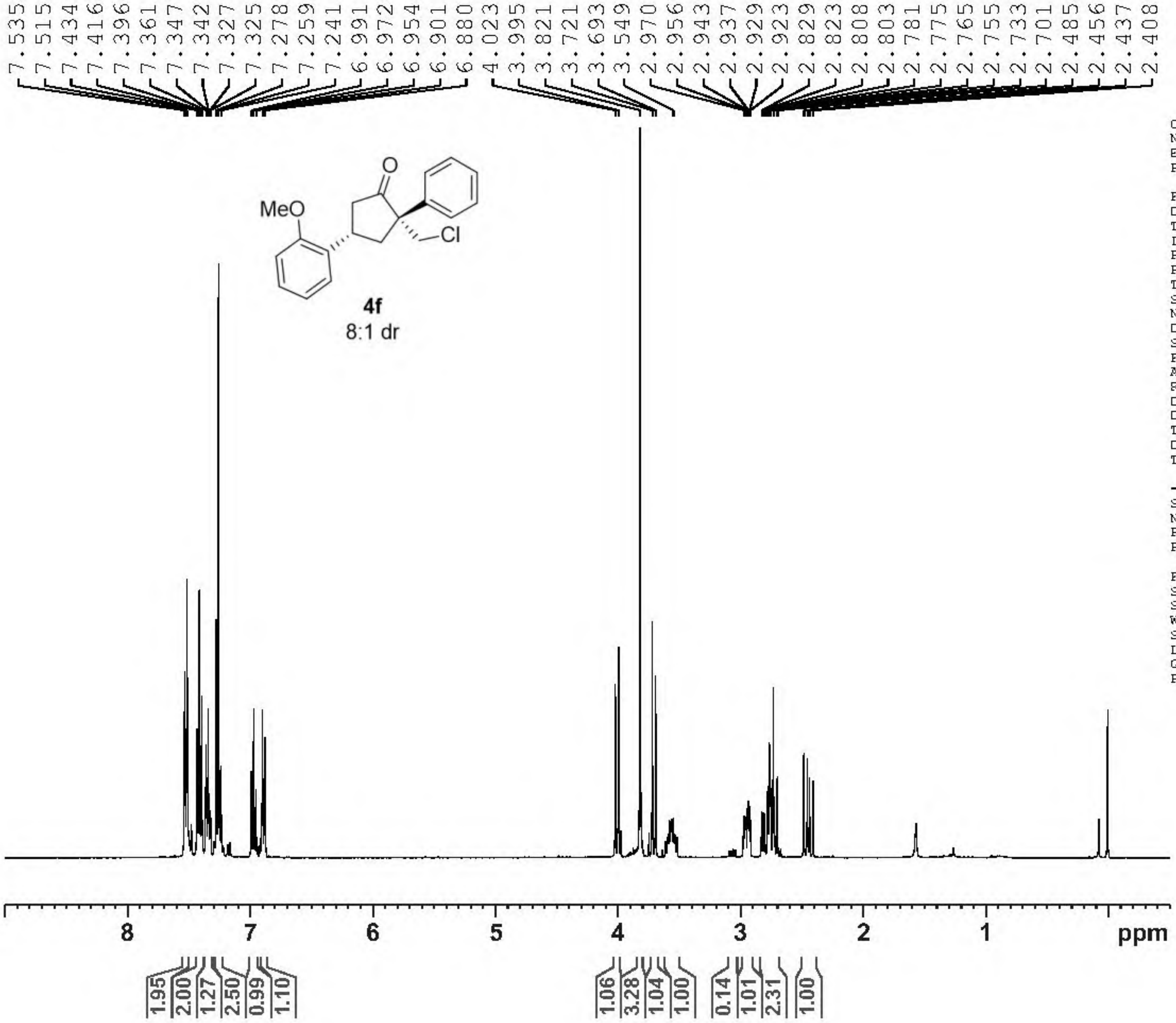

Current Data Parameters
NAME zzj-4-mem-pdt-Ph-oOMe-Ph-H
EXPNO 1
PROCNO 1

F2 - Acquisition Parameters
Date_ 20260509
Time 12.03
INSTRUM spect
PROBHD 5 mm PABBO BB/
PULPROG zg30
TD 65536
SOLVENT CDCl3
NS 16
DS 2
SWH 8012.820 Hz
FIDRES 0.122266 Hz
AQ 4.0894465 sec
RG 103.52
DW 62.400 usec
DE 6.50 usec
TE 296.7 K
D1 1.00000000 sec
TD0 1

-------- CHANNEL f1 --------
SFO1 400.1324710 MHz
NUC1 1H
P1 14.50 usec
PLW1 11.99499989 W

F2 - Processing parameters
SI 65536
SF 400.1300099 MHz
WDW EM
SSB 0
LB 0.30 Hz
GB 0
PC 1.00

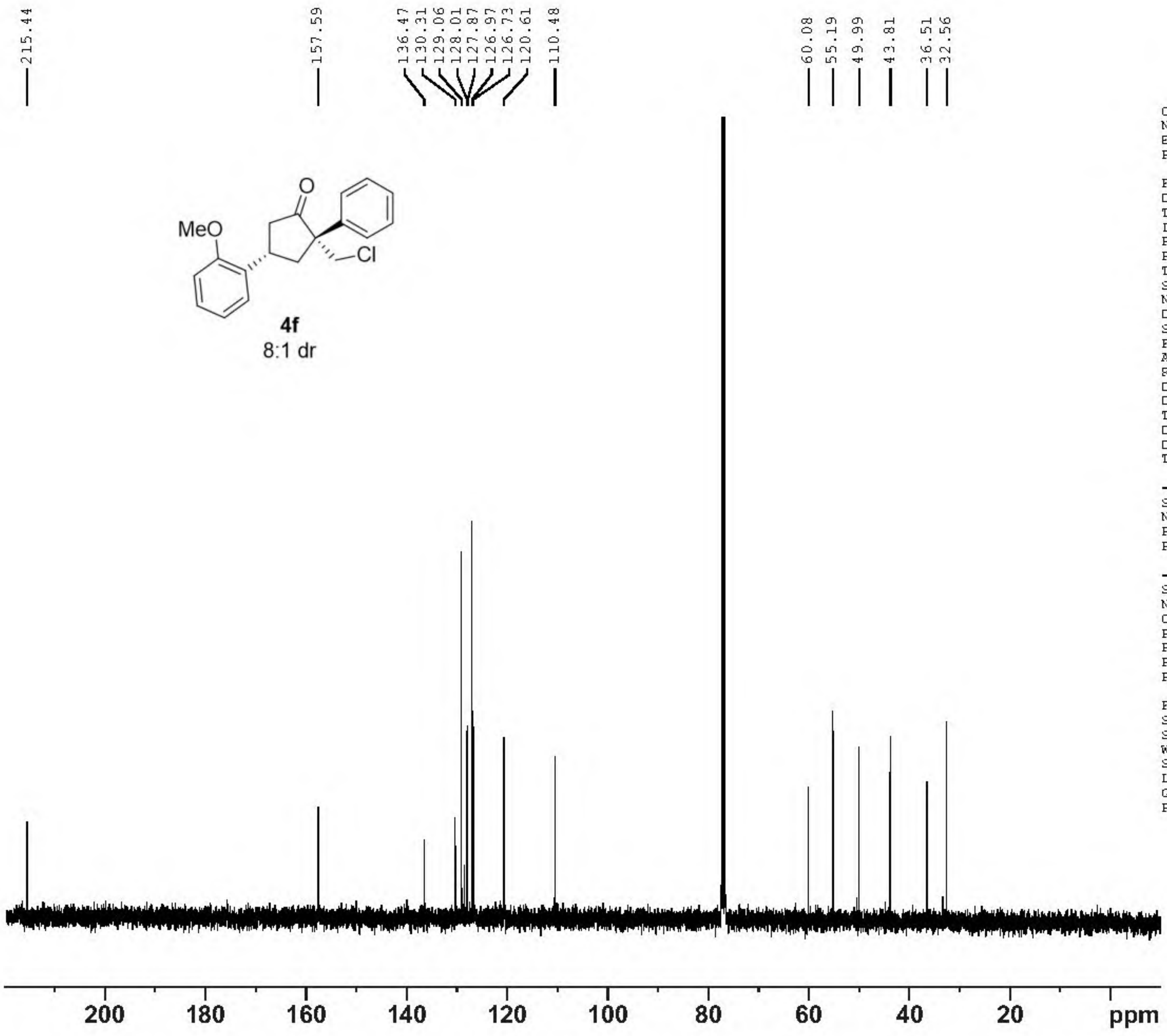


```
Current Data Parameters
NAME      zzj-4-mem-pdt-Ph-oOMe-Ph-C
EXPNO                1
PROCNO               1

F2 - Acquisition Parameters
Date_          20260509
Time              12.06
INSTRUM           spect
PROBHD   5 mm PABBO BB/
PULPROG          zgpg30
TD                65536
SOLVENT           CDCl3
NS                  210
DS                    2
SWH           24038.461 Hz
FIDRES         0.366798 Hz
AQ            1.3631488 sec
RG               196.92
DW               20.800 usec
DE                 6.50 usec
TE                297.3 K
D1           2.00000000 sec
D11          0.03000000 sec
TD0                   1

-------- CHANNEL f1 --------
SFO1        100.6228298 MHz
NUC1                13C
P1                 9.70 usec
PLW1        46.98899841 W

-------- CHANNEL f2 --------
SFO2        400.1316005 MHz
NUC2                 1H
CPDPRG[2        waltz16
PCPD2             90.00 usec
PLW2        11.99499989 W
PLW12        0.34213999 W
PLW13        0.27713001 W

F2 - Processing parameters
SI                32768
SF          100.6127728 MHz
WDW                  EM
SSB                   0
LB                 1.00 Hz
GB                    0
PC                 1.40
```

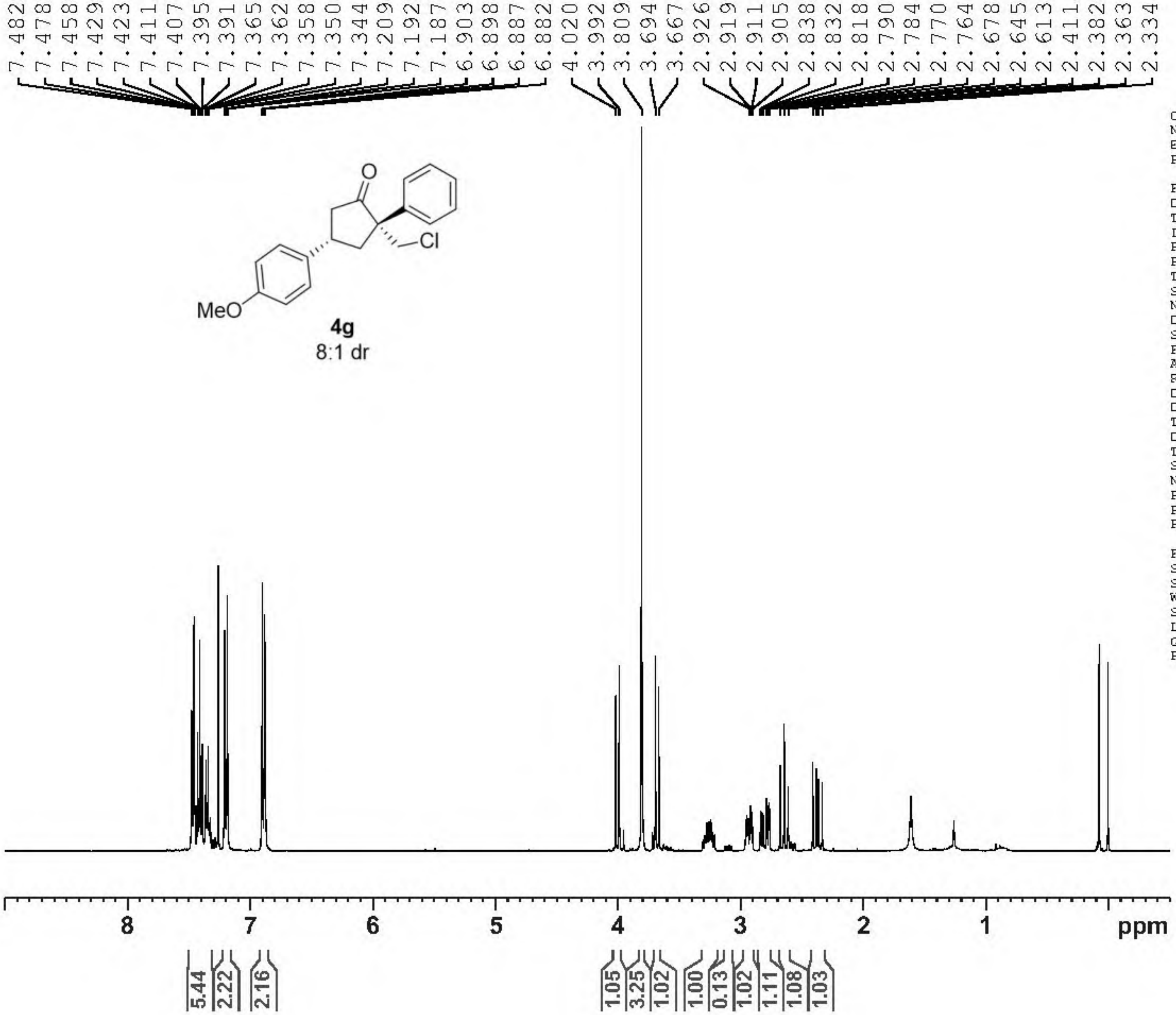

4g
8:1 dr

Current Data Parameters
NAME zzj-4-mem-pdt-Ph-pOMe-Ph-H
EXPNO 1
PROCNO 1

F2 - Acquisition Parameters
Date_ 20260508
Time 20.04 h
INSTRUM AvanceNeo 400MHz
PROBHD Z163739_0629 (
PULPROG zg30
TD 65536
SOLVENT CDCl3
NS 7
DS 2
SWH 8196.722 Hz
FIDRES 0.250144 Hz
AQ 3.9976959 sec
RG 101
DW 61.000 usec
DE 13.89 usec
TE 297.0 K
D1 1.00000000 sec
TD0 1
SFO1 400.1824711 MHz
NUC1 1H
P0 2.67 usec
P1 8.00 usec
PLW1 21.26700020 W

F2 - Processing parameters
SI 65536
SF 400.1800093 MHz
WDW EM
SSB 0
LB 0.30 Hz
GB 0
PC 1.00

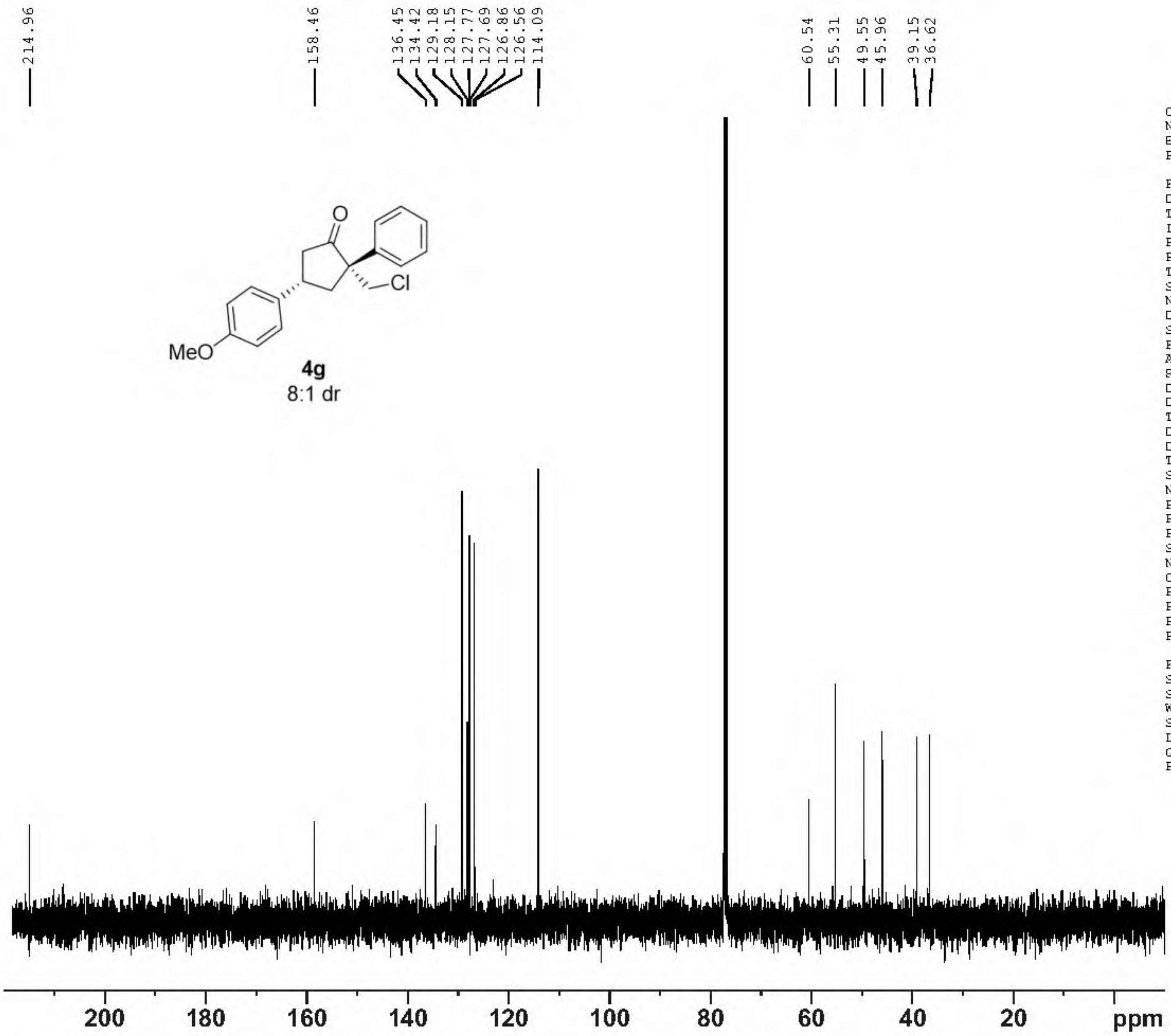


```
Current Data Parameters
NAME      zzj-4-mem-pdt-Ph-pOMe-Ph-C
EXPNO                    3
PROCNO                   1

F2 - Acquisition Parameters
Date_            20260508
Time               20.09 h
INSTRUM  AvanceNeo 400MHz
PROBHD   Z163739_0629 (
PULPROG            zgpg30
TD                  65536
SOLVENT             CDCl3
NS                     50
DS                      4
SWH             23809.523 Hz
FIDRES           0.726609 Hz
AQ              1.3762560 sec
RG                     16
DW                 21.000 usec
DE                   6.50 usec
TE                  297.5 K
D1             2.00000000 sec
D11            0.03000000 sec
TD0                     1
SFO1          100.6354036 MHz
NUC1                  13C
P0                   2.67 usec
P1                   8.00 usec
PLW1          85.25399780 W
SFO2          400.1816007 MHz
NUC2                   1H
CPDPRG[2          waltz65
PCPD2               90.00 usec
PLW2          21.26700020 W
PLW12          0.16802999 W
PLW13          0.08452000 W

F2 - Processing parameters
SI                  32768
SF            100.6253451 MHz
WDW                    EM
SSB                     0
LB                   1.00 Hz
GB                      0
PC                   1.40
```

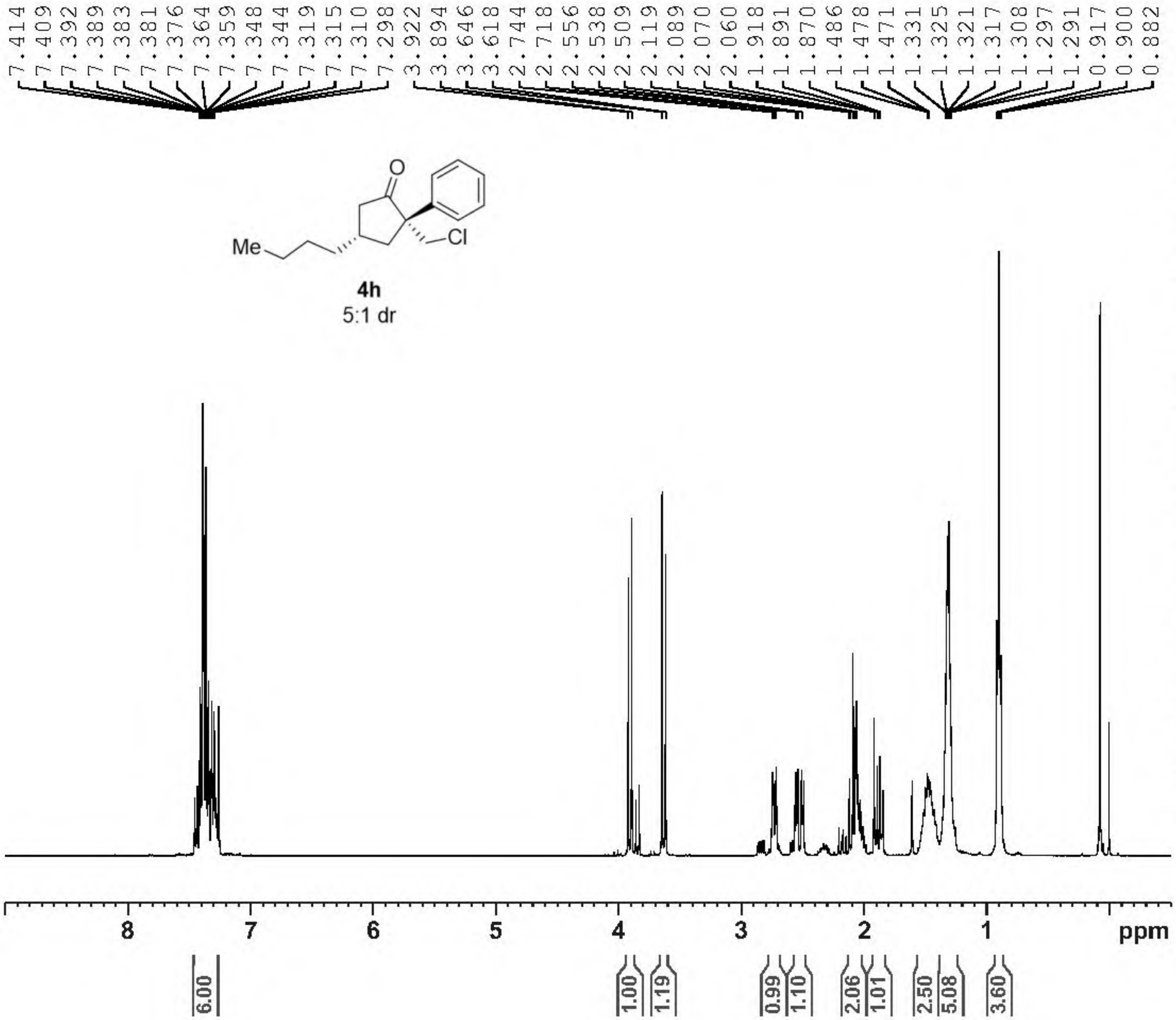


```
Current Data Parameters
NAME      zzj-4-mem-pdt-Bu-H
EXPNO                    1
PROCNO                   1

F2 - Acquisition Parameters
Date_             20250220
Time                 23.36
INSTRUM              spect
PROBHD   5 mm PABBO BB/
PULPROG               zg30
TD                   65536
SOLVENT              CDCl3
NS                      16
DS                       2
SWH               8012.820 Hz
FIDRES            0.122266 Hz
AQ               4.0894465 sec
RG                   54.81
DW                  62.400 usec
DE                    6.50 usec
TE                   296.1 K
D1              1.00000000 sec
TD0                      1

======== CHANNEL f1 ========
SFO1           400.1324710 MHz
NUC1                    1H
P1                   14.50 usec
PLW1           11.99499989 W

F2 - Processing parameters
SI                   65536
SF             400.1300097 MHz
WDW                     EM
SSB                      0
LB                    0.30 Hz
GB                       0
PC                    1.00
```

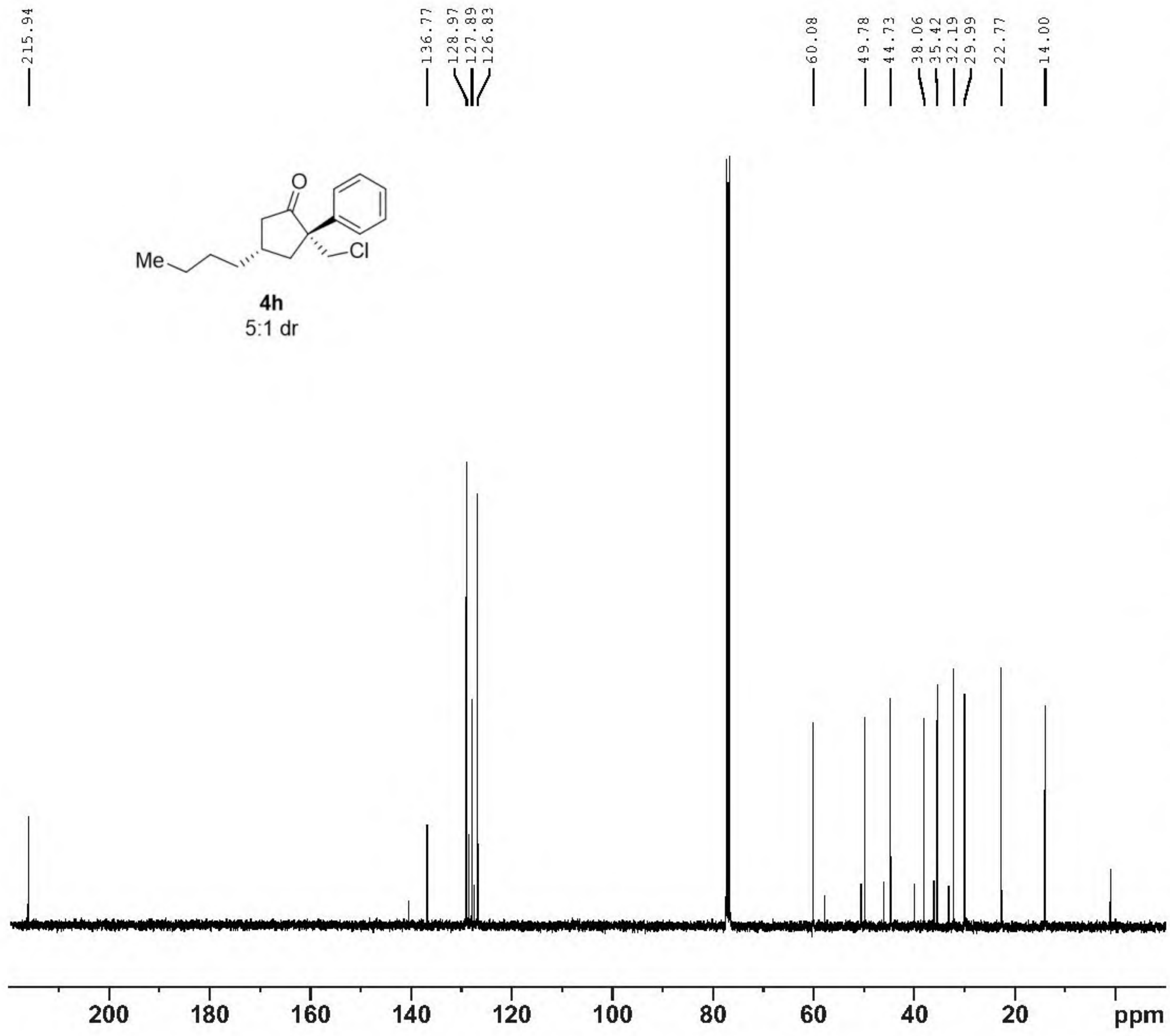


```
Current Data Parameters
NAME      zzj-4-mem-pdtt-Bu-C
EXPNO                       1
PROCNO                      1

F2 - Acquisition Parameters
Date_              20250221
Time                      0
INSTRUM               spect
PROBHD    5 mm PABBO BB/
PULPROG             zgpg30
TD                   65536
SOLVENT              CDCl3
NS                     400
DS                       2
SWH              24038.461 Hz
FIDRES            0.366798 Hz
AQ               1.3631488 sec
RG                  196.92
DW                  20.800 usec
DE                    6.50 usec
TE                   297.0 K
D1              2.00000000 sec
D11             0.03000000 sec
TD0                      1

======== CHANNEL f1 ========
SFO1         100.6228298 MHz
NUC1                   13C
P1                    9.70 usec
PLW1           46.98899841 W

======== CHANNEL f2 ========
SFO2         400.1316005 MHz
NUC2                    1H
CPDPRG[2           waltz16
PCPD2                90.00 usec
PLW2           11.99499989 W
PLW12           0.34213999 W
PLW13           0.27713001 W

F2 - Processing parameters
SI                   32768
SF             100.6127736 MHz
WDW                     EM
SSB                      0
LB                    1.00 Hz
GB                       0
PC                    1.40
```

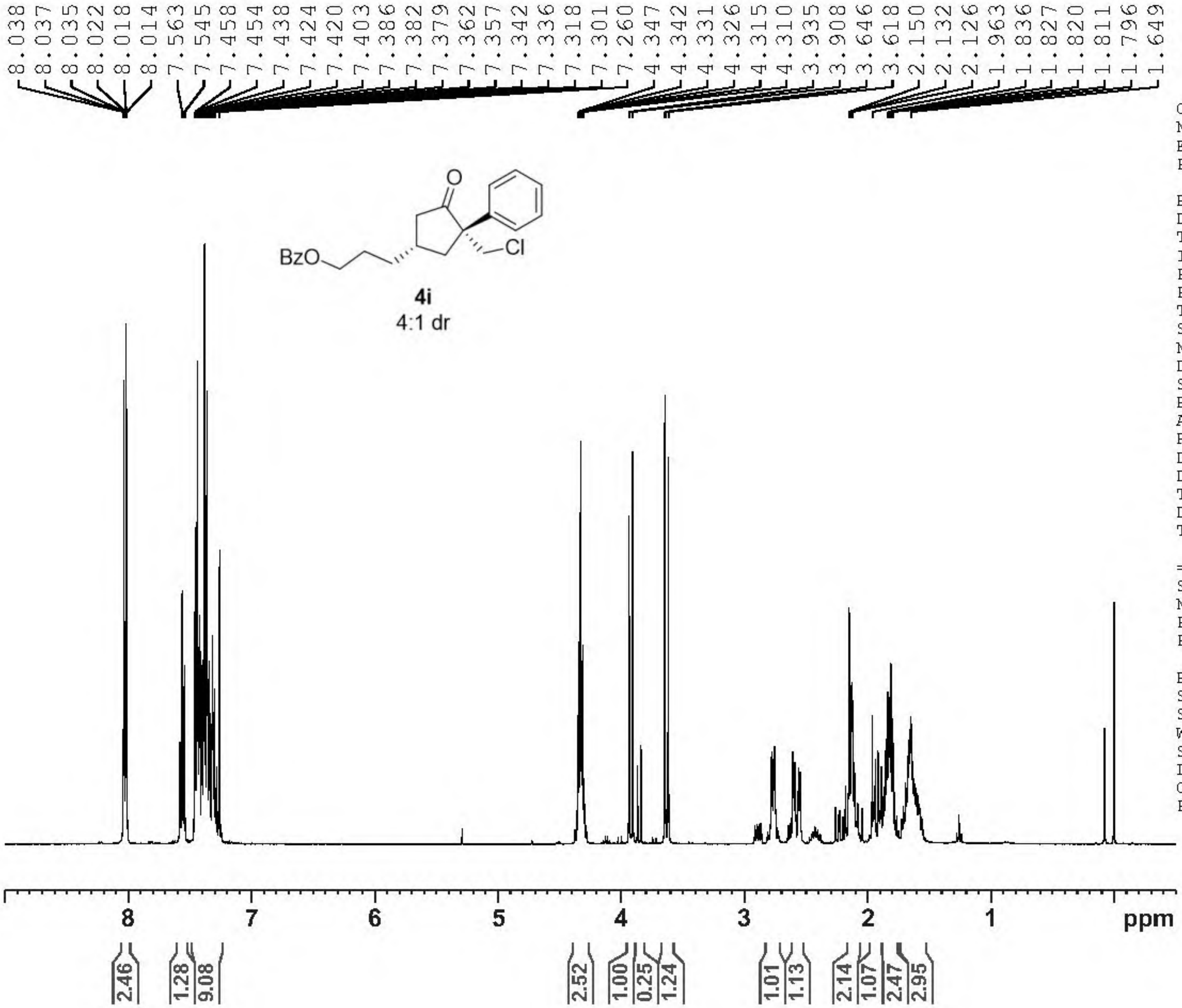

Current Data Parameters
NAME zzj-4-mem-pdt-OBz-H
EXPNO 2
PROCNO 1

F2 - Acquisition Parameters
Date_ 20260129
Time_ 22.40
INSTRUM spect
PROBHD 5 mm PABBO BB/
PULPROG zg30
TD 65536
SOLVENT CDCl3
NS 16
DS 2
SWH 8012.820 Hz
FIDRES 0.122266 Hz
AQ 4.0894465 sec
RG 54.81
DW 62.400 usec
DE 6.50 usec
TE 295.1 K
D1 1.00000000 sec
TD0 1

======== CHANNEL f1 ========
SFO1 400.1324710 MHz
NUC1 1H
P1 14.50 usec
PLW1 11.99499989 W

F2 - Processing parameters
SI 65536
SF 400.1300097 MHz
WDW EM
SSB 0
LB 0.30 Hz
GB 0
PC 1.00

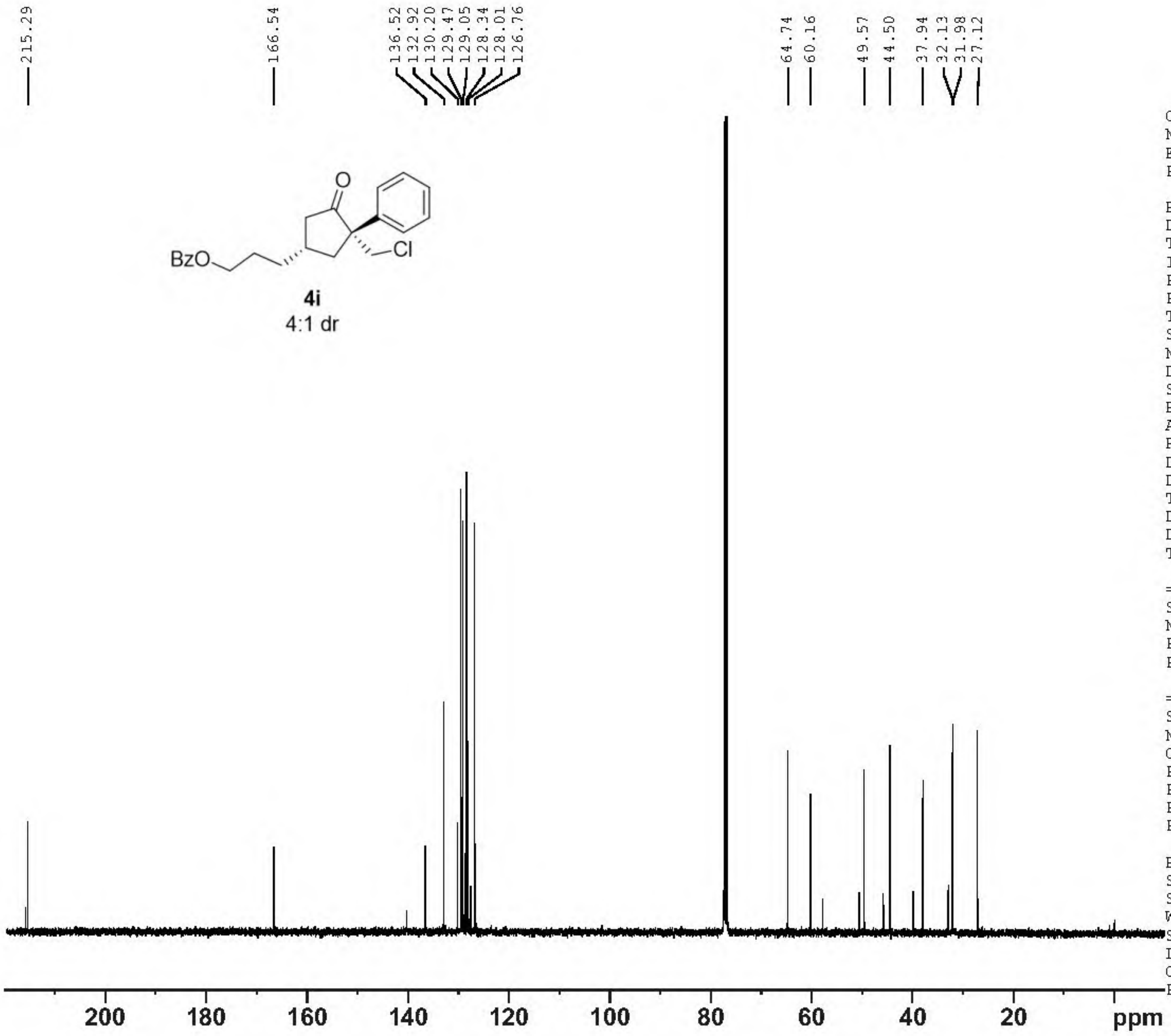

Current Data Parameters
NAME zzj-4-mem-pdt-OBz-C
EXPNO 3
PROCNO 1

F2 - Acquisition Parameters
Date_ 20260129
Time 23.07
INSTRUM spect
PROBHD 5 mm PABBO BB/
PULPROG zgpg30
TD 65536
SOLVENT CDCl3
NS 1000
DS 2
SWH 24038.461 Hz
FIDRES 0.366798 Hz
AQ 1.3631488 sec
RG 196.92
DW 20.800 usec
DE 6.50 usec
TE 296.3 K
D1 2.00000000 sec
D11 0.03000000 sec
TD0 1

======== CHANNEL f1 ========
SFO1 100.6228298 MHz
NUC1 13C
P1 9.70 usec
PLW1 46.98899841 W

======== CHANNEL f2 ========
SFO2 400.1316005 MHz
NUC2 1H
CPDPRG[2 waltz16
PCPD2 90.00 usec
PLW2 11.99499989 W
PLW12 0.34213999 W
PLW13 0.27713001 W

F2 - Processing parameters
SI 32768
SF 100.6127753 MHz
WDW EM
SSB 0
LB 1.00 Hz
GB 0
PC 1.40

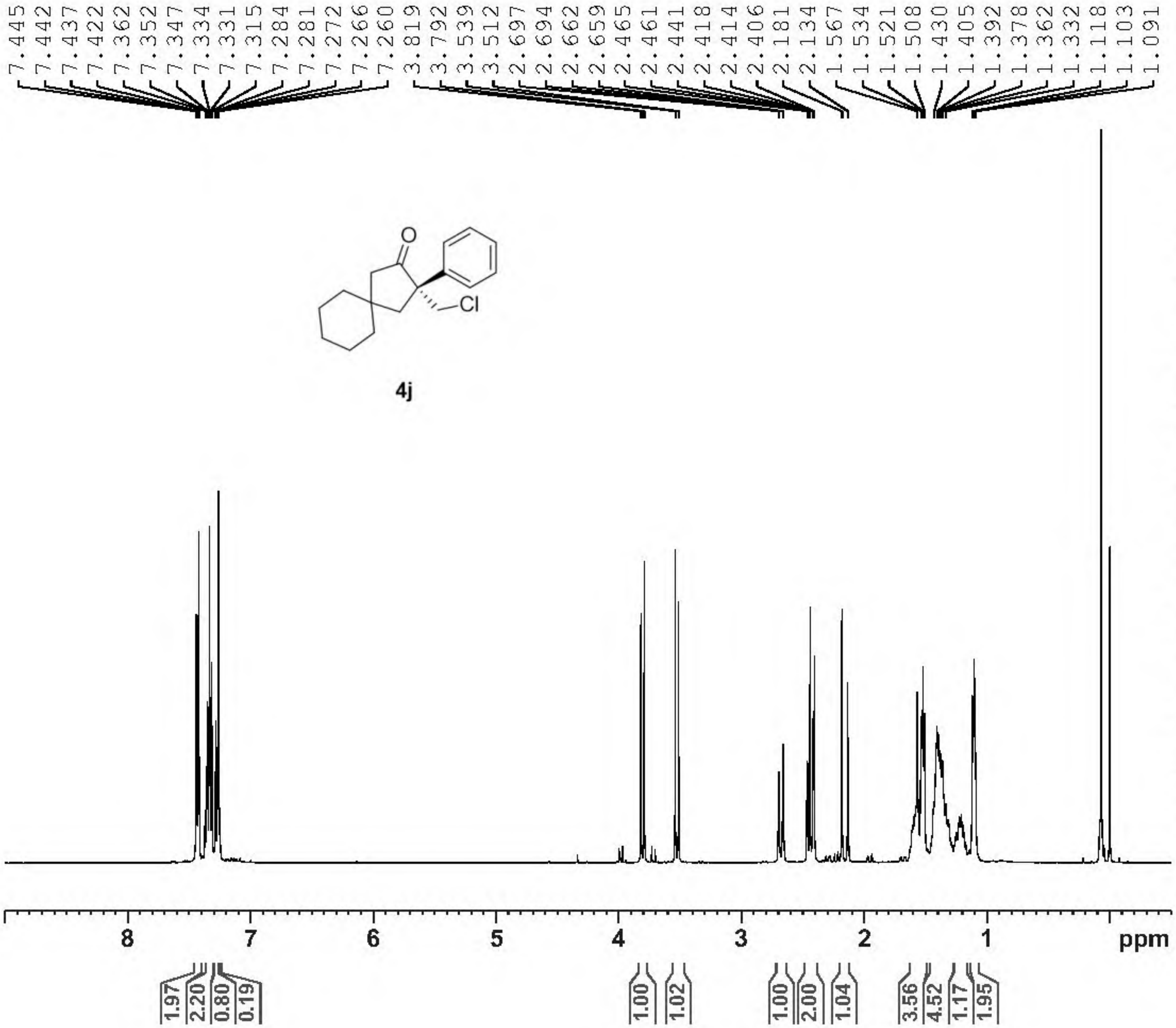

Current Data Parameters
NAME zzj-4-mem-pdt-Cy-H
EXPNO 1
PROCNO 1

F2 - Acquisition Parameters
Date_ 20250228
Time 11.03
INSTRUM spect
PROBHD 5 mm PABBO BB/
PULPROG zg30
TD 65536
SOLVENT CDCl3
NS 16
DS 2
SWH 8012.820 Hz
FIDRES 0.122266 Hz
AQ 4.0894465 sec
RG 126.97
DW 62.400 usec
DE 6.50 usec
TE 295.8 K
D1 1.00000000 sec
TD0 1

======== CHANNEL f1 ========
SFO1 400.1324710 MHz
NUC1 1H
P1 14.50 usec
PLW1 11.99499989 W

F2 - Processing parameters
SI 65536
SF 400.1300098 MHz
WDW EM
SSB 0
LB 0.30 Hz
GB 0
PC 1.00

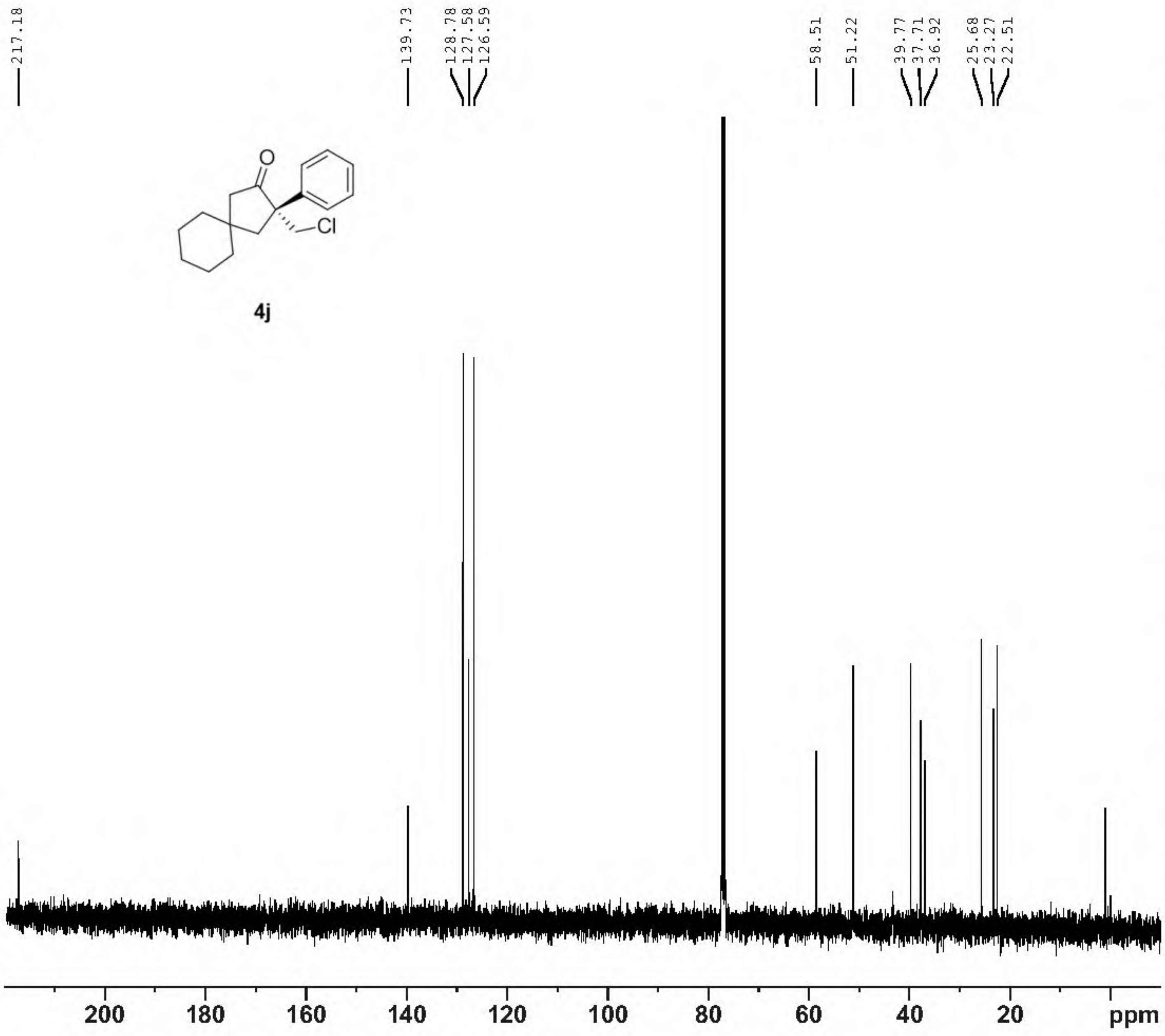


```
Current Data Parameters
NAME      zzj-4-mem-pdt-Cy-C
EXPNO                    1
PROCNO                   1

F2 - Acquisition Parameters
Date_             20250228
Time                 11.05
INSTRUM              spect
PROBHD   5 mm PABBO BB/
PULPROG            zgpg30
TD                   65536
SOLVENT              CDCl3
NS                     200
DS                       2
SWH           24038.461 Hz
FIDRES         0.366798 Hz
AQ            1.3631488 sec
RG                  196.92
DW              20.800 usec
DE                6.50 usec
TE               296.0 K
D1          2.00000000 sec
D11         0.03000000 sec
TD0                      1

======== CHANNEL f1 ========
SFO1       100.6228298 MHz
NUC1                   13C
P1                9.70 usec
PLW1       46.98899841 W

======== CHANNEL f2 ========
SFO2       400.1316005 MHz
NUC2                    1H
CPDPRG[2           waltz16
PCPD2            90.00 usec
PLW2       11.99499989 W
PLW12       0.34213999 W
PLW13       0.27713001 W

F2 - Processing parameters
SI                   32768
SF         100.6127716 MHz
WDW                     EM
SSB                      0
LB                1.00 Hz
GB                       0
PC                    1.40
```

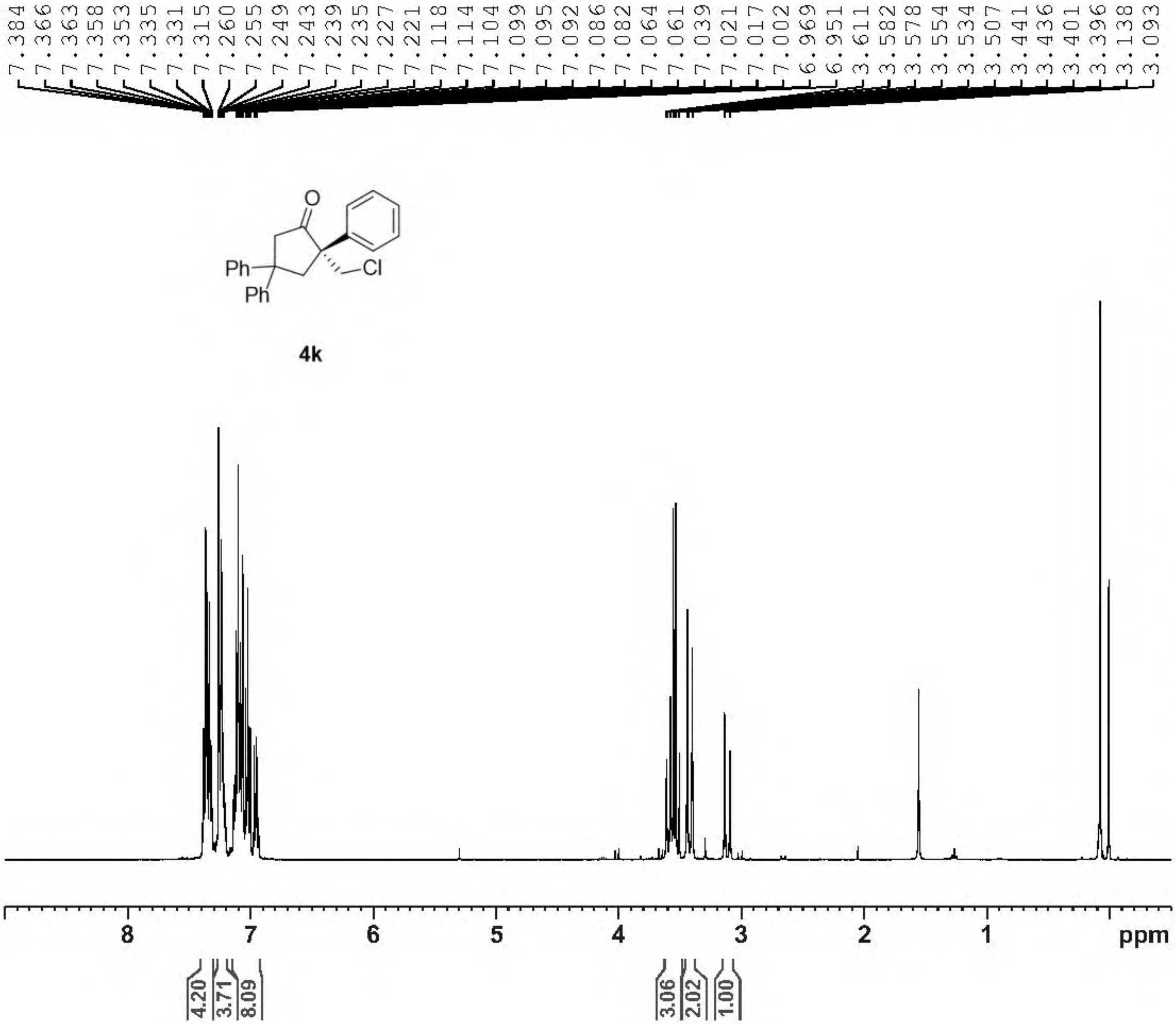

Current Data Parameters
NAME pdt-4-mem-gem-Ph-H
EXPNO 1
PROCNO 1

F2 - Acquisition Parameters
Date_ 20260618
Time 18.09 h
INSTRUM AvanceNeo 400MHz
PROBHD Z163739_0629 (
PULPROG zg30
TD 65536
SOLVENT CDCl3
NS 16
DS 2
SWH 8196.722 Hz
FIDRES 0.250144 Hz
AQ 3.9976959 sec
RG 101
DW 61.000 usec
DE 13.89 usec
TE 297.1 K
D1 1.00000000 sec
TD0 1
SFO1 400.1824711 MHz
NUC1 1H
P0 2.67 usec
P1 8.00 usec
PLW1 21.26700020 W

F2 - Processing parameters
SI 65536
SF 400.1800095 MHz
WDW EM
SSB 0
LB 0.30 Hz
GB 0
PC 1.00

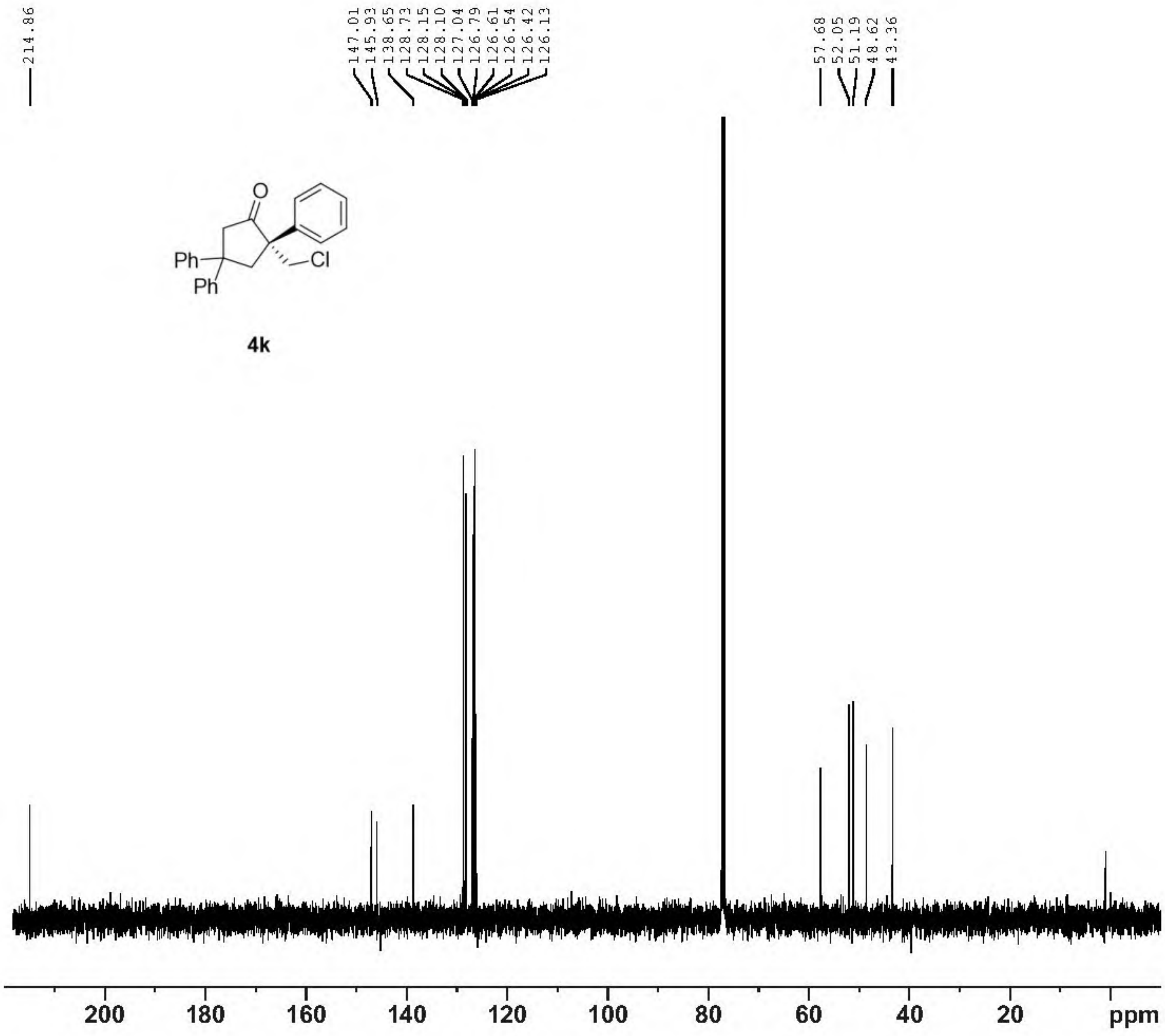


```
Current Data Parameters
NAME       pdt-4-mem-gem-Ph-C
EXPNO                       3
PROCNO                      1

F2 - Acquisition Parameters
Date_                20260618
Time                    18.21 h
INSTRUM    AvanceNeo 400MHz
PROBHD     Z163739_0629 (
PULPROG               zgpg30
TD                      65536
SOLVENT                 CDCl3
NS                        193
DS                          4
SWH                 23809.523 Hz
FIDRES               0.726609 Hz
AQ                  1.3762560 sec
RG                         16
DW                     21.000 usec
DE                       6.50 usec
TE                      297.7 K
D1                 2.00000000 sec
D11                0.03000000 sec
TD0                         1
SFO1              100.6354036 MHz
NUC1                      13C
P0                       2.67 usec
P1                       8.00 usec
PLW1              85.25399780 W
SFO2              400.1816007 MHz
NUC2                       1H
CPDPRG[2              waltz65
PCPD2                   90.00 usec
PLW2              21.26700020 W
PLW12              0.16802999 W
PLW13              0.08452000 W

F2 - Processing parameters
SI                      32768
SF                100.6253451 MHz
WDW                        EM
SSB                         0
LB                       1.00 Hz
GB                          0
PC                       1.40
```

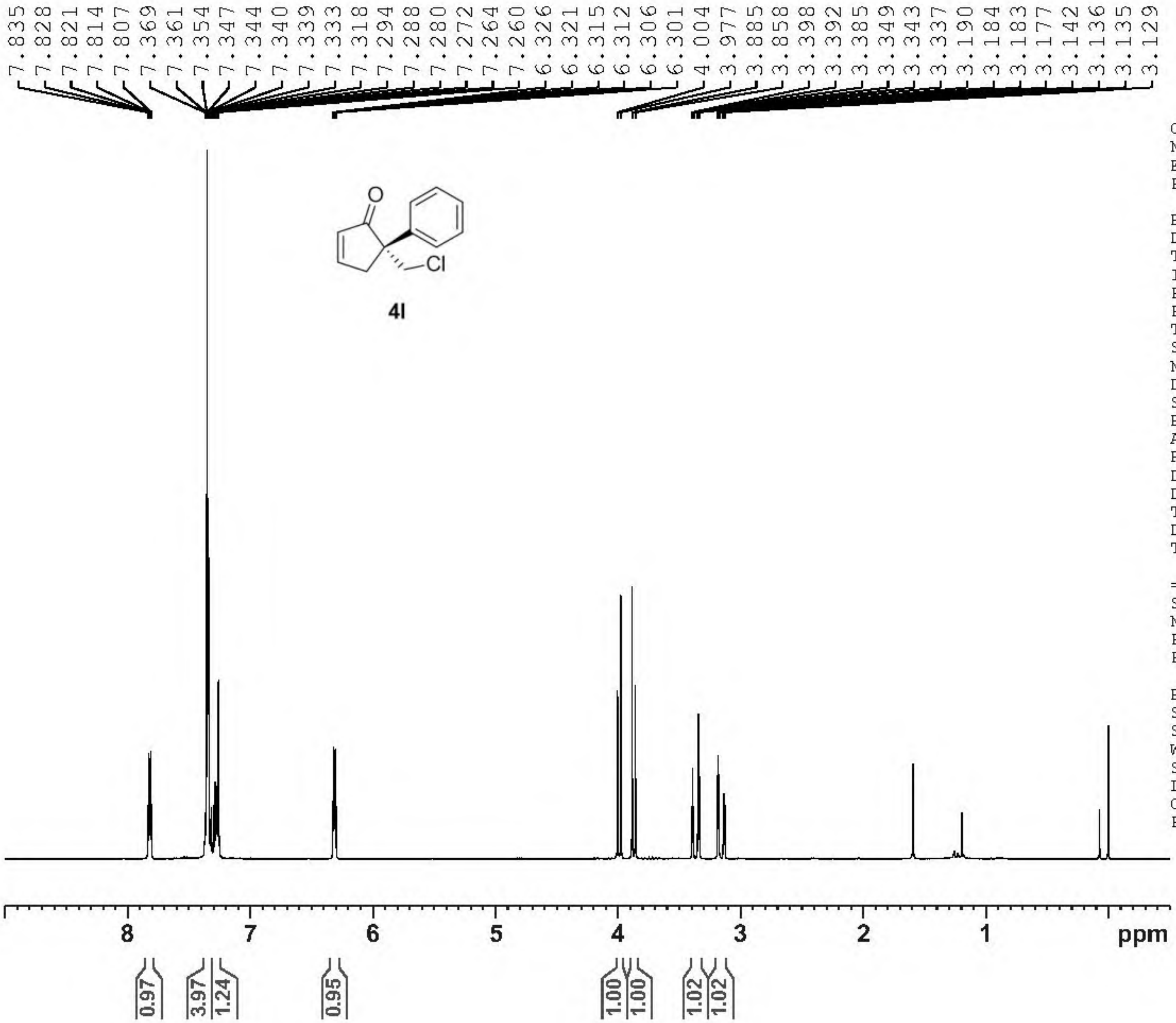


```
Current Data Parameters
NAME       zzj-4-mem-Et3N-Ph-H
EXPNO                    1
PROCNO                   1

F2 - Acquisition Parameters
Date_             20250116
Time                  0.09
INSTRUM              spect
PROBHD    5 mm PABBO BB/
PULPROG               zg30
TD                   65536
SOLVENT              CDCl3
NS                      16
DS                       2
SWH               8012.820 Hz
FIDRES            0.122266 Hz
AQ               4.0894465 sec
RG                  103.52
DW                  62.400 usec
DE                    6.50 usec
TE                   295.9 K
D1              1.00000000 sec
TD0                      1

======== CHANNEL f1 ========
SFO1        400.1324710 MHz
NUC1                    1H
P1                   14.50 usec
PLW1           11.99499989 W

F2 - Processing parameters
SI                   65536
SF          400.1300097 MHz
WDW                     EM
SSB                      0
LB                    0.30 Hz
GB                       0
PC                    1.00
```

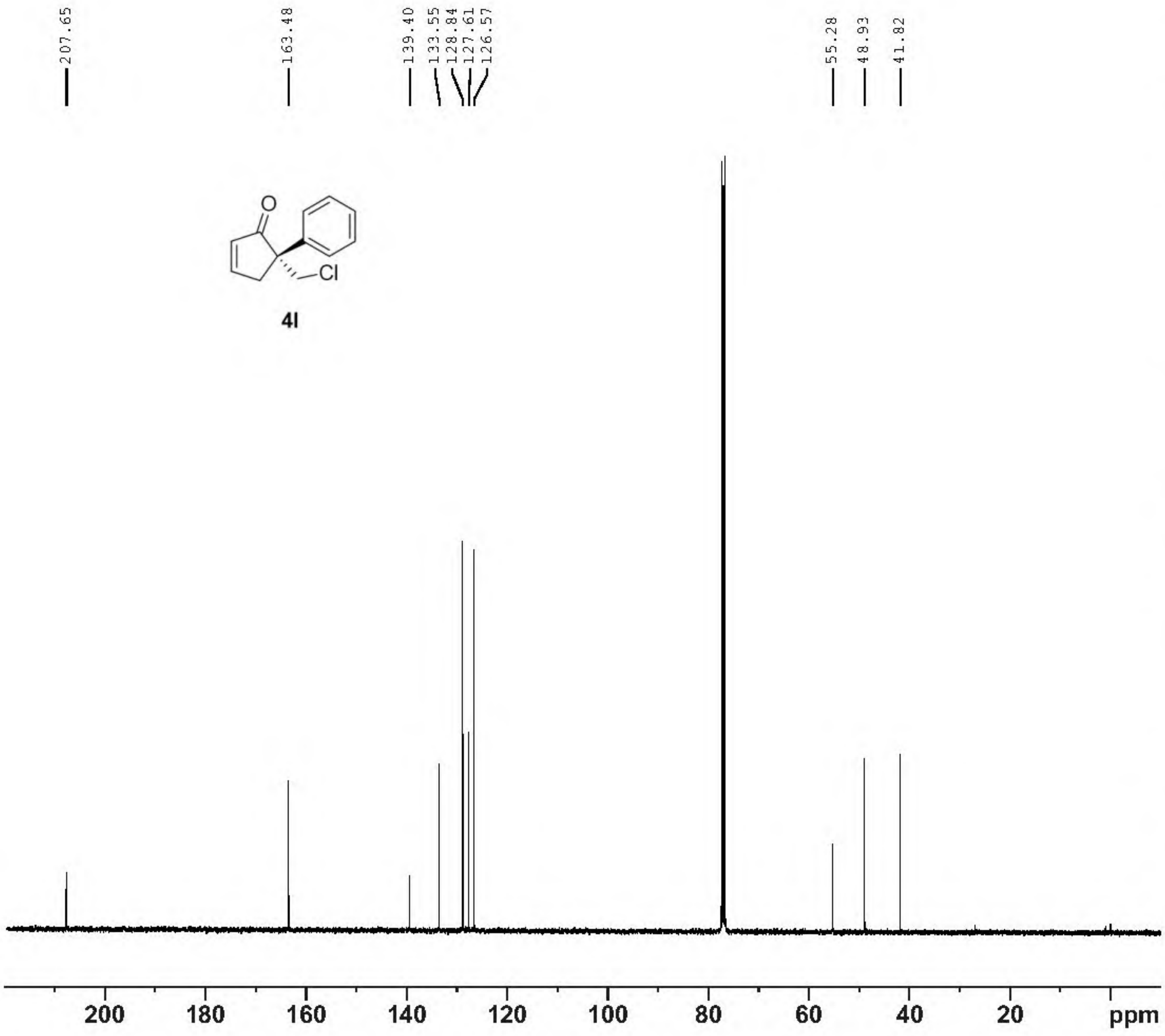

Current Data Parameters
NAME zzj-4-mem-Et3N-Ph-C
EXPNO 1
PROCNO 1

F2 - Acquisition Parameters
Date_ 20250116
Time 1.08
INSTRUM spect
PROBHD 5 mm PABBO BB/
PULPROG zgpg30
TD 65536
SOLVENT CDCl3
NS 1000
DS 2
SWH 24038.461 Hz
FIDRES 0.366798 Hz
AQ 1.3631488 sec
RG 196.92
DW 20.800 usec
DE 6.50 usec
TE 296.9 K
D1 2.00000000 sec
D11 0.03000000 sec
TD0 1

======== CHANNEL f1 ========
SFO1 100.6228298 MHz
NUC1 13C
P1 9.70 usec
PLW1 46.98899841 W

======== CHANNEL f2 ========
SFO2 400.1316005 MHz
NUC2 1H
CPDPRG[2 waltz16
PCPD2 90.00 usec
PLW2 11.99499989 W
PLW12 0.34213999 W
PLW13 0.27713001 W

F2 - Processing parameters
SI 32768
SF 100.6127731 MHz
WDW EM
SSB 0
LB 1.00 Hz
GB 0
PC 1.40

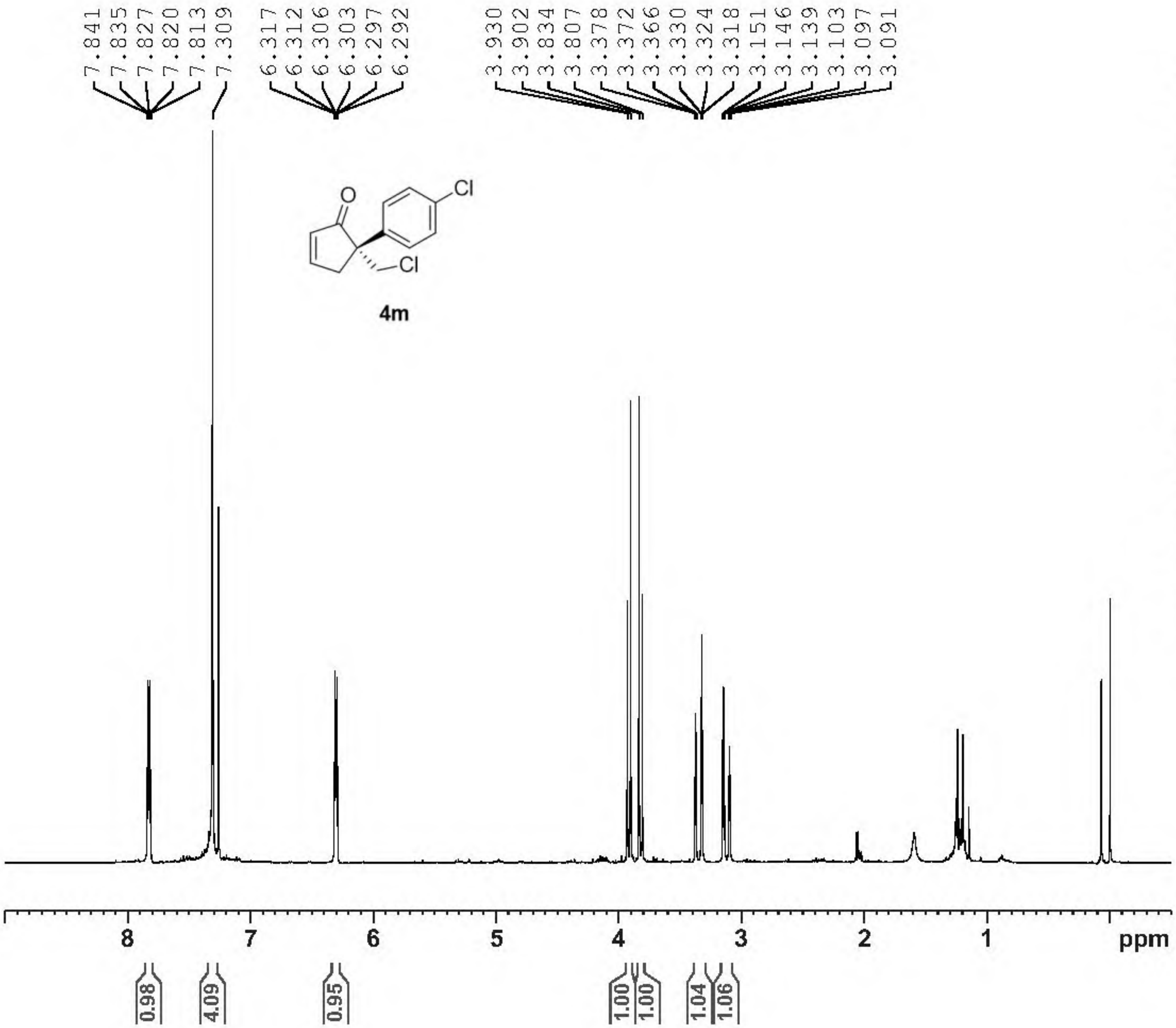


```
Current Data Parameters
NAME      zzj-4-mem-pdt-Et3N-pClPh-H
EXPNO                    1
PROCNO                   1

F2 - Acquisition Parameters
Date_             20250118
Time                 10.48
INSTRUM              spect
PROBHD   5 mm PABBO BB/
PULPROG               zg30
TD                   65536
SOLVENT              CDCl3
NS                      16
DS                       2
SWH              8012.820 Hz
FIDRES           0.122266 Hz
AQ              4.0894465 sec
RG                  112.31
DW                  62.400 usec
DE                    6.50 usec
TE                   294.6 K
D1             1.00000000 sec
TD0                      1

======== CHANNEL f1 ========
SFO1          400.1324710 MHz
NUC1                    1H
P1                   14.50 usec
PLW1           11.99499989 W

F2 - Processing parameters
SI                   65536
SF            400.1300098 MHz
WDW                     EM
SSB                      0
LB                    0.30 Hz
GB                       0
PC                    1.00
```

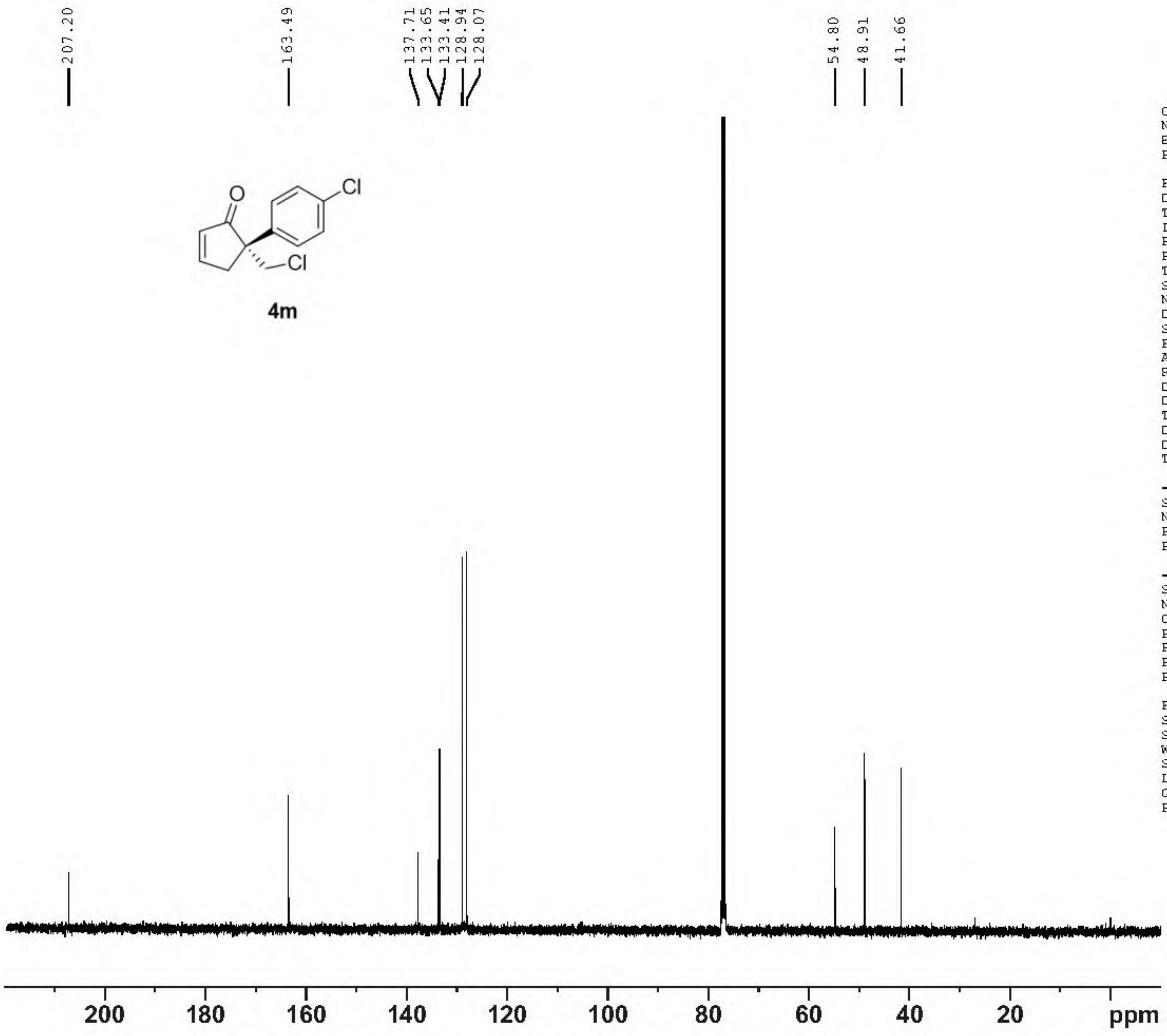


```
Current Data Parameters
NAME      zzj-4-mem-pdt-Et3N-pClPh-C
EXPNO                1
PROCNO               1

F2 - Acquisition Parameters
Date_           20250118
Time               11.18
INSTRUM            spect
PROBHD   5 mm PABBO BB/
PULPROG          zgpg30
TD                 65536
SOLVENT            CDCl3
NS                   500
DS                     2
SWH            24038.461 Hz
FIDRES          0.366798 Hz
AQ             1.3631488 sec
RG                196.92
DW                20.800 usec
DE                  6.50 usec
TE                 295.9 K
D1            2.00000000 sec
D11           0.03000000 sec
TD0                    1

-------- CHANNEL f1 --------
SFO1         100.6228298 MHz
NUC1                 13C
P1                  9.70 usec
PLW1         46.98899841 W

-------- CHANNEL f2 --------
SFO2         400.1316005 MHz
NUC2                  1H
CPDPRG[2         waltz16
PCPD2              90.00 usec
PLW2         11.99499989 W
PLW12         0.34213999 W
PLW13         0.27713001 W

F2 - Processing parameters
SI                 32768
SF           100.6127728 MHz
WDW                   EM
SSB                    0
LB                  1.00 Hz
GB                     0
PC                  1.40
```

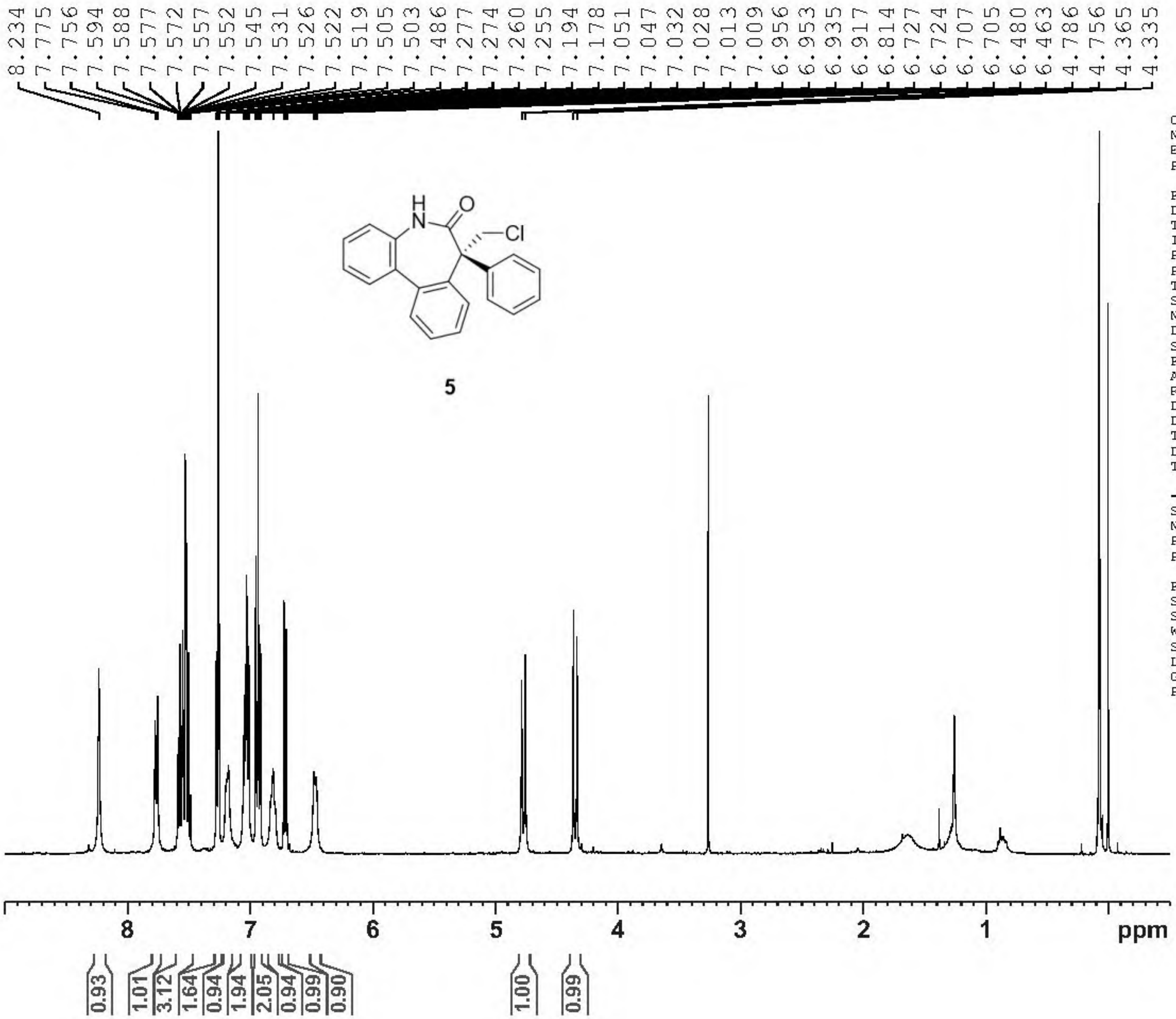

Current Data Parameters
NAME transformation-beckmann-H
EXPNO 1
PROCNO 1

F2 - Acquisition Parameters
Date_ 20241211
Time_ 22.56
INSTRUM spect
PROBHD 5 mm PABBO BB/
PULPROG zg30
TD 65536
SOLVENT CDCl3
NS 16
DS 2
SWH 8012.820 Hz
FIDRES 0.122266 Hz
AQ 4.0894465 sec
RG 112.31
DW 62.400 usec
DE 6.50 usec
TE 296.1 K
D1 1.00000000 sec
TD0 1

-------- CHANNEL f1 --------
SFO1 400.1324710 MHz
NUC1 1H
P1 14.50 usec
PLW1 11.99499989 W

F2 - Processing parameters
SI 65536
SF 400.1300098 MHz
WDW EM
SSB 0
LB 0.30 Hz
GB 0
PC 1.00

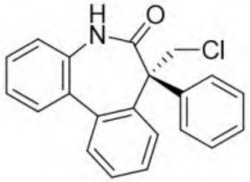

5

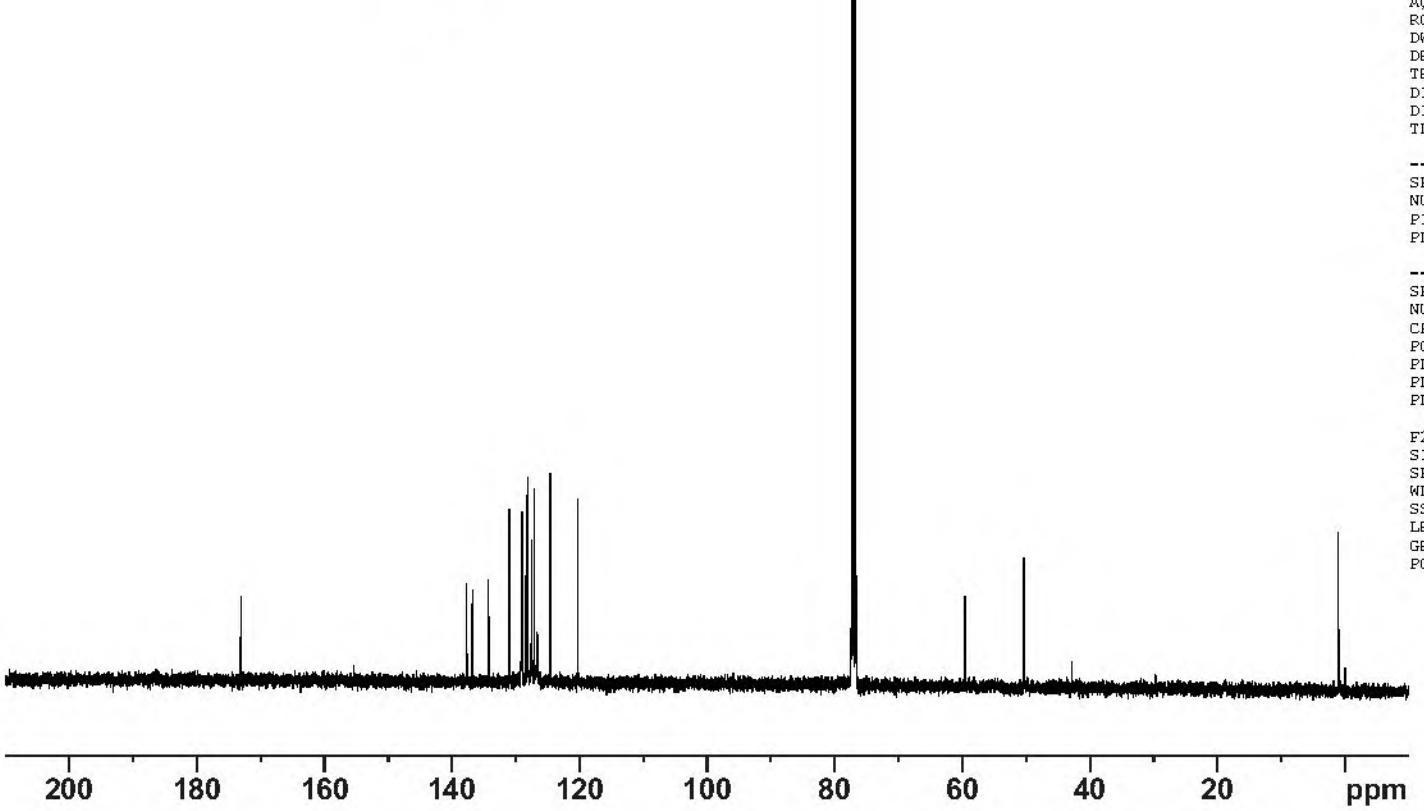


```
Current Data Parameters
NAME      transformation-beckmann-C
EXPNO                  1
PROCNO                 1

F2 - Acquisition Parameters
Date_          20241211
Time              23.54
INSTRUM           spect
PROBHD   5 mm PABBO BB/
PULPROG         zgpg30
TD               65536
SOLVENT           CDCl3
NS                1000
DS                   2
SWH          24038.461 Hz
FIDRES        0.366798 Hz
AQ           1.3631488 sec
RG              196.92
DW              20.800 usec
DE                6.50 usec
TE               297.4 K
D1          2.00000000 sec
D11         0.03000000 sec
TD0                  1

-------- CHANNEL f1 --------
SFO1       100.6228298 MHz
NUC1               13C
P1                9.70 usec
PLW1       46.98899841 W

-------- CHANNEL f2 --------
SFO2       400.1316005 MHz
NUC2                1H
CPDPRG[2       waltz16
PCPD2            90.00 usec
PLW2       11.99499989 W
PLW12       0.34213999 W
PLW13       0.27713001 W

F2 - Processing parameters
SI               32768
SF         100.6127716 MHz
WDW                 EM
SSB                  0
LB                1.00 Hz
GB                   0
PC                1.40
```

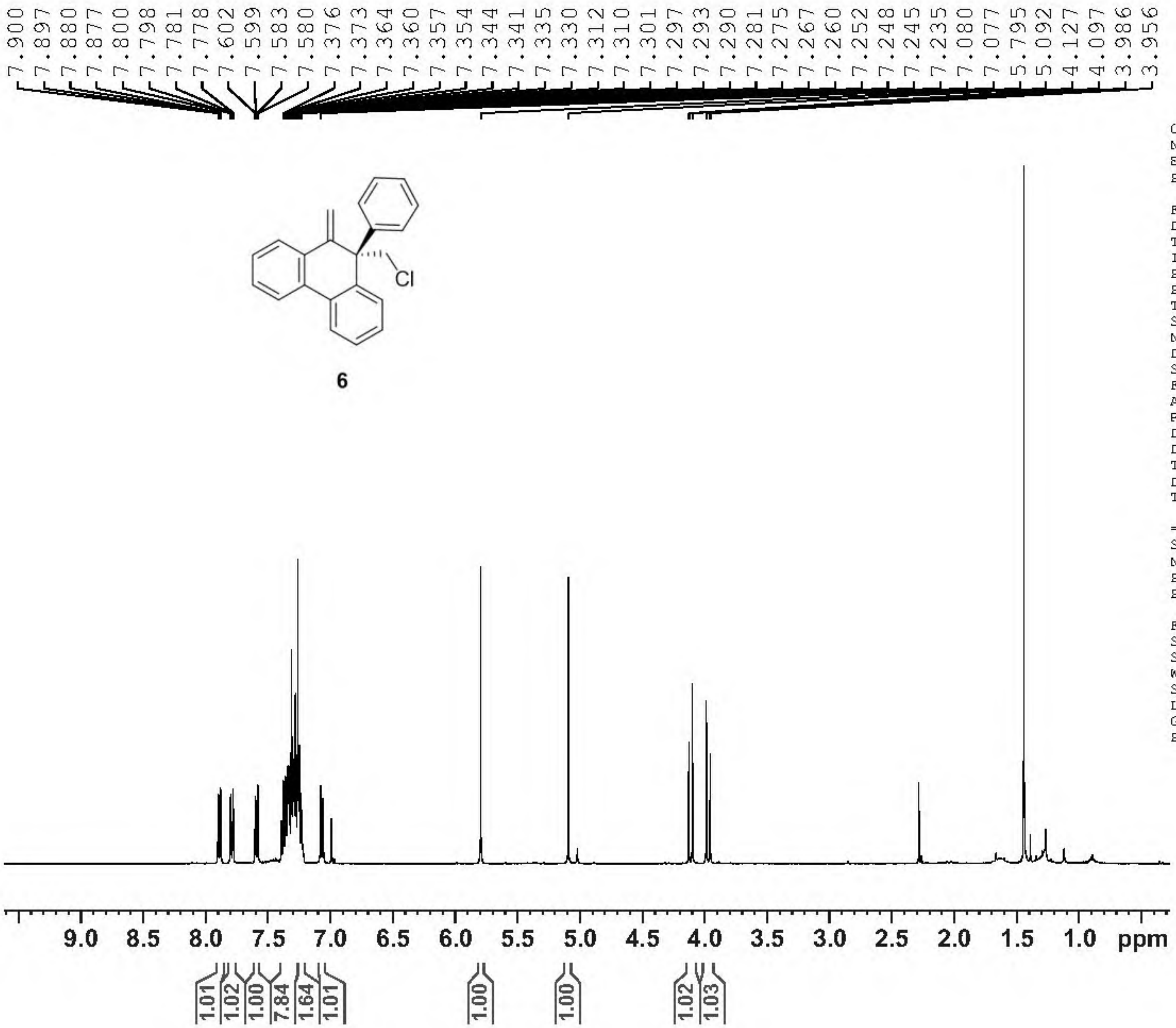


```
Current Data Parameters
NAME     transformation wittig-H
EXPNO                  1
PROCNO                 1

F2 - Acquisition Parameters
Date_           20241106
Time               10.19
INSTRUM            spect
PROBHD   5 mm PABBO BB/
PULPROG             zg30
TD                 65536
SOLVENT            CDCl3
NS                    11
DS                     2
SWH             8012.820 Hz
FIDRES          0.122266 Hz
AQ             4.0894465 sec
RG                103.52
DW                62.400 usec
DE                  6.50 usec
TE                 296.1 K
D1            1.00000000 sec
TD0                    1

======== CHANNEL f1 ========
SFO1        400.1324710 MHz
NUC1                  1H
P1                 14.50 usec
PLW1         11.99499989 W

F2 - Processing parameters
SI                 65536
SF           400.1300099 MHz
WDW                   EM
SSB                    0
LB                  0.30 Hz
GB                     0
PC                  1.00
```

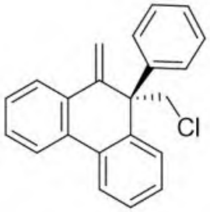

6

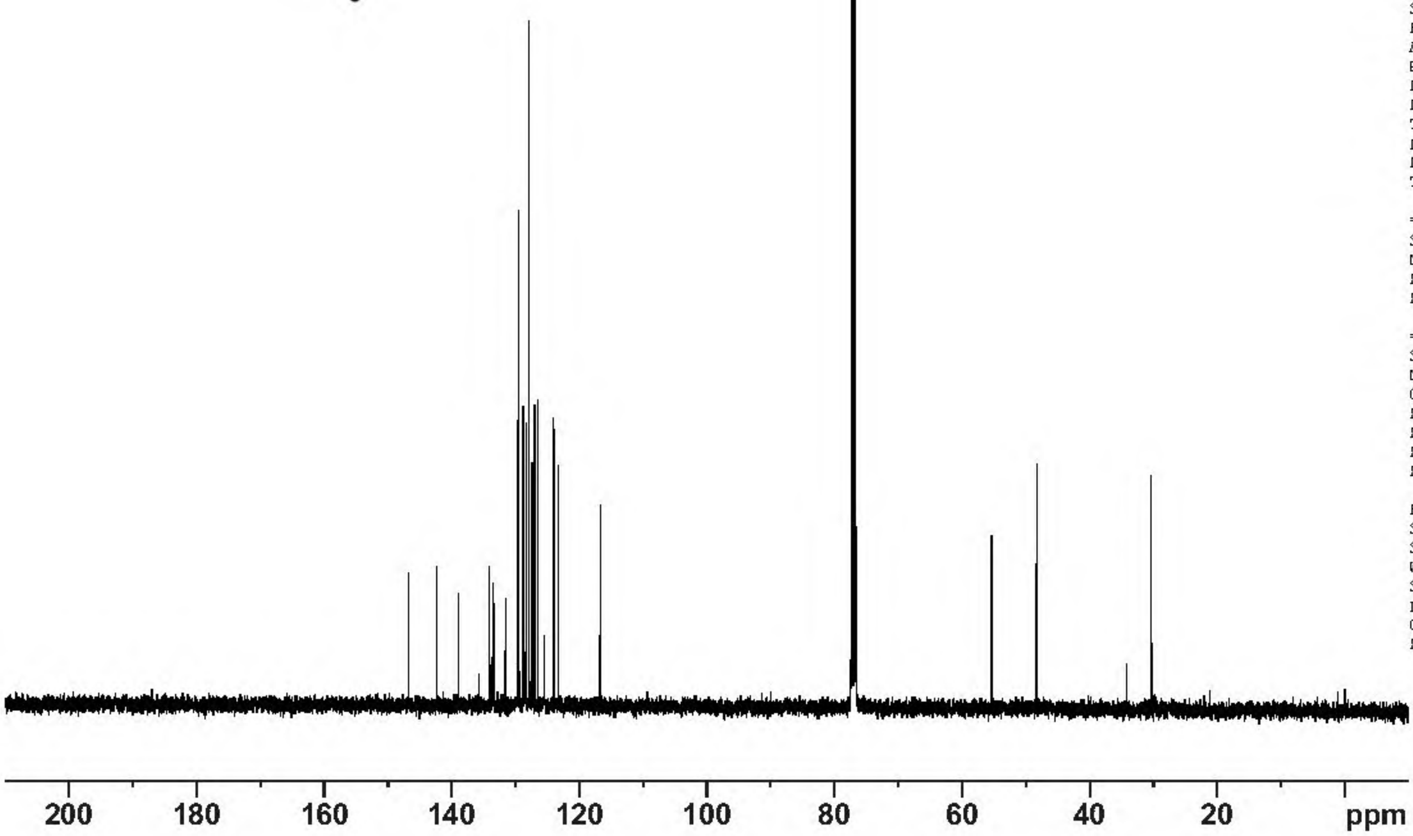


```
Current Data Parameters
NAME      transformation wittig-C
EXPNO                   1
PROCNO                  1

F2 - Acquisition Parameters
Date_            20241106
Time                10.22
INSTRUM             spect
PROBHD   5 mm PABBO BB/
PULPROG           zgpg30
TD                 65536
SOLVENT            CDCl3
NS                   800
DS                     2
SWH            24038.461 Hz
FIDRES          0.366798 Hz
AQ             1.3631488 sec
RG                196.92
DW                20.800 usec
DE                  6.50 usec
TE                 296.7 K
D1            2.00000000 sec
D11           0.03000000 sec
TD0                    1

======== CHANNEL f1 ========
SFO1         100.6228298 MHz
NUC1                 13C
P1                  9.70 usec
PLW1         46.98899841 W

======== CHANNEL f2 ========
SFO2         400.1316005 MHz
NUC2                  1H
CPDPRG[2         waltz16
PCPD2              90.00 usec
PLW2         11.99499989 W
PLW12         0.34213999 W
PLW13         0.27713001 W

F2 - Processing parameters
SI                 32768
SF           100.6127724 MHz
WDW                   EM
SSB                    0
LB                  1.00 Hz
GB                     0
PC                  1.40
```

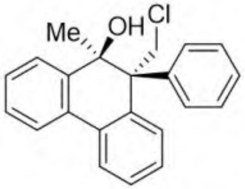

7

10:1 dr

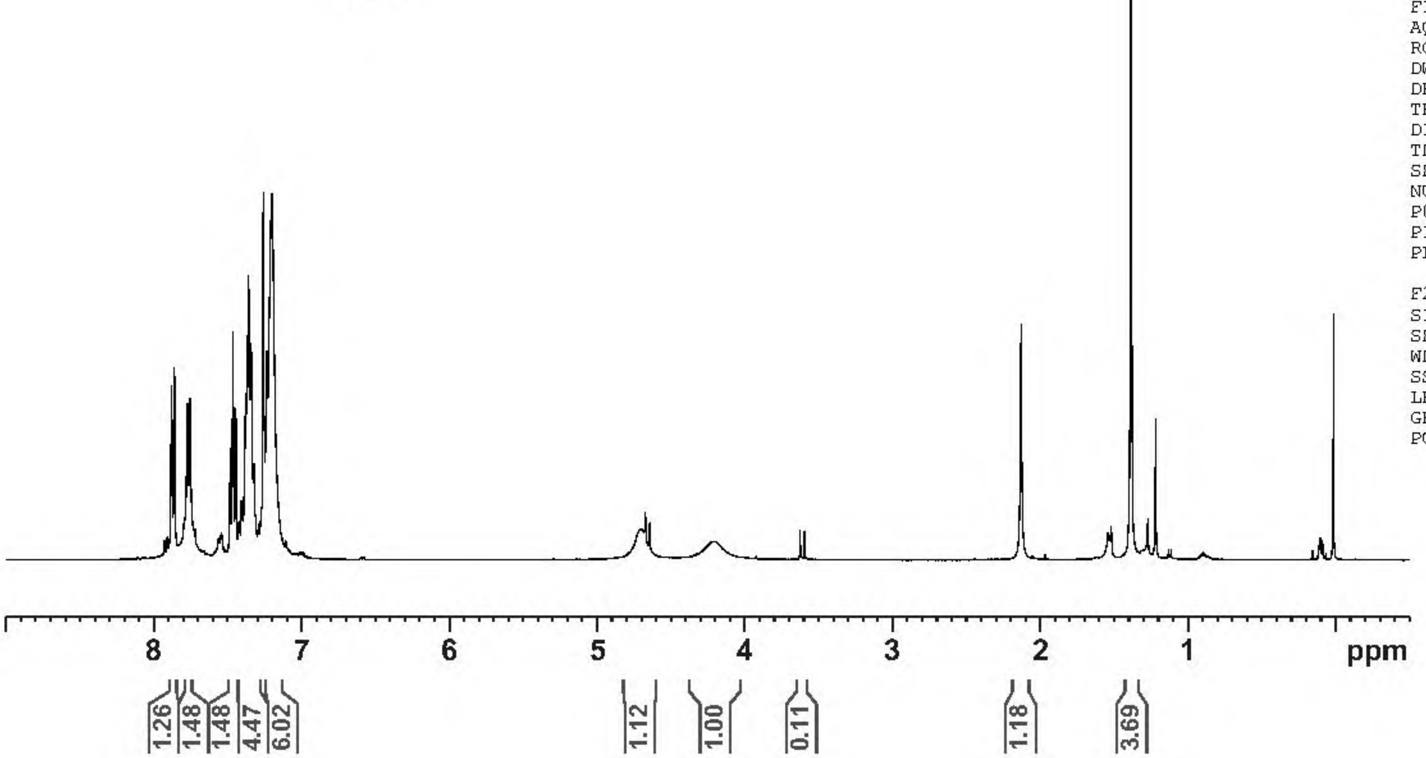

Current Data Parameters
NAME transformation-MeLi-H
EXPNO 1
PROCNO 1

F2 - Acquisition Parameters
Date_ 20241218
Time_ 10.14 h
INSTRUM AvanceNeo 400MHz
PROBHD Z163739_0629 (
PULPROG zg30
TD 65536
SOLVENT CDCl3
NS 16
DS 2
SWH 8196.722 Hz
FIDRES 0.250144 Hz
AQ 3.9976959 sec
RG 101
DW 61.000 usec
DE 13.89 usec
TE 297.0 K
D1 1.00000000 sec
TD0 1
SFO1 400.1824711 MHz
NUC1 1H
P0 2.67 usec
P1 8.00 usec
PLW1 21.26700020 W

F2 - Processing parameters
SI 65536
SF 400.1800098 MHz
WDW EM
SSB 0
LB 0.30 Hz
GB 0
PC 1.00

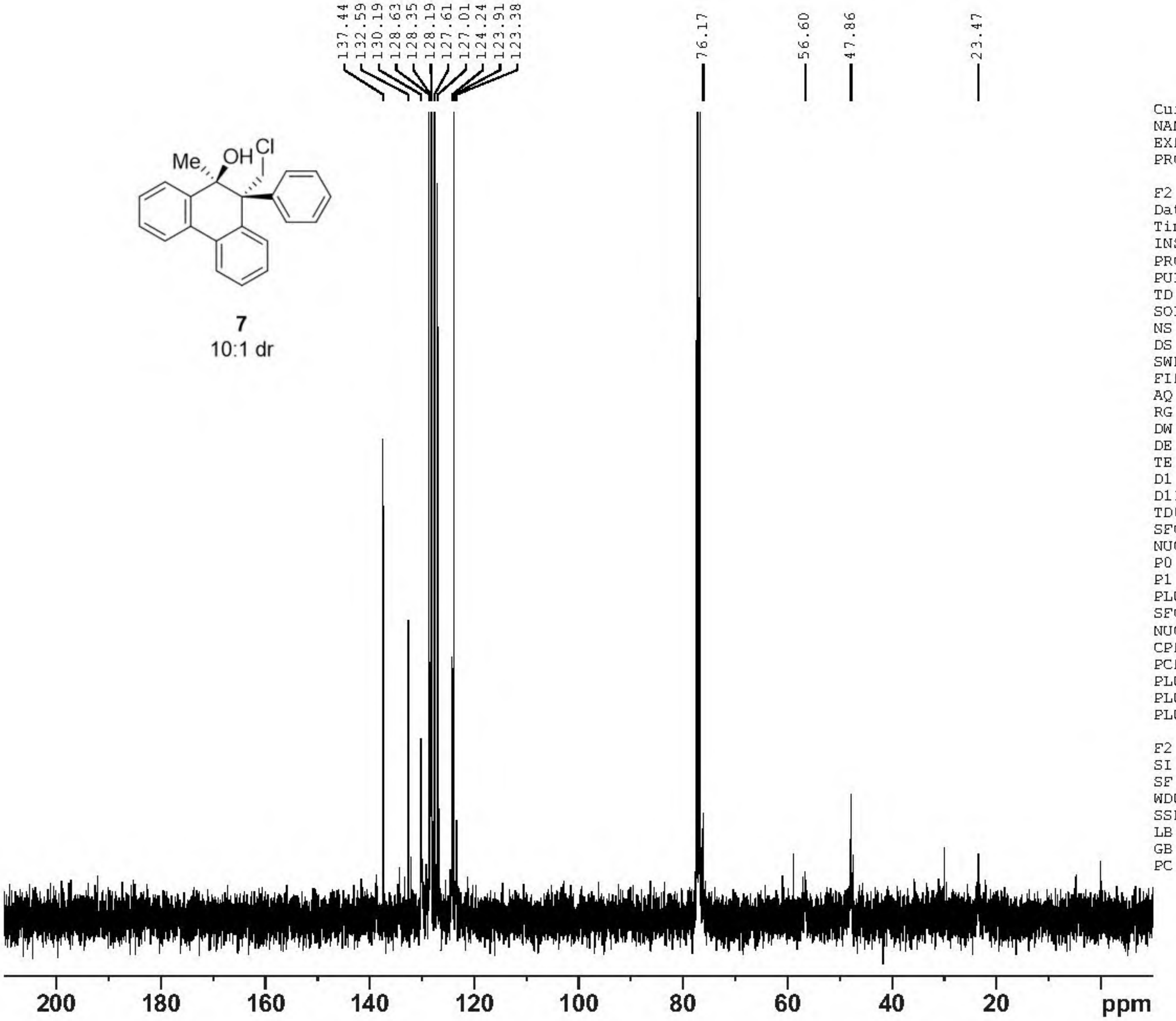

7
10:1 dr

Current Data Parameters
NAME transformation-MeLi-C
EXPNO 3
PROCNO 1

F2 - Acquisition Parameters
Date_ 20241218
Time_ 10.38 h
INSTRUM AvanceNeo 400MHz
PROBHD Z163739_0629 (
PULPROG zgpg30
TD 65536
SOLVENT CDCl3
NS 400
DS 4
SWH 23809.523 Hz
FIDRES 0.726609 Hz
AQ 1.3762560 sec
RG 10
DW 21.000 usec
DE 6.50 usec
TE 297.4 K
D1 2.00000000 sec
D11 0.03000000 sec
TD0 1
SFO1 100.6354036 MHz
NUC1 13C
P0 2.67 usec
P1 8.00 usec
PLW1 85.25399780 W
SFO2 400.1816007 MHz
NUC2 1H
CPDPRG[2 waltz65
PCPD2 90.00 usec
PLW2 21.26700020 W
PLW12 0.16802999 W
PLW13 0.08452000 W

F2 - Processing parameters
SI 32768
SF 100.6253410 MHz
WDW EM
SSB 0
LB 1.00 Hz
GB 0
PC 1.40

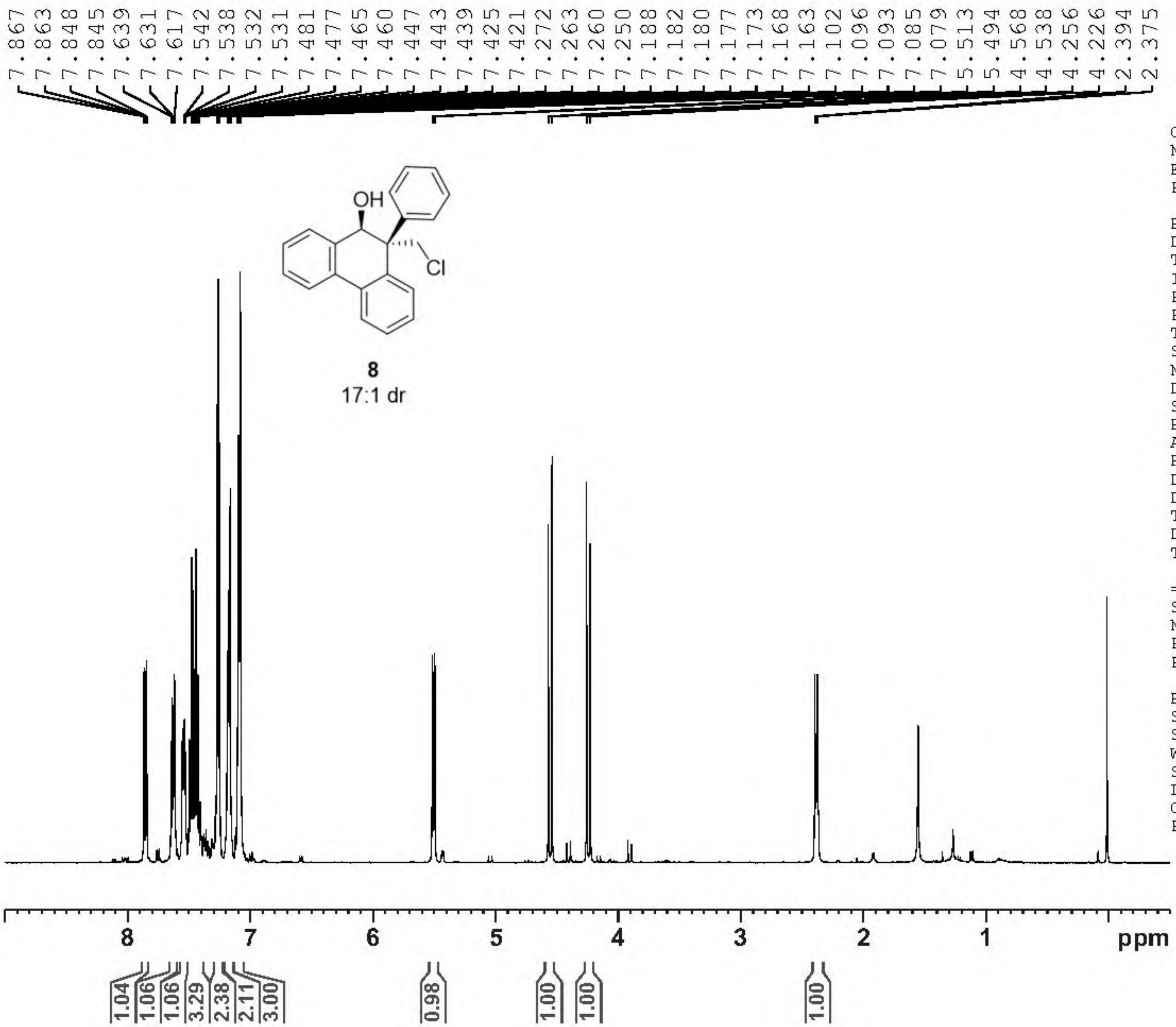


```
Current Data Parameters
NAME          zzj-NaBH4-H
EXPNO                   1
PROCNO                  1

F2 - Acquisition Parameters
Date_            20241018
Time                11.21
INSTRUM             spect
PROBHD   5 mm PABBO BB/
PULPROG              zg30
TD                  65536
SOLVENT             CDCl3
NS                     16
DS                      2
SWH              8012.820 Hz
FIDRES           0.122266 Hz
AQ              4.0894465 sec
RG                 112.31
DW                 62.400 usec
DE                   6.50 usec
TE                  296.3 K
D1             1.00000000 sec
TD0                     1

======== CHANNEL f1 ========
SFO1          400.1324710 MHz
NUC1                   1H
P1                  14.50 usec
PLW1          11.99499989 W

F2 - Processing parameters
SI                  65536
SF            400.1300096 MHz
WDW                    EM
SSB                     0
LB                   0.30 Hz
GB                      0
PC                   1.00
```

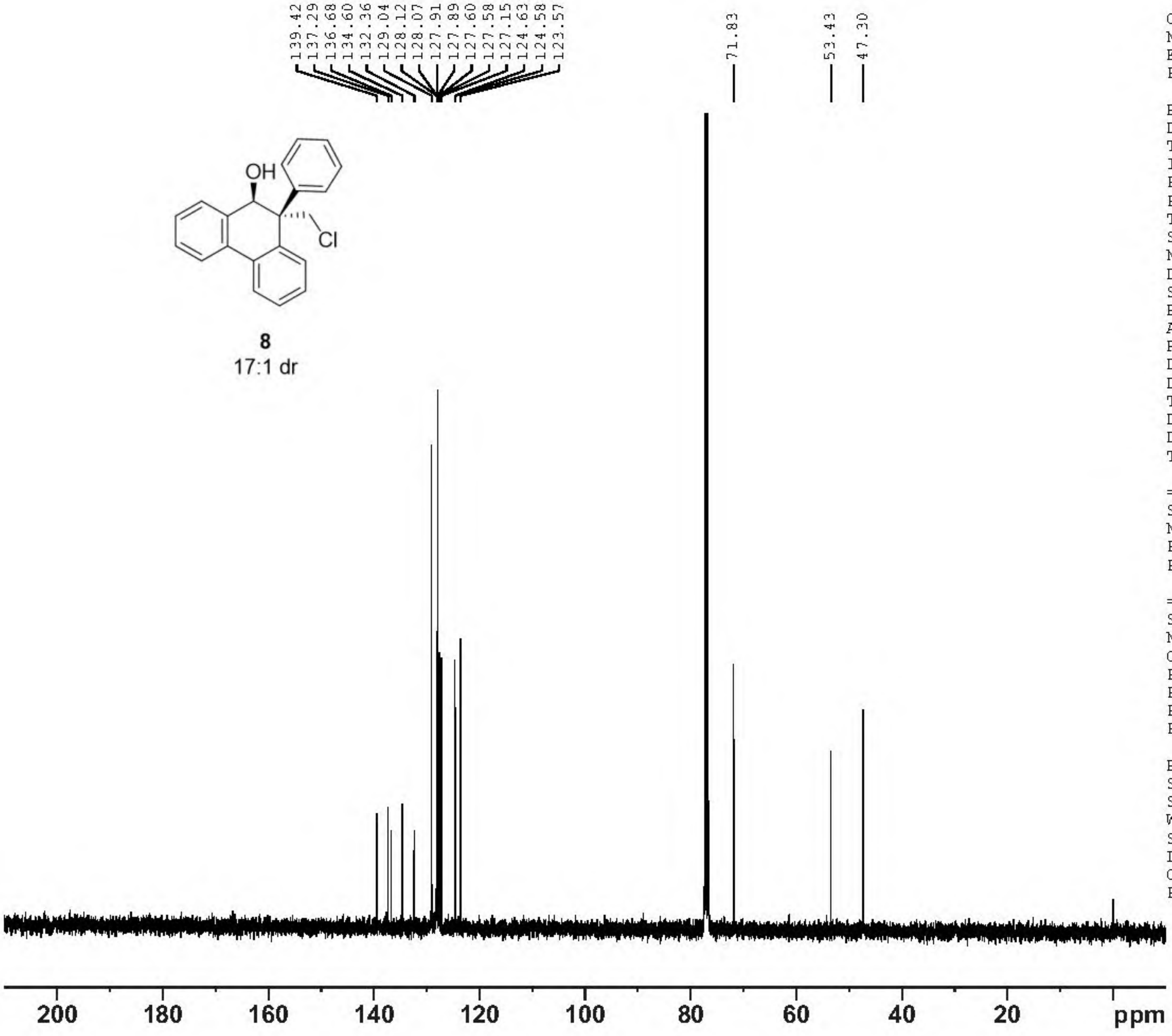

Current Data Parameters
NAME zzj-NaBH4-C
EXPNO 1
PROCNO 1

F2 - Acquisition Parameters
Date_ 20241018
Time 11.53
INSTRUM spect
PROBHD 5 mm PABBO BB/
PULPROG zgpg30
TD 65536
SOLVENT CDCl3
NS 542
DS 2
SWH 24038.461 Hz
FIDRES 0.366798 Hz
AQ 1.3631488 sec
RG 196.92
DW 20.800 usec
DE 6.50 usec
TE 297.4 K
D1 2.00000000 sec
D11 0.03000000 sec
TD0 1

======== CHANNEL f1 ========
SFO1 100.6228298 MHz
NUC1 13C
P1 9.70 usec
PLW1 46.98899841 W

======== CHANNEL f2 ========
SFO2 400.1316005 MHz
NUC2 1H
CPDPRG[2 waltz16
PCPD2 90.00 usec
PLW2 11.99499989 W
PLW12 0.34213999 W
PLW13 0.27713001 W

F2 - Processing parameters
SI 32768
SF 100.6127724 MHz
WDW EM
SSB 0
LB 1.00 Hz
GB 0
PC 1.40

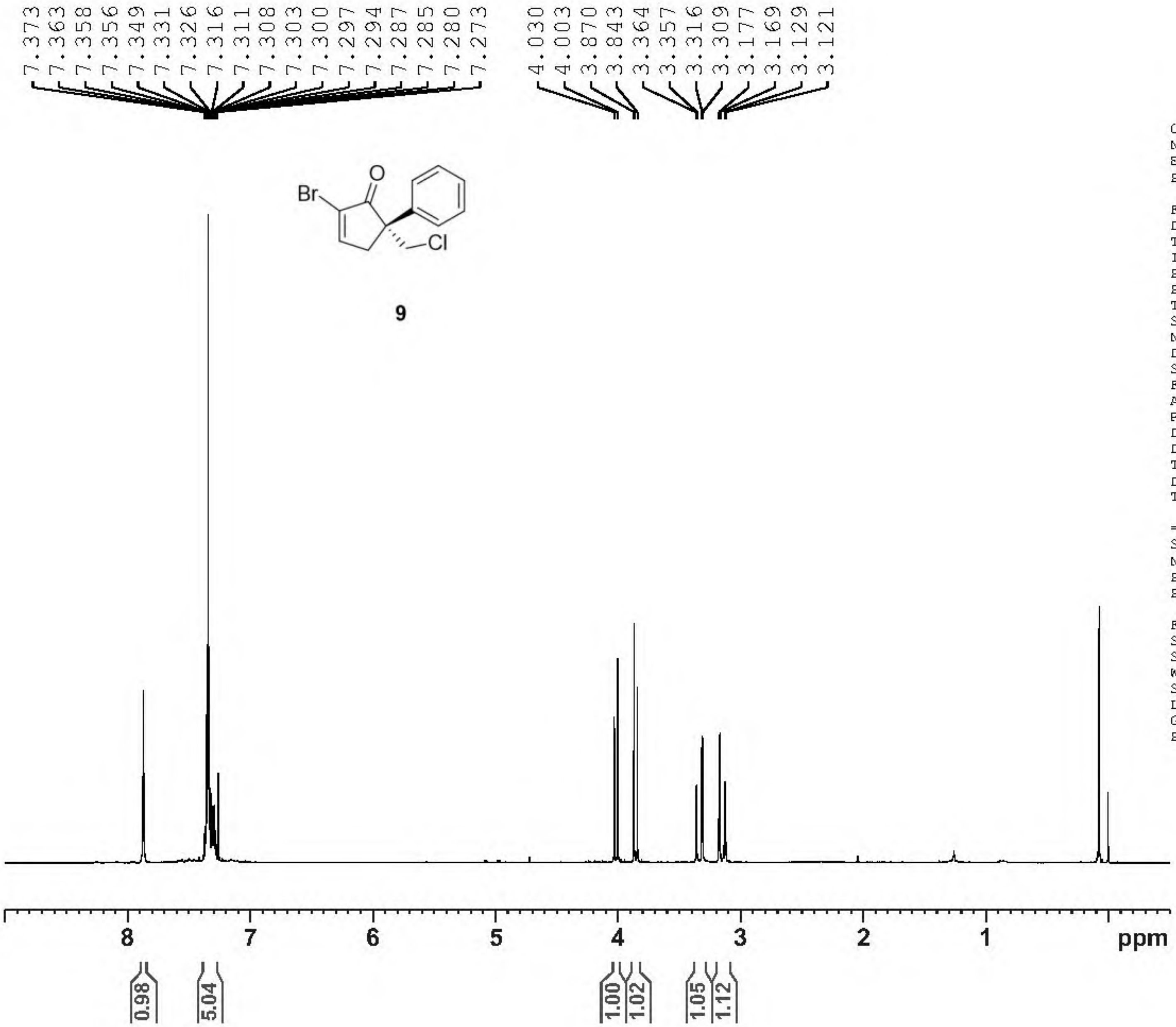


```
Current Data Parameters
NAME      transformation-monoBr-H
EXPNO                   1
PROCNO                  1

F2 - Acquisition Parameters
Date_              20251003
Time                  23.34
INSTRUM               spect
PROBHD    5 mm PABBO BB/
PULPROG                zg30
TD                    65536
SOLVENT               CDCl3
NS                       16
DS                        2
SWH             8012.820 Hz
FIDRES          0.122266 Hz
AQ             4.0894465 sec
RG                    88.84
DW               62.400 usec
DE                 6.50 usec
TE                297.4 K
D1           1.00000000 sec
TD0                       1

======== CHANNEL f1 ========
SFO1        400.1324710 MHz
NUC1                     1H
P1                14.50 usec
PLW1        11.99499989 W

F2 - Processing parameters
SI                    65536
SF          400.1300095 MHz
WDW                      EM
SSB                       0
LB                  0.30 Hz
GB                        0
PC                     1.00
```

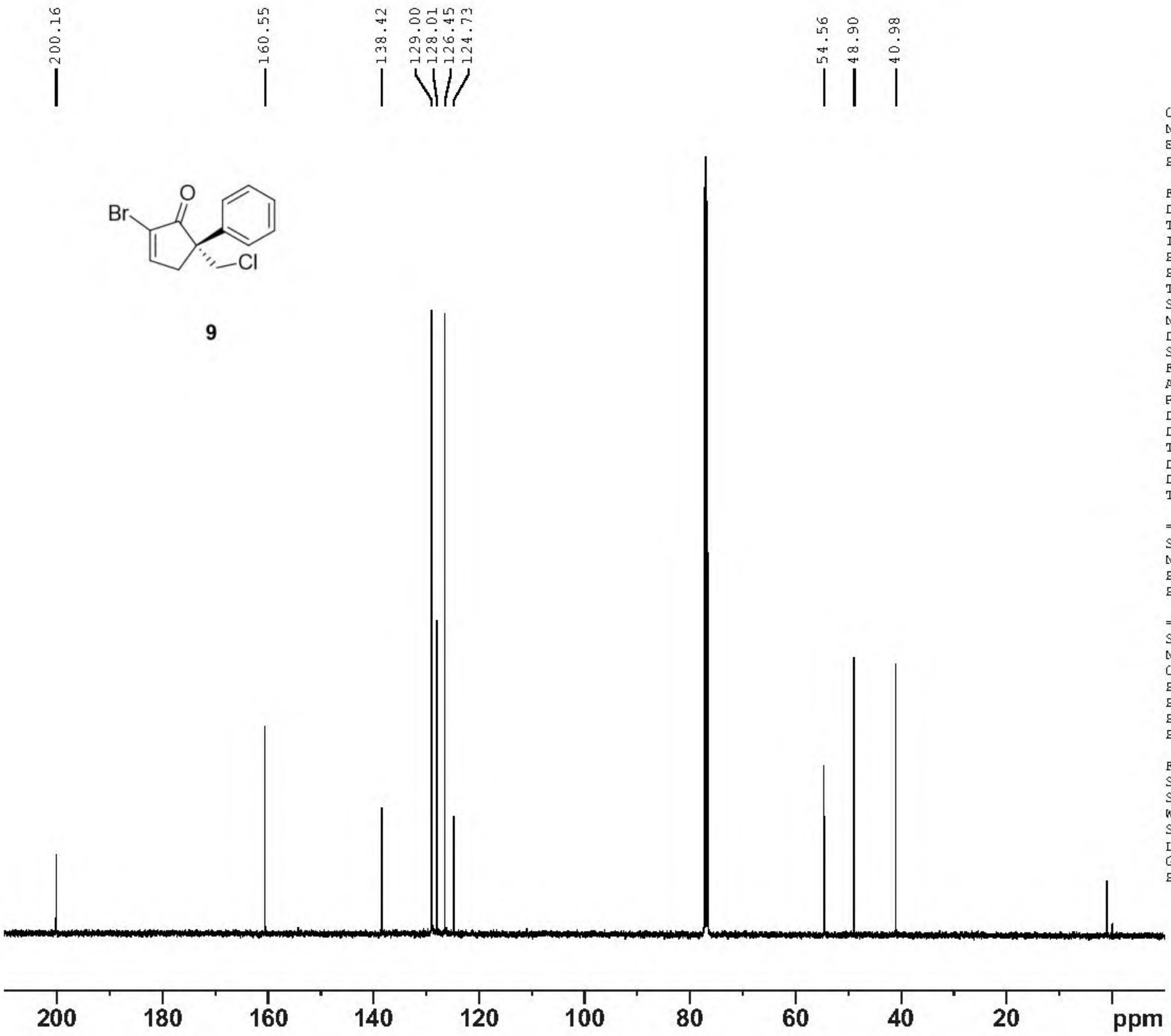

Current Data Parameters

NAME	transformation-monoBr-C	
EXPNO	1	
PROCNO	1	

F2 - Acquisition Parameters

Date_	20251004	
Time	0.32	
INSTRUM	spect	
PROBHD	5 mm PABBO BB/	
PULPROG	zgpg30	
TD	65536	
SOLVENT	CDCl3	
NS	1000	
DS	2	
SWH	24038.461	Hz
FIDRES	0.366798	Hz
AQ	1.3631488	sec
RG	196.92	
DW	20.800	usec
DE	6.50	usec
TE	298.5	K
D1	2.00000000	sec
D11	0.03000000	sec
TD0	1	

======== CHANNEL f1 ========

SFO1	100.6228298	MHz
NUC1	13C	
P1	9.70	usec
PLW1	46.98899841	W

======== CHANNEL f2 ========

SFO2	400.1316005	MHz
NUC2	1H	
CPDPRG[2	waltz16	
PCPD2	90.00	usec
PLW2	11.99499989	W
PLW12	0.34213999	W
PLW13	0.27713001	W

F2 - Processing parameters

SI	32768	
SF	100.6127760	MHz
WDW	EM	
SSB	0	
LB	1.00	Hz
GB	0	
PC	1.40	

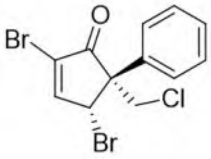

10
15:1 dr

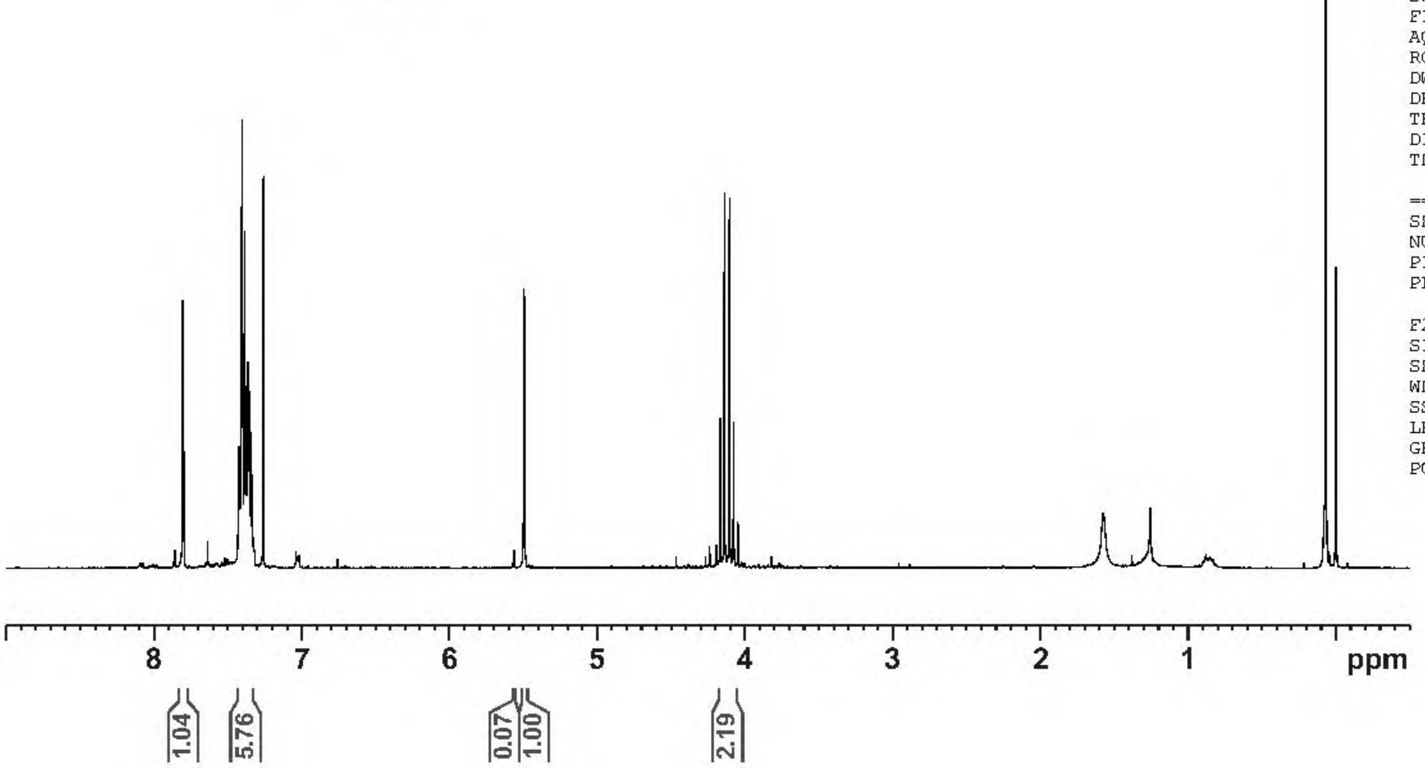


```
Current Data Parameters
NAME     transformation-diBr-H
EXPNO                    1
PROCNO                   1

F2 - Acquisition Parameters
Date_             20251003
Time                 22.31
INSTRUM              spect
PROBHD   5 mm PABBO BB/
PULPROG               zg30
TD                   65536
SOLVENT              CDCl3
NS                      16
DS                       2
SWH             8012.820 Hz
FIDRES          0.122266 Hz
AQ             4.0894465 sec
RG                  142.88
DW                  62.400 usec
DE                    6.50 usec
TE                   296.8 K
D1              1.00000000 sec
TD0                      1

======== CHANNEL f1 ========
SFO1        400.1324710 MHz
NUC1                    1H
P1                   14.50 usec
PLW1           11.99499989 W

F2 - Processing parameters
SI                   65536
SF          400.1300097 MHz
WDW                     EM
SSB                      0
LB                    0.30 Hz
GB                       0
PC                    1.00
```

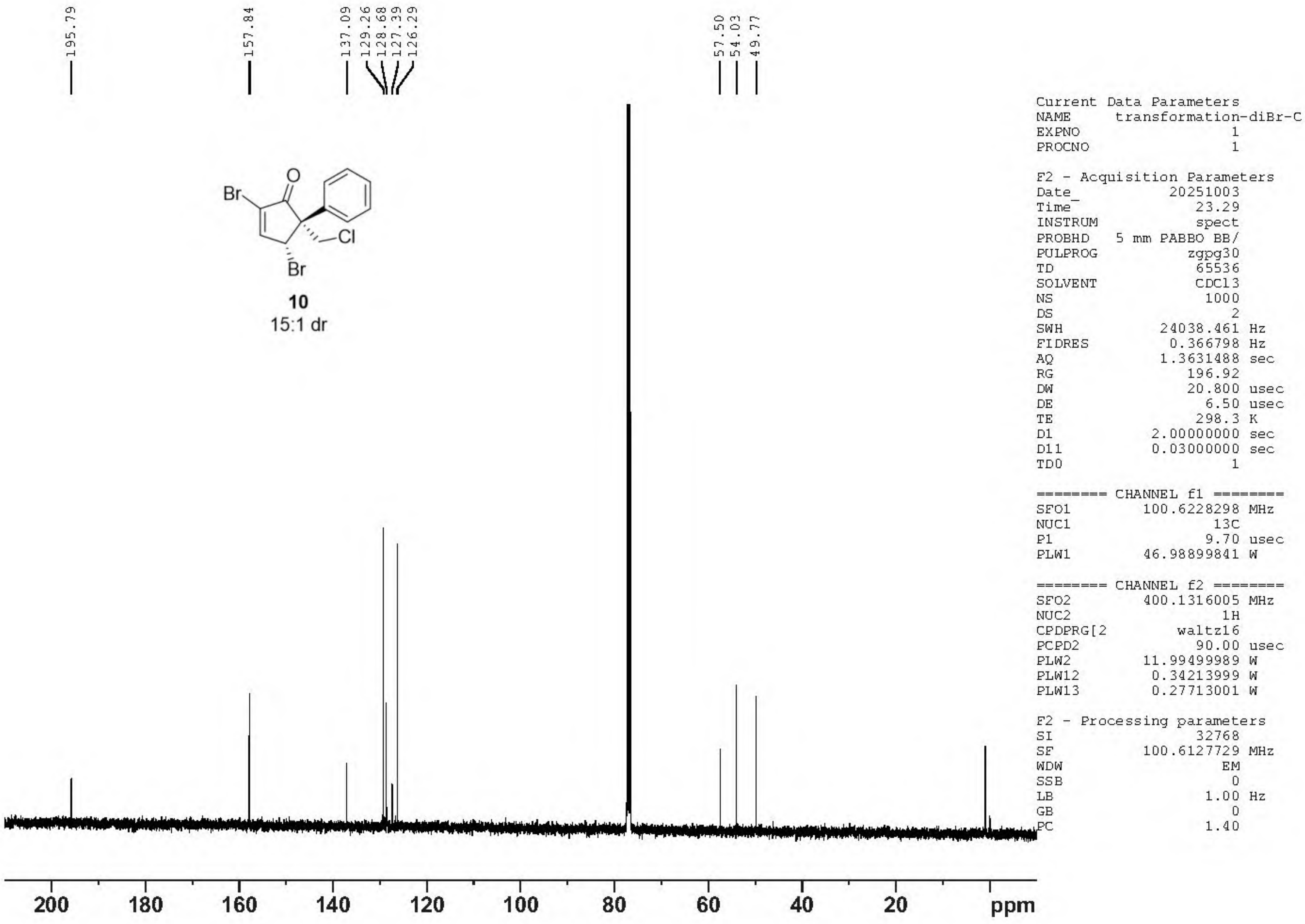
195.79
157.84
137.09
129.26
128.68
127.39
126.29
57.50
54.03
49.77
Br
O
Cl
Br
10
15:1 dr
Current Data Parameters
NAME transformation-diBr-C
EXPNO 1
PROCNO 1
F2 - Acquisition Parameters
Date_ 20251003
Time_ 23.29
INSTRUM spect
PROBHD 5 mm PABBO BB/
PULPROG zgpg30
TD 65536
SOLVENT CDCl3
NS 1000
DS 2
SWH 24038.461 Hz
FIDRES 0.366798 Hz
AQ 1.3631488 sec
RG 196.92
DW 20.800 usec
DE 6.50 usec
TE 298.3 K
D1 2.00000000 sec
D11 0.03000000 sec
TD0 1
======== CHANNEL f1 ========
SFO1 100.6228298 MHz
NUC1 13C
P1 9.70 usec
PLW1 46.98899841 W
======== CHANNEL f2 ========
SFO2 400.1316005 MHz
NUC2 1H
CPDPRG[2 waltz16
PCPD2 90.00 usec
PLW2 11.99499989 W
PLW12 0.34213999 W
PLW13 0.27713001 W
F2 - Processing parameters
SI 32768
SF 100.6127729 MHz
WDW EM
SSB 0
LB 1.00 Hz
GB 0
PC 1.40
200
180
160
140
120
100
80
60
40
20
ppm

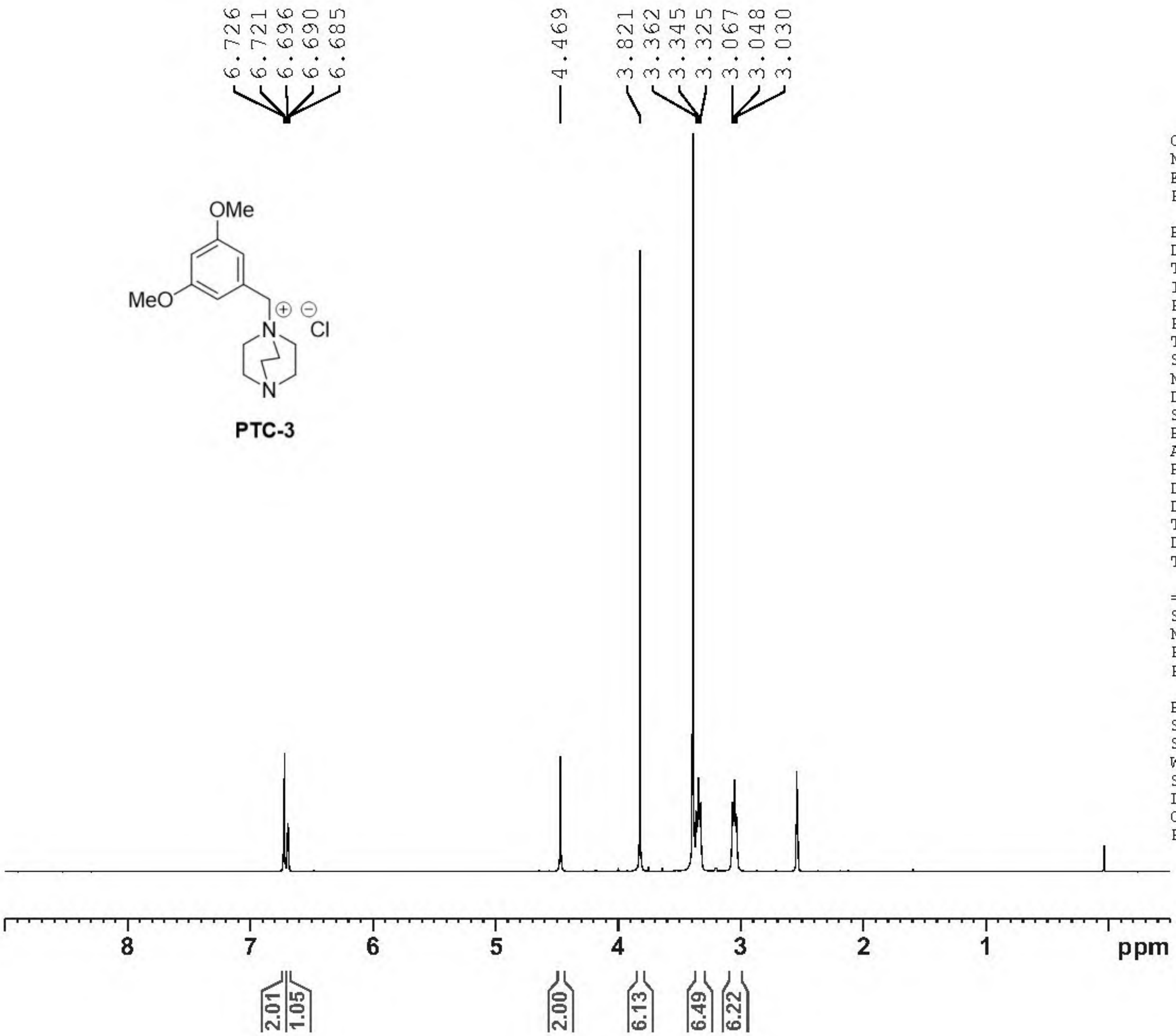


```
Current Data Parameters
NAME        zzj-PTC(OMe)-H
EXPNO                    1
PROCNO                   1

F2 - Acquisition Parameters
Date_             20240831
Time                 22.56
INSTRUM              spect
PROBHD   5 mm PABBO BB/
PULPROG               zg30
TD                   65536
SOLVENT               DMSO
NS                      16
DS                       2
SWH             8012.820 Hz
FIDRES          0.122266 Hz
AQ             4.0894465 sec
RG                  112.31
DW                  62.400 usec
DE                    6.50 usec
TE                   296.2 K
D1            1.00000000 sec
TD0                      1

======== CHANNEL f1 ========
SFO1         400.1324710 MHz
NUC1                    1H
P1                   14.50 usec
PLW1         11.99499989 W

F2 - Processing parameters
SI                   65536
SF           400.1299872 MHz
WDW                     EM
SSB                      0
LB                    0.30 Hz
GB                       0
PC                    1.00
```

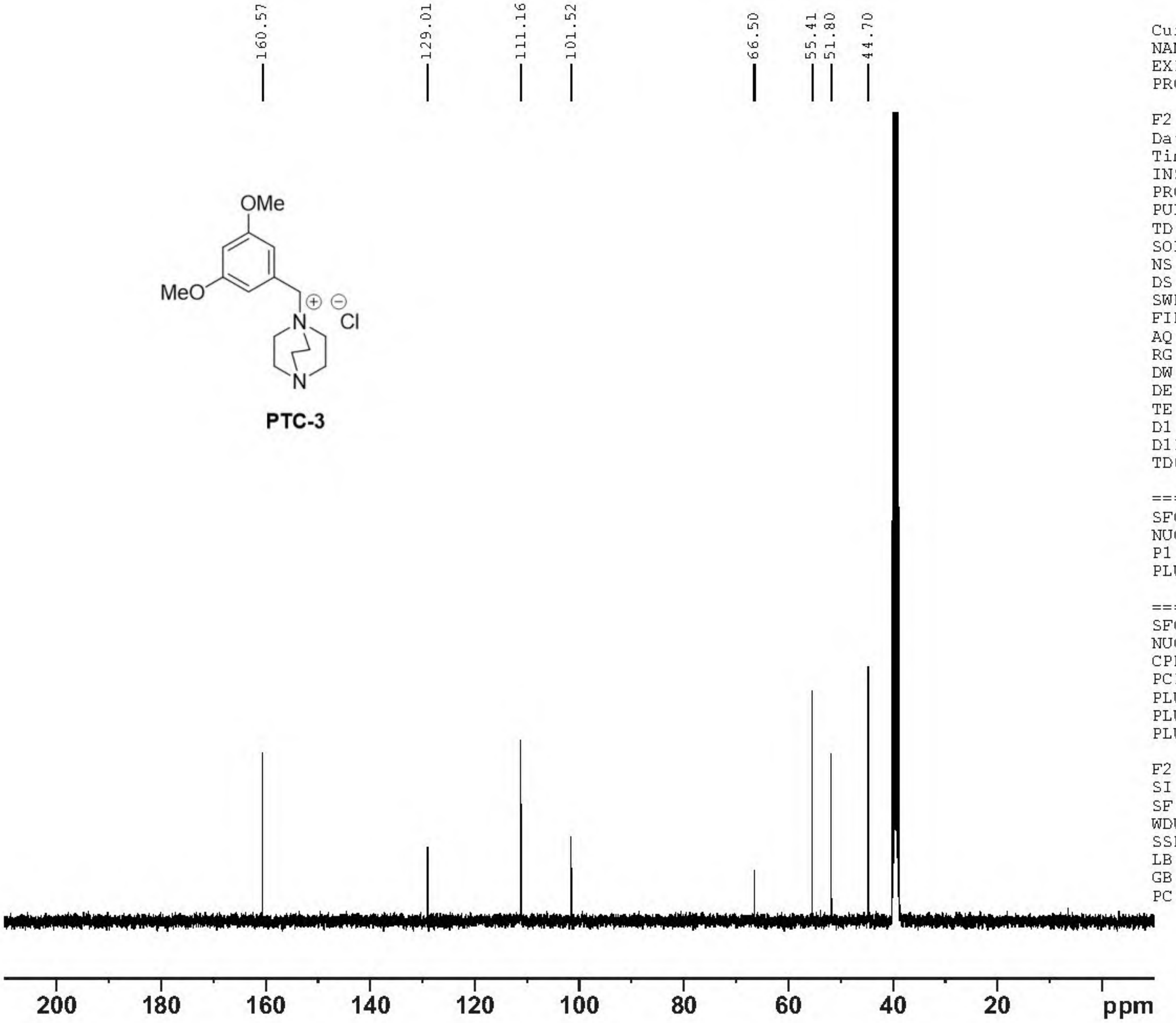


```
Current Data Parameters
NAME      zzj-PTC(OMe)-C
EXPNO                  1
PROCNO                 1

F2 - Acquisition Parameters
Date_           20240831
Time               23.54
INSTRUM            spect
PROBHD   5 mm PABBO BB/
PULPROG          zgpg30
TD                65536
SOLVENT            DMSO
NS                 1000
DS                    2
SWH           24038.461 Hz
FIDRES         0.366798 Hz
AQ            1.3631488 sec
RG               196.92
DW               20.800 usec
DE                 6.50 usec
TE                297.4 K
D1           2.00000000 sec
D11          0.03000000 sec
TD0                   1

======== CHANNEL f1 ========
SFO1        100.6228298 MHz
NUC1                13C
P1                 9.70 usec
PLW1        46.98899841 W

======== CHANNEL f2 ========
SFO2        400.1316005 MHz
NUC2                 1H
CPDPRG[2        waltz16
PCPD2             90.00 usec
PLW2        11.99499989 W
PLW12        0.34213999 W
PLW13        0.27713001 W

F2 - Processing parameters
SI                32768
SF          100.6128178 MHz
WDW                  EM
SSB                   0
LB                 1.00 Hz
GB                    0
PC                 1.40
```

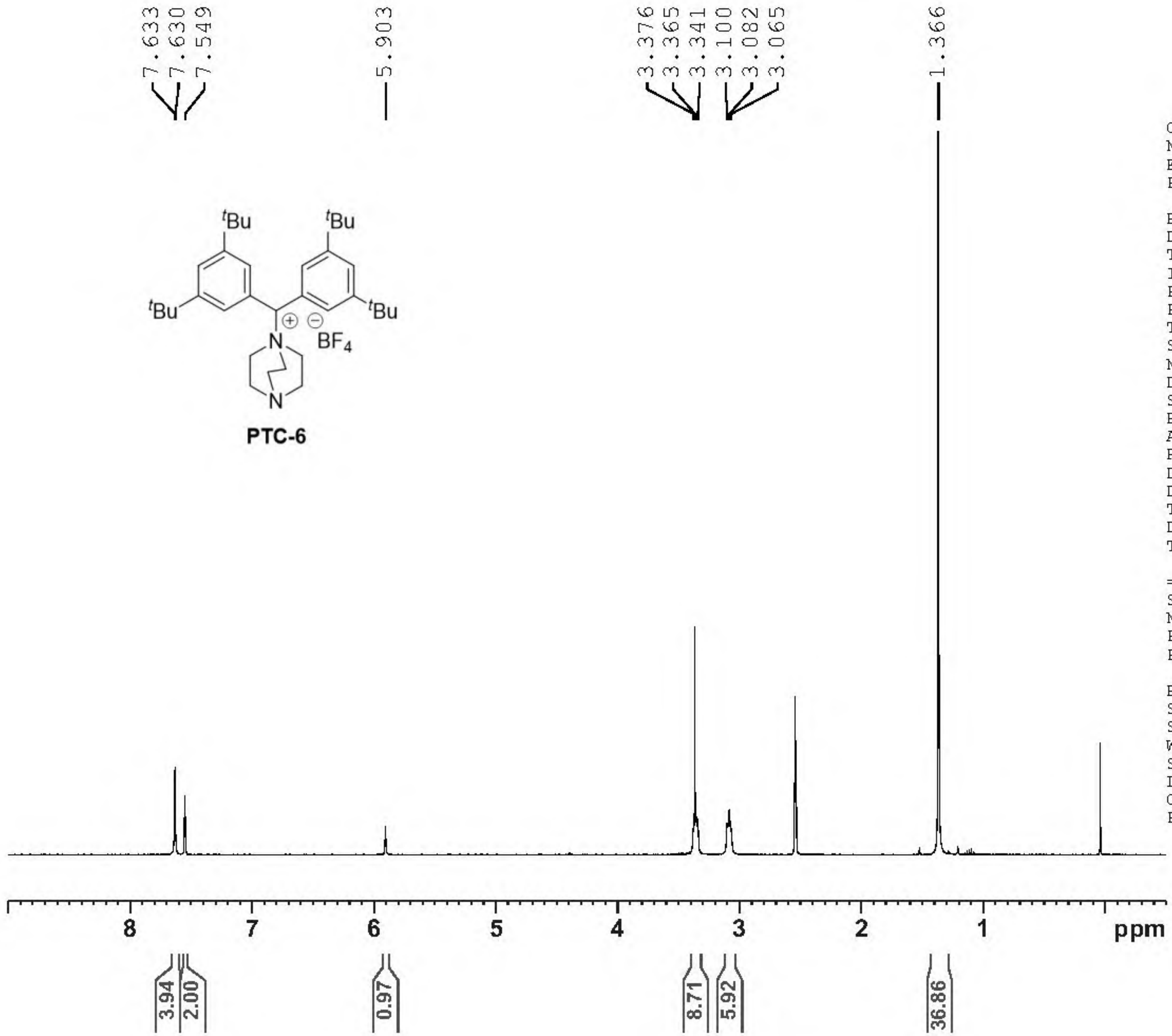


```
Current Data Parameters
NAME          zzj-PTC5-H
EXPNO                  1
PROCNO                 1

F2 - Acquisition Parameters
Date_           20241010
Time               22.47
INSTRUM            spect
PROBHD   5 mm PABBO BB/
PULPROG             zg30
TD                 65536
SOLVENT             DMSO
NS                     5
DS                     2
SWH             8012.820 Hz
FIDRES          0.122266 Hz
AQ             4.0894465 sec
RG                126.97
DW                62.400 usec
DE                  6.50 usec
TE                 296.5 K
D1            1.00000000 sec
TD0                    1

======== CHANNEL f1 ========
SFO1         400.1324710 MHz
NUC1                  1H
P1                 14.50 usec
PLW1         11.99499989 W

F2 - Processing parameters
SI                 65536
SF           400.1299871 MHz
WDW                   EM
SSB                    0
LB                  0.30 Hz
GB                     0
PC                  1.00
```

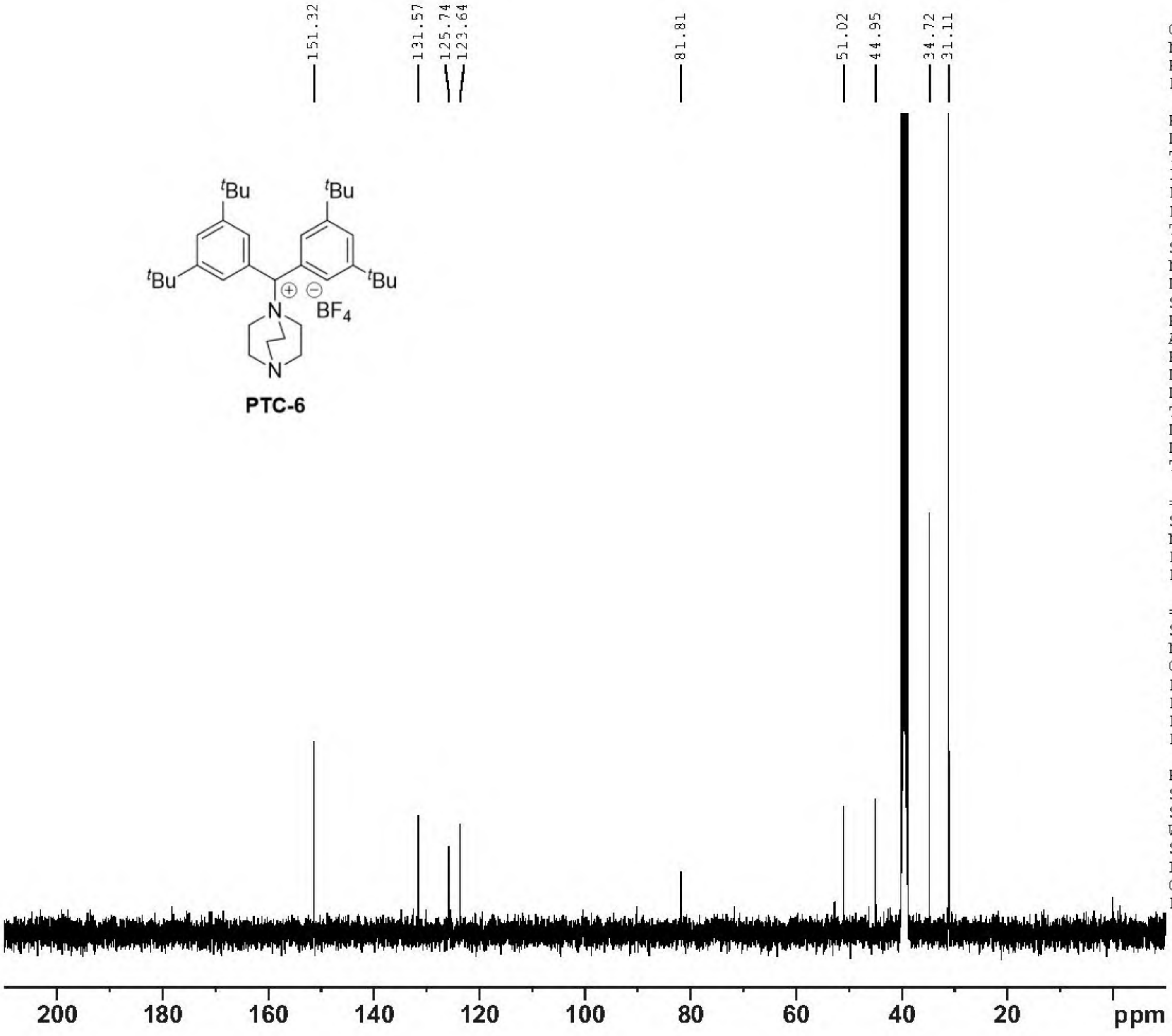

PTC-6

Current Data Parameters
NAME zzj-PTC5-C
EXPNO 1
PROCNO 1

F2 - Acquisition Parameters
Date_ 20241010
Time 23.45
INSTRUM spect
PROBHD 5 mm PABBO BB/
PULPROG zgpg30
TD 65536
SOLVENT DMSO
NS 1000
DS 2
SWH 24038.461 Hz
FIDRES 0.366798 Hz
AQ 1.3631488 sec
RG 196.92
DW 20.800 usec
DE 6.50 usec
TE 297.9 K
D1 2.00000000 sec
D11 0.03000000 sec
TD0 1

======== CHANNEL f1 ========
SFO1 100.6228298 MHz
NUC1 13C
P1 9.70 usec
PLW1 46.98899841 W

======== CHANNEL f2 ========
SFO2 400.1316005 MHz
NUC2 1H
CPDPRG[2 waltz16
PCPD2 90.00 usec
PLW2 11.99499989 W
PLW12 0.34213999 W
PLW13 0.27713001 W

F2 - Processing parameters
SI 32768
SF 100.6128193 MHz
WDW EM
SSB 0
LB 1.00 Hz
GB 0
PC 1.40

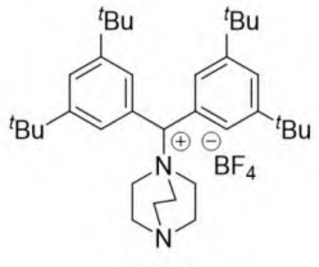

PTC-6

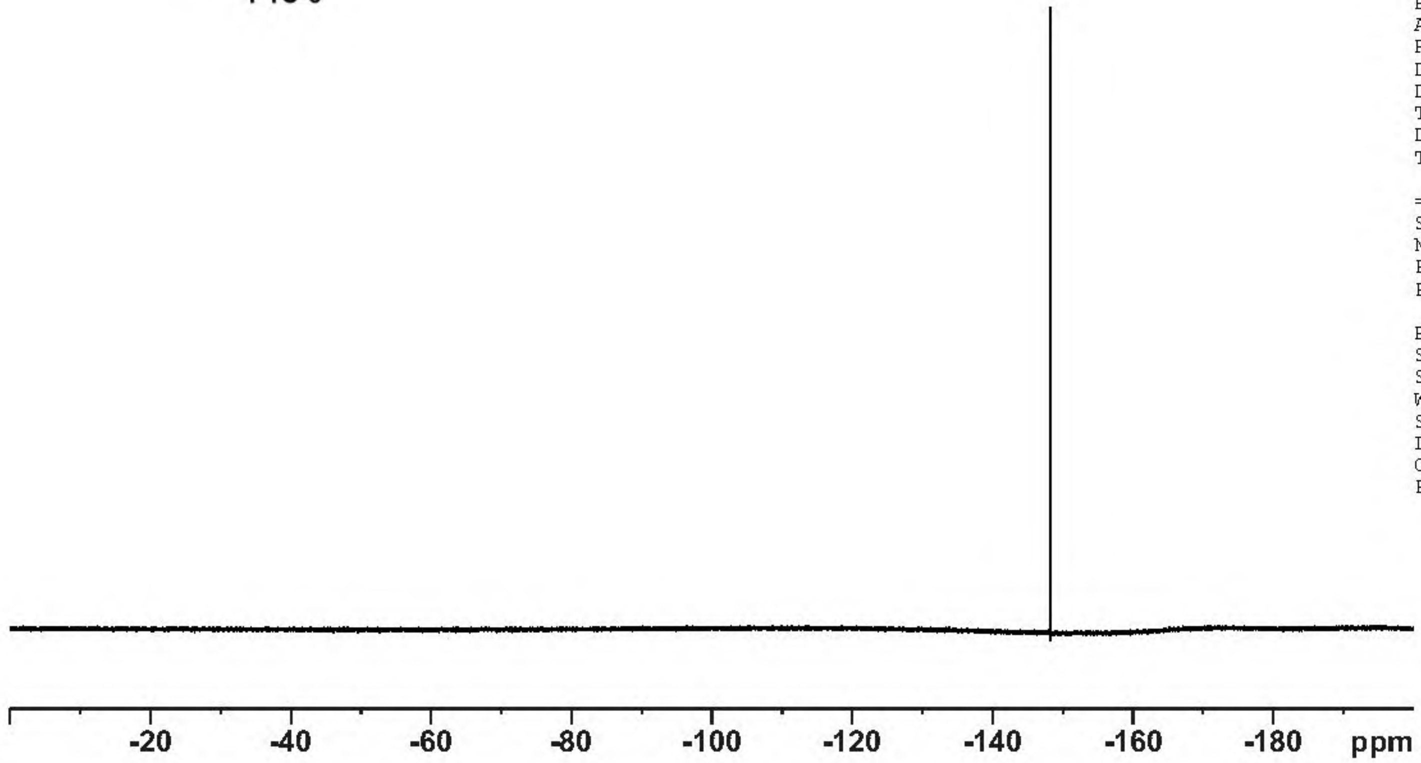

Current Data Parameters
NAME zzj-PTC5-F
EXPNO 1
PROCNO 1

F2 - Acquisition Parameters
Date_ 20241010
Time 23.47
INSTRUM spect
PROBHD 5 mm PABBO BB/
PULPROG zgflqn
TD 131072
SOLVENT DMSO
NS 16
DS 4
SWH 89285.711 Hz
FIDRES 0.681196 Hz
AQ 0.7340032 sec
RG 196.92
DW 5.600 usec
DE 6.50 usec
TE 297.3 K
D1 1.00000000 sec
TD0 1

======== CHANNEL f1 ========
SFO1 376.4607164 MHz
NUC1 19F
P1 14.70 usec
PLW1 15.99600029 W

F2 - Processing parameters
SI 65536
SF 376.4983660 MHz
WDW EM
SSB 0
LB 0.30 Hz
GB 0
PC 1.00

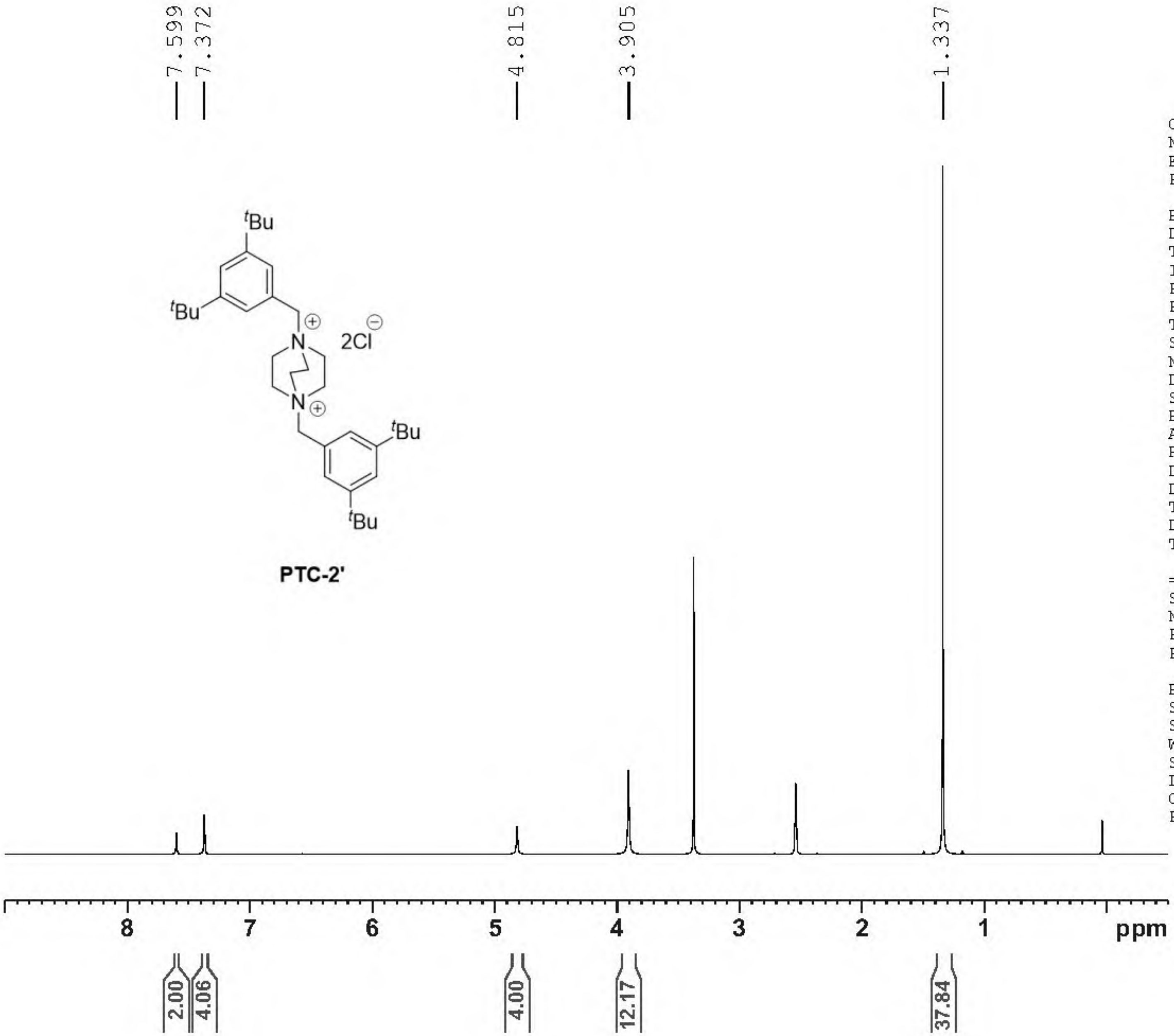


```
Current Data Parameters
NAME          zzj-PTC6-H
EXPNO                  1
PROCNO                 1

F2 - Acquisition Parameters
Date_           20241010
Time_              23.51
INSTRUM            spect
PROBHD   5 mm PABBO BB/
PULPROG             zg30
TD                 65536
SOLVENT             DMSO
NS                    16
DS                     2
SWH             8012.820 Hz
FIDRES          0.122266 Hz
AQ             4.0894465 sec
RG                126.97
DW                62.400 usec
DE                  6.50 usec
TE                 297.0 K
D1            1.00000000 sec
TD0                    1

======== CHANNEL f1 ========
SFO1         400.1324710 MHz
NUC1                  1H
P1                 14.50 usec
PLW1         11.99499989 W

F2 - Processing parameters
SI                 65536
SF           400.1299875 MHz
WDW                   EM
SSB                    0
LB                  0.30 Hz
GB                     0
PC                  1.00
```

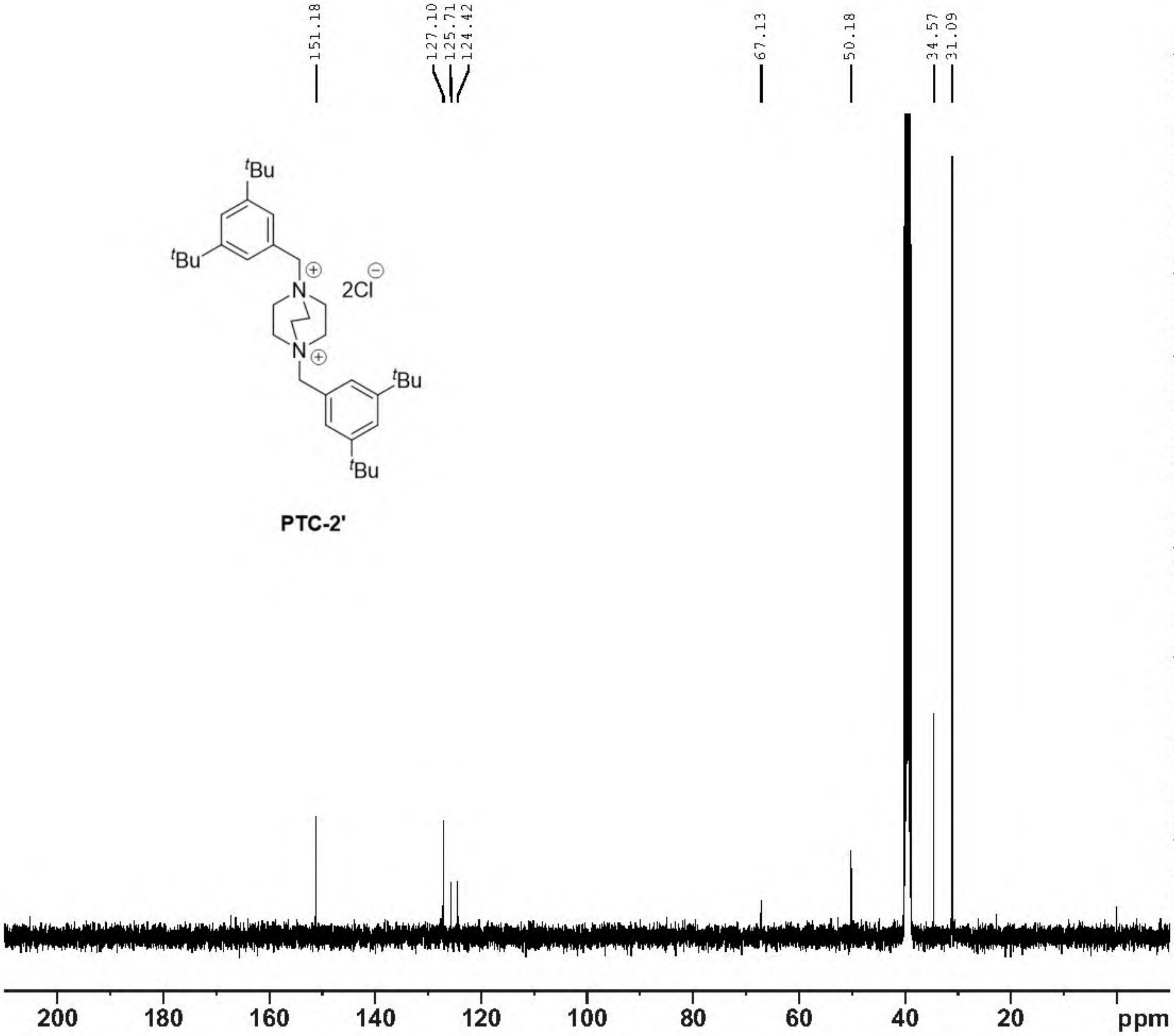

PTC-2'

Current Data Parameters
NAME zzj-PTC6-C
EXPNO 1
PROCNO 1

F2 - Acquisition Parameters
Date_ 20241011
Time 0.49
INSTRUM spect
PROBHD 5 mm PABBO BB/
PULPROG zgpg30
TD 65536
SOLVENT DMSO
NS 1000
DS 2
SWH 24038.461 Hz
FIDRES 0.366798 Hz
AQ 1.3631488 sec
RG 196.92
DW 20.800 usec
DE 6.50 usec
TE 298.1 K
D1 2.00000000 sec
D11 0.03000000 sec
TD0 1

======== CHANNEL f1 ========
SFO1 100.6228298 MHz
NUC1 13C
P1 9.70 usec
PLW1 46.98899841 W

======== CHANNEL f2 ========
SFO2 400.1316005 MHz
NUC2 1H
CPDPRG[2 waltz16
PCPD2 90.00 usec
PLW2 11.99499989 W
PLW12 0.34213999 W
PLW13 0.27713001 W

F2 - Processing parameters
SI 32768
SF 100.6128199 MHz
WDW EM
SSB 0
LB 1.00 Hz
GB 0
PC 1.40

Data File C:\Users\P...Documents\ChemStation\1\Data\SUN\SUN 2024-05-30 22-46-55\1DA-0201.D
Sample Name: zzj-4-21-7-rac

Acq. Operator : SYSTEM Seq. Line : 2
Sample Operator : SYSTEM
Acq. Instrument : HPLC Location : P1-D-01
Injection Date : 30/5/2024 10:59:48 pm Inj : 1
Inj Volume : 2.000 µl
Different Inj Volume from Sample Entry! Actual Inj Volume : 1.000 µl
Method : C:\Users\Public\Documents\ChemStation\1\Data\SUN\SUN 2024-05-30 22-46-55\OD3-05-20.M (Sequence Method)
Last changed : 17/11/2023 2:04:40 pm by SYSTEM
Additional Info : Peak(s) manually integrated

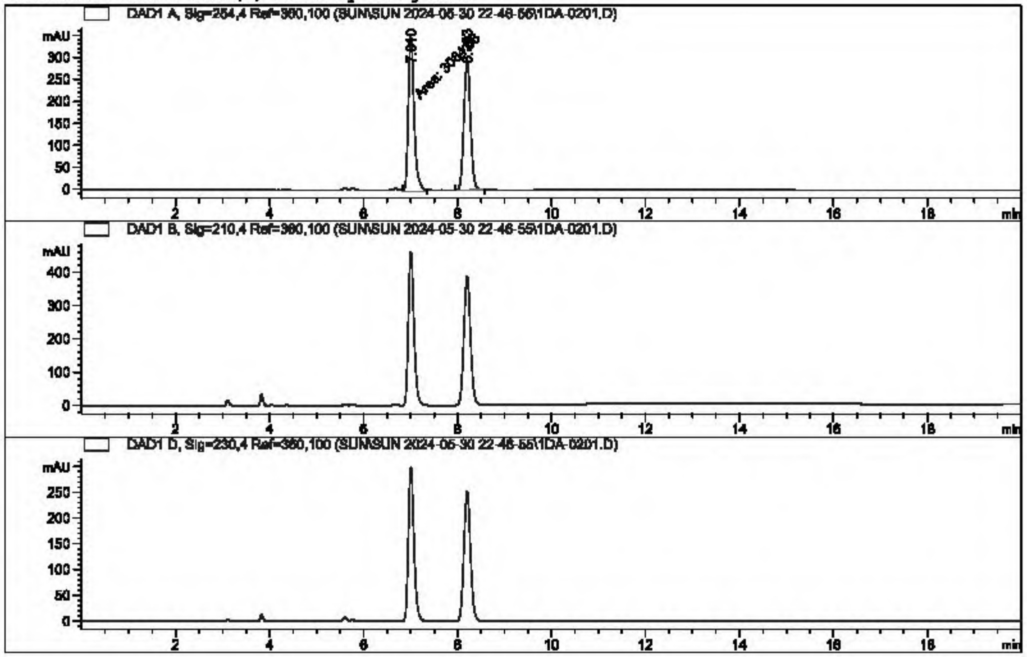

Area Percent Report

Sorted By : Signal
Multiplier : 1.0000
Dilution : 1.0000
Use Multiplier & Dilution Factor with ISTDs

Signal 1: DAD1 A, Sig=254,4 Ref=360,100

Peak #	RetTime [min]	Type	Width [min]	Area [mAU*s]	Height [mAU]	Area %
1	7.010	MM	0.1454	3084.87842	353.52301	51.5803
2	8.203	BB	0.1529	2895.84937	294.06174	48.4197

Data File C:\Users\P...Documents\ChemStation\1\Data\SUN\SUN 2024-05-30 22-46-55\1DA-0201.D
Sample Name: zzj-4-21-7-rac

Peak #	RetTime [min]	Type	Width [min]	Area [mAU*s]	Height [mAU]	Area %
Totals :				5980.72778	647.58475	

Signal 2: DAD1 B, Sig=210,4 Ref=360,100

Signal 3: DAD1 D, Sig=230,4 Ref=360,100

*** End of Report ***

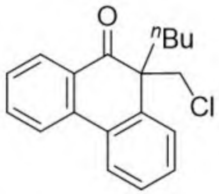

3a-rac

Data File C:\Users\P...\ChemStation\1\Data\SUN\SUN 2023-12-07 12-30-00\OnlineEdited--036.D
Sample Name: zzj-3-6

Acq. Operator : SYSTEM		Seq. Line :	36
Sample Operator : SYSTEM			
Acq. Instrument : HPLC		Location :	P2-E-04
Injection Date : 8/12/2023 12:27:04 am		Inj :	1
		Inj Volume :	2.000 µl

Different Inj Volume from Sample Entry! Actual Inj Volume : 25.000 µl
Method : C:\Users\Public\Documents\ChemStation\1\Data\SUN\SUN 2023-12-07 12-30-00\OD3-05-20.M (Sequence Method)
Last changed : 17/11/2023 2:04:40 pm by SYSTEM
Additional Info : Peak(s) manually integrated

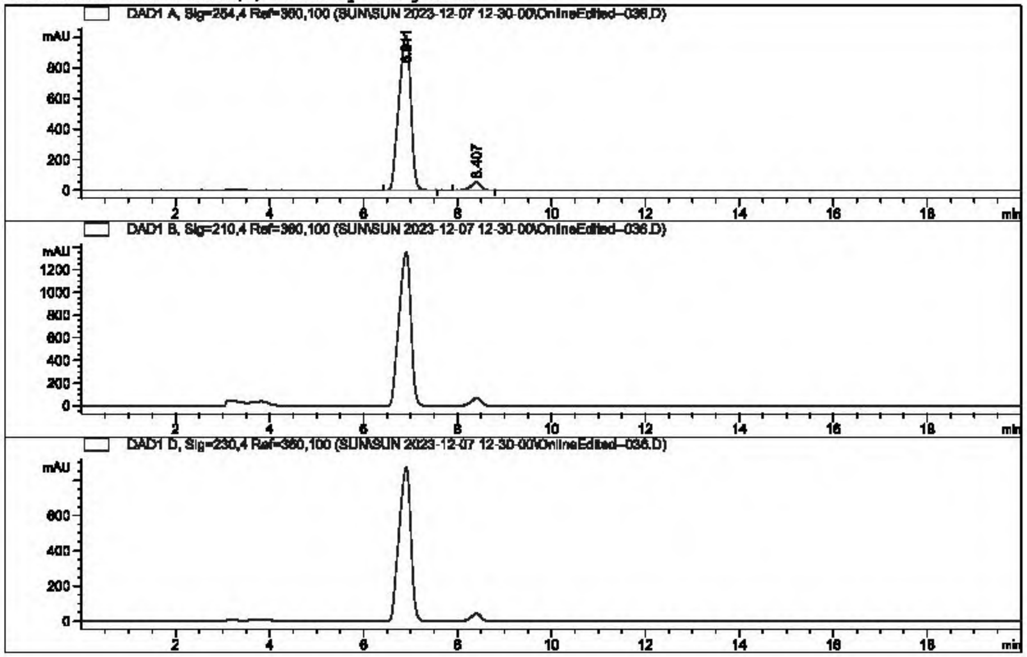

Area Percent Report

Sorted By : Signal
Multiplier : 1.0000
Dilution : 1.0000
Use Multiplier & Dilution Factor with ISTDs

Signal 1: DAD1 A, Sig=254,4 Ref=360,100

Peak #	RetTime [min]	Type	Width [min]	Area [mAU*s]	Height [mAU]	Area %
1	6.911	BB	0.2747	1.77986e4	1006.90277	95.6813
2	8.407	BB	0.2275	803.35712	52.36546	4.3187

Data File C:\Users\P...\ChemStation\1\Data\SUN\SUN 2023-12-07 12-30-00\OnlineEdited--036.D
Sample Name: zzj-3-6

Peak #	RetTime [min]	Type	Width [min]	Area [mAU*s]	Height [mAU]	Area %
Totals :				1.86020e4	1059.26823	

Signal 2: DAD1 B, Sig=210,4 Ref=360,100

Signal 3: DAD1 D, Sig=230,4 Ref=360,100

*** End of Report ***

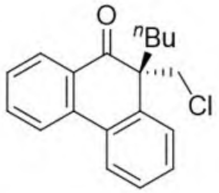

3a

```
Acq. Operator   : SYSTEM                       Seq. Line :  22
Sample Operator : SYSTEM
Acq. Instrument : HPLC                          Location :  P1-D-02
Injection Date  : 7/6/2024 12:56:26 am               Inj :  1
                                              Inj Volume : 2.000 µl
Different Inj Volume from Sample Entry!  Actual Inj Volume : 20.000 µl
Method          : C:\Users\Public\Documents\ChemStation\1\Data\SUN\SUN 2024-06-06 19-05-23\AS3-
                  03-20.M (Sequence Method)
Last changed    : 24/11/2023 8:39:22 pm by SYSTEM
Additional Info : Peak(s) manually integrated
```

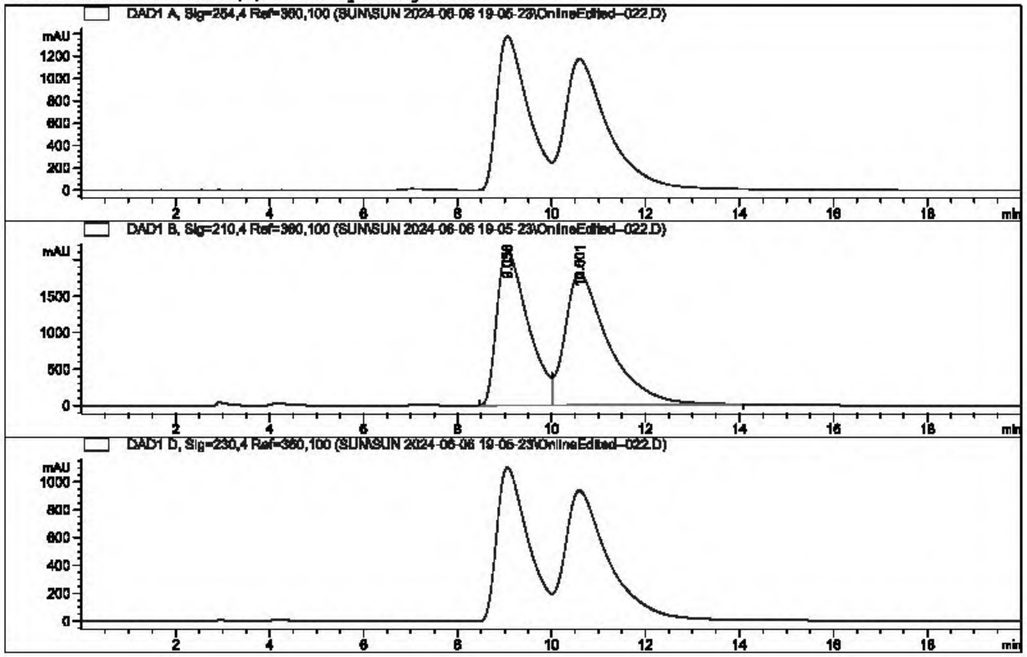

Area Percent Report

```
Sorted By             :      Signal
Multiplier            :      1.0000
Dilution              :      1.0000
Use Multiplier & Dilution Factor with ISTDs
```

Signal 1: DAD1 A, Sig=254,4 Ref=360,100

Data File C:\Users\P...\ChemStation\1\Data\SUN\SUN 2024-06-06 19-05-23\OnlineEdited--022.D
Sample Name: zzj-4-21-12

Signal 2: DAD1 B, Sig=210,4 Ref=360,100

Peak #	RetTime [min]	Type	Width [min]	Area [mAU*s]	Height [mAU]	Area %
1	9.058	BV	0.5436	9.74152e4	2106.17773	45.5968
2	10.601	VBA	0.7598	1.16230e5	1801.40759	54.4032
Totals :				2.13645e5	3907.58533	

Signal 3: DAD1 D, Sig=230,4 Ref=360,100

*** End of Report ***

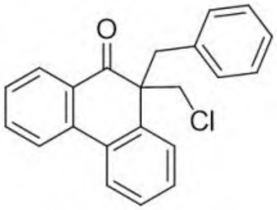

3b-rac

Data File C:\Users\P...\ChemStation\1\Data\SUN\SUN 2024-01-19 09-29-29\OnlineEdited--084.D
Sample Name: zzj-3-75

Acq. Operator : SYSTEM Seq. Line : 84
Sample Operator : SYSTEM
Acq. Instrument : HPLC Location : P1-F-01
Injection Date : 20/1/2024 4:29:32 pm Inj : 1
Inj Volume : 2.000 µl
Different Inj Volume from Sample Entry! Actual Inj Volume : 25.000 µl
Method : C:\Users\Public\Documents\ChemStation\1\Data\SUN\SUN 2024-01-19 09-29-29\AS3-03-20.M (Sequence Method)
Last changed : 24/11/2023 8:39:22 pm by SYSTEM
Additional Info : Peak(s) manually integrated

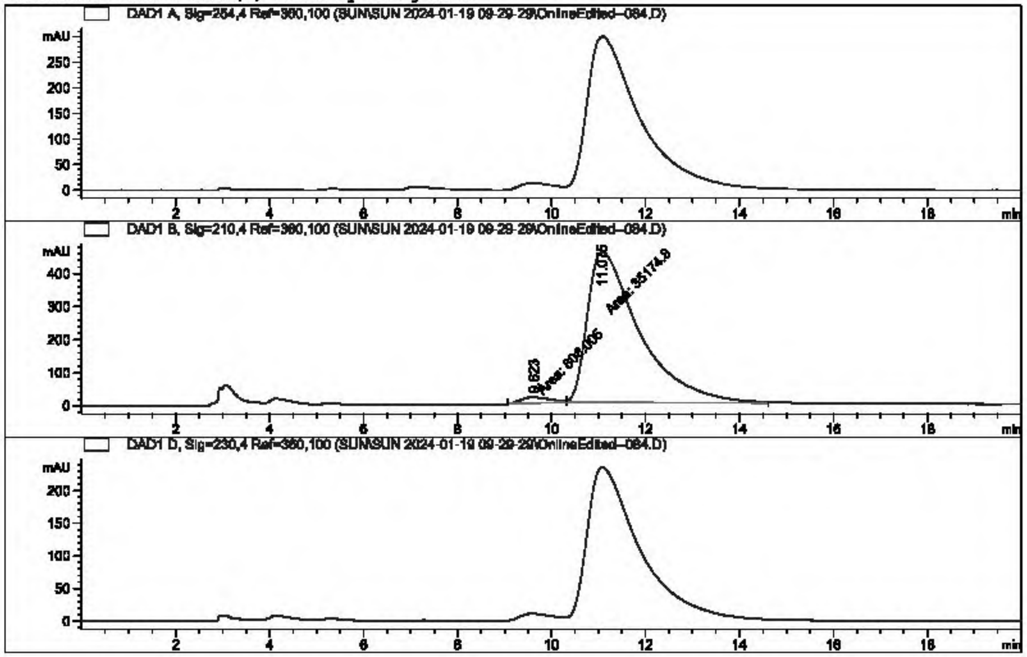

Area Percent Report

Sorted By : Signal
Multiplier : 1.0000
Dilution : 1.0000
Use Multiplier & Dilution Factor with ISTDs

Signal 1: DAD1 A, Sig=254,4 Ref=360,100

Data File C:\Users\P...\ChemStation\1\Data\SUN\SUN 2024-01-19 09-29-29\OnlineEdited--084.D
Sample Name: zzj-3-75

Signal 2: DAD1 B, Sig=210,4 Ref=360,100

Peak #	RetTime [min]	Type	Width [min]	Area [mAU*s]	Height [mAU]	Area %
1	9.623	MM	0.7375	808.00494	18.26054	2.2455
2	11.075	MM	1.2855	3.51748e4	456.04828	97.7545

Totals : 3.59828e4 474.30882

Signal 3: DAD1 D, Sig=230,4 Ref=360,100

*** End of Report ***

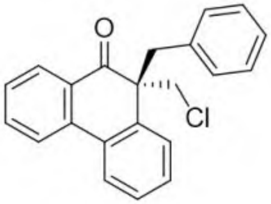

3b

```
=====================================================================
Acq. Operator   : SYSTEM                        Seq. Line :   8
Sample Operator : SYSTEM
Acq. Instrument : HPLC                           Location :   P1-D-02
Injection Date  : 10/6/2024 6:59:26 pm                Inj :   1
                                               Inj Volume : 2.000 µl
Different Inj Volume from Sample Entry!  Actual Inj Volume : 5.000 µl
Method          : C:\Users\Public\Documents\ChemStation\1\Data\SUN\SUN 2024-06-10 17-07-17\IBN3-
                  05-30.M (Sequence Method)
Last changed    : 29/8/2022 6:09:41 pm by SYSTEM
Additional Info : Peak(s) manually integrated
```

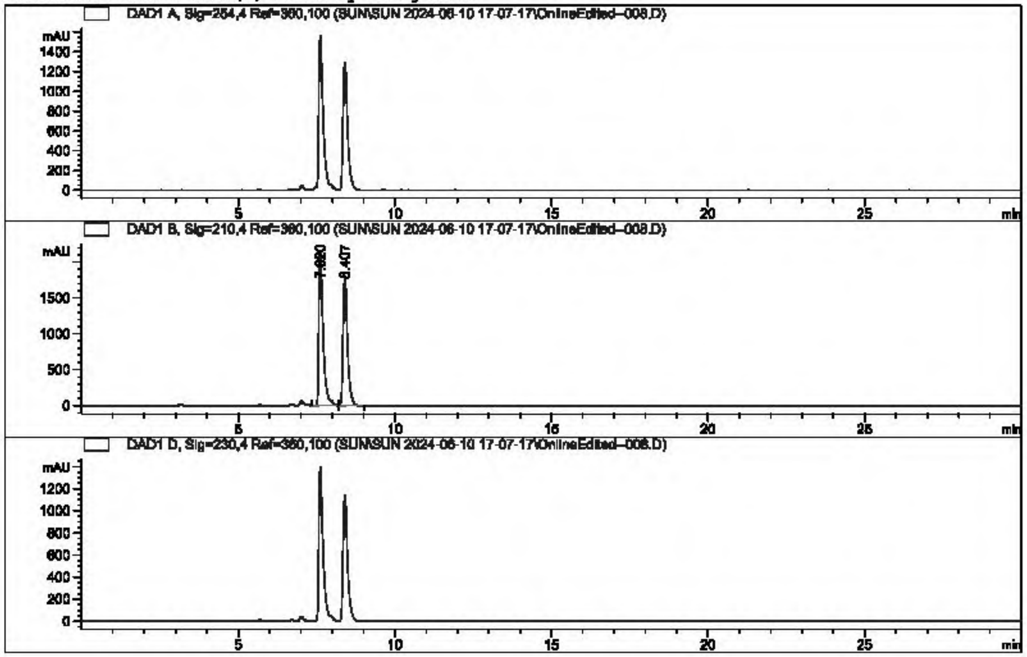


```
=====================================================================
                         Area Percent Report
=====================================================================

Sorted By             :      Signal
Multiplier            :      1.0000
Dilution              :      1.0000
Use Multiplier & Dilution Factor with ISTDs
```

Signal 1: DAD1 A, Sig=254,4 Ref=360,100

Data File C:\Users\P...\ChemStation\1\Data\SUN\SUN 2024-06-10 17-07-17\OnlineEdited--008.D
Sample Name: zzj-4-21-13

Signal 2: DAD1 B, Sig=210,4 Ref=360,100

Peak #	RetTime [min]	Type	Width [min]	Area [mAU*s]	Height [mAU]	Area %
1	7.620	BB	0.1421	2.03788e4	2139.37109	53.8698
2	8.407	BB	0.1504	1.74510e4	1772.62073	46.1302
Totals :				3.78298e4	3911.99182	

Signal 3: DAD1 D, Sig=230,4 Ref=360,100

*** End of Report ***

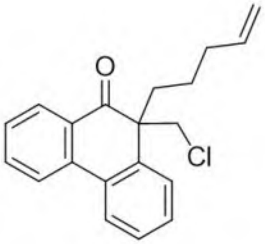

3c-rac

```
Acq. Operator   : SYSTEM                        Seq. Line :  15
Sample Operator : SYSTEM
Acq. Instrument : HPLC                           Location :  P1-C-06
Injection Date  : 13/4/2024 10:35:42 pm               Inj :   1
                                               Inj Volume : 2.000 µl
Different Inj Volume from Sample Entry!  Actual Inj Volume : 25.000 µl
Method          : C:\Users\Public\Documents\ChemStation\1\Data\SUN\SUN 2024-04-13 19-43-27\IBN3-
                  05-30.M (Sequence Method)
Last changed    : 29/8/2022 6:09:41 pm by SYSTEM
Additional Info : Peak(s) manually integrated
```

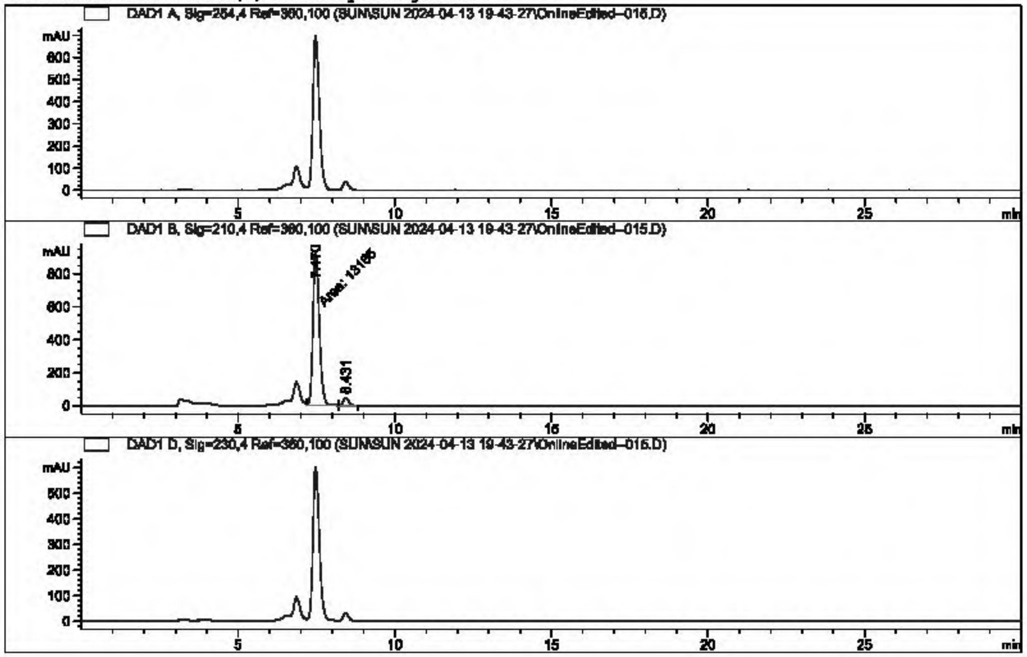

Area Percent Report

```
Sorted By             :      Signal
Multiplier            :      1.0000
Dilution              :      1.0000
Use Multiplier & Dilution Factor with ISTDs
```

Signal 1: DAD1 A, Sig=254,4 Ref=360,100

Data File C:\Users\P...\ChemStation\1\Data\SUN\SUN 2024-04-13 19-43-27\OnlineEdited--015.D
Sample Name: zzj-3-161-ibn

Signal 2: DAD1 B, Sig=210,4 Ref=360,100

Peak #	RetTime [min]	Type	Width [min]	Area [mAU*s]	Height [mAU]	Area %
1	7.470	FM	0.2355	1.31650e4	931.76965	95.8091
2	8.431	BB	0.1940	575.85962	46.48138	4.1909
Totals :				1.37408e4	978.25104	

Signal 3: DAD1 D, Sig=230,4 Ref=360,100

*** End of Report ***

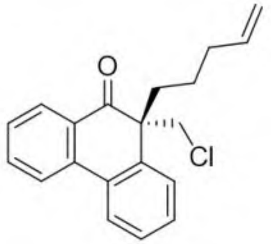

3c

```
Acq. Operator   : SYSTEM                        Seq. Line :  10
Sample Operator : SYSTEM
Acq. Instrument : HPLC                           Location :   P1-D-03
Injection Date  : 10/6/2024 7:42:11 pm                Inj :   1
                                               Inj Volume : 2.000 µl
Different Inj Volume from Sample Entry!  Actual Inj Volume : 5.000 µl
Method          : C:\Users\Public\Documents\ChemStation\1\Data\SUN\SUN 2024-06-10 17-07-17\OD3-5
                  -30.M (Sequence Method)
Last changed    : 22/8/2022 12:41:47 pm by SYSTEM
Additional Info : Peak(s) manually integrated
```

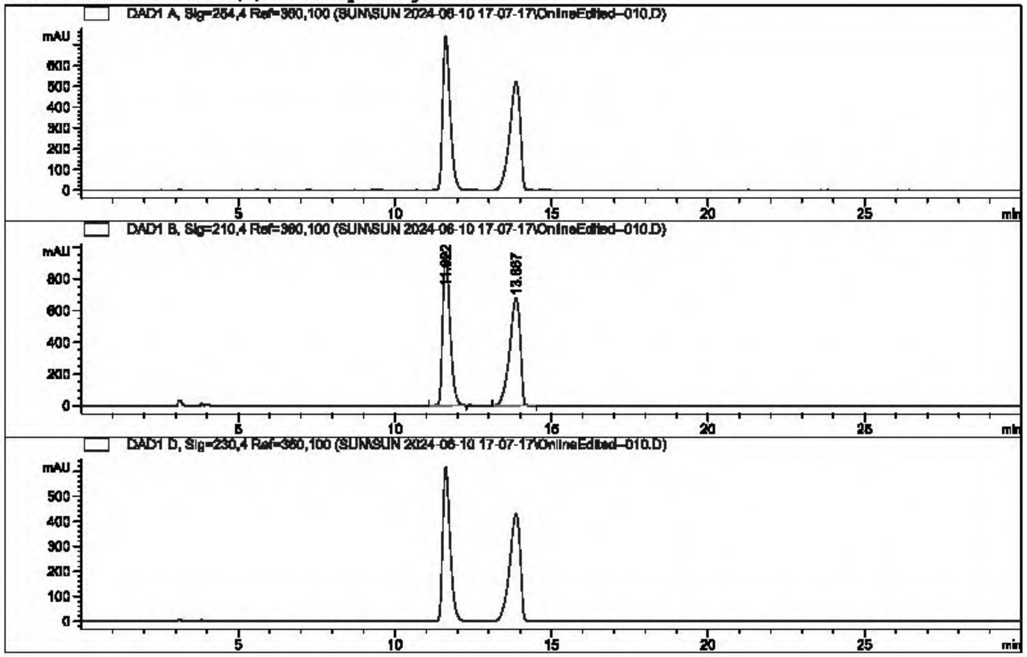

Area Percent Report

```
Sorted By             :      Signal
Multiplier            :      1.0000
Dilution              :      1.0000
Use Multiplier & Dilution Factor with ISTDs
```

Signal 1: DAD1 A, Sig=254,4 Ref=360,100

Data File C:\Users\P...\ChemStation\1\Data\SUN\SUN 2024-06-10 17-07-17\OnlineEdited--010.D
Sample Name: zzj-4-21-14

Signal 2: DAD1 B, Sig=210,4 Ref=360,100

Peak #	RetTime [min]	Type	Width [min]	Area [mAU*s]	Height [mAU]	Area %
1	11.622	BB	0.2489	1.56960e4	966.68018	49.8362
2	13.867	BB	0.3718	1.57992e4	673.28259	50.1638

Totals : 3.14952e4 1639.96277

Signal 3: DAD1 D, Sig=230,4 Ref=360,100

*** End of Report ***

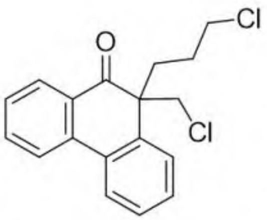

3d-rac

Acq. Operator : SYSTEM Seq. Line : 14
Sample Operator : SYSTEM
Acq. Instrument : HPLC Location : P1-C-01
Injection Date : 16/3/2024 12:52:11 am Inj : 1
Inj Volume : 2.000 µl
Different Inj Volume from Sample Entry! Actual Inj Volume : 25.000 µl
Method : C:\Users\Public\Documents\ChemStation\1\Data\SUN\SUN 2024-03-15 20-35-52\OD3-5-30.M (Sequence Method)
Last changed : 22/8/2022 12:41:47 pm by SYSTEM
Additional Info : Peak(s) manually integrated

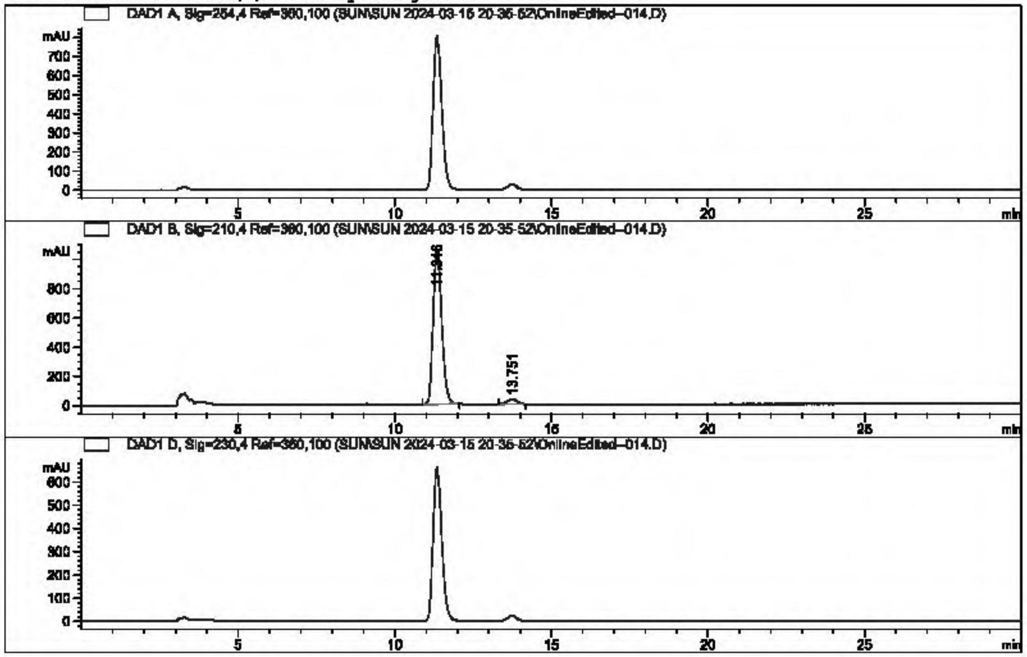

Area Percent Report

Sorted By : Signal
Multiplier : 1.0000
Dilution : 1.0000
Use Multiplier & Dilution Factor with ISTDs

Signal 1: DAD1 A, Sig=254,4 Ref=360,100

Data File C:\Users\P...\ChemStation\1\Data\SUN\SUN 2024-03-15 20-35-52\OnlineEdited--014.D
Sample Name: zzj-3-133

Signal 2: DAD1 B, Sig=210,4 Ref=360,100

Peak #	RetTime [min]	Type	Width [min]	Area [mAU*s]	Height [mAU]	Area %
1	11.346	VV R	0.2267	1.98741e4	1038.83264	96.6414
2	13.751	VV R	0.2294	690.68420	35.67509	3.3586

Totals : 2.05648e4 1074.50773

Signal 3: DAD1 D, Sig=230,4 Ref=360,100

*** End of Report ***

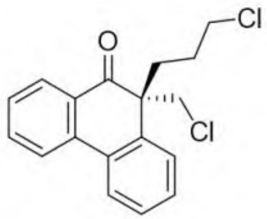

3d

Data File C:\Users\P...Documents\ChemStation\1\Data\SUN\SUN 2024-06-25 21-39-59\1AA-0201.D
Sample Name: zzj-4-38-15-rac

Acq. Operator	: SYSTEM	Seq. Line :	2
Sample Operator	: SYSTEM		
Acq. Instrument	: HPLC	Location :	P1-A-01
Injection Date	: 25/6/2024 9:53:24 pm	Inj :	1
		Inj Volume :	2.000 µl

Different Inj Volume from Sample Entry! Actual Inj Volume : 5.000 µl
Method : C:\Users\Public\Documents\ChemStation\1\Data\SUN\SUN 2024-06-25 21-39-59\IE3-20-30.M (Sequence Method)
Last changed : 21/9/2022 10:09:09 am by SYSTEM
Additional Info : Peak(s) manually integrated

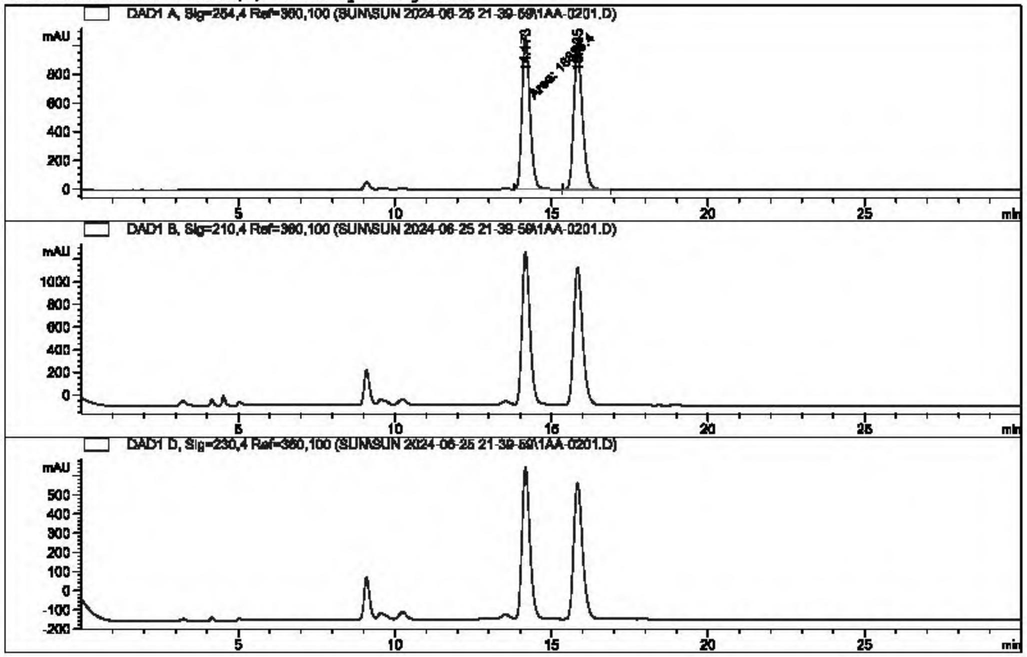

Area Percent Report

Sorted By	:	Signal
Multiplier	:	1.0000
Dilution	:	1.0000

Use Multiplier & Dilution Factor with ISTDs

Signal 1: DAD1 A, Sig=254,4 Ref=360,100

Peak #	RetTime [min]	Type	Width [min]	Area [mAU*s]	Height [mAU]	Area %
1	14.173	FM	0.2953	1.88854e4	1065.95264	49.9056
2	15.835	BB	0.3054	1.89569e4	957.87402	50.0944

Data File C:\Users\P...Documents\ChemStation\1\Data\SUN\SUN 2024-06-25 21-39-59\1AA-0201.D
Sample Name: zzj-4-38-15-rac

Peak #	RetTime [min]	Type	Width [min]	Area [mAU*s]	Height [mAU]	Area %
Totals :				3.78423e4	2023.82666	

Signal 2: DAD1 B, Sig=210,4 Ref=360,100

Signal 3: DAD1 D, Sig=230,4 Ref=360,100

*** End of Report ***

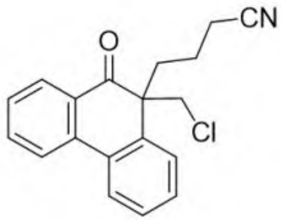

3e-rac

Data File C:\Users\P...Documents\ChemStation\1\Data\SUN\SUN 2024-04-08 22-14-29\1CA-0201.D
Sample Name: zzj-3-157

```
=====================================================================
Acq. Operator   : SYSTEM                        Seq. Line :    2
Sample Operator : SYSTEM
Acq. Instrument : HPLC                           Location :   P1-C-01
Injection Date  : 8/4/2024 10:27:39 pm                Inj :    1
                                               Inj Volume : 2.000 µl
Different Inj Volume from Sample Entry!  Actual Inj Volume : 25.000 µl
Method          : C:\Users\Public\Documents\ChemStation\1\Data\SUN\SUN 2024-04-08 22-14-29\IE3-
                  20-30.M (Sequence Method)
Last changed    : 21/9/2022 10:09:09 am by SYSTEM
Additional Info : Peak(s) manually integrated
```

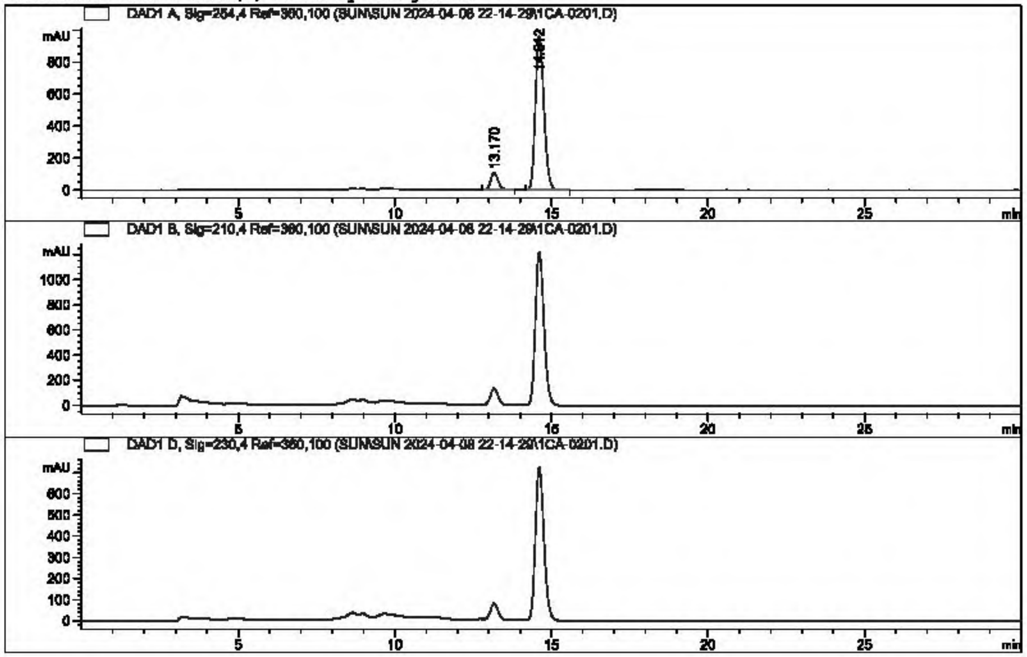


```
=====================================================================
                         Area Percent Report
=====================================================================

Sorted By             :      Signal
Multiplier            :      1.0000
Dilution              :      1.0000
Use Multiplier & Dilution Factor with ISTDs
```

Signal 1: DAD1 A, Sig=254,4 Ref=360,100

Peak #	RetTime [min]	Type	Width [min]	Area [mAU*s]	Height [mAU]	Area %
1	13.170	BB	0.2666	1769.68811	104.72842	8.8188
2	14.612	BB	0.2968	1.82974e4	960.39771	91.1812

Data File C:\Users\P...Documents\ChemStation\1\Data\SUN\SUN 2024-04-08 22-14-29\1CA-0201.D
Sample Name: zzj-3-157

Peak #	RetTime [min]	Type	Width [min]	Area [mAU*s]	Height [mAU]	Area %
Totals :				2.00671e4	1065.12612	

Signal 2: DAD1 B, Sig=210,4 Ref=360,100

Signal 3: DAD1 D, Sig=230,4 Ref=360,100

*** End of Report ***

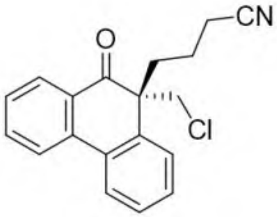

3e

```
Acq. Operator   : SYSTEM                        Seq. Line :  12
Sample Operator : SYSTEM
Acq. Instrument : HPLC                           Location :   P1-B-04
Injection Date  : 10/7/2024 12:52:39 am               Inj :   1
                                               Inj Volume : 2.000 µl
Different Inj Volume from Sample Entry!  Actual Inj Volume : 1.000 µl
Method          : C:\Users\Public\Documents\ChemStation\1\Data\SUN\SUN 2024-07-09 22-34-11\IBN3-
                  05-10.M (Sequence Method)
Last changed    : 31/7/2023 10:33:52 am by SYSTEM
Additional Info : Peak(s) manually integrated
```

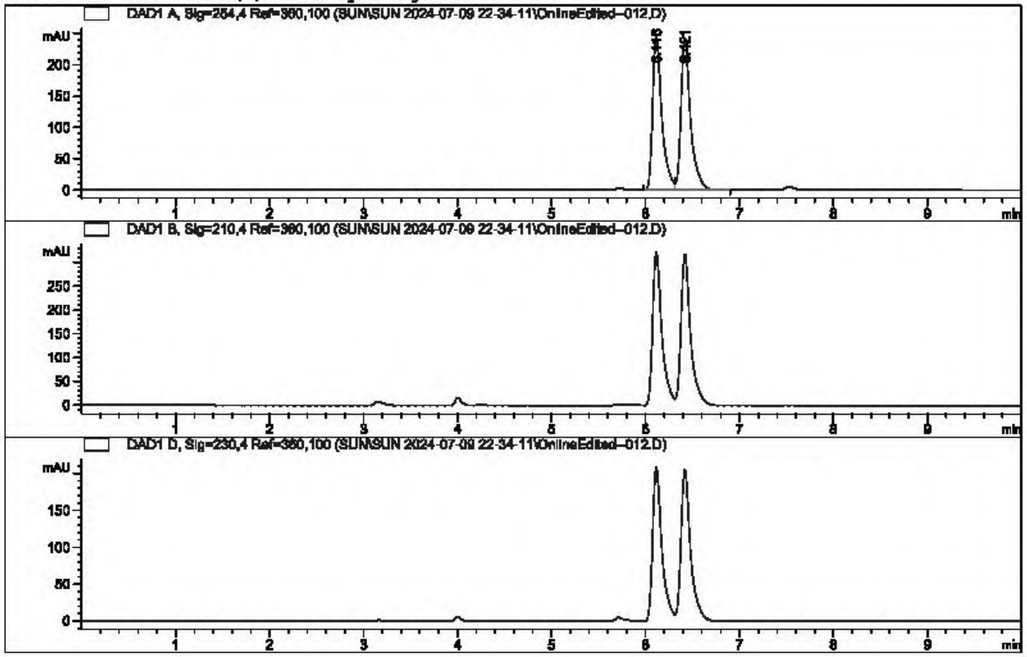

Area Percent Report

```
Sorted By             :      Signal
Multiplier            :      1.0000
Dilution              :      1.0000
Use Multiplier & Dilution Factor with ISTDs
```

Signal 1: DAD1 A, Sig=254,4 Ref=360,100

Peak #	RetTime [min]	Type	Width [min]	Area [mAU*s]	Height [mAU]	Area %
1	6.116	BV	0.1017	1676.24377	245.35626	49.3436
2	6.421	VB	0.1057	1720.84290	241.37105	50.6564

Data File C:\Users\P...\ChemStation\1\Data\SUN\SUN 2024-07-09 22-34-11\OnlineEdited--012.D
Sample Name: zzj-4-38-19

Peak #	RetTime [min]	Type	Width [min]	Area [mAU*s]	Height [mAU]	Area %
Totals :				3397.08667	486.72731	

Signal 2: DAD1 B, Sig=210,4 Ref=360,100

Signal 3: DAD1 D, Sig=230,4 Ref=360,100

*** End of Report ***

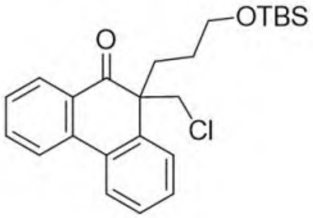

3f-rac

Data File C:\Users\P...\ChemStation\1\Data\SUN\SUN 2024-02-29 20-23-39\OnlineEdited--006.D
Sample Name: zzj-3-107

```
=====================================================================
Acq. Operator   : SYSTEM                        Seq. Line :   6
Sample Operator : SYSTEM
Acq. Instrument : HPLC                           Location :   P1-F-05
Injection Date  : 29/2/2024 9:52:02 pm                Inj :   1
                                               Inj Volume : 2.000 µl
Different Inj Volume from Sample Entry!  Actual Inj Volume : 25.000 µl
Method          : C:\Users\Public\Documents\ChemStation\1\Data\SUN\SUN 2024-02-29 20-23-39\IBN3-
                  05-10.M (Sequence Method)
Last changed    : 31/7/2023 10:33:52 am by SYSTEM
Additional Info : Peak(s) manually integrated
```

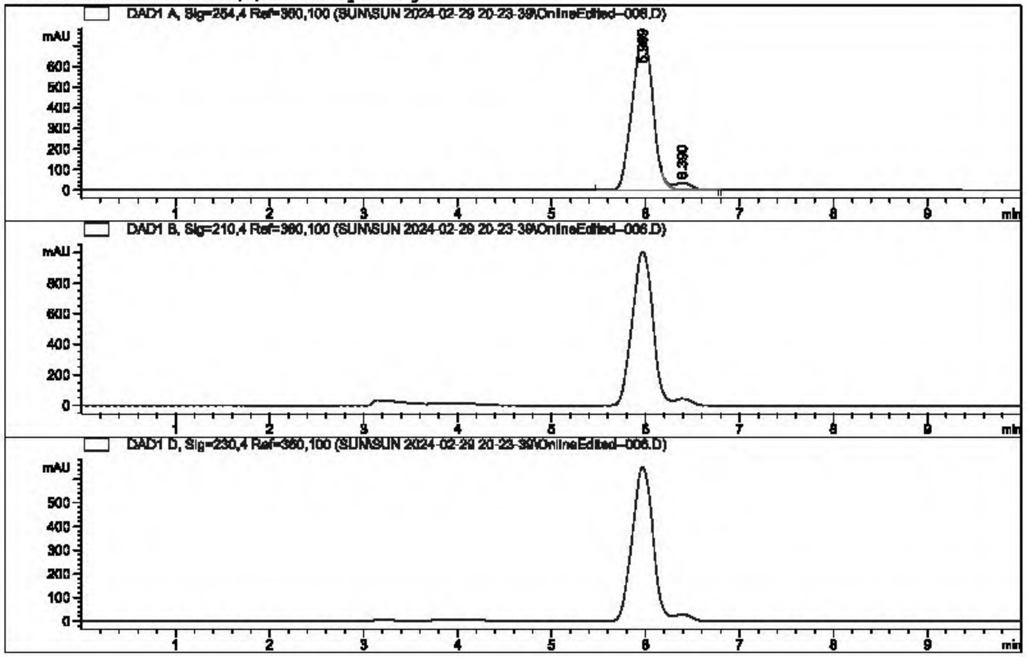


```
=====================================================================
                         Area Percent Report
=====================================================================

Sorted By             :      Signal
Multiplier            :      1.0000
Dilution              :      1.0000
Use Multiplier & Dilution Factor with ISTDs
```

Signal 1: DAD1 A, Sig=254,4 Ref=360,100

Peak #	RetTime [min]	Type	Width [min]	Area [mAU*s]	Height [mAU]	Area %
1	5.969	BV R	0.2370	1.14746e4	747.85388	96.2743
2	6.390	VV E	0.1829	444.04669	33.54881	3.7257

Data File C:\Users\P...\ChemStation\1\Data\SUN\SUN 2024-02-29 20-23-39\OnlineEdited--006.D
Sample Name: zzj-3-107

Peak #	RetTime [min]	Type	Width [min]	Area [mAU*s]	Height [mAU]	Area %
Totals :				1.19186e4	781.40269	

Signal 2: DAD1 B, Sig=210,4 Ref=360,100

Signal 3: DAD1 D, Sig=230,4 Ref=360,100

```
=====================================================================
```

*** End of Report ***

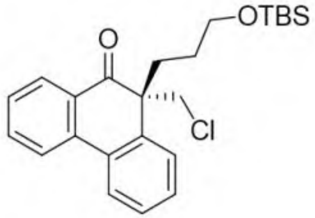

3f

Data File C:\Users\P...Documents\ChemStation\1\Data\SUN\SUN 2024-07-10 23-10-40\1AB-0201.D
Sample Name: zzj-4-38-20

Acq. Operator : SYSTEM   Seq. Line : 2
Sample Operator : SYSTEM
Acq. Instrument : HPLC   Location : P1-A-02
Injection Date : 10/7/2024 11:23:46 pm   Inj : 1
Inj Volume : 2.000 µl
Different Inj Volume from Sample Entry! Actual Inj Volume : 1.000 µl
Method : C:\Users\Public\Documents\ChemStation\1\Data\SUN\SUN 2024-07-10 23-10-40\OD3-10-20.M (Sequence Method)
Last changed : 15/8/2022 10:27:52 pm by SYSTEM
Additional Info : Peak(s) manually integrated

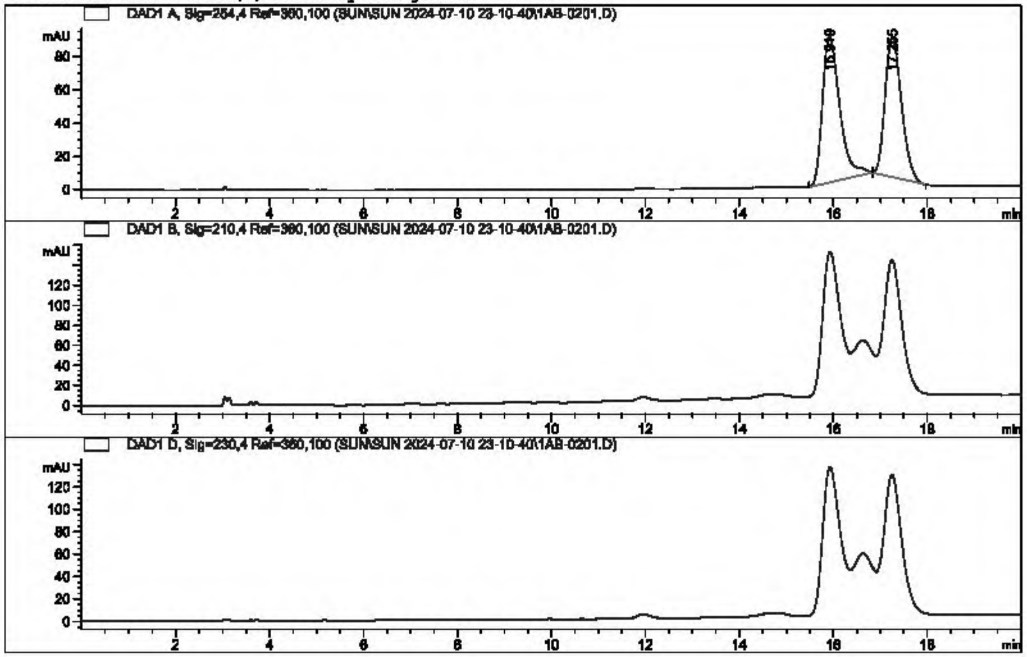

Area Percent Report

Sorted By : Signal
Multiplier : 1.0000
Dilution : 1.0000
Use Multiplier & Dilution Factor with ISTDs

Signal 1: DAD1 A, Sig=254,4 Ref=360,100

Peak #	RetTime [min]	Type	Width [min]	Area [mAU*s]	Height [mAU]	Area %
1	15.919	BB	0.3852	2239.81934	87.63538	52.3117
2	17.255	BB	0.3724	2041.85706	84.19557	47.6883

Data File C:\Users\P...Documents\ChemStation\1\Data\SUN\SUN 2024-07-10 23-10-40\1AB-0201.D
Sample Name: zzj-4-38-20

Peak #	RetTime [min]	Type	Width [min]	Area [mAU*s]	Height [mAU]	Area %
Totals :				4281.67639	171.83095	

Signal 2: DAD1 B, Sig=210,4 Ref=360,100

Signal 3: DAD1 D, Sig=230,4 Ref=360,100

*** End of Report ***

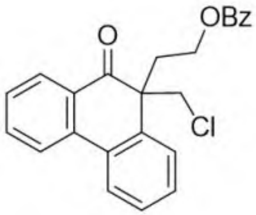

3g-rac

Data File C:\Users\P...\ChemStation\1\Data\SUN\SUN 2024-04-24 10-12-35\OnlineEdited--014.D
Sample Name: zzj-3-179

===

Acq. Operator : SYSTEM Seq. Line : 14
Sample Operator : SYSTEM
Acq. Instrument : HPLC Location : P1-B-02
Injection Date : 24/4/2024 1:59:09 pm Inj : 1
Inj Volume : 2.000 µl
Different Inj Volume from Sample Entry! Actual Inj Volume : 25.000 µl
Method : C:\Users\Public\Documents\ChemStation\1\Data\SUN\SUN 2024-04-24 10-12-35\OD3-10-20.M (Sequence Method)
Last changed : 15/8/2022 10:27:52 pm by SYSTEM
Additional Info : Peak(s) manually integrated

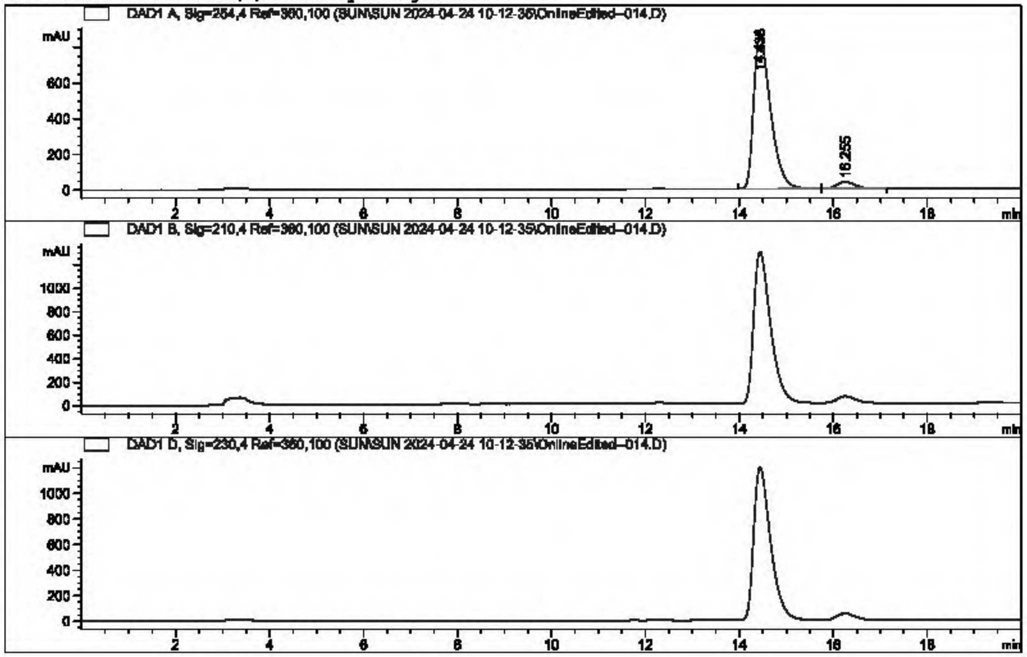

===

Area Percent Report

===

Sorted By : Signal
Multiplier : 1.0000
Dilution : 1.0000
Use Multiplier & Dilution Factor with ISTDs

Signal 1: DAD1 A, Sig=254,4 Ref=360,100

Peak #	RetTime [min]	Type	Width [min]	Area [mAU*s]	Height [mAU]	Area %
1	14.436	BB	0.3797	2.13594e4	852.95215	95.7182
2	16.255	BB	0.3790	955.48431	36.85534	4.2818

Data File C:\Users\P...\ChemStation\1\Data\SUN\SUN 2024-04-24 10-12-35\OnlineEdited--014.D
Sample Name: zzj-3-179

Peak #	RetTime [min]	Type	Width [min]	Area [mAU*s]	Height [mAU]	Area %
Totals :				2.23149e4	889.80748	

Signal 2: DAD1 B, Sig=210,4 Ref=360,100

Signal 3: DAD1 D, Sig=230,4 Ref=360,100

===

*** End of Report ***

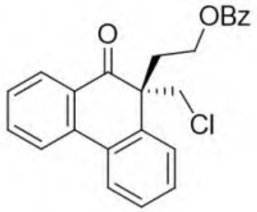

3g

Data File C:\Users\P...Documents\ChemStation\1\Data\SUN\SUN 2024-06-25 21-39-59\1AB-0401.D
Sample Name: zzj-4-38-16-rac

===

Acq. Operator : SYSTEM Seq. Line : 4
Sample Operator : SYSTEM
Acq. Instrument : HPLC Location : P1-A-02
Injection Date : 25/6/2024 10:35:51 pm Inj : 1
Inj Volume : 2.000 µl
Different Inj Volume from Sample Entry! Actual Inj Volume : 5.000 µl
Acq. Method : C:\Users\Public\Documents\ChemStation\1\Data\SUN\SUN 2024-06-25 21-39-59\OD3-20-30.M
Last changed : 4/1/2023 3:52:32 pm by SYSTEM
Analysis Method : C:\Users\Public\Documents\ChemStation\1\Data\SUN\SUN 2024-06-25 21-39-59\OD3-20-30.M (Sequence Method)
Last changed : 13/12/2025 2:17:48 pm by SYSTEM
(modified after loading)
Additional Info : Peak(s) manually integrated

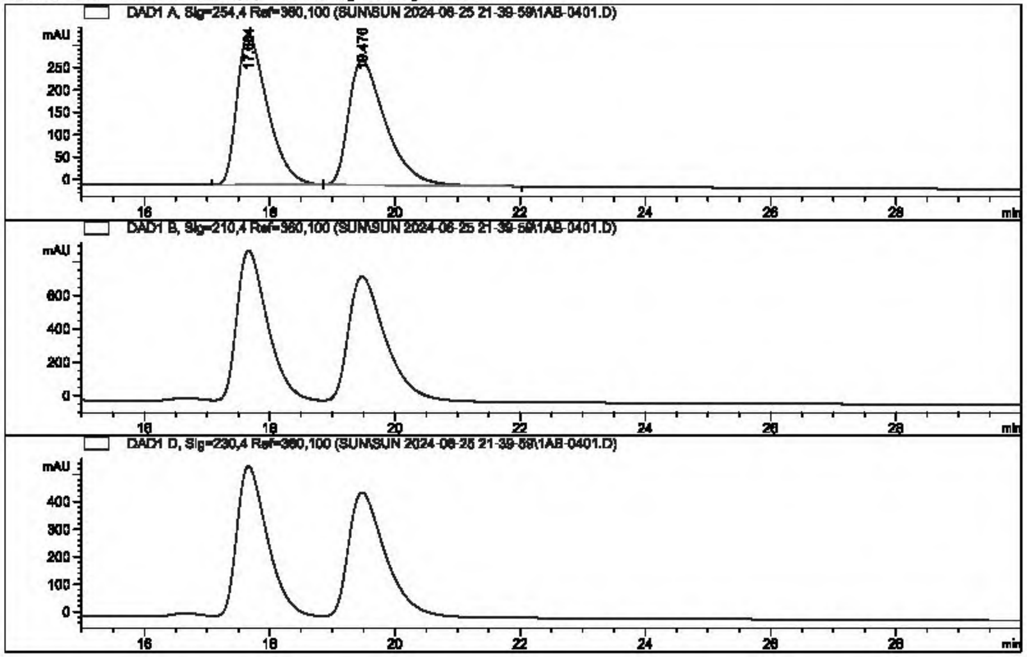

===

Area Percent Report

===

Sorted By : Signal
Multiplier : 1.0000
Dilution : 1.0000
Use Multiplier & Dilution Factor with ISTDs

Data File C:\Users\P...Documents\ChemStation\1\Data\SUN\SUN 2024-06-25 21-39-59\1AB-0401.D
Sample Name: zzj-4-38-16-rac

Signal 1: DAD1 A, Sig=254,4 Ref=360,100

Peak #	RetTime [min]	Type	Width [min]	Area [mAU*s]	Height [mAU]	Area %
1	17.664	BB	0.5106	1.13345e4	335.08646	49.9008
2	19.476	BB	0.5997	1.13796e4	277.64941	50.0992
Totals :				2.27141e4	612.73587	

Signal 2: DAD1 B, Sig=210,4 Ref=360,100

Signal 3: DAD1 D, Sig=230,4 Ref=360,100

*** End of Report ***

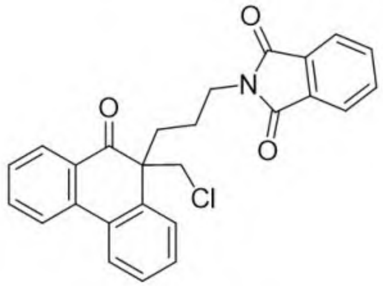

3h-rac

Data File C:\Users\P...Documents\ChemStation\1\Data\SUN\SUN 2024-03-26 22-28-37\1CE-0201.D
Sample Name: zzj-3-147

```
=====================================================================
Acq. Operator   : SYSTEM                        Seq. Line :   2
Sample Operator : SYSTEM
Acq. Instrument : HPLC                           Location :   P1-C-05
Injection Date  : 26/3/2024 10:41:27 pm               Inj :   1
                                               Inj Volume : 2.000 µl
Different Inj Volume from Sample Entry!  Actual Inj Volume : 25.000 µl
Acq. Method     : C:\Users\Public\Documents\ChemStation\1\Data\SUN\SUN 2024-03-26 22-28-37\OD3-
                  20-30.M
Last changed    : 4/1/2023 3:52:32 pm by SYSTEM
Analysis Method : C:\Users\Public\Documents\ChemStation\1\Data\SUN\SUN 2024-03-26 22-28-37\OD3-
                  20-30.M (Sequence Method)
Last changed    : 13/12/2025 2:19:08 pm by SYSTEM
                  (modified after loading)
Additional Info : Peak(s) manually integrated
```

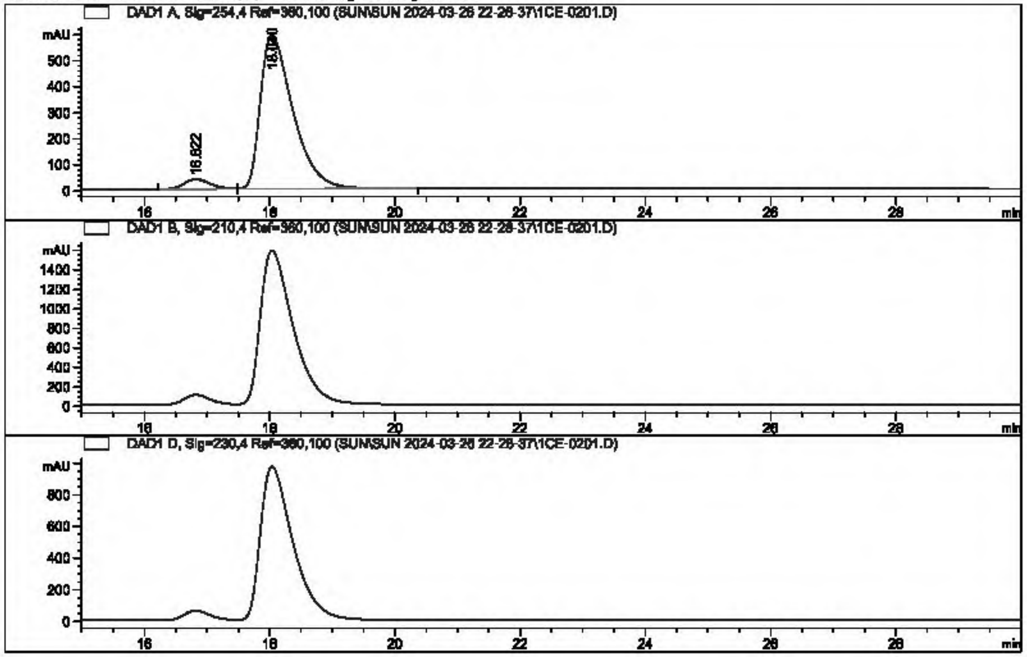


```
=====================================================================
                         Area Percent Report
=====================================================================

Sorted By             :      Signal
Multiplier            :      1.0000
Dilution              :      1.0000
Use Multiplier & Dilution Factor with ISTDs
```


Data File C:\Users\P...Documents\ChemStation\1\Data\SUN\SUN 2024-03-26 22-28-37\1CE-0201.D
Sample Name: zzj-3-147

Signal 1: DAD1 A, Sig=254,4 Ref=360,100

Peak #	RetTime [min]	Type	Width [min]	Area [mAU*s]	Height [mAU]	Area %
1	16.822	BV	0.4257	1124.99133	38.41579	4.9838
2	18.040	VB	0.5351	2.14479e4	591.43933	95.0162
Totals :				2.25729e4	629.85512	

Signal 2: DAD1 B, Sig=210,4 Ref=360,100

Signal 3: DAD1 D, Sig=230,4 Ref=360,100

*** End of Report ***

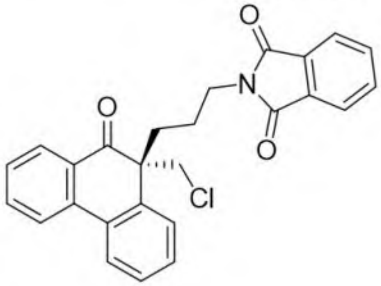

3h

Data File C:\Users\P...Documents\ChemStation\1\Data\SUN\SUN 2024-05-30 22-46-55\1DB-0401.D
Sample Name: zzj-4-21-8-rac

Acq. Operator	: SYSTEM	Seq. Line :	4
Sample Operator	: SYSTEM		
Acq. Instrument	: HPLC	Location :	P1-D-02
Injection Date	: 30/5/2024 11:32:06 pm	Inj :	1
		Inj Volume :	2.000 µl

Different Inj Volume from Sample Entry! Actual Inj Volume : 1.000 µl
Method : C:\Users\Public\Documents\ChemStation\1\Data\SUN\SUN 2024-05-30 22-46-55\AD3-05-20.M (Sequence Method)
Last changed : 19/3/2023 10:16:19 pm by SYSTEM
Additional Info : Peak(s) manually integrated

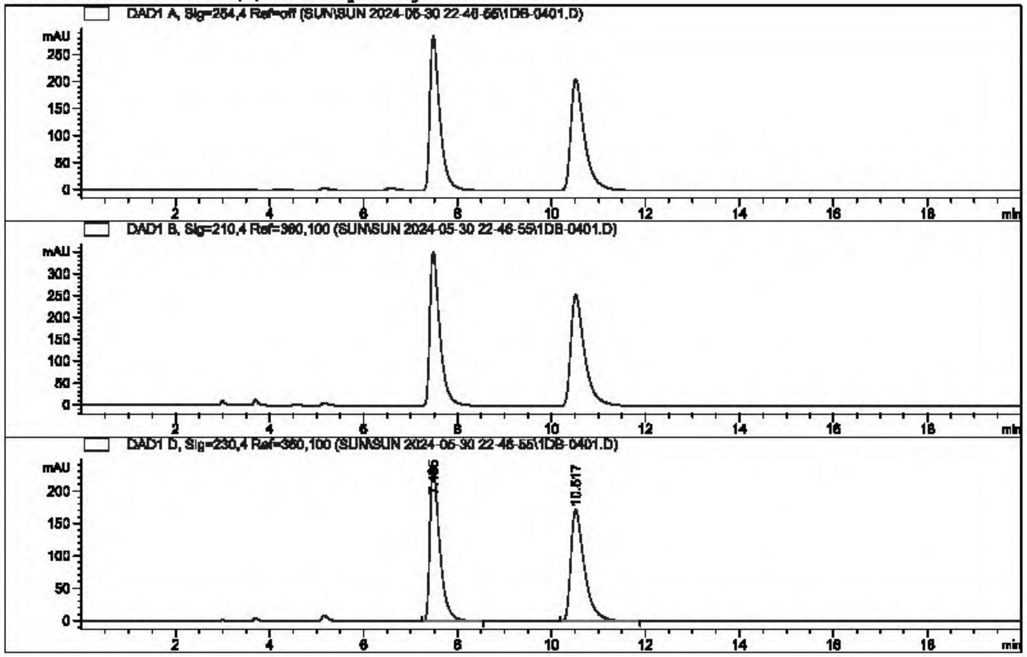

Area Percent Report

Sorted By : Signal
Multiplier : 1.0000
Dilution : 1.0000
Use Multiplier & Dilution Factor with ISTDs

Signal 1: DAD1 A, Sig=254,4 Ref=off

Data File C:\Users\P...Documents\ChemStation\1\Data\SUN\SUN 2024-05-30 22-46-55\1DB-0401.D
Sample Name: zzj-4-21-8-rac

Signal 2: DAD1 B, Sig=210,4 Ref=360,100

Signal 3: DAD1 D, Sig=230,4 Ref=360,100

Peak #	RetTime [min]	Type	Width [min]	Area [mAU*s]	Height [mAU]	Area %
1	7.485	BB	0.2166	3447.78467	237.14478	50.0331
2	10.517	BB	0.3004	3443.22656	171.40492	49.9669
Totals :				6891.01123	408.54970	

*** End of Report ***

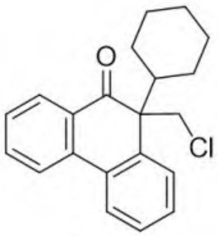

3i-rac

```
=====================================================================
Acq. Operator   : SYSTEM                         Seq. Line :  15
Sample Operator : SYSTEM
Acq. Instrument : HPLC                            Location :  P1-A-01
Injection Date  : 2/1/2024 2:19:25 am                  Inj :  1
                                               Inj Volume : 2.000 µl
Different Inj Volume from Sample Entry!  Actual Inj Volume : 25.000 µl
Method          : C:\Users\Public\Documents\ChemStation\1\Data\SUN\SUN 2024-01-01 21-21-18\AD3-
                  05-20.M (Sequence Method)
Last changed    : 19/3/2023 10:16:19 pm by SYSTEM
Additional Info : Peak(s) manually integrated
```

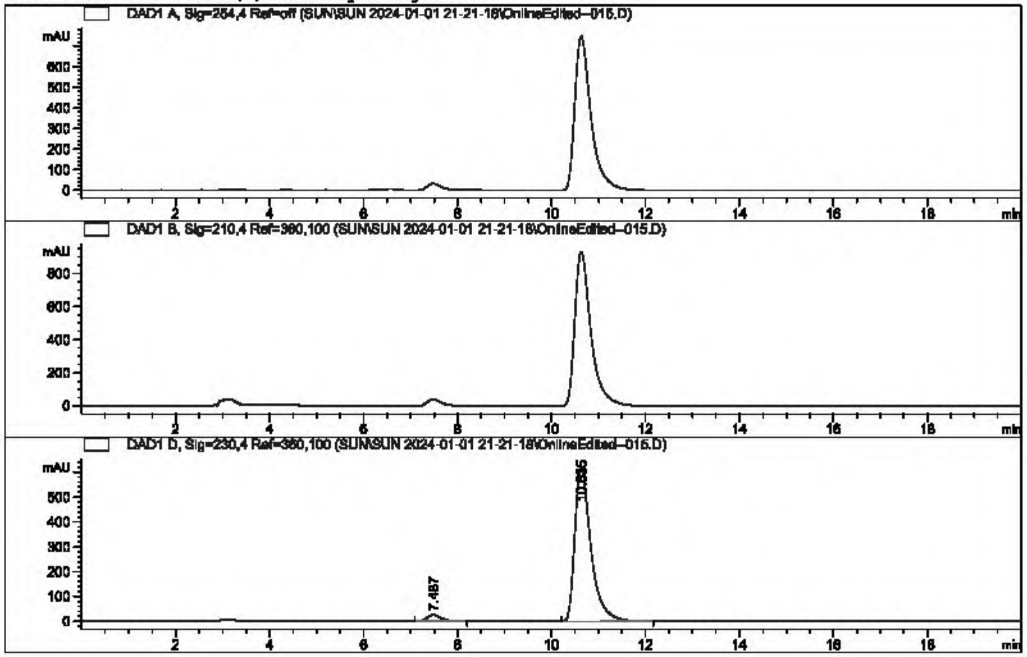


```
=====================================================================
                         Area Percent Report
=====================================================================

Sorted By             :      Signal
Multiplier            :      1.0000
Dilution              :      1.0000
Use Multiplier & Dilution Factor with ISTDs


Signal 1: DAD1 A, Sig=254,4 Ref=off
```


Data File C:\Users\P...\ChemStation\1\Data\SUN\SUN 2024-01-01 21-21-18\OnlineEdited--015.D
Sample Name: zzj-3-52

Signal 2: DAD1 B, Sig=210,4 Ref=360,100

Signal 3: DAD1 D, Sig=230,4 Ref=360,100

Peak #	RetTime [min]	Type	Width [min]	Area [mAU*s]	Height [mAU]	Area %
1	7.487	BB	0.2416	530.21179	25.98433	3.4694
2	10.635	BV R	0.3615	1.47522e4	619.13037	96.5306

Totals : 1.52824e4 645.11470

*** End of Report ***

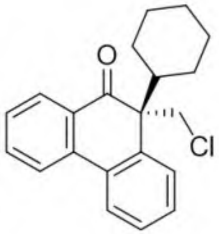

3i

Data File C:\Users\P...\ChemStation\1\Data\SUN\SUN 2024-07-03 14-56-28\OnlineEdited--016.D
Sample Name: zzj-4-38-18-rac

Acq. Operator : SYSTEM   Seq. Line : 16
Sample Operator : SYSTEM
Acq. Instrument : HPLC   Location : P1-D-10
Injection Date : 3/7/2024 7:19:53 pm   Inj : 1
Inj Volume : 2.000 µl
Method : C:\Users\Public\Documents\ChemStation\1\Data\SUN\SUN 2024-07-03 14-56-28\AD3-10-20.M (Sequence Method)
Last changed : 15/8/2022 10:21:32 pm by SYSTEM
Additional Info : Peak(s) manually integrated

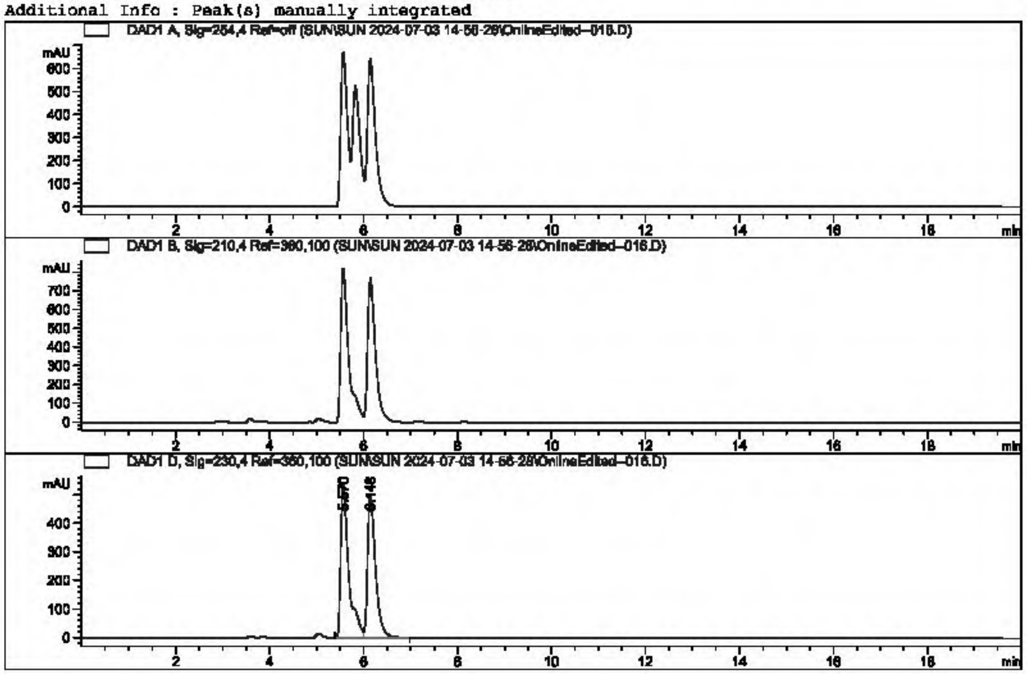

Area Percent Report

Sorted By : Signal
Multiplier : 1.0000
Dilution : 1.0000
Use Multiplier & Dilution Factor with ISTDs

Signal 1: DAD1 A, Sig=254,4 Ref=off

Data File C:\Users\P...\ChemStation\1\Data\SUN\SUN 2024-07-03 14-56-28\OnlineEdited--016.D
Sample Name: zzj-4-38-18-rac

Signal 2: DAD1 B, Sig=210,4 Ref=360,100

Signal 3: DAD1 D, Sig=230,4 Ref=360,100

Peak #	RetTime [min]	Type	Width [min]	Area [mAU*s]	Height [mAU]	Area %
1	5.570	BV	0.1733	6347.91943	537.46118	52.0144
2	6.148	VB	0.1733	5856.25000	503.12961	47.9856

Totals : 1.22042e4 1040.59079

*** End of Report ***

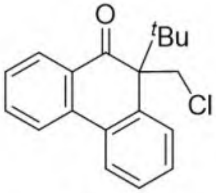

3j-rac

```
Acq. Operator   : SYSTEM                        Seq. Line :   2
Sample Operator : SYSTEM
Acq. Instrument : HPLC                           Location :   P1-B-01
Injection Date  : 13/3/2024 10:25:01 pm               Inj :   1
                                               Inj Volume : 2.000 µl
Different Inj Volume from Sample Entry!  Actual Inj Volume : 25.000 µl
Method          : C:\Users\Public\Documents\ChemStation\1\Data\SUN\SUN 2024-03-13 22-11-38\AD3-
                  10-20.M (Sequence Method)
Last changed    : 15/8/2022 10:21:32 pm by SYSTEM
Additional Info : Peak(s) manually integrated
```

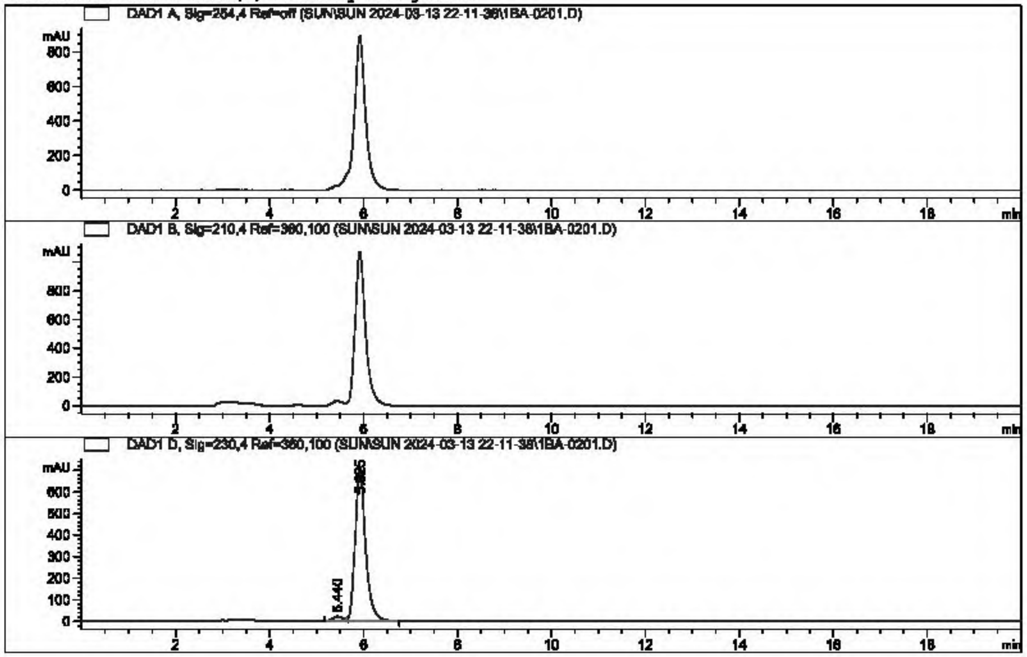

Area Percent Report

```
Sorted By             :      Signal
Multiplier            :      1.0000
Dilution              :      1.0000
Use Multiplier & Dilution Factor with ISTDs
```

Signal 1: DAD1 A, Sig=254,4 Ref=off

Data File C:\Users\P...Documents\ChemStation\1\Data\SUN\SUN 2024-03-13 22-11-38\1BA-0201.D
Sample Name: zzj-3-130

Signal 2: DAD1 B, Sig=210,4 Ref=360,100

Signal 3: DAD1 D, Sig=230,4 Ref=360,100

Peak #	RetTime [min]	Type	Width [min]	Area [mAU*s]	Height [mAU]	Area %
1	5.440	BV E	0.1898	351.72720	21.96917	3.1094
2	5.925	VV R	0.2199	1.09601e4	712.88519	96.8906
Totals :				1.13118e4	734.85437	

*** End of Report ***

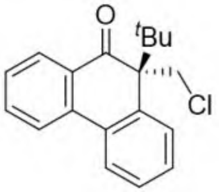

3j

Data File C:\Users\P...Documents\ChemStation\1\Data\SUN\SUN 2024-07-10 09-46-42\1BE-0201.D
Sample Name: zzj-4-38-22

```
Acq. Operator   : SYSTEM                    Seq. Line :   2
Sample Operator : SYSTEM
Acq. Instrument : HPLC                       Location :   P1-B-05
Injection Date  : 10/7/2024 9:59:48 am            Inj :   1
                                           Inj Volume : 2.000 µl
Different Inj Volume from Sample Entry!  Actual Inj Volume : 10.000 µl
Method          : C:\Users\Public\Documents\ChemStation\1\Data\SUN\SUN 2024-07-10 09-46-42\AS3-
                  05-15.M (Sequence Method)
Last changed    : 6/4/2023 9:18:17 pm by SYSTEM
Additional Info : Peak(s) manually integrated
```

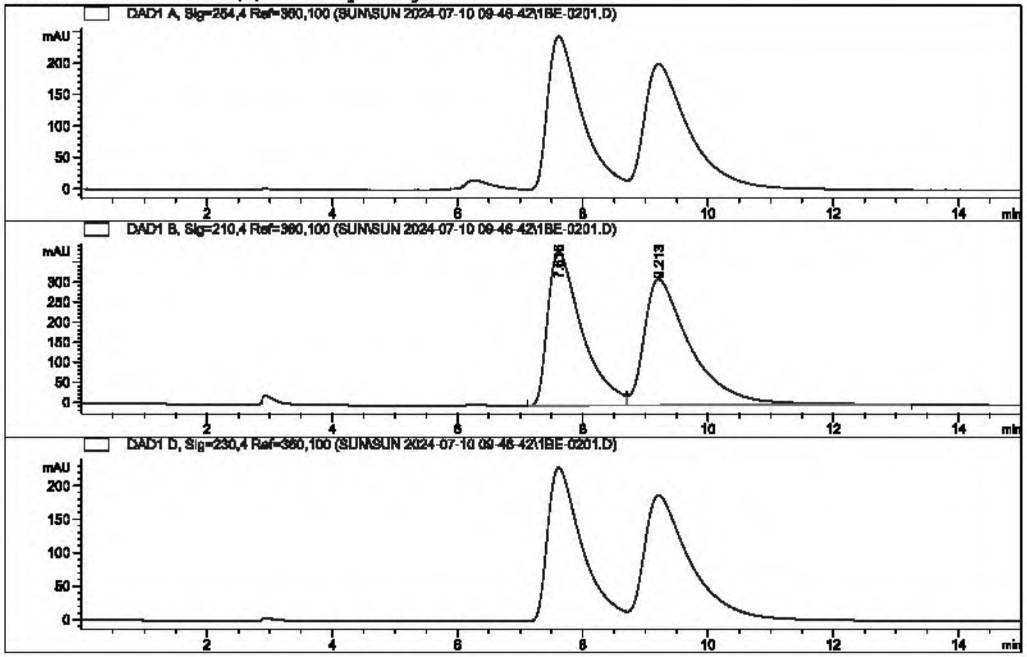

Area Percent Report

```
Sorted By          :      Signal
Multiplier         :      1.0000
Dilution           :      1.0000
Use Multiplier & Dilution Factor with ISTDs
```

Signal 1: DAD1 A, Sig=254,4 Ref=360,100

Data File C:\Users\P...Documents\ChemStation\1\Data\SUN\SUN 2024-07-10 09-46-42\1BE-0201.D
Sample Name: zzj-4-38-22

Signal 2: DAD1 B, Sig=210,4 Ref=360,100

Peak #	RetTime [min]	Type	Width [min]	Area [mAU*s]	Height [mAU]	Area %
1	7.616	BV	0.5470	1.45227e4	383.73749	47.1956
2	9.213	VB	0.7097	1.62486e4	314.28784	52.8044
Totals :				3.07713e4	698.02533	

Signal 3: DAD1 D, Sig=230,4 Ref=360,100

*** End of Report ***

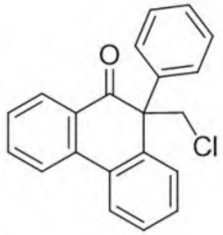

3k-rac

Acq. Operator : SYSTEM Seq. Line : 6
Sample Operator : SYSTEM
Acq. Instrument : HPLC Location : P1-A-02
Injection Date : 6/12/2023 11:27:01 am Inj : 1
Inj Volume : 2.000 µl
Different Inj Volume from Sample Entry! Actual Inj Volume : 25.000 µl
Method : C:\Users\Public\Documents\ChemStation\1\Data\SUN\SUN 2023-12-06 09-39-47\AS3-05-15.M (Sequence Method)
Last changed : 6/4/2023 9:18:17 pm by SYSTEM
Additional Info : Peak(s) manually integrated

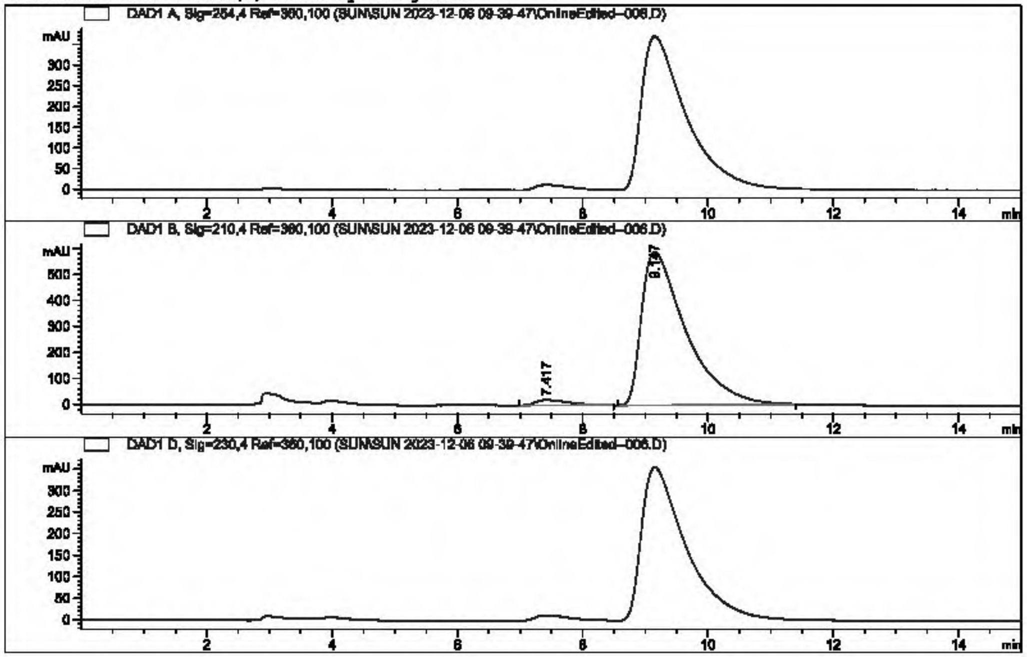

Area Percent Report

Sorted By : Signal
Multiplier : 1.0000
Dilution : 1.0000
Use Multiplier & Dilution Factor with ISTDs

Signal 1: DAD1 A, Sig=254,4 Ref=360,100

Data File C:\Users\P...\ChemStation\1\Data\SUN\SUN 2023-12-06 09-39-47\OnlineEdited--006.D
Sample Name: zzj-3-2

Signal 2: DAD1 B, Sig=210,4 Ref=360,100

Peak #	RetTime [min]	Type	Width [min]	Area [mAU*s]	Height [mAU]	Area %
1	7.417	VV R	0.4272	762.71722	21.02819	2.5763
2	9.147	VV R	0.5736	2.88421e4	588.70667	97.4237

Totals : 2.96049e4 609.73485

Signal 3: DAD1 D, Sig=230,4 Ref=360,100

*** End of Report ***

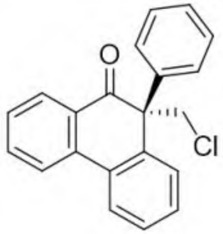

3k

Acq. Operator : SYSTEM Seq. Line : 4
Sample Operator : SYSTEM
Acq. Instrument : HPLC Location : P1-A-04
Injection Date : 21/5/2024 4:10:28 pm Inj : 1
Inj Volume : 2.000 µl
Different Inj Volume from Sample Entry! Actual Inj Volume : 25.000 µl
Method : C:\Users\Public\Documents\ChemStation\1\Data\SUN\SUN 2024-05-21 15-25-06\AD3-10-30.M (Sequence Method)
Last changed : 24/10/2022 9:57:21 am by SYSTEM
Additional Info : Peak(s) manually integrated

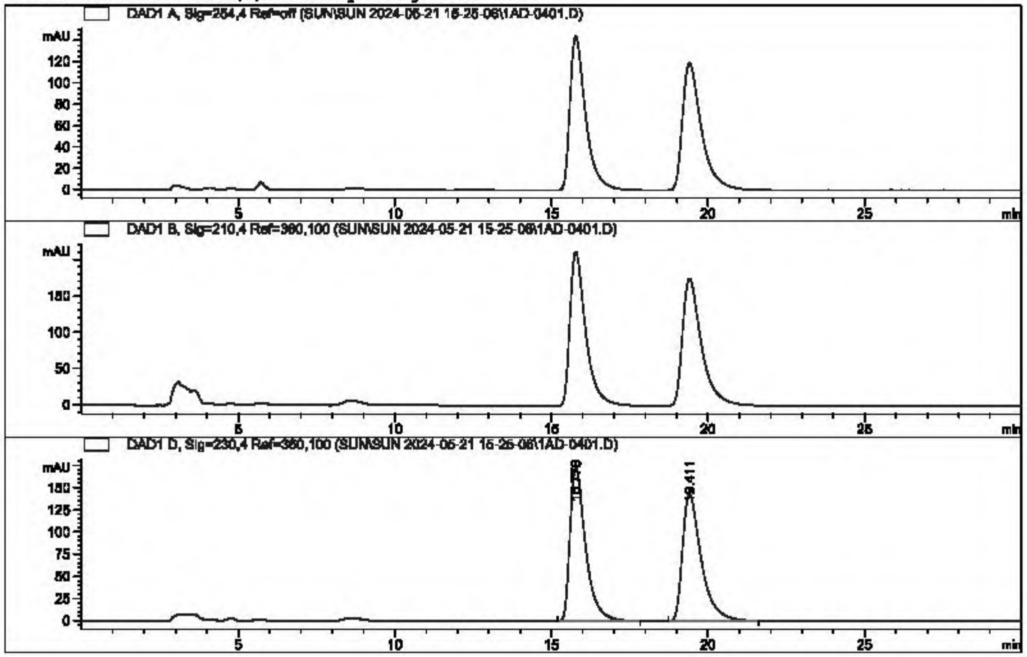

Area Percent Report

Sorted By : Signal
Multiplier : 1.0000
Dilution : 1.0000
Use Multiplier & Dilution Factor with ISTDs

Signal 1: DAD1 A, Sig=254,4 Ref=off

Data File C:\Users\P...Documents\ChemStation\1\Data\SUN\SUN 2024-05-21 15-25-06\1AD-0401.D
Sample Name: zzj-4-18-2

Signal 2: DAD1 B, Sig=210,4 Ref=360,100

Signal 3: DAD1 D, Sig=230,4 Ref=360,100

Peak #	RetTime [min]	Type	Width [min]	Area [mAU*s]	Height [mAU]	Area %
1	15.776	BB	0.5111	6009.18213	173.27591	50.1590
2	19.411	BB	0.6133	5971.07373	142.95880	49.8410

Totals : 1.19803e4 316.23471

*** End of Report ***

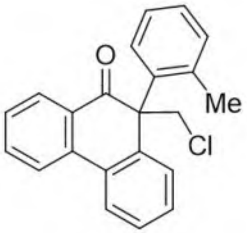

3l-rac

```
=====================================================================
Acq. Operator   : SYSTEM                       Seq. Line :  14
Sample Operator : SYSTEM
Acq. Instrument : HPLC                          Location :  P1-F-01
Injection Date  : 10/1/2024 4:45:25 pm               Inj :  1
                                             Inj Volume : 2.000 µl
Different Inj Volume from Sample Entry!  Actual Inj Volume : 25.000 µl
Method          : C:\Users\Public\Documents\ChemStation\1\Data\SUN\SUN 2024-01-10 12-24-55\AD3-
                  10-30.M (Sequence Method)
Last changed    : 24/10/2022 9:57:21 am by SYSTEM
Additional Info : Peak(s) manually integrated
```

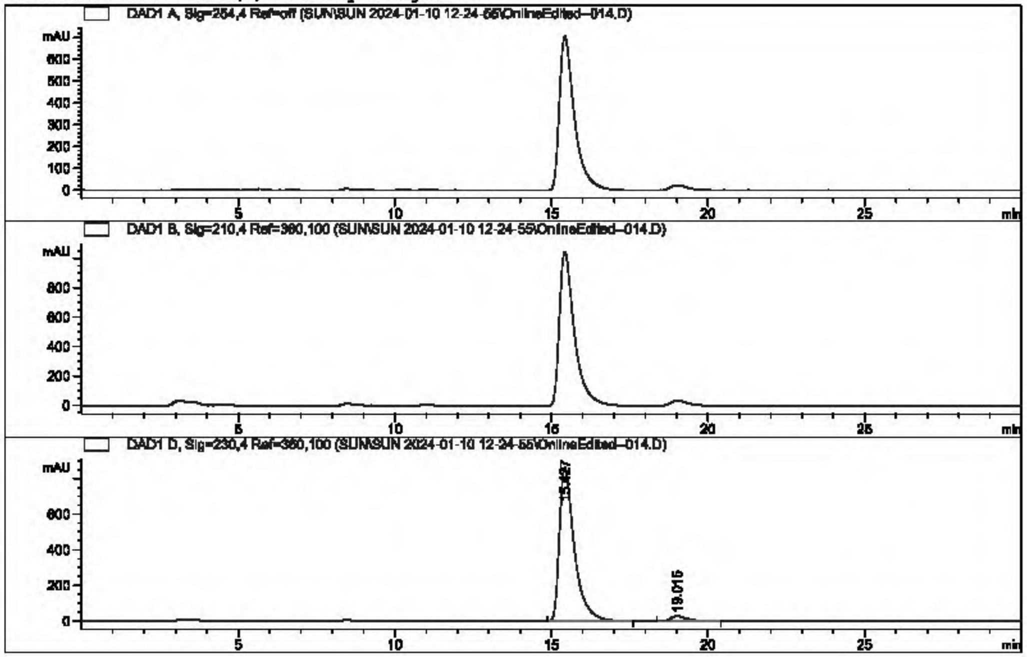


```
=====================================================================
                         Area Percent Report
=====================================================================

Sorted By             :      Signal
Multiplier            :      1.0000
Dilution              :      1.0000
Use Multiplier & Dilution Factor with ISTDs


Signal 1: DAD1 A, Sig=254,4 Ref=off
```


Data File C:\Users\P...\ChemStation\1\Data\SUN\SUN 2024-01-10 12-24-55\OnlineEdited--014.D
Sample Name: zzj-3-67

Signal 2: DAD1 B, Sig=210,4 Ref=360,100

Signal 3: DAD1 D, Sig=230,4 Ref=360,100

Peak #	RetTime [min]	Type	Width [min]	Area [mAU*s]	Height [mAU]	Area %
1	15.427	BV R	0.4607	2.87154e4	864.08649	96.3563
2	19.015	BB	0.4510	1085.88281	28.31257	3.6437

Totals : 2.98013e4 892.39906

*** End of Report ***

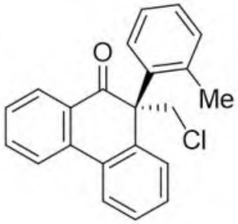

3l

Data File C:\Users\P...Documents\ChemStation\1\Data\SUN\SUN 2024-05-29 12-03-40\1DD-0801.D
Sample Name: zzj-4-18-3-rac-as

```
=====================================================================
Acq. Operator   : SYSTEM                       Seq. Line :   8
Sample Operator : SYSTEM
Acq. Instrument : HPLC                          Location :   P1-D-04
Injection Date  : 29/5/2024 2:08:40 pm               Inj :   1
                                              Inj Volume : 2.000 µl
Different Inj Volume from Sample Entry!  Actual Inj Volume : 20.000 µl
Method          : C:\Users\Public\Documents\ChemStation\1\Data\SUN\SUN 2024-05-29 12-03-40\AS3-
                  05-30.M (Sequence Method)
Last changed    : 15/8/2022 10:23:13 pm by SYSTEM
Additional Info : Peak(s) manually integrated
```

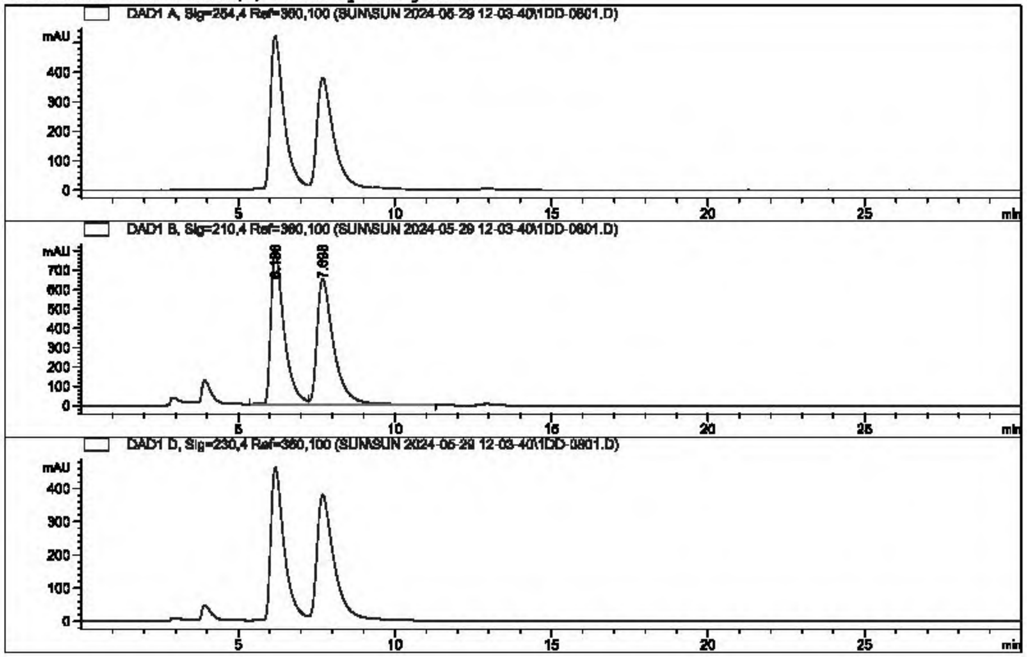


```
=====================================================================
                         Area Percent Report
=====================================================================

Sorted By             :      Signal
Multiplier            :      1.0000
Dilution              :      1.0000
Use Multiplier & Dilution Factor with ISTDs


Signal 1: DAD1 A, Sig=254,4 Ref=360,100
```


Data File C:\Users\P...Documents\ChemStation\1\Data\SUN\SUN 2024-05-29 12-03-40\1DD-0801.D
Sample Name: zzj-4-18-3-rac-as

Signal 2: DAD1 B, Sig=210,4 Ref=360,100

Peak #	RetTime [min]	Type	Width [min]	Area [mAU*s]	Height [mAU]	Area %
1	6.186	BV	0.4333	2.32795e4	787.59448	48.9717
2	7.698	VB	0.5409	2.42571e4	648.54022	51.0283
Totals :				4.75366e4	1436.13470	

Signal 3: DAD1 D, Sig=230,4 Ref=360,100

*** End of Report ***

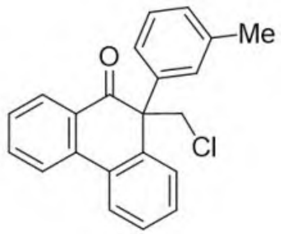

3m-rac

Data File C:\Users\P...Documents\ChemStation\1\Data\SUN\SUN 2024-05-29 20-28-44\1DA-0201.D
Sample Name: zzj-3-77

```
=====================================================================
Acq. Operator   : SYSTEM                        Seq. Line :   2
Sample Operator : SYSTEM
Acq. Instrument : HPLC                           Location :   P1-D-01
Injection Date  : 29/5/2024 8:41:56 pm                Inj :   1
                                               Inj Volume : 2.000 µl
Different Inj Volume from Sample Entry!  Actual Inj Volume : 20.000 µl
Method          : C:\Users\Public\Documents\ChemStation\1\Data\SUN\SUN 2024-05-29 20-28-44\AS3-
                  05-30.M (Sequence Method)
Last changed    : 15/8/2022 10:23:13 pm by SYSTEM
Additional Info : Peak(s) manually integrated
```

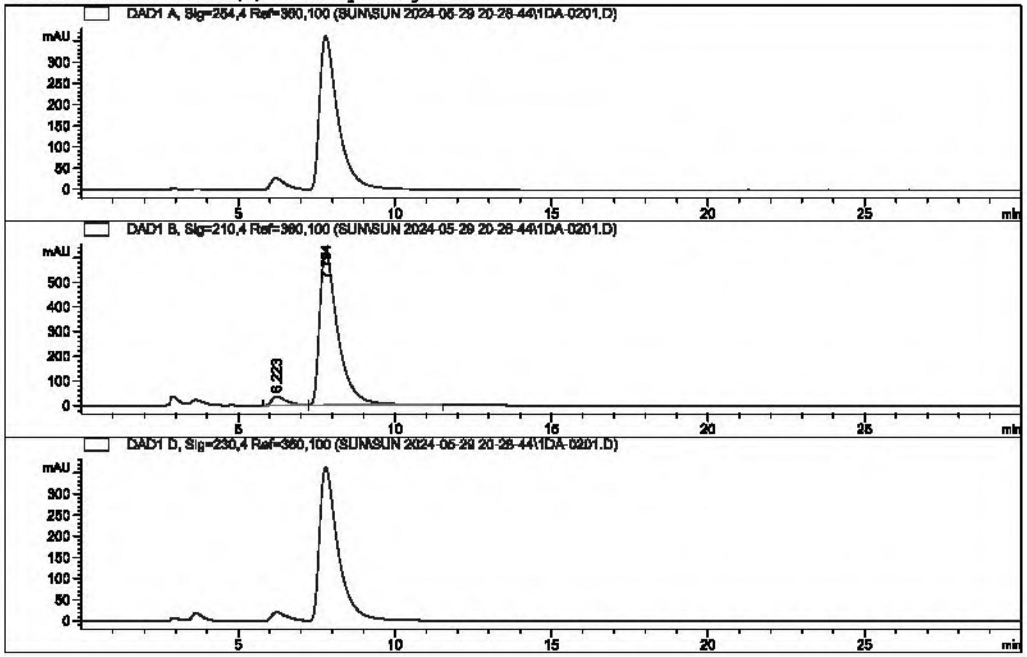

Area Percent Report

```
Sorted By             :      Signal
Multiplier            :      1.0000
Dilution              :      1.0000
Use Multiplier & Dilution Factor with ISTDs
```

Signal 1: DAD1 A, Sig=254,4 Ref=360,100

Data File C:\Users\P...Documents\ChemStation\1\Data\SUN\SUN 2024-05-29 20-28-44\1DA-0201.D
Sample Name: zzj-3-77

Signal 2: DAD1 B, Sig=210,4 Ref=360,100

Peak #	RetTime [min]	Type	Width [min]	Area [mAU*s]	Height [mAU]	Area %
1	6.223	BB	0.4235	1139.28845	35.07180	4.2923
2	7.794	BB	0.5919	2.54033e4	621.51575	95.7077
Totals :				2.65426e4	656.58755	

Signal 3: DAD1 D, Sig=230,4 Ref=360,100

*** End of Report ***

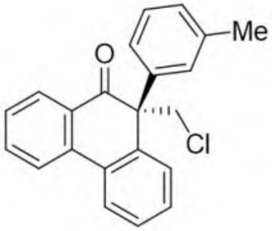

3m

Data File C:\Users\P...Documents\ChemStation\1\Data\SUN\SUN 2024-06-03 22-17-49\1DD-0401.D
Sample Name: zzj-4-21-10-rac

```
Acq. Operator   : SYSTEM                       Seq. Line :   4
Sample Operator : SYSTEM
Acq. Instrument : HPLC                          Location :   P1-D-04
Injection Date  : 3/6/2024 11:13:05 pm               Inj :   1
                                              Inj Volume : 2.000 µl
Different Inj Volume from Sample Entry!  Actual Inj Volume : 20.000 µl
Method          : C:\Users\Public\Documents\ChemStation\1\Data\SUN\SUN 2024-06-03 22-17-49\AS3-
                  15-20.M (Sequence Method)
Last changed    : 6/4/2023 6:42:58 pm by SYSTEM
Additional Info : Peak(s) manually integrated
```

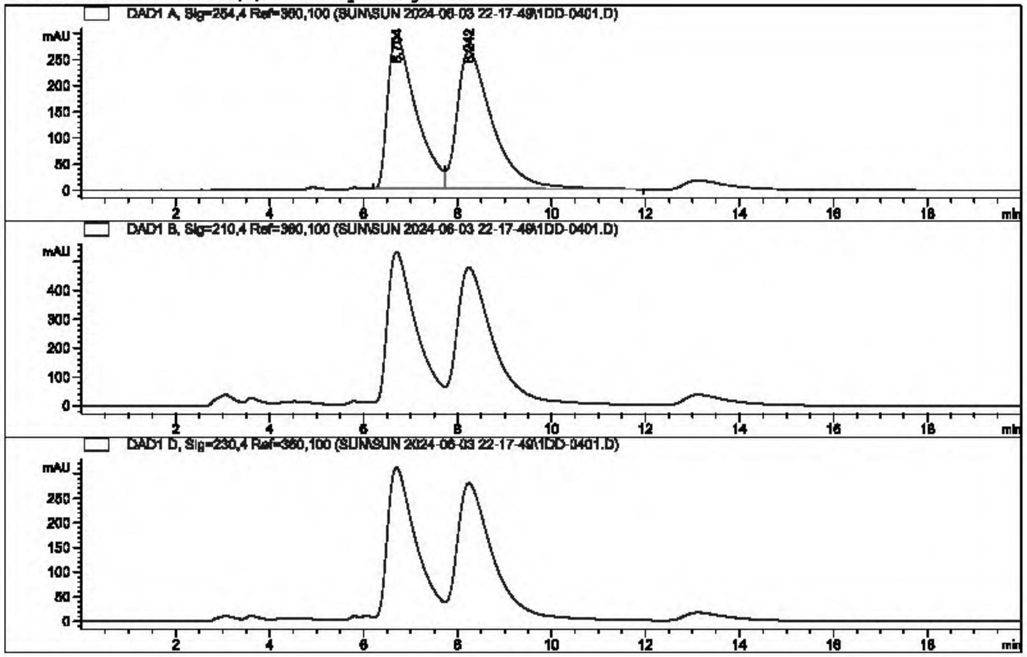

Area Percent Report

```
Sorted By          :      Signal
Multiplier         :      1.0000
Dilution           :      1.0000
Use Multiplier & Dilution Factor with ISTDs
```

Signal 1: DAD1 A, Sig=254,4 Ref=360,100

Peak #	RetTime [min]	Type	Width [min]	Area [mAU*s]	Height [mAU]	Area %
1	6.704	BV	0.5958	1.22094e4	291.08624	46.9103
2	8.242	VB	0.7311	1.38177e4	262.72116	53.0897

Data File C:\Users\P...Documents\ChemStation\1\Data\SUN\SUN 2024-06-03 22-17-49\1DD-0401.D
Sample Name: zzj-4-21-10-rac

Peak #	RetTime [min]	Type	Width [min]	Area [mAU*s]	Height [mAU]	Area %
Totals :				2.60270e4	553.80740	

Signal 2: DAD1 B, Sig=210,4 Ref=360,100

Signal 3: DAD1 D, Sig=230,4 Ref=360,100

*** End of Report ***

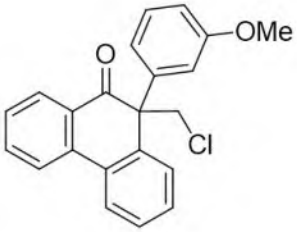

3n-rac

Data File C:\Users\P...\ChemStation\1\Data\SUN\SUN 2024-03-08 20-59-51\OnlineEdited--008.D
Sample Name: zzj-3-121

```
=====================================================================
Acq. Operator   : SYSTEM                        Seq. Line :    8
Sample Operator : SYSTEM
Acq. Instrument : HPLC                           Location :   P1-C-02
Injection Date  : 8/3/2024 11:39:35 pm                Inj :   1
                                               Inj Volume : 2.000 µl
Different Inj Volume from Sample Entry!  Actual Inj Volume : 25.000 µl
Method          : C:\Users\Public\Documents\ChemStation\1\Data\SUN\SUN 2024-03-08 20-59-51\AS3-
                  15-20.M (Sequence Method)
Last changed    : 6/4/2023 6:42:58 pm by SYSTEM
Additional Info : Peak(s) manually integrated
```

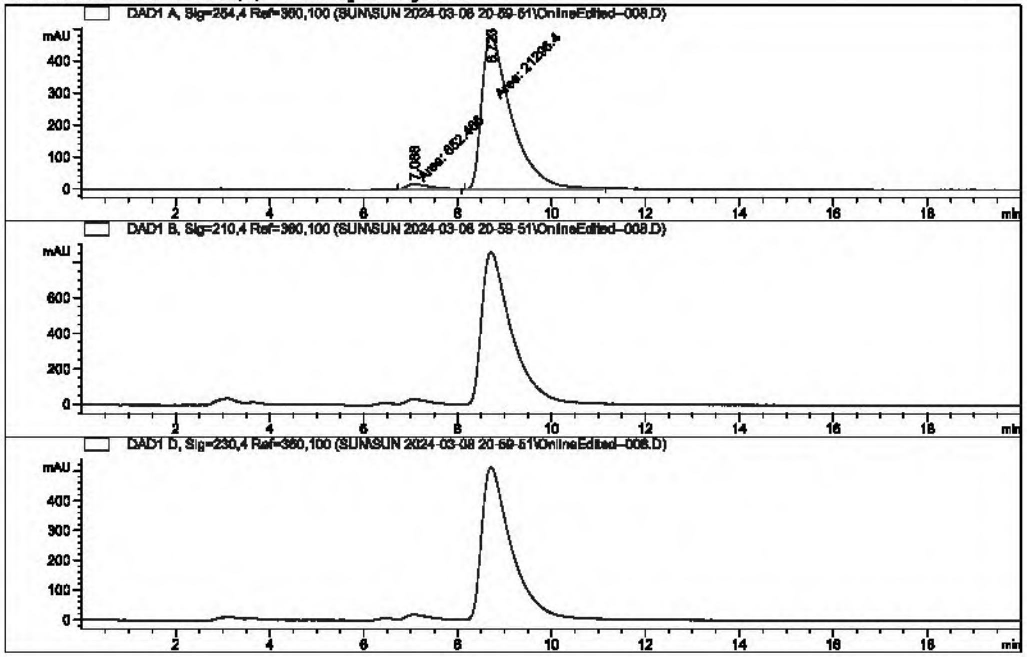

===

Area Percent Report

===

```
Sorted By             :      Signal
Multiplier            :      1.0000
Dilution              :      1.0000
Use Multiplier & Dilution Factor with ISTDs
```

Signal 1: DAD1 A, Sig=254,4 Ref=360,100

Peak #	RetTime [min]	Type	Width [min]	Area [mAU*s]	Height [mAU]	Area %
1	7.088	MM	0.6038	652.46503	18.01022	2.9727
2	8.723	MM	0.7400	2.12964e4	479.62106	97.0273

Data File C:\Users\P...\ChemStation\1\Data\SUN\SUN 2024-03-08 20-59-51\OnlineEdited--008.D
Sample Name: zzj-3-121

Peak #	RetTime [min]	Type	Width [min]	Area [mAU*s]	Height [mAU]	Area %
Totals :				2.19489e4	497.63128	

Signal 2: DAD1 B, Sig=210,4 Ref=360,100

Signal 3: DAD1 D, Sig=230,4 Ref=360,100

===

*** End of Report ***

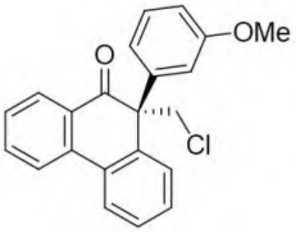

3n

Data File C:\Users\P...\ChemStation\1\Data\SUN\SUN 2024-05-21 15-25-06\OnlineEdited--033.D
Sample Name: zzj-4-18-1-ibn

```
=====================================================================
Acq. Operator   : SYSTEM                        Seq. Line :  33
Sample Operator : SYSTEM
Acq. Instrument : HPLC                           Location :  P1-A-03
Injection Date  : 22/5/2024 2:29:17 am                Inj :  1
                                               Inj Volume : 2.000 µl
Different Inj Volume from Sample Entry!  Actual Inj Volume : 25.000 µl
Acq. Method     : C:\Users\Public\Documents\ChemStation\1\Data\SUN\SUN 2024-05-21 15-25-06\IBN3-
                  05-30.M
Last changed    : 29/8/2022 6:09:41 pm by SYSTEM
Analysis Method : C:\Users\Public\Documents\ChemStation\1\Data\SUN\SUN 2024-05-21 15-25-06\IBN3-
                  05-30.M (Sequence Method)
Last changed    : 13/12/2025 2:42:11 pm by SYSTEM
                  (modified after loading)
Additional Info : Peak(s) manually integrated
```

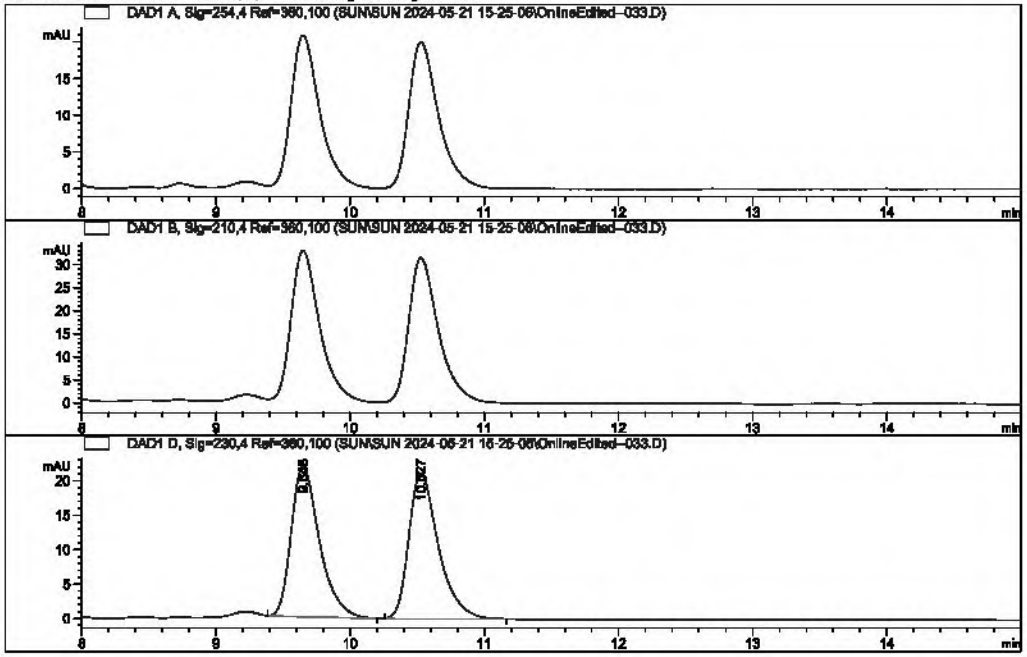


```
=====================================================================
                      Area Percent Report
=====================================================================

Sorted By             :      Signal
Multiplier            :      1.0000
Dilution              :      1.0000
Use Multiplier & Dilution Factor with ISTDs
```


Data File C:\Users\P...\ChemStation\1\Data\SUN\SUN 2024-05-21 15-25-06\OnlineEdited--033.D
Sample Name: zzj-4-18-1-ibn

Signal 1: DAD1 A, Sig=254,4 Ref=360,100

Signal 2: DAD1 B, Sig=210,4 Ref=360,100

Signal 3: DAD1 D, Sig=230,4 Ref=360,100

Peak #	RetTime [min]	Type	Width [min]	Area [mAU*s]	Height [mAU]	Area %
1	9.648	BB	0.2195	319.81589	21.82490	49.7410
2	10.527	BB	0.2269	323.14670	21.13237	50.2590

Totals : 642.96259 42.95727

*** End of Report ***

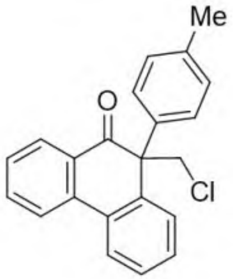

3o-rac

Data File C:\Users\P...\ChemStation\1\Data\SUN\SUN 2024-05-22 17-10-33\OnlineEdited--023.D
Sample Name: zzj-3-59

===

```
Acq. Operator   : SYSTEM                        Seq. Line :  23
Sample Operator : SYSTEM
Acq. Instrument : HPLC                           Location :   P1-B-03
Injection Date  : 23/5/2024 12:02:09 am               Inj :   1
                                               Inj Volume : 2.000 µl
Different Inj Volume from Sample Entry!  Actual Inj Volume : 20.000 µl
Acq. Method     : C:\Users\Public\Documents\ChemStation\1\Data\SUN\SUN 2024-05-22 17-10-33\IBN3-
                  05-30.M
Last changed    : 29/8/2022 6:09:41 pm by SYSTEM
Analysis Method : C:\Users\Public\Documents\ChemStation\1\Data\SUN\SUN 2024-05-22 17-10-33\IBN3-
                  05-30.M (Sequence Method)
Last changed    : 13/12/2025 2:43:16 pm by SYSTEM
                  (modified after loading)
Additional Info : Peak(s) manually integrated
```

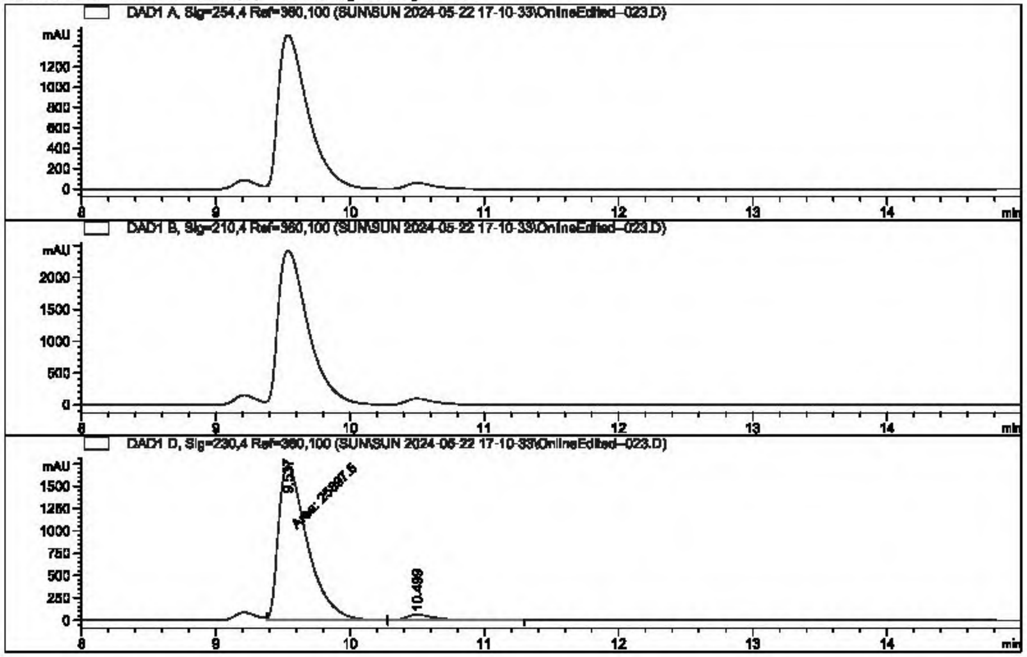

===

Area Percent Report

===

```
Sorted By             :      Signal
Multiplier            :      1.0000
Dilution              :      1.0000
Use Multiplier & Dilution Factor with ISTDs
```


Data File C:\Users\P...\ChemStation\1\Data\SUN\SUN 2024-05-22 17-10-33\OnlineEdited--023.D
Sample Name: zzj-3-59

Signal 1: DAD1 A, Sig=254,4 Ref=360,100

Signal 2: DAD1 B, Sig=210,4 Ref=360,100

Signal 3: DAD1 D, Sig=230,4 Ref=360,100

Peak #	RetTime [min]	Type	Width [min]	Area [mAU*s]	Height [mAU]	Area %
1	9.537	FM	0.2509	2.58975e4	1720.15637	96.4993
2	10.499	VB	0.2203	939.49542	63.97374	3.5007

Totals : 2.68370e4 1784.13012

*** End of Report ***

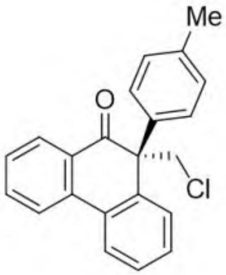

3o

Data File C:\Users\P...Documents\ChemStation\1\Data\SUN\SUN 2024-05-25 19-38-12\1DA-0201.D
Sample Name: zzj-4-18-1-ibn

Acq. Operator : SYSTEM   Seq. Line : 2
Sample Operator : SYSTEM
Acq. Instrument : HPLC   Location : P1-D-01
Injection Date : 25/5/2024 7:51:15 pm   Inj : 1
Inj Volume : 2.000 µl
Different Inj Volume from Sample Entry! Actual Inj Volume : 25.000 µl
Acq. Method : C:\Users\Public\Documents\ChemStation\1\Data\SUN\SUN 2024-05-25 19-38-12\IBN3-05-30.M
Last changed : 29/8/2022 6:09:41 pm by SYSTEM
Analysis Method : C:\Users\Public\Documents\ChemStation\1\Data\SUN\SUN 2024-05-25 19-38-12\IBN3-05-30.M (Sequence Method)
Last changed : 13/12/2025 2:45:53 pm by SYSTEM
(modified after loading)
Additional Info : Peak(s) manually integrated

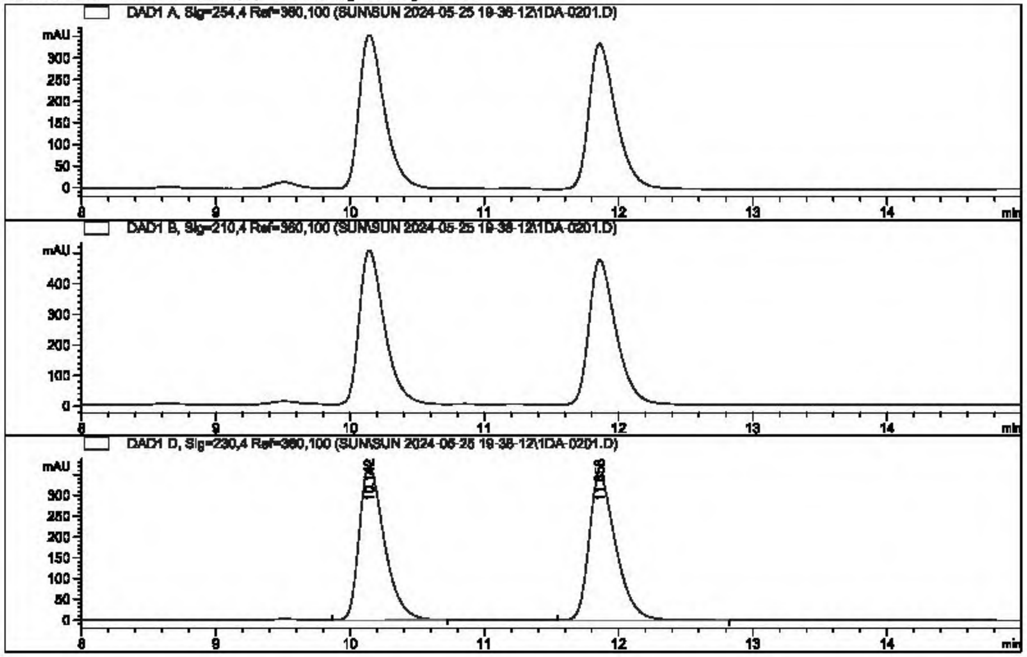

Area Percent Report

Sorted By : Signal
Multiplier : 1.0000
Dilution : 1.0000
Use Multiplier & Dilution Factor with ISTDs

Data File C:\Users\P...Documents\ChemStation\1\Data\SUN\SUN 2024-05-25 19-38-12\1DA-0201.D
Sample Name: zzj-4-18-1-ibn

Signal 1: DAD1 A, Sig=254,4 Ref=360,100

Signal 2: DAD1 B, Sig=210,4 Ref=360,100

Signal 3: DAD1 D, Sig=230,4 Ref=360,100

Peak #	RetTime [min]	Type	Width [min]	Area [mAU*s]	Height [mAU]	Area %
1	10.142	BB	0.2002	4836.35498	370.61929	49.8042
2	11.858	BB	0.2108	4874.38574	349.38324	50.1958

Totals : 9710.74072 720.00253

*** End of Report ***

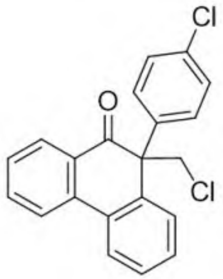

3p-rac

```
=====================================================================
Acq. Operator   : SYSTEM                        Seq. Line :   9
Sample Operator : SYSTEM
Acq. Instrument : HPLC                           Location :   P1-D-01
Injection Date  : 28/5/2024 10:22:24 pm               Inj :   1
                                               Inj Volume : 2.000 µl
Different Inj Volume from Sample Entry!  Actual Inj Volume : 20.000 µl
Acq. Method     : C:\Users\Public\Documents\ChemStation\1\Data\SUN\SUN 2024-05-28 20-11-26\IBN3-
                  05-30.M
Last changed    : 29/8/2022 6:09:41 pm by SYSTEM
Analysis Method : C:\Users\Public\Documents\ChemStation\1\Data\SUN\SUN 2024-05-28 20-11-26\IBN3-
                  05-30.M (Sequence Method)
Last changed    : 13/12/2025 2:49:16 pm by SYSTEM
                  (modified after loading)
Additional Info : Peak(s) manually integrated
```

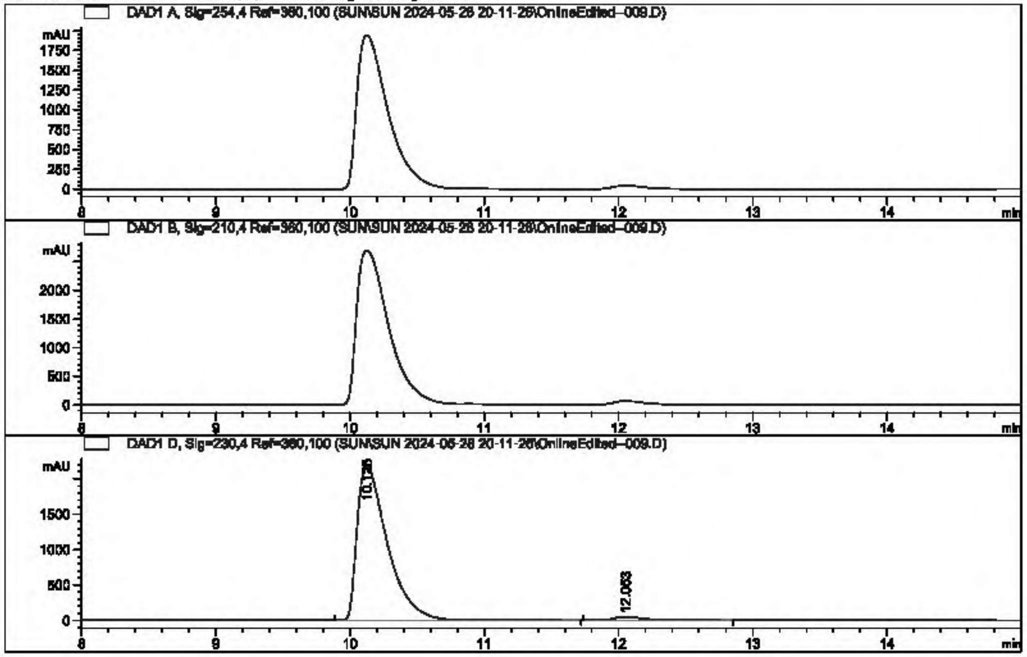


```
=====================================================================
                         Area Percent Report
=====================================================================

Sorted By             :      Signal
Multiplier            :      1.0000
Dilution              :      1.0000
Use Multiplier & Dilution Factor with ISTDs
```


Data File C:\Users\P...\ChemStation\1\Data\SUN\SUN 2024-05-28 20-11-26\OnlineEdited--009.D
Sample Name: zzj-3-56

Signal 1: DAD1 A, Sig=254,4 Ref=360,100

Signal 2: DAD1 B, Sig=210,4 Ref=360,100

Signal 3: DAD1 D, Sig=230,4 Ref=360,100

Peak #	RetTime [min]	Type	Width [min]	Area [mAU*s]	Height [mAU]	Area %
1	10.125	BV R	0.2359	3.42059e4	2174.45801	97.7913
2	12.053	BB	0.2329	772.56665	49.55155	2.2087
Totals :				3.49784e4	2224.00956	

*** End of Report ***

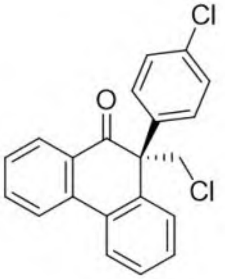

3p

Data File C:\Users\P...Documents\ChemStation\1\Data\SUN\SUN 2024-05-29 12-03-40\1DC-0401.D
Sample Name: zzj-4-18-5-rac-ibn

Acq. Operator : SYSTEM Seq. Line : 4
Sample Operator : SYSTEM
Acq. Instrument : HPLC Location : P1-D-03
Injection Date : 29/5/2024 12:54:02 pm Inj : 1
Inj Volume : 2.000 µl
Different Inj Volume from Sample Entry! Actual Inj Volume : 20.000 µl
Method : C:\Users\Public\Documents\ChemStation\1\Data\SUN\SUN 2024-05-29 12-03-40\IBN3-05-30.M (Sequence Method)
Last changed : 29/8/2022 6:09:41 pm by SYSTEM
Additional Info : Peak(s) manually integrated

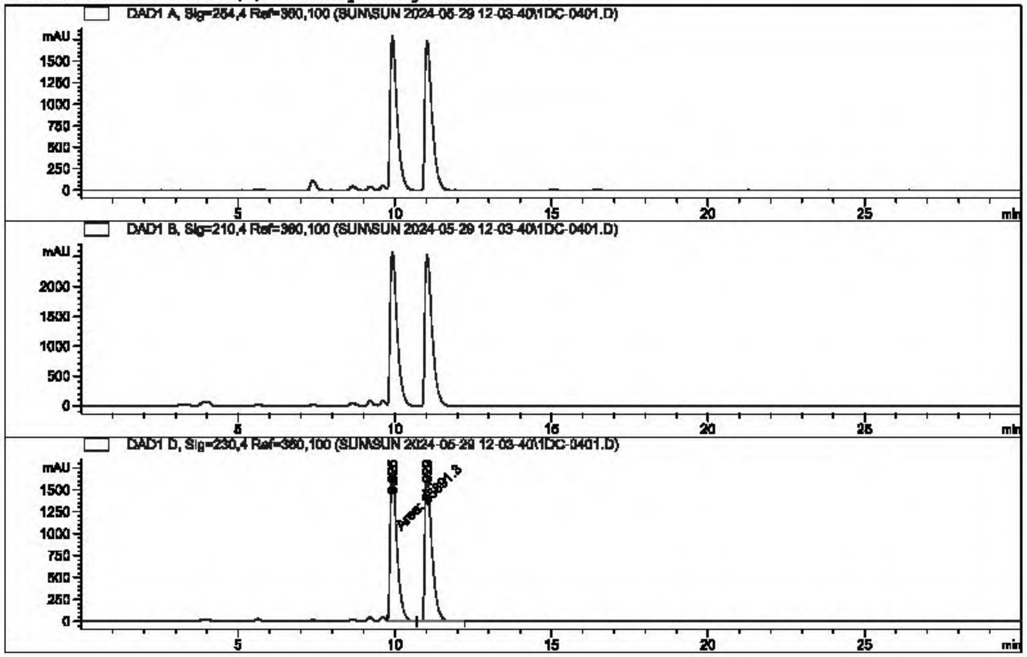

Area Percent Report

Sorted By : Signal
Multiplier : 1.0000
Dilution : 1.0000
Use Multiplier & Dilution Factor with ISTDs

Signal 1: DAD1 A, Sig=254,4 Ref=360,100

Data File C:\Users\P...Documents\ChemStation\1\Data\SUN\SUN 2024-05-29 12-03-40\1DC-0401.D
Sample Name: zzj-4-18-5-rac-ibn

Signal 2: DAD1 B, Sig=210,4 Ref=360,100

Signal 3: DAD1 D, Sig=230,4 Ref=360,100

Peak #	RetTime [min]	Type	Width [min]	Area [mAU*s]	Height [mAU]	Area %
1	9.925	FM	0.2458	2.58913e4	1755.46362	49.9236
2	11.029	BB	0.2284	2.59705e4	1703.37695	50.0764

Totals : 5.18618e4 3458.84058

*** End of Report ***

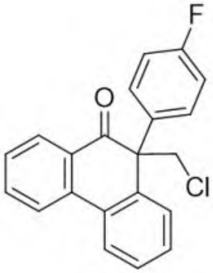

3q-rac

Data File C:\Users\P...Documents\ChemStation\1\Data\SUN\SUN 2024-05-29 20-28-44\1DB-0401.D
Sample Name: zzj-3-86

```
=====================================================================
Acq. Operator   : SYSTEM                        Seq. Line :   4
Sample Operator : SYSTEM
Acq. Instrument : HPLC                           Location :  P1-D-02
Injection Date  : 29/5/2024 9:24:07 pm                Inj :  1
                                               Inj Volume : 2.000 µl
Different Inj Volume from Sample Entry!  Actual Inj Volume : 20.000 µl
Method          : C:\Users\Public\Documents\ChemStation\1\Data\SUN\SUN 2024-05-29 20-28-44\IBN3-
                  05-30.M (Sequence Method)
Last changed    : 29/8/2022 6:09:41 pm by SYSTEM
Additional Info : Peak(s) manually integrated
```

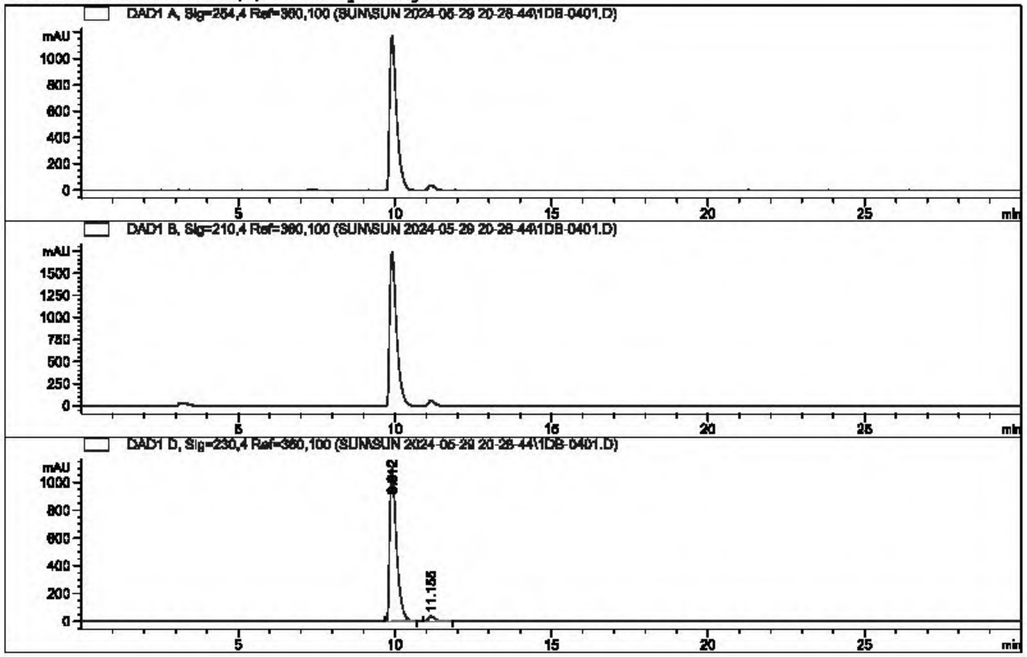

Area Percent Report

```
Sorted By             :      Signal
Multiplier            :      1.0000
Dilution              :      1.0000
Use Multiplier & Dilution Factor with ISTDs
```

Signal 1: DAD1 A, Sig=254,4 Ref=360,100

Data File C:\Users\P...Documents\ChemStation\1\Data\SUN\SUN 2024-05-29 20-28-44\1DB-0401.D
Sample Name: zzj-3-86

Signal 2: DAD1 B, Sig=210,4 Ref=360,100

Signal 3: DAD1 D, Sig=230,4 Ref=360,100

Peak #	RetTime [min]	Type	Width [min]	Area [mAU*s]	Height [mAU]	Area %
1	9.912	BB	0.2294	1.67203e4	1108.83618	97.0116
2	11.155	BB	0.2131	515.06891	35.53341	2.9884

Totals : 1.72354e4 1144.36959

*** End of Report ***

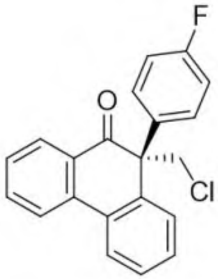

3q

```
=====================================================================
Acq. Operator   : SYSTEM                        Seq. Line :  11
Sample Operator : SYSTEM
Acq. Instrument : HPLC                           Location :   P1-D-02
Injection Date  : 28/5/2024 11:04:48 pm               Inj :   1
                                               Inj Volume : 2.000 µl
Different Inj Volume from Sample Entry!  Actual Inj Volume : 20.000 µl
Acq. Method     : C:\Users\Public\Documents\ChemStation\1\Data\SUN\SUN 2024-05-28 20-11-26\AD3-
                  05-50.M
Last changed    : 5/7/2023 9:48:06 pm by SYSTEM
Analysis Method : C:\Users\Public\Documents\ChemStation\1\Data\SUN\SUN 2024-05-28 20-11-26\AD3-
                  05-50.M (Sequence Method)
Last changed    : 13/12/2025 2:54:14 pm by SYSTEM
                  (modified after loading)
Additional Info : Peak(s) manually integrated
```

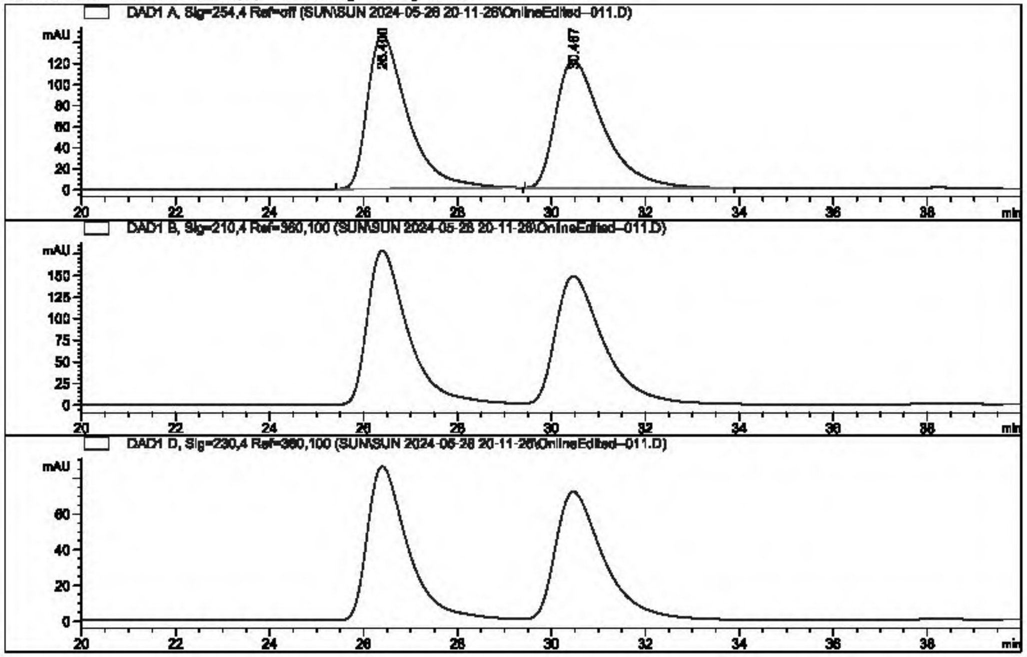


```
=====================================================================
                         Area Percent Report
=====================================================================

Sorted By             :      Signal
Multiplier            :      1.0000
Dilution              :      1.0000
Use Multiplier & Dilution Factor with ISTDs
```


Data File C:\Users\P...\ChemStation\1\Data\SUN\SUN 2024-05-28 20-11-26\OnlineEdited--011.D
Sample Name: zzj-4-18-6-rac

Signal 1: DAD1 A, Sig=254,4 Ref=off

Peak #	RetTime [min]	Type	Width [min]	Area [mAU*s]	Height [mAU]	Area %
1	26.400	BB	0.8124	8669.06836	145.96225	50.4738
2	30.467	BB	0.9355	8506.30078	121.09641	49.5262

Totals : 1.71754e4 267.05865

Signal 2: DAD1 B, Sig=210,4 Ref=360,100

Signal 3: DAD1 D, Sig=230,4 Ref=360,100

*** End of Report ***

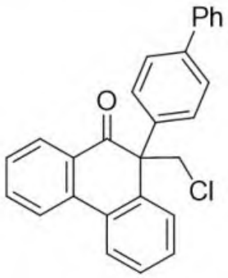

3r-rac

```
Acq. Operator   : SYSTEM                        Seq. Line :  36
Sample Operator : SYSTEM
Acq. Instrument : HPLC                           Location :   P1-F-05
Injection Date  : 5/3/2024 5:21:34 am                 Inj :   1
                                               Inj Volume : 2.000 µl
Different Inj Volume from Sample Entry!  Actual Inj Volume : 25.000 µl
Acq. Method     : C:\Users\Public\Documents\ChemStation\1\Data\SUN\SUN 2024-03-04 18-29-50\AD3-
                  05-50.M
Last changed    : 5/7/2023 9:48:06 pm by SYSTEM
Analysis Method : C:\Users\Public\Documents\ChemStation\1\Data\SUN\SUN 2024-03-04 18-29-50\AD3-
                  05-50.M (Sequence Method)
Last changed    : 13/12/2025 2:55:19 pm by SYSTEM
                  (modified after loading)
Additional Info : Peak(s) manually integrated
```

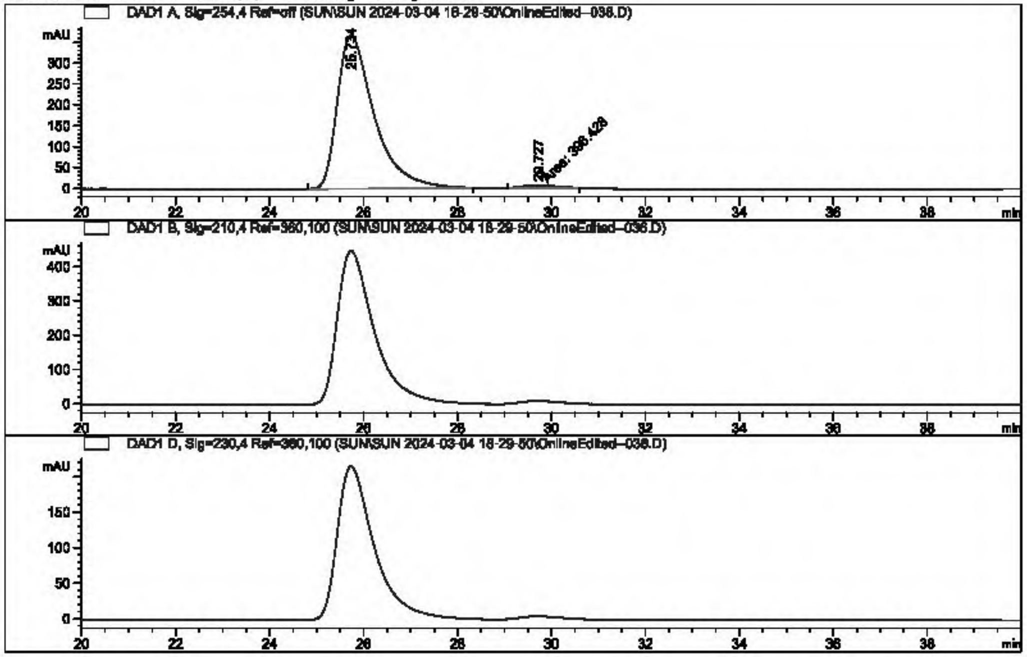

Area Percent Report

```
Sorted By         :     Signal
Multiplier        :     1.0000
Dilution          :     1.0000
Use Multiplier & Dilution Factor with ISTDs
```


Data File C:\Users\P...\ChemStation\1\Data\SUN\SUN 2024-03-04 18-29-50\OnlineEdited--036.D
Sample Name: zzj-3-115

Signal 1: DAD1 A, Sig=254,4 Ref=off

Peak #	RetTime [min]	Type	Width [min]	Area [mAU*s]	Height [mAU]	Area %
1	25.734	BV R	0.6397	2.00161e4	367.92184	98.0579
2	29.727	MM	0.8894	396.42831	7.42878	1.9421

Totals : 2.04125e4 375.35062

Signal 2: DAD1 B, Sig=210,4 Ref=360,100

Signal 3: DAD1 D, Sig=230,4 Ref=360,100

*** End of Report ***

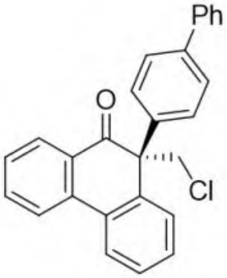

3r

===

Acq. Operator : SYSTEM Seq. Line : 2
Sample Operator : SYSTEM
Acq. Instrument : HPLC Location : P1-D-03
Injection Date : 3/6/2024 10:30:53 pm Inj : 1
Inj Volume : 2.000 µl
Different Inj Volume from Sample Entry! Actual Inj Volume : 20.000 µl
Acq. Method : C:\Users\Public\Documents\ChemStation\1\Data\SUN\SUN 2024-06-03 22-17-49\AD3-10-30.M
Last changed : 24/10/2022 9:57:21 am by SYSTEM
Analysis Method : C:\Users\Public\Documents\ChemStation\1\Data\SUN\SUN 2024-06-03 22-17-49\AD3-10-30.M (Sequence Method)
Last changed : 13/12/2025 2:57:36 pm by SYSTEM
(modified after loading)
Additional Info : Peak(s) manually integrated

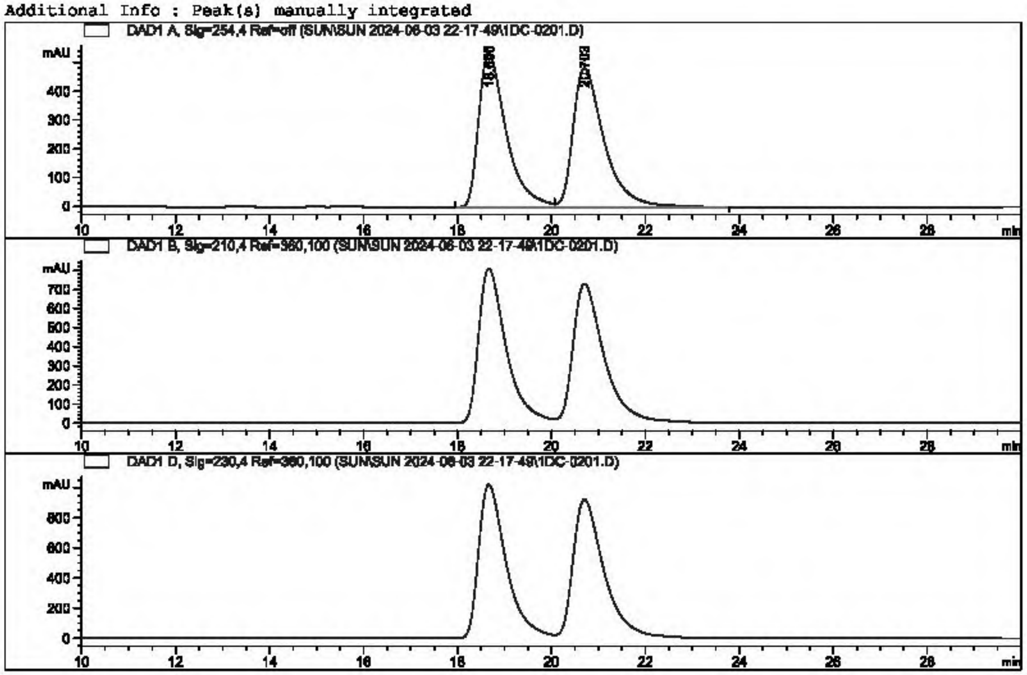

===

Area Percent Report

===

Sorted By : Signal
Multiplier : 1.0000
Dilution : 1.0000
Use Multiplier & Dilution Factor with ISTDs

Data File C:\Users\P...Documents\ChemStation\1\Data\SUN\SUN 2024-06-03 22-17-49\1DC-0201.D
Sample Name: zzj-4-21-9-rac

Signal 1: DAD1 A, Sig=254,4 Ref=off

Peak #	RetTime [min]	Type	Width [min]	Area [mAU*s]	Height [mAU]	Area %
1	18.666	BV	0.6107	2.18349e4	536.11926	49.5704
2	20.703	VB	0.6685	2.22133e4	483.50229	50.4296
Totals :				4.40482e4	1019.62155	

Signal 2: DAD1 B, Sig=210,4 Ref=360,100

Signal 3: DAD1 D, Sig=230,4 Ref=360,100

*** End of Report ***

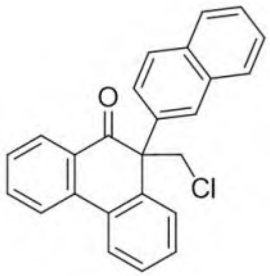

3s-rac

Data File C:\Users\P...\ChemStation\1\Data\SUN\SUN 2024-01-23 21-16-42\OnlineEdited--017.D
Sample Name: zzj-3-80

Acq. Operator	: SYSTEM	Seq. Line :	17
Sample Operator	: SYSTEM		
Acq. Instrument	: HPLC	Location :	P1-A-01
Injection Date	: 24/1/2024 3:36:17 am	Inj :	1
		Inj Volume :	2.000 µl

Different Inj Volume from Sample Entry! Actual Inj Volume : 25.000 µl
Acq. Method : C:\Users\Public\Documents\ChemStation\1\Data\SUN\SUN 2024-01-23 21-16-42\AD3-10-30.M
Last changed : 24/10/2022 9:57:21 am by SYSTEM
Analysis Method : C:\Users\Public\Documents\ChemStation\1\Data\SUN\SUN 2024-01-23 21-16-42\AD3-10-30.M (Sequence Method)
Last changed : 13/12/2025 2:59:02 pm by SYSTEM
(modified after loading)
Additional Info : Peak(s) manually integrated

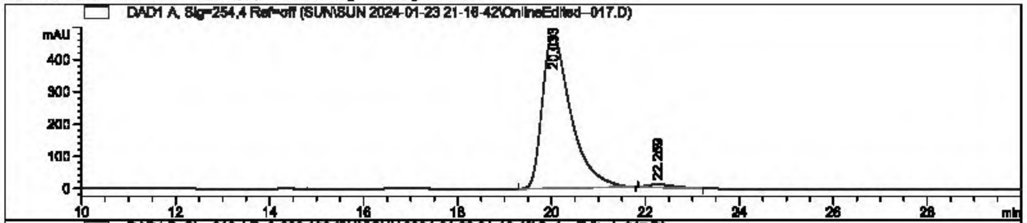

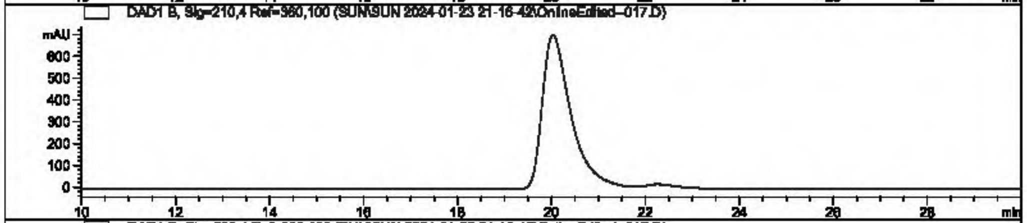

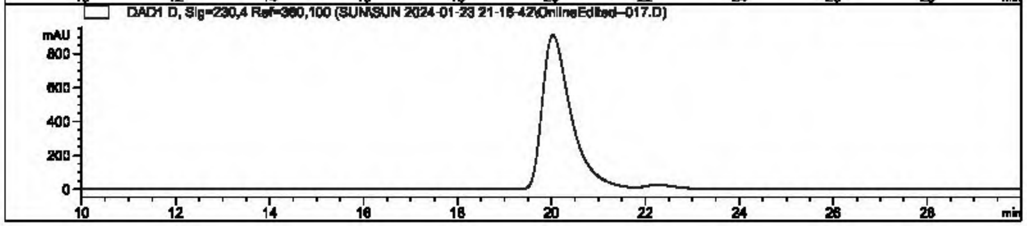

Area Percent Report

Sorted By : Signal
Multiplier : 1.0000
Dilution : 1.0000
Use Multiplier & Dilution Factor with ISTDs

Data File C:\Users\P...\ChemStation\1\Data\SUN\SUN 2024-01-23 21-16-42\OnlineEdited--017.D
Sample Name: zzj-3-80

Signal 1: DAD1 A, Sig=254,4 Ref=off

Peak #	RetTime [min]	Type	Width [min]	Area [mAU*s]	Height [mAU]	Area %
1	20.033	BB	0.5284	2.00332e4	475.60245	98.3063
2	22.259	BB	0.4265	345.15326	9.50857	1.6937
Totals :				2.03784e4	485.11101	

Signal 2: DAD1 B, Sig=210,4 Ref=360,100

Signal 3: DAD1 D, Sig=230,4 Ref=360,100

*** End of Report ***

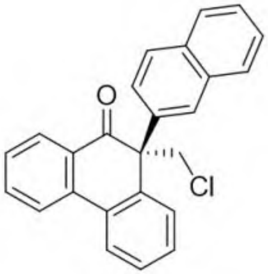

3s

Data File C:\Users\P...Documents\ChemStation\1\Data\SUN\SUN 2024-07-10 23-10-40\1AC-0401.D
Sample Name: zzj-4-38-21

```
Acq. Operator   : SYSTEM                    Seq. Line :   4
Sample Operator : SYSTEM
Acq. Instrument : HPLC                       Location :   P1-A-03
Injection Date  : 10/7/2024 11:55:45 pm           Inj :   1
                                          Inj Volume : 2.000 µl
Different Inj Volume from Sample Entry!  Actual Inj Volume : 1.000 µl
Acq. Method     : C:\Users\Public\Documents\ChemStation\1\Data\SUN\SUN 2024-07-10 23-10-40\OD3-
                  10-30.M
Last changed    : 27/10/2022 8:12:28 pm by SYSTEM
Analysis Method : C:\Users\Public\Documents\ChemStation\1\Data\SUN\SUN 2024-07-10 23-10-40\OD3-
                  10-30.M (Sequence Method)
Last changed    : 13/12/2025 3:03:24 pm by SYSTEM
                  (modified after loading)
Additional Info : Peak(s) manually integrated
```

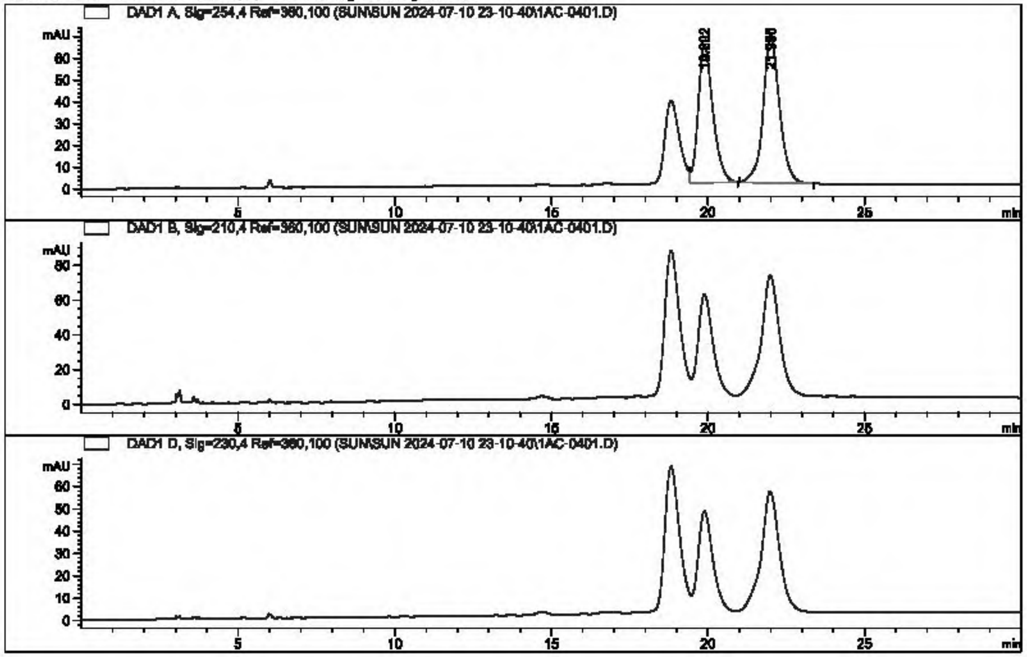

Area Percent Report

```
Sorted By          :      Signal
Multiplier         :      1.0000
Dilution           :      1.0000
Use Multiplier & Dilution Factor with ISTDs
```


Data File C:\Users\P...Documents\ChemStation\1\Data\SUN\SUN 2024-07-10 23-10-40\1AC-0401.D
Sample Name: zzj-4-38-21

Signal 1: DAD1 A, Sig=254,4 Ref=360,100

Peak #	RetTime [min]	Type	Width [min]	Area [mAU*s]	Height [mAU]	Area %
1	19.892	VB	0.4850	2074.84473	63.37363	45.7352
2	21.996	BB	0.5053	2461.80200	67.77515	54.2648
Totals :				4536.64673	131.14878	

Signal 2: DAD1 B, Sig=210,4 Ref=360,100

Signal 3: DAD1 D, Sig=230,4 Ref=360,100

*** End of Report ***

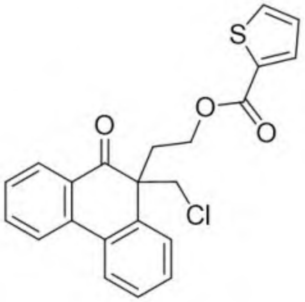

3t-rac

Data File C:\Users\P...Documents\ChemStation\1\Data\SUN\SUN 2024-05-11 09-32-52\1AB-0201.D
Sample Name: zzj-4-8

Acq. Operator : SYSTEM Seq. Line : 2
Sample Operator : SYSTEM
Acq. Instrument : HPLC Location : P1-A-02
Injection Date : 11/5/2024 9:46:02 am Inj : 1
Inj Volume : 2.000 µl
Different Inj Volume from Sample Entry! Actual Inj Volume : 25.000 µl
Method : C:\Users\Public\Documents\ChemStation\1\Data\SUN\SUN 2024-05-11 09-32-52\OD3-10-30.M (Sequence Method)
Last changed : 27/10/2022 8:12:28 pm by SYSTEM
Additional Info : Peak(s) manually integrated

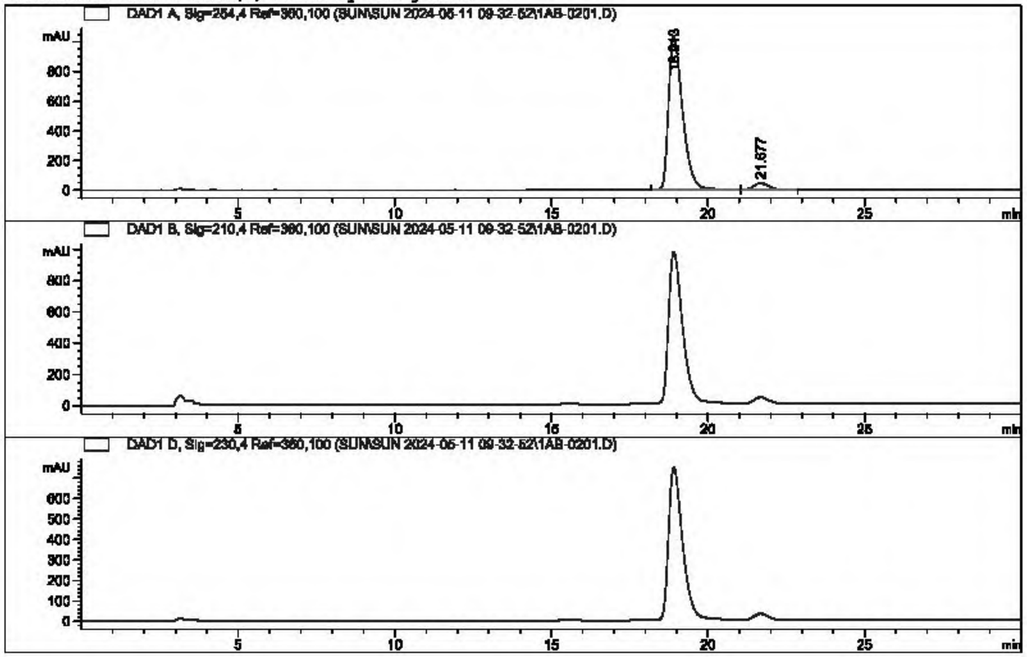

Area Percent Report

Sorted By : Signal
Multiplier : 1.0000
Dilution : 1.0000
Use Multiplier & Dilution Factor with ISTDs

Signal 1: DAD1 A, Sig=254,4 Ref=360,100

Peak #	RetTime [min]	Type	Width [min]	Area [mAU*s]	Height [mAU]	Area %
1	18.913	BB	0.4694	3.19861e4	1031.20764	95.6737
2	21.677	BB	0.4595	1446.38623	44.99364	4.3263

Data File C:\Users\P...Documents\ChemStation\1\Data\SUN\SUN 2024-05-11 09-32-52\1AB-0201.D
Sample Name: zzj-4-8

Peak #	RetTime [min]	Type	Width [min]	Area [mAU*s]	Height [mAU]	Area %
Totals :				3.34325e4	1076.20128	

Signal 2: DAD1 B, Sig=210,4 Ref=360,100

Signal 3: DAD1 D, Sig=230,4 Ref=360,100

*** End of Report ***

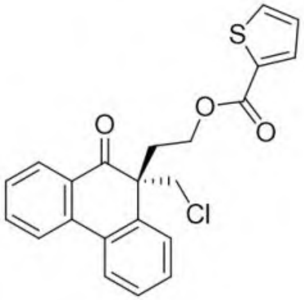

3t

Acq. Operator : SYSTEM Seq. Line : 6
Sample Operator : SYSTEM
Acq. Instrument : HPLC Location : P1-D-01
Injection Date : 10/6/2024 6:26:57 pm Inj : 1
Inj Volume : 2.000 µl
Different Inj Volume from Sample Entry! Actual Inj Volume : 5.000 µl
Method : C:\Users\Public\Documents\ChemStation\1\Data\SUN\SUN 2024-06-10 17-07-17\OD3-05-20.M (Sequence Method)
Last changed : 17/11/2023 2:04:40 pm by SYSTEM
Additional Info : Peak(s) manually integrated

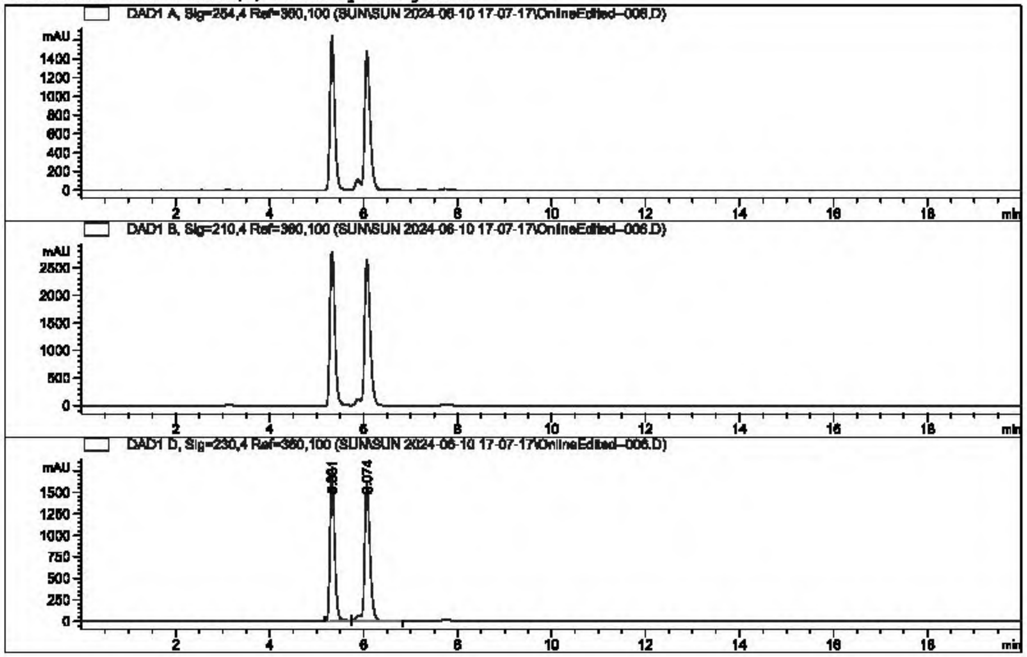

Area Percent Report

Sorted By : Signal
Multiplier : 1.0000
Dilution : 1.0000
Use Multiplier & Dilution Factor with ISTDs

Signal 1: DAD1 A, Sig=254,4 Ref=360,100

Data File C:\Users\P...\ChemStation\1\Data\SUN\SUN 2024-06-10 17-07-17\OnlineEdited--006.D
Sample Name: zzj-4-6-4

Signal 2: DAD1 B, Sig=210,4 Ref=360,100

Signal 3: DAD1 D, Sig=230,4 Ref=360,100

Peak #	RetTime [min]	Type	Width [min]	Area [mAU*s]	Height [mAU]	Area %
1	5.331	BV R	0.1027	1.20391e4	1792.10388	48.5192
2	6.074	VV R	0.1175	1.27739e4	1602.84961	51.4808

Totals : 2.48130e4 3394.95349

*** End of Report ***

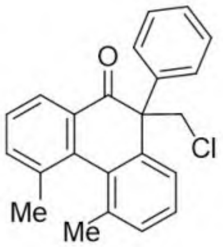

3u-rac

```
=====================================================================
Acq. Operator   : SYSTEM                        Seq. Line :  26
Sample Operator : SYSTEM
Acq. Instrument : HPLC                           Location :   P1-A-05
Injection Date  : 25/6/2024 12:35:17 am               Inj :   1
                                            Inj Volume : 2.000 µl
Different Inj Volume from Sample Entry!  Actual Inj Volume : 20.000 µl
Acq. Method     : C:\Users\Public\Documents\ChemStation\1\Data\SUN\SUN 2024-06-24 15-21-13\OD3-5
                  -30.M
Last changed    : 22/8/2022 12:41:47 pm by SYSTEM
Analysis Method : C:\Users\Public\Documents\ChemStation\1\Data\SUN\SUN 2024-06-24 15-21-13\OD3-5
                  -30.M (Sequence Method)
Last changed    : 13/12/2025 3:09:44 pm by SYSTEM
                  (modified after loading)
Additional Info : Peak(s) manually integrated
```

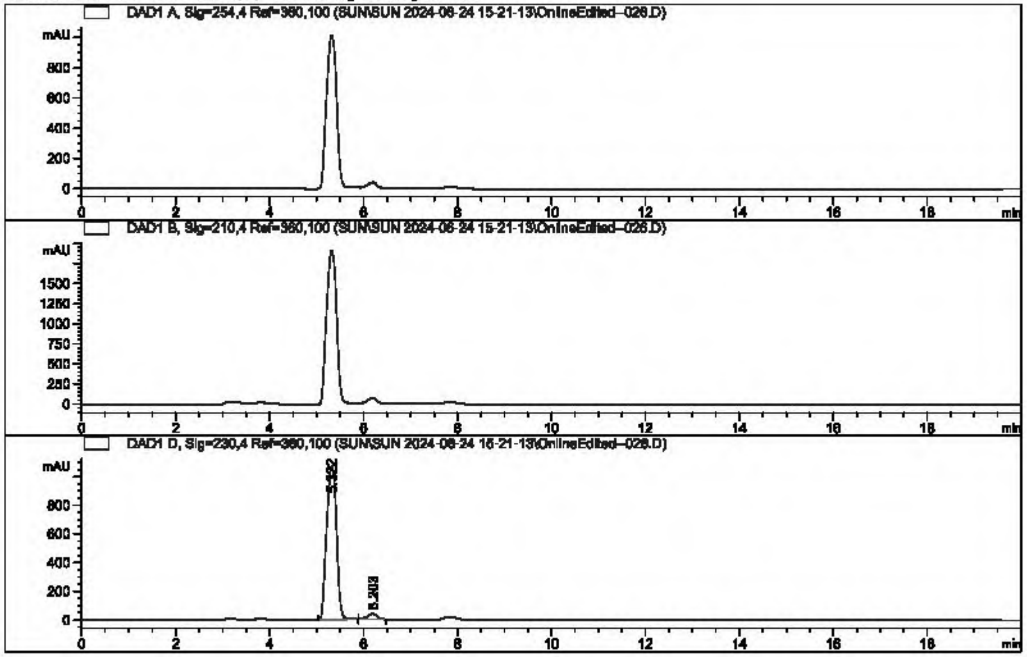

Area Percent Report

```
Sorted By             :      Signal
Multiplier            :      1.0000
Dilution              :      1.0000
Use Multiplier & Dilution Factor with ISTDs
```


Data File C:\Users\P...\ChemStation\1\Data\SUN\SUN 2024-06-24 15-21-13\OnlineEdited--026.D
Sample Name: zzj-4-36-od

Signal 1: DAD1 A, Sig=254,4 Ref=360,100

Signal 2: DAD1 B, Sig=210,4 Ref=360,100

Signal 3: DAD1 D, Sig=230,4 Ref=360,100

Peak #	RetTime [min]	Type	Width [min]	Area [mAU*s]	Height [mAU]	Area %
1	5.322	BB	0.2490	1.56903e4	1070.49988	96.8224
2	6.203	BB	0.2125	514.94037	37.91261	3.1776
Totals :				1.62052e4	1108.41248	

*** End of Report ***

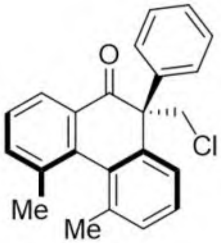

3u

Data File C:\Users\P...emStation\1\Data\SUN\SUN 2024-12-23 10-16-13\015-P1-D3-zzj-5-32-5.D
Sample Name: zzj-5-32-5

```
Acq. Operator   : SYSTEM                      Seq. Line :  15
Sample Operator : SYSTEM
Acq. Instrument : HPLC                         Location :  P1-D-03
Injection Date  : 23/12/2024 2:34:52 pm             Inj :  1
                                             Inj Volume : 2.000 µl
Method          : C:\Users\Public\Documents\ChemStation\1\Data\SUN\SUN 2024-12-23 10-16-13\OD3-
                  05-20.M (Sequence Method)
Last changed    : 17/11/2023 2:04:40 pm by SYSTEM
Additional Info : Peak(s) manually integrated
```

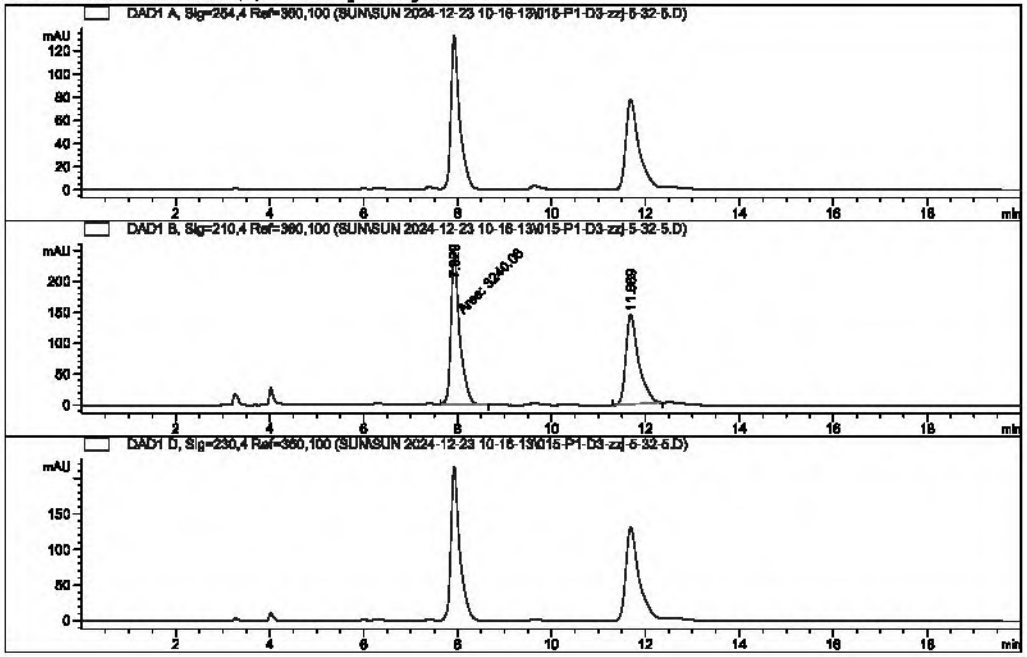

Area Percent Report

```
Sorted By              :      Signal
Multiplier             :      1.0000
Dilution               :      1.0000
Use Multiplier & Dilution Factor with ISTDs
```

Signal 1: DAD1 A, Sig=254,4 Ref=360,100

Data File C:\Users\P...emStation\1\Data\SUN\SUN 2024-12-23 10-16-13\015-P1-D3-zzj-5-32-5.D
Sample Name: zzj-5-32-5

Signal 2: DAD1 B, Sig=210,4 Ref=360,100

Peak #	RetTime [min]	Type	Width [min]	Area [mAU*s]	Height [mAU]	Area %
1	7.929	FM	0.2175	3240.08154	248.28004	54.1946
2	11.689	BB	0.2724	2738.52173	144.98320	45.8054
Totals :				5978.60327	393.26324	

Signal 3: DAD1 D, Sig=230,4 Ref=360,100

*** End of Report ***

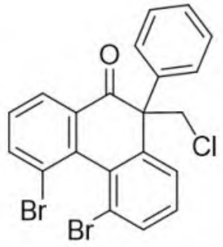

3v-rac

Acq. Operator	: SYSTEM	Seq. Line :	10
Sample Operator	: SYSTEM		
Acq. Instrument	: HPLC	Location :	P1-A-07
Injection Date	: 27/8/2024 2:03:32 am	Inj :	1
		Inj Volume :	2.000 µl

Different Inj Volume from Sample Entry! Actual Inj Volume : 20.000 µl

Method : C:\Users\Public\Documents\ChemStation\1\Data\SUN\SUN 2024-08-26 22-32-41\OD3-05-20.M (Sequence Method)

Last changed : 17/11/2023 2:04:40 pm by SYSTEM

Additional Info : Peak(s) manually integrated

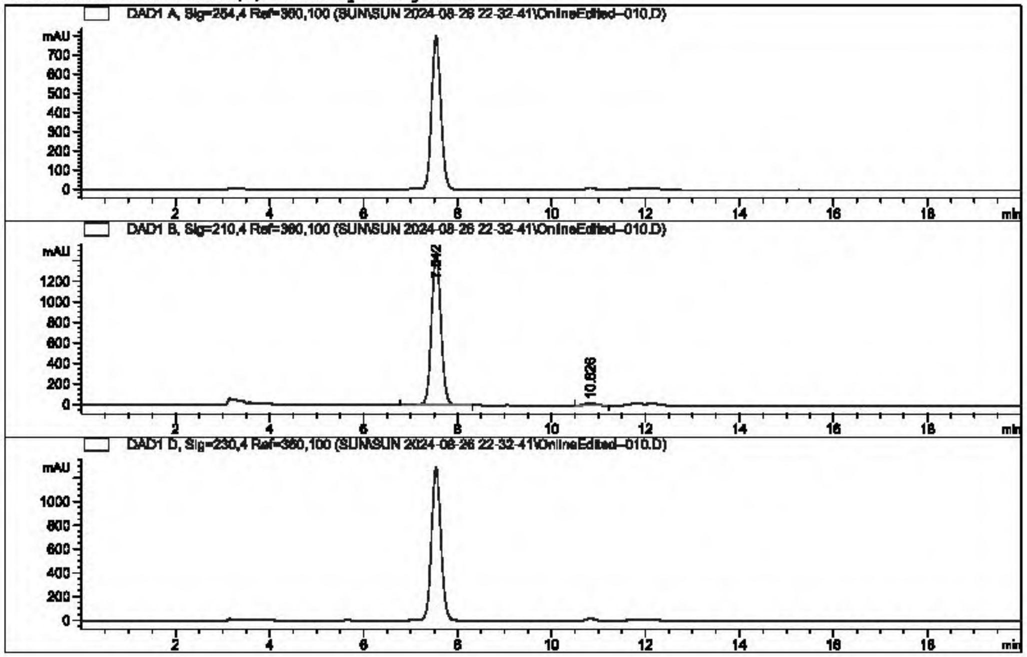

Area Percent Report

Sorted By	:	Signal
Multiplier	:	1.0000
Dilution	:	1.0000

Use Multiplier & Dilution Factor with ISTDs

Signal 1: DAD1 A, Sig=254,4 Ref=360,100

Data File C:\Users\P...\ChemStation\1\Data\SUN\SUN 2024-08-26 22-32-41\OnlineEdited--010.D
Sample Name: zzj-4-98-scale up

Signal 2: DAD1 B, Sig=210,4 Ref=360,100

Peak #	RetTime [min]	Type	Width [min]	Area [mAU*s]	Height [mAU]	Area %
1	7.542	VB R	0.2144	2.07258e4	1489.48206	98.4006
2	10.826	BB	0.2101	336.87357	23.04143	1.5994

Totals : 2.10627e4 1512.52349

Signal 3: DAD1 D, Sig=230,4 Ref=360,100

*** End of Report ***

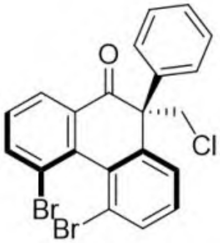

3v

```
=====================================================================
Acq. Operator   : SYSTEM                        Seq. Line :  14
Sample Operator : SYSTEM
Acq. Instrument : HPLC                           Location :   P1-D-09
Injection Date  : 3/7/2024 6:47:09 pm                 Inj :   1
                                             Inj Volume : 2.000 µl
Different Inj Volume from Sample Entry!  Actual Inj Volume : 10.000 µl
Acq. Method     : C:\Users\Public\Documents\ChemStation\1\Data\SUN\SUN 2024-07-03 14-56-28\AD3-
                  03-20.M
Last changed    : 16/6/2023 5:27:25 pm by SYSTEM
Analysis Method : C:\Users\Public\Documents\ChemStation\1\Data\SUN\SUN 2024-07-03 14-56-28\AD3-
                  03-20.M (Sequence Method)
Last changed    : 13/12/2025 3:40:34 pm by SYSTEM
                  (modified after loading)
Additional Info : Peak(s) manually integrated
```

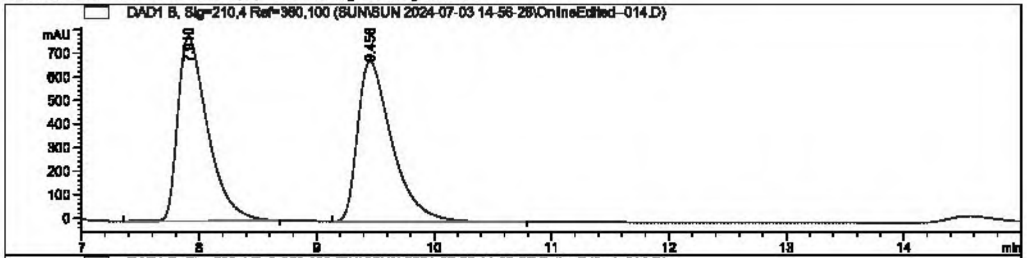

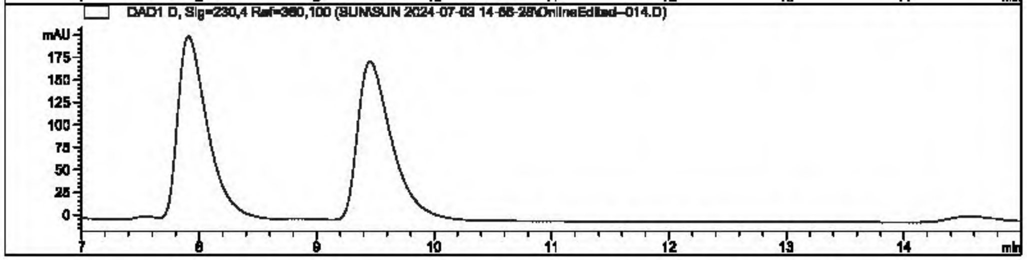


```
=====================================================================
                         Area Percent Report
=====================================================================

Sorted By             :      Signal
Multiplier            :      1.0000
Dilution              :      1.0000
Use Multiplier & Dilution Factor with ISTDs
```

Signal 1: DAD1 B, Sig=210,4 Ref=360,100

Peak #	RetTime [min]	Type	Width [min]	Area [mAU*s]	Height [mAU]	Area %
1	7.910	VB R	0.2657	1.38116e4	782.64911	50.0116
2	9.456	BB	0.3051	1.38052e4	679.15662	49.9884

Totals : 2.76168e4 1461.80573

Data File C:\Users\P...\ChemStation\1\Data\SUN\SUN 2024-07-03 14-56-28\OnlineEdited--014.D
Sample Name: zzj-4-38-17-rac

Signal 2: DAD1 D, Sig=230,4 Ref=360,100

```
=====================================================================
                         *** End of Report ***
```

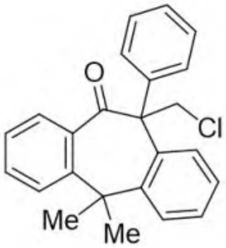

3w-rac

Data File C:\Users\P...\ChemStation\1\Data\SUN\SUN 2024-04-17 10-37-28\OnlineEdited--055.D
Sample Name: zzj-3-171

```
=====================================================================
Acq. Operator   : SYSTEM                        Seq. Line :  55
Sample Operator : SYSTEM
Acq. Instrument : HPLC                           Location :   P1-C-01
Injection Date  : 18/4/2024 1:14:55 am                Inj :   1
                                               Inj Volume : 2.000 µl
Different Inj Volume from Sample Entry!  Actual Inj Volume : 25.000 µl
Acq. Method     : C:\Users\Public\Documents\ChemStation\1\Data\SUN\SUN 2024-04-17 10-37-28\AD3-
                  03-20.M
Last changed    : 16/6/2023 5:27:25 pm by SYSTEM
Analysis Method : C:\Users\Public\Documents\ChemStation\1\Data\SUN\SUN 2024-04-17 10-37-28\AD3-
                  03-20.M (Sequence Method)
Last changed    : 13/12/2025 3:41:56 pm by SYSTEM
                  (modified after loading)
Additional Info : Peak(s) manually integrated
```

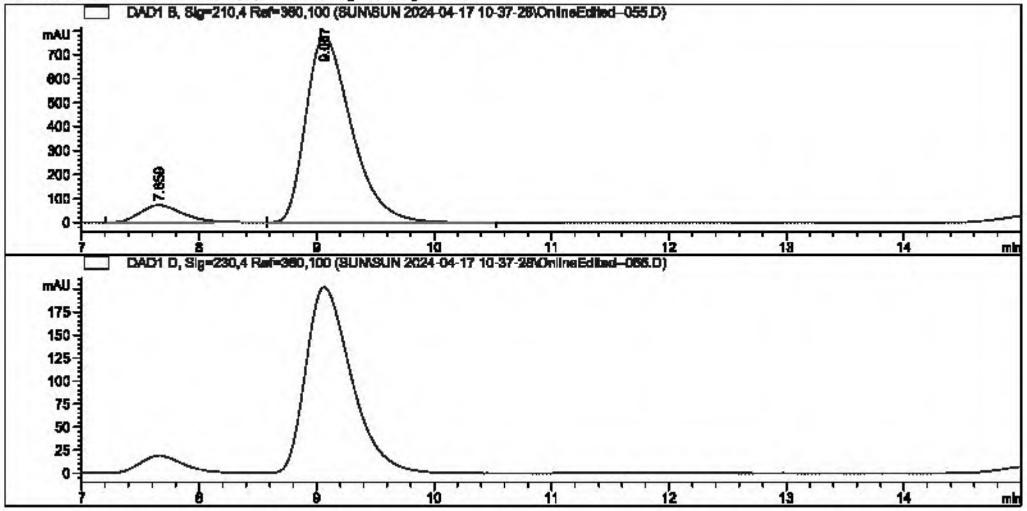

Area Percent Report

```
Sorted By             :      Signal
Multiplier            :      1.0000
Dilution              :      1.0000
Use Multiplier & Dilution Factor with ISTDs
```

Signal 1: DAD1 B, Sig=210,4 Ref=360,100

Peak #	RetTime [min]	Type	Width [min]	Area [mAU*s]	Height [mAU]	Area %
1	7.659	BB	0.3723	1788.47998	71.49641	7.9051
2	9.067	BB	0.4152	2.08358e4	774.95978	92.0949

Totals : 2.26242e4 846.45619

Data File C:\Users\P...\ChemStation\1\Data\SUN\SUN 2024-04-17 10-37-28\OnlineEdited--055.D
Sample Name: zzj-3-171

Signal 2: DAD1 D, Sig=230,4 Ref=360,100

*** End of Report ***

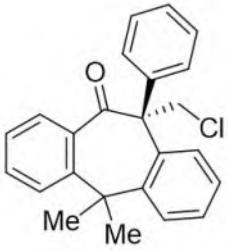

3w

Data File C:\Users\P...\ChemStation\1\Data\SUN\SUN 2024-03-23 14-10-29\OnlineEdited--013.D
Sample Name: zzj-3-143-1

===

Acq. Operator : SYSTEM   Seq. Line : 13
Sample Operator : SYSTEM
Acq. Instrument : HPLC   Location : P1-C-01
Injection Date : 23/3/2024 6:32:08 pm   Inj : 1
Inj Volume : 2.000 µl
Different Inj Volume from Sample Entry! Actual Inj Volume : 25.000 µl
Method : C:\Users\Public\Documents\ChemStation\1\Data\SUN\SUN 2024-03-23 14-10-29\IE3-05-20.M (Sequence Method)
Last changed : 18/8/2022 4:00:29 pm by SYSTEM
Additional Info : Peak(s) manually integrated

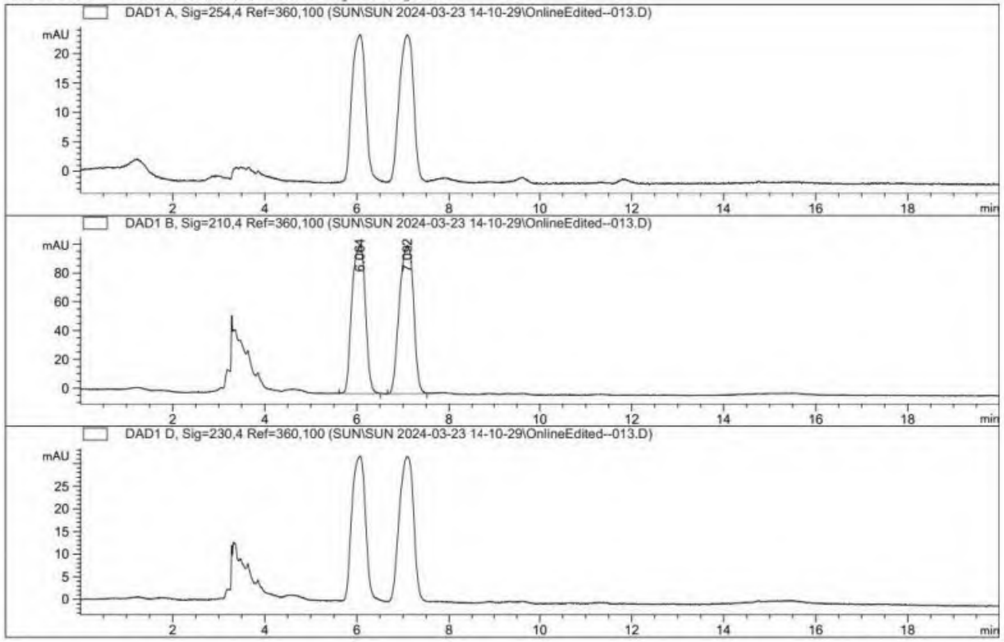

===

Area Percent Report

===

Sorted By : Signal
Multiplier : 1.0000
Dilution : 1.0000
Use Multiplier & Dilution Factor with ISTDs

Signal 1: DAD1 A, Sig=254,4 Ref=360,100

Data File C:\Users\P...\ChemStation\1\Data\SUN\SUN 2024-03-23 14-10-29\OnlineEdited--013.D
Sample Name: zzj-3-143-1

Signal 2: DAD1 B, Sig=210,4 Ref=360,100

Peak #	RetTime [min]	Type	Width [min]	Area [mAU*s]	Height [mAU]	Area %
1	6.064	VV R	0.2401	2083.89917	102.73712	48.5722
2	7.092	VV R	0.2529	2206.41699	102.99458	51.4278
Totals :				4290.31616	205.73170	

Signal 3: DAD1 D, Sig=230,4 Ref=360,100

===

*** End of Report ***

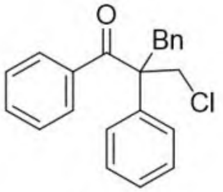

3x-rac

Data File C:\Users\P...\ChemStation\1\Data\SUN\SUN 2024-04-10 21-31-11\OnlineEdited--027.D
Sample Name: zzj-3-162-2

===

```
Acq. Operator   : SYSTEM                        Seq. Line :  27
Sample Operator : SYSTEM
Acq. Instrument : HPLC                           Location :  P1-C-03
Injection Date  : 11/4/2024 3:53:05 am                Inj :  1
                                               Inj Volume : 2.000 µl
Different Inj Volume from Sample Entry!  Actual Inj Volume : 25.000 µl
Acq. Method     : C:\Users\Public\Documents\ChemStation\1\Data\SUN\SUN 2024-04-10 21-31-11\IE3-
                  05-20.M
Last changed    : 18/8/2022 4:00:29 pm by SYSTEM
Analysis Method : C:\Users\Public\Documents\ChemStation\1\Data\SUN\SUN 2024-04-10 21-31-11\IE3-
                  05-20.M (Sequence Method)
Last changed    : 20/6/2026 4:50:27 pm by SYSTEM
                  (modified after loading)
Additional Info : Peak(s) manually integrated
```

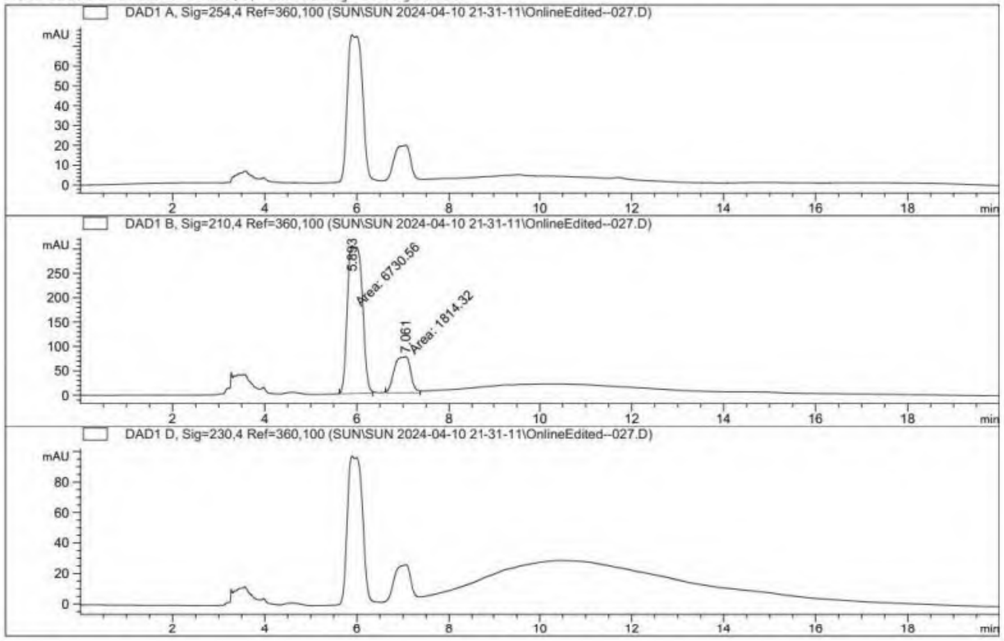

===
Area Percent Report
===

```
Sorted By          :      Signal
Multiplier         :      1.0000
Dilution           :      1.0000
Use Multiplier & Dilution Factor with ISTDs
```


Data File C:\Users\P...\ChemStation\1\Data\SUN\SUN 2024-04-10 21-31-11\OnlineEdited--027.D
Sample Name: zzj-3-162-2

Signal 1: DAD1 A, Sig=254,4 Ref=360,100

Signal 2: DAD1 B, Sig=210,4 Ref=360,100

Peak #	RetTime [min]	Type	Width [min]	Area [mAU*s]	Height [mAU]	Area %
1	5.893	MM	0.3697	6730.56152	303.40286	78.7672
2	7.061	MM	0.4090	1814.31555	73.92905	21.2328
Totals :				8544.87708	377.33192	

Signal 3: DAD1 D, Sig=230,4 Ref=360,100

===
*** End of Report ***

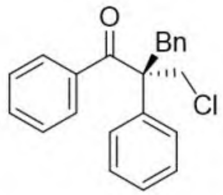

3x

Data File C:\Users\P...mStation\1\Data\SUN\SUN 2026-06-20 22-34-19\003-P2-C1-zzj-Br-rac1.D
Sample Name: zzj-Br-rac1

===

```
Acq. Operator   : SYSTEM                        Seq. Line :   3
Sample Operator : SYSTEM
Acq. Instrument : HPLC                           Location :  P2-C-01
Injection Date  : 20/6/2026 11:10:02 pm               Inj :   1
                                               Inj Volume : 2.000 µl
Different Inj Volume from Sample Entry!  Actual Inj Volume : 10.000 µl
Acq. Method     : C:\Users\Public\Documents\ChemStation\1\Data\SUN\SUN 2026-06-20 22-34-19\AS3-
                  03-20.M
Last changed    : 24/11/2023 8:39:22 pm by SYSTEM
Analysis Method : C:\Users\Public\Documents\ChemStation\1\Data\SUN\SUN 2026-06-20 22-34-19\AS3-
                  03-20.M (Sequence Method)
Last changed    : 20/6/2026 11:35:15 pm by SYSTEM
                  (modified after loading)
Additional Info : Peak(s) manually integrated
```

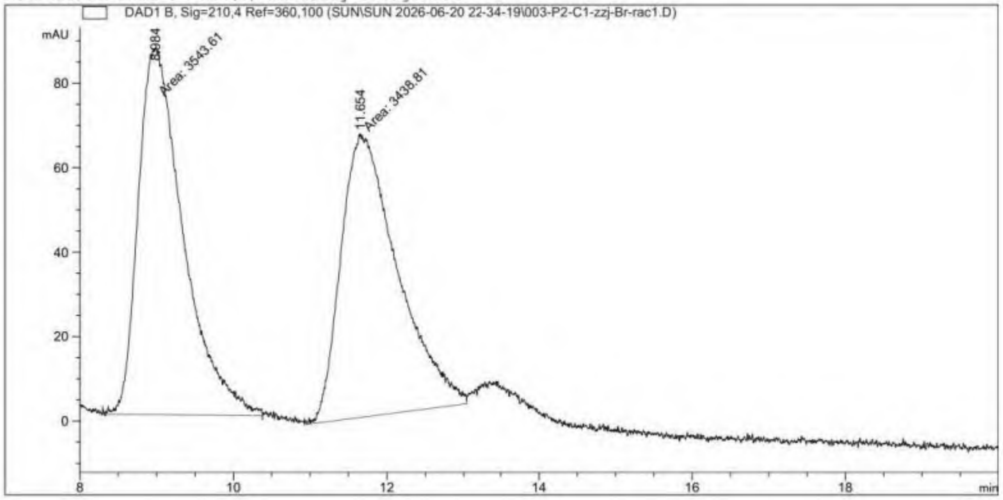

===
Area Percent Report
===

```
Sorted By             :      Signal
Multiplier            :      1.0000
Dilution              :      1.0000
Use Multiplier & Dilution Factor with ISTDs
```

Signal 1: DAD1 B, Sig=210,4 Ref=360,100

Peak #	RetTime [min]	Type	Width [min]	Area [mAU*s]	Height [mAU]	Area %
1	8.984	MM	0.6794	3543.61035	86.92609	50.7504
2	11.654	MM	0.8530	3438.81421	67.19090	49.2496

Totals : 6982.42456 154.11700

Data File C:\Users\P...mStation\1\Data\SUN\SUN 2026-06-20 22-34-19\003-P2-C1-zzj-Br-rac1.D
Sample Name: zzj-Br-rac1

===
*** End of Report ***

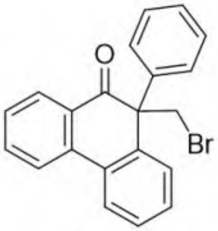

3y-rac

===

Acq. Operator : SYSTEM   Seq. Line : 2
Sample Operator : SYSTEM
Acq. Instrument : HPLC   Location : P2-C-05
Injection Date : 12/6/2026 10:28:59 am   Inj : 1
Inj Volume : 2.000 µl
Different Inj Volume from Sample Entry! Actual Inj Volume : 20.000 µl
Acq. Method : C:\Users\Public\Documents\ChemStation\1\Data\SUN\SUN 2026-06-12 10-14-39\AS3-03-30.M
Last changed : 2/3/2023 2:41:33 pm by SYSTEM
Analysis Method : C:\Users\Public\Documents\ChemStation\1\Data\SUN\SUN 2026-06-12 10-14-39\AS3-03-30.M (Sequence Method)
Last changed : 20/6/2026 11:37:51 pm by SYSTEM
(modified after loading)
Additional Info : Peak(s) manually integrated

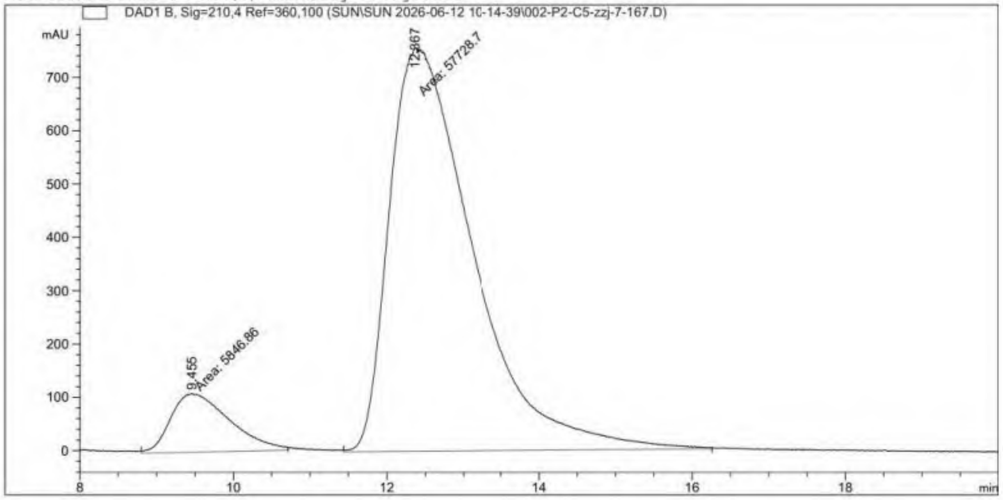

===
Area Percent Report
===

Sorted By : Signal
Multiplier : 1.0000
Dilution : 1.0000
Use Multiplier & Dilution Factor with ISTDs

Signal 1: DAD1 B, Sig=210,4 Ref=360,100

Peak #	RetTime [min]	Type	Width [min]	Area [mAU*s]	Height [mAU]	Area %
1	9.455	MM	0.8832	5846.85645	110.32996	9.1967
2	12.367	MM	1.2718	5.77287e4	756.49414	90.8033

Totals : 6.35755e4 866.82410

Data File C:\Users\P...hemStation\1\Data\SUN\SUN 2026-06-12 10-14-39\002-P2-C5-zzj-7-167.D
Sample Name: zzj-7-167

===
*** End of Report ***

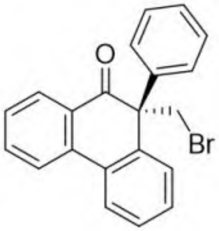

3y

===

Acq. Operator : SYSTEM Seq. Line : 21
Sample Operator : SYSTEM
Acq. Instrument : HPLC Location : P1-A-06
Injection Date : 19/12/2024 4:09:38 am Inj : 1
Inj Volume : 2.000 µl
Method : C:\Users\Public\Documents\ChemStation\1\Data\SUN\SUN 2024-12-18 21-45-11\ID3-03-30.M (Sequence Method)
Last changed : 8/4/2024 9:59:11 am by SYSTEM
Additional Info : Peak(s) manually integrated

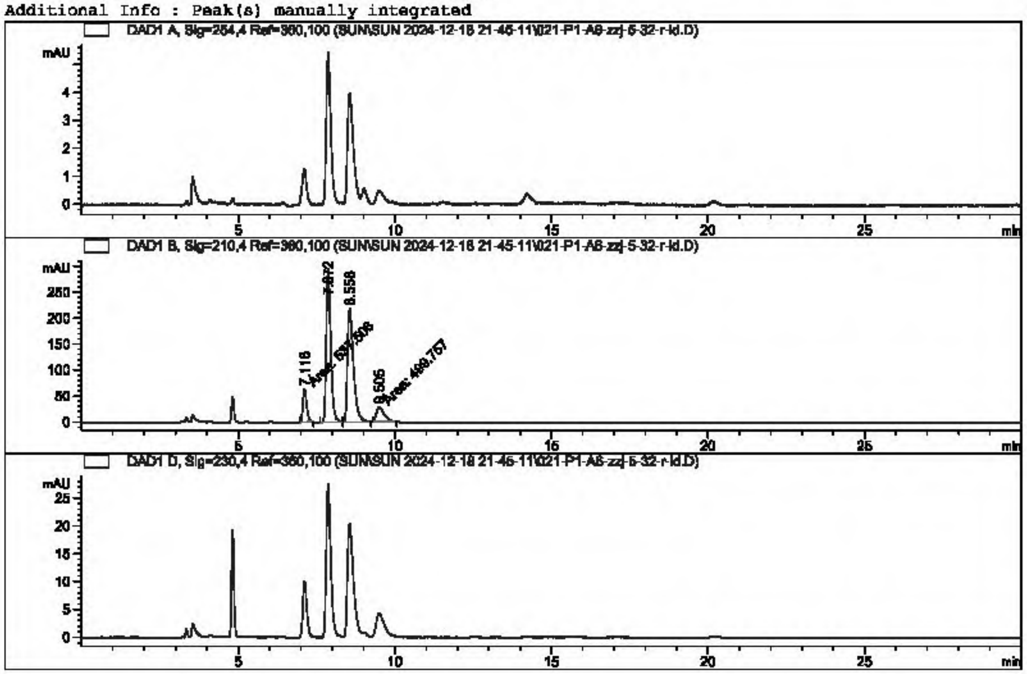

===

Area Percent Report

===

Sorted By : Signal
Multiplier : 1.0000
Dilution : 1.0000
Use Multiplier & Dilution Factor with ISTDs

Signal 1: DAD1 A, Sig=254,4 Ref=360,100

Data File C:\Users\P...tation\1\Data\SUN\SUN 2024-12-18 21-45-11\021-P1-A6-zzj-5-32-r-id.D
Sample Name: zzj-5-32-r-id

Signal 2: DAD1 B, Sig=210,4 Ref=360,100

Peak #	RetTime [min]	Type	Width [min]	Area [mAU*s]	Height [mAU]	Area %
1	7.118	MM	0.1421	537.50604	63.02570	7.7313
2	7.872	BV	0.1526	2956.66797	295.90262	42.5276
3	8.558	VV	0.2042	2958.42651	217.45087	42.5529
4	9.505	MM	0.2992	499.75735	27.83954	7.1883

Totals : 6952.35788 604.21872

Signal 3: DAD1 D, Sig=230,4 Ref=360,100

*** End of Report ***

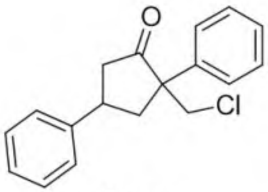

4a-rac

Data File C:\Users\P...ChemStation\1\Data\SUN\SUN 2024-12-20 13-44-15\031-P1-D1-zzj-5-32.D
Sample Name: zzj-5-32

===

Acq. Operator : SYSTEM Seq. Line : 31
Sample Operator : SYSTEM
Acq. Instrument : HPLC Location : P1-D-01
Injection Date : 20/12/2024 10:53:50 pm Inj : 1
Inj Volume : 2.000 µl
Different Inj Volume from Sample Entry! Actual Inj Volume : 20.000 µl
Method : C:\Users\Public\Documents\ChemStation\1\Data\SUN\SUN 2024-12-20 13-44-15\ID3-03-30.M (Sequence Method)
Last changed : 8/4/2024 9:59:11 am by SYSTEM
Additional Info : Peak(s) manually integrated

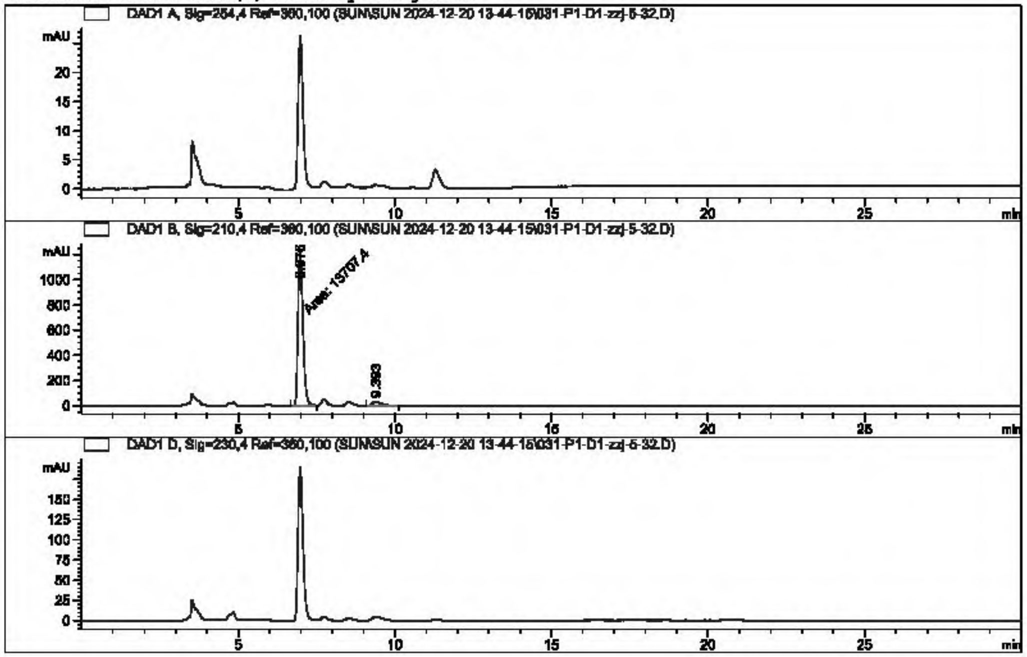

===

Area Percent Report

===

Sorted By : Signal
Multiplier : 1.0000
Dilution : 1.0000
Use Multiplier & Dilution Factor with ISTDs

Signal 1: DAD1 A, Sig=254,4 Ref=360,100

Data File C:\Users\P...ChemStation\1\Data\SUN\SUN 2024-12-20 13-44-15\031-P1-D1-zzj-5-32.D
Sample Name: zzj-5-32

Signal 2: DAD1 B, Sig=210,4 Ref=360,100

Peak #	RetTime [min]	Type	Width [min]	Area [mAU*s]	Height [mAU]	Area %
1	6.976	MM	0.1862	1.37074e4	1227.15833	95.6344
2	9.393	BB	0.2574	625.72174	30.75024	4.3656

Totals : 1.43332e4 1257.90856

Signal 3: DAD1 D, Sig=230,4 Ref=360,100

===

*** End of Report ***

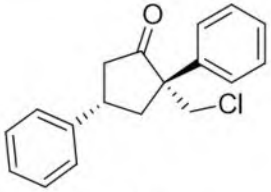

4a

Data File C:\Users\P...mStation\1\Data\SUN\SUN 2026-01-15 17-02-47\008-P1-A1-zzj-7-36-ic.D
Sample Name: zzj-7-36-ic

Acq. Operator : SYSTEM Seq. Line : 8
Sample Operator : SYSTEM
Acq. Instrument : HPLC Location : P1-A-01
Injection Date : 15/1/2026 7:03:29 pm Inj : 1
Inj Volume : 2.000 µl
Different Inj Volume from Sample Entry! Actual Inj Volume : 20.000 µl
Method : C:\Users\Public\Documents\ChemStation\1\Data\SUN\SUN 2026-01-15 17-02-47\IC3-03-40.M (Sequence Method)
Last changed : 3/4/2023 9:32:18 am by SYSTEM
Additional Info : Peak(s) manually integrated

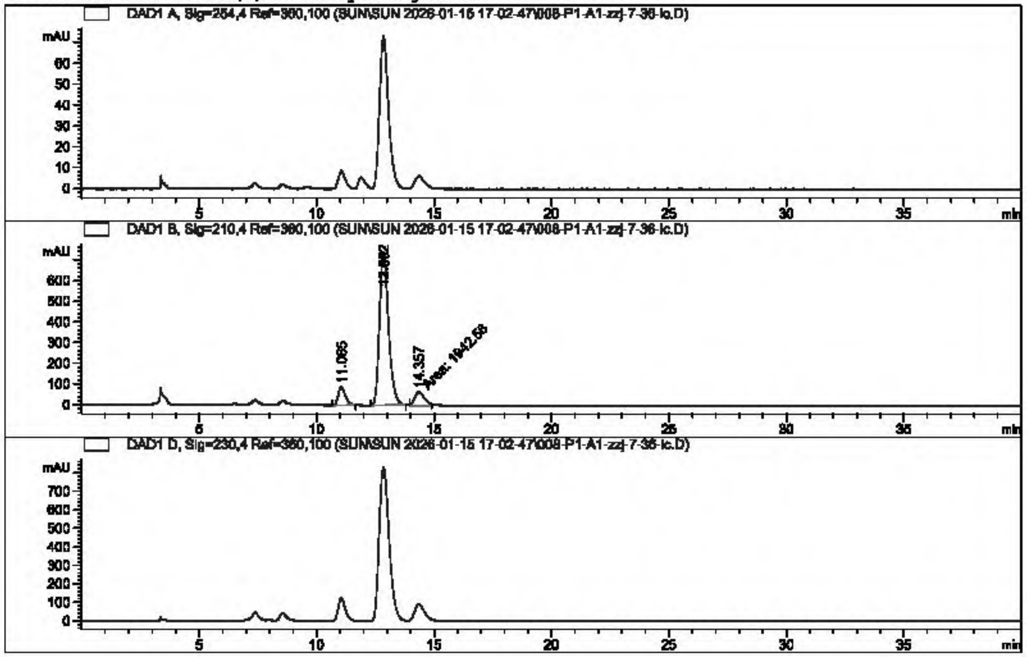

Area Percent Report

Sorted By : Signal
Multiplier : 1.0000
Dilution : 1.0000
Use Multiplier & Dilution Factor with ISTDs

Signal 1: DAD1 A, Sig=254,4 Ref=360,100

Data File C:\Users\P...mStation\1\Data\SUN\SUN 2026-01-15 17-02-47\008-P1-A1-zzj-7-36-ic.D
Sample Name: zzj-7-36-ic

Signal 2: DAD1 B, Sig=210,4 Ref=360,100

Peak #	RetTime [min]	Type	Width [min]	Area [mAU*s]	Height [mAU]	Area %
1	11.065	VV R	0.2476	1834.78589	88.03583	7.8014
2	12.862	BV R	0.3165	1.97414e4	739.31604	83.9390
3	14.357	MM	0.4886	1942.56226	66.25981	8.2596

Totals : 2.35188e4 893.61168

Signal 3: DAD1 D, Sig=230,4 Ref=360,100

*** End of Report ***

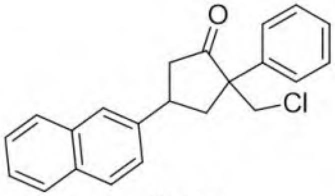

4b-rac

```
Acq. Operator   : SYSTEM                        Seq. Line :  12
Sample Operator : SYSTEM
Acq. Instrument : HPLC                           Location :  P1-A-02
Injection Date  : 15/1/2026 8:48:32 pm                Inj :  1
                                              Inj Volume : 2.000 µl
Different Inj Volume from Sample Entry!  Actual Inj Volume : 20.000 µl
Method          : C:\Users\Public\Documents\ChemStation\1\Data\SUN\SUN 2026-01-15 17-02-47\IC3-
                  03-40.M (Sequence Method)
Last changed    : 3/4/2023 9:32:18 am by SYSTEM
Additional Info : Peak(s) manually integrated
```

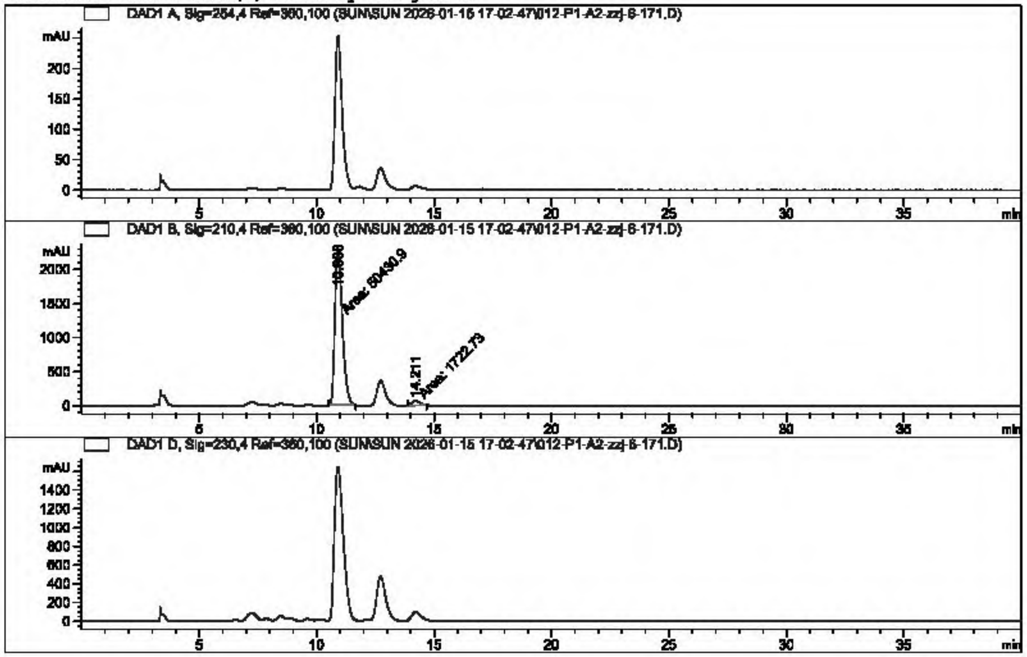

Area Percent Report

```
Sorted By             :      Signal
Multiplier            :      1.0000
Dilution              :      1.0000
Use Multiplier & Dilution Factor with ISTDs
```

Signal 1: DAD1 A, Sig=254,4 Ref=360,100

Data File C:\Users\P...hemStation\1\Data\SUN\SUN 2026-01-15 17-02-47\012-P1-A2-zzj-6-171.D
Sample Name: zzj-6-171

Signal 2: DAD1 B, Sig=210,4 Ref=360,100

Peak #	RetTime [min]	Type	Width [min]	Area [mAU*s]	Height [mAU]	Area %
1	10.908	MM	0.3740	5.04309e4	2247.58276	96.6968
2	14.211	MM	0.4322	1722.72925	66.43024	3.3032

Totals : 5.21537e4 2314.01301

Signal 3: DAD1 D, Sig=230,4 Ref=360,100

*** End of Report ***

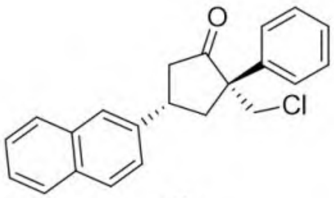

4b

Data File C:\Users\P...tation\1\Data\SUN\SUN 2026-03-17 21-27-27\002-P2-C1-zzj-7-88-2-oz.D
Sample Name: zzj-7-88-2-oz

===

Acq. Operator	: SYSTEM	Seq. Line :	2
Sample Operator	: SYSTEM		
Acq. Instrument	: HPLC	Location :	P2-C-01
Injection Date	: 17/3/2026 9:42:10 pm	Inj :	1
		Inj Volume :	2.000 µl

Different Inj Volume from Sample Entry! Actual Inj Volume : 10.000 µl
Acq. Method : C:\Users\Public\Documents\ChemStation\1\Data\SUN\SUN 2026-03-17 21-27-27\OZ3-05-20.M
Last changed : 6/3/2026 7:19:13 pm by SYSTEM
Analysis Method : C:\Users\Public\Documents\ChemStation\1\Data\SUN\SUN 2026-03-17 21-27-27\OZ3-05-20.M (Sequence Method)
Last changed : 7/5/2026 7:33:38 pm by SYSTEM
(modified after loading)
Additional Info : Peak(s) manually integrated

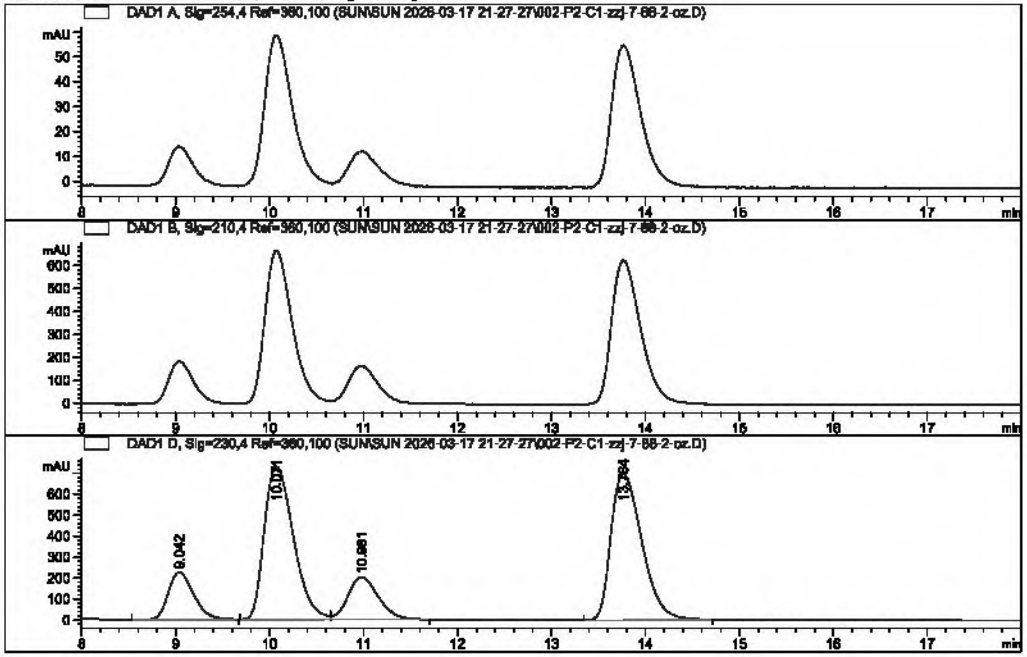

===

Area Percent Report

===

Sorted By	:	Signal
Multiplier	:	1.0000
Dilution	:	1.0000

Use Multiplier & Dilution Factor with ISTDs

Data File C:\Users\P...tation\1\Data\SUN\SUN 2026-03-17 21-27-27\002-P2-C1-zzj-7-88-2-oz.D
Sample Name: zzj-7-88-2-oz

Signal 1: DAD1 A, Sig=254,4 Ref=360,100

Signal 2: DAD1 B, Sig=210,4 Ref=360,100

Signal 3: DAD1 D, Sig=230,4 Ref=360,100

Peak #	RetTime [min]	Type	Width [min]	Area [mAU*s]	Height [mAU]	Area %
1	9.042	VV R	0.2597	4244.77881	225.45750	10.4750
2	10.071	BV	0.2581	1.57597e4	730.75574	38.8906
3	10.981	VV R	0.2667	4545.87891	201.89355	11.2180
4	13.764	VV R	0.2741	1.59728e4	696.34137	39.4165

Totals : 4.05231e4 1854.44817

===

*** End of Report ***

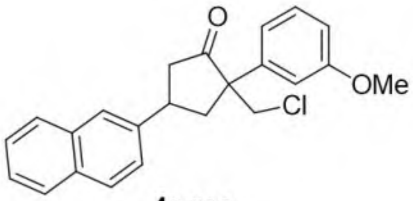

4c-rac

Acq. Operator	: SYSTEM	Seq. Line :	2
Sample Operator	: SYSTEM		
Acq. Instrument	: HPLC	Location :	P2-C-01
Injection Date	: 19/3/2026 3:22:06 pm	Inj :	1
		Inj Volume :	2.000 µl

Different Inj Volume from Sample Entry! Actual Inj Volume : 5.000 µl
Acq. Method : C:\Users\Public\Documents\ChemStation\1\Data\SUN\SUN 2026-03-19 15-07-53\OZ3-05-20.M
Last changed : 6/3/2026 7:19:13 pm by SYSTEM
Analysis Method : C:\Users\Public\Documents\ChemStation\1\Data\SUN\SUN 2026-03-19 15-07-53\OZ3-05-20.M (Sequence Method)
Last changed : 7/5/2026 7:36:07 pm by SYSTEM
(modified after loading)
Additional Info : Peak(s) manually integrated

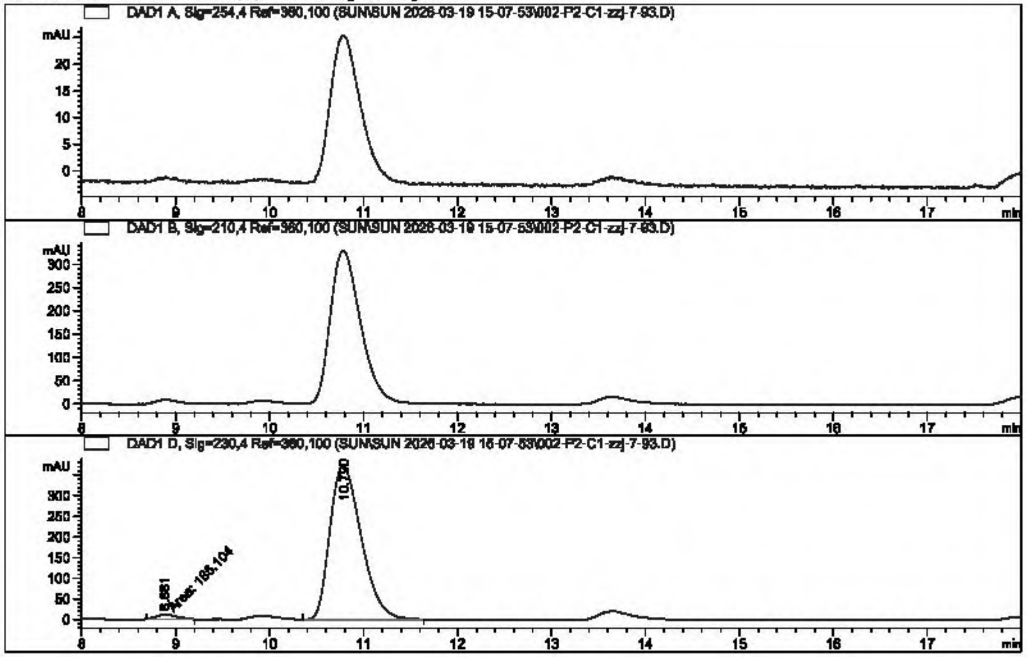

Area Percent Report

Sorted By	:	Signal
Multiplier	:	1.0000
Dilution	:	1.0000

Use Multiplier & Dilution Factor with ISTDs

Signal 1: DAD1 A, Sig=254,4 Ref=360,100

Signal 2: DAD1 B, Sig=210,4 Ref=360,100

Signal 3: DAD1 D, Sig=230,4 Ref=360,100

Peak #	RetTime [min]	Type	Width [min]	Area [mAU*s]	Height [mAU]	Area %
1	8.881	MM	0.2680	185.10446	11.50974	2.0985
2	10.790	VV R	0.2775	8635.83789	371.06482	97.9015

Totals : 8820.94235 382.57456

*** End of Report ***

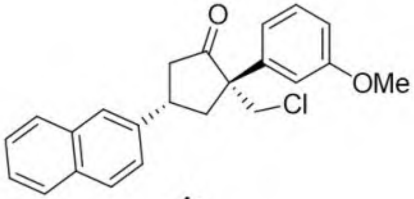

4c

```
Acq. Operator   : SYSTEM                      Seq. Line :  12
Sample Operator : SYSTEM
Acq. Instrument : HPLC                         Location :  P1-A-01
Injection Date  : 25/2/2026 6:48:23 pm              Inj :  1
                                             Inj Volume : 2.000 µl
Different Inj Volume from Sample Entry!  Actual Inj Volume : 10.000 µl
Method          : C:\Users\Public\Documents\ChemStation\1\Data\SUN\SUN 2026-02-25 15-35-10\OZ3-5
                  -30.M (Sequence Method)
Last changed    : 25/8/2024 11:48:27 am by SYSTEM
Additional Info : Peak(s) manually integrated
```

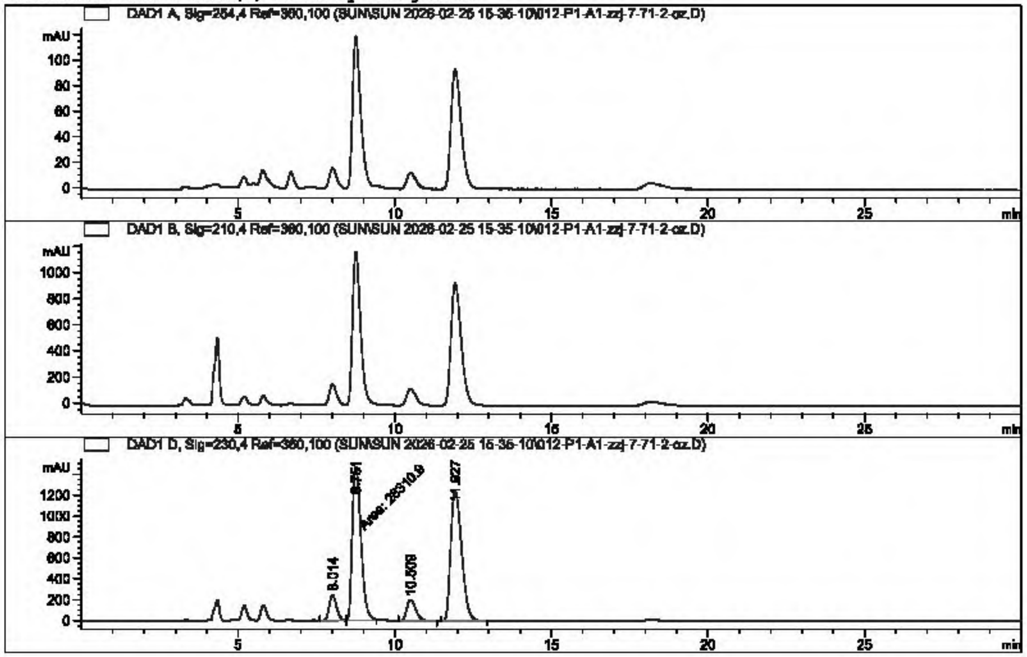

Area Percent Report

```
Sorted By              :      Signal
Multiplier             :      1.0000
Dilution               :      1.0000
Use Multiplier & Dilution Factor with ISTDs
```

Signal 1: DAD1 A, Sig=254,4 Ref=360,100

Data File C:\Users\P...tation\1\Data\SUN\SUN 2026-02-25 15-35-10\012-P1-A1-zzj-7-71-2-oz.D
Sample Name: zzj-7-71-2-oz

Signal 2: DAD1 B, Sig=210,4 Ref=360,100

Signal 3: DAD1 D, Sig=230,4 Ref=360,100

Peak #	RetTime [min]	Type	Width [min]	Area [mAU*s]	Height [mAU]	Area %
1	8.014	BB	0.2293	4056.62280	248.64893	6.0760
2	8.751	MM	0.3222	2.83109e4	1464.26379	42.4042
3	10.509	BV R	0.2792	4155.91797	203.15636	6.2248
4	11.927	VV R	0.2881	3.02410e4	1239.46912	45.2951

Totals : 6.67644e4 3155.53819

*** End of Report ***

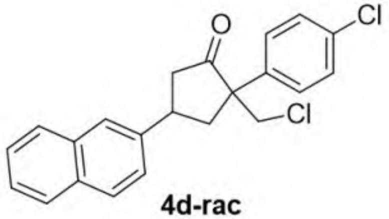

4d-rac

```
Acq. Operator   : SYSTEM                        Seq. Line :   2
Sample Operator : SYSTEM
Acq. Instrument : HPLC                           Location :   P1-A-01
Injection Date  : 28/2/2026 10:32:02 am               Inj :   1
                                           Inj Volume : 2.000 µl
Different Inj Volume from Sample Entry!  Actual Inj Volume : 5.000 µl
Method          : C:\Users\Public\Documents\ChemStation\1\Data\SUN\SUN 2026-02-28 10-22-40\OZ3-5
                  -30.M (Sequence Method)
Last changed    : 25/8/2024 11:48:27 am by SYSTEM
Additional Info : Peak(s) manually integrated
```

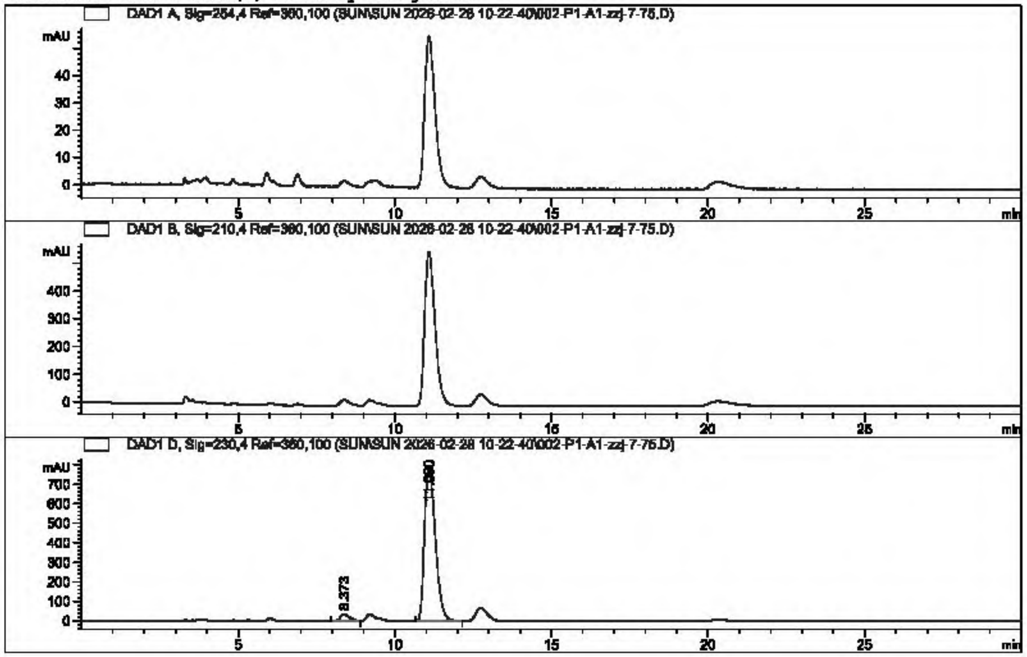

Area Percent Report

```
Sorted By             :      Signal
Multiplier            :      1.0000
Dilution              :      1.0000
Use Multiplier & Dilution Factor with ISTDs
```

Signal 1: DAD1 A, Sig=254,4 Ref=360,100

Data File C:\Users\P...ChemStation\1\Data\SUN\SUN 2026-02-28 10-22-40\002-P1-A1-zzj-7-75.D
Sample Name: zzj-7-75

Signal 2: DAD1 B, Sig=210,4 Ref=360,100

Signal 3: DAD1 D, Sig=230,4 Ref=360,100

Peak #	RetTime [min]	Type	Width [min]	Area [mAU*s]	Height [mAU]	Area %
1	8.373	VV R	0.2479	744.37054	35.53163	3.7637
2	11.090	VV R	0.2887	1.90330e4	788.30450	96.2363

Totals : 1.97774e4 823.83614

*** End of Report ***

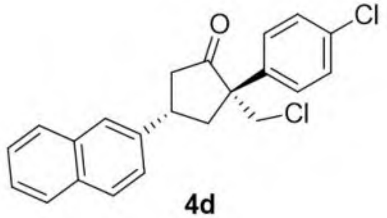

4d

Acq. Operator : SYSTEM Seq. Line : 52
Sample Operator : SYSTEM
Acq. Instrument : HPLC Location : P2-C-03
Injection Date : 8/4/2026 9:27:02 am Inj : 1
Inj Volume : 2.000 µl
Different Inj Volume from Sample Entry! Actual Inj Volume : 15.000 µl
Acq. Method : C:\Users\Public\Documents\ChemStation\1\Data\SUN\SUN 2026-04-07 12-12-40\OD3-15-20.M
Last changed : 15/4/2023 3:00:25 pm by SYSTEM
Analysis Method : C:\Users\Public\Documents\ChemStation\1\Data\SUN\SUN 2026-04-07 12-12-40\OD3-15-20.M (Sequence Method)
Last changed : 7/5/2026 7:46:49 pm by SYSTEM
(modified after loading)
Additional Info : Peak(s) manually integrated

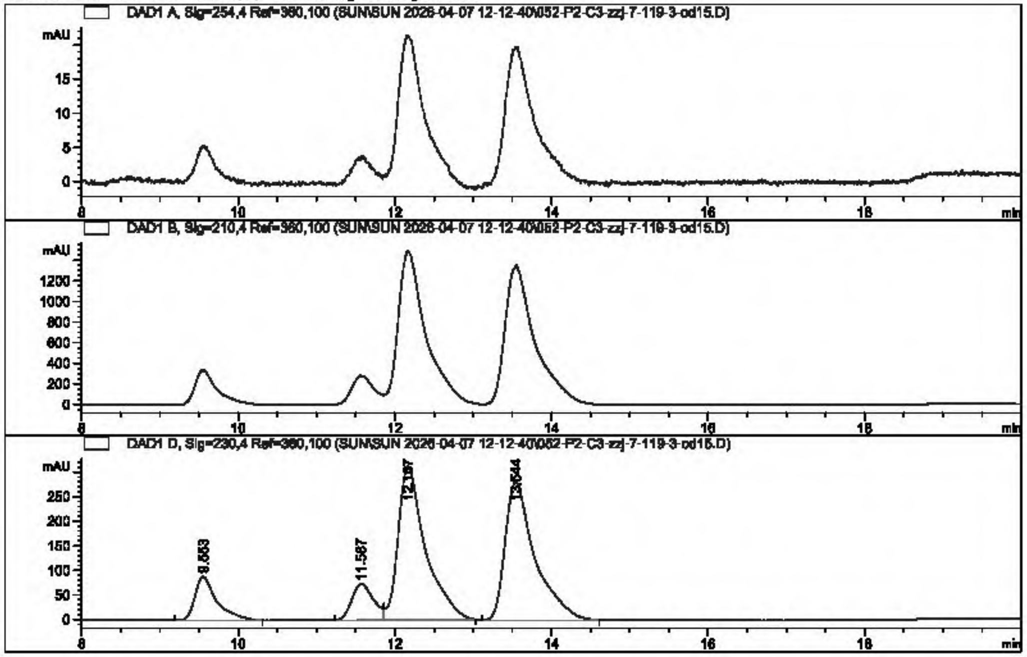

Area Percent Report

Sorted By : Signal
Multiplier : 1.0000
Dilution : 1.0000
Use Multiplier & Dilution Factor with ISTDs

Data File C:\Users\P...ion\1\Data\SUN\SUN 2026-04-07 12-12-40\052-P2-C3-zzj-7-119-3-od15.D
Sample Name: zzj-7-119-3-od15

Signal 1: DAD1 A, Sig=254,4 Ref=360,100

Signal 2: DAD1 B, Sig=210,4 Ref=360,100

Signal 3: DAD1 D, Sig=230,4 Ref=360,100

Peak #	RetTime [min]	Type	Width [min]	Area [mAU*s]	Height [mAU]	Area %
1	9.553	VV R	0.2343	1698.22327	88.12160	9.3698
2	11.567	BV	0.2303	1400.59680	72.67616	7.7277
3	12.167	VV R	0.3075	7569.51172	312.01486	41.7641
4	13.544	VV R	0.3164	7456.12305	280.25406	41.1385

Totals : 1.81245e4 753.06667

*** End of Report ***

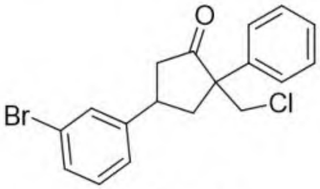

4e-rac

Data File C:\Users\P...ion\1\Data\SUN\SUN 2026-04-07 12-12-40\053-P2-C6-zzj-7-119-6-od15.D
Sample Name: zzj-7-119-6-od15

```
=====================================================================
Acq. Operator   : SYSTEM                        Seq. Line :  53
Sample Operator : SYSTEM
Acq. Instrument : HPLC                           Location :   P2-C-06
Injection Date  : 8/4/2026 9:48:56 am                 Inj :   1
                                               Inj Volume : 2.000 µl
Different Inj Volume from Sample Entry!  Actual Inj Volume : 5.000 µl
Acq. Method     : C:\Users\Public\Documents\ChemStation\1\Data\SUN\SUN 2026-04-07 12-12-40\OD3-
                  15-20.M
Last changed    : 15/4/2023 3:00:25 pm by SYSTEM
Analysis Method : C:\Users\Public\Documents\ChemStation\1\Data\SUN\SUN 2026-04-07 12-12-40\OD3-
                  15-20.M (Sequence Method)
Last changed    : 7/5/2026 7:50:07 pm by SYSTEM
                  (modified after loading)
Additional Info : Peak(s) manually integrated
```

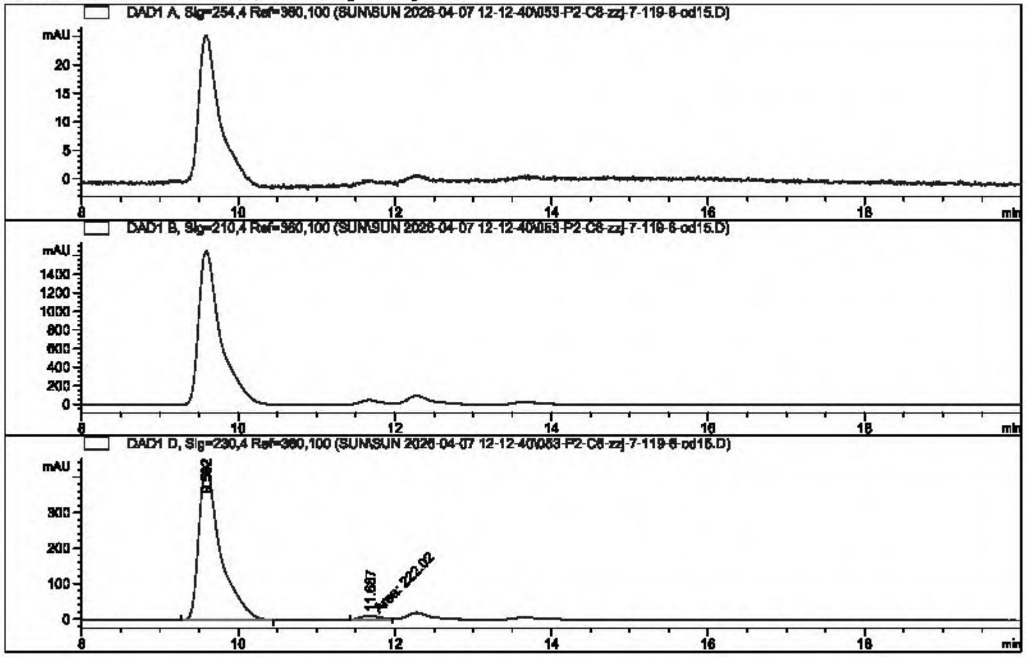


```
=====================================================================
                        Area Percent Report
=====================================================================

Sorted By             :      Signal
Multiplier            :      1.0000
Dilution              :      1.0000
Use Multiplier & Dilution Factor with ISTDs
```


Data File C:\Users\P...ion\1\Data\SUN\SUN 2026-04-07 12-12-40\053-P2-C6-zzj-7-119-6-od15.D
Sample Name: zzj-7-119-6-od15

Signal 1: DAD1 A, Sig=254,4 Ref=360,100

Signal 2: DAD1 B, Sig=210,4 Ref=360,100

Signal 3: DAD1 D, Sig=230,4 Ref=360,100

Peak #	RetTime [min]	Type	Width [min]	Area [mAU*s]	Height [mAU]	Area %
1	9.592	VB R	0.2606	8325.90527	432.51523	97.4026
2	11.687	MM	0.2894	222.01991	12.78592	2.5974

Totals : 8547.92519 445.30115

*** End of Report ***

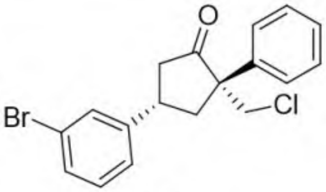

4e

Data File C:\Users\P...on\1\Data\SUN\SUN 2026-05-09 10-31-25\004-P2-C1-zzj-7-119-2-ibn01.D
Sample Name: zzj-7-119-2-ibn01

```
=====================================================================
Acq. Operator   : SYSTEM                        Seq. Line :   4
Sample Operator : SYSTEM
Acq. Instrument : HPLC                           Location :   P2-C-01
Injection Date  : 9/5/2026 11:27:05 am                Inj :   1
                                              Inj Volume : 2.000 µl
Different Inj Volume from Sample Entry!  Actual Inj Volume : 20.000 µl
Method          : C:\Users\Public\Documents\ChemStation\1\Data\SUN\SUN 2026-05-09 10-31-25\IBN3-
                  01-40.M (Sequence Method)
Last changed    : 25/11/2023 2:29:44 pm by SYSTEM
Additional Info : Peak(s) manually integrated
```

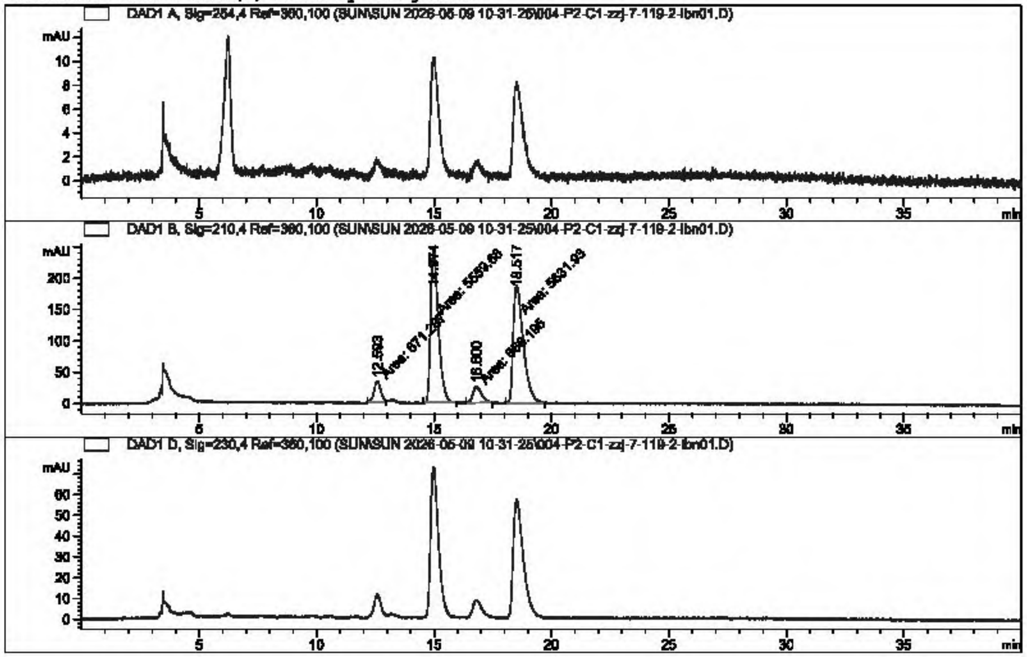


```
=====================================================================
                         Area Percent Report
=====================================================================
```

Sorted By : Signal
Multiplier : 1.0000
Dilution : 1.0000
Use Multiplier & Dilution Factor with ISTDs

Signal 1: DAD1 A, Sig=254,4 Ref=360,100

Data File C:\Users\P...on\1\Data\SUN\SUN 2026-05-09 10-31-25\004-P2-C1-zzj-7-119-2-ibn01.D
Sample Name: zzj-7-119-2-ibn01

Signal 2: DAD1 B, Sig=210,4 Ref=360,100

Peak #	RetTime [min]	Type	Width [min]	Area [mAU*s]	Height [mAU]	Area %
1	12.593	MM	0.3333	671.28717	33.56937	5.3578
2	14.974	MM	0.3857	5559.68115	240.27306	44.3742
3	16.800	MM	0.4227	666.19513	26.26972	5.3172
4	18.517	MM	0.4977	5631.93262	188.60481	44.9508

Totals : 1.25291e4 488.71696

Signal 3: DAD1 D, Sig=230,4 Ref=360,100

*** End of Report ***

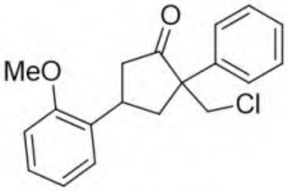

4f-rac

```
Acq. Operator   : SYSTEM                    Seq. Line :   8
Sample Operator : SYSTEM
Acq. Instrument : HPLC                       Location :   P2-C-01
Injection Date  : 9/5/2026 5:51:35 pm             Inj :   1
                                           Inj Volume : 2.000 µl
Different Inj Volume from Sample Entry!  Actual Inj Volume : 10.000 µl
Method          : C:\Users\Public\Documents\ChemStation\1\Data\SUN\SUN 2026-05-09 14-31-12\IBN3-
                  01-40.M (Sequence Method)
Last changed    : 25/11/2023 2:29:44 pm by SYSTEM
Additional Info : Peak(s) manually integrated
```

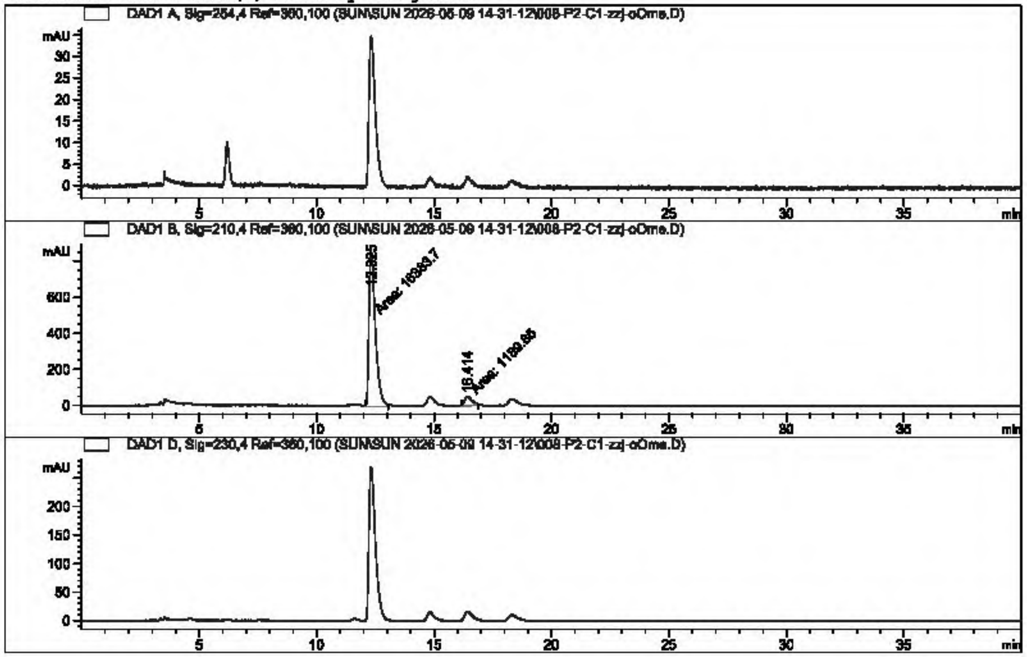

Area Percent Report

```
Sorted By             :      Signal
Multiplier            :      1.0000
Dilution              :      1.0000
Use Multiplier & Dilution Factor with ISTDs
```

Signal 1: DAD1 A, Sig=254,4 Ref=360,100

Data File C:\Users\P...ChemStation\1\Data\SUN\SUN 2026-05-09 14-31-12\008-P2-C1-zzj-oOme.D
Sample Name: zzj-oOme

Signal 2: DAD1 B, Sig=210,4 Ref=360,100

Peak #	RetTime [min]	Type	Width [min]	Area [mAU*s]	Height [mAU]	Area %
1	12.325	MM	0.3181	1.63837e4	858.29498	93.2293
2	16.414	MM	0.3824	1189.84851	51.85636	6.7707
Totals :				1.75736e4	910.15134	

Signal 3: DAD1 D, Sig=230,4 Ref=360,100

*** End of Report ***

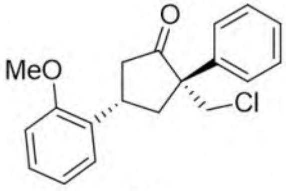

4f

```
Acq. Operator   : SYSTEM                        Seq. Line :  45
Sample Operator : SYSTEM
Acq. Instrument : HPLC                           Location :   P2-C-01
Injection Date  : 5/4/2026 5:29:14 pm                 Inj :   1
                                               Inj Volume : 2.000 µl
Different Inj Volume from Sample Entry!  Actual Inj Volume : 20.000 µl
Acq. Method     : C:\Users\Public\Documents\ChemStation\1\Data\SUN\SUN 2026-04-04 21-30-57\OX3-
                  10-30.M
Last changed    : 27/9/2022 9:53:02 am by SYSTEM
Analysis Method : C:\Users\Public\Documents\ChemStation\1\Data\SUN\SUN 2026-04-04 21-30-57\OX3-
                  10-30.M (Sequence Method)
Last changed    : 10/4/2026 2:42:10 pm by SYSTEM
                  (modified after loading)
Additional Info : Peak(s) manually integrated
```

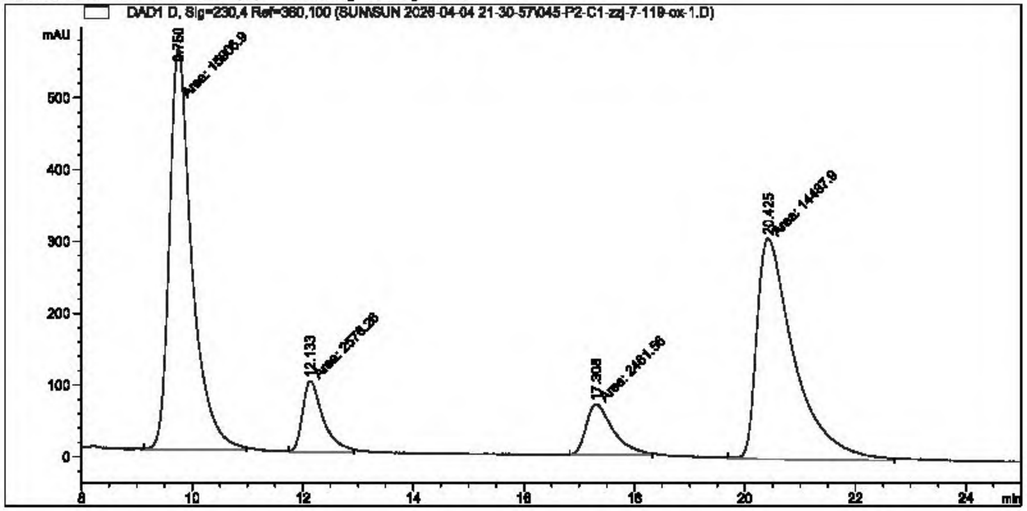

Area Percent Report

```
Sorted By             :      Signal
Multiplier            :      1.0000
Dilution              :      1.0000
Use Multiplier & Dilution Factor with ISTDs
```

Signal 1: DAD1 D, Sig=230,4 Ref=360,100

Peak #	RetTime [min]	Type	Width [min]	Area [mAU*s]	Height [mAU]	Area %
1	9.750	MM	0.4737	1.59059e4	559.62433	44.8919
2	12.133	MM	0.4341	2576.26050	98.90456	7.2711
3	17.308	MM	0.5765	2461.55981	71.16553	6.9473
4	20.425	MM	0.7861	1.44879e4	307.16678	40.8897

Totals :   3.54317e4   1036.86119

*** End of Report ***

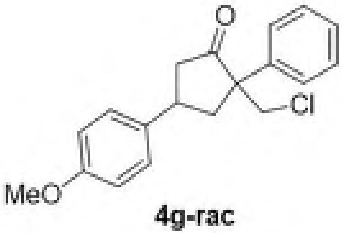

4g-rac

```
Acq. Operator   : SYSTEM                        Seq. Line :  15
Sample Operator : SYSTEM
Acq. Instrument : HPLC                           Location :   P2-C-01
Injection Date  : 10/4/2026 1:49:18 pm                Inj :   1
                                               Inj Volume : 2.000 µl
Different Inj Volume from Sample Entry!  Actual Inj Volume : 20.000 µl
Acq. Method     : C:\Users\Public\Documents\ChemStation\1\Data\SUN\SUN 2026-04-10 10-09-25\OX3-
                  10-30.M
Last changed    : 27/9/2022 9:53:02 am by SYSTEM
Analysis Method : C:\Users\Public\Documents\ChemStation\1\Data\SUN\SUN 2026-04-10 10-09-25\OX3-
                  10-30.M (Sequence Method)
Last changed    : 10/4/2026 2:46:58 pm by SYSTEM
                  (modified after loading)
Additional Info : Peak(s) manually integrated
```

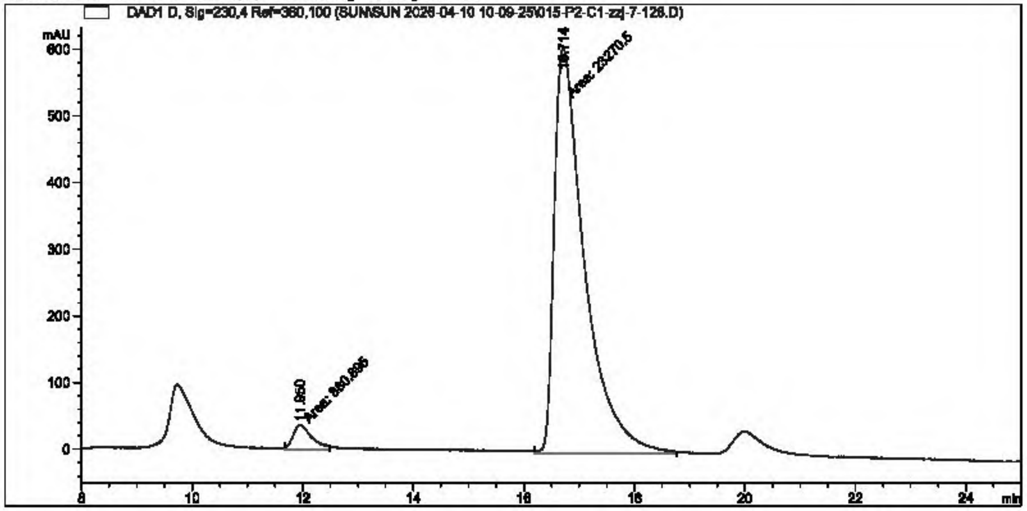

Area Percent Report

```
Sorted By             :      Signal
Multiplier            :      1.0000
Dilution              :      1.0000
Use Multiplier & Dilution Factor with ISTDs
```

Signal 1: DAD1 D, Sig=230,4 Ref=360,100

Peak #	RetTime [min]	Type	Width [min]	Area [mAU*s]	Height [mAU]	Area %
1	11.950	MM	0.4010	880.89465	36.61377	3.6474
2	16.714	MM	0.6376	2.32705e4	608.30780	96.3526

Totals : 2.41514e4 644.92157

Data File C:\Users\P...hemStation\1\Data\SUN\SUN 2026-04-10 10-09-25\015-P2-C1-zzj-7-126.D
Sample Name: zzj-7-126

*** End of Report ***

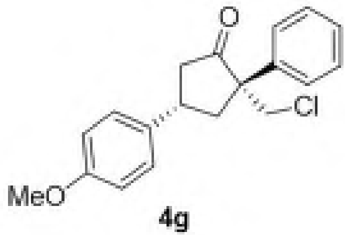

4g

Data File C:\Users\P...tation\1\Data\SUN\SUN 2025-02-18 15-42-37\032-P1-E3-zzj-5-71-1-ox.D
Sample Name: zzj-5-71-1-ox

===

Acq. Operator : SYSTEM Seq. Line : 32
Sample Operator : SYSTEM
Acq. Instrument : HPLC Location : P1-E-03
Injection Date : 19/2/2025 1:04:28 am Inj : 1
Inj Volume : 2.000 µl
Different Inj Volume from Sample Entry! Actual Inj Volume : 5.000 µl
Acq. Method : C:\Users\Public\Documents\ChemStation\1\Data\SUN\SUN 2025-02-18 15-42-37\OX3-01-40.M
Last changed : 7/10/2022 10:57:41 pm by SYSTEM
Analysis Method : C:\Users\Public\Documents\ChemStation\1\Data\SUN\SUN 2025-02-18 15-42-37\OX3-01-40.M (Sequence Method)
Last changed : 13/12/2025 3:20:59 pm by SYSTEM
(modified after loading)
Additional Info : Peak(s) manually integrated

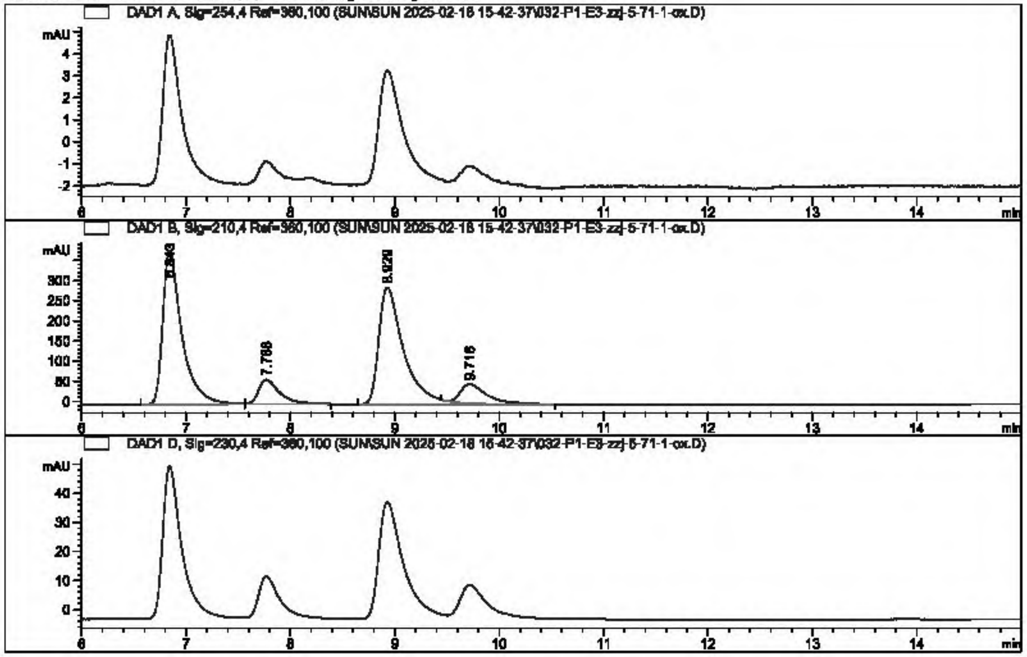

===

Area Percent Report

===

Sorted By : Signal
Multiplier : 1.0000
Dilution : 1.0000
Use Multiplier & Dilution Factor with ISTDs

Data File C:\Users\P...tation\1\Data\SUN\SUN 2025-02-18 15-42-37\032-P1-E3-zzj-5-71-1-ox.D
Sample Name: zzj-5-71-1-ox

Signal 1: DAD1 A, Sig=254,4 Ref=360,100

Signal 2: DAD1 B, Sig=210,4 Ref=360,100

Peak #	RetTime [min]	Type	Width [min]	Area [mAU*s]	Height [mAU]	Area %
1	6.843	BV	0.1865	4835.75781	381.12085	42.4658
2	7.768	VV R	0.1990	860.12921	61.17213	7.5533
3	8.929	BV R	0.2447	4827.88867	290.08328	42.3967
4	9.716	VB E	0.2498	863.64581	47.90570	7.5842

Totals : 1.13874e4 780.28197

Signal 3: DAD1 D, Sig=230,4 Ref=360,100

===

*** End of Report ***

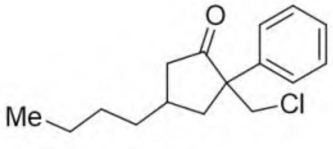

4h-rac

Data File C:\Users\P...mStation\1\Data\SUN\SUN 2025-03-05 09-50-36\004-P1-E1-zzj-5-73-ox.D
Sample Name: zzj-5-73-ox

```
Acq. Operator   : SYSTEM                        Seq. Line :    4
Sample Operator : SYSTEM
Acq. Instrument : HPLC                           Location :   P1-E-01
Injection Date  : 5/3/2025 10:45:44 am                Inj :    1
                                               Inj Volume : 2.000 µl
Different Inj Volume from Sample Entry!  Actual Inj Volume : 5.000 µl
Acq. Method     : C:\Users\Public\Documents\ChemStation\1\Data\SUN\SUN 2025-03-05 09-50-36\OX3-
                  01-20.M
Last changed    : 8/10/2022 12:00:39 pm by SYSTEM
Analysis Method : C:\Users\Public\Documents\ChemStation\1\Data\SUN\SUN 2025-03-05 09-50-36\OX3-
                  01-20.M (Sequence Method)
Last changed    : 13/12/2025 3:25:32 pm by SYSTEM
                  (modified after loading)
Additional Info : Peak(s) manually integrated
```

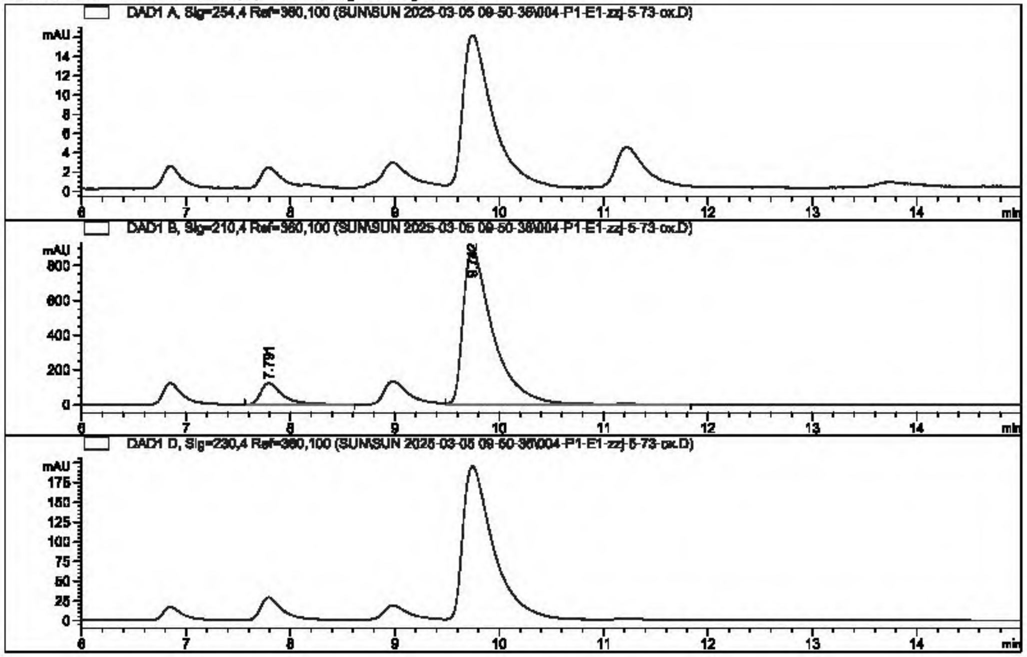

Area Percent Report

```
Sorted By        :     Signal
Multiplier       :     1.0000
Dilution         :     1.0000
Use Multiplier & Dilution Factor with ISTDs
```


Data File C:\Users\P...mStation\1\Data\SUN\SUN 2025-03-05 09-50-36\004-P1-E1-zzj-5-73-ox.D
Sample Name: zzj-5-73-ox

Signal 1: DAD1 A, Sig=254,4 Ref=360,100

Signal 2: DAD1 B, Sig=210,4 Ref=360,100

Peak #	RetTime [min]	Type	Width [min]	Area [mAU*s]	Height [mAU]	Area %
1	7.791	VV R	0.2121	1777.90393	123.05591	9.0478
2	9.742	VV R	0.2839	1.78723e4	886.11438	90.9522
Totals :				1.96502e4	1009.17029	

Signal 3: DAD1 D, Sig=230,4 Ref=360,100

*** End of Report ***

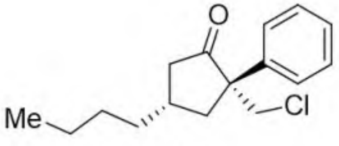

4h

```
Acq. Operator   : SYSTEM                        Seq. Line :  16
Sample Operator : SYSTEM
Acq. Instrument : HPLC                           Location :   P1-A-01
Injection Date  : 17/1/2026 5:24:44 pm                Inj :   1
                                               Inj Volume : 2.000 µl
Different Inj Volume from Sample Entry!  Actual Inj Volume : 20.000 µl
Method          : C:\Users\Public\Documents\ChemStation\1\Data\SUN\SUN 2026-01-17 11-54-01\IE3-
                  10-30.M (Sequence Method)
Last changed    : 26/9/2022 10:26:38 pm by SYSTEM
Additional Info : Peak(s) manually integrated
```

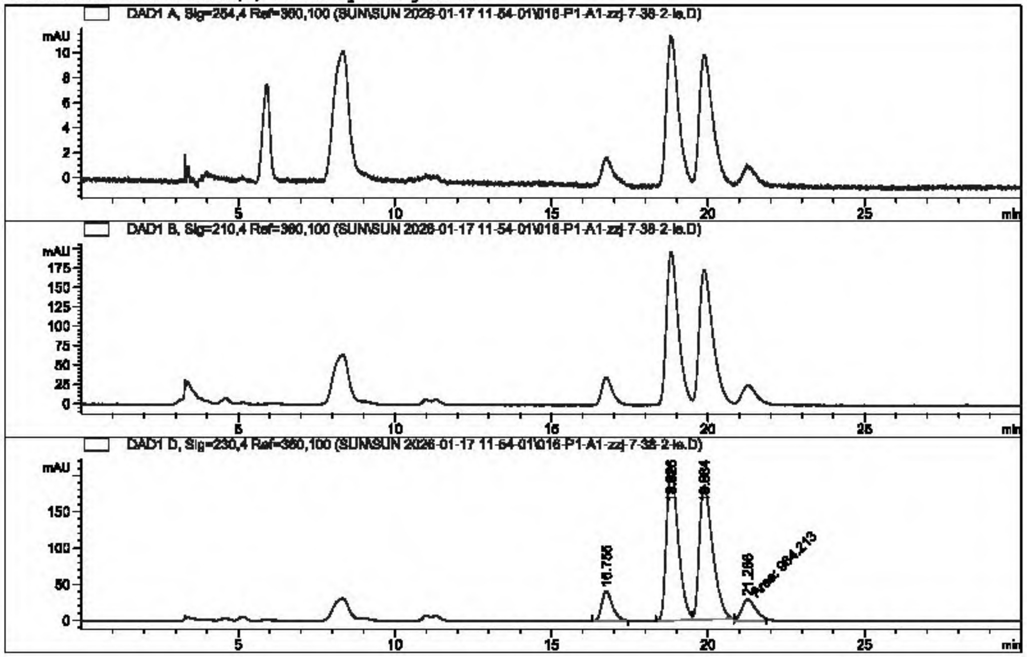

Area Percent Report

```
Sorted By          :     Signal
Multiplier         :     1.0000
Dilution           :     1.0000
Use Multiplier & Dilution Factor with ISTDs
```

Signal 1: DAD1 A, Sig=254,4 Ref=360,100

Data File C:\Users\P...tation\1\Data\SUN\SUN 2026-01-17 11-54-01\016-P1-A1-zzj-7-38-2-ie.D
Sample Name: zzj-7-38-2-ie

Signal 2: DAD1 B, Sig=210,4 Ref=360,100

Signal 3: DAD1 D, Sig=230,4 Ref=360,100

Peak #	RetTime [min]	Type	Width [min]	Area [mAU*s]	Height [mAU]	Area %
1	16.755	VV R	0.2799	980.95624	41.48576	7.5474
2	18.826	BV	0.3588	5549.07764	211.35822	42.6941
3	19.884	VB	0.3519	5503.04834	185.08588	42.3399
4	21.266	MM	0.5371	964.21320	29.92022	7.4186
Totals :				1.29973e4	467.85007	

*** End of Report ***

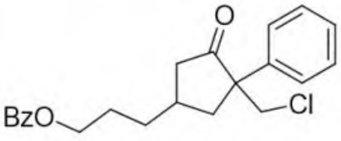

4i-rac

```
=====================================================================
Acq. Operator   : SYSTEM                    Seq. Line :   2
Sample Operator : SYSTEM
Acq. Instrument : HPLC                       Location :   P1-A-02
Injection Date  : 21/1/2026 10:52:03 pm           Inj :   1
                                          Inj Volume : 2.000 µl
Different Inj Volume from Sample Entry!  Actual Inj Volume : 10.000 µl
Method          : C:\Users\Public\Documents\ChemStation\1\Data\SUN\SUN 2026-01-21 22-39-07\IE3-
                  10-30.M (Sequence Method)
Last changed    : 26/9/2022 10:26:38 pm by SYSTEM
Additional Info : Peak(s) manually integrated
```

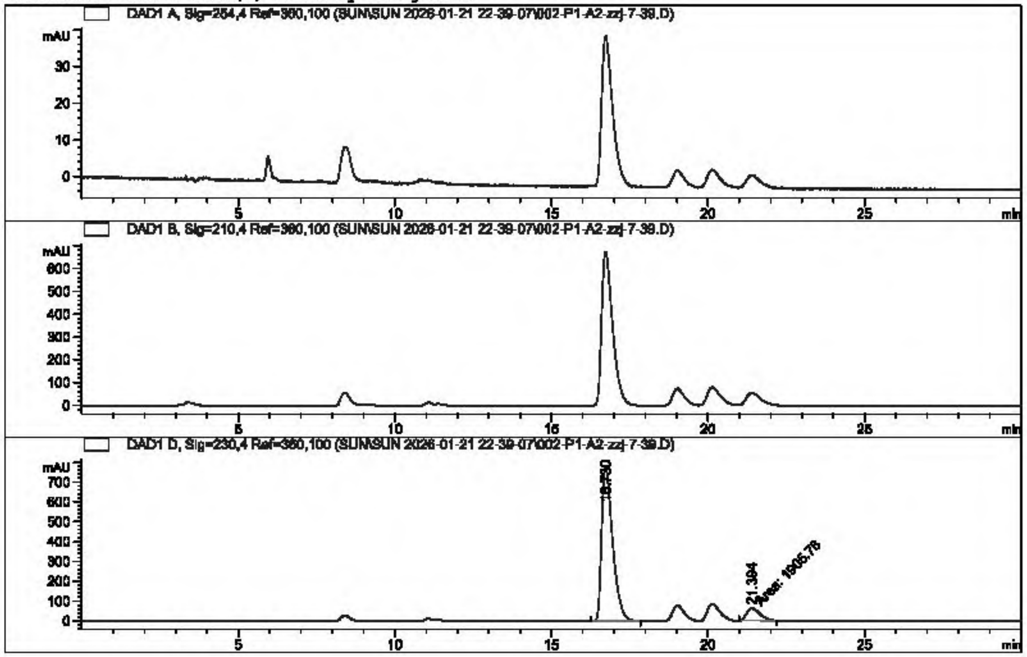


```
=====================================================================
                         Area Percent Report
=====================================================================

Sorted By             :      Signal
Multiplier            :      1.0000
Dilution              :      1.0000
Use Multiplier & Dilution Factor with ISTDs
```

Signal 1: DAD1 A, Sig=254,4 Ref=360,100

Data File C:\Users\P...ChemStation\1\Data\SUN\SUN 2026-01-21 22-39-07\002-P1-A2-zzj-7-39.D
Sample Name: zzj-7-39

Signal 2: DAD1 B, Sig=210,4 Ref=360,100

Signal 3: DAD1 D, Sig=230,4 Ref=360,100

Peak #	RetTime [min]	Type	Width [min]	Area [mAU*s]	Height [mAU]	Area %
1	16.730	VV R	0.3361	1.89293e4	774.69781	90.8531
2	21.394	MM	0.5071	1905.75818	62.63240	9.1469

Totals : 2.08351e4 837.33021

*** End of Report ***

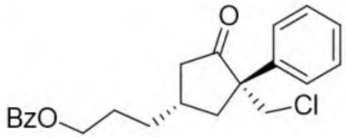

4i

Data File C:\Users\P...ation\1\Data\SUN\SUN 2025-01-18 10-06-17\007-P1-E1-zzj-5-50-3-rac.D
Sample Name: zzj-5-50-3-rac

```
=====================================================================
Acq. Operator   : SYSTEM                        Seq. Line :   7
Sample Operator : SYSTEM
Acq. Instrument : HPLC                           Location :   P1-E-01
Injection Date  : 18/1/2025 11:45:13 am               Inj :   1
                                               Inj Volume : 2.000 µl
Different Inj Volume from Sample Entry!  Actual Inj Volume : 1.000 µl
Method          : C:\Users\Public\Documents\ChemStation\1\Data\SUN\SUN 2025-01-18 10-06-17\IC3-
                  03-10.M (Sequence Method)
Last changed    : 3/4/2023 9:31:53 am by SYSTEM
Additional Info : Peak(s) manually integrated
```

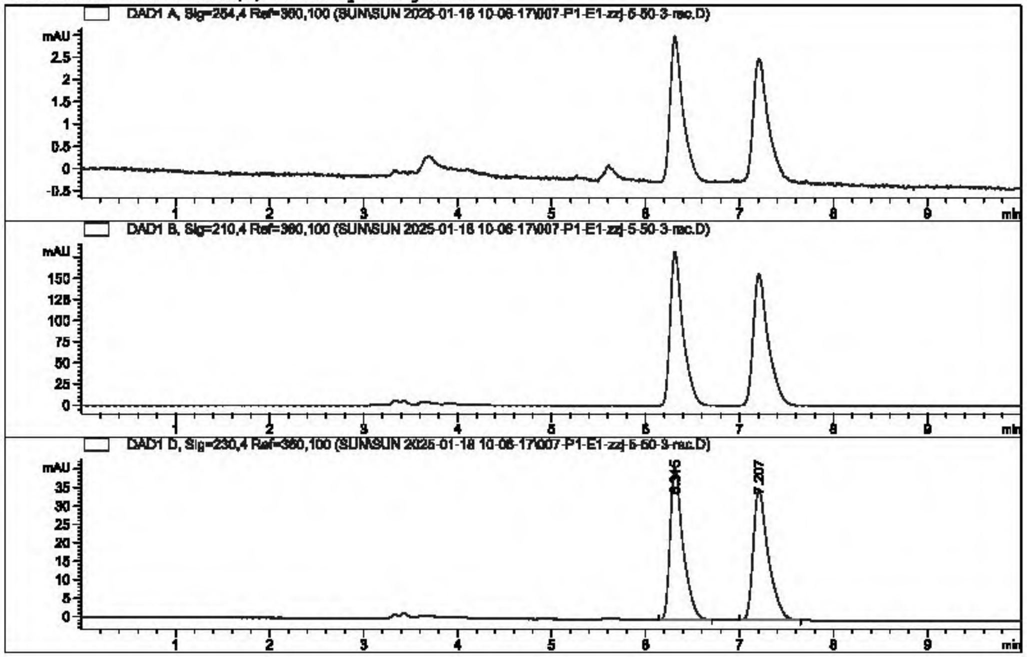


```
=====================================================================
                         Area Percent Report
=====================================================================

Sorted By             :      Signal
Multiplier            :      1.0000
Dilution              :      1.0000
Use Multiplier & Dilution Factor with ISTDs


Signal 1: DAD1 A, Sig=254,4 Ref=360,100
```


Data File C:\Users\P...ation\1\Data\SUN\SUN 2025-01-18 10-06-17\007-P1-E1-zzj-5-50-3-rac.D
Sample Name: zzj-5-50-3-rac

Signal 2: DAD1 B, Sig=210,4 Ref=360,100

Signal 3: DAD1 D, Sig=230,4 Ref=360,100

Peak #	RetTime [min]	Type	Width [min]	Area [mAU*s]	Height [mAU]	Area %
1	6.315	BB	0.1394	392.30472	41.28610	49.9891
2	7.207	BB	0.1611	392.47546	35.32145	50.0109

Totals : 784.78018 76.60755

*** End of Report ***

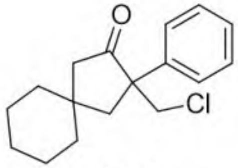

4j-rac

Acq. Operator : SYSTEM Seq. Line : 6
Sample Operator : SYSTEM
Acq. Instrument : HPLC Location : P1-E-02
Injection Date : 11/2/2025 12:32:29 pm Inj : 1
Inj Volume : 2.000 µl
Different Inj Volume from Sample Entry! Actual Inj Volume : 5.000 µl
Method : C:\Users\Public\Documents\ChemStation\1\Data\SUN\SUN 2025-02-11 11-14-57\IC3-03-10.M (Sequence Method)
Last changed : 3/4/2023 9:31:53 am by SYSTEM
Additional Info : Peak(s) manually integrated

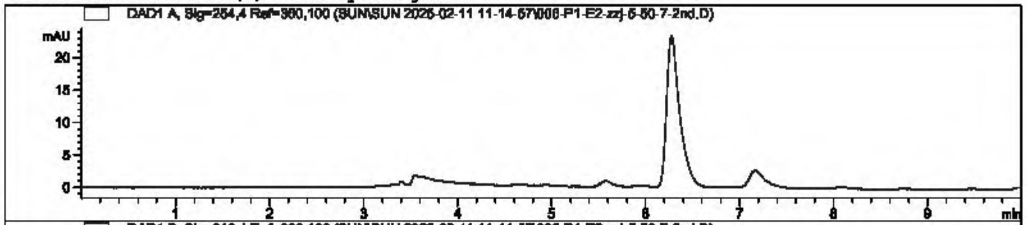

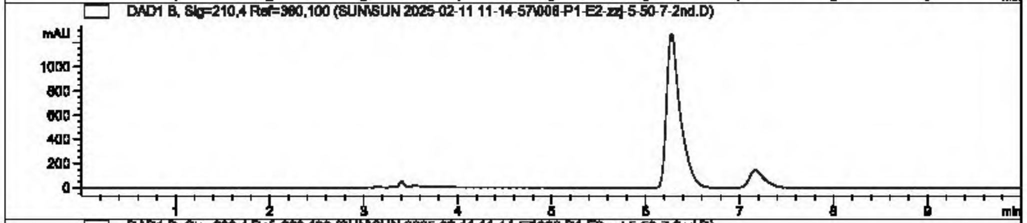

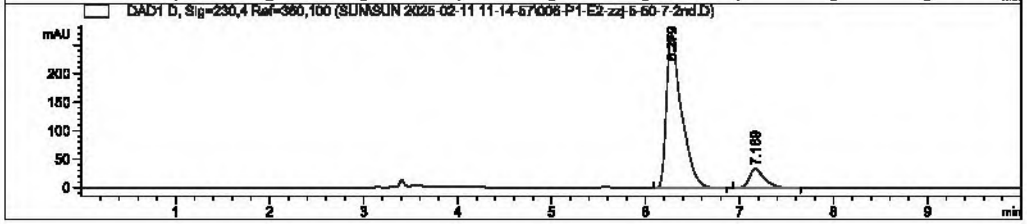

Area Percent Report

Sorted By : Signal
Multiplier : 1.0000
Dilution : 1.0000
Use Multiplier & Dilution Factor with ISTDs

Signal 1: DAD1 A, Sig=254,4 Ref=360,100

Data File C:\Users\P...ation\1\Data\SUN\SUN 2025-02-11 11-14-57\006-P1-E2-zzj-5-50-7-2nd.D
Sample Name: zzj-5-50-7-2nd

Signal 2: DAD1 B, Sig=210,4 Ref=360,100

Signal 3: DAD1 D, Sig=230,4 Ref=360,100

Peak #	RetTime [min]	Type	Width [min]	Area [mAU*s]	Height [mAU]	Area %
1	6.279	BB	0.1601	2983.47266	269.61353	88.9465
2	7.169	BB	0.1656	370.75909	33.13275	11.0535

Totals : 3354.23175 302.74627

*** End of Report ***

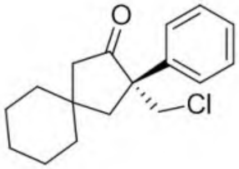

4j

===

Acq. Operator : SYSTEM Seq. Line : 8
Sample Operator : SYSTEM
Acq. Instrument : HPLC Location : P1-B-02
Injection Date : 15/11/2024 7:01:24 pm Inj : 1
Inj Volume : 2.000 µl
Different Inj Volume from Sample Entry! Actual Inj Volume : 10.000 µl
Acq. Method : C:\Users\Public\Documents\ChemStation\1\Data\SUN\SUN 2024-11-15 16-41-35\IBN3-05-30.M
Last changed : 29/8/2022 6:09:41 pm by SYSTEM
Analysis Method : C:\Users\Public\Documents\ChemStation\1\Data\SUN\SUN 2024-11-15 16-41-35\IBN3-05-30.M (Sequence Method)
Last changed : 18/6/2026 9:25:04 pm by SYSTEM
(modified after loading)
Additional Info : Peak(s) manually integrated

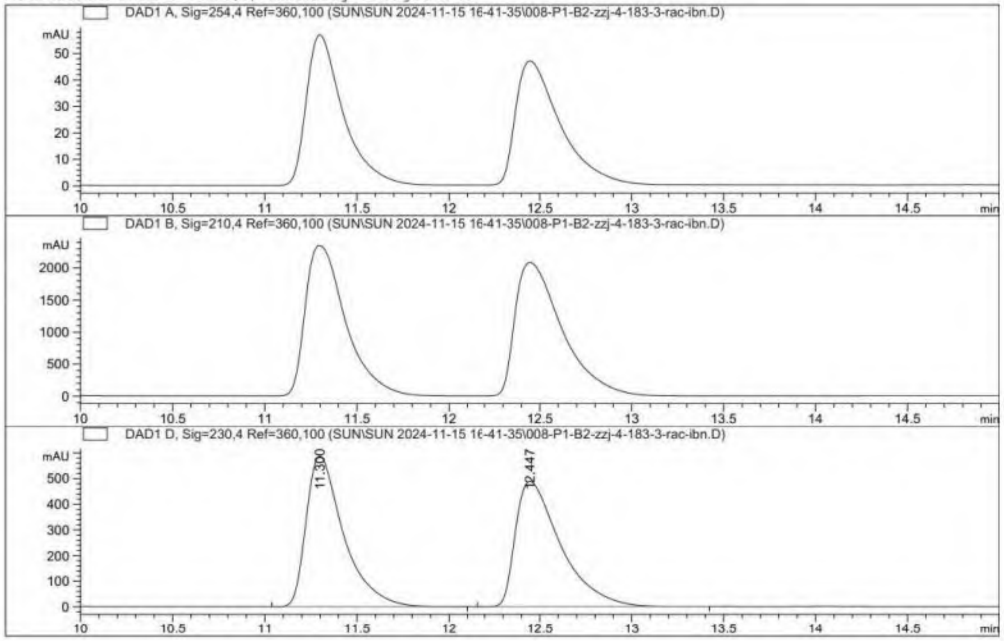

===

Area Percent Report

===

Sorted By : Signal
Multiplier : 1.0000
Dilution : 1.0000
Use Multiplier & Dilution Factor with ISTDs

Data File C:\Users\P...\1\Data\SUN\SUN 2024-11-15 16-41-35\008-P1-B2-zzj-4-183-3-rac-ibn.D
Sample Name: zzj-4-183-3-rac-ibn

Signal 1: DAD1 A, Sig=254,4 Ref=360,100

Signal 2: DAD1 B, Sig=210,4 Ref=360,100

Signal 3: DAD1 D, Sig=230,4 Ref=360,100

Peak #	RetTime [min]	Type	Width [min]	Area [mAU*s]	Height [mAU]	Area %
1	11.300	BB	0.2113	8263.31738	586.82837	49.8423
2	12.447	BB	0.2572	8315.60059	488.34119	50.1577

Totals : 1.65789e4 1075.16956

===

*** End of Report ***

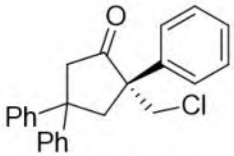

4k-rac

Data File C:\Users\P...hemStation\1\Data\SUN\SUN 2026-06-11 21-08-53\002-P2-C4-zzj-7-170.D
Sample Name: zzj-7-170

===

Acq. Operator : SYSTEM Seq. Line : 2
Sample Operator : SYSTEM
Acq. Instrument : HPLC Location : P2-C-04
Injection Date : 11/6/2026 9:22:40 pm Inj : 1
Inj Volume : 2.000 µl
Different Inj Volume from Sample Entry! Actual Inj Volume : 5.000 µl
Acq. Method : C:\Users\Public\Documents\ChemStation\1\Data\SUN\SUN 2026-06-11 21-08-53\IBN3-05-30.M
Last changed : 29/8/2022 6:09:41 pm by SYSTEM
Analysis Method : C:\Users\Public\Documents\ChemStation\1\Data\SUN\SUN 2026-06-11 21-08-53\IBN3-05-30.M (Sequence Method)
Last changed : 18/6/2026 9:26:56 pm by SYSTEM
(modified after loading)
Additional Info : Peak(s) manually integrated

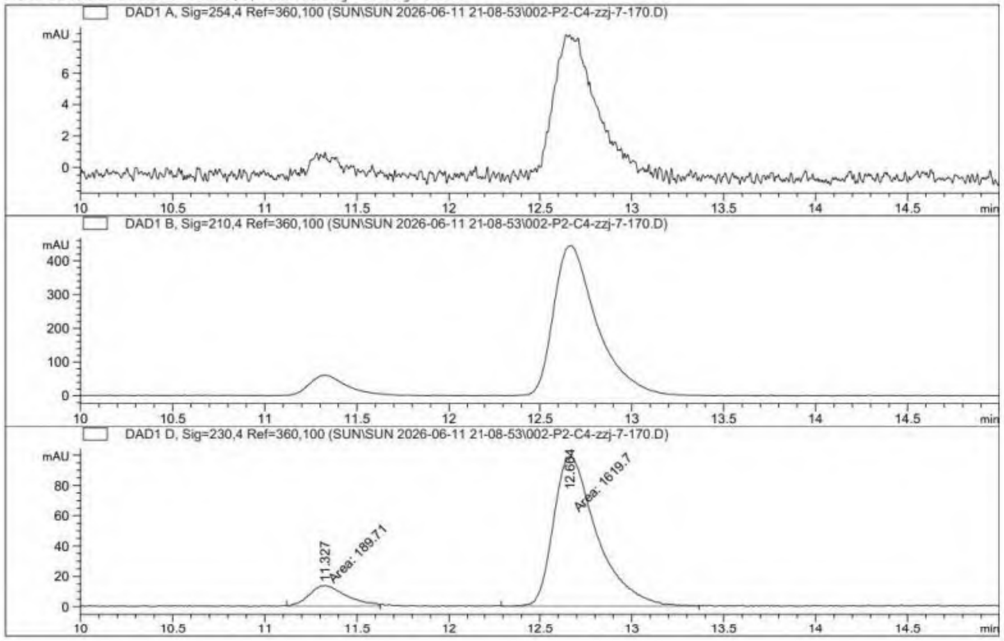

===

Area Percent Report

===

Sorted By : Signal
Multiplier : 1.0000
Dilution : 1.0000
Use Multiplier & Dilution Factor with ISTDs

Data File C:\Users\P...hemStation\1\Data\SUN\SUN 2026-06-11 21-08-53\002-P2-C4-zzj-7-170.D
Sample Name: zzj-7-170

Signal 1: DAD1 A, Sig=254,4 Ref=360,100

Signal 2: DAD1 B, Sig=210,4 Ref=360,100

Signal 3: DAD1 D, Sig=230,4 Ref=360,100

Peak #	RetTime [min]	Type	Width [min]	Area [mAU*s]	Height [mAU]	Area %
1	11.327	MM	0.2337	189.71002	13.53093	10.4847
2	12.664	MM	0.2729	1619.69543	98.90169	89.5153

Totals : 1809.40546 112.43262

===

*** End of Report ***

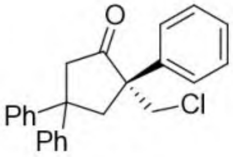

4k

```
=====================================================================
Acq. Operator   : SYSTEM                       Seq. Line :  13
Sample Operator : SYSTEM
Acq. Instrument : HPLC                          Location :  P1-B-03
Injection Date  : 6/1/2025 9:48:38 pm                Inj :  1
                                              Inj Volume : 2.000 µl
Different Inj Volume from Sample Entry!  Actual Inj Volume : 1.000 µl
Method          : C:\Users\Public\Documents\ChemStation\1\Data\SUN\SUN 2025-01-06 18-34-34\ID3-
                  05-30.M (Sequence Method)
Last changed    : 21/12/2024 9:29:52 pm by SYSTEM
Additional Info : Peak(s) manually integrated
```

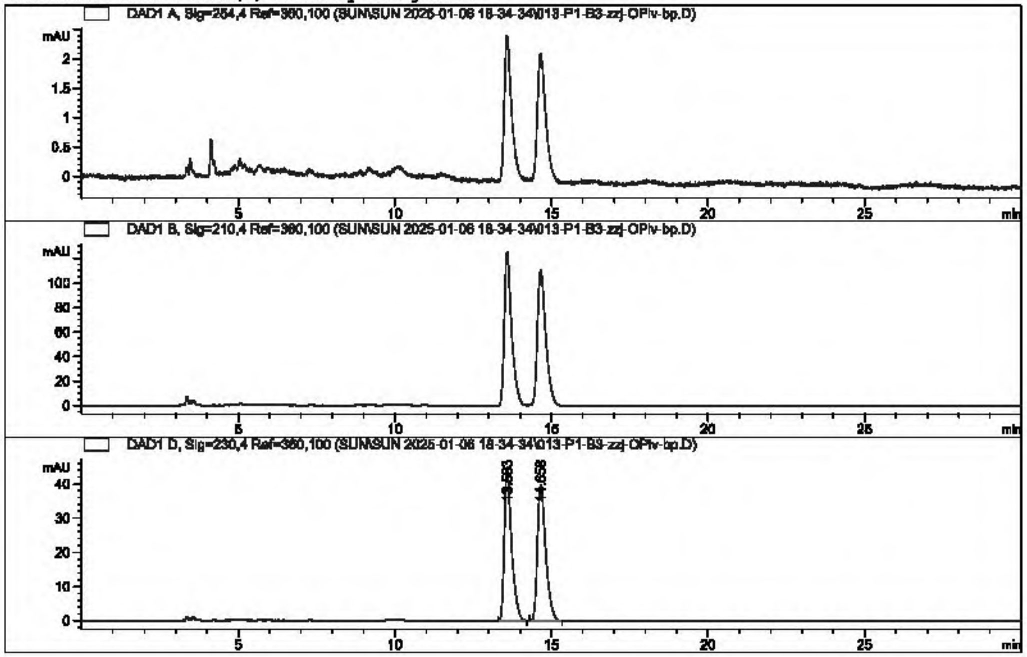


```
=====================================================================
                         Area Percent Report
=====================================================================

Sorted By             :      Signal
Multiplier            :      1.0000
Dilution              :      1.0000
Use Multiplier & Dilution Factor with ISTDs


Signal 1: DAD1 A, Sig=254,4 Ref=360,100
```


Signal 2: DAD1 B, Sig=210,4 Ref=360,100

Signal 3: DAD1 D, Sig=230,4 Ref=360,100

Peak #	RetTime [min]	Type	Width [min]	Area [mAU*s]	Height [mAU]	Area %
1	13.583	BB	0.2356	726.26581	44.92715	49.8217
2	14.658	BB	0.2625	731.46399	39.40814	50.1783

Totals : 1457.72980 84.33529

*** End of Report ***

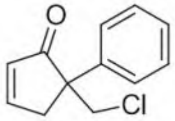

4l-rac

```
=====================================================================
Acq. Operator   : SYSTEM                        Seq. Line :  11
Sample Operator : SYSTEM
Acq. Instrument : HPLC                           Location :   P1-C-01
Injection Date  : 16/1/2025 1:33:37 pm                Inj :  1
                                               Inj Volume : 2.000 µl
Different Inj Volume from Sample Entry!  Actual Inj Volume : 1.000 µl
Method          : C:\Users\Public\Documents\ChemStation\1\Data\SUN\SUN 2025-01-16 10-32-15\ID3-
                  05-30.M (Sequence Method)
Last changed    : 21/12/2024 9:29:52 pm by SYSTEM
Additional Info : Peak(s) manually integrated
```

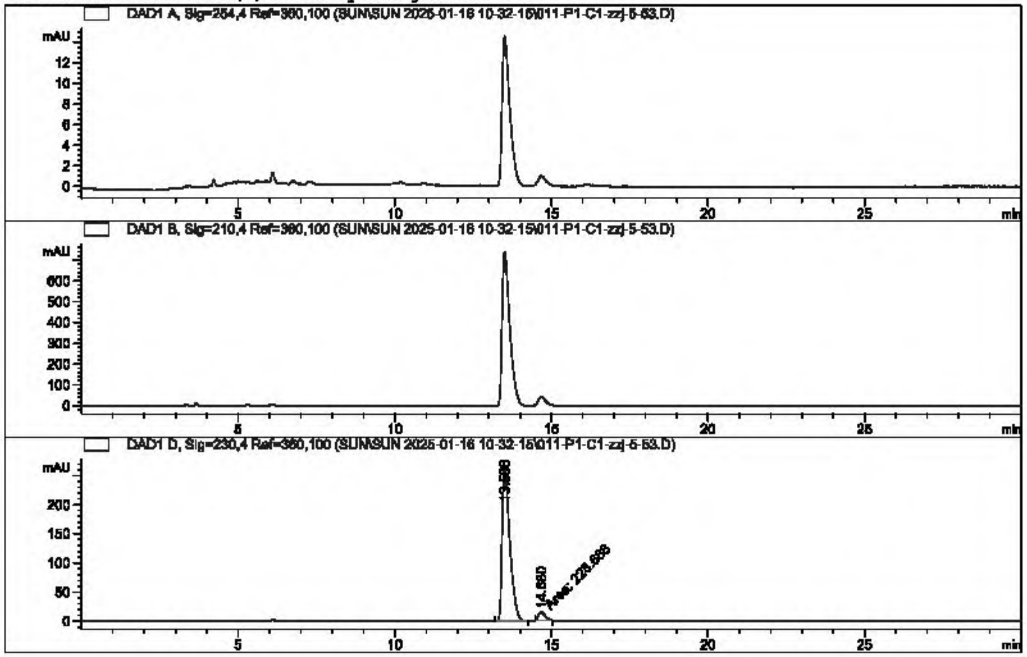


```
=====================================================================
                         Area Percent Report
=====================================================================

Sorted By             :      Signal
Multiplier            :      1.0000
Dilution              :      1.0000
Use Multiplier & Dilution Factor with ISTDs
```

Signal 1: DAD1 A, Sig=254,4 Ref=360,100

Signal 2: DAD1 B, Sig=210,4 Ref=360,100

Signal 3: DAD1 D, Sig=230,4 Ref=360,100

Peak #	RetTime [min]	Type	Width [min]	Area [mAU*s]	Height [mAU]	Area %
1	13.508	BB	0.2537	4506.56787	263.97635	95.1705
2	14.680	MM	0.2681	228.68817	14.21768	4.8295

Totals : 4735.25604 278.19403

*** End of Report ***

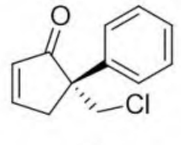

4l

Data File C:\Users\P...ation\1\Data\SUN\SUN 2025-03-07 22-40-33\002-P1-E1-zzj-5-71-4-ibn.D
Sample Name: zzj-5-71-4-ibn

===

```
Acq. Operator   : SYSTEM                        Seq. Line :   2
Sample Operator : SYSTEM
Acq. Instrument : HPLC                           Location :   P1-E-01
Injection Date  : 7/3/2025 10:53:24 pm                Inj :   1
                                              Inj Volume : 2.000 µl
Different Inj Volume from Sample Entry!  Actual Inj Volume : 5.000 µl
Acq. Method     : C:\Users\Public\Documents\ChemStation\1\Data\SUN\SUN 2025-03-07 22-40-33\IBN3-
                  05-30.M
Last changed    : 29/8/2022 6:09:41 pm by SYSTEM
Analysis Method : C:\Users\Public\Documents\ChemStation\1\Data\SUN\SUN 2025-03-07 22-40-33\IBN3-
                  05-30.M (Sequence Method)
Last changed    : 13/12/2025 3:34:22 pm by SYSTEM
                  (modified after loading)
Additional Info : Peak(s) manually integrated
```

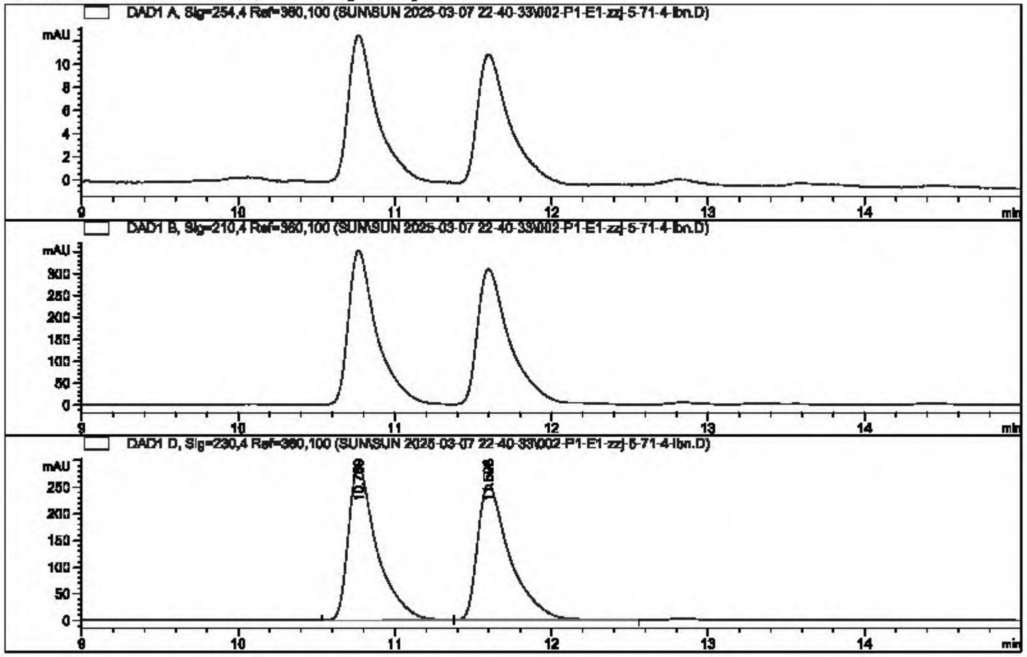

===

Area Percent Report

===

```
Sorted By             :      Signal
Multiplier            :      1.0000
Dilution              :      1.0000
Use Multiplier & Dilution Factor with ISTDs
```


Data File C:\Users\P...ation\1\Data\SUN\SUN 2025-03-07 22-40-33\002-P1-E1-zzj-5-71-4-ibn.D
Sample Name: zzj-5-71-4-ibn

Signal 1: DAD1 A, Sig=254,4 Ref=360,100

Signal 2: DAD1 B, Sig=210,4 Ref=360,100

Signal 3: DAD1 D, Sig=230,4 Ref=360,100

Peak #	RetTime [min]	Type	Width [min]	Area [mAU*s]	Height [mAU]	Area %
1	10.769	BB	0.1851	3630.47998	287.89362	50.0096
2	11.598	BB	0.2105	3629.09302	253.46677	49.9904

Totals : 7259.57300 541.36038

===

*** End of Report ***

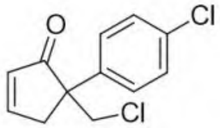

4m-rac

Data File C:\Users\P...Station\1\Data\SUN\SUN 2025-01-18 21-06-36\006-P1-E1-zzj-5-57-ibn.D
Sample Name: zzj-5-57-ibn

```
=====================================================================
Acq. Operator   : SYSTEM                        Seq. Line :    6
Sample Operator : SYSTEM
Acq. Instrument : HPLC                           Location :   P1-E-01
Injection Date  : 18/1/2025 10:44:06 pm               Inj :    1
                                               Inj Volume : 2.000 µl
Different Inj Volume from Sample Entry!  Actual Inj Volume : 1.000 µl
Acq. Method     : C:\Users\Public\Documents\ChemStation\1\Data\SUN\SUN 2025-01-18 21-06-36\IBN3-
                  05-30.M
Last changed    : 29/8/2022 6:09:41 pm by SYSTEM
Analysis Method : C:\Users\Public\Documents\ChemStation\1\Data\SUN\SUN 2025-01-18 21-06-36\IBN3-
                  05-30.M (Sequence Method)
Last changed    : 13/12/2025 3:35:31 pm by SYSTEM
                  (modified after loading)
Additional Info : Peak(s) manually integrated
```

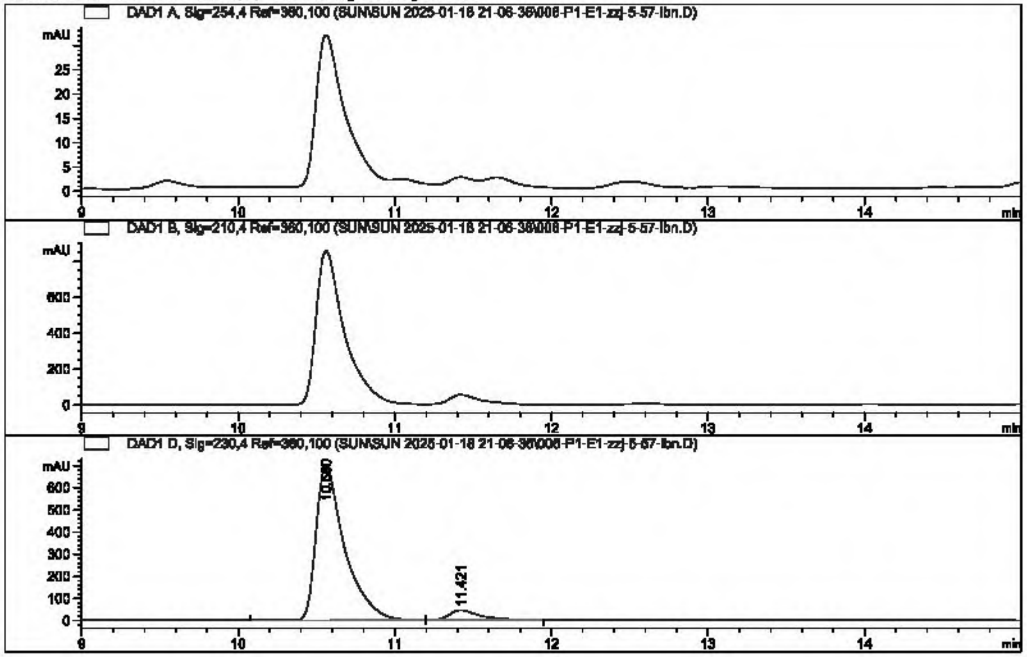


```
=====================================================================
                         Area Percent Report
=====================================================================

Sorted By             :      Signal
Multiplier            :      1.0000
Dilution              :      1.0000
Use Multiplier & Dilution Factor with ISTDs
```


Data File C:\Users\P...Station\1\Data\SUN\SUN 2025-01-18 21-06-36\006-P1-E1-zzj-5-57-ibn.D
Sample Name: zzj-5-57-ibn

Signal 1: DAD1 A, Sig=254,4 Ref=360,100

Signal 2: DAD1 B, Sig=210,4 Ref=360,100

Signal 3: DAD1 D, Sig=230,4 Ref=360,100

Peak #	RetTime [min]	Type	Width [min]	Area [mAU*s]	Height [mAU]	Area %
1	10.560	VB R	0.1873	8846.74707	694.73401	93.6022
2	11.421	BB	0.1986	604.68567	45.04316	6.3978

Totals : 9451.43274 739.77717

*** End of Report ***

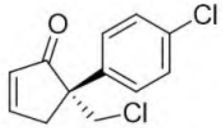

4m

Data File C:\Users\P...\ChemStation\1\Data\SUN\SUN 2024-07-05 09-18-46\OnlineEdited--012.D
Sample Name: zzj-4-49-id

Acq. Operator : SYSTEM   Seq. Line : 12
Sample Operator : SYSTEM
Acq. Instrument : HPLC   Location : P1-B-01
Injection Date : 5/7/2024 12:56:31 pm   Inj : 1
Inj Volume : 2.000 µl
Different Inj Volume from Sample Entry! Actual Inj Volume : 10.000 µl
Acq. Method : C:\Users\Public\Documents\ChemStation\1\Data\SUN\SUN 2024-07-05 09-18-46\ID3-20-30.M
Last changed : 21/9/2022 10:08:04 am by SYSTEM
Analysis Method : C:\Users\Public\Documents\ChemStation\1\Data\SUN\SUN 2024-07-05 09-18-46\ID3-20-30.M (Sequence Method)
Last changed : 5/7/2024 7:40:36 pm by SYSTEM
Additional Info : Peak(s) manually integrated

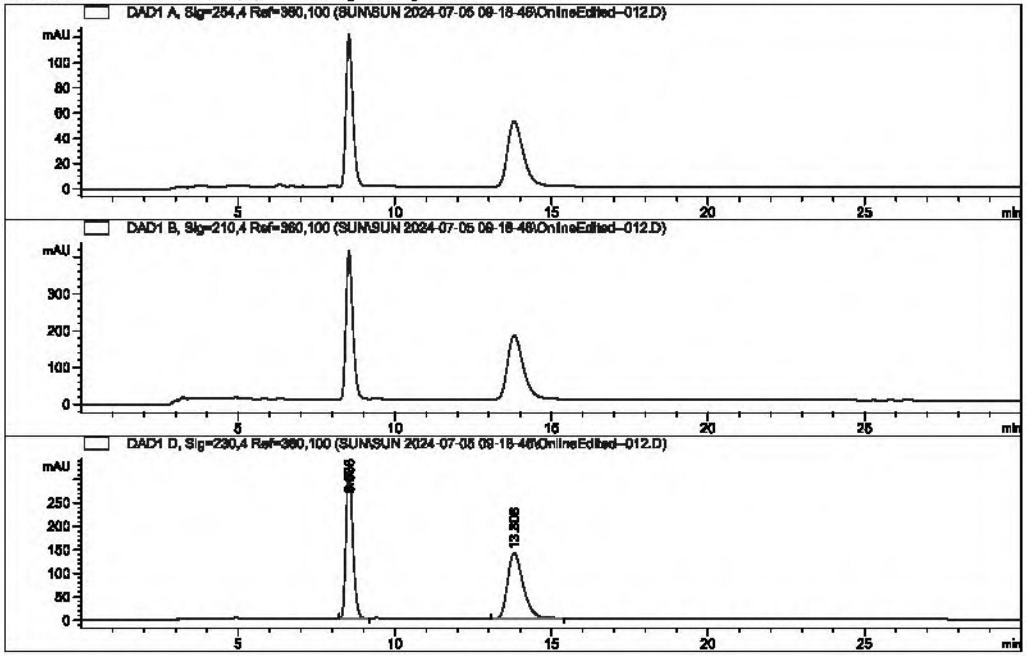

Area Percent Report

Sorted By : Signal
Multiplier : 1.0000
Dilution : 1.0000
Use Multiplier & Dilution Factor with ISTDs

Data File C:\Users\P...\ChemStation\1\Data\SUN\SUN 2024-07-05 09-18-46\OnlineEdited--012.D
Sample Name: zzj-4-49-id

Signal 1: DAD1 A, Sig=254,4 Ref=360,100

Signal 2: DAD1 B, Sig=210,4 Ref=360,100

Signal 3: DAD1 D, Sig=230,4 Ref=360,100

Peak #	RetTime [min]	Type	Width [min]	Area [mAU*s]	Height [mAU]	Area %
1	8.538	BB	0.2320	4879.54639	324.40683	50.1208
2	13.808	BB	0.5248	4856.03418	139.44635	49.8792
Totals :				9735.58057	463.85318	

*** End of Report ***

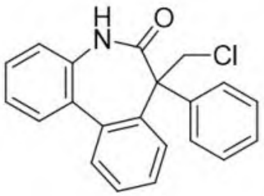

5-rac

Acq. Operator : SYSTEM Seq. Line : 31
Sample Operator : SYSTEM
Acq. Instrument : HPLC Location : P1-B-01
Injection Date : 5/7/2024 7:13:45 pm Inj : 1
Inj Volume : 2.000 µl
Different Inj Volume from Sample Entry! Actual Inj Volume : 10.000 µl
Acq. Method : C:\Users\Public\Documents\ChemStation\1\Data\SUN\SUN 2024-07-05 09-18-46\ID3-20-30.M
Last changed : 5/7/2024 7:40:29 pm by SYSTEM
(modified after loading)
Analysis Method : C:\Users\Public\Documents\ChemStation\1\Data\SUN\SUN 2024-07-05 09-18-46\ID3-20-30.M (Sequence Method)
Last changed : 5/7/2024 7:40:36 pm by SYSTEM
Additional Info : Peak(s) manually integrated

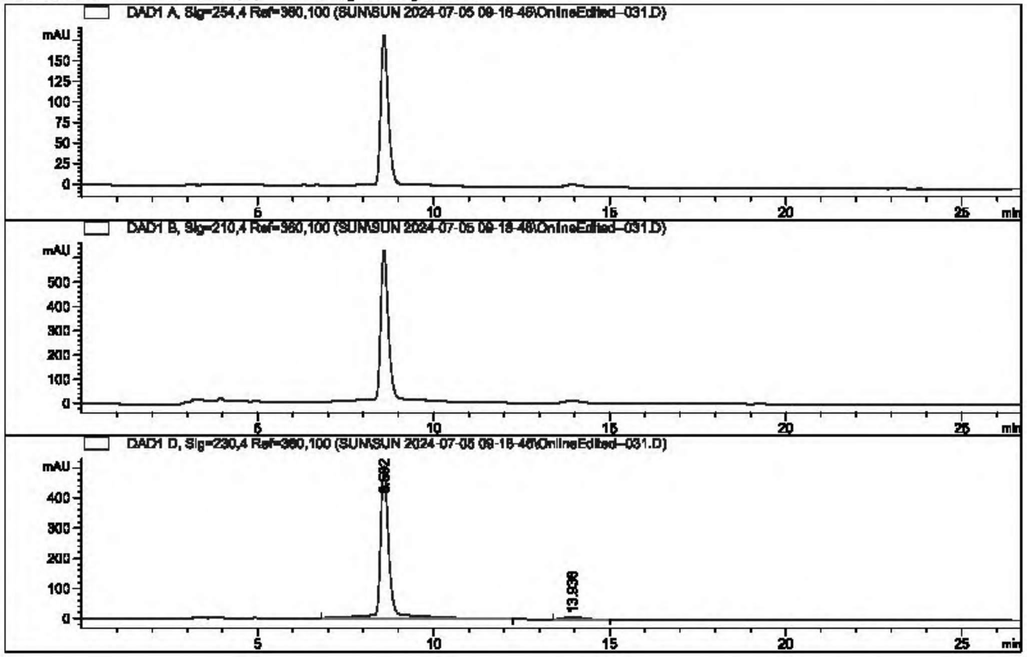

Area Percent Report

Sorted By : Signal
Multiplier : 1.0000
Dilution : 1.0000
Use Multiplier & Dilution Factor with ISTDs

Data File C:\Users\P...\ChemStation\1\Data\SUN\SUN 2024-07-05 09-18-46\OnlineEdited--031.D
Sample Name: zzj-4-33

Signal 1: DAD1 A, Sig=254,4 Ref=360,100

Signal 2: DAD1 B, Sig=210,4 Ref=360,100

Signal 3: DAD1 D, Sig=230,4 Ref=360,100

Peak #	RetTime [min]	Type	Width [min]	Area [mAU*s]	Height [mAU]	Area %
1	8.592	BB	0.2616	9009.91797	505.01044	96.8046
2	13.936	BB	0.3977	297.40634	8.79043	3.1954

Totals : 9307.32431 513.80087

*** End of Report ***

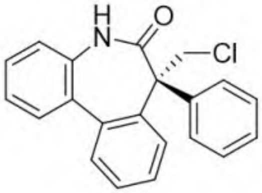

5

Data File C:\Users\P...tation\1\Data\SUN\SUN 2024-11-07 10-24-19\059-P1-A3-zzj-4-169-rac.D
Sample Name: zzj-4-169-rac

===

Acq. Operator : SYSTEM Seq. Line : 59
Sample Operator : SYSTEM
Acq. Instrument : HPLC Location : P1-A-03
Injection Date : 8/11/2024 3:16:32 am Inj : 1
Inj Volume : 2.000 µl
Method : C:\Users\Public\Documents\ChemStation\1\Data\SUN\SUN 2024-11-07 10-24-19\IBN3-01-40.M (Sequence Method)
Last changed : 25/11/2023 2:29:44 pm by SYSTEM
Additional Info : Peak(s) manually integrated

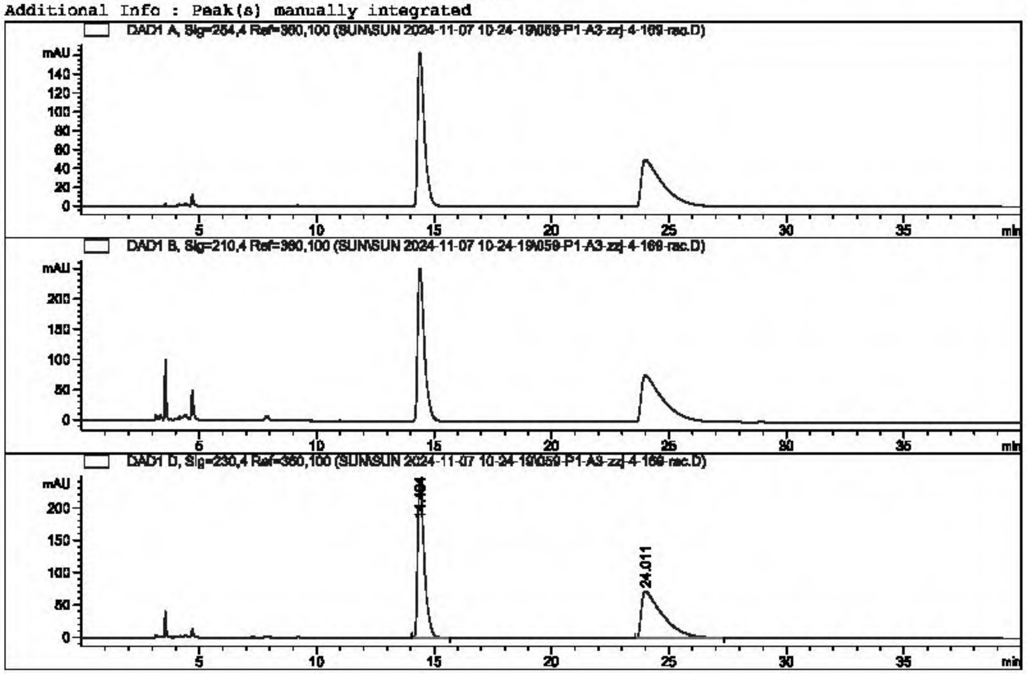

===

Area Percent Report

===

Sorted By : Signal
Multiplier : 1.0000
Dilution : 1.0000
Use Multiplier & Dilution Factor with ISTDs

Signal 1: DAD1 A, Sig=254,4 Ref=360,100

Data File C:\Users\P...tation\1\Data\SUN\SUN 2024-11-07 10-24-19\059-P1-A3-zzj-4-169-rac.D
Sample Name: zzj-4-169-rac

Signal 2: DAD1 B, Sig=210,4 Ref=360,100

Signal 3: DAD1 D, Sig=230,4 Ref=360,100

Peak #	RetTime [min]	Type	Width [min]	Area [mAU*s]	Height [mAU]	Area %
1	14.404	BB	0.2838	4567.68262	237.60219	50.1351
2	24.011	BB	0.8056	4543.07373	72.24596	49.8649
Totals :				9110.75635	309.84814	

*** End of Report ***

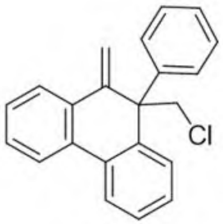

6-rac

Acq. Operator : SYSTEM Seq. Line : 57
Sample Operator : SYSTEM
Acq. Instrument : HPLC Location : P2-D-01
Injection Date : 27/10/2024 3:44:01 am Inj : 1
Inj Volume : 2.000 µl
Method : C:\Users\Public\Documents\ChemStation\1\Data\SUN\SUN 2024-10-26 14-32-23\IBN3-01-40.M (Sequence Method)
Last changed : 25/11/2023 2:29:44 pm by SYSTEM
Additional Info : Peak(s) manually integrated

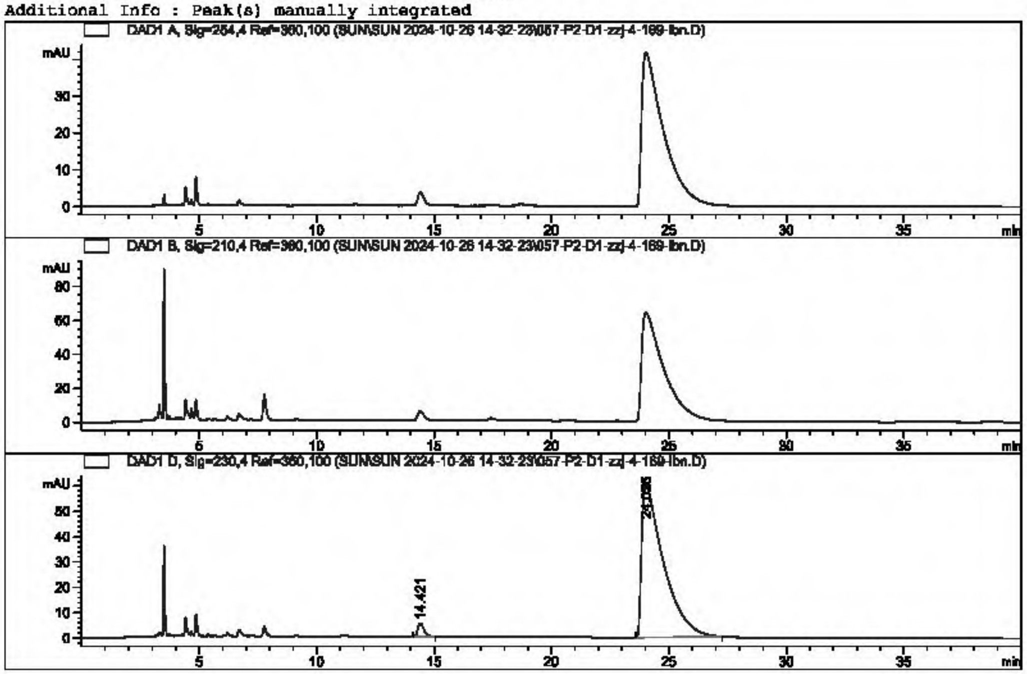

Area Percent Report

Sorted By : Signal
Multiplier : 1.0000
Dilution : 1.0000
Use Multiplier & Dilution Factor with ISTDs

Signal 1: DAD1 A, Sig=254,4 Ref=360,100

Data File C:\Users\P...tation\1\Data\SUN\SUN 2024-10-26 14-32-23\057-P2-D1-zzj-4-169-ibn.D
Sample Name: zzj-4-169-ibn

Signal 2: DAD1 B, Sig=210,4 Ref=360,100

Signal 3: DAD1 D, Sig=230,4 Ref=360,100

Peak #	RetTime [min]	Type	Width [min]	Area [mAU*s]	Height [mAU]	Area %
1	14.421	BB	0.2299	95.34566	5.27373	2.3955
2	24.025	BB	0.8248	3884.86450	60.37077	97.6045

Totals : 3980.21016 65.64450

*** End of Report ***

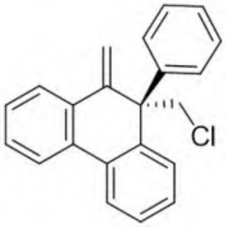

6

Acq. Operator : SYSTEM Seq. Line : 14
Sample Operator : SYSTEM
Acq. Instrument : HPLC Location : P1-E-02
Injection Date : 5/3/2025 2:07:20 pm Inj : 1
Inj Volume : 2.000 µl
Different Inj Volume from Sample Entry! Actual Inj Volume : 20.000 µl
Acq. Method : C:\Users\Public\Documents\ChemStation\1\Data\SUN\SUN 2025-03-05 09-50-36\ID3-05-20.M
Last changed : 18/8/2022 3:59:32 pm by SYSTEM
Analysis Method : C:\Users\Public\Documents\ChemStation\1\Data\SUN\SUN 2025-03-05 09-50-36\ID3-05-20.M (Sequence Method)
Last changed : 13/12/2025 3:44:31 pm by SYSTEM
(modified after loading)
Additional Info : Peak(s) manually integrated

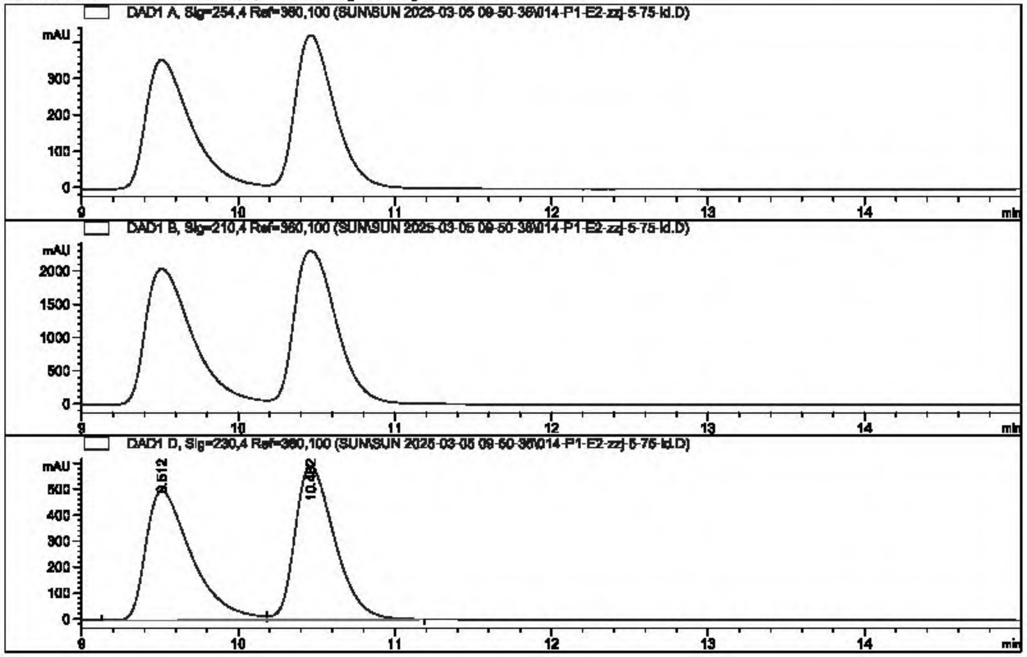

Area Percent Report

Sorted By : Signal
Multiplier : 1.0000
Dilution : 1.0000
Use Multiplier & Dilution Factor with ISTDs

Data File C:\Users\P...mStation\1\Data\SUN\SUN 2025-03-05 09-50-36\014-P1-E2-zzj-5-75-id.D
Sample Name: zzj-5-75-id

Signal 1: DAD1 A, Sig=254,4 Ref=360,100

Signal 2: DAD1 B, Sig=210,4 Ref=360,100

Signal 3: DAD1 D, Sig=230,4 Ref=360,100

Peak #	RetTime [min]	Type	Width [min]	Area [mAU*s]	Height [mAU]	Area %
1	9.512	BV	0.3025	1.01024e4	498.32443	49.3415
2	10.462	VB	0.2678	1.03720e4	590.81128	50.6585
Totals :				2.04744e4	1089.13571	

*** End of Report ***

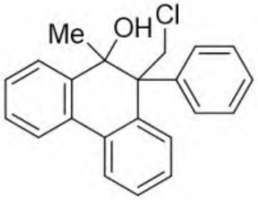

7-rac

Data File C:\Users\P...ChemStation\1\Data\SUN\SUN 2026-05-11 16-09-37\006-P2-C1-zzj-MeLi.D
Sample Name: zzj-MeLi

Acq. Operator : SYSTEM Seq. Line : 6
Sample Operator : SYSTEM
Acq. Instrument : HPLC Location : P2-C-01
Injection Date : 11/5/2026 5:37:46 pm Inj : 1
Inj Volume : 2.000 µl
Different Inj Volume from Sample Entry! Actual Inj Volume : 1.000 µl
Acq. Method : C:\Users\Public\Documents\ChemStation\1\Data\SUN\SUN 2026-05-11 16-09-37\ID3-05-20.M
Last changed : 18/8/2022 3:59:32 pm by SYSTEM
Analysis Method : C:\Users\Public\Documents\ChemStation\1\Data\SUN\SUN 2026-05-11 16-09-37\ID3-05-20.M (Sequence Method)
Last changed : 11/5/2026 6:25:41 pm by SYSTEM
(modified after loading)
Additional Info : Peak(s) manually integrated

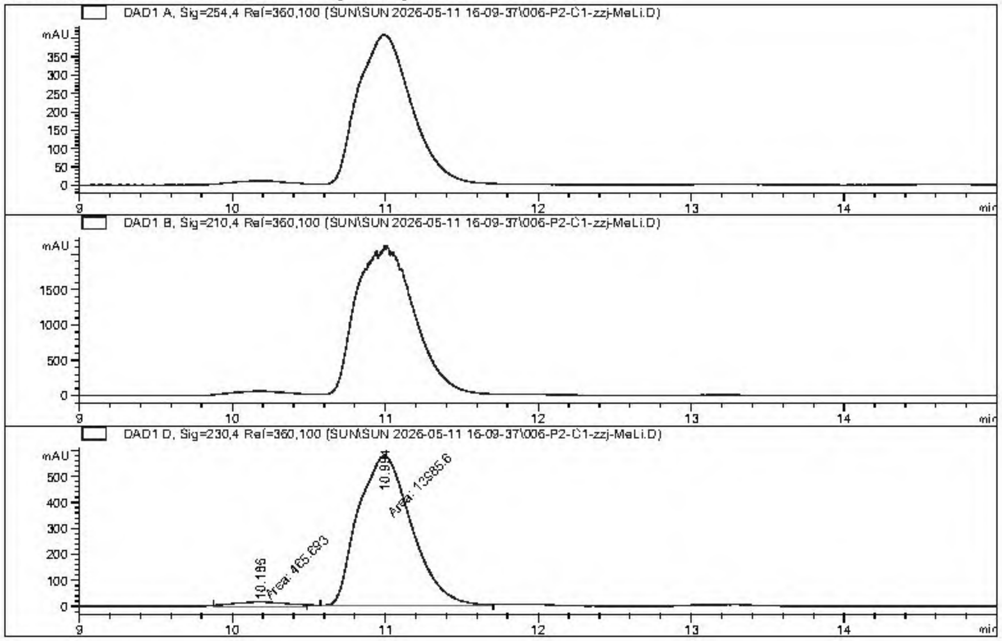

Area Percent Report

Sorted By : Signal
Multiplier : 1.0000
Dilution : 1.0000
Use Multiplier & Dilution Factor with ISTDs

Data File C:\Users\P...ChemStation\1\Data\SUN\SUN 2026-05-11 16-09-37\006-P2-C1-zzj-MeLi.D
Sample Name: zzj-MeLi

Signal 1: DAD1 A, Sig=254,4 Ref=360,100

Signal 2: DAD1 B, Sig=210,4 Ref=360,100

Signal 3: DAD1 D, Sig=230,4 Ref=360,100

Peak #	RetTime [min]	Type	Width [min]	Area [mAU*s]	Height [mAU]	Area %
1	10.186	MM	0.4354	465.69266	17.82556	3.2225
2	10.994	MM	0.4045	1.39856e4	576.22754	96.7775

Totals : 1.44513e4 594.05310

*** End of Report ***

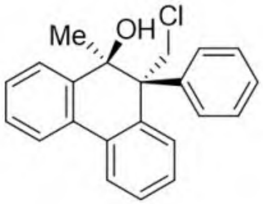

7

===

Acq. Operator : SYSTEM   Seq. Line : 4
Sample Operator : SYSTEM
Acq. Instrument : HPLC   Location : P1-B-02
Injection Date : 6/7/2024 9:54:57 am   Inj : 1
Inj Volume : 2.000 µl
Different Inj Volume from Sample Entry! Actual Inj Volume : 1.000 µl
Method : C:\Users\Public\Documents\ChemStation\1\Data\SUN\SUN 2024-07-06 09-09-49\AD3-05-30.M (Sequence Method)
Last changed : 3/9/2022 11:16:06 pm by SYSTEM
Additional Info : Peak(s) manually integrated

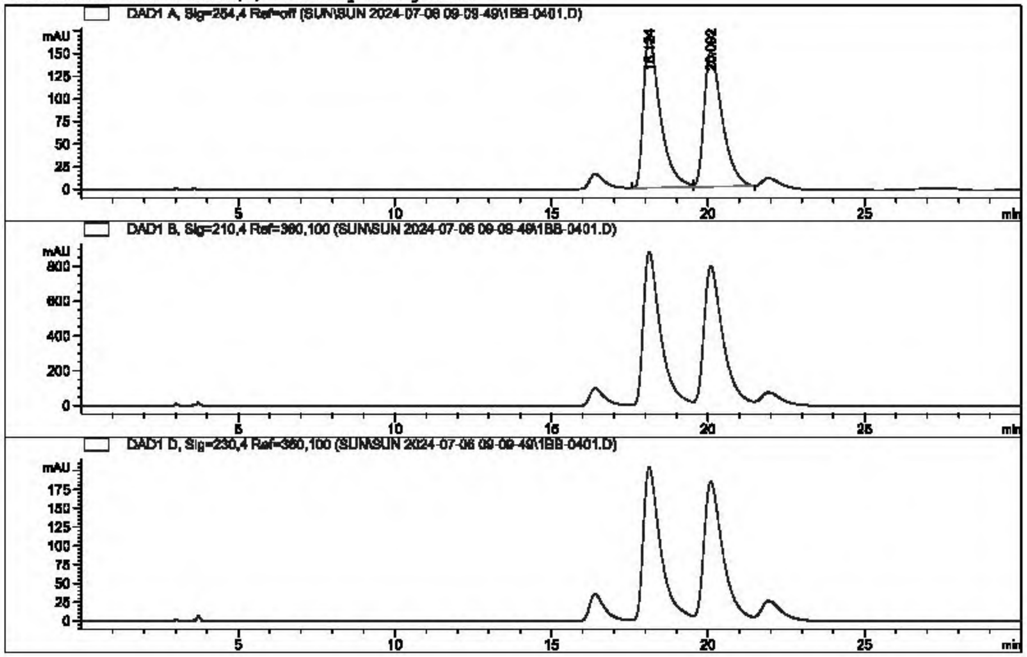

===

Area Percent Report

===

Sorted By : Signal
Multiplier : 1.0000
Dilution : 1.0000
Use Multiplier & Dilution Factor with ISTDs

Signal 1: DAD1 A, Sig=254,4 Ref=off

Peak #	RetTime [min]	Type	Width [min]	Area [mAU*s]	Height [mAU]	Area %
1	18.124	BV	0.5378	6236.80420	167.16403	51.1159
2	20.092	VB	0.5713	5964.49268	149.55174	48.8841

Data File C:\Users\P...Documents\ChemStation\1\Data\SUN\SUN 2024-07-06 09-09-49\1BB-0401.D
Sample Name: zzj-4-52-ad05

Peak #	RetTime [min]	Type	Width [min]	Area [mAU*s]	Height [mAU]	Area %
Totals :				1.22013e4	316.71577	

Signal 2: DAD1 B, Sig=210,4 Ref=360,100

Signal 3: DAD1 D, Sig=230,4 Ref=360,100

===

*** End of Report ***

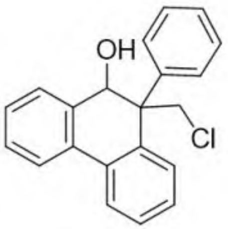

8-rac

Data File C:\Users\P...\ChemStation\1\Data\SUN\SUN 2024-08-12 11-22-25\OnlineEdited--019.D
Sample Name: zzj-4-85

Acq. Operator : SYSTEM Seq. Line : 19
Sample Operator : SYSTEM
Acq. Instrument : HPLC Location : P1-B-04
Injection Date : 12/8/2024 4:32:38 pm Inj : 1
Inj Volume : 2.000 µl
Method : C:\Users\Public\Documents\ChemStation\1\Data\SUN\SUN 2024-08-12 11-22-25\AD3-05-30.M (Sequence Method)
Last changed : 3/9/2022 11:16:06 pm by SYSTEM
Additional Info : Peak(s) manually integrated

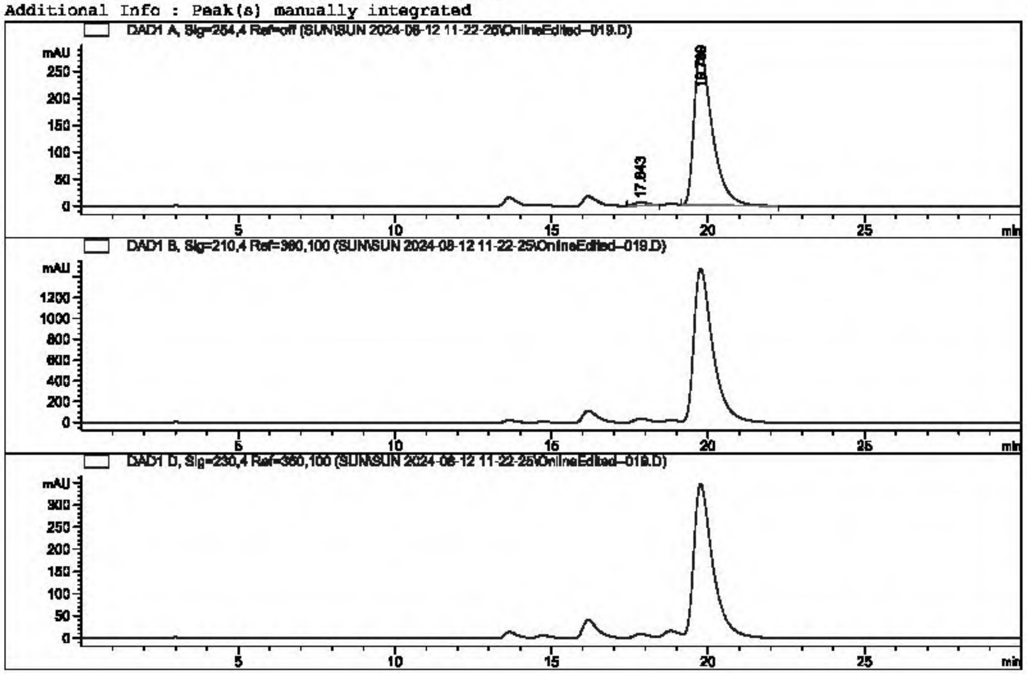

Area Percent Report

Sorted By : Signal
Multiplier : 1.0000
Dilution : 1.0000
Use Multiplier & Dilution Factor with ISTDs

Signal 1: DAD1 A, Sig=254,4 Ref=off

Peak #	RetTime [min]	Type	Width [min]	Area [mAU*s]	Height [mAU]	Area %
1	17.843	BB	0.3421	180.53667	6.20189	1.5487
2	19.769	BB	0.5974	1.14770e4	281.95685	98.4513

Data File C:\Users\P...\ChemStation\1\Data\SUN\SUN 2024-08-12 11-22-25\OnlineEdited--019.D
Sample Name: zzj-4-85

Totals : 1.16575e4 288.15874

Signal 2: DAD1 B, Sig=210,4 Ref=360,100

Signal 3: DAD1 D, Sig=230,4 Ref=360,100

*** End of Report ***

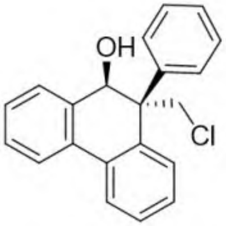

8

```
=====================================================================
Acq. Operator   : SYSTEM                        Seq. Line :    2
Sample Operator : SYSTEM
Acq. Instrument : HPLC                           Location :   P1-A-01
Injection Date  : 25/10/2025 11:06:51 am              Inj :    1
                                               Inj Volume : 2.000 µl
Different Inj Volume from Sample Entry!  Actual Inj Volume : 20.000 µl
Acq. Method     : C:\Users\Public\Documents\ChemStation\1\Data\SUN\SUN 2025-10-25 10-53-28\ID3-
                  05-30.M
Last changed    : 21/12/2024 9:29:52 pm by SYSTEM
Analysis Method : C:\Users\Public\Documents\ChemStation\1\Data\SUN\SUN 2025-10-25 10-53-28\ID3-
                  05-30.M (Sequence Method)
Last changed    : 7/11/2025 8:05:34 pm by SYSTEM
                  (modified after loading)
Additional Info : Peak(s) manually integrated
```

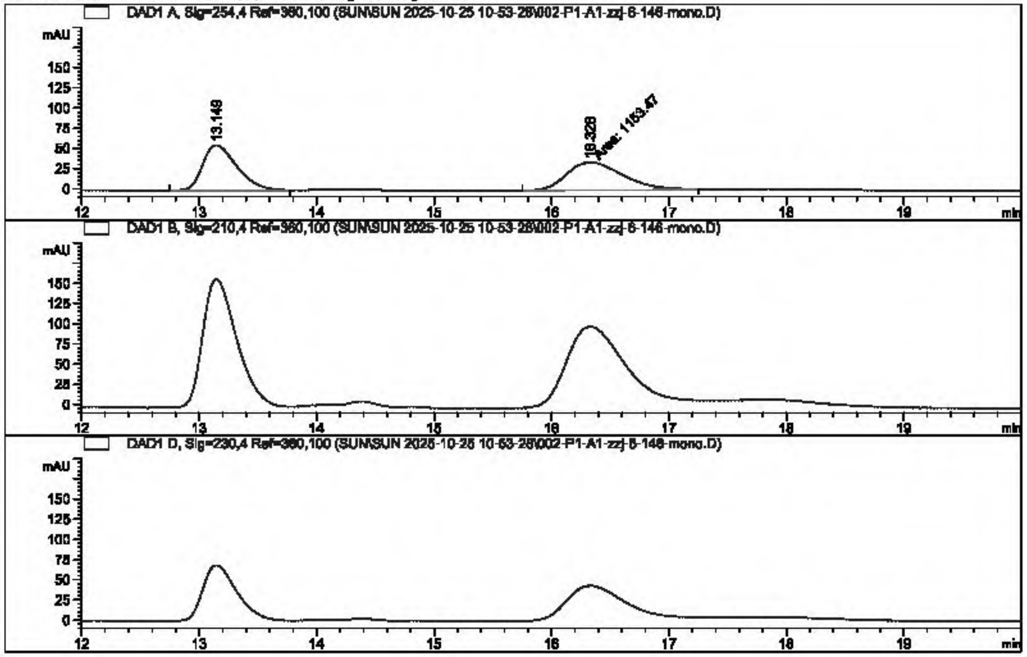

===

Area Percent Report

===

```
Sorted By          :      Signal
Multiplier         :      1.0000
Dilution           :      1.0000
Use Multiplier & Dilution Factor with ISTDs
```


Data File C:\Users\P...ation\1\Data\SUN\SUN 2025-10-25 10-53-28\002-P1-A1-zzj-6-146-mono.D
Sample Name: zzj-6-146-mono

Signal 1: DAD1 A, Sig=254,4 Ref=360,100

Peak #	RetTime [min]	Type	Width [min]	Area [mAU*s]	Height [mAU]	Area %
1	13.149	VB R	0.2378	1123.30151	55.82436	49.3374
2	16.328	MM	0.5581	1153.47461	34.44892	50.6626
Totals :				2276.77612	90.27328	

Signal 2: DAD1 B, Sig=210,4 Ref=360,100

Signal 3: DAD1 D, Sig=230,4 Ref=360,100

*** End of Report ***

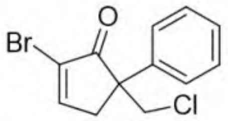

9-rac

Data File C:\Users\P...on\1\Data\SUN\SUN 2025-09-23 11-10-25\002-P1-A1-zzj-6-93-major-id.D
Sample Name: zzj-6-93-major-id

```
=====================================================================
Acq. Operator   : SYSTEM                        Seq. Line :    2
Sample Operator : SYSTEM
Acq. Instrument : HPLC                           Location :   P1-A-01
Injection Date  : 23/9/2025 11:23:54 am               Inj :   1
                                               Inj Volume : 2.000 µl
Different Inj Volume from Sample Entry!  Actual Inj Volume : 20.000 µl
Acq. Method     : C:\Users\Public\Documents\ChemStation\1\Data\SUN\SUN 2025-09-23 11-10-25\ID3-
                  05-30.M
Last changed    : 21/12/2024 9:29:52 pm by SYSTEM
Analysis Method : C:\Users\Public\Documents\ChemStation\1\Data\SUN\SUN 2025-09-23 11-10-25\ID3-
                  05-30.M (Sequence Method)
Last changed    : 13/12/2025 4:22:29 pm by SYSTEM
                  (modified after loading)
Additional Info : Peak(s) manually integrated
```

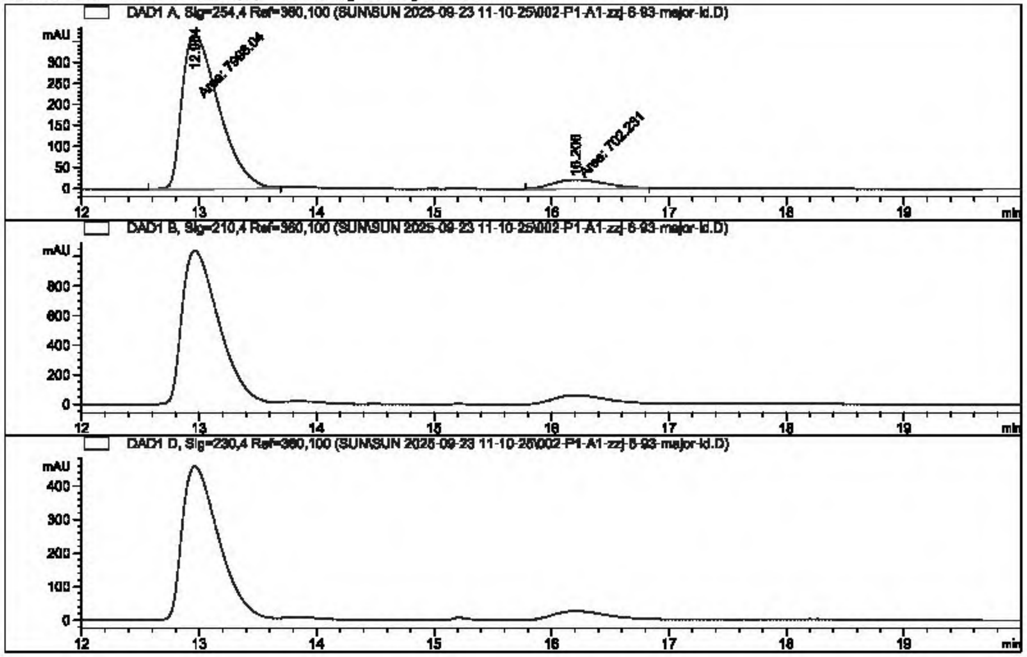


```
=====================================================================
                         Area Percent Report
=====================================================================

Sorted By             :      Signal
Multiplier            :      1.0000
Dilution              :      1.0000
Use Multiplier & Dilution Factor with ISTDs
```


Data File C:\Users\P...on\1\Data\SUN\SUN 2025-09-23 11-10-25\002-P1-A1-zzj-6-93-major-id.D
Sample Name: zzj-6-93-major-id

Signal 1: DAD1 A, Sig=254,4 Ref=360,100

Peak #	RetTime [min]	Type	Width [min]	Area [mAU*s]	Height [mAU]	Area %
1	12.964	MF	0.3633	7998.03564	366.87042	91.9286
2	16.206	MM	0.5288	702.23096	22.13129	8.0714

Totals : 8700.26660 389.00171

Signal 2: DAD1 B, Sig=210,4 Ref=360,100

Signal 3: DAD1 D, Sig=230,4 Ref=360,100

*** End of Report ***

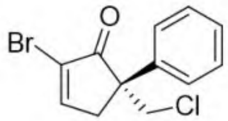

9

```
=====================================================================
Acq. Operator   : SYSTEM                        Seq. Line :    2
Sample Operator : SYSTEM
Acq. Instrument : HPLC                           Location :   P1-A-02
Injection Date  : 24/10/2025 11:07:23 am              Inj :    1
                                               Inj Volume : 2.000 µl
Different Inj Volume from Sample Entry!  Actual Inj Volume : 15.000 µl
Acq. Method     : C:\Users\Public\Documents\ChemStation\1\Data\SUN\SUN 2025-10-24 10-54-00\AS3-
                  05-30.M
Last changed    : 15/8/2022 10:23:13 pm by SYSTEM
Analysis Method : C:\Users\Public\Documents\ChemStation\1\Data\SUN\SUN 2025-10-24 10-54-00\AS3-
                  05-30.M (Sequence Method)
Last changed    : 7/11/2025 8:08:41 pm by SYSTEM
                  (modified after loading)
Additional Info : Peak(s) manually integrated
```

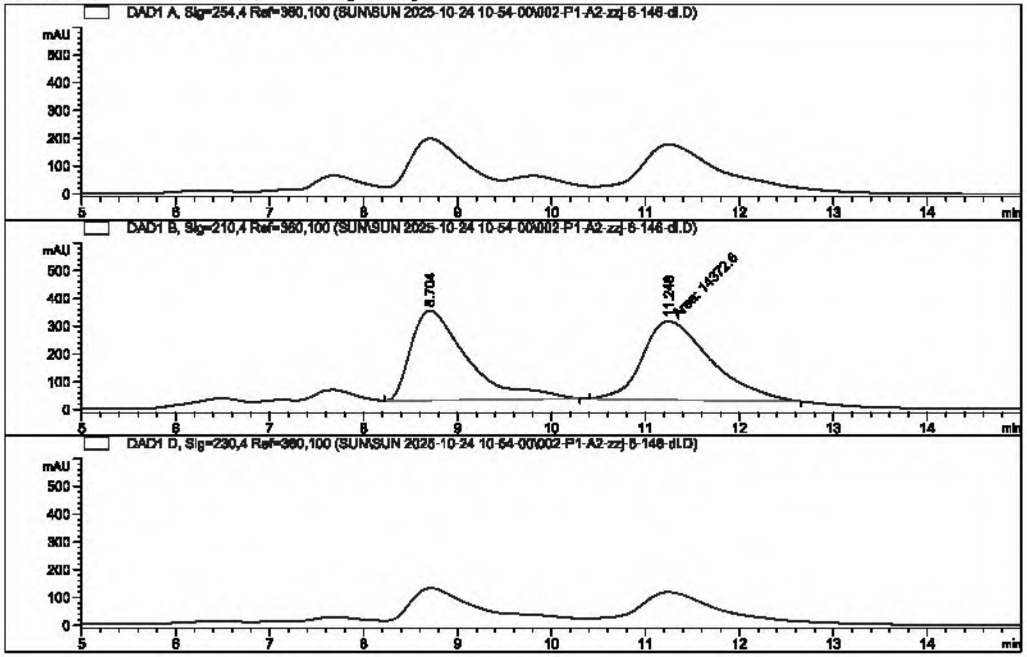


```
=====================================================================
                         Area Percent Report
=====================================================================

Sorted By             :      Signal
Multiplier            :      1.0000
Dilution              :      1.0000
Use Multiplier & Dilution Factor with ISTDs
```


Data File C:\Users\P...Station\1\Data\SUN\SUN 2025-10-24 10-54-00\002-P1-A2-zzj-6-146-di.D
Sample Name: zzj-6-146-di

Signal 1: DAD1 A, Sig=254,4 Ref=360,100

Signal 2: DAD1 B, Sig=210,4 Ref=360,100

Peak #	RetTime [min]	Type	Width [min]	Area [mAU*s]	Height [mAU]	Area %
1	8.704	BV R	0.4824	1.33618e4	322.04022	48.1777
2	11.248	MM	0.8447	1.43726e4	283.57394	51.8223
Totals :				2.77345e4	605.61417	

Signal 3: DAD1 D, Sig=230,4 Ref=360,100

*** End of Report ***

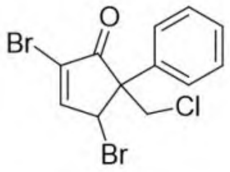

10-rac

===

Acq. Operator : SYSTEM Seq. Line : 22
Sample Operator : SYSTEM
Acq. Instrument : HPLC Location : P1-A-02
Injection Date : 23/9/2025 4:14:23 pm Inj : 1
Inj Volume : 2.000 µl
Different Inj Volume from Sample Entry! Actual Inj Volume : 20.000 µl
Acq. Method : C:\Users\Public\Documents\ChemStation\1\Data\SUN\SUN 2025-09-23 11-10-25\AS3-05-30.M
Last changed : 15/8/2022 10:23:13 pm by SYSTEM
Analysis Method : C:\Users\Public\Documents\ChemStation\1\Data\SUN\SUN 2025-09-23 11-10-25\AS3-05-30.M (Sequence Method)
Last changed : 13/12/2025 4:20:51 pm by SYSTEM
(modified after loading)
Additional Info : Peak(s) manually integrated

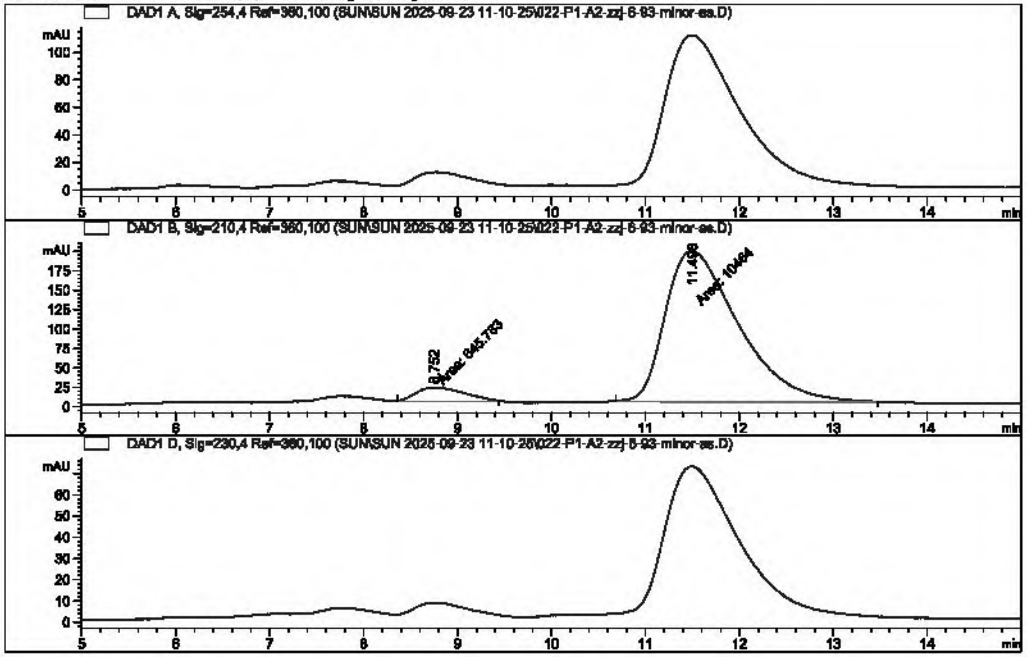

===

Area Percent Report

===

Sorted By : Signal
Multiplier : 1.0000
Dilution : 1.0000
Use Multiplier & Dilution Factor with ISTDs

Data File C:\Users\P...on\1\Data\SUN\SUN 2025-09-23 11-10-25\022-P1-A2-zzj-6-93-minor-as.D
Sample Name: zzj-6-93-minor-as

Signal 1: DAD1 A, Sig=254,4 Ref=360,100

Signal 2: DAD1 B, Sig=210,4 Ref=360,100

Peak #	RetTime [min]	Type	Width [min]	Area [mAU*s]	Height [mAU]	Area %
1	8.752	MM	0.5991	645.78302	17.96574	5.8127
2	11.498	MM	0.8971	1.04640e4	194.40518	94.1873

Totals : 1.11098e4 212.37092

Signal 3: DAD1 D, Sig=230,4 Ref=360,100

*** End of Report ***

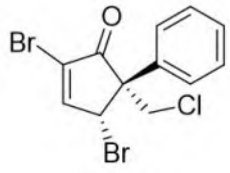

10
